# Catalytic Enantioselective Multicomponent Reactions of Sulfoxonium Ylides Enabled by a Formal Rearrangement—A Versatile Entry to Enantioenriched α‐Sulfanyl Carbonyl Compounds

**DOI:** 10.1002/anie.2074669

**Published:** 2026-05-17

**Authors:** Nicolò Santarelli, Pietro Pecchini, Nunzio Matera, Andrea Pellegrini, Riccardo Fabbri, Irati Celada Cubero, Leire Navarro Rubio, Cristina Di Pietro, Andrea Mazzanti, Mariafrancesca Fochi, Luca Bernardi

**Affiliations:** ^1^ Department of Industrial Chemistry “Toso Montanari” Center For Chemical Catalysis – C3 and INSTM RU Bologna Alma Mater Studiorum – University of Bologna Bologna Italy

**Keywords:** asymmetric catalysis, multicomponent reactions, organocatalysis, rearrangment, sulfur ylides

## Abstract

Sulfur ylides have emerged as versatile carbenoids for catalytic enantioselective X–H insertion reactions (X = C, N, O, and S), while offering a better safety profile compared to traditional diazo‐based metal carbene precursors. However, the realization of valuable multicomponent reactions (MCRs) with sulfur ylides has been so far out of reach. Here, we report the enantioselective MCR of sulfoxonium ylides, aldehydes, and thiols catalyzed by a chiral phosphoric acid. Departing from carbenoid reactivity, the reaction pathway entails two sequential but nonoverlapping catalytic cycles, where the assembly of the components is followed by a delayed stereodetermining rearrangement across the central C─C bond of the molecule. The organocatalytic MCR delivers β‐hydroxy‐α‐sulfanyl carbonyl products as single anti‐diastereoisomers and generally in high yields and enantioselectivities. These products cannot be readily accessed by other catalytic means and are synthetic linchpins to a variety of α‐sulfanyl carbonyl compounds via stereospecific substitutions of their hydroxy group.

## Graphical abstract

A catalytic network embedding a formal rearrangement enables the first catalytic enantioselective multicomponent reaction (MCR) of sulfur ylides. In the presence of a bulky chiral phosphoric acid, the reaction combines sulfoxonium ylides, aldehydes and thiols, and affords synthetically versatile β‐hydroxy‐α‐sulfanyl carbonyl compounds in enantioenriched form.

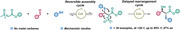

## 1 Introduction

**SCHEME 1:**
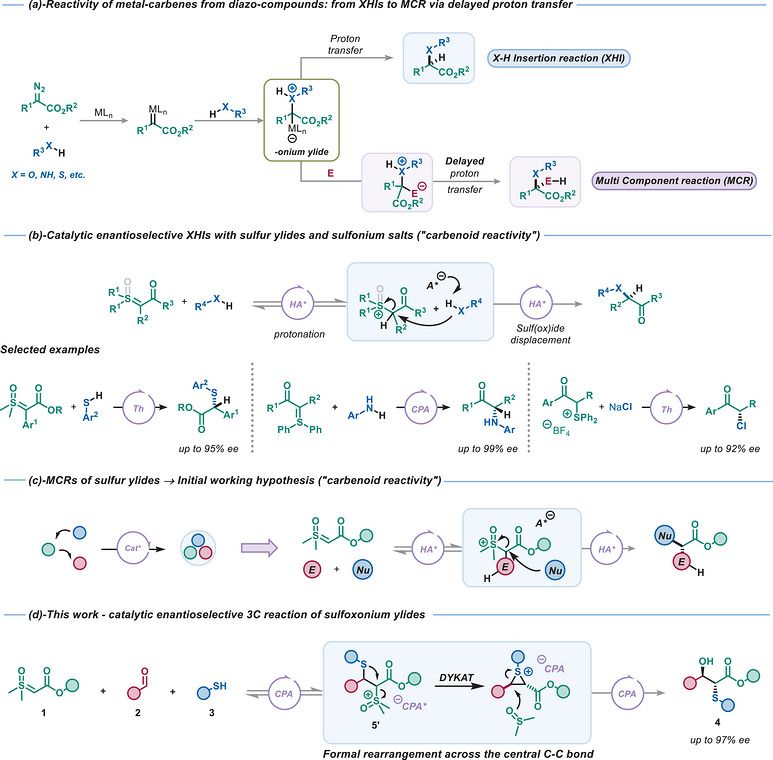
Background and concept. Th = Chiral thiourea; CPA = Chiral phosphoric acid; DYKAT = Dynamic kinetic asymmetric transformation.

Multicomponent reactions (MCRs) are one‐pot procedures in which three or more substrates generate a product whose structure incorporates most atoms of the starting molecules [1]. In the organic chemistry toolbox, MCRs stand out for their capability to build molecular complexity in an efficient and convergent fashion [2, 3]. However, the design and the realization of a new MCR is challenging [4], as the components must react in an orchestrated manner. These challenges are amplified if catalysis and stereocontrol come into play [5, 6, 7]. Yet, rewards can be high. A case in point is the exceedingly versatile MC platform built on electrophilic metal carbenes generated in situ from α‐diazocarbonyl compounds (Scheme 1a) [8, 9, 10]. Here, the classical insertion pathway of metal carbenes into polarized X─H bonds (XHIs) is intercepted by an electrophile. A synergistic combination of an achiral transition metal complex and a chiral catalyst – typically a chiral phosphoric acid (CPA) – delays the proton‐transfer event through the electrophilic trapping of the ‐onium ylide and exerts stereocontrol.

The synthetic utility of sulfur ylides, disclosed in the 1960's for the synthesis of epoxides and cyclopropanes [11, 12, 13, 14], has been extended in the last decades over an impressive range of transformations [15, 16, 17, 18, 19, 20, 21, 22, 23]. In this context, stabilized sulfoxonium ylides have been particularly successful in formal XHIs. Early examples with metal catalysts [24] disclosed alternatives to the traditional XHIs with α‐diazocarbonyl derivatives [25, 26]. Bypassing the safety issues of diazo compounds [27], applications of these transformations in industrial settings have been envisioned [28]. More recently, catalytic enantioselective XHIs of α,α‐disubstituted sulfoxonium ylides using organic catalysts have emerged (Scheme 1b) [29, 30, 31, 32, 33, 34, 35, 36, 37, 38, 39]. These reactions – proceeding via reversible protonation of the ylide followed by enantiodetermining DMSO displacement – have also been extended to sulfonium ylides [40, 41] and sulfonium salts [42, 43]. This chemistry provides unique opportunities in the preparation of α‐functionalized carbonyl compounds in enantioenriched form. However, despite this promising background, sulfur ylides have not been engaged in catalytic enantioselective MCRs so far [44].

**SCHEME 2:**
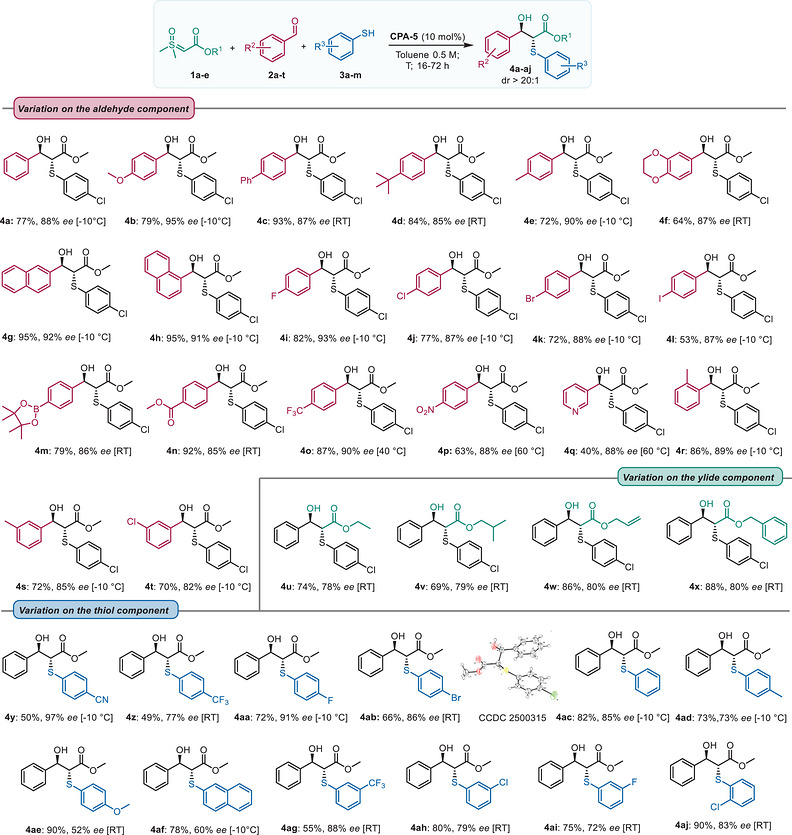
Scope of the catalytic enantioselective MCR. Conditions: **1** (0.1 mmol), **2** (0.25 mmol), **3** (0.25 mmol), **CPA‐5** (10 mol%), toluene (0.5 M), 16–72 h. dr > 20:1 by ^1^H NMR analysis of the crude mixture. Isolated yield after chromatography on silica gel. Enantiomeric excess determined by CSP HPLC analysis.

Motivated by the attractiveness of MCRs and sulfur ylides, we questioned whether it was possible to introduce these compounds in catalytic enantioselective MCRs by leveraging their carbenoid reactivity. We hypothesized that a stabilized sulfoxonium ylide could react with an electrophile, instead of a proton as in the XHIs, affording, after the nucleophilic trapping of the resulting sulfoxonium intermediate, a MCR product (Scheme 1c). Our investigation culminated in the enantioselective 3CR between stabilized sulfoxonium ylides **1**, aldehydes **2**, and thiophenols **3**, catalyzed by CPAs (Scheme 1d). Unexpectedly, experimental inspection of the reaction pathway revealed a fundamental departure from the hypothesized carbenoid mechanism, pointing to a peculiar rearrangement across the central C─C bond of sulfoxonium species **5’** as the stereodetermining step of the process. This MC methodology affords synthetically versatile β‐hydroxy‐α‐sulfanyl carbonyl derivatives **4** bearing two contiguous stereogenic centers in a direct and modular manner. These compounds are not readily accessed via direct enantioselective aldol reactions [45]. Because of the low acidity of α‐sulfanyl carbonyl derivatives, direct enolization processes require designed amides as pronucleophiles and chiral Lewis acid [46, 47] or superbase [48, 49, 50] catalysts.

## 2 Results and Discussion

### 2.1 Reaction Discovery and Development

Our efforts began by examining the reaction between ylide **1a**, benzaldehyde **2a**, and 4‐chlorothiophenol **3a** (Table 1). In toluene at 40°C, and in the absence of a catalyst, the reaction afforded selectively the S‐H insertion [51] product **6a** (entry 1). However, we were delighted to observe that the introduction of the achiral phosphoric acid catalyst **DPP** [(PhO)_2_P(O)OH] could also engage benzaldehyde **2a** in the process. The innate S‐H insertion reactivity of the substrates was overcome—at least in part—by the desired MCR, unlocking access to product **4a** (entry 2). Full diastereoselectivity, favouring the anti‐isomer of **4a**, was observed with **DPP** and in all subsequent experiments. A screening of common organocatalysts showed that only acidic species such as **DPP** could induce the 3C reactivity, while less acidic ones (e.g. thioureas) were ineffective. CPAs [52] were thus the obvious option for the development of the enantioselective MCR.

**TABLE 1:** Selected optimization results.^a^

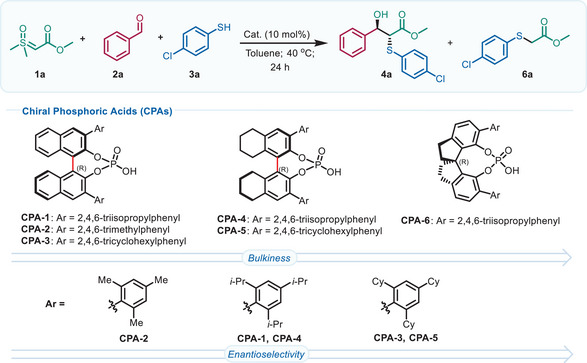						
Entry	Catalyst	2a [equiv]	3a [equiv]	Conc. [M]	Yield [%]	*ee* [%]
1	None	1.5	1.5	0.25	**6a**‐79	n/a
2	**DPP** ^b^	1.5	1.5	0.25	**4a**‐47; **6a**‐15	n/a
3	**CPA‐1**	1.5	1.5	0.25	**4a**‐27	80
4	**CPA‐2**	1.5	1.5	0.25	**4a**‐43	44
5	**CPA‐1**	1.5	1.5	0.5	**4a**‐42	77
6	**CPA‐1**	2.5	2.5	0.5	**4a**‐58	71
7	**CPA‐3**	2.5	2.5	0.5	**4a**‐60	76
8	**CPA‐4**	2.5	2.5	0.5	**4a**‐68	83
9	**CPA‐5**	2.5	2.5	0.5	**4a**‐79	85
10	**CPA‐6**	2.5	2.5	0.5	**4a**‐31	10^c^
11^d^	**CPA‐5**	2.5	2.5	0.5	**4a**‐80	88

^a^ Conditions: the reaction of **1a** (0.10 mmol), **2a** (0.15–0.25 mmol), **3a** (0.15–0.25 mmol) and catalyst (10 mol%) was carried out in toluene (0.2–0.4 mL) at 40 °C for 24 h. Yield was determined by ^1^H NMR analysis of the crude mixture using bibenzyl as internal standard. Only *anti*‐**4a** was detected. Except entries 1 and 2, <15% **6a** was present in the crude mixtures. Enantiomeric excess of **4a** was determined by chiral stationary phase HPLC.

^b^ 20 mol% catalyst.

^c^ *ent*‐**4a** as major enantiomer.

^d^ Reaction performed at –10 °C for 72 h.

A preliminary screening identified catalyst **CPA‐1** bearing bulky 2,4,6‐triisopropylphenyl substituents as a promising lead, furnishing **4a** with a good 80% *ee* but a modest 27% yield (entry 3). The importance of the bulkiness of the 3,3’‐substituents appeared clearly when the less hindered 2,4,6‐trimethylphenyl derivative **CPA‐2** was tested (entry 4). With **CPA‐1**, we were pleased to observe a noticeable increase in the reaction yield for higher concentrations, without a significant erosion in the enantioselectivity (entry 5). At this stage, we detected in the mixture the disulfide **7a**, which we initially attributed to parasitic dimerization of **3a** due to e.g. adventitious oxygen or reaction with the DMSO released during the MCR. Although these hypotheses proved later to be erroneous (see the section Mechanistic studies), the detection of the disulfide **7a** suggested increasing the equivalents of **3a**. Indeed, a larger excess of **3a** – and of **2a** – led to a considerable improvement in yield, accompanied by a small decrease in enantioselectivity (entry 6). We then tested the sterically more demanding 2,4,6‐tricyclohexylphenyl catalyst **CPA‐3**. Gratifyingly, a small improvement in the enantioselectivity was observed (entry 7), thus confirming the positive relationship between bulkiness and enantioinduction. An investigation on the catalyst chiral backbone showed that the (*R*)‐H_8_‐BINOL derivative **CPA‐4** performed remarkably better than its fully aromatic counterpart **CPA‐1** (compare entries 8 and 6). This result led to a focus on **CPA‐5**, which combines 2,4,6‐tricyclohexylphenyl groups and the (*R*)‐H_8_‐BINOL core. Pleasingly, **CPA‐5** resulted in an additional improvement, affording product **4a** in 79% yield and 85% *ee* (entry 9). We then tested the (*R*)‐SPINOL derivative **CPA‐6**. This catalyst promoted the reaction but afforded the product with very low enantiomeric excess (10% *ee*, entry 10). In SPINOL derivatives, the substituents are closer than in their BINOL counterparts, resulting in smaller and less flexible chiral pockets [53, 54]. Considering also that the more twisted H_8_‐BINOL [55] derivatives **CPA‐4** and **CPA‐5** outperform their BINOL counterparts **CPA‐1** and **CPA‐3**, we speculate that this reaction is better accommodated with catalysts characterized by relatively open and less confined chiral cavities, where the bulky substituents may combine steric effects with stabilizing noncovalent interactions [56]. Finally, a small but noticeable improvement in the enantioselectivity of the reaction, without compromising its yield, was achieved by performing the reaction at a lower temperature (–10°C) for a longer time (entry 11).

### 2.2 Reaction Scope and Synthetic Elaborations

Considering the conditions reported in entry 11, Table 1, we moved to explore the generality of this MCR by varying its three reaction components **1**–**3**. We initially noticed that some thiophenols and aldehydes exhibited reduced reactivity at –10°C. However, satisfactory results could be achieved also for these substrates by tuning the reaction temperature (Scheme 2). Pleasingly, as in the model reaction with **1a**, **2a**, and **3a**, complete diastereoselectivity for the anti‐isomers of products **4** was observed in all the cases.

We first focused on the aldehyde partner **2**. Besides unsubstituted benzaldehyde **2a**, aldehydes bearing electron‐donating/neutral groups on the *para*‐, *meta*‐, and *ortho*‐positions smoothly underwent the 3CR, delivering products **4b**–**e**, **4r** and **4s** in good to excellent yields (72%–93%) and enantioselectivities (85–95% *ee*). Furthermore, the protocol was tolerant toward more sterically demanding poly‐substituted benzaldehydes (**4f**–**h**: 64%–95% yield, 87%–92% *ee*). Similarly, benzaldehydes carrying halides at different positions efficiently delivered products **4i**–**l** and **4t**, except for *para*‐iodobenzaldehyde, which rendered **4l** only in a moderate yield (53%) but with a good enantiomeric excess (87%). Boronic acid ester and ester substituents were well tolerated too, as products **4m** and **4n** were obtained with satisfactory results (79%–92% yield, 85%–86% *ee*). Electron‐poorer substrates necessitated higher temperatures to undergo the reaction. Benzaldehydes bearing *para*‐trifluoromethyl and *para*‐nitro substituents led to formation of products **4o** and **4p** only at 40°C and 60°C, respectively. Likewise, the electron‐poor heteroaromatic nicotinaldehyde evolved into product **4q** only under thermal activation. These higher reaction temperatures did not compromise the enantioselectivities of these reactions (≥ 88% *ee*). Then, the sulfoxonium ylides **1b**–**e** were tested, affording adducts with alkyl (**4u**,**v**), allyl (**4w**), and benzyl (**4x**) esters. Although the enantioselectivities of these reactions (78%–80% *ee*) were slightly lower than the MCR performed with **1a**, products **4u**–**x** carry orthogonally protected carboxylic groups, enhancing the synthetic versatility of products **4**. Finally, we turned our attention to the thiophenol **3** partner. Electron‐deficient thiophenols, such as the *para*‐cyano and *para*‐trifluoromethyl derivatives, led to the corresponding products **4y** and **4z** only in moderate yields (49%–55%). The combination of lower reaction rates, with competing formation of the S‐H insertion side‐products **6** likely due to the increased acidity of these thiols, accounts for these results. Nevertheless, the cyano derivative **4y** was obtained with excellent enantiomeric excess (97% *ee*). In fact, with the exception of 4‐(trifluoromethyl)thiophenol that rendered a modest 77% *ee* in product **4z**, a positive relationship between the electron withdrawing properties of the substituent and enantioselectivity can be evidenced in the reactions with *para*‐substituted thiophenols (**4y** (NC–): 97% *ee*; **4aa**, **4a**, **4ab** (F/Cl/Br): 86%–91% *ee*; **4ac** (H): 85% *ee*; **4ad** (Me): 73% *ee*; **4ae** (MeO): 56% *ee*). Finally, 2‐naphthalenethiol, and different *meta*‐ and *ortho*‐substituted thiophenols, afforded the corresponding products **4af**–**aj** with moderate to good yields (55%–90%) and variable enantioselectivities (60%–88% *ee*). Additional experiments with aliphatic aldehydes and thiols, and with different stabilized ylides, indicated that, at the current stage, the scope of the enantioselective MC protocol is limited to (hetero)aromatic aldehydes, thiophenols, and sulfoxonium ylide esters (see the Supporting Information, page S55). X‐ray analysis of the *para*‐bromothiophenol adduct **4ab** [57], and comparison with literature data for **4ac** [45], led to the assignment of the relative and absolute configuration of the adducts **4** as (2*R*,3*R*).

**SCHEME 3:**
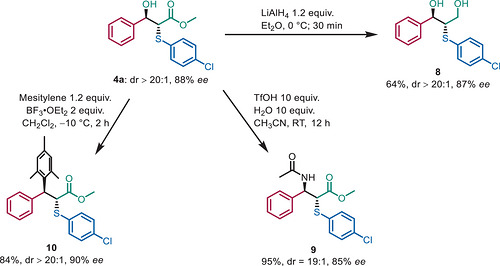
Synthetic elaborations on the catalytic product **4a**.

We investigated the synthetic versatility of products **4** by first reducing the carbonyl group of adduct **4a** with lithium aluminium hydride [45], which rendered the 1,3‐dihydroxy‐2‐sulfanyl derivative **8** (Scheme 3). Then, we focused on the nucleophilic substitutions of the hydroxyl group of compounds **4** harnessing the anchimeric assistance of the sulfanyl moiety. In β‐aryl‐β‐hydroxy sulfanyls, the polarizable lone pairs on the sulfur atom facilitate the formation of episulfonium ion intermediates under acidic conditions, enabling stereospecific substitutions of the β‐hydroxyl group with a variety of nucleophiles [58]. To test the feasibility of these stereospecific substitutions on our products, compound **4a** was subjected to two representative transformations, which delightfully enabled the introduction of the acetamide (**9**) and mesitylene (**10**) functionalities in high yields (Scheme 3). The Friedel–Crafts reaction with mesitylene proceeded with full stereospecificity, while a minor erosion of the stereochemical integrity of the catalytic product **4a** was observed in the Ritter reaction. This validates the use of MC adducts **4** as synthetic linchpins for stereodefined α‐sulfanyl carbonyl derivatives [59], including highly sought β‐amino structures [49, 60].

### 2.3 Mechanistic Studies

**SCHEME 4:**
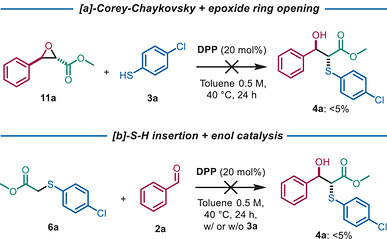
(a) Control experiment with the putative epoxide intermediate **11a**. (b) Control experiment with S‐H insertion product **6a**.

The multicomponent nature of the reaction leaves the stage open to several mechanistic scenarios. Initially, two hypotheses were examined: a Corey–Chaykovsky epoxidation followed by ring‐opening, and an S‐H insertion reaction giving compounds **6**, followed by enol catalysis [61] (see the Supporting Information, page S21). However, the putative epoxide intermediates **11** were never detected in the reaction mixtures, and control experiments with epoxide **11a** indicated a complete lack of reactivity (Scheme 4a). Similarly, no reactivity was observed when the S‐H insertion product **6a** was subjected to the reaction conditions, likely due to insufficient acidity (Scheme 4b).

**SCHEME 5:**
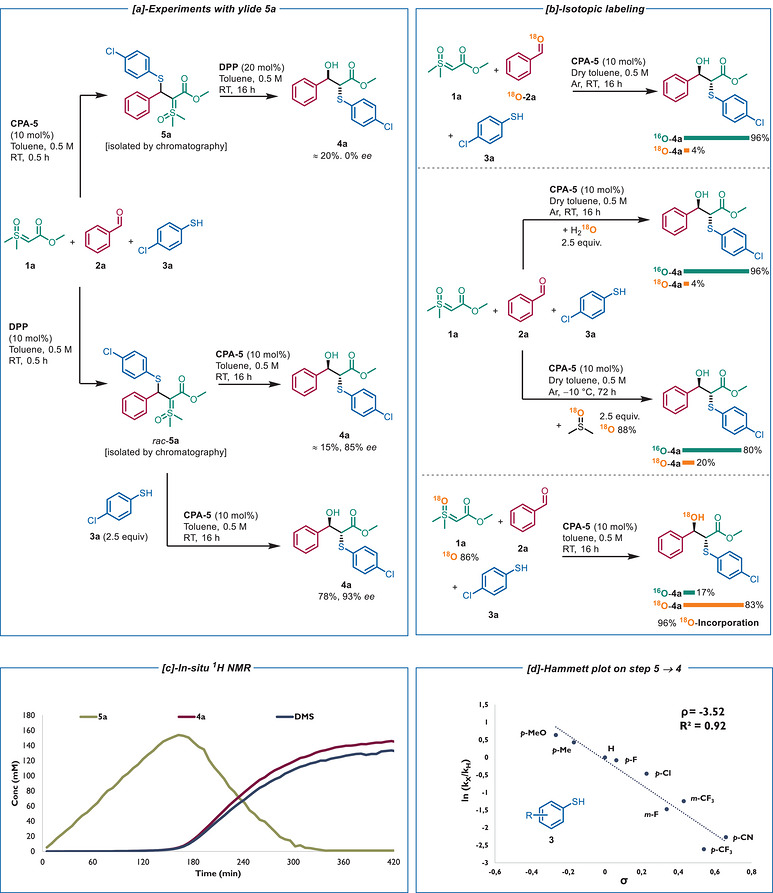
Selected mechanistic experiments. (a) The α,α‐disubstituted ylide **5a** gives the product **4a** in the presence of thiol **3a**. Formation of **5a** is not stereodetermining. (b) The benzylic oxygen atom of product **4a** stems from the dimethylsulfoxonium portion of the ylide **1a**, and not from benzaldehyde **2a** or water. Isotopic ratios determined by ESI‐MS. (c) Conditions: **1a** (0.1 mmol), **2a** (0.25 mmol), **3a** (0.25 mmol), **CPA‐5** (10 mol%), 1,3,5‐trimethoxybenzene as internal standard (0.033 mmol), CDCl_3_ (0.2 M), NMR tube, RT. ^1^H NMR analysis every 3.5 min. Formation of **4a** parallels DMS and starts when [**5a**] ~ [**5a**]_max_. (d) Conditions: **1a** (0.1 mmol), **2a** (0.25 mmol), **3** (0.25 mmol), **CPA‐5** (10 mol%), 1,3,5‐trimethoxybenzene as internal standard (0.033 mmol), CDCl_3_ (0.2 M), NMR tube, RT. ^1^H NMR analysis every 3.5–6 min. The **5** → **4** process is faster with electron‐donating substituents on thiophenol **3**.

These possibilities were thus ruled out and, as initially hypothesized (Scheme 1c), a plausible reaction mechanism appeared to align with the typical carbenoid reactivity of stabilized sulfoxonium ylides [62]. A first hint suggesting a different mechanism came from the identification of the α,α‐disubstituted sulfoxonium ylides **5** as main products in the reactions with electron poor aldehydes at –10°C, where formation of adducts **4** occurred only at higher temperatures (e.g. **4o**–**q**, see Scheme 2). Moreover, for short reaction times, ylides **5** were observed also for other substrates. To examine the role of these compounds in the catalytic MCR, we treated **5a**—isolated from a **CPA‐5** catalyzed reaction performed for a short time—with the achiral **DPP** catalyst (Scheme 5a). This experiment resulted in extensive degradation of **5a**, due to its relatively labile nature, but also in the low yield formation of adduct **4a**. Importantly, **4a** was found to be racemic. In a complementary test, a racemic sample of **5a** was prepared using the achiral catalyst **DPP** and subsequently treated with catalyst **CPA‐5**. This reaction rendered **4a** in low yield, but with an enantiomeric excess comparable to the enantioselective protocol. Moreover, a third experiment showed that the efficiency of the conversion of *rac*‐**5a** to enantioenriched **4a** is dramatically enhanced by the presence of thiophenol **3a** in the mixture. Thus, it could be concluded that, in the presence of thiophenol **3a**, the α,α‐disubstituted ylide **5a** evolves proficiently to the MC product **4a**, and that formation of **5a** does not govern the enantioselectivity of the process. Although informative, these experiments did not fully elucidate the role of **5a** in the reaction. **5a** was proved to be in equilibrium with the starting substrates **1a**–**3a** (see the Supporting Information for cross‐over experiments, page S30). Thus, **5a** could be an off‐cycle species in the catalytic cycle, and the three components react via an enantioselective carbenoid‐like pathway. As such, we envisioned that experiments with isotopic labels designed to investigate the origin of the benzylic oxygen atom could be conclusive (Scheme 5b). The absence of ^18^O incorporation in **4a** when using ^18^O‐labeled benzaldehyde **2a** and 2.5 equivs of H_2_^18^O in the reaction ruled out reliably the carbenoid‐like pathway and excluded water as the benzylic oxygen source. Conversely, the partial incorporation of ^18^O in **4a** when 2.5 equivs of ^18^O‐DMSO were used suggested that the benzylic oxygen atom of product **4a** could stem from the dimethylsulfoxonium portion of the ylide. This hypothesis proved correct: a nearly quantitative incorporation of ^18^O in **4a** was observed when labelled ylide ^18^O‐**1a** was employed in the reaction. We also monitored the reaction progress by an in situ ^1^H NMR experiment (Scheme 5c). This experiment revealed, as suspected, that formation of **5a** is followed by its disappearance and concomitant accumulation of product **4a**, pointing to **5a** as reaction intermediate. Another key piece of information gained from this experiment was the detection of dimethylsulfide (DMS) in the mixture, with a kinetic profile paralleling the formation of **4a**. Moreover, it was observed that formation of **4a** and DMS does not start at t_0_, but only after about 150 min, when [**5a**] has reached its maximum ([**5a**] ~ [**5a**]_max_). In other words, **4a** and DMS are generated only once production of **5a** has halted, and ylide **1a** has been consumed. This lack of overlap between the formation of α,α‐disubstituted ylides **5** and their conversion to products **4** proved to be general and allowed the study of the two rates with different aldehydes **2** and thiols **3** using in situ ^1^H NMR experiments (see the Supporting Information, page S47). Amongst the data gathered with these experiments, the Hammett plot built using the rates of the conversion **5** → **4** in the reactions with different thiophenols **3**, appeared particularly meaningful (vide infra). A strongly negative linear relationship between the Hammett σ parameter of the thiophenol substituents and the reaction rate *(ρ* = –3.52, Scheme 5d) clearly indicated that substituents capable of stabilizing a positive charge at the sulfur atom expedite dramatically the conversion of **5** to **4**.

**SCHEME 6:**
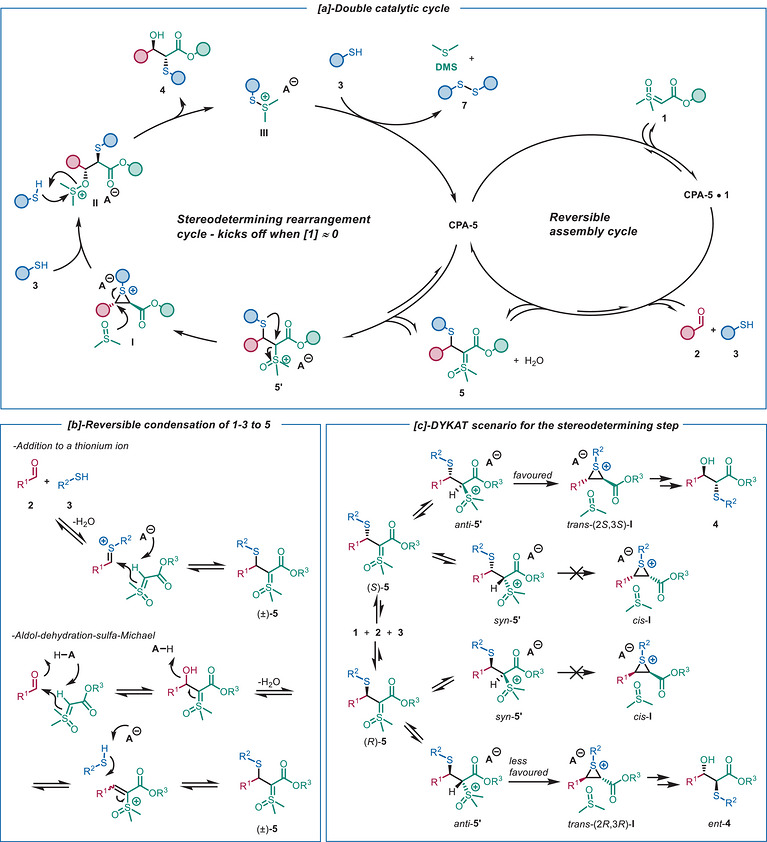
(a) Double catalytic cycle (assembly cycle and rearrangement cycle) for the MCR. (b) Mechanistic hypotheses for the formation of ylide **5**. (c) DYKAT scenario for the stereodetermining conversion of ylide **5** to episulfonium **I**.

Putting all pieces of this mechanistic puzzle together, we landed with a plausible double catalytic cycle for the process (Scheme 6a). Thus, reversible formation of **5** by condensation of **1**, **2,** and **3** in the presence of **CPA‐5** (the “*assembly cycle*”) is followed by the diastereo‐ and enantio‐determining evolution of **5** into product **4** (the “*rearrangement cycle*”).

In the nonstereodetermining assembly cycle, the racemic α,α‐disubstituted ylide **5** results either from attack of the complex between ylide **1** and **CPA‐5** to an elusive thionium ion [63], or via a multistep process–aldol, dehydration, sulfa‐Michael–evoking the chemistry of α‐diazocarbonyl compounds [64] (see Scheme 6b and the Supporting Information, page S26). Considering that sulfur ylides tend to give strong interactions with Brønsted acid catalysts [33, 34, 41, 65], we speculate that the high affinity of **CPA‐5** for the starting ylide **1** – vs the α,α‐disubstituted ylide **5** – confines **CPA‐5** in the assembly cycle as long as substantial amounts of ylide **1** are present in the mixture (Scheme 6a). Indeed, a control experiment demonstrated that ylide **1a** inhibits the **CPA‐5** catalyzed conversion from **5a** to **4a** (see the Supporting Information, page S30).

The diastereo‐ and enantio‐determining conversion of **5** to **4** starts with the reversible protonation of the ylide **5** by **CPA‐5** providing **5’**and triggering the rearrangement across the central C─C bond of the molecule (Scheme 6a). In a DYKAT scenario [66] (Scheme 6c) entailing rapid racemization of **5**, only the two anti‐isomers of **5’** undergo ring‐closure to *trans*‐episulfoniums **I** [67, 68, 69, 70, 71], given the full antidiastereoselectivity of the reaction, and formation of the (2*S*,3*S*)‐**I** isomer is favored over its enantiomeric counterpart, accounting for the major enantiomer of the product **4**. The Hammett plot reported in Scheme 5d is fully consistent with the formation of the episulfonium ion **I** as the rate determining step of the rearrangement cycle and reinforces the plausibility of this DYKAT scenario. The formal rearrangement is then completed (Scheme 6a) via the ring‐opening of episulfonium **I** by the oxygen atom of the just‐displaced DMSO [72], resulting in intermediate **II**. Another molecule of thiophenol **3** – or another nucleophile such as water [73]—then comes into play, scavenging the dimethylsulfonium group from **II**, and affording the enantioenriched MC product **4** as well as the S‐(dimethylsulfonium)sulfide **III**. Formation of DMS and of disulfide **7** releases **CPA‐5** closing the rearrangement cycle. Although, under certain conditions, derailments of the rearrangement cycle toward ring‐opening of **I** by other nucleophiles were observed (see the Supporting Information, page S8 and S25) [71], the preference of **I** to suffer attack by the DMSO oxygen versus exogenous nucleophiles present in the reaction mixture – especially thiophenols **3** – seems surprising. Considering also that only a moderate level of ^18^O incorporation was detected even by using an excess of exogenous ^18^O‐DMSO (Scheme 5b), the ring closure of **5’** to the episulfonium ion **I**, and the ring opening of **I** by “endogenous” DMSO, appears to be intimately linked processes, and not independent reactions. Indeed, a cross‐over experiment with labelled ylide ^18^O‐**1a** and a nonlabelled ylide with a different ester group (allyl: **1d**) did not show any evidence of DMSO scrambling, that is, incorporation of ^18^O was observed exclusively in **4a** and not in product **4w** derived from nonlabelled ylide **1d** (see Supporting Information, page S44).

## 3 Conclusion

Chiral phosphoric acid catalysts (CPA) promote enantioselective MCRs between stabilized sulfoxonium ylides **1**, aldehydes **2**, and thiophenols **3** [74], overtaking the innate S‐H insertion reactivity of these sulfur ylides with thiols. The catalytic MC process affords β‐hydroxy‐α‐sulfanyl carbonyl derivatives **4** – otherwise difficult to obtain directly through asymmetric catalysis – as single antidiastereoisomers, in moderate to excellent yields (up to 95% yield) and enantioselectivities (up to 97% *ee*). The anchimeric assistance offered by the sulfur atom makes it possible for stereospecific substitutions of the β‐hydroxyl group with different nucleophiles. The typical carbenoid reactivity of sulfoxonium ylides did not reconcile with the experimental findings of this MCR, prompting an extensive exploration of mechanistic scenarios. Ultimately, a reaction network embedding two nonoverlapping sequential catalytic cycles and a formal rearrangement appears to be the most convincing option. The departure of this chemistry from both the catalytic pathways typical of α‐diazocarbonyl compounds, and of the XHI reactions of sulfur ylides, is apparent. We are eagerly exploring the synthetic possibilities offered by this uncommon and serendipitously discovered catalytic network.

## Author Contributions

**Pietro Pecchini**: conceptualization, investigation, writing – original draft, methodology, validation, writing – review and editing, supervision, visualization. **Irati Celada Cubero**: investigation, methodology. **Nunzio Matera**: conceptualization, investigation, writing – review and editing, writing – original draft, methodology, visualization. **Luca Bernardi**: conceptualization, funding acquisition, writing – original draft, writing – review and editing, project administration, supervision, methodology, visualization. **Andrea Mazzanti**: data curation, writing – review and editing, methodology, visualization. **Leire Navarro Rubio**: investigation, methodology. **Cristina Di Pietro**: investigation, methodology. **Riccardo Fabbri**: investigation, methodology. **Nicolò Santarelli**: conceptualization, investigation, writing – original draft, writing – review and editing, methodology, validation, supervision, visualization. **Mariafrancesca Fochi**: writing – review and editing, project administration, resources, methodology. **Andrea Pellegrini**: conceptualization, writing – review and editing, methodology.

## Conflicts of Interest

The authors declare no conflicts of interest.

## Supporting information



The authors have cited additional references within the Supporting Information [75, 76, 77, 78, 79, 80, 81, 82, 83, 84, 85, 86, 87, 88, 89].**Supporting File 1**: anie72697‐sup‐0001‐SuppMat.pdf.

**Supporting File 2**: anie72697‐sup‐0002‐Data.zip.

## Data Availability

The data that support the findings of this study are available from the corresponding author upon reasonable request.
