# Supplementary material for: Catalytic Enantioselective Multicomponent Reactions of Sulfoxonium Ylides Enabled by a Formal Rearrangement—A Versatile Entry to Enantioenriched α‐Sulfanyl Carbonyl Compounds
Source: Angew Chem Int Ed Engl. 2026 May 17;65(28):e2074669. doi: 10.1002/anie.2074669 (PMC13340488; doi:10.1002/anie.2074669)
Supplement: Supplementary file 1 — The authors have cited additional references within the Supporting Information [75, 76, 77, 78, 79, 80, 81, 82, 83, 84, 85, 86, 87, 88, 89].Supporting File 1: anie72697‐sup‐0001‐SuppMat.pdf. [file ANIE-65-e2074669-s002.pdf]

Supporting Information

Catalytic enantioselective multicomponent reactions of sulfoxonium ylides enabled by a formal rearrangement – a versatile entry to enantioenriched α-sulfanyl carbonyl compounds

*Nicolò Santarelli†, Pietro Pecchini†, Nunzio Matera, Andrea Pellegrini, Riccardo Fabbri, Irati Celada Cubero, Leire Navarro Rubio, Cristina Di Pietro, Andrea Mazzanti, Mariafrancesca Fochi, Luca Bernardi**

† Equal contribution

Department of Industrial Chemistry "Toso Montanari", Center for Chemical Catalysis – C3, and INSTM RU Bologna, Alma Mater Studiorum – University of Bologna, V. Gobetti 85, 40129 Bologna (Italy). E-mail: luca.bernardi2@unibo.it

Table of contents

General methods and materials S1

Selected optimization results S2

Reaction side-products, coproducts, and intermediates S8

α,α-Disubstituted ylides **5** S8

S-H insertion products **6** S13

Disulfides **7** S13

Catalyst-trapped adducts **12** S14

2,3-Bis(arylthio) carbonyl derivatives **13** S18

Mechanistic studies S21

Hypothesis 1: Corey-Chaykovsky epoxidation followed by ring-opening S21

Hypothesis 2: X-H insertion reactions followed by enol catalysis S22

Hypothesis 3: addition to the aldehyde followed by DMSO displacement (“carbenoid reactivity”) S24

Mechanistic proposal S25

Proposal overview S25

Possible pathways for the formation of α,α-disubstituted ylide **5** S26

Enantiodetermining step S28

Rearrangement of **5** to **4**: promoters and inhibitors S30

Cross-over experiments between **5** and the substrates – racemization of ylide **5** S30

Identification of the oxygen source of the hydroxyl moiety of products **4** S36

Reaction kinetics and Hammett’s plots by in situ ^{1}H NMR S47

Limitations of the methodology S55

Experimental details S57

General procedures S57

General procedure **A**: synthesis of ylides **1a-e** S57

General procedure **B**: catalytic enantioselective synthesis of compounds **4** via MCR at -10 °C S57

General procedure **C**: catalytic enantioselective synthesis of compounds **4** via MCR at RT S58

General procedure **D**: catalytic enantioselective synthesis of compounds **4** via MCR at 60 °C S58

General procedure **E**: synthesis of racemic reference compounds **4** S59

Synthesis of catalyst **CPA-5** [(*R*)-H_8-TCYP] S60

Synthesis and characterization of the catalytic products **4** S64

Synthetic elaborations S178

Reduction of the ester moiety S178

Ritter reaction via episulfonium ion S181

Friedel-Crafts reaction with mesitylene S184

X-ray data for compound **4ab** S187

General methods and materials

The ^{1}H, ^{13}C, ^{19}F and ^{31}P NMR spectra were recorded on a Varian Mercury 400 MHz, Varian Inova 600 MHz or a Bruker Ascend Advance Neo 600 MHz, equipped with a Prodigy cryoprobe, spectrometer. 2D NMR spectra (gCOSY, gHSQC, gHMBC) were recorded on a Bruker Ascend Advance Neo 600 MHz spectrometer equipped with a Prodigy cryoprobe. Chemical shifts (δ) are reported in ppm relative to residual $CHCl_3$ signals for ^{1}H (7.26 ppm) and $CDCl_3$ for ^{13}C (77.0 ppm) NMR, using $CF_3C_6H_5$ as external reference calibrated at -63.72 ppm for ^{19}F NMR, and 85% H_3PO_4 as external reference calibrated to 0 ppm for ^{31}P NMR. Chromatographic separations were carried out using a Büchi Chromatography system Pure C-815 Flash, with FlashPure EcoFlex cartridge (4 to 40 g), 50 µg irregular. High Resolution Mass Spectra (HRMS) were recorded on a Waters Synapt MALDI Q-Tof G2S spectrometer. HPLC-MS analysis for mechanistic investigations were conducted with an Agilent 1260 infinity II analytical instrument with quaternary pump (800 bar) and MS module Infinity Lab LC/MSD iQ series with single quadrupole, equipped with a reverse phase column Infinity Lab Poroshell 120 EC-C18, 4.6 x 150 mm, 2.7 µm. Optical rotations were measured on a JASCO P 2000 IRM equipped with a sodium lamp and are reported as follows: $[\alpha_D]_\lambda^T$ (c = g/100 mL in chloroform). The enantiomeric excess (ee) of the products was determined using Agilent 1100 or 1200 series HPLC instruments equipped with chiral stationary phase columns, and a UV detector operating at 230 or 254 nm. Enantiopure samples (of the major enantiomer) for X-ray analysis were prepared using Waters™ 600 preparative HPLC equipped with Waters™ 2487 dual λ absorbance detector and 600 controller, and a preparative chiral AD-H stationary phase column (250 mm x 20 mm), 5 µm. X-ray data were acquired on a Bruker APEX-2 diffractometer. Analytical grade solvents and commercially available reagents were used as received, unless otherwise stated. Dry THF was obtained by distillation from Na/benzophenone before use or through SPS distillation system Pure Process Technology model PPT-SPS-5-CM. Solvents dryness was measured with a Karl-Fischer system, model Metrohm Eco Coulometer w/ gen. Electrode. The chemicals used for the reactions are commercial, provided by BLD pharm, Fluorochem, TCI, Thermo-Fischer, Sigma-Aldrich/Merck. Racemic samples (references for HPLC analysis) were obtained using diphenyl phosphoric acid (**DPP**) as catalyst, as outlined in the relevant section. The absolute and relative configuration of product **4ab** was assigned by X-ray analysis, and of **4ac** by comparison with literature data. The absolute and relative configuration of the remaining compounds **4** were assigned by analogy.

Selected optimization results

This section collects and summarizes a selection of the experiments performed during the optimization process. At the outset, it was decided to use 4-chlorothiophenol **3a** as a representative substrate. **3a** is solid at RT. It is easier to handle and less malodourous compared to other, more volatile, thiophenols. The reactions were performed in a screw-capped vial equipped with a magnetic stirring bar, without precautions to exclude moisture or air, using untreated solvents. Ylide **1a** and catalysts were weighed in the vial. The solvent was added, followed by aldehyde **2** and thiophenol **3**. After stirring for the given time, the solvents were evaporated and the residue analyzed by ^{1}H NMR ($CDCl_3$) to determine conversion, diastereomeric ratio, and yield (bibenzyl or dibromomethane as internal standard). In all cases, only the *anti*-diasteroisomers **4** were detected. The enantiomeric excess of product **4** was determined by CSP HPLC analysis (see section: Synthesis and characterization of the catalytic products **4**).

***Table S1**: Preliminary tests with different organocatalysts – feasibility of the reaction*

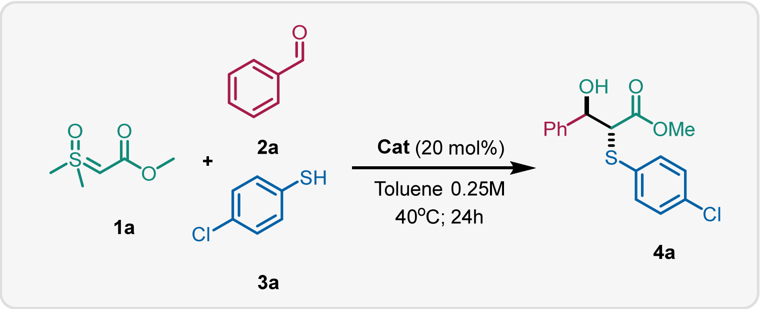

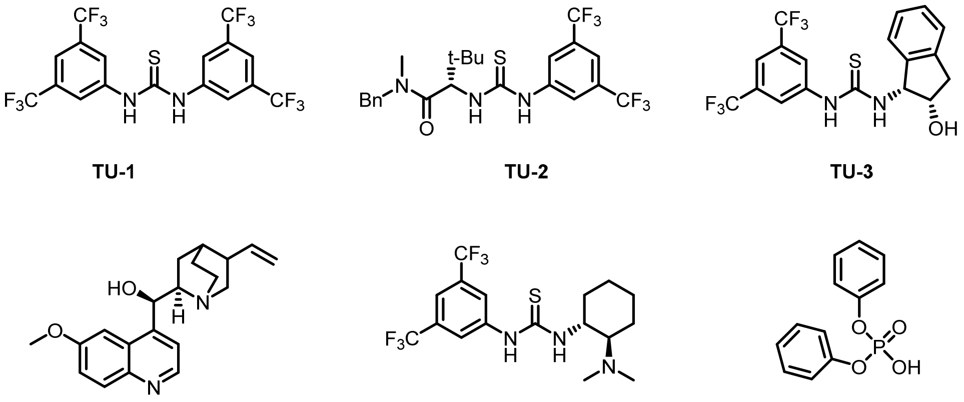

Entry[a]	*Catalyst*	*Yield **4a**[b] (%)*
1	**TU-1**	<5
2	**TU-2**	<5
3	**TU-3**	<5
4	**QN-1**	<5
5	**TU-4**	<5
6	**DPP**	47

[a] Standard conditions: 0.1 mmol **1a**, 0.15 mmol **2a**, 0.15 mmol **3a**, 0.02 mmol catalyst**,** 0.4 mL toluene, 40 °C, 24 h. [b] Determined by ^{1}H-NMR using dibenzyl as internal standard.

As evident from *Table S1*, only the phosphoric acid catalyst (**DPP**) promotes the desired MCR (entry 6).

First set of Chiral Phosphoric Acid catalysts

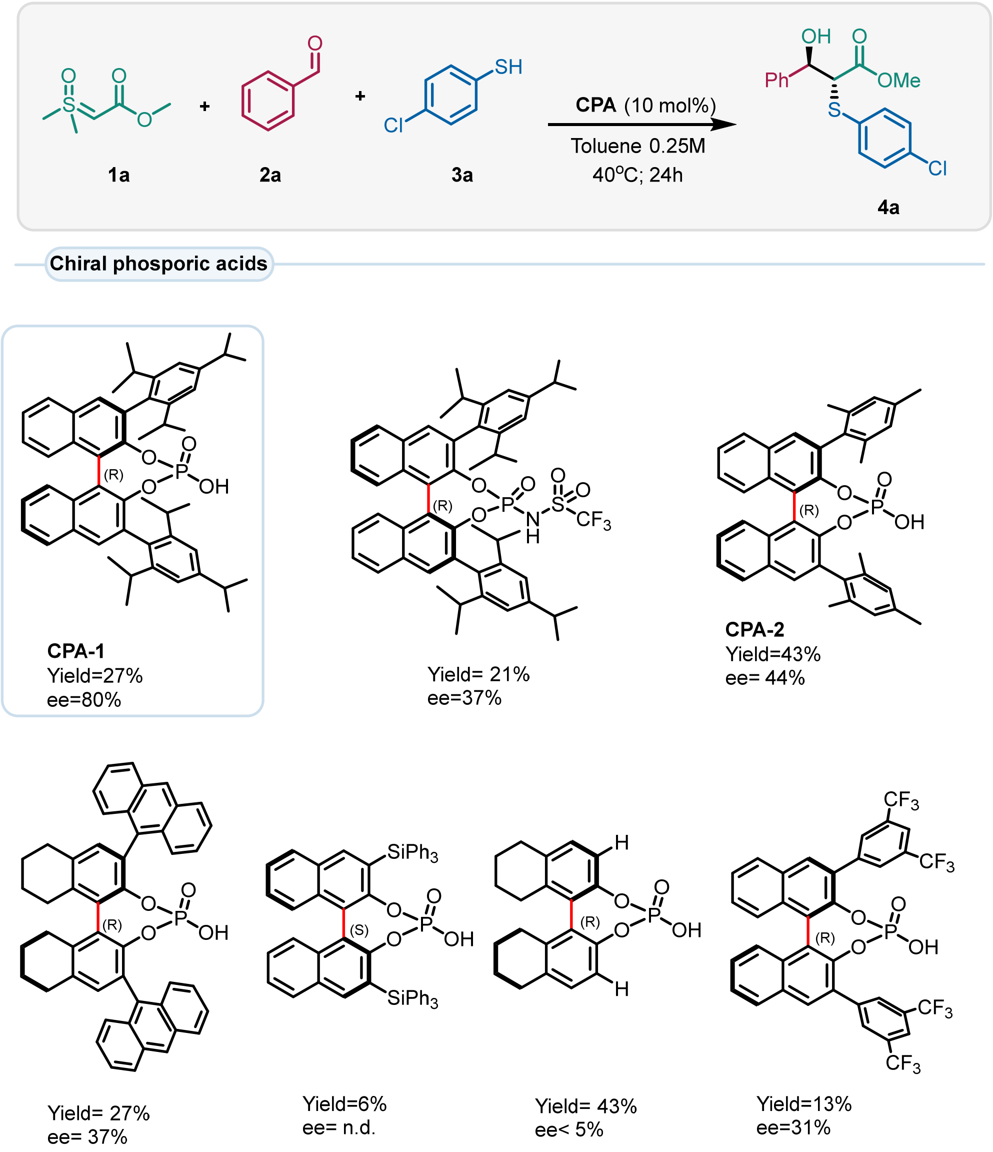

Scheme S1 Standard conditions: 0.1 mmol **1a**, 0.15 mmol **2a**, 0.15 mmol **3a**, 0.01 mmol catalyst, 0.4 mL toluene, 40 °C, 24 h. Yield determined by ^{1}H-NMR using dibenzyl as internal standard. ee determined by CSP HPLC. n.d.: not determined.

Among the catalysts of this first set, **CPA-1** ((*R*)-TRIP) stands out as the most effective catalyst in terms of enantiomeric excess (Scheme S1).

Second set of Chiral Phosphoric Acid catalysts

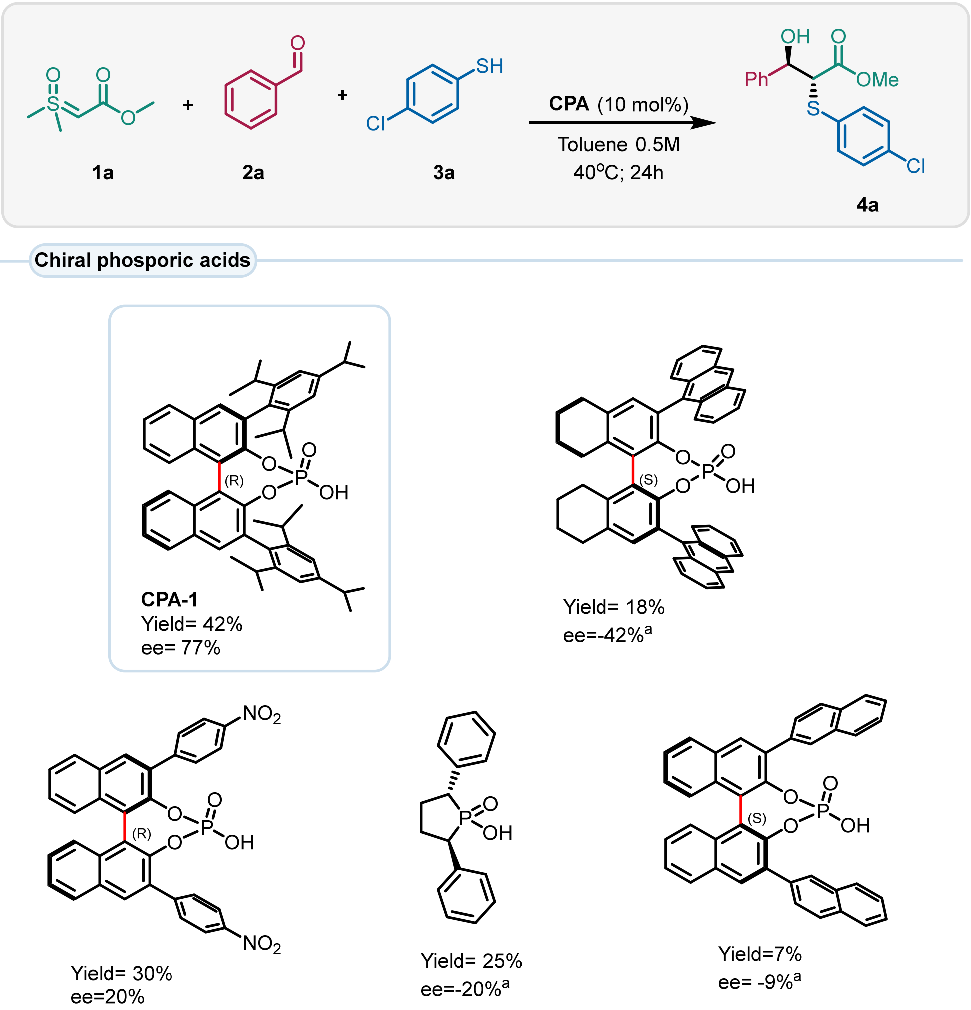

Scheme S2 Standard conditions: 0.1 mmol **1a**, 0.15 mmol **2a**, 0.15 mmol **3a**, 0.01 mmol catalyst, 0.2 mL toluene, 40 °C, 24 h. Yield determined by ^{1}H-NMR using dibenzyl as internal standard. ee determined by CSP HPLC. [a] Major enantiomer: (2*S*,3*S*)-**4a**.

Increasing the concentration from 0.25 M to 0.5 M, improved the yield for **CPA-1**, but not for the 9-anthracenyl derivative (compare results in Scheme S1 and Scheme S2). Other catalysts were tested too. However, none of these structures provided results better than with **CPA-1**.

***Table S2**: Aromatic solvents*

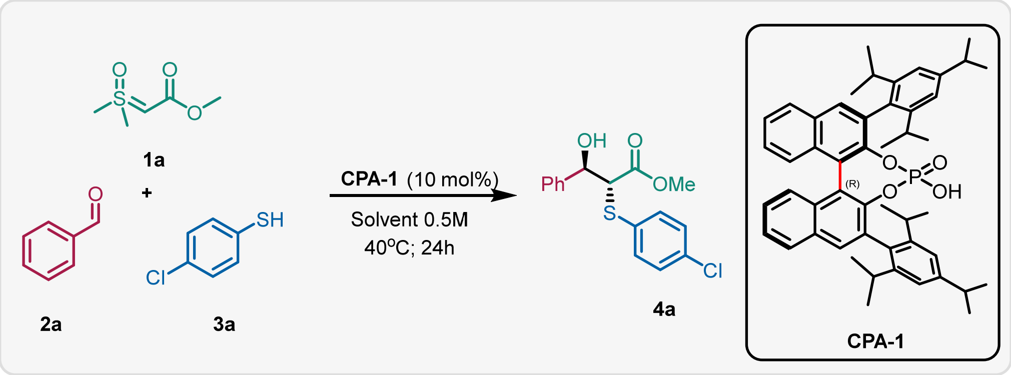

Entry[a]	*Solvent*	*Yield*[b] *(%)*	*ee*[c] *(%)*
1	Toluene	42	77
2	Anisole	56	67
3	Chlorobenzene	54	66
4	Fluorobenzene	58	66
5	*para*-Xylene	54	65
6	Cumene	48	64
7	Xylenes mixture	55	63
8[d]	Toluene	58	71

[a] Standard conditions: 0.1 mmol **1a**, 0.15 mmol **2a**, 0.15 mmol **3a**, 0.01 mmol **CPA-1**, 0.2 mL solvent, 40 °C, 24 h. [b] Determined by ^{1}H-NMR using dibenzyl as an internal standard. [c] Determined by CSP HPLC. [d] Variation from standard conditions: 0.25 mmol **2a**, 0.25 mmol **3a**.

Several solvents were tested in the reaction but did not give promising results. The focus was set on a screening of aromatic solvents (Table S2). However, none of these solvents tested gave results better than toluene (entry 1).

In parallel investigations with 4-fluorothiophenol **3d**, performed to follow the reaction course by ^{19}F NMR, we detected the corresponding disulfide **7d** already at the first stages of the reaction (see Section Disulfide). Moreover, other experiments showed that an excess of benzaldehyde **2a** could be helpful to the reaction too. These observations led us to use larger amounts of thiol **3a** and aldehyde **2a** in the multicomponent reaction (2.5 equivalents each, entry 8). These conditions gave lower enantiomeric excess but ensured a more acceptable yield value and were applied in subsequent experiments.

***Table S3**: Third set of Chiral Phosphoric Acids: different chiral scaffolds and hindered aromatic groups*

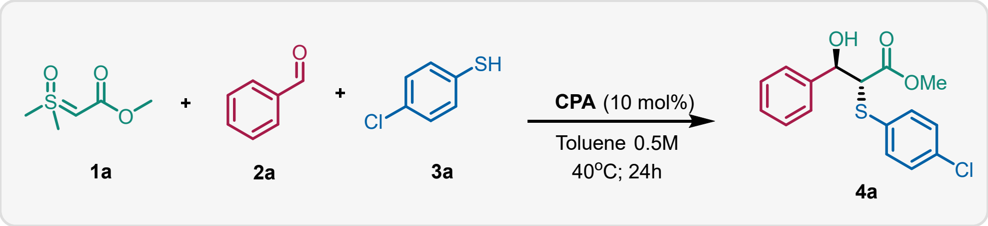

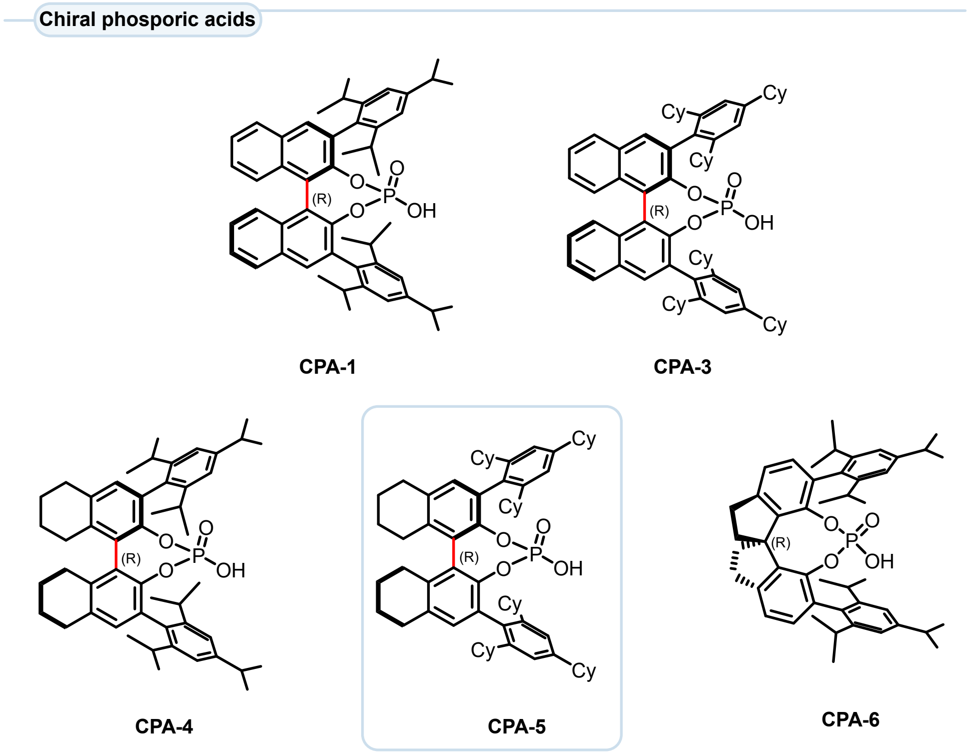

Entry[a]	*CPA*	*Yield*[b] *(%)*	*ee*[c] *(%)*
1	**CPA-1**	58	71
2	**CPA-3**	60	76
3	**CPA-4**	68	83
4	**CPA-5**	79	85
5	**CPA-6**	31	10[d]

[a] Standard conditions: 0.1 mmol **1a**, 0.25 mmol **2a**, 0.25 mmol **3a**, 0.01 mmol **CPA**, 0.2 mL toluene, 40 °C, 24 h. [b] Determined by ^{1}H NMR analysis of the crude mixture using bibenzyl as internal standard. [c] Determined by CSP HPLC. [d] Major enantiomer: (2*S*,3*S*)-**4a**.

As shown in Table S3, increasing the steric hindrance of the 3,3’ aromatic groups as in **CPA-3** ((*R*)-TCYP) improved the enantioselectivity (compare entries 2 and 1). Concurrently, increasing the dihedral angle of the binaphthyl core by moving from **CPA-1** to the corresponding **CPA-4** ((*R*)-H_8-TRIP) gave a notable improvement too (compare entries 1 and 3). Using the (*R*)-SPINOL derivative **CPA-6** ((*R*)-STRIP) was not successful (entry 5). The two positive features (hindered 2,4,6-$Cy_3C_6H_2$ 3,3’-substituents, and (*R*)-H_8-BINOL scaffold) were combined in catalyst **CPA-5** ((*R*)-H_8-TCYP), which rendered the best results obtained so far (entry 4).

***Table S4**: Different temperatures and substrates.*

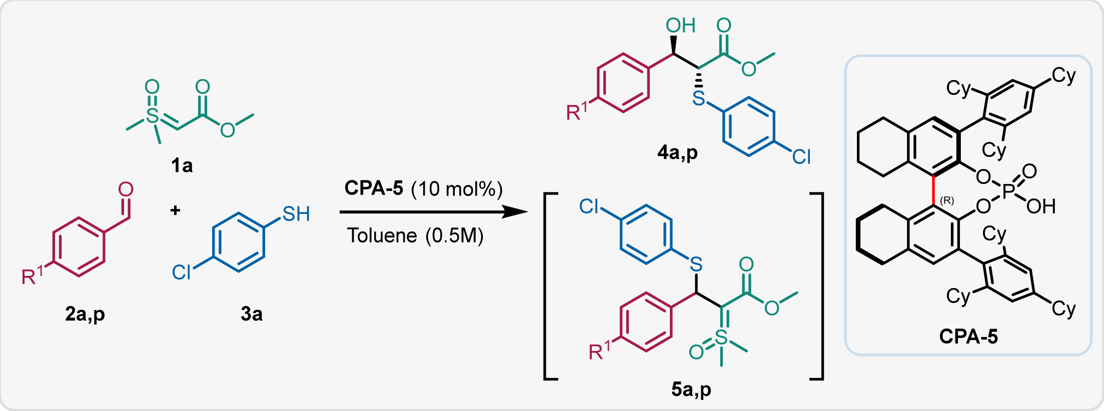

Entry[a]	**2**	*R*[1]	*T (°C)*	*Time (h)*	**4**	*Yield*[b] *(%)*	*ee*[c] *(%)*
1	**2a**	H	40	24	**4a**	79	85
2	**2a**	H	-10	72	**4a**	80	88
3	**2p**	NO_2	-10	72	**4p**	<5[d]	-
4	**2p**	NO_2	60	16	**4p**	63[e]	88

[a] Standard conditions: 0.1 mmol **1a**, 0.25 mmol **2**, 0.25 mmol **3**, 0.01 mmol **CPA-5**, 0.2 mL toluene. [b] Determined by ^{1}H-NMR using dibromomethane as an internal standard. [c] Determined by CSP HPLC. [d] Intermediate **5p** as main product. [e] Isolated yield.

A decrease in temperature enhanced the enantiomeric excess of product **4a**, and using a longer reaction time the yield was the same as that obtained at 40 °C (entries 1,2). However, when these conditions were applied to electron-poor aldehydes and thiophenols, the reactions stalled at intermediates **5** (see section α,α-Disubstituted ylides **5**). For example, the reaction with 4-nitrobenzaldehyde **2p** led just to intermediate **5p** after 72 h at -10 °C, with no evidence of formation of product **4p** (entry 3). On the other hand, evolution from intermediate **5p** to product **4p** occurred at higher temperatures. Performing the reaction at 60 °C afforded product **4p** in acceptable yield after only 16 h, maintaining a satisfactory ee value (entry 4). Entries 2 and 4 outline two sets of optimized conditions for the catalytic reaction, according to the inherent electronic substituents on the aromatic rings of the substrates (general procedures **B** and **D**, see section: General procedures).

Reaction side-products, coproducts, and intermediates

In this section, compounds **5-7** and **11,12** detected during the optimization of the reaction are reported and discussed (Scheme S3). In some cases, the identification of these compounds has significantly informed the reaction optimization and the mechanistic studies.

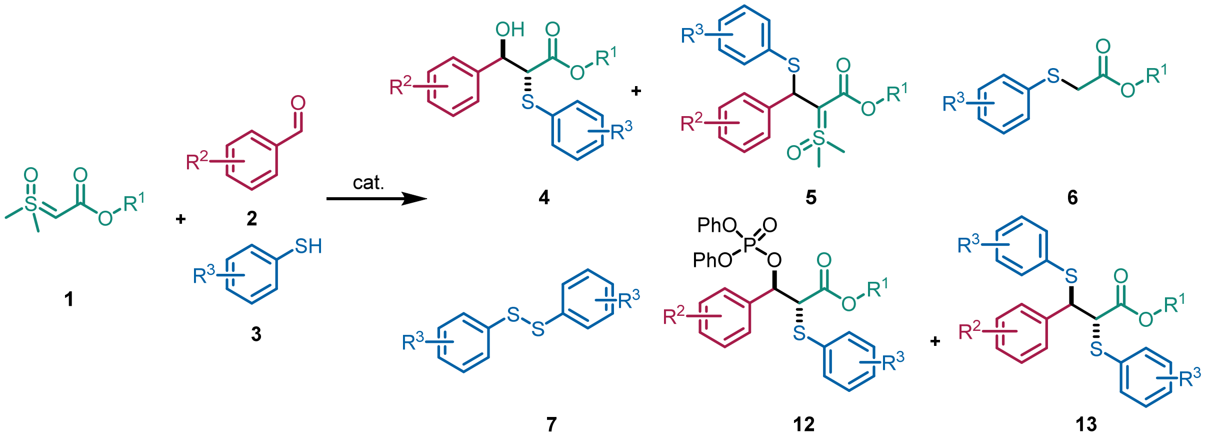

***Scheme S3**: Products identified during the reaction optimization*

α,α-Disubstituted ylides **5**

Compounds **5** were initially detected and identified during the study of the reaction scope, specifically with aldehyde substrates bearing electron withdrawing substituents (see Table S4). In these cases, formation of compounds **5** occurs readily at sub-zero temperatures, but evolution of **5** to the products **4** requires higher temperatures. However, the formation of these compounds **5** was found to be general. All substrates employed in the 3CR led to adducts **5** as main products, at short reaction times and/or low temperatures.

The isolation of these species presented several difficulties, mainly related to the poor stability of compounds **5** on silica. Despite these challenges, **5a**, formed in the model reaction between **1a**, **2a** and **3a**, was isolated. However, the presence of minor impurities on the spectra complicated its full characterization. To address this, we synthesized compound **5p** derived from the electron-poor 4-nitrobenzaldehyde **2p**, which thanks to its reduced reactivity turned out to be easier to purify. Accordingly, compound **5p** was fully characterized by 2D NMRs. The ^{1}H NMR spectra of **5a** and **5p** present a characteristic signal at ca. 5.6 ppm (br s, 1H), which was considered diagnostic of the presence of **5** in the reaction mixtures of other substrates.

5a - Methyl 3-((4-chlorophenyl)thio)-2-(dimethyl(oxo)-λ^{6}-sulfaneylidene)-3-phenylpropanoate

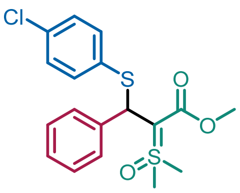

^{1}H NMR (600 MHz, CDCl$_3$) δ 7.55 (d, *J* = 7.6 Hz, 2H), 7.33 – 7.27 (m, 3H), 7.23 – 7.17 (m, 4H), 5.66 (br s, 1H), 3.63 (s, 3H), 3.34 (s, 3H), 3.03 (s,3H).

^{13}C NMR (151 MHz, CDCl$_3$) δ 142.1, 135.7, 135.6, 133.1, 132.6, 129.1, 128.8, 128.0, 127.9, 126.6, 50.2, 48.8, 44.4, 43.0.

ESI MS [M+Na]$^{+}$: 405 m/z

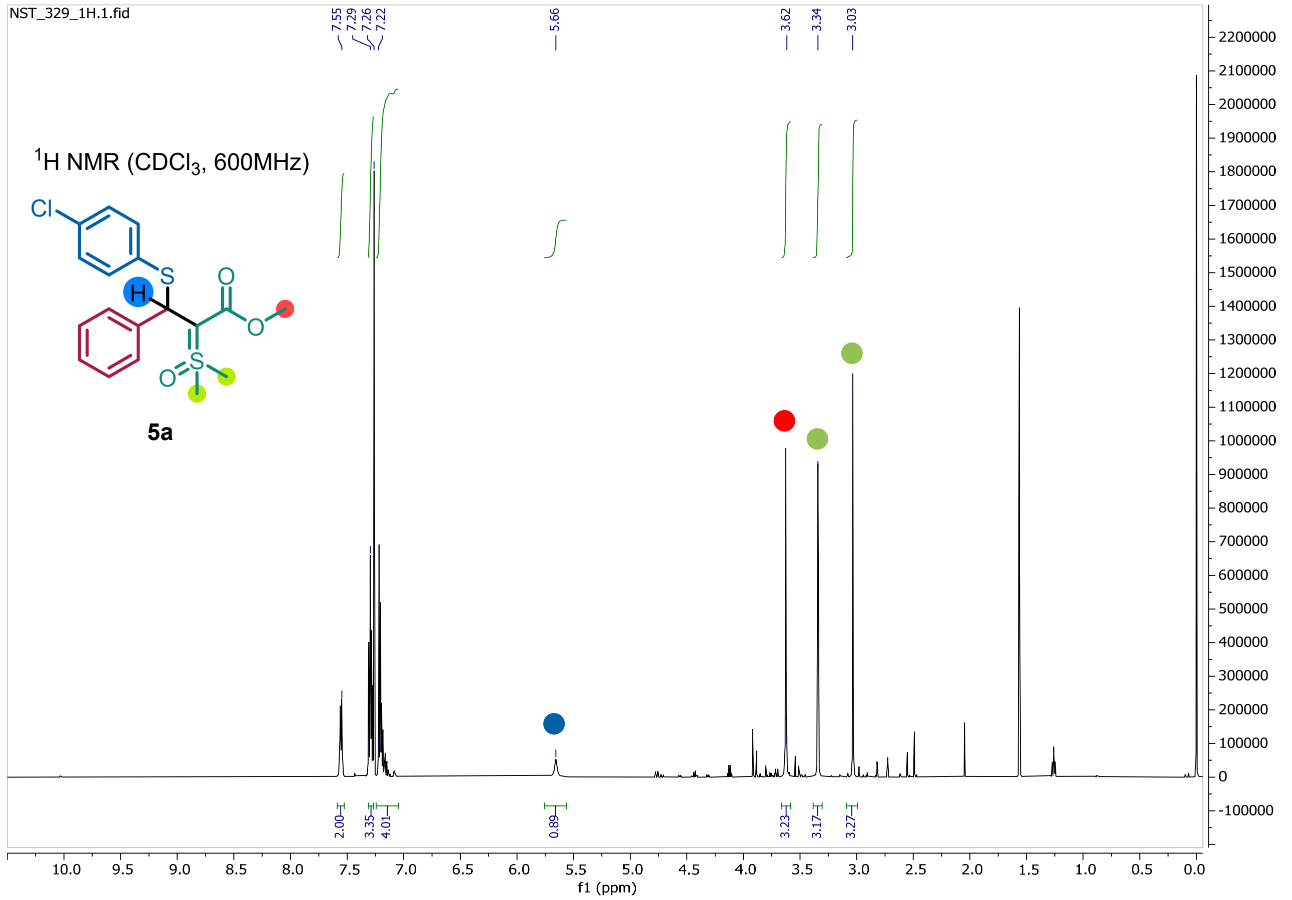
NST_329_1H.1.fid
^{1}H NMR (CDCl$_3$, 600MHz)
7.55
7.29
7.26
7.22
5.66
3.62
3.34
3.03
2.00
3.35
4.01
0.89
3.23
3.17
3.27
5a
f1 (ppm)

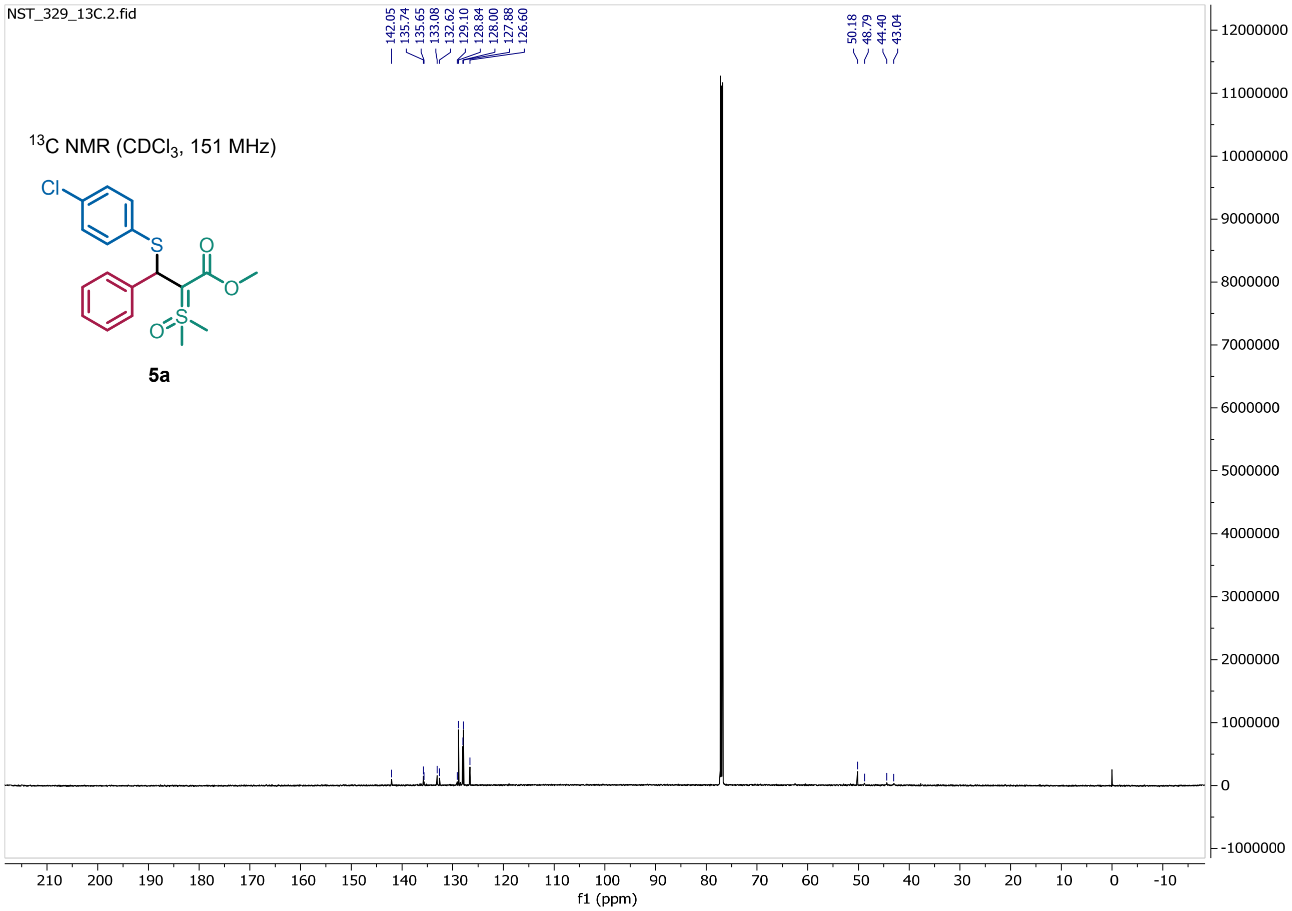
NST_329_13C.2.fid
^{13}C NMR (CDCl$_3$, 151 MHz)
142.05
135.74
135.65
133.08
132.62
129.10
128.84
128.00
127.88
126.60
50.18
48.79
44.40
43.04
5a
f1 (ppm)

5p - Methyl 3-((4-chlorophenyl)thio)-2-(dimethyl(oxo)-l6-sulfaneylidene)-3-(4-nitrophenyl) propanoate

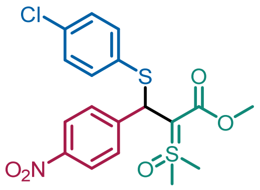

^{1}H NMR (600 MHz, $CDCl_3$) δ 8.07 – 8.04 (m, 2H), 7.64 (d, *J* = 8.3 Hz, 2H), 7.23 – 7.18 (m, 2H), 7.17 – 7.12 (m, 2H), 5.57 (s, 1H), 3.53 (s, 3H), 3.32 (s, 3H), 3.05 (s, 3H).

^{13}C NMR (151 MHz, $CDCl_3$) δ 166.26, 149.81, 146.43, 134.64, 133.15, 129.05, 128.87, 123.16, 61.88, 50.20, 48.33, 44.48, 43.01.

ESI MS $[M+Na]^+$: 428 m/z.

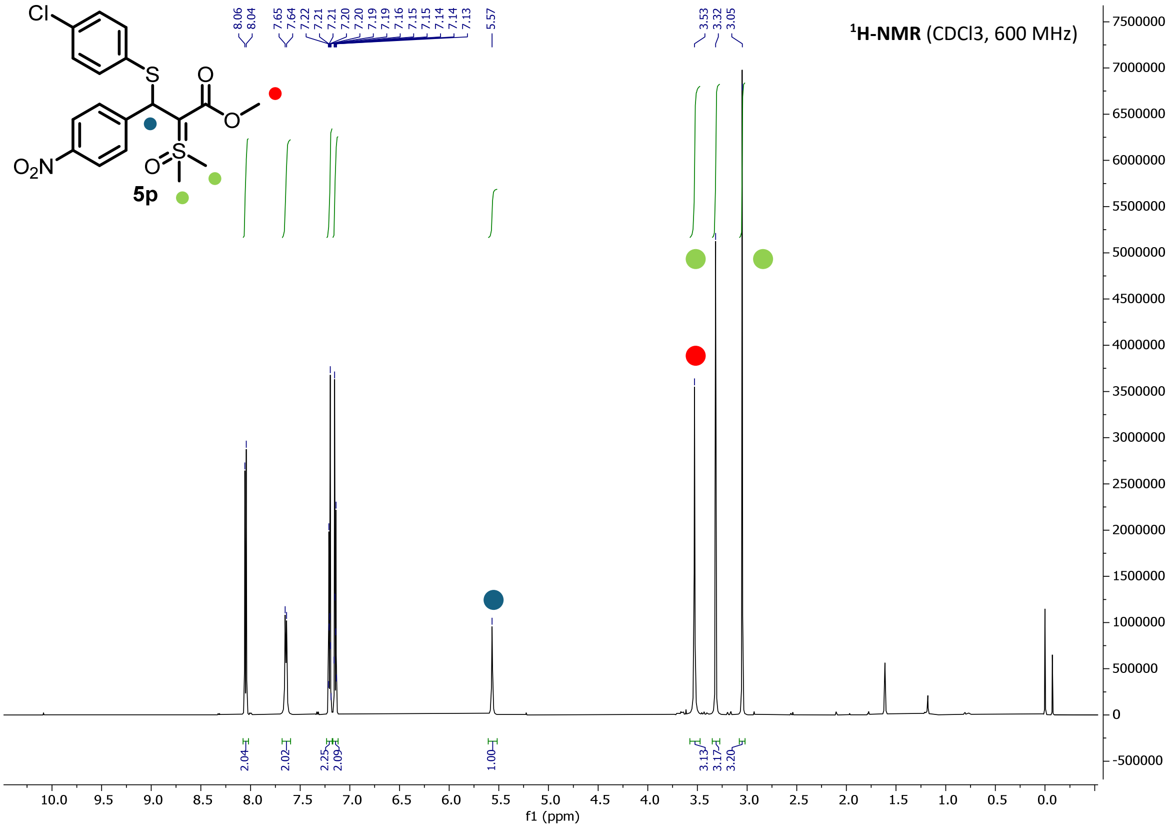

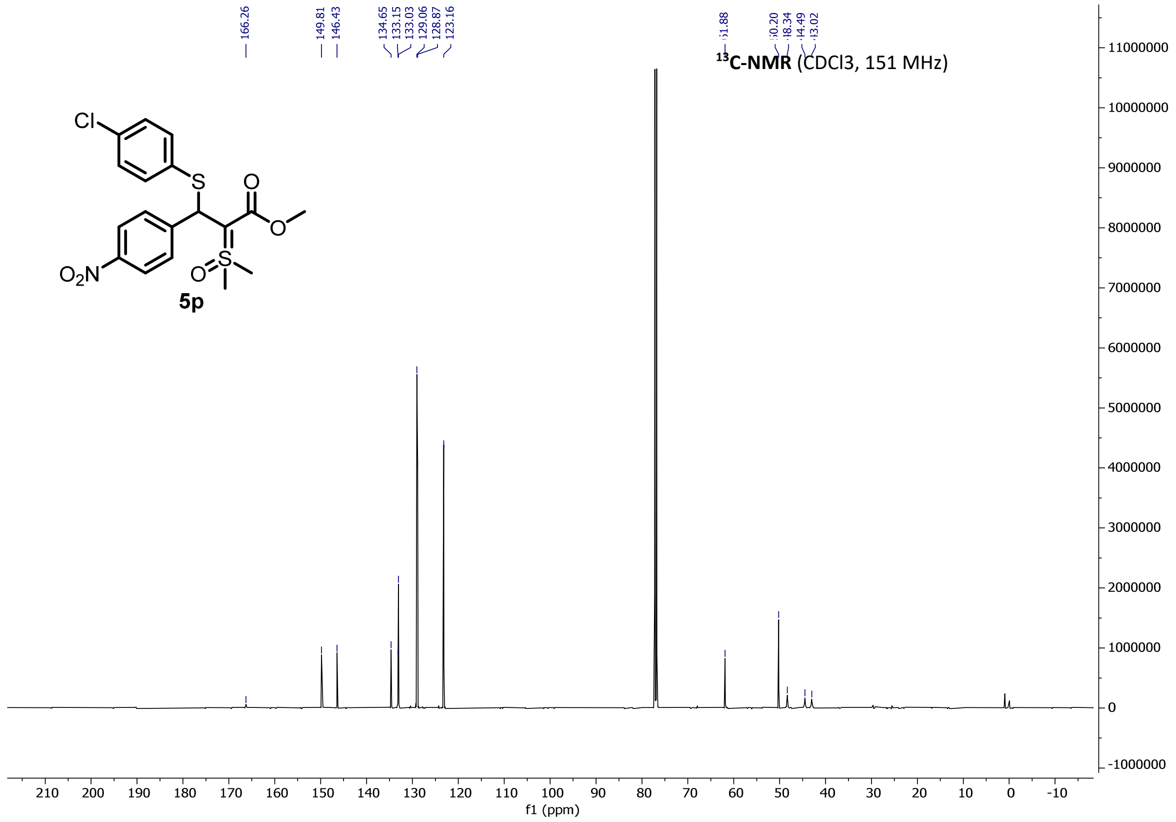

166.26
149.81
146.43
134.65
133.15
133.03
129.06
128.87
123.16
13C-NMR (CDCl3, 151 MHz)
5p
f1 (ppm)

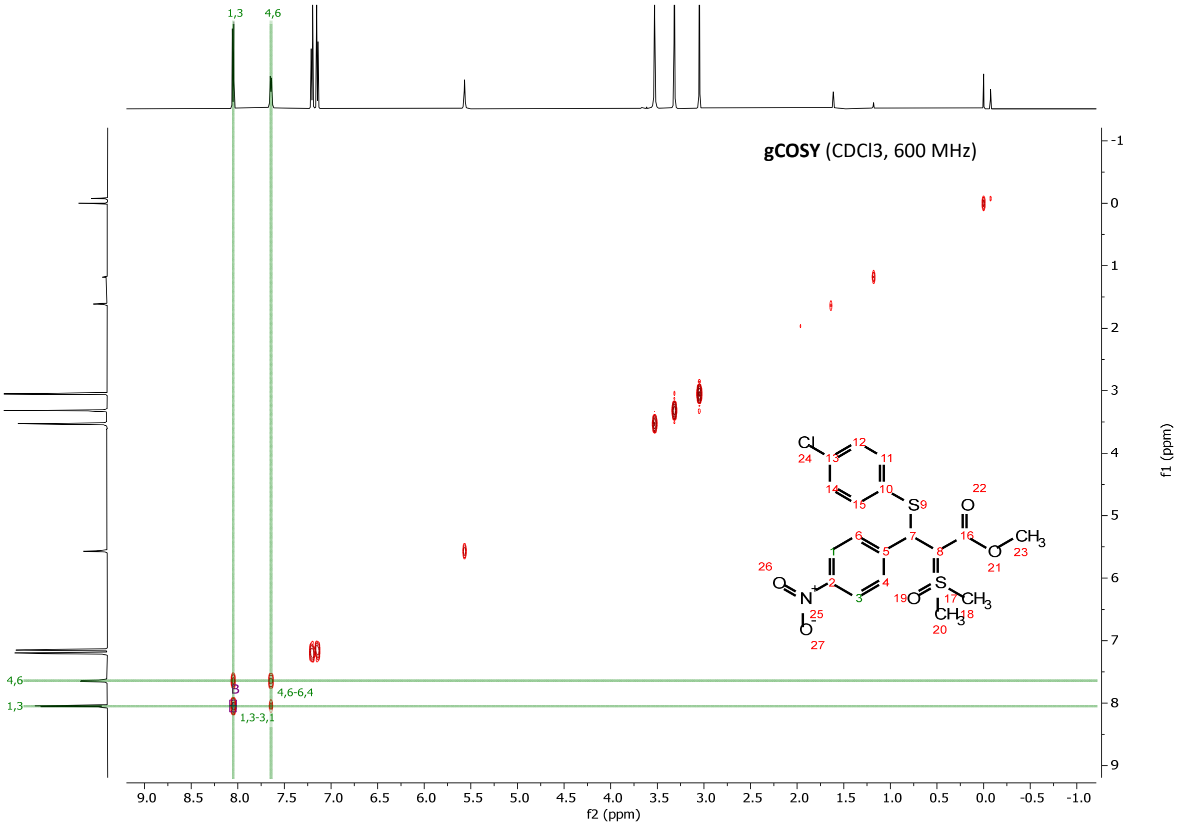

gCOSY (CDCl3, 600 MHz)
1,3
4,6
4,6-6,4
1,3-3,1
f2 (ppm)
f1 (ppm)

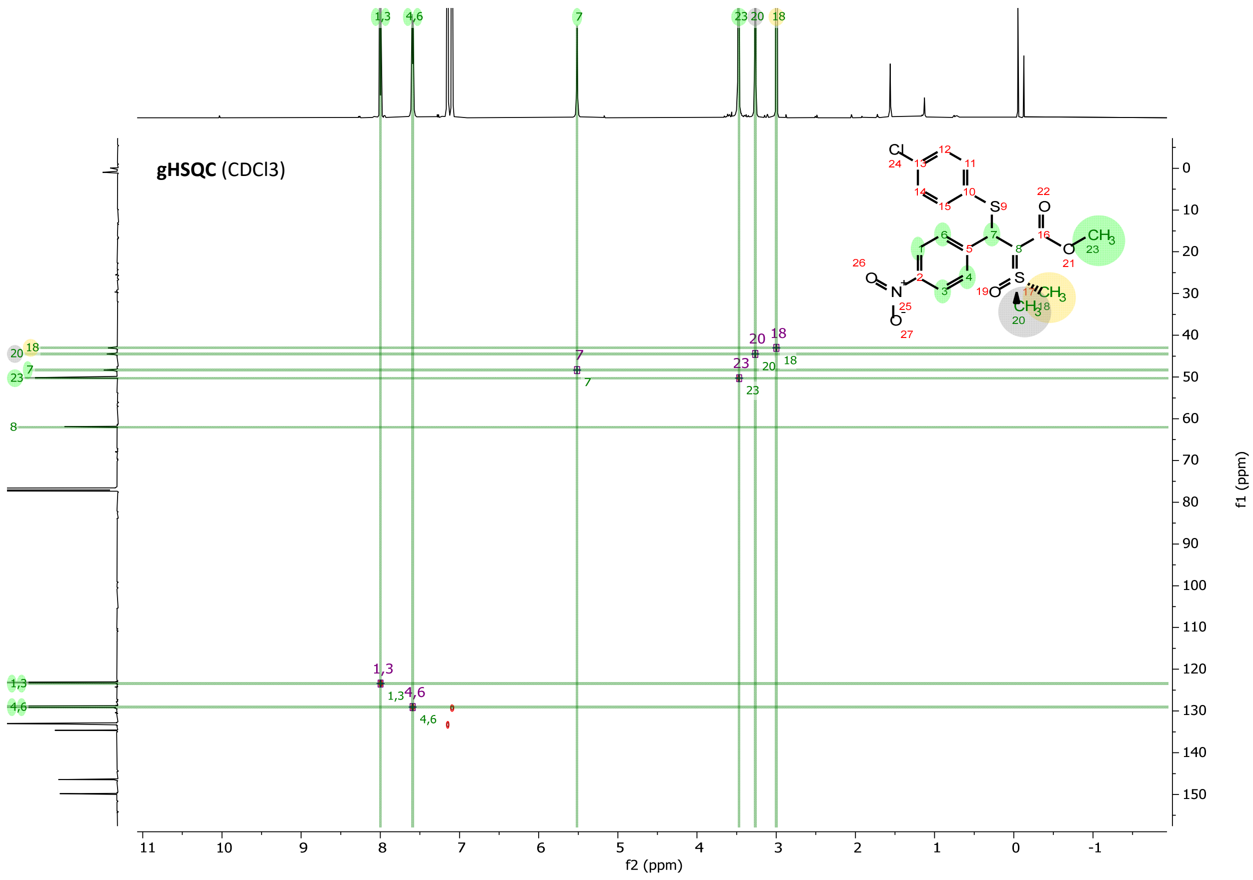
gHSQC (CDCl3)
f2 (ppm)
f1 (ppm)
1,3
4,6
7
23
20
18
8

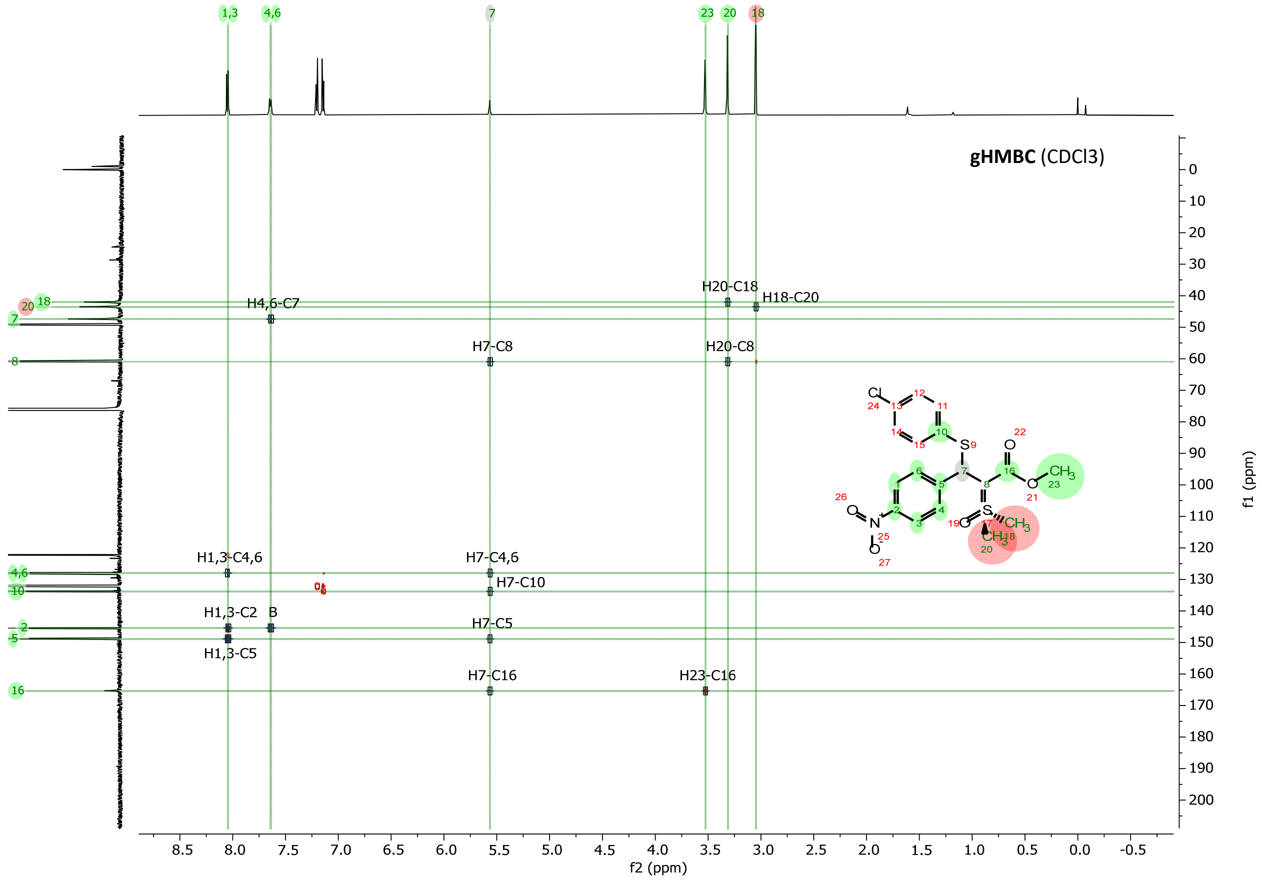
gHMBC (CDCl3)
f2 (ppm)
f1 (ppm)
H4,6-C7
H20-C18
H18-C20
H7-C8
H20-C8
H1,3-C4,6
H7-C4,6
H7-C10
H1,3-C2
B
H7-C5
H1,3-C5
H7-C16
H23-C16

S-H insertion products **6**

This type of reactivity was reported by Dias and Burtoloso in 2016[1]. In our case, the S-H insertion product **6a**[2] is the main reaction product when the reaction is performed in the absence of catalyst, at 40 °C in toluene (79% yield). However, this pathway is slower than the phosphoric acid-catalyzed reaction that leads to the 3C product **4a**, at least in toluene. Thus, in general this side pathway leading to **6** does not compete with the catalytic three-component catalytic reaction in toluene. Only in relatively polar solvents, like acetonitrile, the formation of **6** becomes more relevant. Indeed, Dias and Burtoloso reported their catalyst-free S-H insertion in acetonitrile. Other exceptions were observed in the reactions with the most acidic thiophenols used in this study (*e.g.* 4-(trifluoromethyl)benzenethiol **3c**). The ^{1}H NMR spectra of the crude mixtures of these catalytic reactions presented signals compatible with the corresponding S-H insertion products **6**. Thus, it can be surmised that more acidic thiophenols react faster than less acidic ones in the catalyst-free S-H insertion reaction, since they can protonate the sulfoxonium ylide, and thus compete to some extent with the catalytic three-component reaction. In other cases, the competition is not relevant.

Disulfides **7**

Disulfides **7** were detected in reaction mixtures from the outset of the project and were initially considered as side-products. Since their formation can severely affect the yield of **4,** removing from the reaction mixture up to two equivalents of thiophenols **3**, several attempts were put in place to avoid their formation, which was initially ascribed to the oxidation of **3** by adventitious oxygen and/or light. Thus, catalytic reactions were set up under highly controlled conditions (deoxygenated mixture, light protection, inert atmosphere, radical scavengers, etc.) but did not lead to useful results. It was empirically found that by using the thiol in excess (2.5 equivalents) the yield could be improved to reasonable levels. In parallel, to gain some insights on this oxidation, experiments with 4-fluorobenzaldehyde (**2i**) and 4-fluorothiophenol (**3d**), facilitating reaction monitoring and allowing to follow the formation of disulfide **7d**[3] by ^{19}F NMR, were performed (Table S5). An experiment where thiol **3d** was left standing in the reaction solvent (untreated, no inert atmosphere) for 96 h, showed a marginal conversion of the thiol **3d** to the disulfide **7d** (entry 1). This result showed that the oxidation of **3d** to **7d** is not caused exclusively by oxygen/light, accounting for the inefficacy of controlled conditions (inert atmosphere, etc.) for yield improvement. As expected, neither the 4-fluorobenzaldehyde **2i** nor the ylide **1a** promoted the formation of **7d** (entries 2,3), which instead proceeded to completion within the timeframe of the reaction (3 h) when all reaction components were present (entry 4).

Table S5**: Control experiments for the detection of disulfide **7d

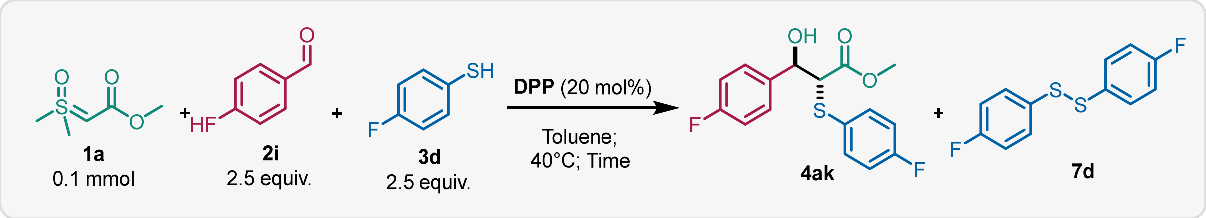

Entry	*Reaction components/additives*	*Time (h)*	*Yield[a] **7d** (%)*
1	**3d**	96	11%
2	**3d** + **1a**	24	<5%
3	**3d** + **2i** + **1a**	24	<5%
4	**3d** + **2i** + **1a** + **DPP**	3	>95%

[a] Determined by ^{19}F NMR using 1,3-bis(trifluoromethyl)benzene as internal standard.

[1] R. M. P. Dias, A. C. B. Burtoloso, *Org. Lett.*, **2016**, *18*, 3034-3037.
[2] E. Labarrios, A. Jerezano, F. Jiménez, M. d. C. Cruz, F. Delgado, L. G. Zepeda, J. Tamariz, *J. Heterocyclic Chem.*, **2014**, *51*, 954–971.
[3] S. Sun, J. Li, L. Pan, H. Liu, Y. Guo, Z. Gao, X. Bi, *Org. Biomol. Chem.* **2022**, *20*, 8885–8892.

Considering that, under acidic conditions, DMSO – the hypothesized co-product if the catalytic reaction - can oxidize thiols delivering disulfides and dimethylsulfide (DMS)[4], experiments were set up with DMSO alone, and with DMSO (1 equiv.) in the presence of the acidic **DPP** catalyst. It was first observed that DMSO alone oxidizes thiol **3d** slowly, leading to only 34% of **7d** after 24 h. On the contrary, the presence of **DPP** catalyzes the oxidation of **3d** by DMSO, leading to full oxidation after 24 h (Table S6, entry 4). However, by following the oxidation over time, the kinetics of this oxidation was found to be slower than the catalytic reaction itself. The catalytic reaction goes to completion in less than 3 h. Within this timeframe, only 27% of the thiophenol **3d** was oxidized by DMSO in the presence of **DPP** (entry 3). Moreover, part of the disulfide **7d** was already present from the reaction outset, in the batch of **3d** used for these experiments (entry 1), thus making the oxidation of **3d** by DMSO even less relevant. These results are in line with the proposed mechanism (see section Mechanistic proposal), where disulfides **7** are co-products, rather than side-products, of the reactions - their formation is inherently linked to the mechanism of the reaction.

Table S6. *Formation of disulfide* ***7d*** *over time.*

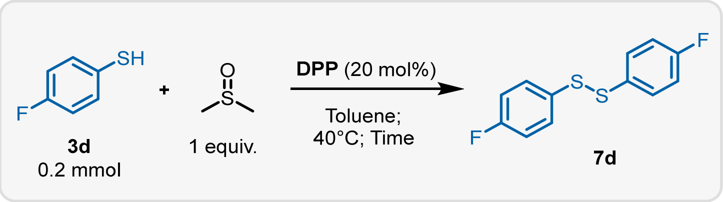

Entry	*Time (h)*	*Thiophenol[a]* ***3d*** *(%)*	*Disulfide[a]* ***7d*** *(%)*
1[b]	0	91	9
2	2	82	18
3	3	73	27
4	24	<5%	>95%

[a] Determined by ^{19}F NMR using 1,3-bis(trifluoromethyl)benzene as internal standard. [b] ^{19}F NMR analysis of thiol **3d** before the reaction.

Catalyst-trapped adducts **12**

When carrying out the reactions with **DPP** catalyst – to obtain the reference racemic samples for CSP HPLC analysis - we often noticed the formation of catalyst-trapped side-products **12**. Depending on the nature of the thiophenol or of the aldehyde, these compounds formed in up to 20% yield, in line with the catalyst loading. Ultimately, we could isolate and characterize a representative compound **12a** in the reaction with 4-nitrobenzaldehyde **2p** and 4-fluorothiophenol **3d** (Scheme S4). In contrast with other aldehydes and thiols, this substrate combination rendered an adduct (**12a**) sufficiently stable to allow isolation by chromatography on silica gel, and ultimately its characterization and structural assignment by 2D NMR and ESI-MS experiments. Its ^{1}H NMR spectrum presents a characteristic signal at 5.85 ppm (dd, J = 10.3, 7.2 Hz, 1H), which was considered diagnostic of the presence of catalyst-trapped adducts **12** in the reactions with other substrates.

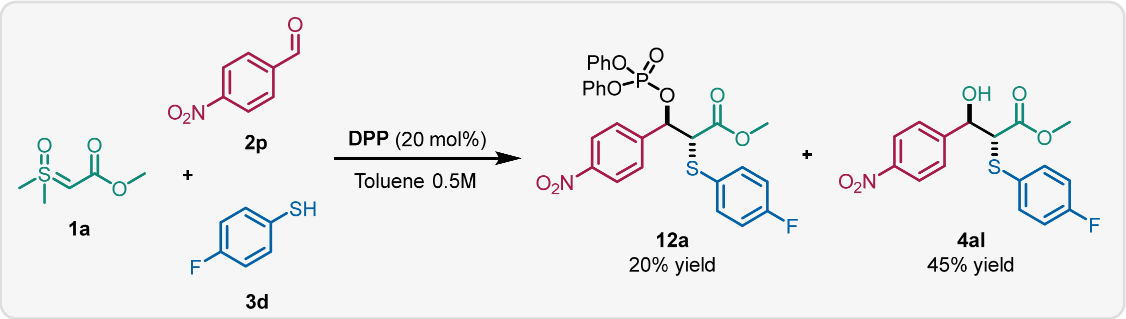

***Scheme S4. DPP** catalyst-trapped adduct **12a** isolated and characterized.*

[4] P. Natarajan, H. Sharma, M. Kaur, P. Sharma, *Tetrahedron Lett.* **2015**, *56*, 5578–5582.

12a: Methyl (2*R**,3*R**)-3-((diphenoxyphosphoryl)oxy)-2-((4-fluorophenyl)thio)-3-(4-nitrophenyl)propanoate

^{1}H NMR (400 MHz, $CDCl_3$) δ 8.04 (d, *J* = 8.7 Hz, 2H), 7.42 (d, *J* = 8.7 Hz, 2H), 7.31 (d, *J* = 7.9 Hz, 2H), 7.21 – 7.02 (m, 8H), 6.91 – 6.81 (m, 4H), 5.85 (dd, *J* = 10.3, 7.2 Hz, 1H), 3.92 (d, *J* = 10.3 Hz, 1H), 3.57 (s, 3H).

^{13}C NMR (151 MHz, $CDCl_3$) δ 169.0, 163.2 (d, *J* = 250.8 Hz), 150.2 (d, *J* = 7.2 Hz), 149.9 (d, *J* = 7.1 Hz), 148.3, 142.4, 136.2 (d, *J* = 8.7 Hz), 129.8, 129.6, 129.1, 126.6 (d, *J* = 3.6 Hz), 125.5, 125.4, 123.5, 120.0 (d, *J* = 5.0 Hz), 119.8 (d, *J* = 4.9 Hz), 116.4 (d, *J* = 22.0 Hz), 79.8 (d, *J* = 5.6 Hz), 57.1 (d, *J* = 10.0 Hz), 52.8.

^{19}F NMR (376 MHz, $CDCl_3$) δ -110.78 – -110.93 (m, 1F).

^{31}P NMR (243 MHz, $CDCl_3$) δ -13.70 (d, *J* = 7.2 Hz, 1P).

ESI MS $[M+Na]^+$: 606 m/z

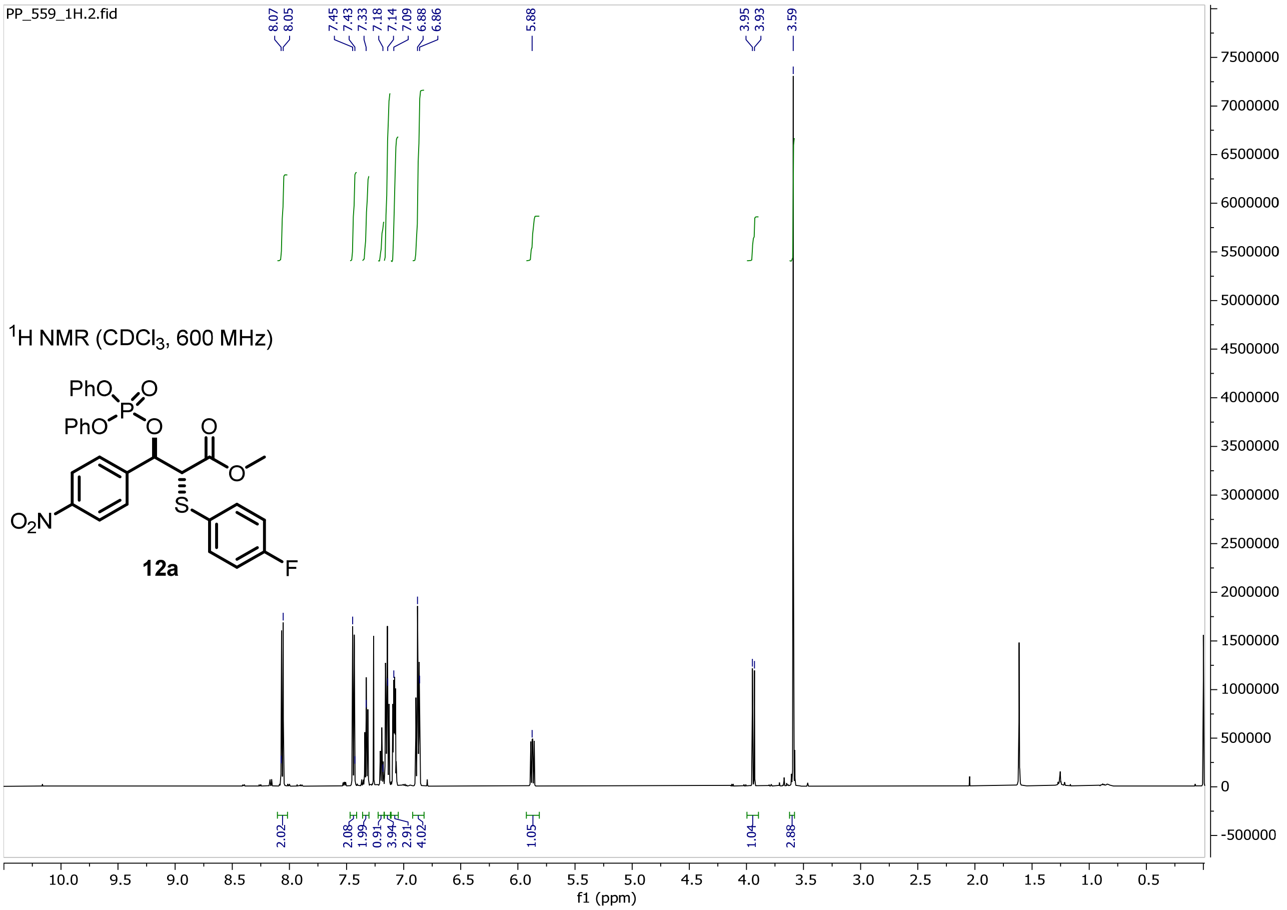

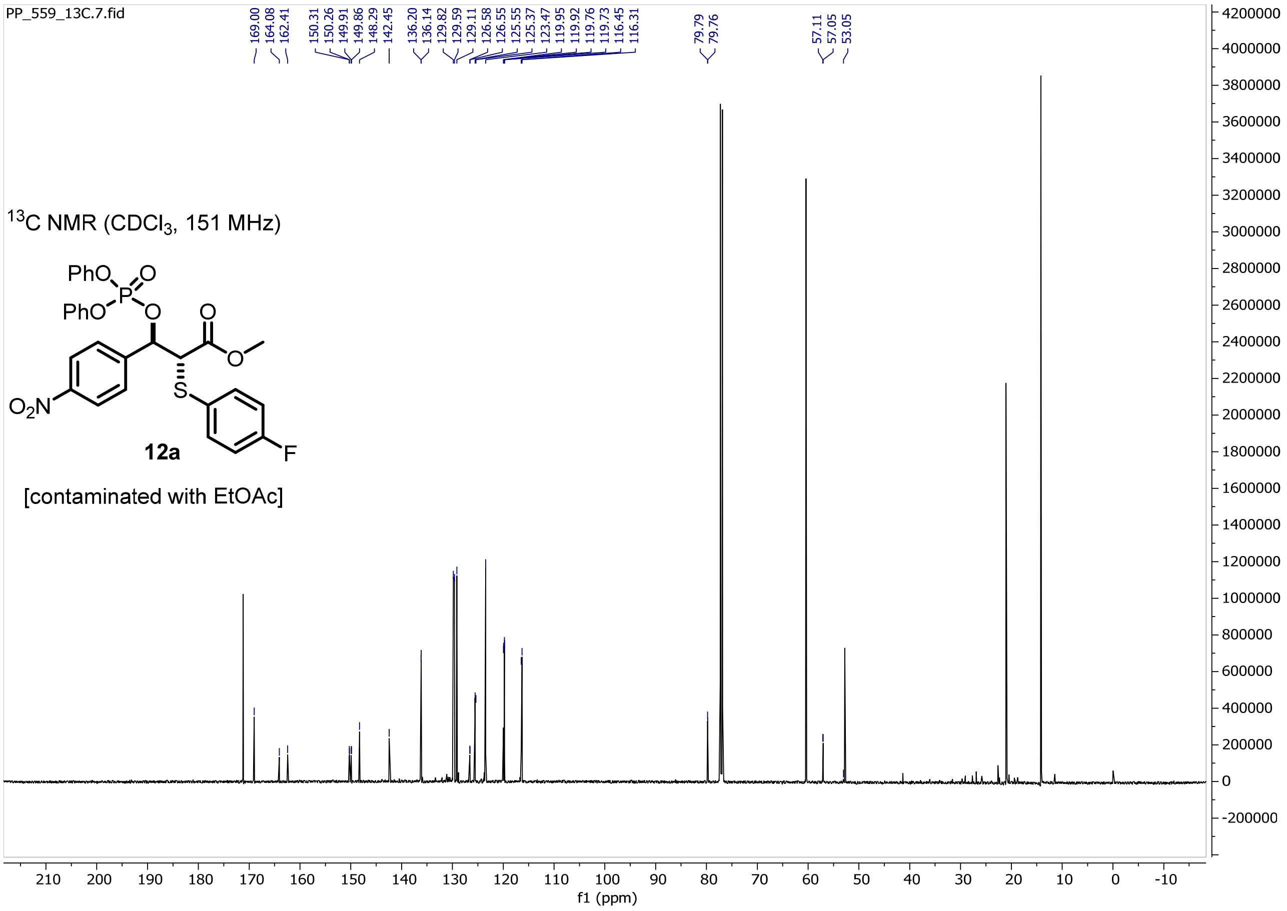
PP_559_13C.7.fid
169.00
164.08
162.41
150.31
150.26
149.91
149.86
148.29
142.45
136.20
136.14
129.82
129.59
129.11
126.58
126.55
125.55
125.37
123.47
119.95
119.92
119.76
119.73
116.45
116.31
79.79
79.76
57.11
57.05
53.05
13C NMR (CDCl3, 151 MHz)
PhO
O
P
PhO
O
O
O
O2N
S
F
12a
[contaminated with EtOAc]
f1 (ppm)

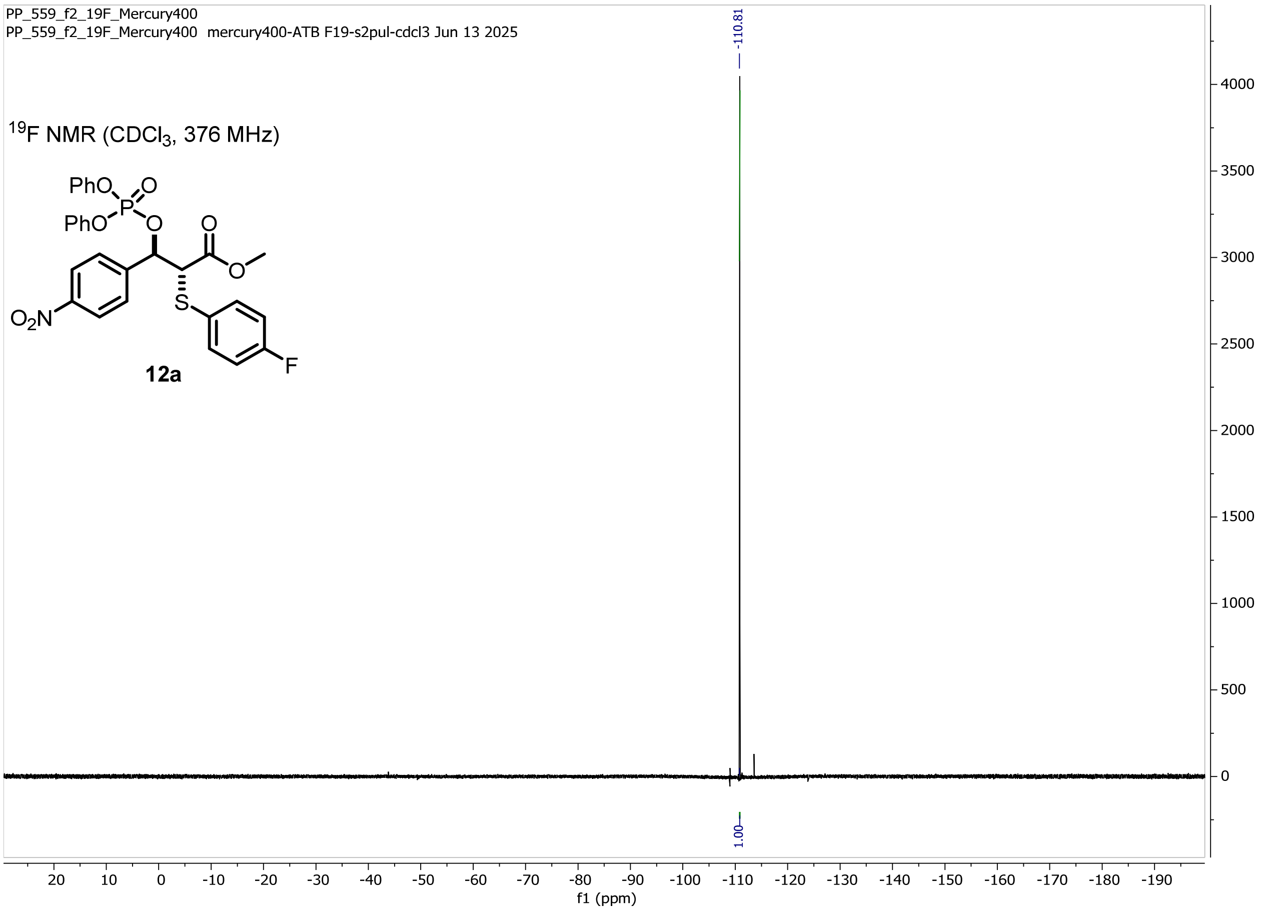
PP_559_f2_19F_Mercury400
PP_559_f2_19F_Mercury400 mercury400-ATB F19-s2pul-cdcl3 Jun 13 2025
-110.81
19F NMR (CDCl3, 376 MHz)
PhO
O
P
PhO
O
O
O
O2N
S
F
12a
1.00
f1 (ppm)

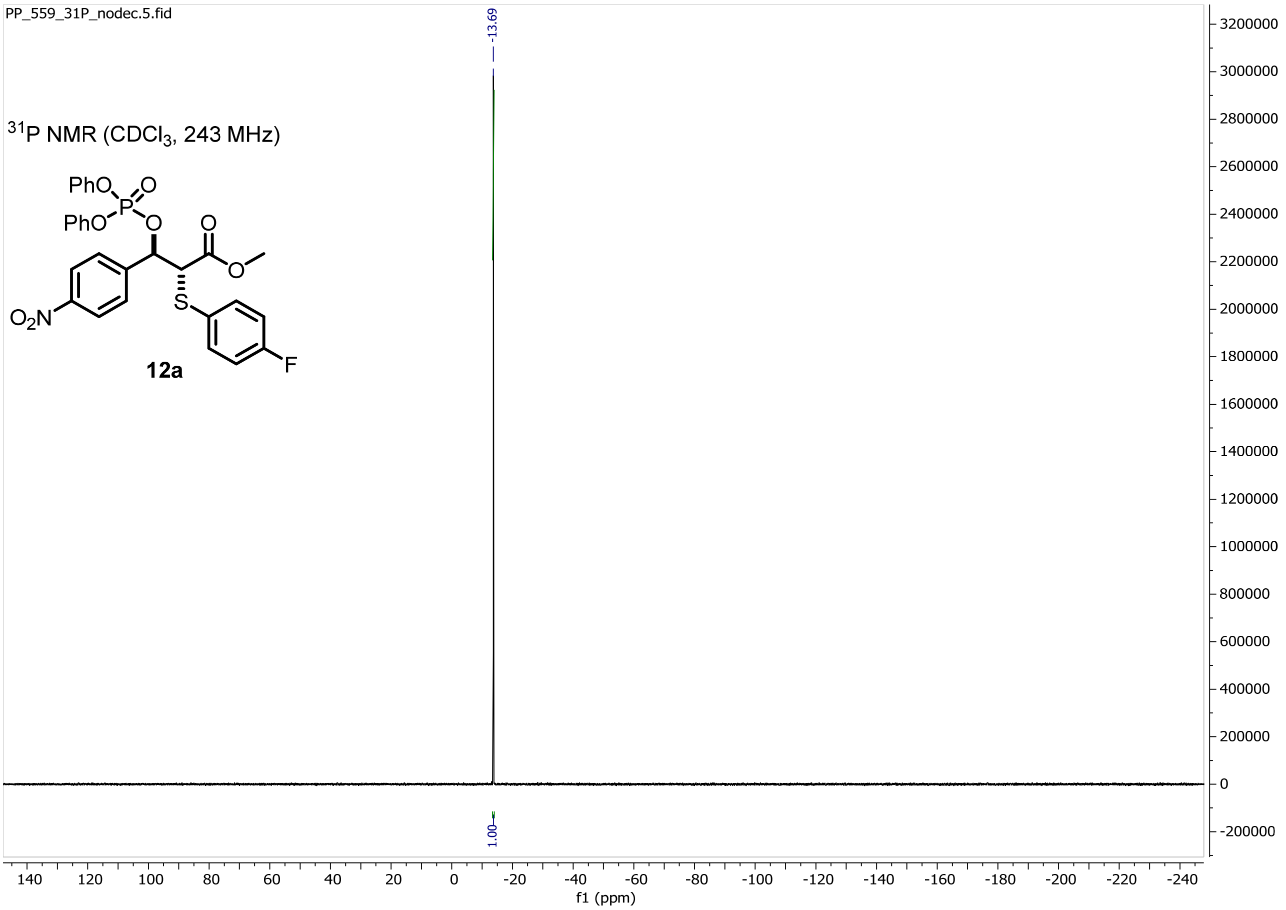

We initially hypothesized **12** could originate from products **4**, in a consecutive reaction. However, a control experiment performed by treating product **4a** in the presence of **DPP** revealed no formation of the corresponding **12b** (Scheme S5). Furthermore, analysis of the reaction progress showed that **12b** forms concomitantly with **4a**.

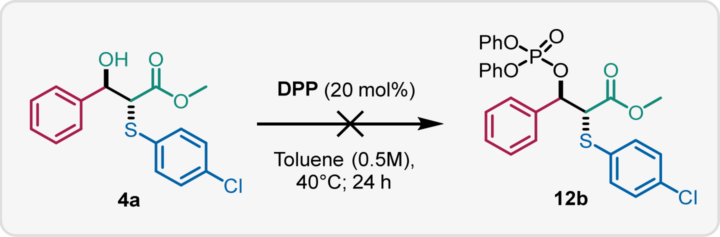

Scheme S5. *Control experiment showing that side-products* ***12*** *do not form from products* ***4****.*

A second hypothesis considered catalyst-trapped adducts **12** as off-cycle or in-cycle intermediates of the catalytic processes. However, the isolated adduct **12a** did not render the corresponding product **4al** under the reaction conditions (Scheme S6).

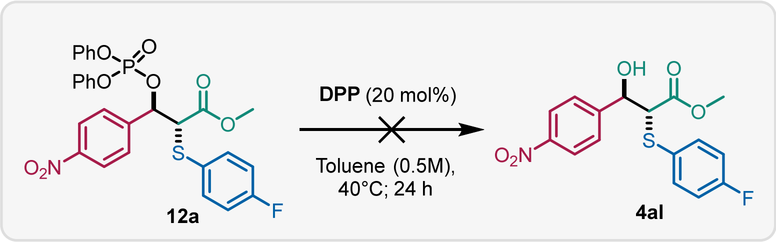

Scheme S6*. Control experiment showing that* ***12*** *are not reaction intermediates.*

Overall, these experiments indicate that **12** are dead-end compounds resulting in catalyst trapping and deactivation. Importantly, these side-products **12** do not form with the optimal chiral phosphoric acid catalyst **CPA-5**, possibly for

the steric hindrance of this chiral catalyst, thus making catalyst deactivation through this pathway not relevant for the catalytic asymmetric process.

2,3-Bis(arylthio) carbonyl derivatives **13**

During the study of the reaction, we observed under certain conditions the formation of 2,3-bis(arylthio) carbonyl derivatives **13**. A distinct trend was observed in the formation of these side-products **13**, wherein an increase in solvent polarity correlated with a higher yield of **13**. Specifically, in toluene, the formation of **13** consistently remained below 10% with all substrates tested and under a variety of conditions (catalysts, temperature, etc.). In contrast, in acetonitrile, the yield of byproduct **13** increased to approximately 30%, as shown in Scheme S7 for a representative example. In methanol, a polar protic solvent, it reached approximately 70%. A control experiment verified that product **13** does not form from the three component adduct **4** under the reaction conditions. Product **13a** derived from fluorine-containing substrates **2i** and **3d**, employed to facilitate reaction monitoring by ^{19}F NMR, was isolated and characterized. Products **13** formed always with full diastereoselectivities (dr > 20:1, likely in favor of the *anti*-isomers). This trend of yield **13** *vs* solvent polarity is in line with the mechanistic proposal.

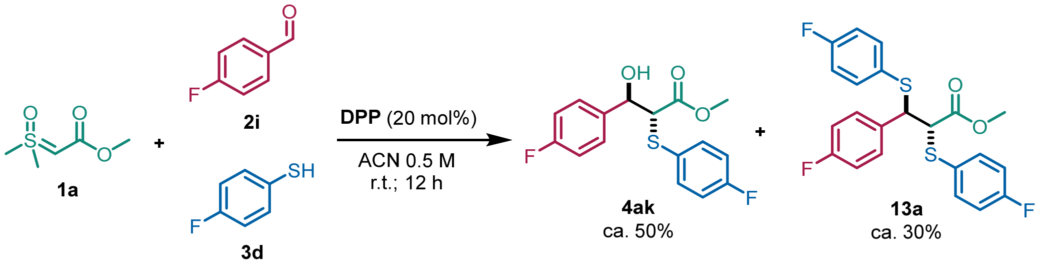

***Scheme S7**. A representative example of the formation of side-product **13**.*

13a - Methyl (2*S**,3*R**)-3-(4-fluorophenyl)-2,3-bis((4-fluorophenyl)thio)propanoate

^{1}H NMR (600 MHz, $CDCl_3$) δ 7.18 – 7.14 (m, 2H), 7.13 – 7.08 (m, 2H), 6.95 – 6.87 (m, 8H), 4.28 (d, *J* = 12.0 Hz, 1H), 3.95 (d, *J* = 12.1 Hz, 1H), 3.76 (s, 3H).

^{13}C NMR (151 MHz, $CDCl_3$) δ 170.8, 163.4 (d, *J* = 249.2 Hz), 163.3 (d, *J* = 250.4 Hz), 162.9 (d, *J* = 246.5 Hz), 137.7 (d, *J* = 8.3 Hz), 136.8 (d, *J* = 8.4 Hz), 133.4 (d, *J* = 3.2 Hz), 130.2 (d, *J* = 8.1 Hz), 126.9 (d, *J* = 3.4 Hz), 126.5 (d, *J* = 3.5 Hz), 116.1 (d, *J* = 21.8 Hz), 116.0 (d, *J* = 21.8 Hz), 115.1 (d, *J* = 21.4 Hz), 55.5, 53.9, 52.5.

^{19}F NMR (376 MHz, $CDCl_3$) δ -111.48 – -111.61 (m, 1F), -111.66 – -111.80 (m, 1F), -113.83 – -113.95 (m, 1F).

ESI MS $[M+Na]^+$: 457 m/z

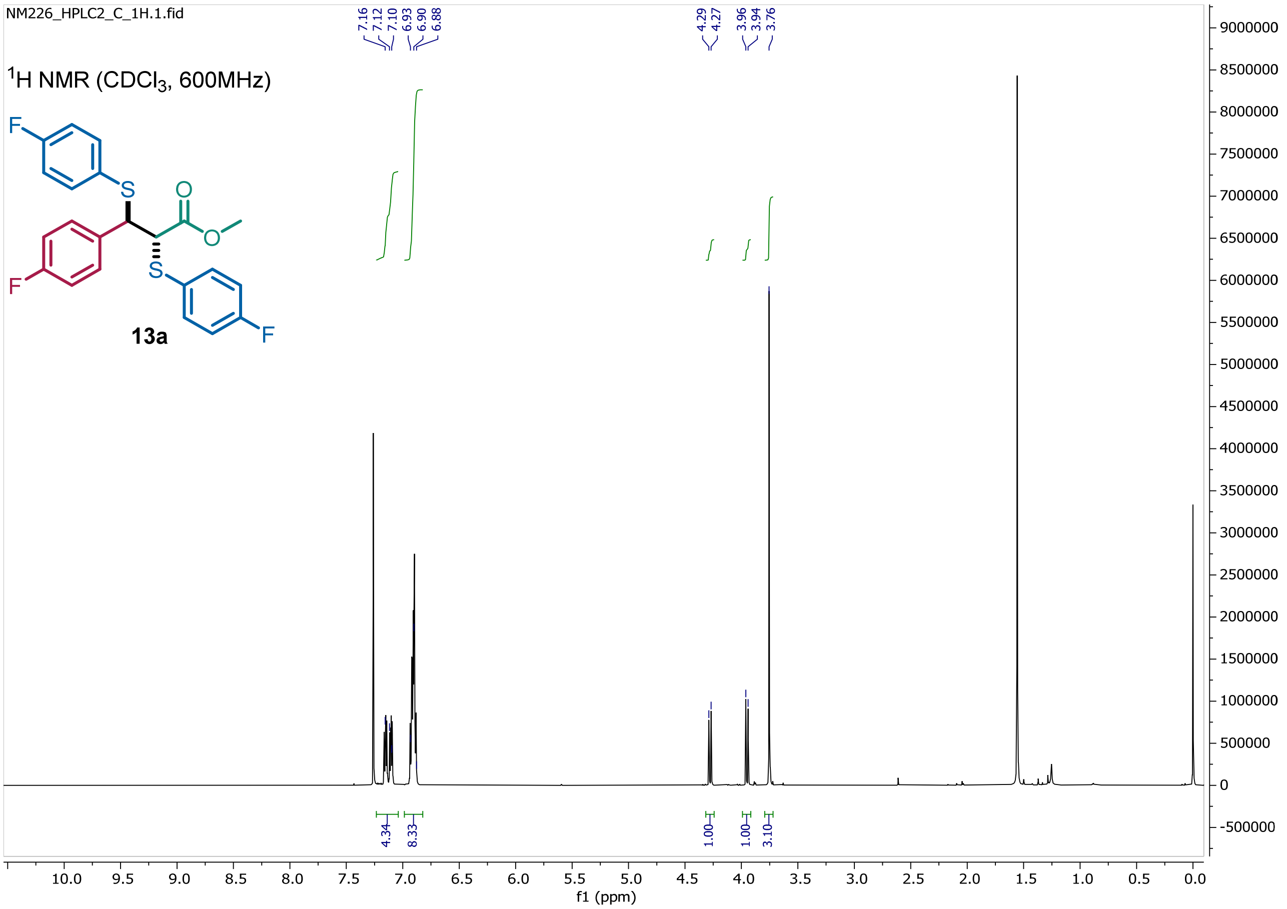
NM226_HPLC2_C_1H.1.fid
^{1}H NMR (CDCl$_3$, 600MHz)
13a
7.16
7.12
7.10
6.93
6.90
6.88
4.29
4.27
3.96
3.94
3.76
4.34
8.33
1.00
1.00
3.10
f1 (ppm)

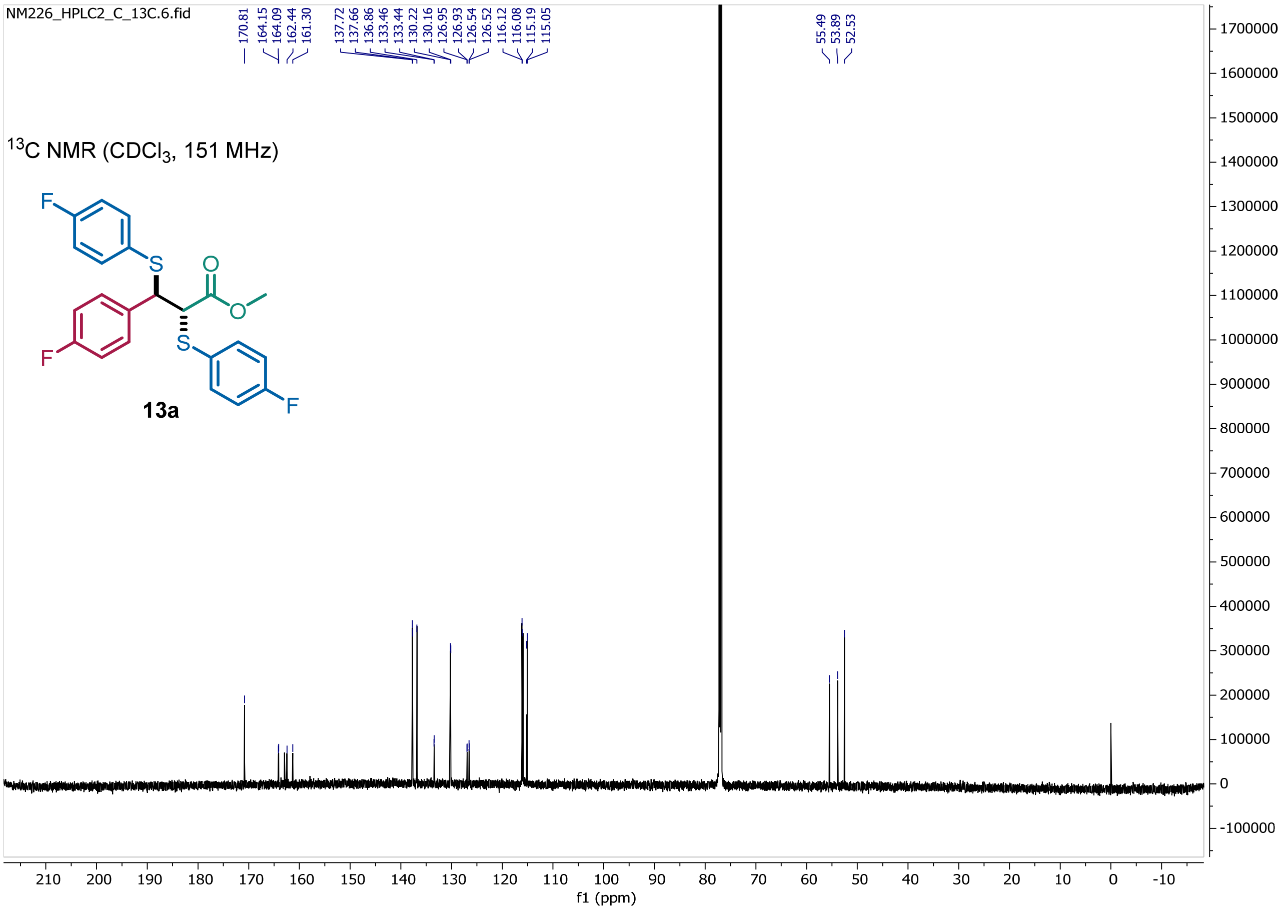
NM226_HPLC2_C_13C.6.fid
^{13}C NMR (CDCl$_3$, 151 MHz)
13a
170.81
164.15
164.09
162.44
161.30
137.72
137.66
136.86
133.46
133.44
130.22
130.16
126.95
126.93
126.54
126.52
116.12
116.08
115.19
115.05
55.49
53.89
52.53
f1 (ppm)

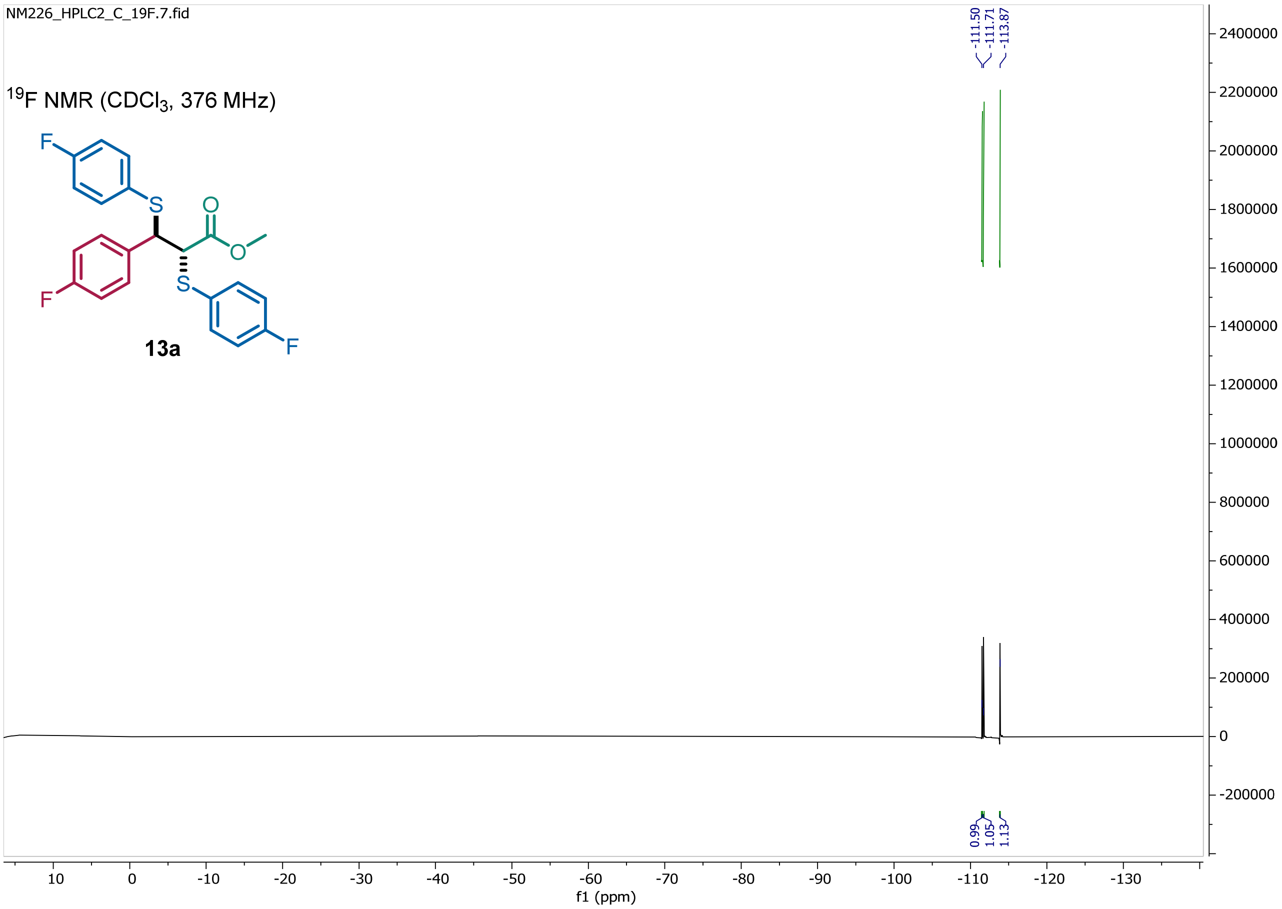
NM226_HPLC2_C_19F.7.fid
19F NMR (CDCl3, 376 MHz)
13a
-111.50
-111.71
-113.87
0.99
1.05
1.13
f1 (ppm)

Mechanistic studies

Our efforts to rationalize the reaction pathway of this multicomponent process started by putting forward several mechanistic hypotheses. Experiments were then set up to assess the viability of these hypotheses. Some of these experiments, especially the ones discarding the least-likely pathways, were performed at the early stages of the project, using the achiral **DPP** catalyst and under un-optimized reaction conditions.

Hypothesis 1: Corey-Chaykovsky epoxidation followed by ring-opening

A first mechanistic scenario involves a Corey-Chaykovsky epoxidation between the ylide **1** and the aldehyde **2**, followed by a nucleophilic ring opening of the *trans*-epoxide intermediate **11a** by the thiophenol **3** (Scheme S8).

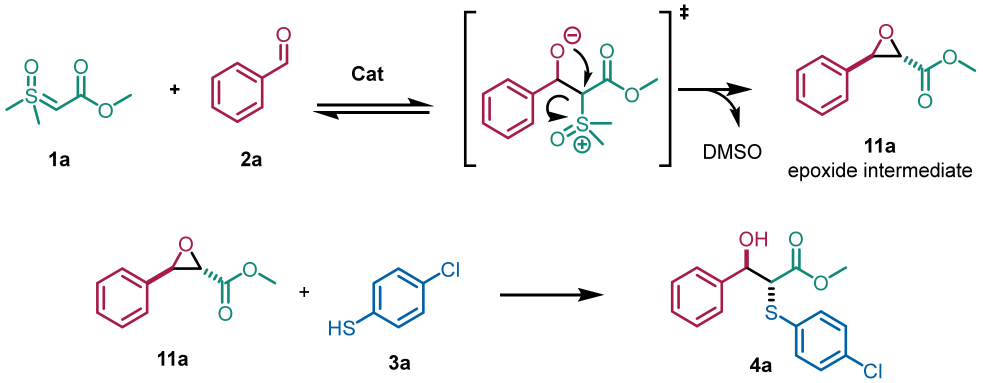

Scheme S8*. Hypothesis 1: Corey-Chaykovsky epoxidation followed by ring-opening with thiophenols.*

This possibility did not appear so likely, since the Corey-Chaykovsky epoxidation with stabilized sulfoxonium ylides is not common. Moreover, epoxides were never detected in the crude mixtures of the reactions. However, since the Corey-Chaykovsky epoxidation is well known for the more reactive un-stabilized sulfoxonium ylides, control experiments were performed to exclude this pathway.

First, only ylide **1a** and aldehyde **2a** were mixed in toluene, in the presence of **DPP** catalyst, without adding thiophenol **3a**. We did not observe any trace of *cis*- or *trans*- epoxide (Scheme S9). The ylide was however totally consumed, and ^{1}H NMR showed a complex mixture of products. It is important to note that the epoxide was not detected at short reaction times. Thus, the mixture of products obtained after 24 h is likely not the result of the degradation of the epoxide.

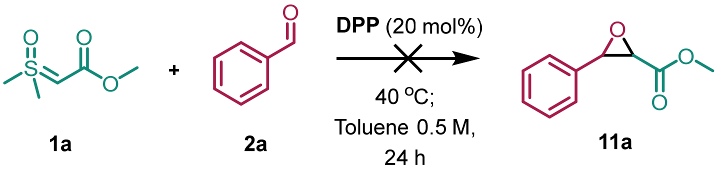

Scheme S9*. Attempted formation of the putative epoxide intermediate* ***11a*** *via a Corey-Chaykovsky reaction.*

Then, the commercially available *trans*-epoxide **11a** was reacted with thiophenol **3a** under catalytic conditions (Scheme S10). No formation of product **4a** was noticed, and this time the starting materials did not show any conversion.

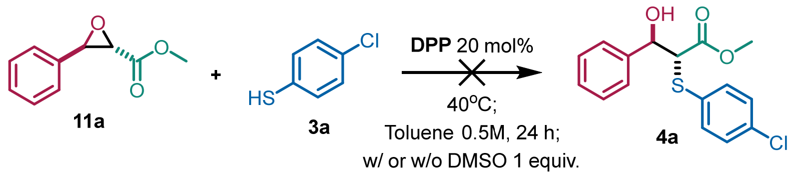

Scheme S10*. Attempted ring-opening of the epoxide* ***11a*** *by thiophenol* ***3a*** *under the reaction conditions.*

Overall, these experiments convincingly demonstrated that the epoxidation is not involved in the reaction pathway.

Hypothesis 2: X-H insertion reactions followed by enol catalysis

Considering the aptitude of stabilized sulfoxonium ylides to undergo insertion into polarized X-H bonds, a second mechanistic hypothesis involved an X-H insertion reaction as the first step of the process. Some NMR experiments showed a strong interaction between the ylide **1a** and the **DPP** catalyst (Figure S1). The ca. +5 ppm shift of the ^{31}P NMR signal of **DPP** in the presence of excess **1a** is in line with the literature[5] and indicative of the protonation of ylide **1a** by **DPP**. Although these NMR experiments were performed in CD_3CN – a solvent more polar than the one used in the reactions – to ensure sufficient solubility, the facile protonation of the ylide **1a** suggested a possible relevance of an X-H insertion in the mechanism, since insertion reactions proceeds via protonation of the ylide by acidic catalysts.

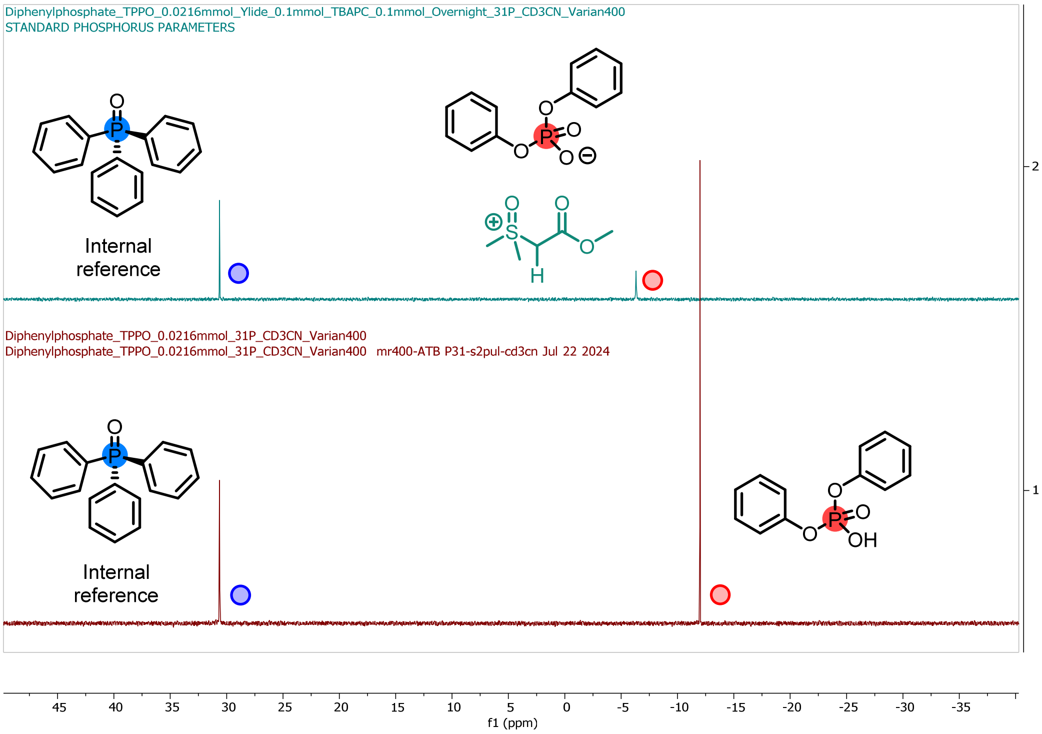

***Figure S1**. ^{31}P NMR experiments, using triphenylphosphine oxide as internal reference. Bottom: **DPP** (0.02 mmol) in CD_3CN (0.7 mL). Top: **DPP** (0.02 mmol) and **1a** (0.1 mmol) in CD_3CN (0.7 mL).*

Accordingly, an S-H insertion reaction of the ylide **1** on the thiophenol **3** could be the first step of the process, affording **6a**. Product **4a** would derive then from an acid catalyzed enolization of this product **5** and subsequent aldol addition ("enol catalysis[6]" (Scheme S11)).

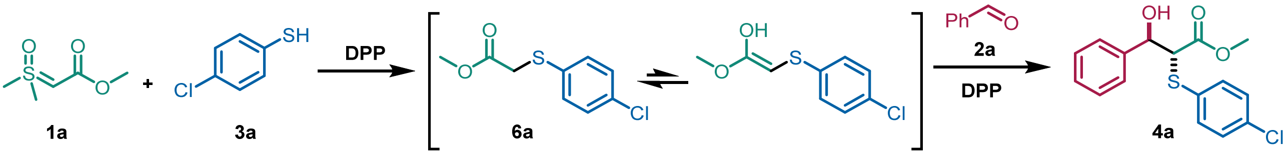

***Scheme S11**. Hypothesis 2: S-H insertion reaction followed by aldol reaction via enol catalysis.*

Thus, we synthetized product **6a** and we tested it under the reaction conditions with benzaldehyde **2a** (Scheme S12). However, we did not obtain any trace of product **4a**. This mechanistic hypothesis could be thus discarded.

[5] W. Guo, M. Wang, Z. Han, H. Huang, J. Sun, *Chem. Sci.* **2021**, *12*, 11191–11196.
[6] I. Felker, G. Pupo, P. Kraft, B. List, *Angew. Chem. Int. Ed.* **2015**, *54*, 1960–1964.

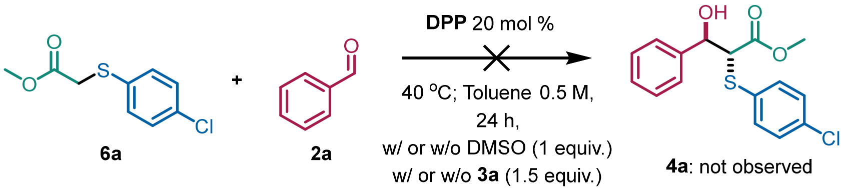

***Scheme S12**. Attempted aldol reaction between **6a** and **2a** via enol catalysis.*

It was then considered that the sulfoxonium ylide could undergo instead an insertion reaction into the O-H bond of the catalyst, resulting in product **II** (Scheme S13). The enol tautomer of **II** could attack the aldehyde. The final step would be the nucleophilic displacement of the phosphate (**III**) by the thiophenol, leading to the formation of product **4** so restoring the catalyst.

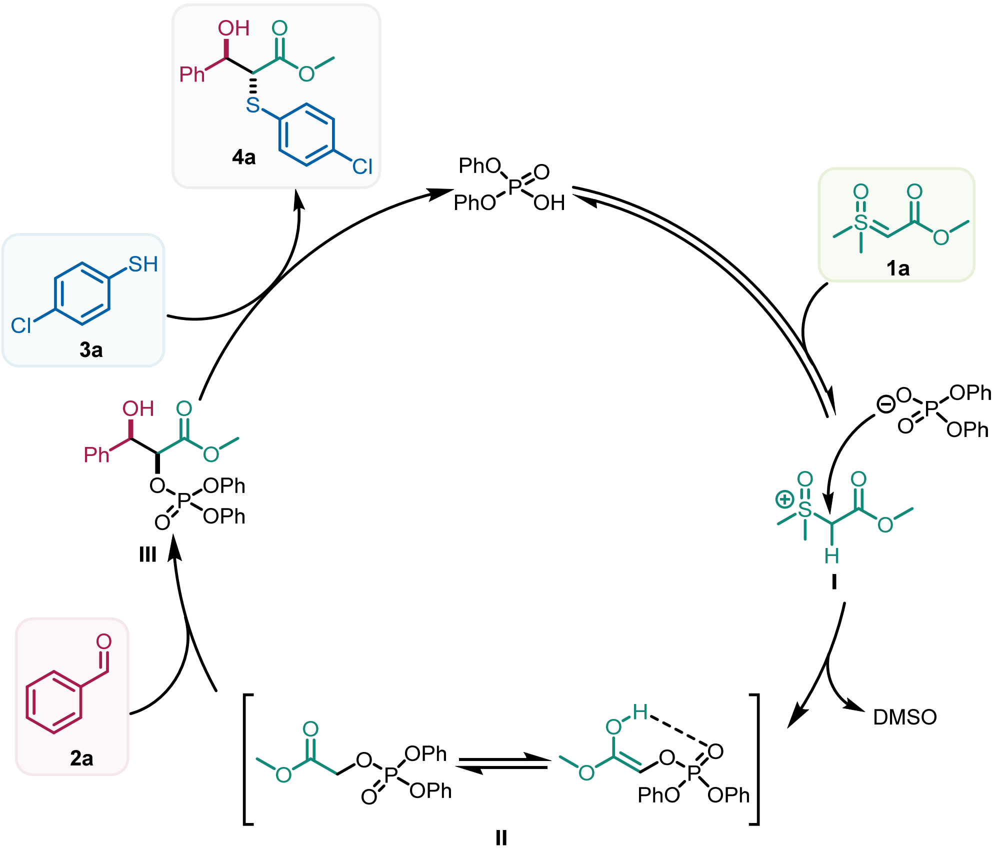

***Scheme S13**. Hypothesis 2: O-H insertion followed by aldol reaction and S_N2 displacement.*

Although the formation of **II** was not observed neither in the NMR experiments nor in the catalytic reactions, we synthetized an O-H insertion product **II** using a reported procedure[7], and subjected it to the reaction conditions, using **2i** and **3d** as aldehyde and thiophenol substrates (Scheme S14). No reactivity was observed, making this pathway unlikely too.

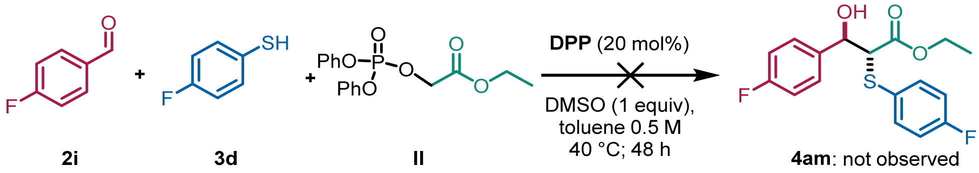

***Scheme S14**. Attempted use of an O-H insertion product **II** as reaction intermediate.*

[7] F. Tan, W. Wang, X. Huang, Y. Zhong, T. Song, J. Wang, L. Mei, *J. Org. Chem.* **2024**, *89*, 2588–2598.

Hypothesis 3: addition to the aldehyde followed by DMSO displacement ("carbenoid reactivity")

The experiments described in the previous section suggested that the NMR-observed protonation is off-cycle. Thus, a neutral, nucleophilic ylide, possibly complexed with the catalyst[8], attacks the aldehyde **2** (Scheme S15). The intermediate formed should be electrophilic and prone to undergo DMSO displacement by the thiophenol **3**, exploiting the driving force typical of X-H insertion chemistry (release of DMSO).

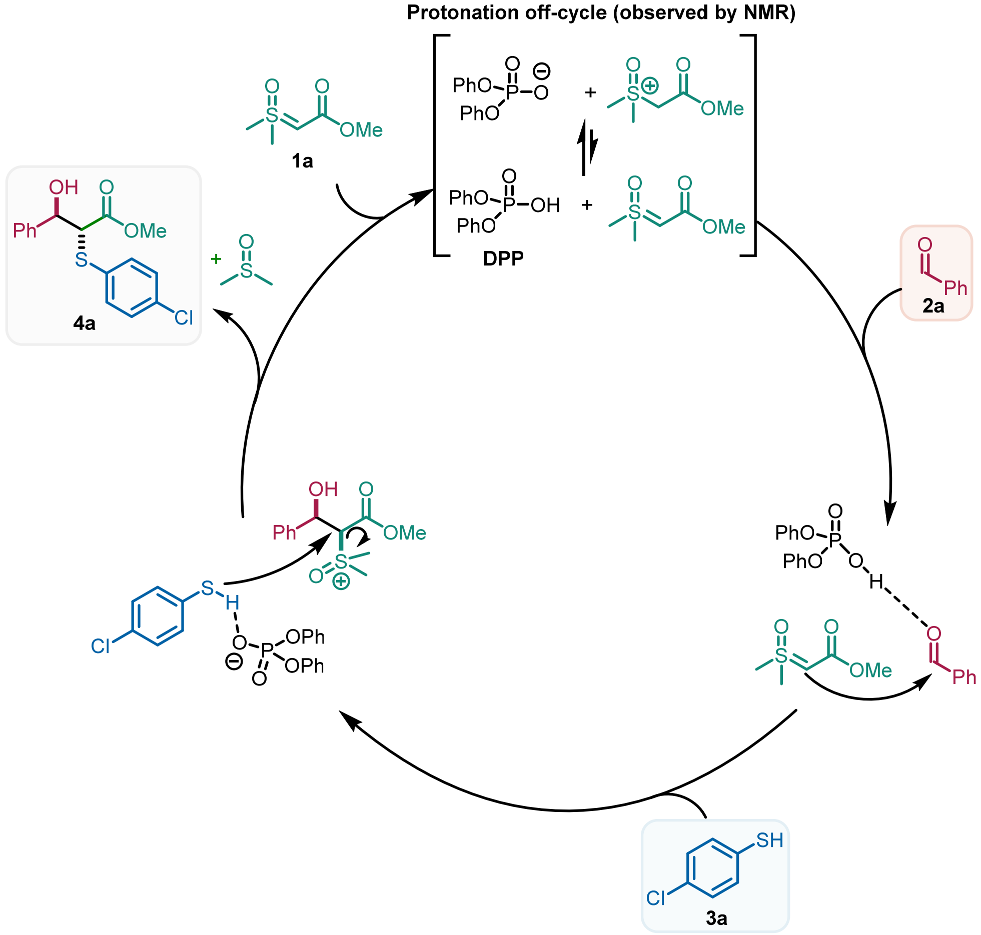

***Scheme S15**. Hypothesis 3: nucleophilic attack of the ylide **1** to the aldehyde **2** followed by DMSO displacement.*

However, all attempts to detect by NMR some sort of reaction intermediate/complex by mixing ylide **1a**, **DPP** catalyst and an aldehyde **2** –thus omitting thiophenol **3**- were unsuccessful. Addition of different aldehydes **2** to a mixture of ylide **1a** + **DPP** (10-20 mol%) in $CDCl_3$ or other deuterated solvents including toluene-d_8 resulted in fast consumption of the ylide **1a**, and formation of several unidentified compounds. Nevertheless, this mechanism appeared likely, and in line with the typical carbenoid-like reactivity of stabilized sulfoxonium ylides. The identification of the disubstituted ylide **5a** as main product at short reaction times did not exclude this pathway, since this compound could be – indeed, it is – in equilibrium with the starting substrates **1**, **2** and **3**. Thus, ylide **5** could be an off-cycle species (Scheme S16), and product **4a** form via the catalytic cycle outlined in Scheme S15.

[8] M. Ke, J. Zheng, J. Zong, K. Tang, J. Wang, G. Zheng, B. Zhang, D. Cheng, Z. Ju, F. Chen, *Chem. Sci.* **2025**, *16*, 8108–8113.

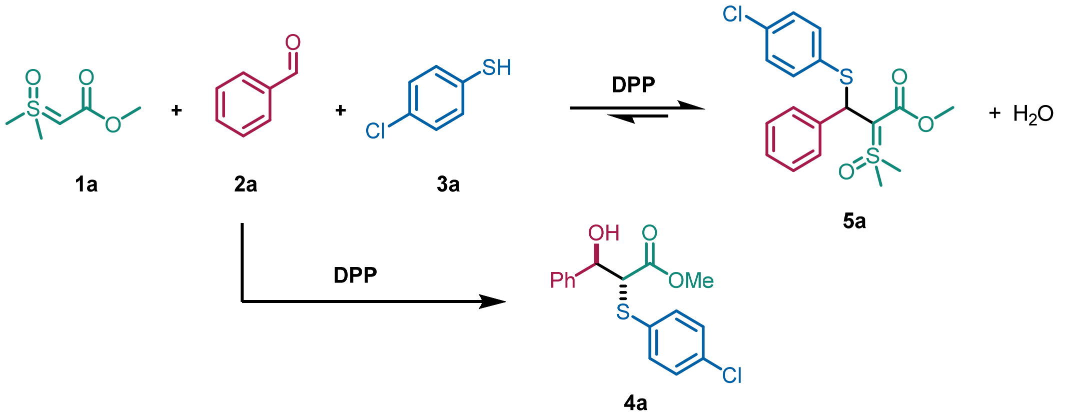

***Scheme S16**. Hypothesis 3 with disubstituted ylide **5a** as off-cycle species.*

However, it was not possible to reconcile this pathway with the results of the experiments performed with labelled (^{18}O) compounds (see section "Identification of the oxygen source of the hydroxyl moiety"). The lack of ^{18}O incorporation in product **4a** using either ^{18}O-**2a** or excess $H_2{}^{18}O$ as additive is not compatible with this mechanistic hypothesis, which was thus discarded.

Mechanistic proposal

Proposal overview

Ultimately, elaborating the results obtained from several experiments, described in the following subsections, it was possible to formulate a plausible mechanistic proposal, discussed in the main text of the article (Scheme S17).

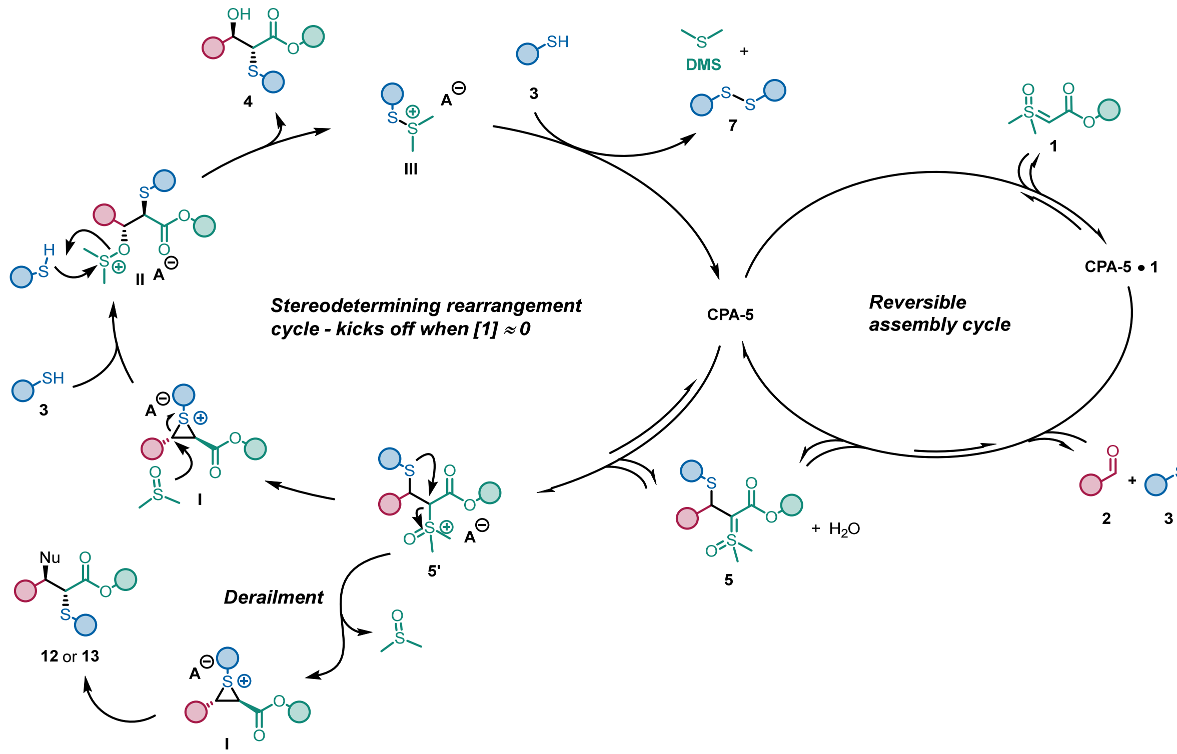

***Scheme S17.** Plausible catalytic cycle accounting for the experimental results.*

This proposal accounts for all experimental observations, including the formation of the detected side- and co-products. In fact, the formation of the by-products **12** and **13** (Figure S2), observed in some instances during the study of the reaction, can be rationalized considering derailments of the rearrangement cycle. In both cases, these products may originate from the ring opening of the episulfonium ion **I** by an external nucleophile, instead of DMSO. Compound **13a** was observed only in polar solvents like CH_3CN and MeOH. Enhanced solvent polarity likely facilitates the disruption of weak interactions between DMSO and the episulfonium ion, thereby promoting competitive nucleophilic

attack by other nucleophiles – specifically the thiophenol – present in the reaction medium. **DPP**-trapped species **12a** may derive instead from the ring opening of the episulfonium ion by its phosphate counterion[9]. This type of side-product was not observed with the chiral catalyst **CPA-5**. It is probably due to the steric hindrance of the chiral catalyst **CPA-5**, which does not allow the chiral catalyst to act as a nucleophile.

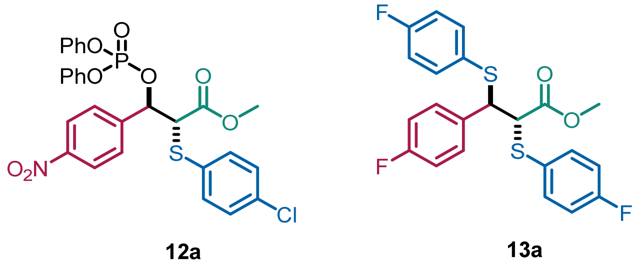

***Figure S2**. Isolated side-products **12a** and **13a** derived from the ring opening of the episulfonium ion by an external nucleophile (vs DMSO).*

It is important to stress that, according to this proposal, three equivalents of thiophenol **3** are present in the balanced reaction equation, of which one gets embedded in the product **4** and two are sacrificed as disulfide **7**. This would make the thiophenols **3** the limiting reagents in our procedures, which typically employ a 2.5:1 molar ratio between thiophenols **3** and ylides **1**. However, recognizing that other species such as water might concur at the dimethylsulfonium displacement from **II**[10], it was preferred to keep considering the ylide **1** as the limiting reagent.

In the next sections, some of the experiments that led to this mechanistic proposal are reported and discussed.

*Possible pathways for the formation of α,α-disubstituted ylide **5***

The first hypothesis that was put forward accounting for the formation of ylide **5** is shown in Scheme S18 and involves the addition of the ylide **1** to the aldehyde **2**, followed by deprotonation of the sulfoxonium adduct resulting in formation of a β-hydroxy α,α-disubstituted ylide, and ultimately substitution of the hydroxyl group of this ylide by the thiophenol **3** via a vinyl sulfoxonium ion. The last step reminds of the reported reactivity of β-hydroxy α-diazo carbonyl compounds with nucleophiles in the presence of Lewis acids[11].

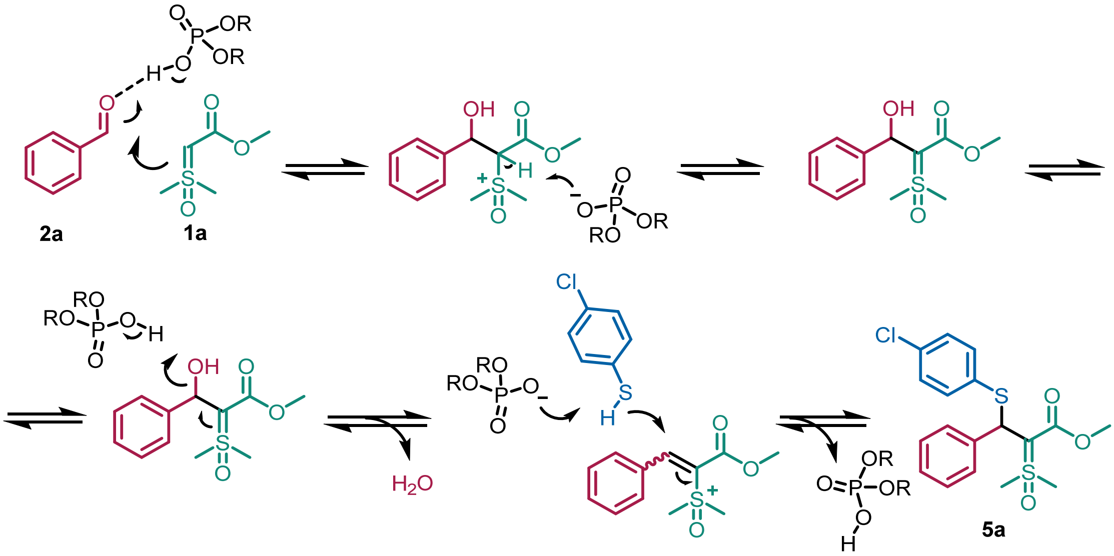

***Scheme S18**. First hypothesis for the formation of **5a**: addition to the aldehyde **2a** followed by substitution of the hydroxyl group with thiol **3a**.*

[9] F. Duarte, R. S. Paton, *J. Am. Chem. Soc.* **2017**, *139*, 8886–8896.
[10] Y. Ashikari, T. Nokami, J.-i. Yoshida, *Org. Lett.* **2012**, *14*, 938–941.
[11] A. M. Peck, M. Brewer, *Org. Lett.* **2023**, *25*, 2647–2651.

However, as stated earlier, all attempts to detect by NMR adducts deriving from the reaction between aldehydes **2** and ylides **1** in the presence of **DPP** were unsuccessful. Addition of different aldehydes **2** to a mixture of ylide **1a** + **DPP** (10-20 mol%) in $CDCl_3$ or other deuterated solvents including toluene-d_8 resulted in fast consumption of the ylide **1a**, and formation of several unidentified compounds. This prompted the formulation of alternative pathways, amongst which the one involving a reactive thionium ion intermediate, sketched in Scheme S19, appeared especially appealing. In the literature, thionium ion formed from aldehydes and thiols under acid catalysis have been proposed as transient reaction intermediates[12,13]. These chemical species have short lifetimes, but high reactivity, and could form intermediate **5a** by suffering addition of the ylide **1a** followed by deprotonation of adduct **5'a**. Since **5'a** is an intermediate in the rearrangement cycle, which does not start until ylide **1a** is present, its deprotonation must be accompanied by the complexation/trapping of the catalyst by ylide **1a**.

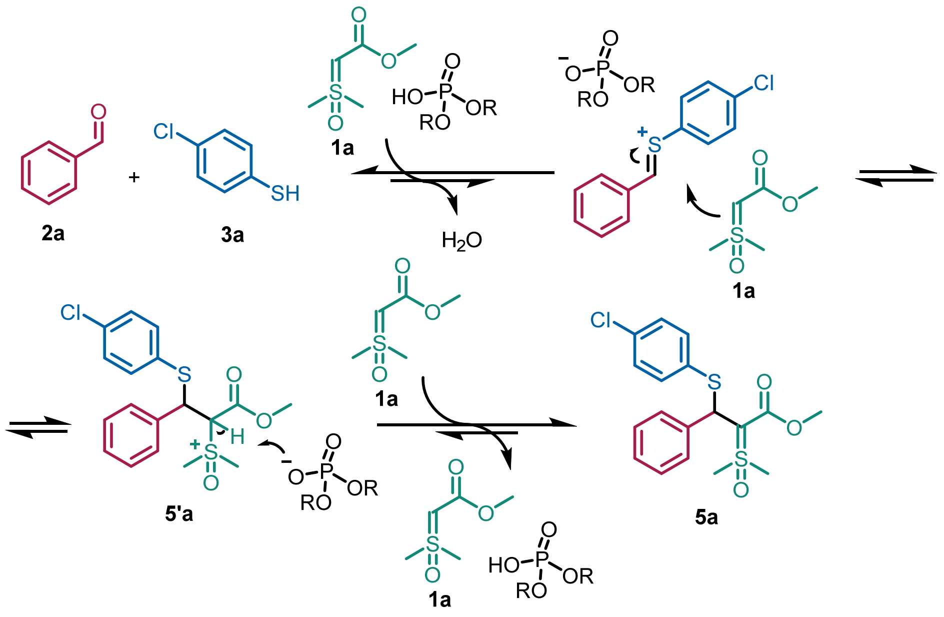

Scheme S19. *Second hypothesis for the formation of **5a**: addition to a transient thionium ion.*

To gain some hints on the viability of this hypothesis, the idea has been to generate the thionium ion under other conditions, using reported procedures, and check if the stabilized sulfoxonium ylide **1a** would react with it. Since the Pummerer reaction is one of the most common reactions involving thionium ion intermediates (Scheme S20), the focus was set on this reaction to check if its intermediate could be intercepted by a sulfoxonium ylide **1**.

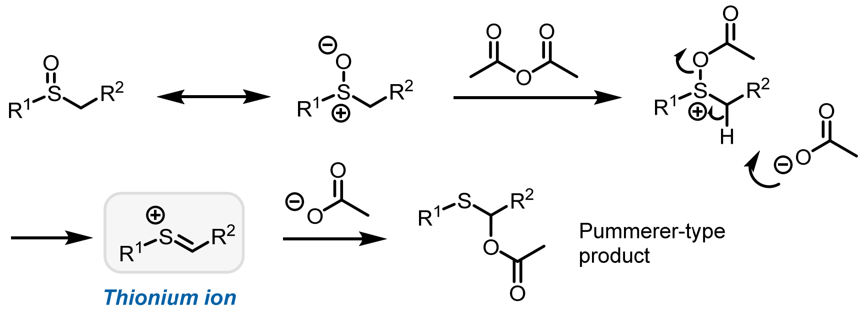

Scheme S20. *The Pummerer reaction.*

To do that, benzylphenylsulfoxide was reacted with ylide **1a** under Pummerer conditions - that is, in the presence of TFAA and a base. In a reaction where ylide **1a** was added after 10 minutes, formation of product **4a** was observed by ^{1}H NMR and HPLC-MS, alongside its trifluoroacetylated derivative **4'a**. Both products might stem from intermediate **5**: compound **4a** *via* the rearrangement, and **4'a** *via* the trapping of the episulfonium ion by trifluoroacetate, or

[12] L. H. S. Smith, S. C. Coote, H. F. Sneddon, D. J. Procter, *Angew. Chem. Int. Ed.* **2010**, *49*, 5832–5844.
[13] R. Parnes, S. Narute, D. Pappo, *Org. Lett.* **2014**, *16*, 5922–5925.

trifluoroacetylation of **4a**. Thus, this experiment suggests that ylides **1** have a suitable reactivity towards thionium ions, and that the second hypothesis justifying the formation of **5** involving a thionium ion may be more likely than the first.

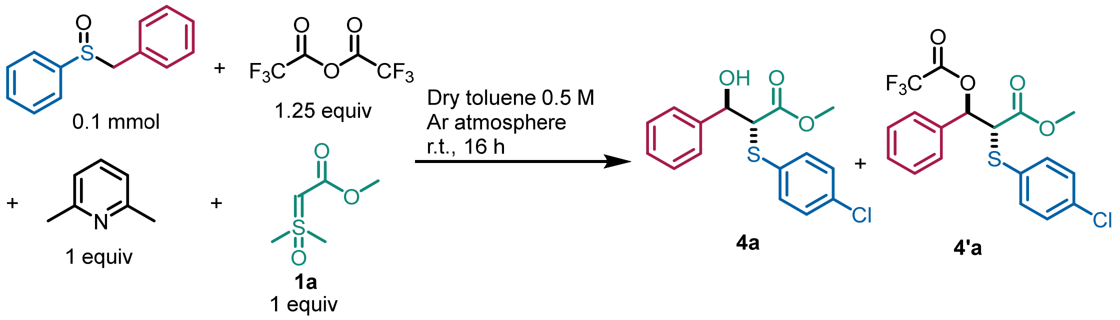

Scheme S21. *Evidence of formation of product **4a** in the reaction of ylide **1a** with a thionium ion formed under Pummerer conditions.*

Enantiodetermining step

The experiments with labelled additives, and *in situ* ^{1}H NMR, show that intermediates **5** evolve to products **4** via a formal rearrangement under phosphoric acid catalysis. However, these experiments did not clarify which step of the reaction is the enantiodetermining one - that is, if the configuration at the sulfur-bearing chirality center of **5** establishes the configuration of the ultimate product **4**, or not. Unfortunately, CSP-HPLC analyses of ylide **5a** were inconclusive, due to its relatively poor stability. Therefore, a few control experiments were performed.

The first experiment consisted in the synthesis of a potentially enantioenriched ylide **5a** using the optimal chiral catalyst **CPA-5** (Scheme S22). Following the isolation of the ylide **5a**, this species was subjected to the reaction conditions in the presence of the achiral phosphoric acid **DPP**. This effectively eliminated any potential source of chirality within the reaction mixture in the rearrangement step. The hypothesis was that, if the ylide **5a** itself was enantioenriched at this stage, at least a minor degree of enantioenrichment in the product **4a** would be observed. However, product **4a** was obtained in a fully racemic form, suggesting that the product configuration is not established during the first step.

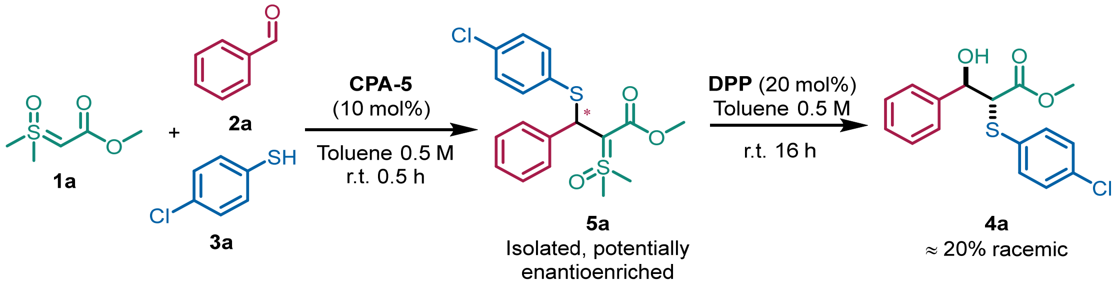

Scheme S22. *Control experiment showing that **5a** leads to rac-**4a** using the achiral **DPP** catalyst during the rearrangement.*

In the second control experiment, the approach was reversed (Scheme S23). First, the disubstituted ylide (**5a**) was synthetized with an achiral catalyst (**DPP**). The isolated ylide **5a** was then subjected to the reaction conditions using **CPA-5** as chiral catalyst. In this experiment, from a racemic disubstituted ylide (**5a**), product **4a** was afforded with an enantiomeric excess comparable to the one obtained under standard enantioselective conditions.

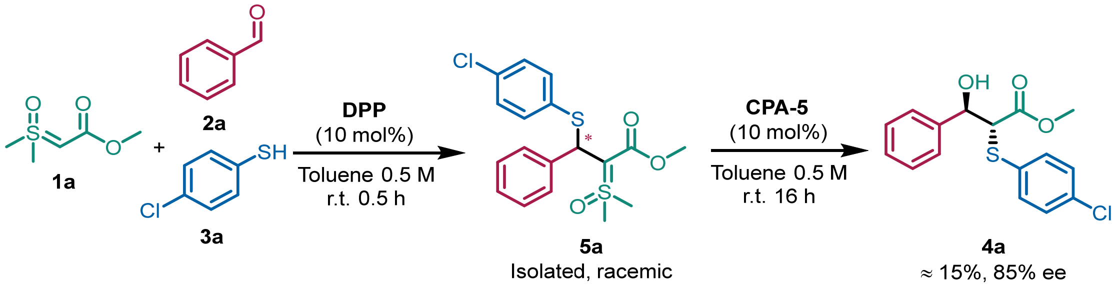

Scheme S23*. Control experiment showing that racemic* ***5a*** *leads to enantioenriched* ***4a****.*

From these results, it is possible to affirm that the configuration of the product **4** is established during the rearrangement, presumably during the formation of the episulfonium ion **I**, implying a racemization event at the chirality center of **5**. Overall, we rationalize the stereoselectivity of the process with the DYKAT scenario depicted in Scheme S24 for product **4a**.

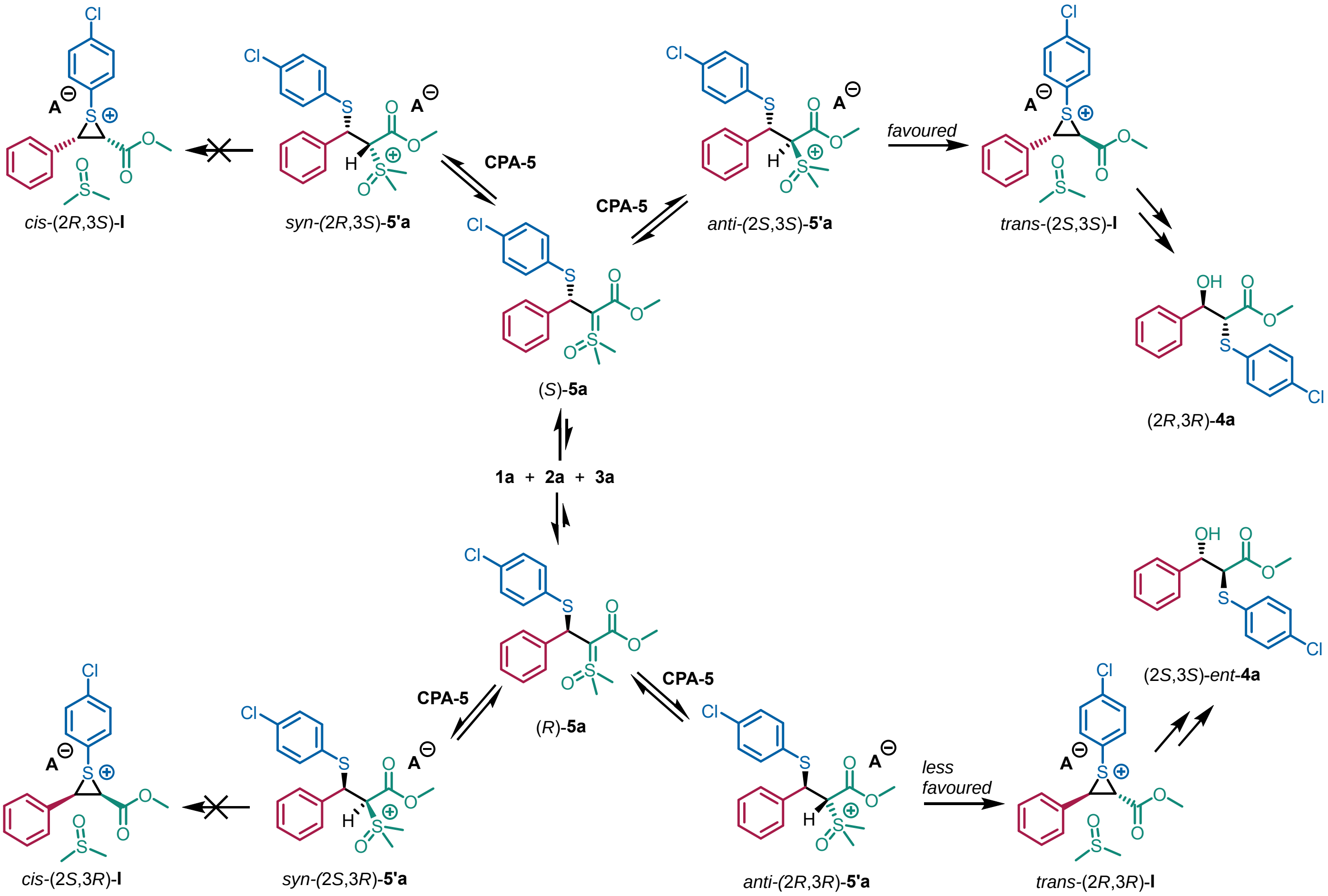

Scheme S24*. DYKAT scenario rationalizing the stereoselectivity of the process.*

This scenario involves a fast racemization of ylide **5a** via equilibration with starting materials **1a**-**3a**, fast and reversible protonation of ylide **5a** by **CPA-5**, followed by the rate- and stereo-determining ring-closure of sulfoxonium salt **5'a** to the episulfonium **I**. Since the MCR proceeds with full *anti*-diastereoselectivity, we surmise that only the *anti*-isomers of sulfoxonium salts **5'a** undergo ring-closure to the corresponding *trans*-episulfonium ions. Given the absolute configuration of product **4a** obtained in the reaction, formation of (2*S*,3*S*)-episulfonium is favoured over its enantiomeric counterpart. At the present stage, it is not clear whether reversible protonation of ylide **5a** by catalyst **CPA-5** delivers all isomers of **5'a**, or if such protonation occurs with some selectivity too. Nevertheless, one of the Hammett studies reported in the section “Hammett's plots accounting for benzaldehyde and thiophenol electronics.” showed clearly that the conversion of ylides **5** to products **4** is dramatically accelerated by electron donating groups on the sulfur atom. This is fully consistent with the hypothesis that the formation of the episulfonium ion is the rate

determining step of the rearrangement cycle, and that this is the step that determines the diastereo- and enantioselectivity of the whole process. While electron donating groups at sulfur could also be beneficial in subsequent steps of the rearrangement cycle (*e.g.* **II** → **III**, or **III** → **7**, Scheme S17), another Hammett study showed that the rate of the rearrangement is influenced also by the substituents on the aldehydes **2**. Such substituents would be unlikely to influence the **II** → **III** or **III** → **7** steps, thus reinforcing the hypothesis that episulfonium ion formation is the key stereodetermining step of the MCR.

*Rearrangement of **5** to **4**: promoters and inhibitors*

The experiments of the previous section rendered product **4a** from isolated **5a** only in low yield, comparable to the catalyst loading, due to extensive degradation of **5a**. It was found that the addition of thiophenol **3** could greatly improve the yield of the reaction (Scheme S25), accounting for its role in the rearrangement cycle as dimethylsulfonium scavenger. It seemed that, without a thiophenol, the reaction proceeds stoichiometrically - that is, thiophenol is essential for catalyst turnover. Curiously, this reaction rendered product **4a** with higher enantiomeric excess than the MCR.

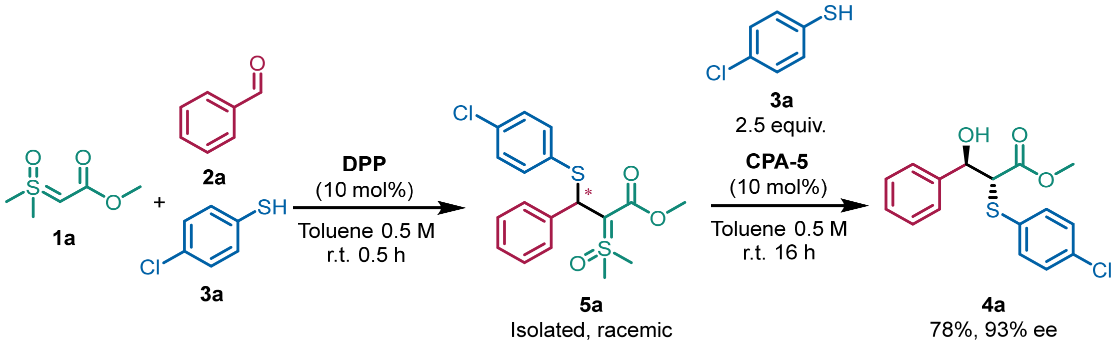

Scheme S25. *Control experiment showing that thiophenol **3a** assists the rearrangement step.*

On the contrary, the rearrangement step was substantially inhibited when ylide **1a** was added to the reaction (Scheme S26). This result, interpreted with the higher affinity of the catalyst for ylide **1a**, compared to **5a** or other species, justifies the fact that the rearrangement cycle does not start until **1a** is present in the mixture.

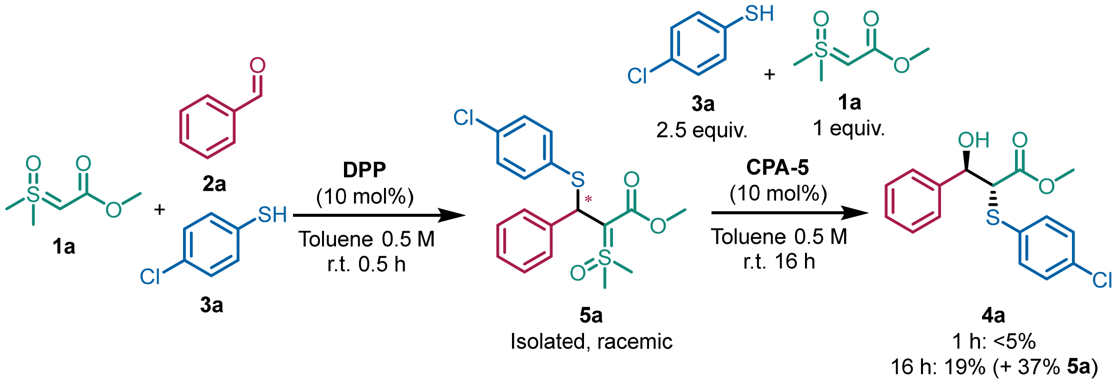

Scheme S26. *Control experiment showing that **1a** inhibits the rearrangement step.*

*Cross-over experiments between **5** and the substrates – racemization of ylide **5**.*

During the control experiments described in the previous section, we noticed the formation of small amounts of benzaldehyde **2a** and thiophenol **3a**, starting from the isolated disubstituted ylide **5a**. It was the cornerstone of the following mechanism considerations, suggesting that **5a** is in equilibrium with the starting substrates **1a**, **2a**, and **3a** thus providing the dynamic racemization pathway required to convert racemic **5a** to enantioenriched **4a** via DYKAT. To prove it, *para*-fluorothiophenol **3d** and *para*-fluorobenzaldehyde **2i** - to track products formation using ^{19}F NMR - were used to perform some scrambling experiments with non-fluorine-containing disubstituted ylide **5a**. In this way, it would have been possible to prove an equilibrium of the intermediate **5a** with its starting materials before the formation of product **4a**. This product was tested to be irreversibly formed in another control experiment.

First, the disubstituted ylide (**5a**) was subjected to the reaction conditions with 2.5 equivalents of *para*-fluorothiophenol **3d** and 20 mol% of the **DPP** catalyst (Scheme S27). From the ^{1}H and ^{19}F NMR spectra of the reaction crude, the incorporation of *para*-fluorothiophenol **3d** in the product was evident. The overall yield (ca. 70%) was calculated from the ^{1}H NMR spectrum using dibromomethane as internal standard, exploiting the overlapped benzylic doublets of the two products (Figure S3). It was not possible to determine the ratio between **4a** and **4aa** from the ^{1}H NMR spectrum, due to the lack of isolated signals for the two products. However, it was possible to estimate from the ^{19}F NMR spectrum the amount of *para*-fluorothiophenol **3d** incorporated, that is, the amount of product **4aa**, being aware that the fluoride residues could derive only from this reagent (Figure S4). Considering that 2.5 equivalents of **3d** were used, product **4aa** was determined to be around 55%.

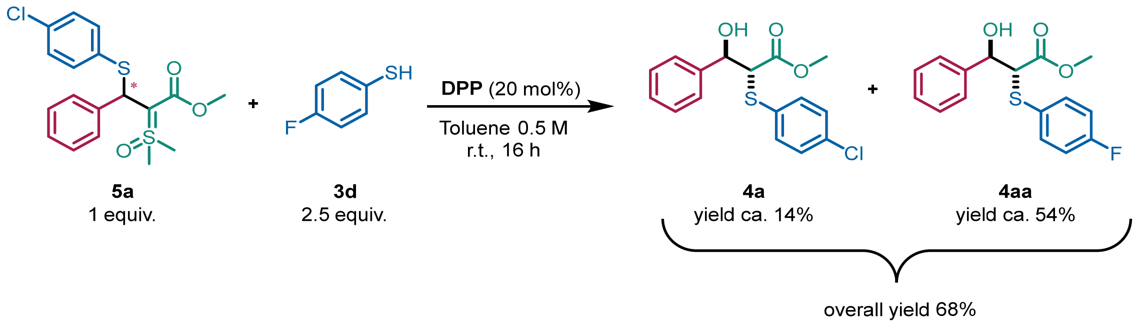

***Scheme S27**. Cross-over experiment with para-fluorothiophenol **3d**. Cross-over product **4aa** is observed.*

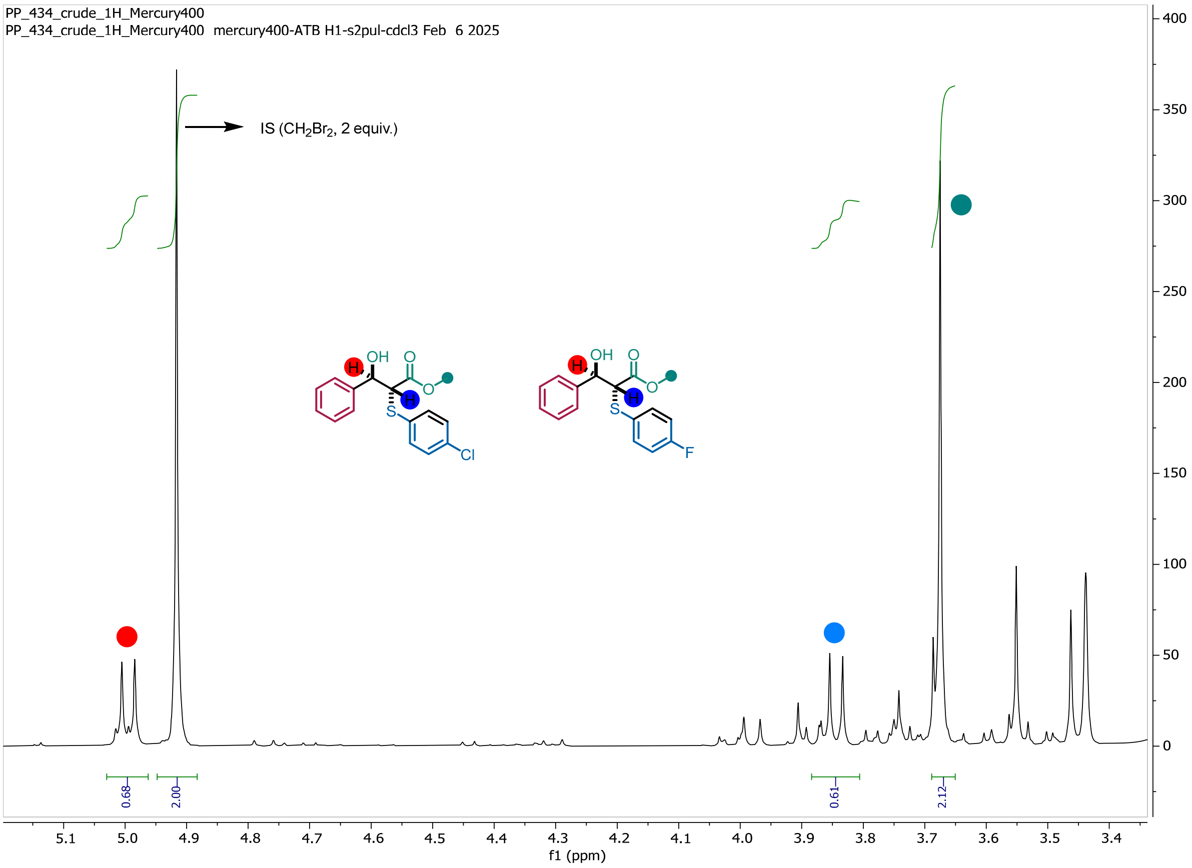

***Figure S3**. ^{1}H NMR spectrum of the crude mixture of the reaction of **5a** in the presence of **3d** (dibromomethane as internal standard).*

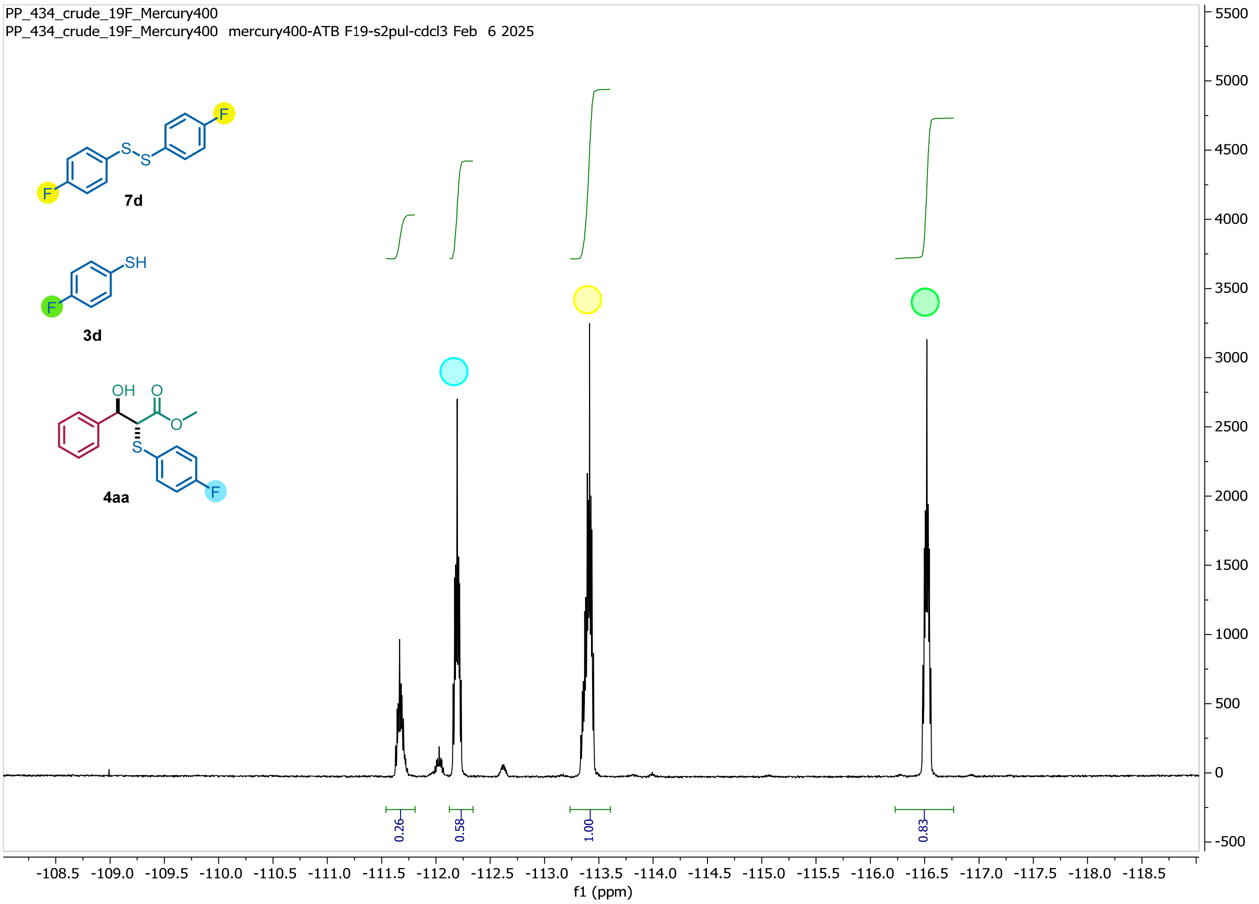

Figure S4. *^{19}F NMR spectrum of the crude mixture of the reaction of **5a** in the presence of **3d**.*

In a second cross-over experiment, *para*-fluorobenzaldehyde **2i** was used in the reaction with **5a**. *para*-Chlorothiophenol **3a** was added too, to improve the reaction efficiency. Products **4** were obtained in ca. 75% yield (Scheme S28), as measured by ^{1}H NMR using dibromomethane as internal standard (Figure S5). However, although the reaction proceeded efficiently, it furnished exclusively product **4a** without any evidence of aldehyde scrambling (see ^{19}F NMR, Figure S6).

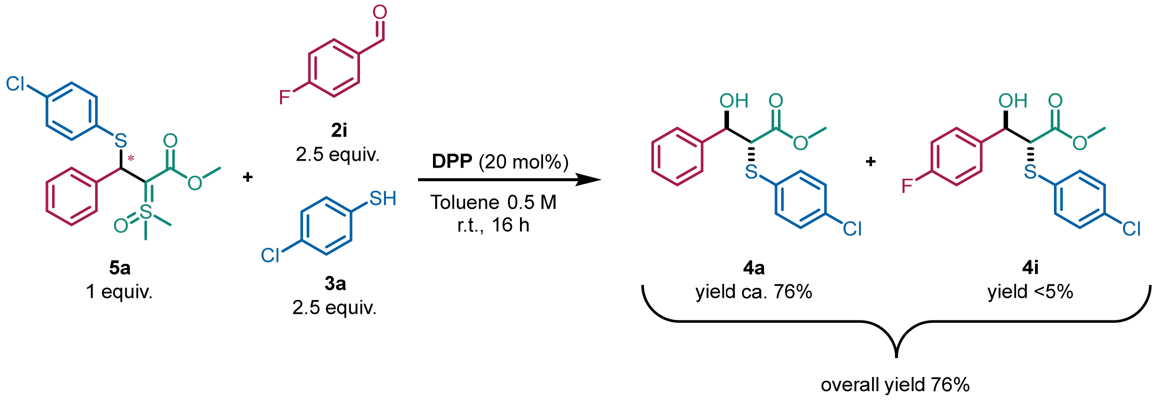

Scheme S28. *Cross-over experiment with para-fluorobenzaldehyde **2i**. Cross-over product **4i** is not observed.*

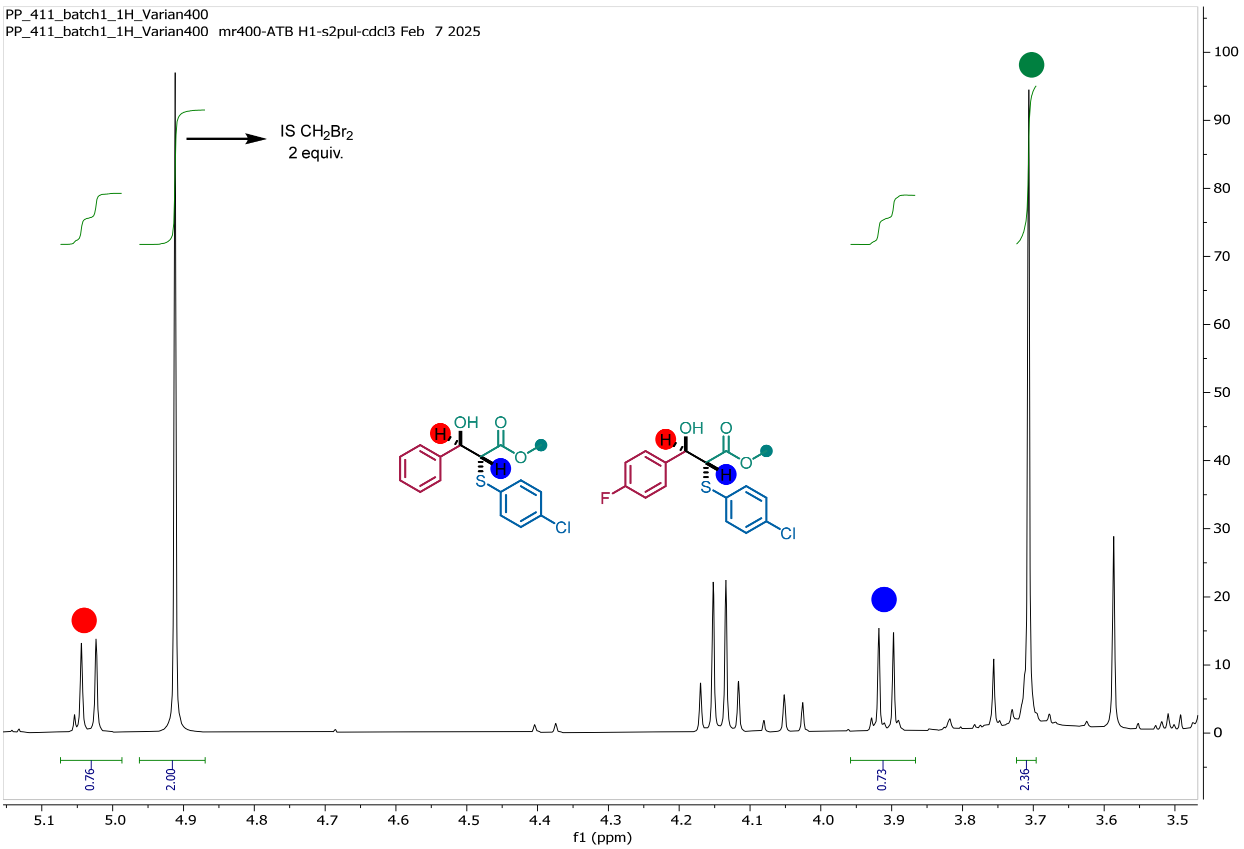

***Figure S5**. ^{1}H NMR spectrum of the crude mixture of the reaction of **5a** in the presence of **2i** and **3a**.*

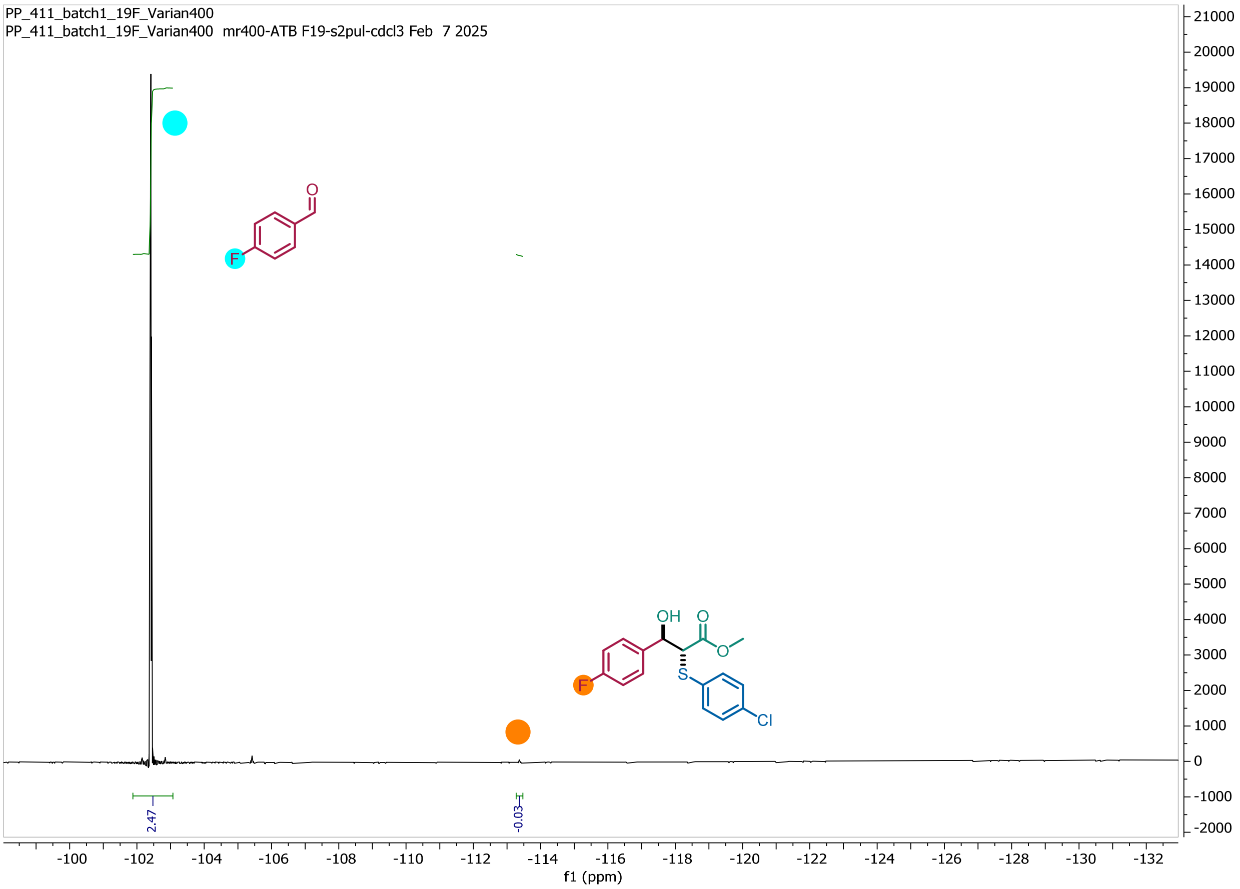

***Figure S6**. ^{19}F NMR spectrum of the crude mixture of the reaction of **5a** in the presence of **2i** and **3a**.*

Suspecting that the lack of cross-over was due to the different reactivity of the two aldehydes in the reaction, the complementary cross-over experiment was set in place. Thus, intermediate **5i** was prepared with the standard

methodology, and subjected to the reaction conditions in the presence of benzaldehyde **2a** and thiophenol **3a** (Scheme S29). In this case, aldehyde scrambling was indeed observed. ^{1}H NMR analysis indicated an overall 60% yield in the two products **4a** and **4i** but did not allow to determine their ratio due to signals overlap (Figure S7). Conversely, ^{19}F NMR analysis served only to detect *para*-fluorobenzaldehyde **2i** in the crude, suggesting a certain degree of reversibility (Figure S8). Ultimately, proof of the formation of cross-over product **4a** was achieved by HPLC-MS analysis of the crude mixture, which indicated the presence of both **4a** and **4i** species (Figure S9).

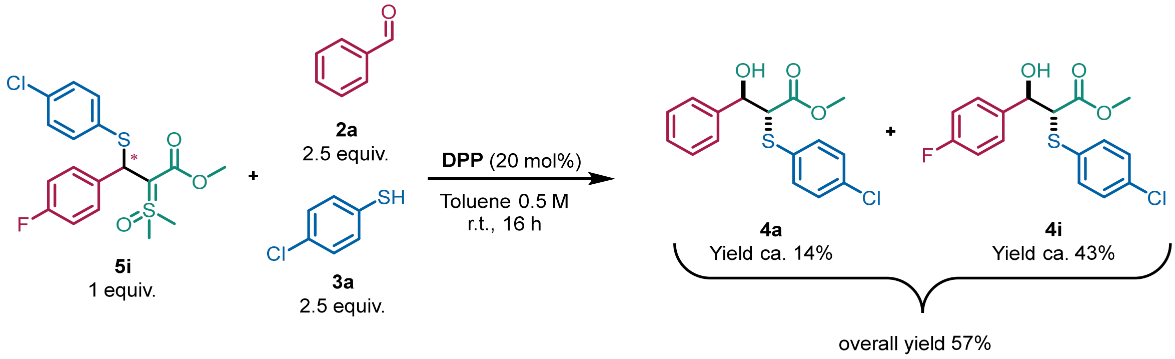

***Scheme S29**. Cross-over experiment with benzaldehyde **2a**. Cross-over product **4a** is observed.*

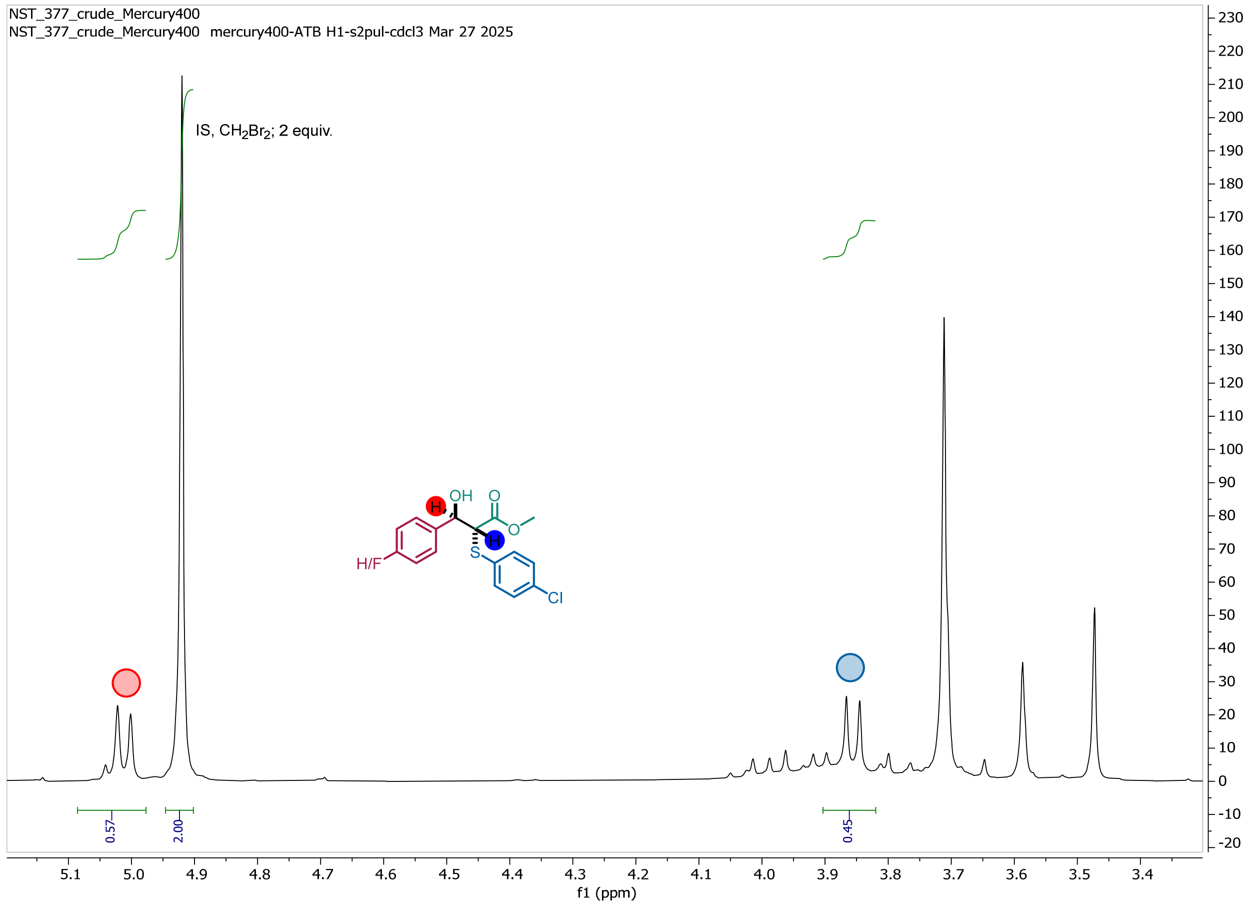

***Figure S7**. ^{1}H NMR spectrum of the crude mixture of the reaction of **5i** in the presence of **2a** and **3a**.*

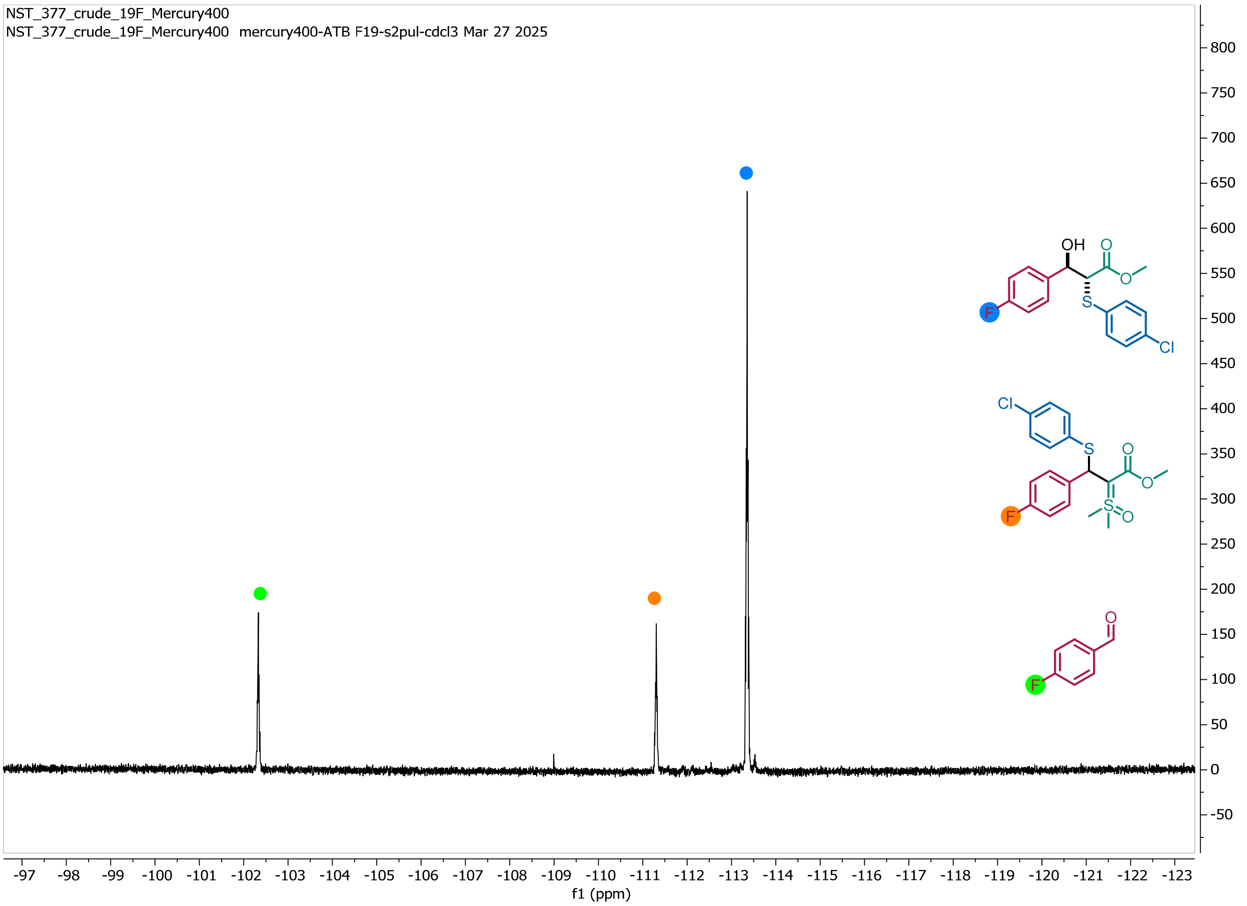

Figure S8. ^{19}F NMR spectrum of the crude mixture of the reaction of ***5i*** in the presence of ***2a*** and ***3a***.

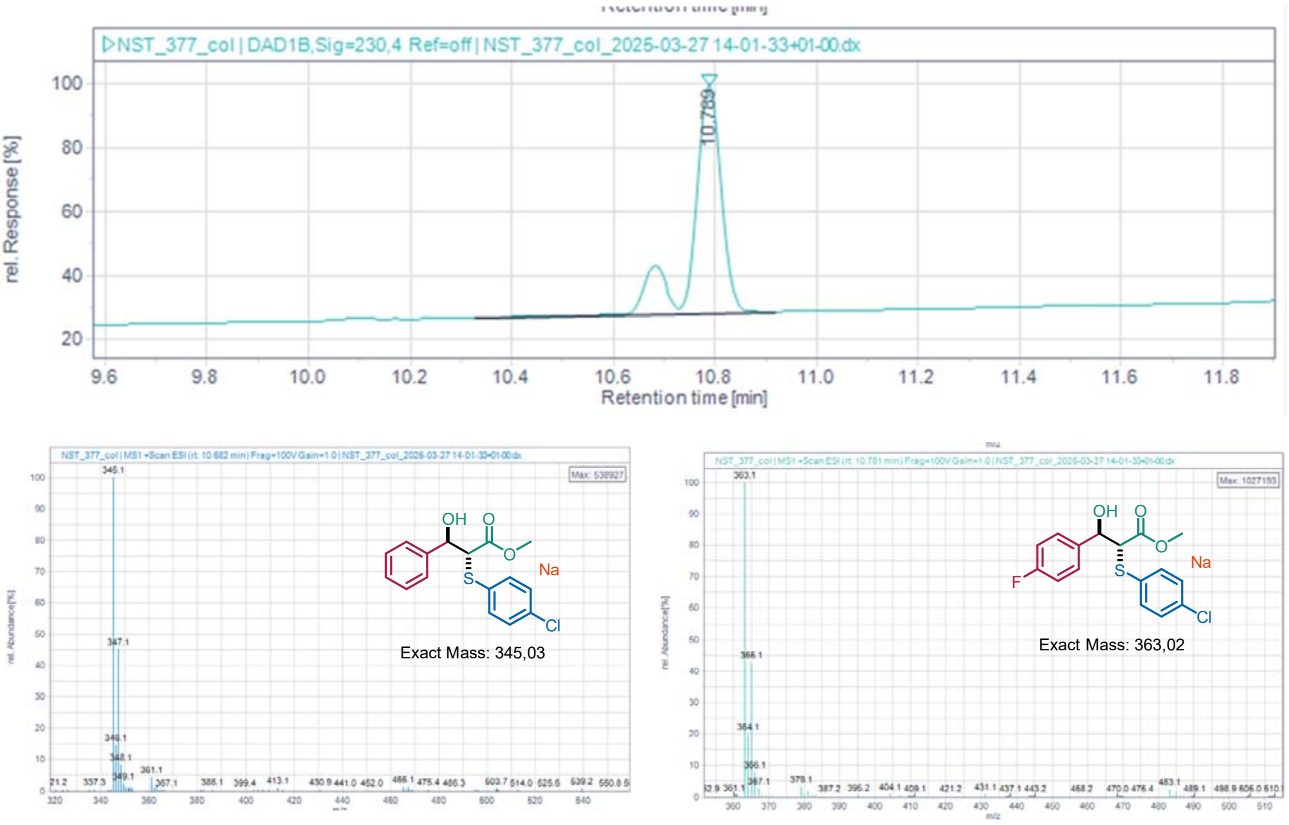

Figure S9. HPLC-MS chromatogram of the crude mixture of the reaction of ***5i*** in the presence of ***2a*** and ***3a***, and ESI-MS spectra of the bands at 10.68 and 10.79 min.

The cross-over experiment with the *para*-fluorothiophenol **3d** appears as convincing evidence that intermediates **5** are in equilibrium with the starting thiols **3**, demonstrating the viability of racemization pathways accounting for the control of the configuration of products **4** via a DYKAT on compounds **5**. Combined with the experiments with the aldehyde substrate **1**, the thionium ion is suggested as possible intermediate species, although the moderate degree of cross-over observed with the aldehyde does not fully exclude alternative scenarios.

*Identification of the oxygen source of the hydroxyl moiety of products **4***

The key experiments that hinted towards the rearrangement of **5** as the second step of the process involved the aldehyde, additives and ylide labelled with ^{18}O, allowing to identify the origin of the oxygen atom of the hydroxyl group of products **4**.

First, since these experiments were performed with product **4a**, it is important to underline that the mass spectrum of this product, carrying chloride and sulfur atoms, presents a significant [M+2] peak, due to the natural isotopic abundance of these elements. For this reason, the standard unlabeled substrate **4a** was analyzed by HPLC-MS, which renders [M + Na^+] signals in the ESI-MS spectrum (Figure S10). The intensity of the [M + Na^+ +2] peak was found to be 36.6% of the [M + Na^+] peak, and this value was used as the reference to evaluate the ^{18}O incorporation of the product **4a** in the reactions with ^{18}O-labelled substrates/additives.

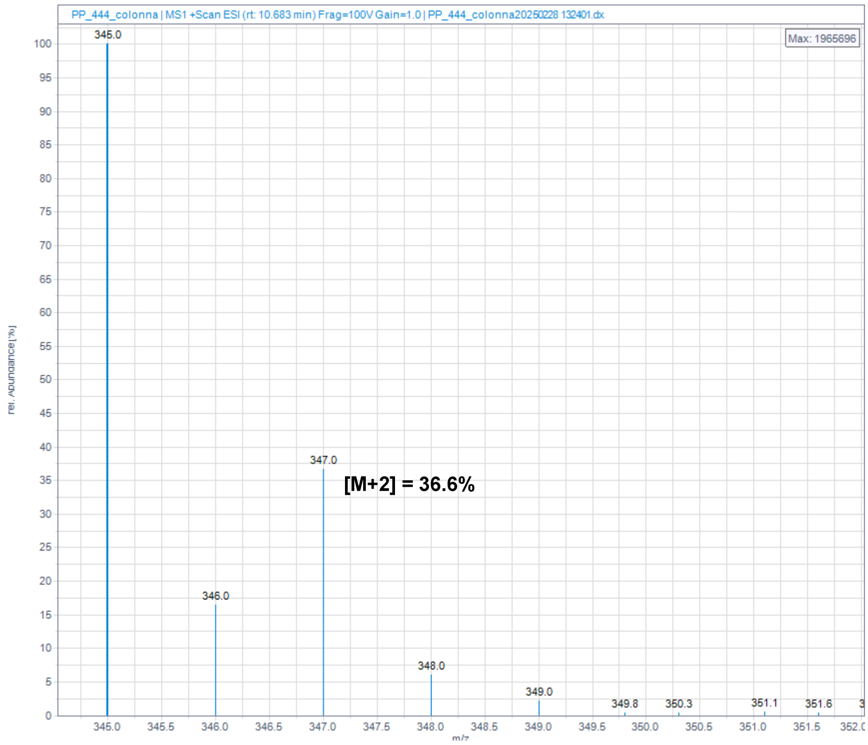

***Figure S10**. ESI-MS spectrum of unlabeled compound **4a** (zoomed on the region of M + Na^+).*

The first experiments were performed with ^{18}O-labelled water and benzaldehyde, and these experiments excluded all oxygen sources except DMSO. Before these experiments, it was verified that the reactions could be run under dry conditions, which are necessary to avoid false results from adventitious (thus unlabeled) water. The results reported in Scheme S30 show that the reaction renders similar results in dry (9 ppm water content, by Karl Fischer measurement) and untreated toluene.

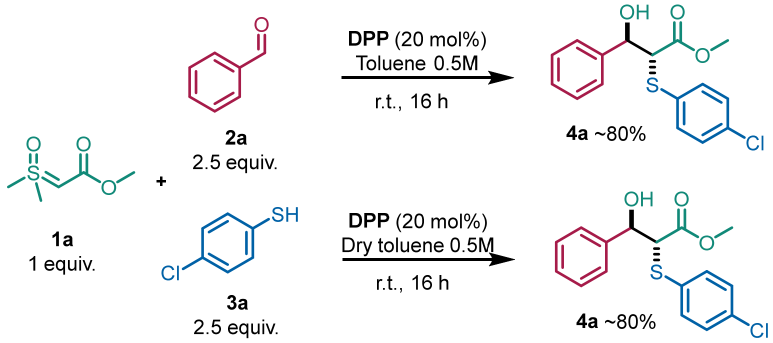

Scheme S30. *Reaction performed in untreated and dry toluene.*

Then, it was also verified that incorporation of labelled oxygen does not occur by water exchange directly on the product **4a**, in the presence of an acidic catalyst. Unlabeled *rac*-**4a** was treated with an excess (2.5 equivalents) of $H_2{}^{18}O$ in the presence of **DPP**, under rigorously dry conditions (Scheme S31). This experiment led to only a minor incorporation of ^{18}O in the product (Figure S11).

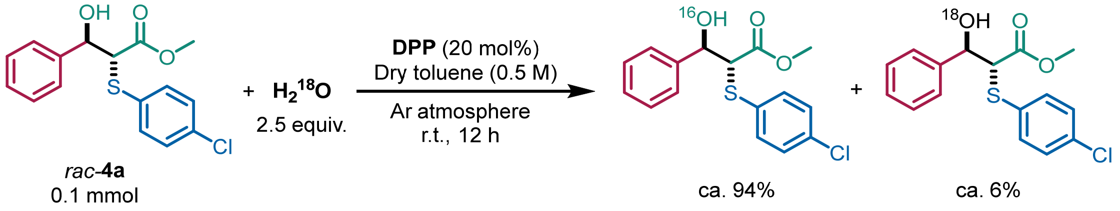

Scheme S31. *Minor ^{18}O incorporation into product **4a** when treated with $H_2{}^{18}O$.*

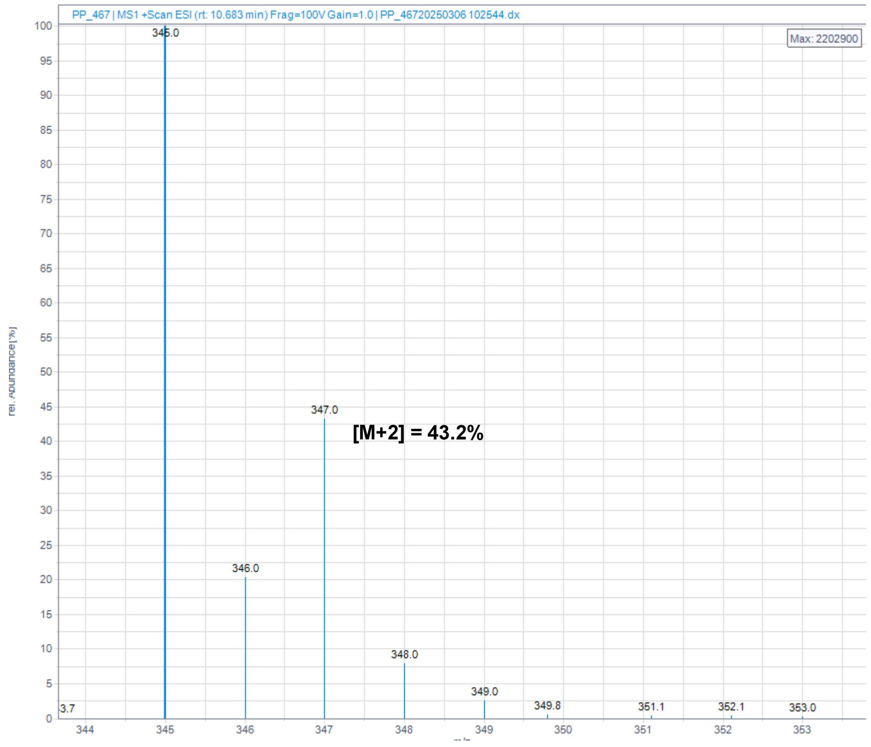

Figure S11. *ESI-MS of the crude reaction mixture resulting from treatment of unlabeled **4a** with $H_2{}^{18}O$ (zoomed on in the region of M + Na$^+$).*

A set of experiments was then conducted with a commercial batch of benzaldehyde labelled with ^{18}O (95% of ^{18}O) (Scheme S32). All the experiments were performed using a freshly opened bottle, to avoid oxygen exchange with the air moisture. The experiments 2 and 3 with the chiral catalyst **CPA-5** showed a negligible incorporation of ^{18}O into product **4a** (Figure S12), while experiment 1 with **DPP** catalyst showed a small degree of incorporation, compatible

with the value observed by putting in contact unlabeled **4a** with $H_2{}^{18}O$ in the presence of **DPP** (Scheme S31). Indeed, during the reaction, $H_2{}^{18}O$ forms.

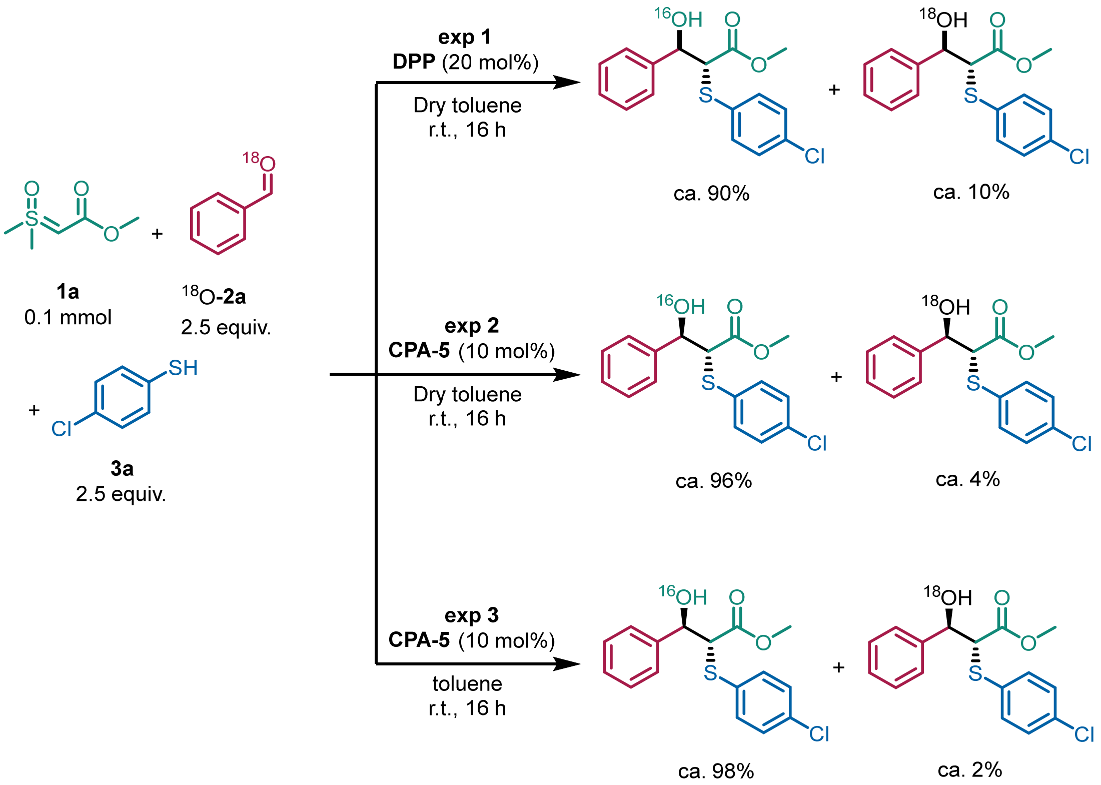

Scheme S32. *Experiments with labelled benzaldehyde* ^{18}*O-**2a**.*

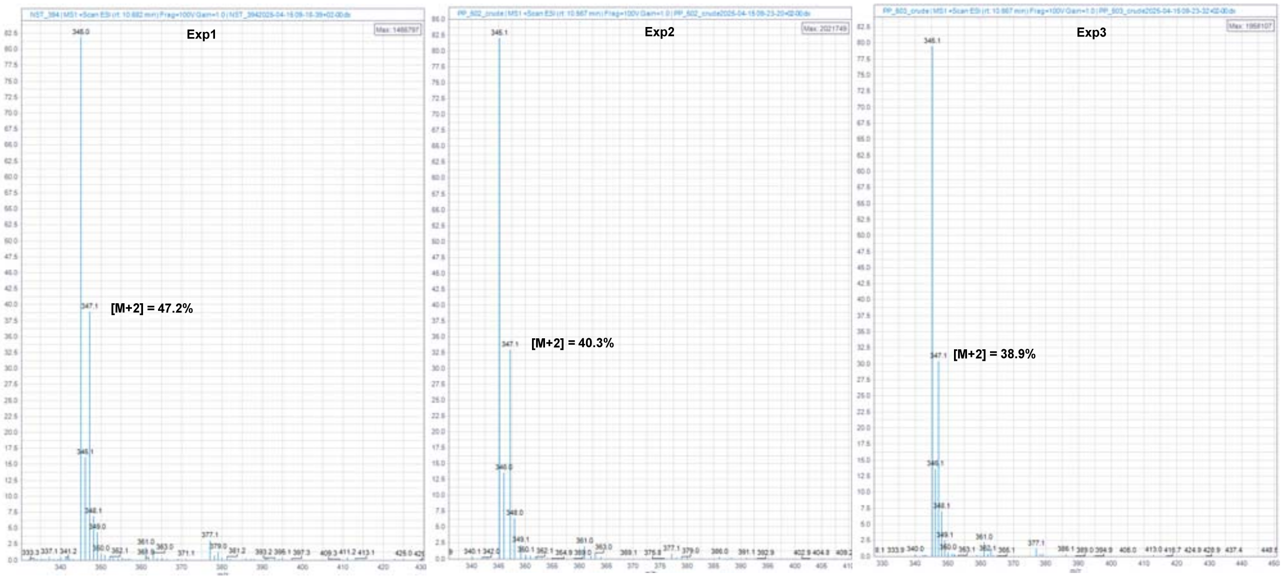

Figure S12. *ESI-MS of products **4a** obtained in the experiments with labelled benzaldehyde* ^{18}*O-**2a** (zoomed on in the region of M + Na*$^+$*). The percentage of the [M+ 2] peak was adjusted as [M + 2]/[M]%, to take into account that the [M] peak is not the base peak in these spectra.*

These experiments provide evidence that the oxygen atom of the hydroxyl group of the product **4a** does not derive from benzaldehyde **2a**, nor from the water resulting from a condensation reaction involving benzaldehyde. That is, the reaction does not proceed through addition of the ylide **1** to the aldehyde **2**, followed by DMSO displacement (see the section: Hypothesis 3: addition to the aldehyde followed by DMSO displacement). To exclude in full the involvement of water, several experiments using ^{18}O-labeled water were then performed.

In the first experiments, 2.5 equivalents of $H_2{}^{18}O$ were added to the 3C reaction performed using either **DPP** or **CPA-5** catalyst (Scheme S33). These experiments furnished an [M+2] peak of less than 39.9%, corresponding to a minor (ca. 3%) ^{18}O incorporation in **4a** (Figure S13).

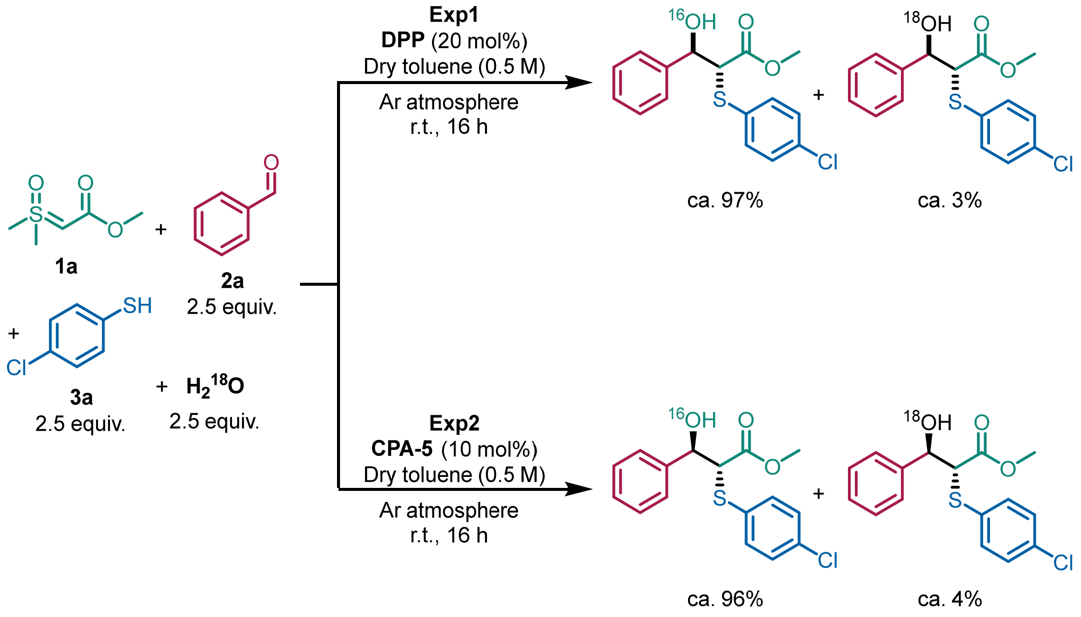

Scheme S33. *Catalytic 3C reactions performed in the presence of $H_2{}^{18}O$.*

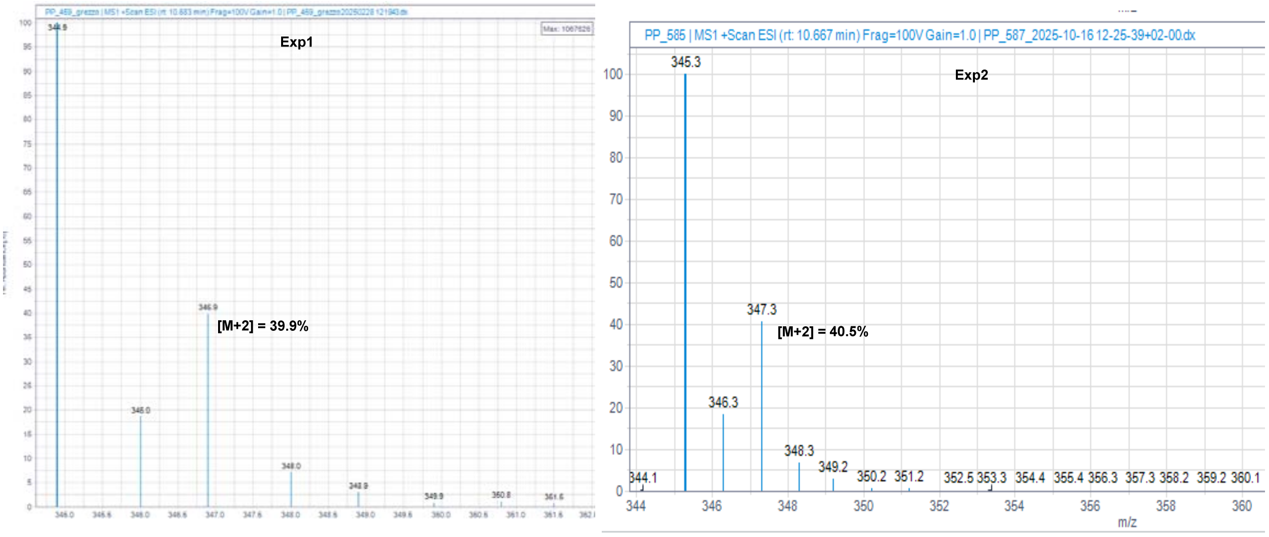

Figure S13. *ESI-MS of products **4a** obtained in the catalytic MC reactions performed in the presence of $H_2{}^{18}O$ (zoomed on in the region of $M + Na^+$).*

Then, two experiments with isolated *rac*-**5a** and 2.5 equivalents of $H_2{}^{18}O$ were performed, using catalysts **DPP** or **CPA-5**, and thiophenol **3a** to assist the conversion of **5a** to **4a** (Scheme S34). In this way, unlabeled water produced in the condensation reaction giving **5a** is not present in the system. Using either **DPP** or **CPA-5** catalyst, the product **4a** contained less than 11% of ^{18}O (Figure S14).

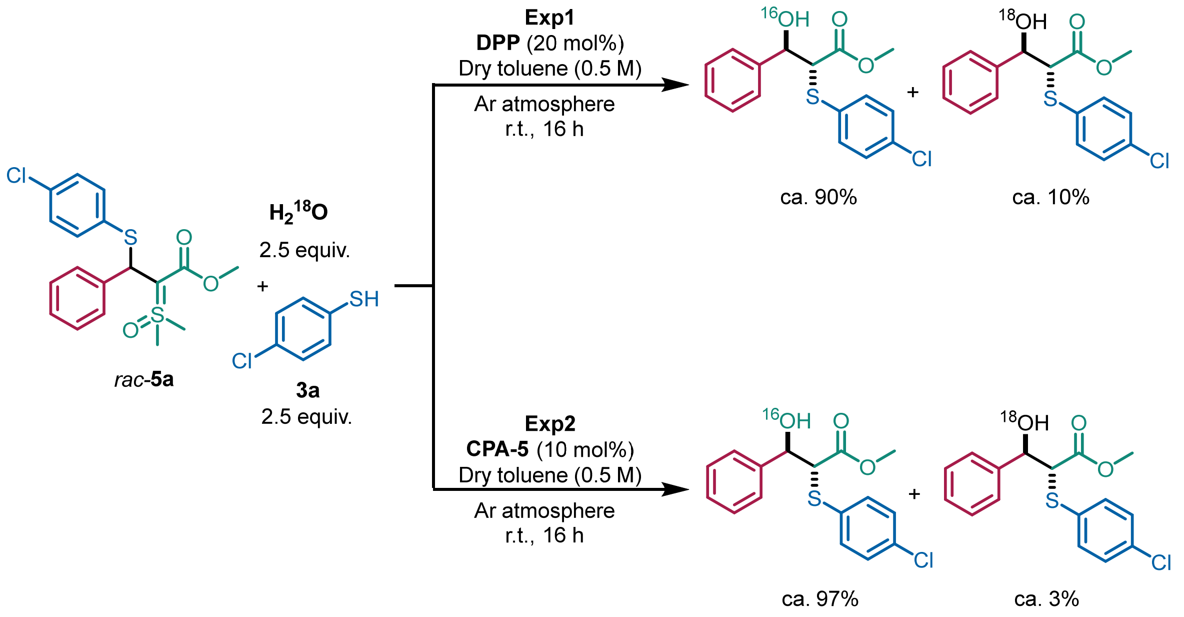

Scheme S34. *Conversion of rac-**5a** to **4a** catalysed by **DPP** and **CPA-5** performed in the presence of* $H_2{}^{18}O$.

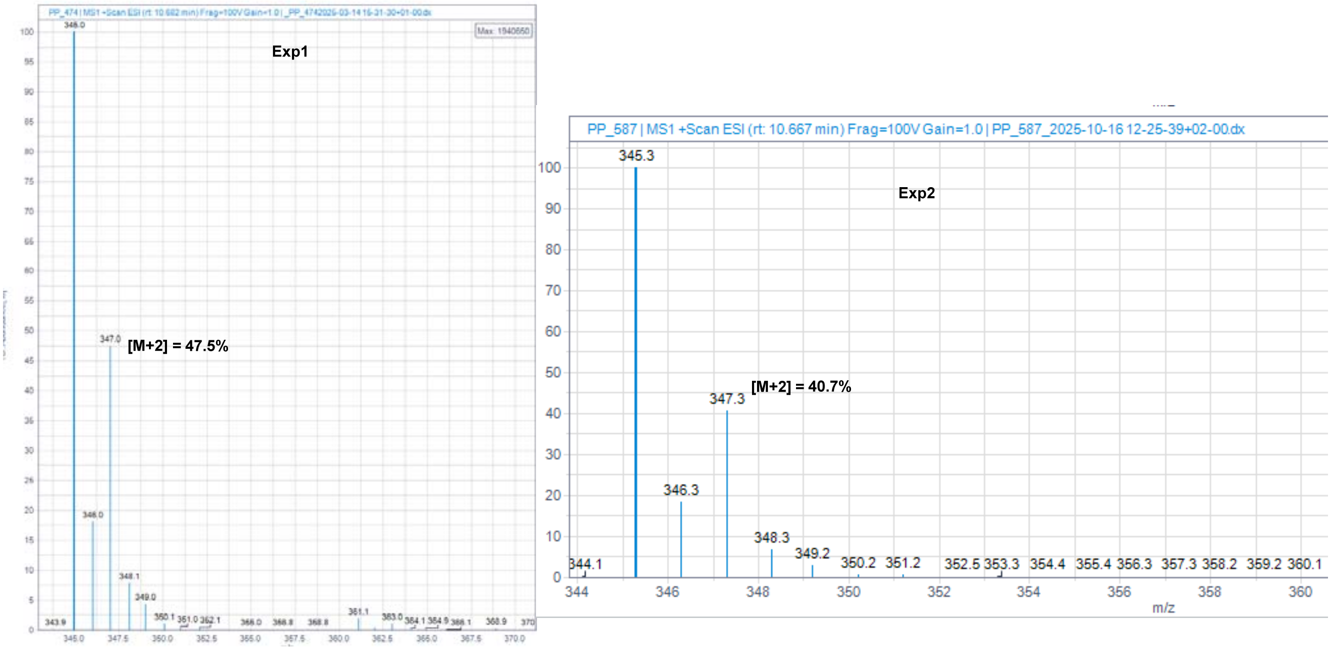

Figure S14. *ESI-MS of products **4a** obtained in the catalytic reactions with isolated rac-**5a**, performed in the presence of* $H_2{}^{18}O$ *(zoomed on in the region of* $M + Na^+$*)*

Overall, these experiments confirmed that water is not the oxygen source for the hydroxyl group of products **4**, safely excluding pathways such as the ring-opening of the episulfoniun ion derived from **5a** by a water molecule. Thus, the dimethylsulfoxide group of the ylide **1** was left as the only source of oxygen for the hydroxyl group of products **4**. Experiments with labeled ^{18}O-DMSO were planned, to check if exogenous DMSO could deliver its oxygen resulting in labelled products **4**. ^{18}O-DMSO was prepared by modifying a literature procedure[14] (Scheme S35). The main modifications are related to the second step of the synthesis, where the sulfonium salt, once added, tends to clump, impeding the stirring of the mixture. If this happens, it is better to remove the round bottom flask from the ice bath, and stir it manually, until the mixture appears stirrable and more salt can be added. Moreover, it was found to be more convenient to isolate the thus obtained ^{18}O-DMSO by chromatography, eluting with a EtOAc/MeOH 95:5 mixture. With

[14] N. Mupparapu, S. Khan, S. Battula, M. Kushwaha, A. P. Gupta, Q. N. Ahmed, R. A. Vishwakarma, *Org. Lett.*, **2014**, *16*, 1152–1155.

these modifications, ^{18}O-DMSO was obtained in 72% yield (703 mg; 639 µl) and with an 88% of ^{18}O incorporation, measured by GC-MS.

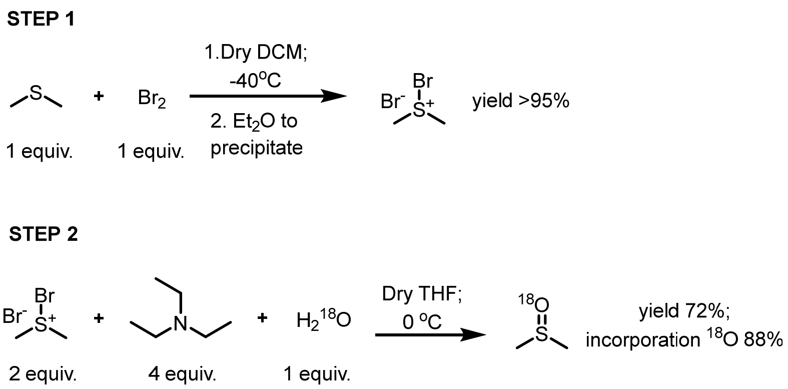

Scheme S35*. Preparation of labelled ^{18}O-DMSO.*

The first three experiments were run with the **DPP** catalyst and increasing amounts (from 1 to 5 equivalents) of ^{18}O-DMSO (Scheme S36). Incorporation of ^{18}O in the product **4a** was observed (Figure S15), with a trend reflecting the amount of ^{18}O-DMSO added to the system, reaching a 34% incorporation value when 5 equiv. were used.

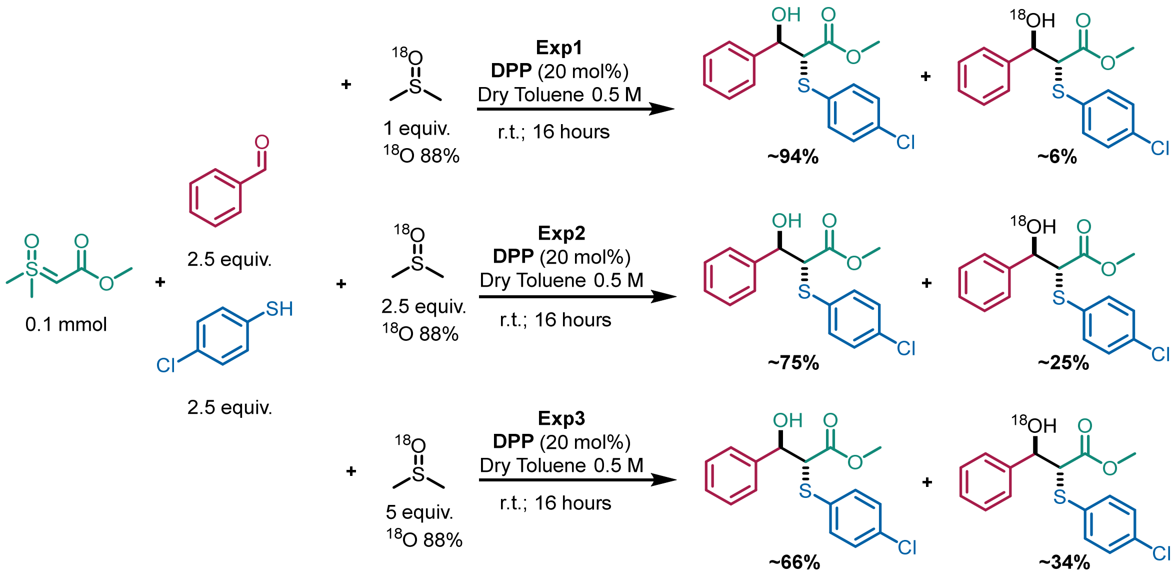

Scheme S36*. MCR catalysed by* ***DPP*** *in the presence of ^{18}O-DMSO.*

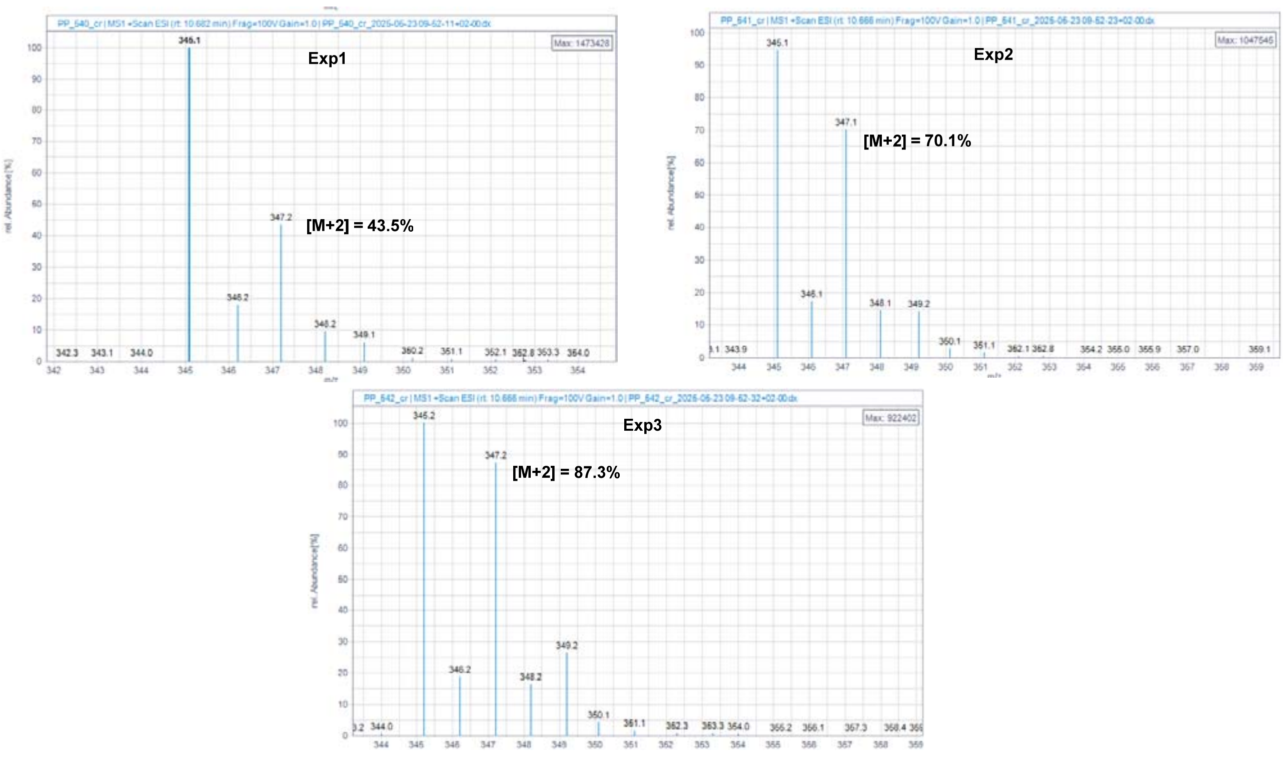

Figure S15. *ESI-MS of products **4a** obtained in the experiments with ^{18}O-DMSO and **DPP** catalyst (zoomed on in the region of M + Na^{+}).*

Another experiment was set up using 2.5 equivalents of ^{18}O-DMSO under the optimized conditions of the enantioselective reaction, using **CPA-5** as catalyst (Scheme S37). The obtained results (Figure S16) were consistent with the ones observed with **DPP** as catalyst. The slightly lower incorporation of the enantioselective reaction is likely the result of the less favored involvement of exogenous DMSO in a reaction at a lower temperature.

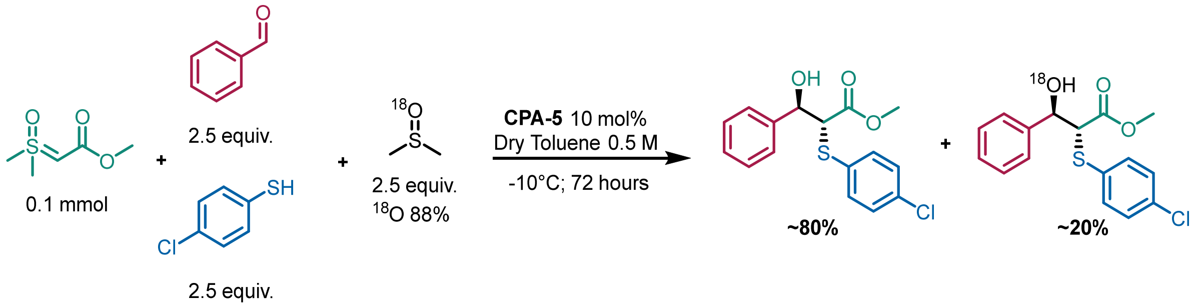

Scheme S37. *Experiment with **CPA-5** catalyst and ^{18}O-DMSO.*

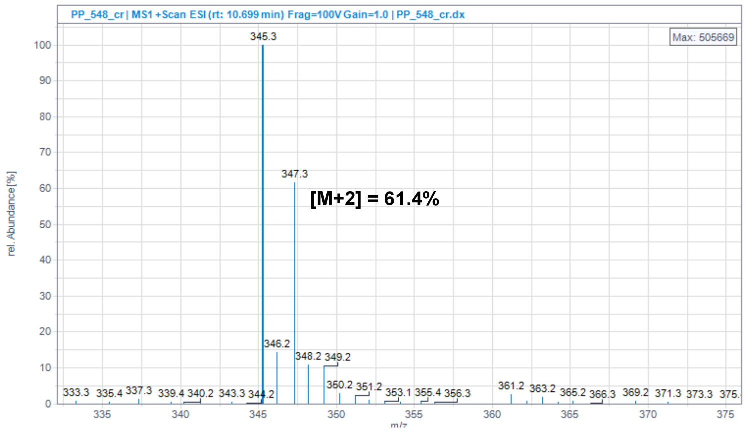

Figure S16. *ESI-MS of product **4a** obtained in the experiment with ^{18}O-DMSO and **CPA-5** catalyst (zoomed on in the region of M + Na^{+}).*

It was also verified that products **4a** previously synthetized (racemic and enantioenriched), put in contact with ^{18}O-DMSO in the presence of the catalysts (Scheme S38), did not incorporate ^{18}O in their structure (Figure S17).

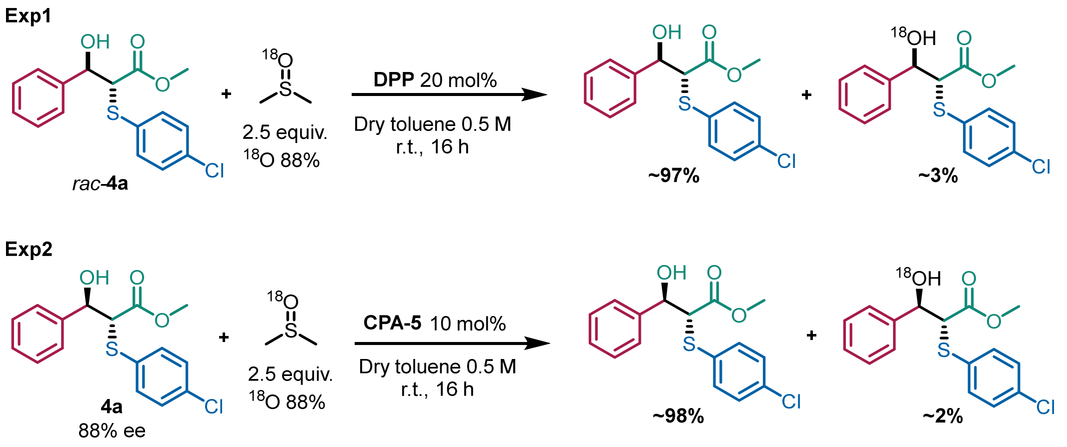

***Scheme S38**. Control experiments with products **4a** and ^{18}O-DMSO.*

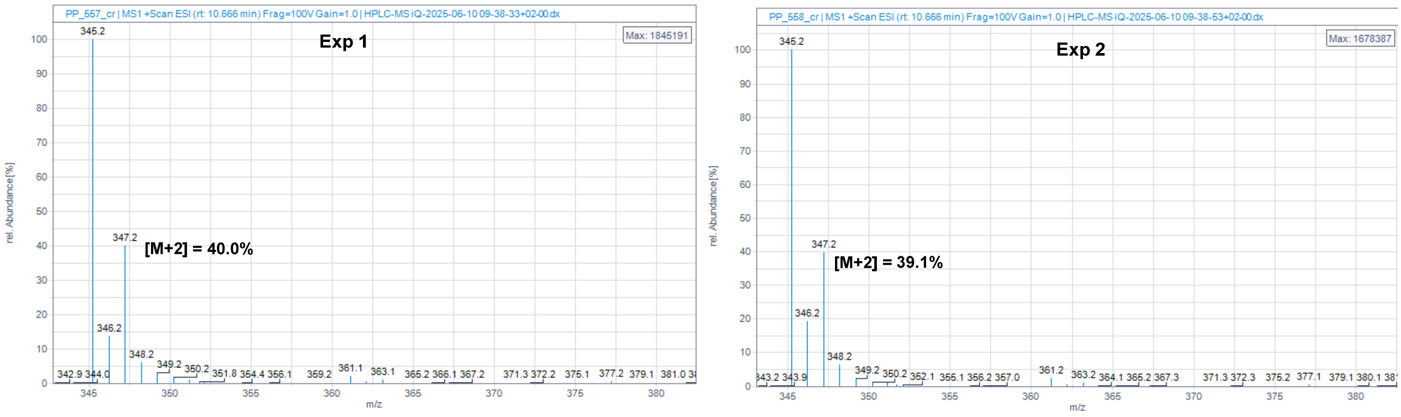

***Figure S17**. ESI-MS of products **4a** after the control experiments (zoomed on in the region of M + Na$^+$).*

These experiments provided evidence that DMSO can take part in the reaction mechanism releasing its oxygen into product **4** but gave only indirect proof that the origin of the hydroxyl oxygen of products **4** is the dimethylsulfoxonium oxygen of the ylide **1**. To obtain direct evidence, we embarked us in the synthesis of labelled ylide ^{18}O-**1a** (Scheme S39). Its synthesis consists of two steps starting from ^{18}O-DMSO. ^{18}O-labelled trimethylsulfoxonium iodide was prepared by slightly modifying a reported procedure for the unlabeled salt[15]. Modifications entailed collection of the salt from the reaction mixture by decantation, instead of filtration, and skipping the recrystallization step. The crude salt was obtained in 42% yield and used for the preparation of ^{18}O-**1a** following general procedure A (See page S57). The ^{18}O enrichment was determined on the isolated compound **1a** by comparing its ESI-MS spectrum with the spectrum of the corresponding unlabeled **1a**. The calculated 86% ^{18}O enrichment is consistent with the enrichment of the starting ^{18}O-DMSO used in this synthesis.

[15] E. T. Ledingham, C. J. Merritt, C. J. Sumby, M. K. Taylor, B. W. Greatrex, *Synthesis* **2017**, *49*, 2652-2662.

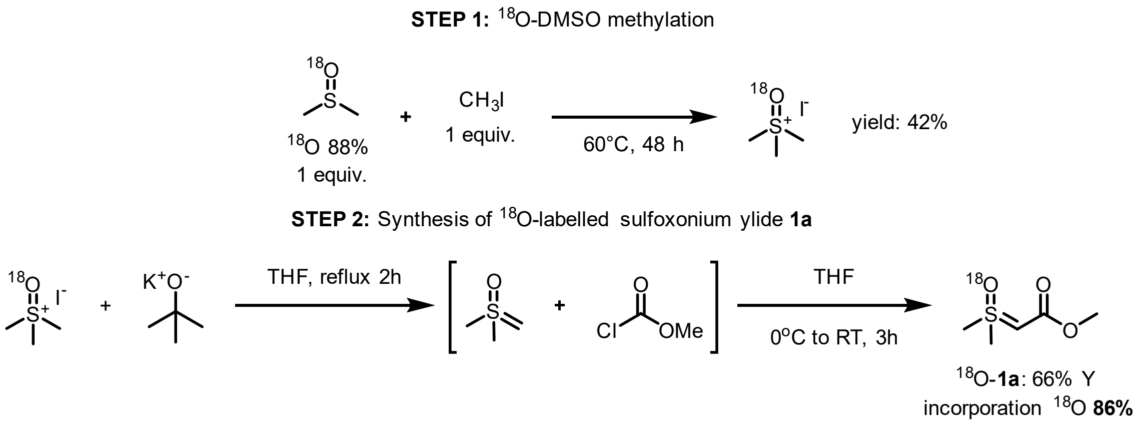

***Scheme S39**. Synthesis of ^{18}O-**1a**.*

We could thus carry out the **CPA-5** catalyzed asymmetric MCR using ^{18}O-**1a** as the ylide component (Scheme S40). ESI-MS analysis of product **4a** obtained from this reaction showed an 82.5% ^{18}O enrichment (**Figure S18**). Considering that the starting ylide **1a** presented an 86% ^{18}O enrichment, this translates to a nearly quantitative (96%) incorporation of the ^{18}O of ylide **1a** into product **4a**, unequivocably proving the origin of the benzylic oxygen of product **4a**.

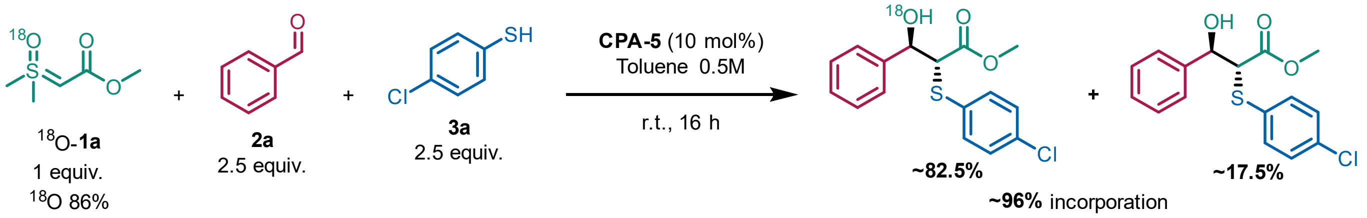

***Scheme S40**. Catalytic MCR with ^{18}O-**1a**.*

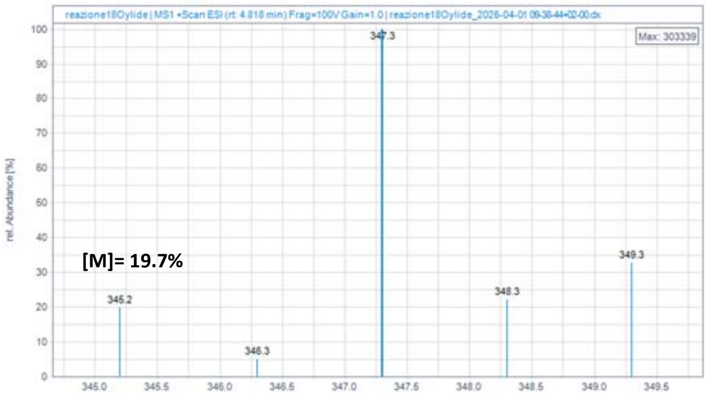

***Figure S18**. ESI-MS of product **4a** obtained in the catalytic reaction with ^{18}O-**1a** (zoomed on in the region of M + Na$^+$). In the calculation of the enrichment, (19.7*0.366)% was subtracted from the [M+2] peak.*

Having at hand the labelled ylide ^{18}O-**1a** allowed to inspect further the DMSO "journey" from ylide **1** to product **4**. Specifically, we wondered whether, after displacement, DMSO could leave its parent episulfonium-catalyst ion pair (**I**) and react with another episulfonium-catalyst intermediate (**I**), as suggested by the isolation of the "derailment" byproducts **12** and **13** under some conditions (see proposed catalytic cycle in Scheme S17). We thus designed a cross-over experiment with ylides ^{18}O-**1a** and **1d**, which carry a methyl and an allyl ester, respectively. If DMSO scrambling occurred, the reaction should also produce a labelled allyl product ^{18}O-**4w**. However, such cross-over experiment would be reliable only if labelled and unlabelled ylides react with comparable kinetics during the rearrangement step. This was verified by setting up a catalytic reaction with both **1a** and **1d** (Scheme S41) and following its evolution by sampling the mixture at regular times and analyzing it with ^{1}H NMR. As shown in Figure S19, ^{1}H NMR analysis after 1 h showed a broad, slightly doubled signal at ca. 5.65 ppm, which was assigned to the reaction intermediates **5**. Formation of the products **4a** and **4w** had not started yet. After 4 h, the formation of the products **4a** and **4w** appeared from their characteristic signals at 5.00 (d, J = 8.2 Hz) and 5.02 (d, J = 8.1 Hz) ppm, along with the decrease of the broad signal of

intermediates **5**. Thus, although **4a** formed somewhat faster than **4w**, the two rearrangements evolved in parallel, indicating the consistency of the planned cross-over experiment.

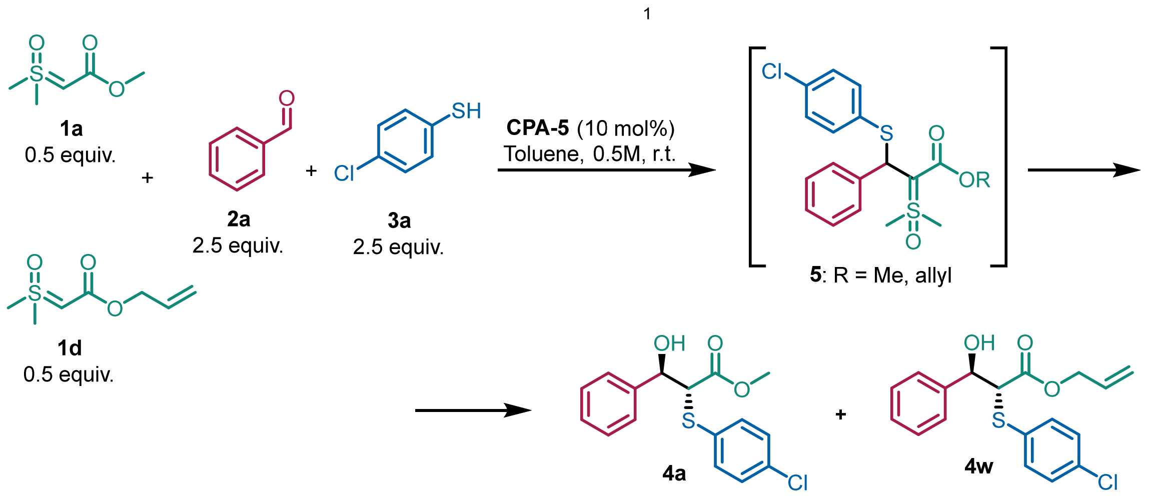

***Scheme S41**. Qualitative assessment of the relative kinetics of the rearrangement steps with ylides **1a** and **1d**.*

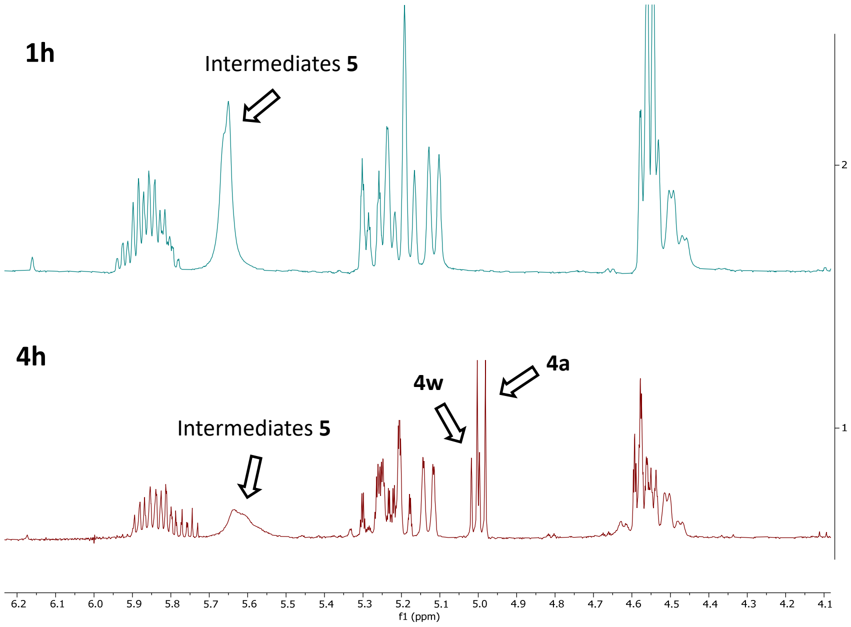

***Figure S19**. ^{1}H NMR spectra of the samples from the reaction with ylides **1a** and **1d** at different times.*

The experiment with labelled ylide ^{18}O-**1a** and unlabelled ylide **1d** was thus set up (Scheme S42). HPLC-MS analysis of the reaction crude showed the formation of the two products **4a** and **4w** (Figure S20a). Inspection of the ESI-MS spectrum of the band relative to **4a** indicated an 83% ^{18}O enrichment for ^{18}O-**4a** (Figure S20b), fully in line with the reaction that employed exclusively ^{18}O-**1a**. Conversely, comparison of the ESI-MS spectra of compound **4w** (Figure S20d) with the one of the HPLC band of **4w** obtained in the cross-over reaction (Figure S20c), showed unambiguously that ^{18}O incorporation in **4w** did not occur in this experiment.

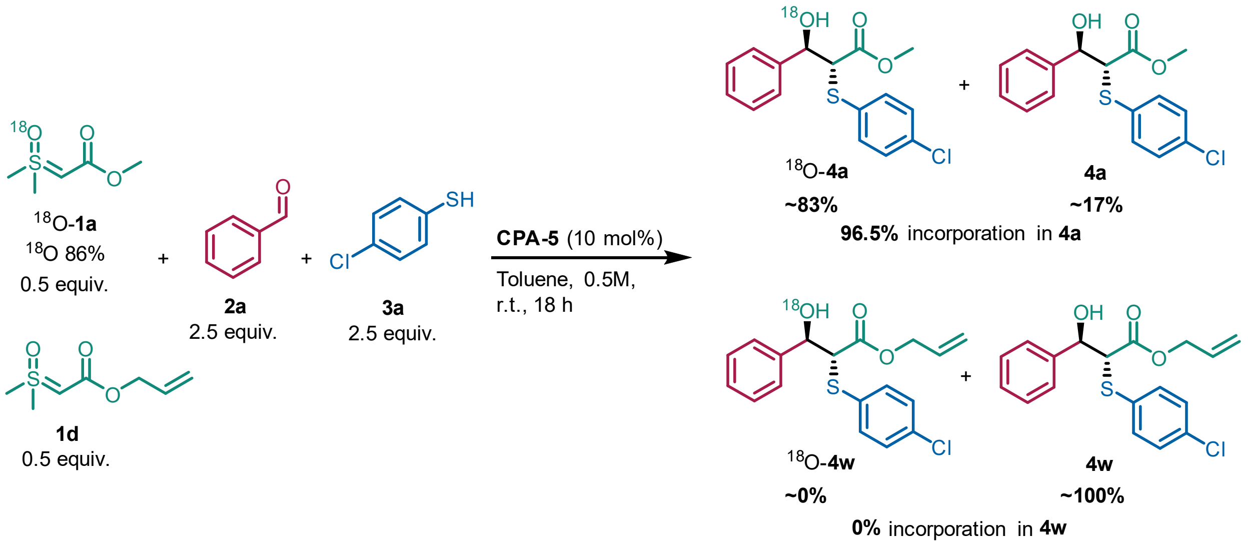

***Scheme S42**. Cross-over experiment with labelled ylide ^{18}O-**1a** and un-labelled ylide **1d**.*

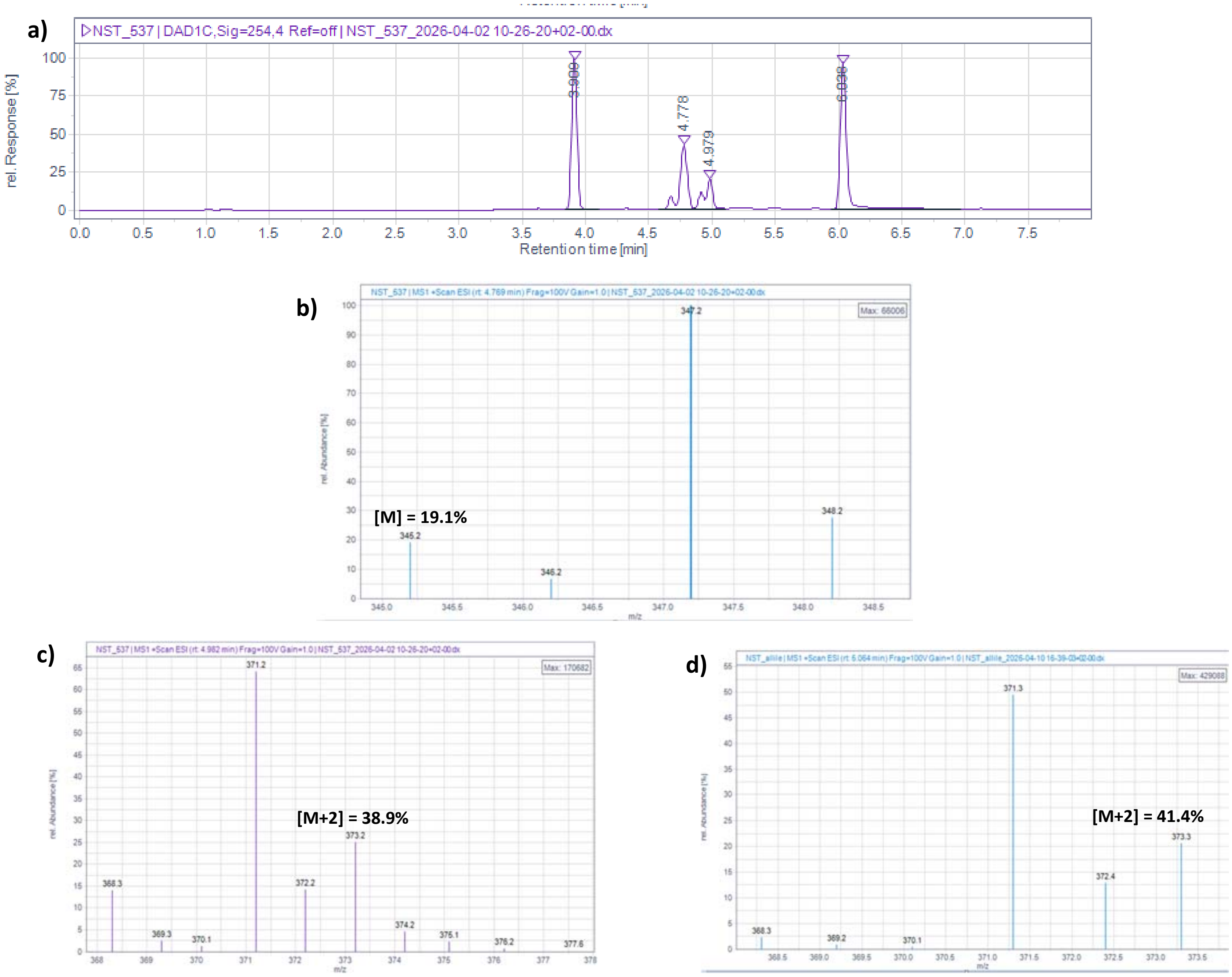

***Figure S20.** a) HPLC chromatogram of the crude mixture of the cross-over experiment. b) ESI-MS of the band at t = 4.77 min of the chromatogram, relative to compound **4a** obtained in the cross-over experiment. In the calculation of the enrichment, (19.7*0.366)% was subtracted from the [M+2] peak. c) ESI-MS of the band at 4.89 min of the chromatogram, relative to compound **4w** obtained in the cross-over experiment. d) ESI-MS of compound **4w** obtained in the standard reaction. ESI-MS spectra are zoomed on in the region of M + Na^{+}. In c) and d), the percentage of the [M+ 2] peak was adjusted as [M + 2]/[M]%, to take into account that the [M] peak is not the base peak in these spectra.*

It is thus clear from the experiments reported in this section that the displaced DMSO molecule acts as an O-nucleophile in the catalytic mechanism. The role of DMSO as O-nucleophile is known in the literature.[16] Besides the venerable Kornblum oxidation - where the base promoted elimination of DMS from O-alkyl adducts of DMSO delivers carbonyl compounds–examples resulting in hydroxyl groups have been reported.[17,18,19] However, the migration of DMSO into the same molecule–as part of a formal rearrangement–appears a peculiarity of this reaction. In fact, the preference of the episulfonium ion intermediate to suffer attack by the DMSO oxygen *vs* the other nucleophiles present in the reaction mixture–especially thiophenols–seems somewhat surprising. In this context, it is worth noting that only a moderate level of ^{18}O incorporation in **4a** was observed even by using a large excess of exogenous ^{18}O-DMSO. Since the exogenous ^{18}O-DMSO competes with the "endogenous" DMSO, which is released during the catalyzed episulfonium ion formation, and is thus present only in sub-stoichiometric amounts, each equivalent of exogeneous ^{18}O-DMSO used in the experiments above translates to a 5-10-fold excess. Considering also the complete lack of DMSO scrambling observed in the cross-over experiment, we can conclude that the ring-closure to the episulfonium, and its ring opening by "endogenous" DMSO are intimately linked processes and are not independent events. This may ultimately justify the dominance of DMSO over exogenous nucleophiles in the episulfonium ion ring opening, and prompts to define the process as a formal rearrangement reaction.

Reaction kinetics and Hammett's plots by in situ ^1H NMR

To get an insight into the reaction kinetics – that is, on the evolution of the different species, including intermediates **5**, with time - the reaction course was followed by *in-situ* ^1H NMR, which was also used to study the influence of electronic effects on the reaction. Hammett's plots were built changing the substituents on either benzaldehyde **2** or thiophenol **3** ring. For running these experiments, deuterated chloroform ($CDCl_3$) was chosen as solvent instead of toluene-d_8 because of the better solubility of ylide **1a** and catalyst **CPA-5**. For the same reason, the dilution of the reaction was increased (0.2 M instead of 0.5 M). The catalytic reaction presents a similar behavior in $CDCl_3$ and toluene. For example, a reaction between **1a**, **2a** and **3a**, catalyzed by **CPA-5** and performed in $CDCl_3$ at -10 °C, gives product **4a** as a single *anti*-diastereoisomer with full conversion and 88% ee, validating the feasibility of this approach.

General procedure for in-situ ^1H NMR monitoring

In a 5 mm NMR tube, ylide **1a** (0.1 mmol; 1 equiv.), the catalyst **CPA-5** (10.0 mg, 0.01 mmol; 0.1 equiv.) and 1,3,5-trimethoxybenzene (7.0 mg, 0.033 mmol; 0.33 equiv; internal standard) were dissolved in $CDCl_3$ (0.5 mL; 0.2 M). Then, aldehyde **2** (0.25 mmol; 2.5 equiv.) and thiophenol **3** (0.25 mmol; 2.5 equiv.) were added in this order (Scheme S43). The reaction was monitored *via* ^1H NMR for a minimum of 12 hours, recording a ^1H NMR spectrum every 3.5 or 6 minutes.

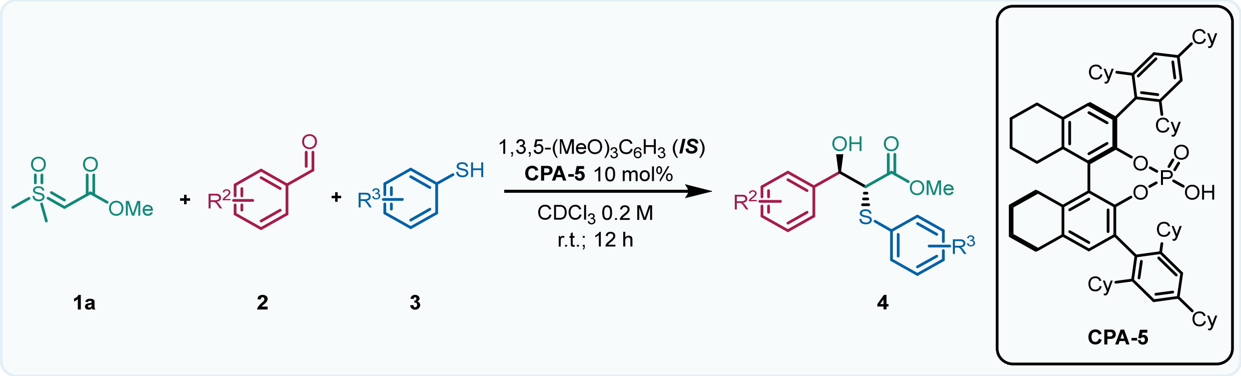

Scheme S43. *General reaction scheme for in-situ ^1H NMR monitoring.*

[16] H. Lu, Z. Tong, L. Peng, Z. Wang, S.-F. Yin, N. Kambe, R. Qiu, *Top. Curr. Chem.* **2022**, *380*, 55.
[17] H. Liu, D. Ji, Y. Mei, Y. Liu, C. Liu, X. Wang, Q. Chen, *Nat. Chem.* **2024**, *16*, 1505–1514.
[18] Y. Ashikari, T. Nokami, J.-i. Yoshida. *Org. Lett.* **2012**, *14*, 938–941.
[19] S. Song, X. Huang, Y.-F. Liang, C. Tang, X. Lia, N. Jiao, *Green Chem.* **2015**, *17*, 2727–2731.

In-situ ¹H NMR monitoring of the model reaction

The model reaction of ylide **1a** with benzaldehyde **2a** and *para*-chlorothiophenol **3a** was monitored using this approach (Scheme S44), focusing on the formation of product **4a** but also on the evolution of the intermediate **5** with time. Remarkably, dimethylsulfide (DMS) was detected too, with its characteristic singlet at 2.1 ppm.

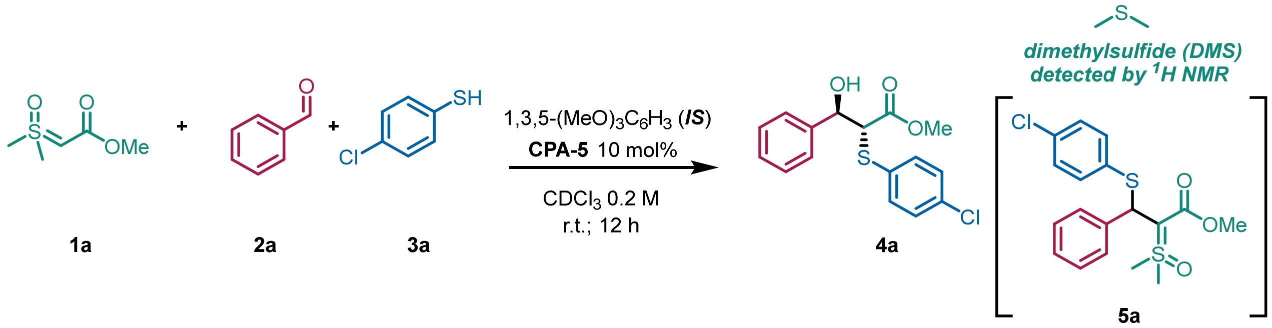

***Scheme S44**. Model reaction and species monitored by in situ ¹H NMR.*

A concentration (mM) / time (min) graph of these species was built based on their normalized integral values (Figure S21). Intermediate ylide **5a** started appearing at t = 0, as expected, and then reached a maximum concentration after about 150 min from the first recorded spectrum. Interestingly, the formation of product **4a** starts only at that time, that is, only when all **5a** has formed. In fact, the final concentration of **4a** (ca. 150 mM) is comparable to the maximum concentration of **5a**, observed after 150 min when **4a** starts forming. It can be speculated that the strong interactions observed by ¹H NMR between ylide **1a** and a phosphoric acid like **DPP** (see section: "Hypothesis 2: X-H insertion reactions followed by enol catalysis") makes the **CPA-5** catalyst unavailable for the rearrangement until ylide **1a** is present in the mixture. For some reasons, the phosphoric acid catalyst might have more affinity for ylide **1a** than for its disubstituted counterpart **5a**. The appearance of product **4a** started after 150 min, and was concurrent with the decrease of **5a**, as expected. Remarkably, this behavior was observed even when studying thiophenols and benzaldehydes whose electronics were leading to increased reaction rates in the rearrangement step rather than the first ($k_2 > k_1$; a striking example is *p*-methoxythiophenol where $k_2 = 3.0$ and $k_1 = 0.9$ and even for this case no product formation could be noticed before reaching $[\mathbf{5}]_{max}$). We were also able to detect dimethylsulfide (DMS, 2.1 ppm, s) and track its formation which parallels the appearance of product **4a**, with an imperfect overlap due to the volatility of DMS. This behavior is in line with the proposed mechanism, which involves release of DMS (see section: "Proposal overview").

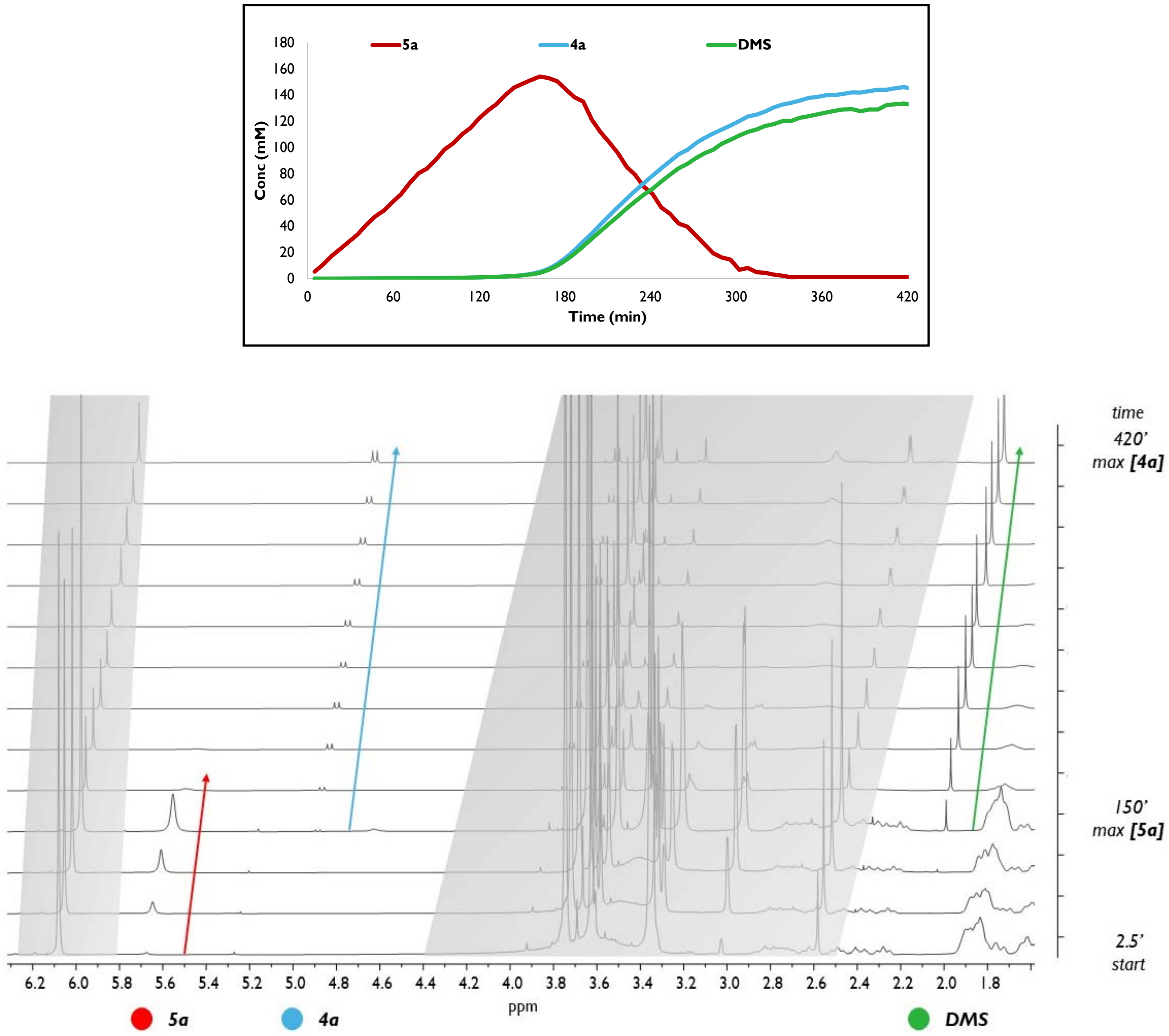

Figure S21. *Concentration vs time plot for **5a**, **4a**, and DMS, elaborated from the in situ* ^{1}*H NMR experiments, and stacked NMR spectra. For better visualization, the intensity of **5a** integrals was amplified and grey areas were added where tracking of starting materials, intermediates or products was not feasible.*

Hammett's plots accounting for benzaldehyde and thiophenol electronics.

The viability of the reaction monitoring by *in-situ* NMR made it possible to study the effect of substitution around the benzaldehyde **2** and thiophenol **3** benzene ring by building Hammett's plots, selecting substrates from the reaction scope (Scheme S45). Knowing the reaction to be constituted by two sequential but non-overlapping steps (first formation of intermediate ylide **5** and then formation of product **4**), the initial rate of formation of intermediate ylide **5** as rate constant for the first step ($k_{1\ obs}$) and its initial rate of disappearance as rate constant for the second step ($k_{2\ obs}$) were used.

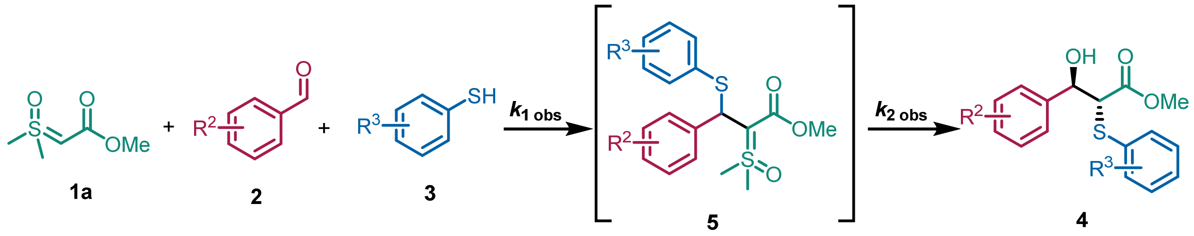

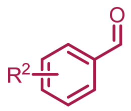

2

R_2	σ_p	σ_m
p-*t*Bu	-0.20	-
p-Me	-0.17	-
m-Me	-	-0.07
p-H	0.00	-
p-Cl	0.23	-
p-Br	0.23	-
m-Cl	-	0.37
p-CO_2Me	0.45	-
p-CF_3	0.54	-

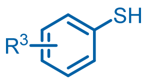

3

R_3	σ_p	σ_m
p-MeO	-0.27	-
p-Me	-0.17	-
p-F	0.06	-
p-Cl	0.23	-
p-H	0.00	-
m-F	-	0.34
m-CF_3	-	0.43
p-CF_3	0.54	-
p-CN	0.66	-

Scheme S45*. Substrates* ***2*** *and* ***3*** *used for the Hammett's plots, and the σ-constants of their substituents.*

Benzaldehyde substitution: First step (formation of intermediate 5)

For the first step of the reaction concerning the formation of intermediate **5**, different substituents on the benzaldehyde aromatic ring affect $k_{1\ obs}$ strongly, with values ranging from 0.71 mM/min to 4.57 mM / min (Scheme S46). A Hammett plot was built using these data and a clear linear relation was found, with a positive ρ value. Thus, decrease of electron density on the ring of the benzaldehydes **2** leads to an increase in the reaction rate of the first step of the reaction. This could be interpreted considering the higher reactivity of thionium ions resulting from electron-poor benzaldehydes.

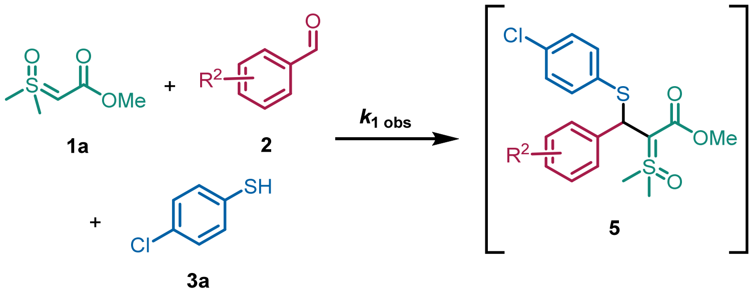

R_2	σ_p	σ_m
p-tBu	-0.20	-
p-Me	-0.17	-
m-Me	-	-0.07
p-H	0.00	-
p-Cl	0.23	-
p-Br	0.23	-
m-Cl	-	0.37
p-CO_2Me	0.45	-
p-CF_3	0.54	-

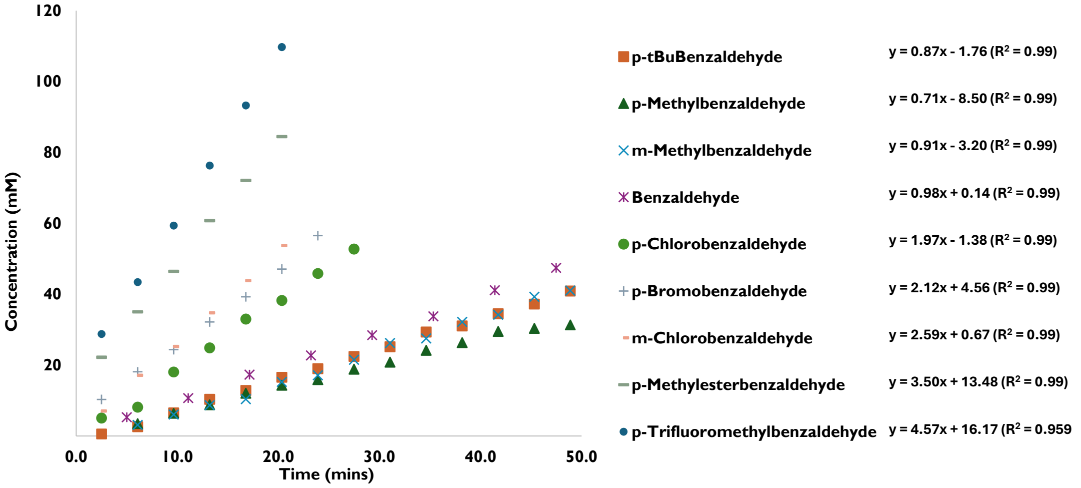

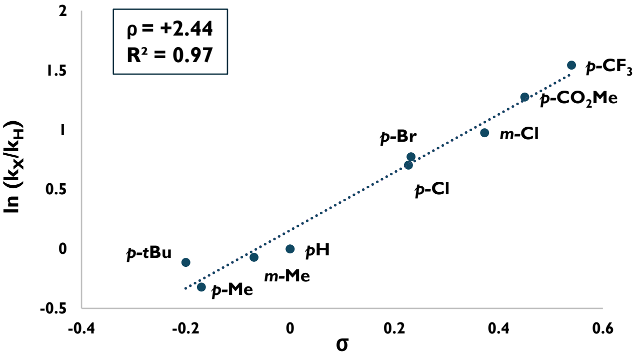

3

***Scheme S46**. Concentration of intermediates **5** vs time in the reactions with different aldehydes **2**, by in-situ ^{1}H NMR (first step), and resulting Hammett plot.*

Benzaldehyde substitution: Second step (formation of product 4)

For the second step of the reaction concerning the formation of product **4** with different benzaldehydes, only mild and strong electron withdrawing substituents affect the reaction rate significantly (from -0.23 mM/min to -0.82 mM/min) (Scheme S47). Electron rich substituents did not affect significantly the initial rate of disappearance. A Hammett plot was built using these data and a strong linear relation was found for mild and strong electron withdrawing substituents - from *para*-chloro to *para*-CF_3 substituents - accompanied by a non-linear zone for electron neutral and electron-rich ones. The strongly negative ρ is consistent with the hypothesis of episulfonium ion formation as the rate determining step. The reaction rate is affected by the electronic nature of the substituent on the benzaldehyde ring. Decreasing the electron density on the ring, lowers the ring closure rate likely because the system develops a positive charge, mainly localized on the episulfonium ion sulfur atom.

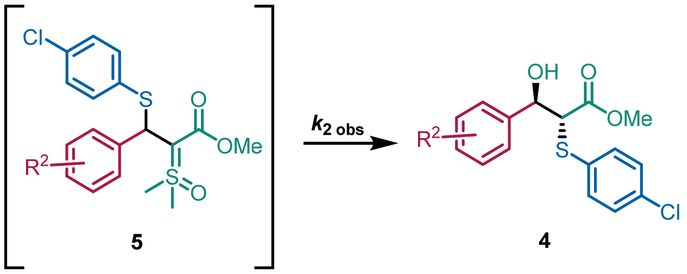

R_2	σ_p	σ_m
*p-t*Bu	*-0.20*	-
*p-*Me	*-0.17*	-
*m-*Me	-	*-0.07*
*p-*H	*0.00*	-
*p-*Cl	*0.23*	-
*p-*Br	*0.23*	-
*m-*Cl	-	*0.37*
p-CO_2Me	*0.45*	-
p-CF_3	*0.54*	-

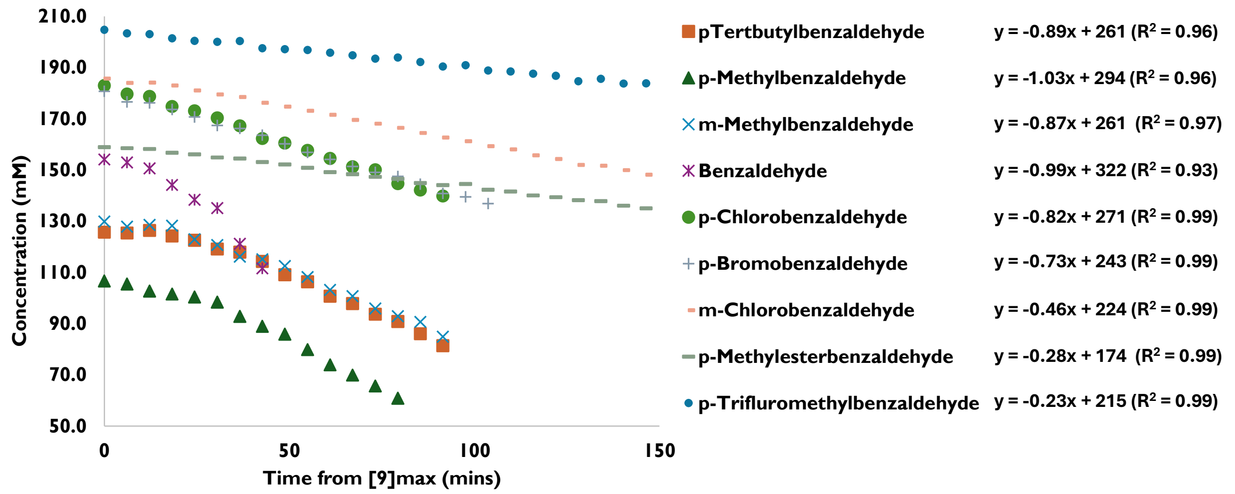

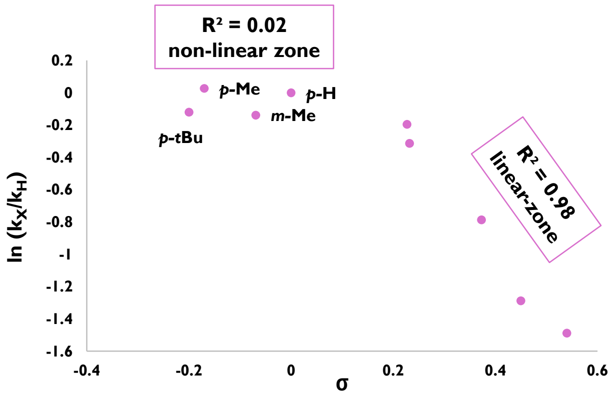

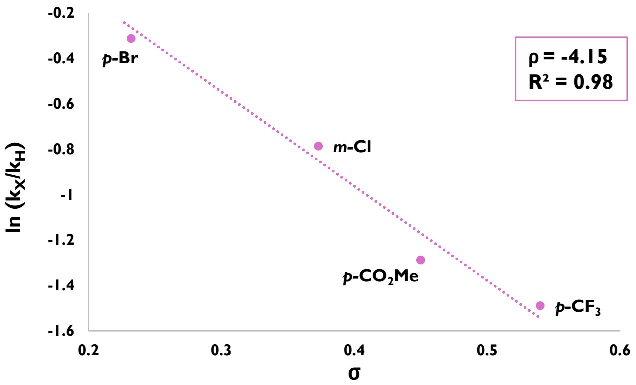

Scheme S47. *Concentration of intermediates* **5** *vs time in the reactions with different aldehydes* **2**, *by in-situ* [1]*H NMR (second step), and resulting Hammett's plot.*

Thiophenol substitution: First step (formation of intermediate 5)

For the first step of the reaction concerning the formation of intermediate **5**, different substituents on the thiophenol aromatic ring affect the reaction rate only to a minor extent, with values ranging from 0.78 mM/min to 1.31 mM/min (Scheme S48). A Hammett plot was built from these data, and no evident linear relation was found. A possible explanation for this behavior is that its electron poor, benzylic carbon dominates the reactivity of the thionium ion.

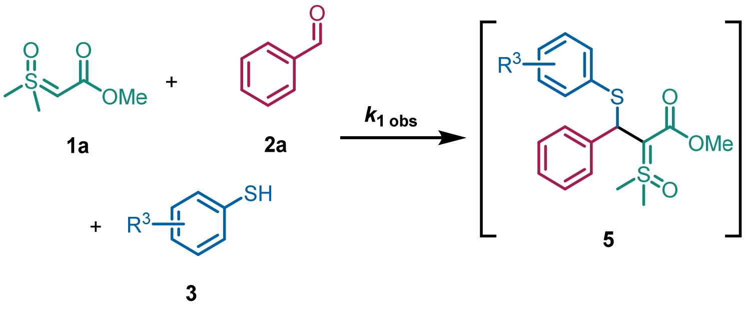

R_3	σ_p	σ_m
p-MeO	*-0.27*	-
p-Me	*-0.17*	-
p-F	*0.06*	-
p-Cl	*0.23*	-
p-H	*0.00*	-
m-F	-	*0.34*
m-CF_3	-	*0.43*
p-CF_3	*0.54*	-
p-CN	*0.66*	-

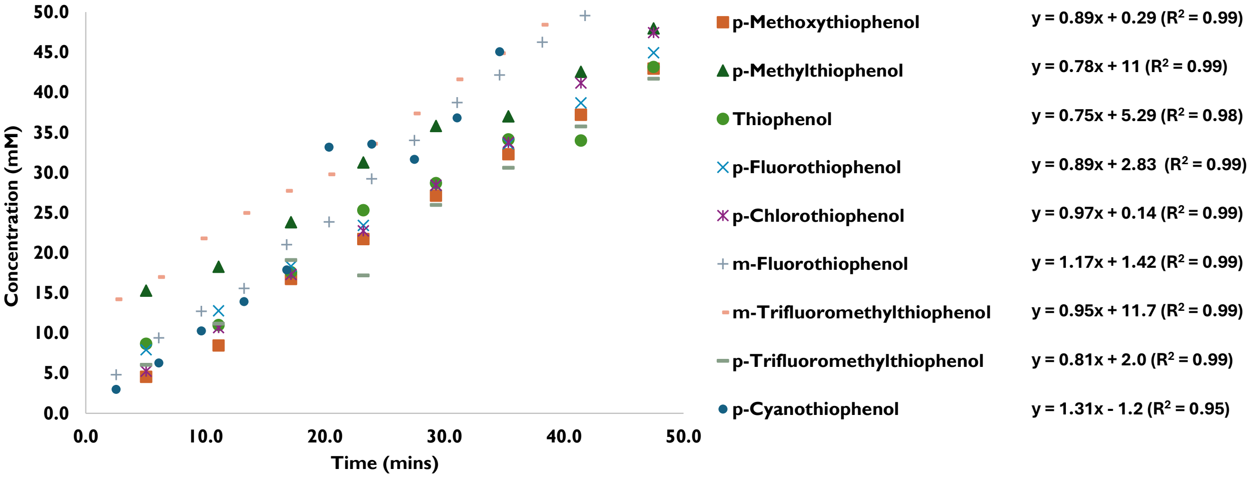

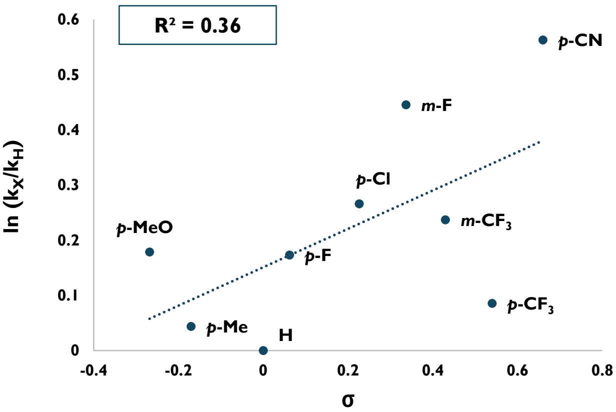

***Scheme S48**. Concentration of intermediates **5** vs time in the reactions with different thiophenols **3**, by in-situ ^{1}H NMR (first step), and resulting Hammett plot.*

Thiophenol substitution: Second step (formation of product 4)

For the second step of the reaction concerning the formation of product **4**, the substitution on the thiophenol aromatic ring affects dramatically the reaction rate (from -0.12 mM/min to -3.0 mM/min, which translates into a 25-fold change of $\boldsymbol{k}_{2\ obs}$) (Scheme S49). A Hammett plot was built using these data and a linear relation was found. As for the case of benzaldehydes substitution, the strongly negative ρ is consistent with the hypothesis of episulfonium ion formation as the rate determining step of the process. A higher electron density on the sulfur atom likely facilitates the ring closure to form the episulfonium ion.

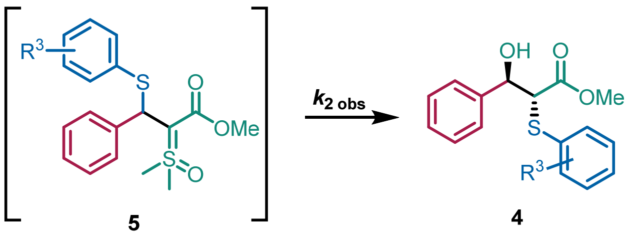

R_3	σ_p	σ_m
p-MeO	*-0.27*	-
p-Me	*-0.17*	-
p-F	*0.06*	-
p-Cl	*0.23*	-
p-H	*0.00*	-
m-F	-	*0.34*
m-CF_3	-	*0.43*
p-CF_3	*0.54*	-
p-CN	*0.66*	-

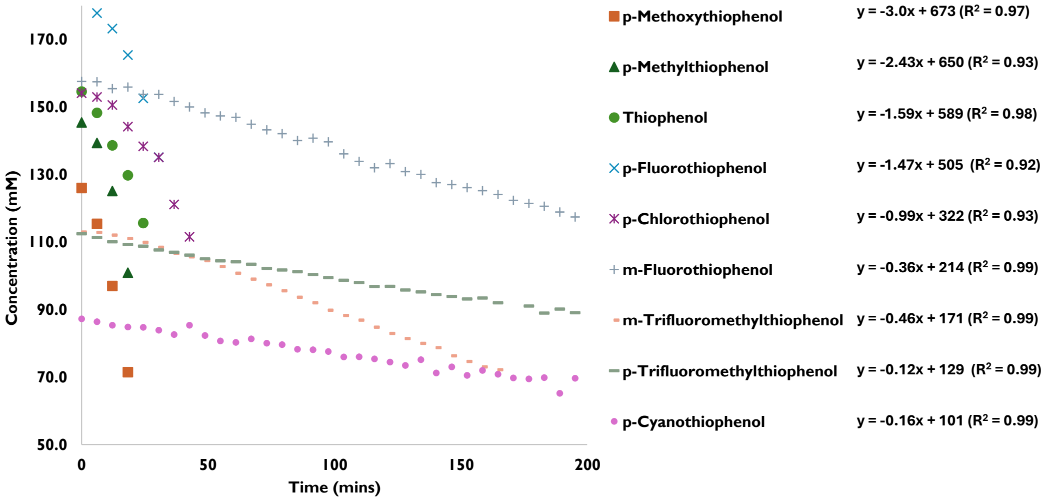

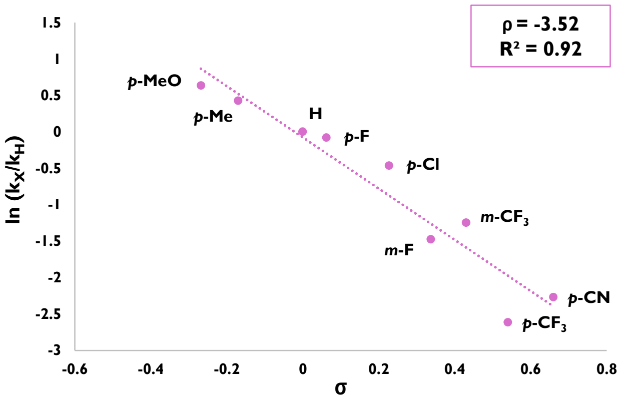

Scheme S49. *Concentration of intermediates* ***5*** *vs time in the reactions with different thiophenols* ***3****, by in-situ* ^{1}*H NMR (second step), and resulting Hammett's plot.*

Limitations of the methodology

***Table S7**: Exploration of the catalytic MCR with sulfoxonium ylides **1f-k** stabilized by ketone and amide groups.*

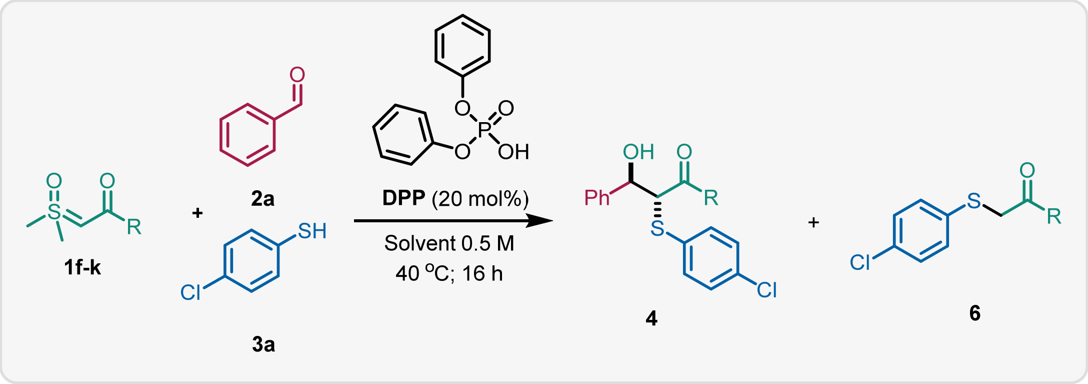

Entry[a]	***1**: R*	*Solvent*	***4**-Yield*[b] *(%)*	***6**-Yield*[b] *(%)*
1	**1f**: R = NHPh	Toluene	<5	>95
2	**1f**: R = NHPh	CH_3CN	<5	84
3	**1g**: R = NEt_2	Toluene	<5	71
4[c]	**1g**: R = NEt_2	CH_3CN	<5	73
5	**1h**: R = NPh_2	Toluene	<5	78
6	**1i**: R = Me	Toluene	<5	<5
7	**1i**: R = Me	CH_3CN	<5	<5
8	**1j**: R = *t*-Bu	Toluene	<5	60
9	**1k**: R = 4-$MeOC_6H_4$	Toluene	42	32
10[c,d]	**1k**: R = 4-$MeOC_6H_4$	Toluene	<5	75

[a] Conditions: 0.2 mmol **1**, 0.45 mmol **2a**, 0.45 mmol **3a**, 0.04 mmol **DPP** catalyst, 0.4 mL solvent, 40 °C, 16 h. [b] Determined by ^{1}H-NMR using CH_2Br_2 as internal standard. [c] RT. [d] **CPA-1** [(*R*)-TRIP, 0.02 mmol] catalyst.

As shown in Table S7, using the **DPP** catalyst only ylide **1k** carrying an aromatic ketone led to the formation of the MC product **4**, along with its S-H insertion adduct **6** (entry 9). However, when the CPA catalyst **CPA-1** was used, the MC product **4** was not obtained (entry 10). NMR signals compatible with ylide intermediates **5** were not observed in any of the reactions reported in Table S7.

The reactivity of aliphatic aldehydes in the MCR was explored using isobutyraldehyde **2u** and hexanal **2v** (Scheme S50). In the crude mixtures of reactions with **DPP** catalyst, the catalyst-trapped adducts **12b** and **12c** could be tentatively identified. These species formed in a yield comparable to the catalyst loading (ca. 20%). That is, the episulfonium intermediates instead of undergoing attack by DMSO leading to **4an** and **4ao** were trapped by the catalyst. Adding 5 equivalents of exogeneous DMSO in a reaction with **2u** did not change the reaction outcome. Moreover, a reaction with **CPA-1** [(*R*)-TRIP] gave an intractable mixture of products, with no identifiable species.

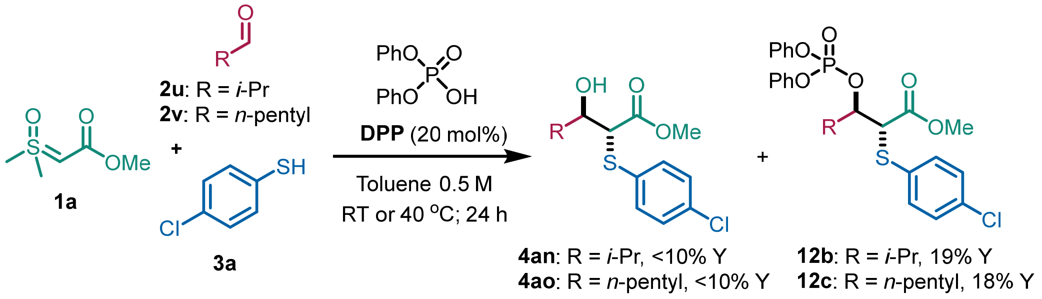

***Scheme S50**. Representative results of the catalytic MCR with aliphatic aldehydes.*

1-Pentanethiol **3n** was used as a representative aliphatic thiol. Both reactions with **DPP** and **CPA-1** [(*R*)-TRIP] afforded the desired MC product **4ap** as single diastereoisomer, but only in low yield (Scheme S51). Moreover, the CPA did not induce any enantioselectivity in the reaction.

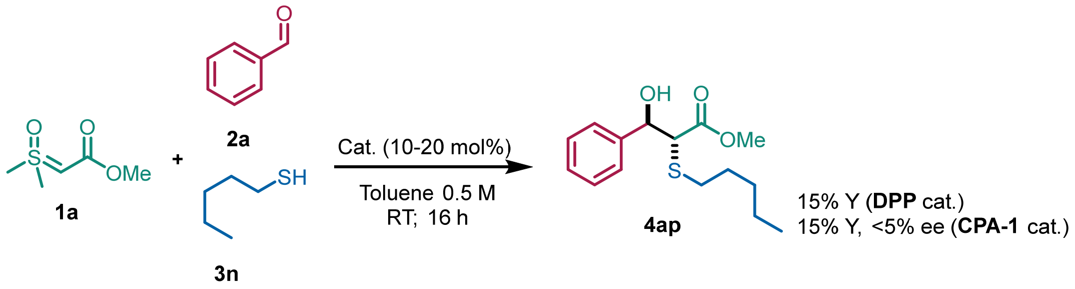

Scheme S51*. Representative results of the catalytic MCR with an aliphatic thiol.*

Summarizing these attempts to expand the substrate scope of the MCRs, an ylide carrying an aromatic ketone (**1k**), and an aliphatic thiol (**3n**), provided promising results in terms of reactivity, but the representative **CPA-1** [(*R*)-TRIP] could not provide solid results. Conversely, amide-derived ylides gave exclusively the S-H insertion products **6**. The **DPP** catalyst could combine the three reaction components in reactions with aliphatic aldehydes **2u** and **2v**, but this catalyst was trapped by the episulfonium intermediate and did not afford the β-hydroxy-α-sulfanyl products **4**.

Experimental details

General procedures

*General procedure **A**: synthesis of ylides **1a-e**[20]*

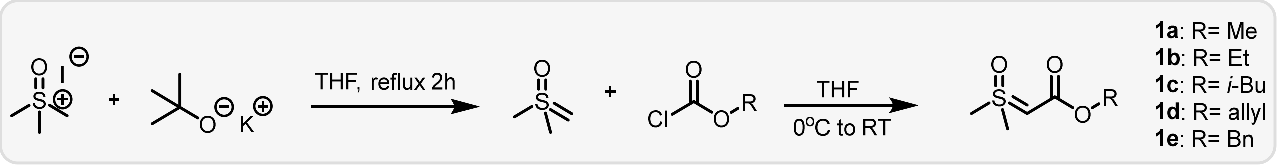

Under N_2, trimethylsulfoxonium iodide (6.6 g, 30 mmol, 3.0 equiv.) was suspended in dry THF (60 mL) in a flame-dried round bottom flask that was protected from light with aluminum foil. Potassium *tert*-butoxide (4.49 g, 40 mmol, 4.0 equiv.) was added and the mixture was stirred at reflux for 2 hours. After cooling to 0 °C, the corresponding chloroformate (1.0 equiv.) in THF (25 mL) was added dropwise to the mixture via a dropping funnel. After stirring at room temperature for 1 h, the mixture was filtered through a plug of Celite®, and the plug washed with CH_2Cl_2. After evaporation of all volatiles, purification by flash chromatography on silica gel (100% EtOAc) afforded sulfoxonium ylides **1a-e**.

*General procedure **B**: catalytic enantioselective synthesis of compounds **4** via MCR at -10 °C*

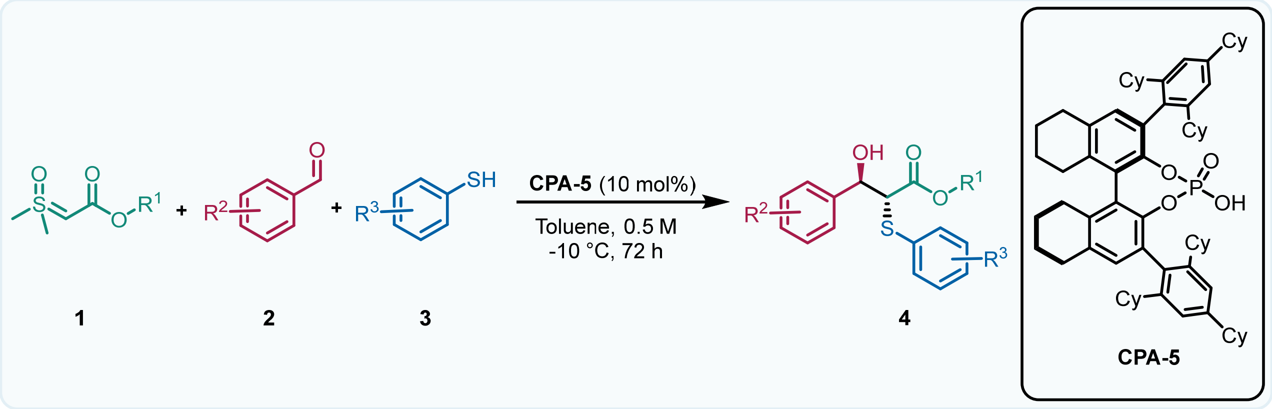

In a vial equipped with a magnetic stirring bar, ylide **1** (0.1 mmol; 1 equiv.) and the catalyst **CPA-5** (10.0 mg, 0.01 mmol; 0.1 equiv.) were suspended in toluene (0.2 mL; 0.5 M). The mixture was placed in a bath previously cooled to -10 °C. After a few minutes, aldehyde **2** (0.25 mmol; 2.5 equiv.) and thiophenol **3** (0.25 mmol; 2.5 equiv.) were added in this order. The resulting mixture was stirred for 72 h at the same temperature. Next, the solvent was evaporated under vacuum and the residue analyzed by ^{1}H NMR to determine the diastereomeric ratio. Flash chromatography on silica gel (*n*-hexane/EtOAc mixtures) afforded compounds **4**.

[20] Procedure adapted from: C. Janot, P. Palamini, B. C. Dobson, J. Muir, C. Aïssa, *Org. Lett.* **2019**, *21*, 296–299.

*General procedure **C**: catalytic enantioselective synthesis of compounds **4** via MCR at RT*

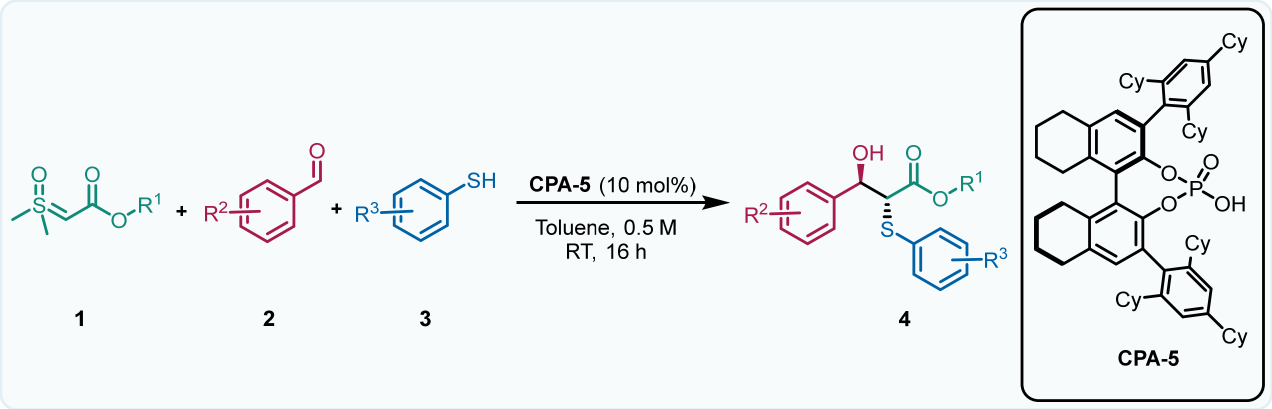

In a vial equipped with a magnetic stirring bar, ylide **1** (0.1 mmol; 1 equiv.) and the catalyst **CPA-5** (10.0 mg, 0.01 mmol; 0.1 equiv.) were suspended in toluene (0.2 mL; 0.5 M). Then, aldehyde **2** (0.25 mmol; 2.5 equiv.) and thiophenol **3** (0.25 mmol; 2.5 equiv.) were added in this order. The resulting mixture was stirred for 16 h at RT. Next, the solvent was evaporated under vacuum and the residue analyzed by ^{1}H NMR to determine the diastereomeric ratio. Flash chromatography on silica gel (*n*-hexane/EtOAc mixtures) afforded compounds **4**.

*General procedure **D**: catalytic enantioselective synthesis of compounds **4** via MCR at 60 °C*

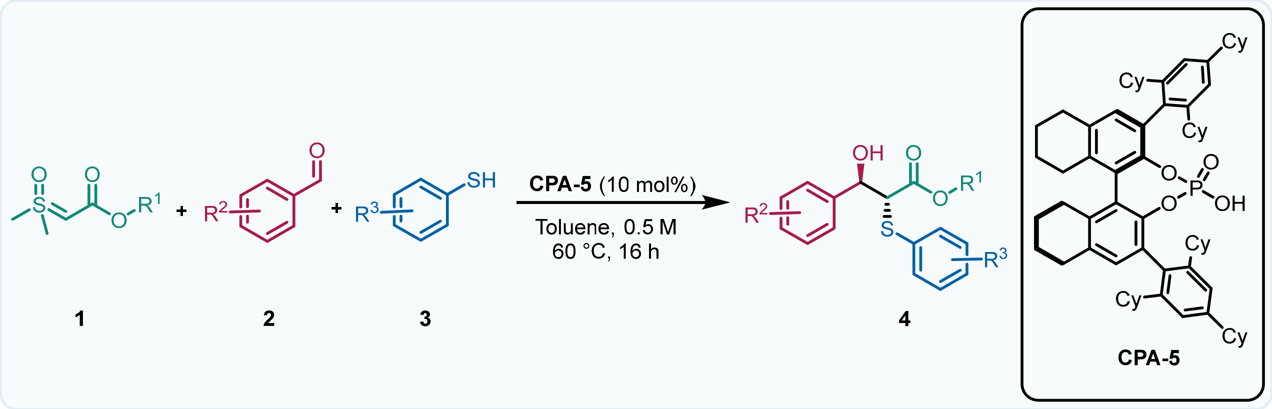

In a vial equipped with a magnetic stirring bar, ylide **1** (0.1 mmol; 1 equiv.) and the catalyst **CPA-5** (10.0 mg, 0.01 mmol; 0.1 equiv.) were suspended in toluene (0.2 mL; 0.5 M). Then, aldehyde **2** (0.25 mmol; 2.5 equiv.) and thiophenol **3** (0.25 mmol; 2.5 equiv.) were added in this order, and the vial immediately placed in a pre-heated (60 °C) bath. The resulting mixture was stirred for 16 h at this temperature. Next, the solvent was evaporated under vacuum and the residue analyzed by ^{1}H NMR to determine the diastereomeric ratio. Flash chromatography on silica gel (*n*-hexane/EtOAc mixtures) afforded compounds **4**.

*General procedure **E**: synthesis of racemic reference compounds **4***

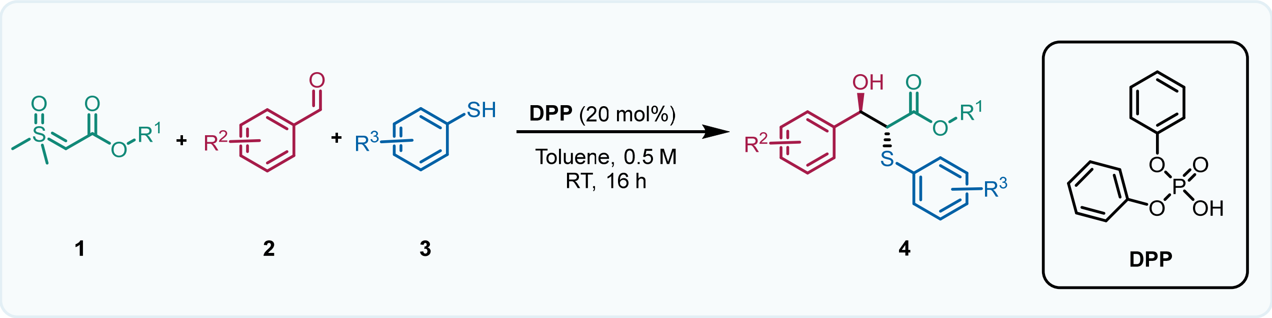

In a vial equipped with a magnetic stirring bar, ylide **1** (0.1 mmol; 1 equiv.) and the catalyst **DPP** (5.0 mg, 0.02 mmol, 0.2 equiv.) were suspended in toluene (0.2 mL; 0.5 M). Then, aldehyde **2** (0.25 mmol, 2.5 equiv.) and thiophenol **3** (0.25 mmol, 2.5 equiv.) were added in this order. The resulting mixture was stirred for 16 h at RT. Next, the solvent was evaporated under vacuum, and the residue analyzed by ^{1}H NMR. Flash chromatography on silica gel (*n*-hexane/EtOAc mixtures) afforded racemic compounds **4**.

Synthesis of catalyst **CPA-5** [(*R*)-H_8-TCYP]

The synthesis of catalyst **CPA-5** [(*R*)-H_8-TCYP] was adapted from different literature sources. **CPA-5** from commercial sources, or obtained with the synthesis outlined here, was used for the catalytic reactions.

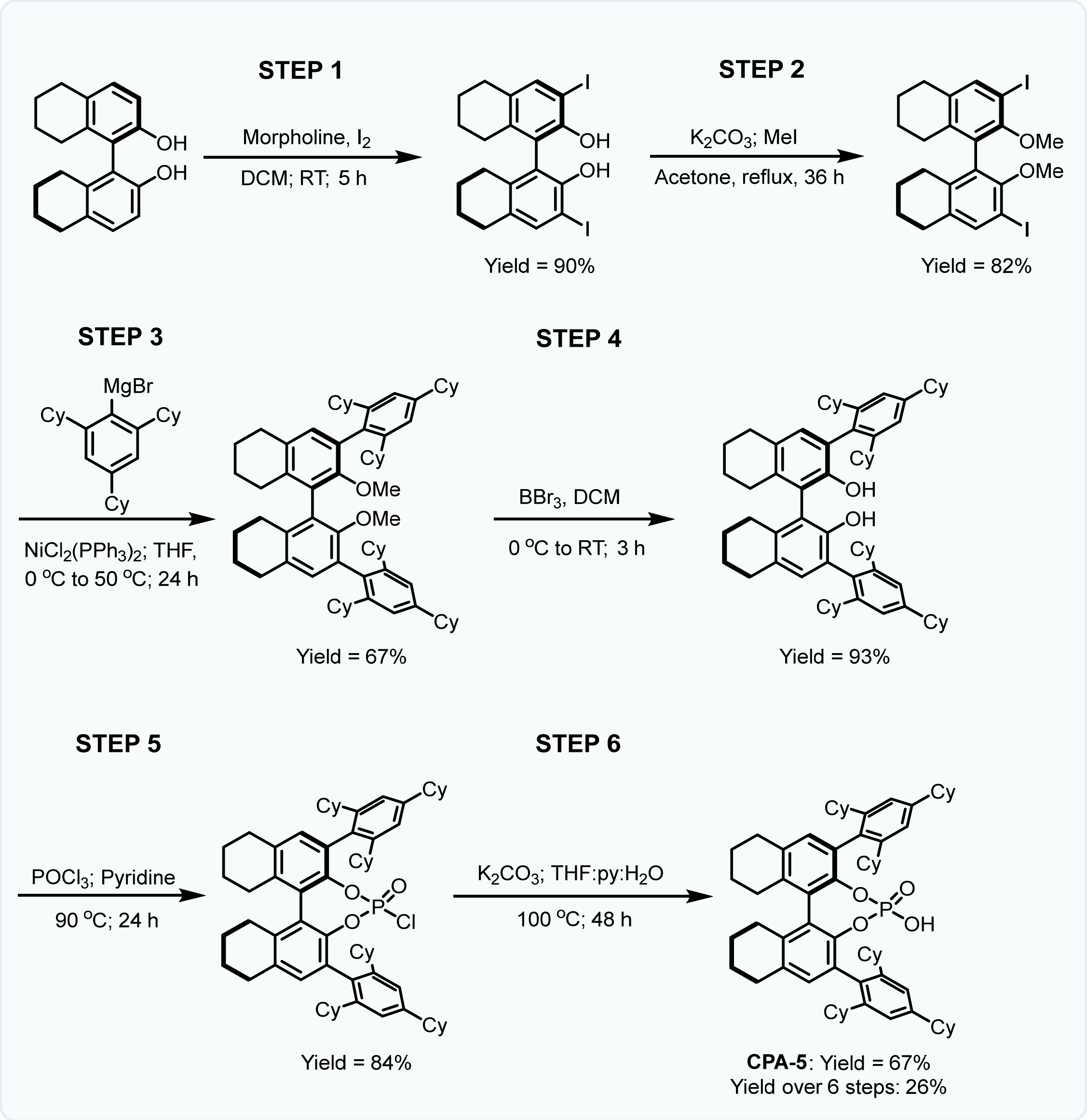

Step 1: **Synthesis of (*R*)-3,3'-diiodo-5,5',6,6',7,7',8,8'-octahydro-[1,1'-binaphthalene]-2,2'-diol**[21]

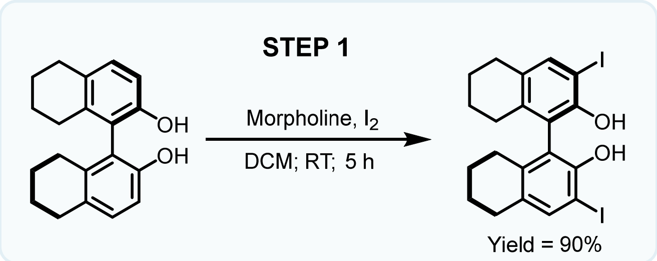

To a solution of (*R*)-H_8-BINOL (5.0 g, 16.98 mmol, 1.0 equiv.) in DCM (120 mL, 0.14 M) were added successively, at RT, morpholine (8.8 mL, 101.88 mmol, 6.0 equiv.) and I_2 (8.6 g, 33.96 mmol, 2.0 equiv.). The mixture was stirred at this temperature for 5 h and turned progressively red. Then, aq. HCl (12 M, 10 mL) was added, and the phases separated. The aqueous layer was extracted with DCM and the combined organic phases were washed successively with a saturated aq. $Na_2S_2O_3$ solution (3 × 100 mL) and brine, then dried over $MgSO_4$, filtered and concentrated under reduced

[21] G. Pousse, A. Devineau, V. Dalla, L. Humphreys, M.-C. Lasne, J. Rouden, J. Blanchet, *Tetrahedron* **2009**, *65*, 10617–10622.

pressure. The resulting residue was purified by flash chromatography (*n*-hexane/EtOAc = 9:1) to afford the title compound as white solid (8.35 g, 15.3 mmol, 90% yield). Spectral data match the ones reported in the literature.

Step 2: **Synthesis of (*R*)-3,3'-diiodo-2,2'-dimethoxy-5,5',6,6',7,7',8,8'-octahydro-1,1'-binaphthalene**[22]

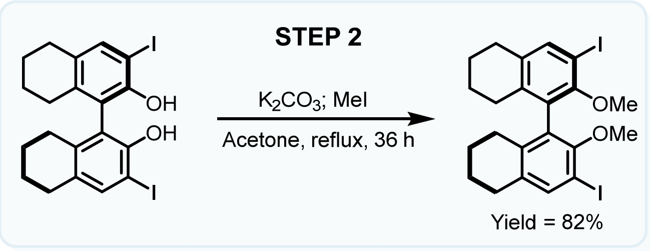

K_2CO_3 (7.48 g, 3.4 equiv.) and MeI (5.95 mL, 96 mmol, 6 equiv.) were successively added to a refluxing solution of (*R*)-3,3′-diiodo-5,5′,6,6′,7,7′,8,8′- octahydro-[1,1′-binaphthalene]-2,2′-diol (8.7 g, 16 mmol, 1 equiv.) in acetone (32 mL, 0.5 M). The mixture was stirred for 24 h at reflux, then additional MeI (2.93 mL, 48 mmol, 3 equiv.) was added, and the mixture was stirred at reflux for another 12 h. The mixture was then allowed to cool to RT and H_2O (20 mL) was added. The resulting suspension was stirred for 4 h at RT then filtered. The resulting white solid was washed with H_2O and dried in an oven at 65 °C to give the title compound as a white solid (7.53 g, 13.12 mmol, 82% yield). Spectral data match the ones reported in the literature.

Step 3: **Synthesis of (*R*)-2,2'-dimethoxy-3,3'-bis(2,4,6-tricyclohexylphenyl)-5,5',6,6',7,7',8,8'-octahydro-1,1'-binaphthalene**[23]

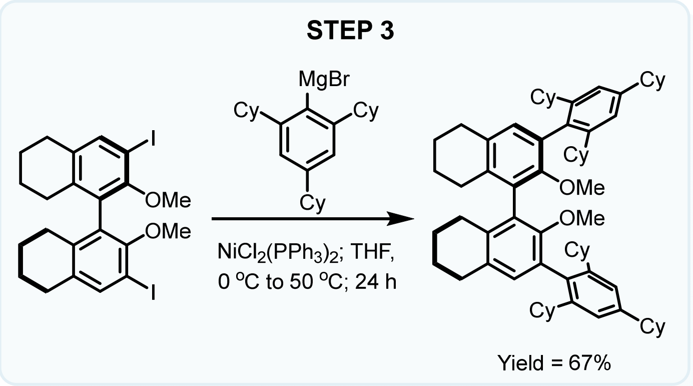

(2,4,6-Tricyclohexylphenyl)magnesium bromide solution[24]: Into a 250 mL round bottom flask, magnesium (559 mg, 23.0 mmol) was added. The flask was heated and cooled to RT under an atmosphere of argon and then suspended in dry THF (10 mL). To the mixture at RT was slowly added 1-bromo-2,4,6-tricyclohexylbenzene (8.87 g, 20.9 mmol) dissolved in dry THF (50 mL). After completion of the addition of bromide, the reaction mixture was heated under reflux for 6 h, upon which, a black/grey Grignard solution was formed. Kumada coupling[23]: To a stirred suspension of (*R*)-3,3'-diiodo-2,2'-dimethoxy-5,5',6,6',7,7',8,8'-octahydro-1,1'-binaphthalene (2.0 g, 3.48 mmol, 1 equiv.) and $NiCl_2(PPh_3)_2$ (0.228 g, 0.35 mmol, 0.1 equiv.) in anhydrous and degassed THF (17 mL, 0.2 M), was added the thus prepared Grignard solution (20.9 mmol, 6 equiv.) over 5 min at 0 °C. The resulting brown suspension was stirred for 24 h at 50 °C under N_2 atmosphere. During this time, the formation of large amount of solids was observed. The reaction was quenched by dropwise addition of cold saturated aq. NH_4Cl (30 mL) at 0 °C. After stirring for 15 min, the layers were separated, and the aqueous phase was extracted with EtOAc (3 x 50 mL). The combined organic phases were dried over Na_2SO_4, filtered and evaporated under reduced pressure. The crude mixture was purified by flash

[22] S. Brenet , C. Minozzi , B. Clarens , L. Amiri , F. Berthiol, *Synthesis,* **2015**, 47, 3859–3873.
[23] C. M. Avila, J. S. Patel, Y. Reddi, M. Saito, H. M. Nelson, H. P. Shunatona, M. S. Sigman, R. B. Sunoj, D. F. Toste, *Angew. Chem. Int. Ed.* **2017**, 56, 5806 –5811.
[24] V. Rauniyar, Z. J. Wang, H. E. Burks, F. D. Toste, *J. Am. Chem. Soc.* **2011**, *133*, 8486–8489.

chromatography (PE/EtOAc = 20:1) to afford the title compound as white foam (2.25 g, 2.33 mmol, 67% yield). The spectral data match with the ones reported in the literature.

Step 4: Synthesis of (*R*)-3,3'-bis(2,4,6-tricyclohexylphenyl)-5,5',6,6',7,7',8,8'-octahydro-[1,1'-binaphthalene]-2,2'-diol[23]

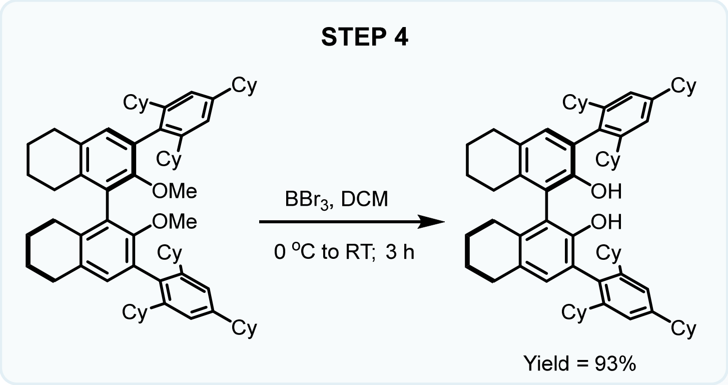

To a solution of (*R*)-2,2'-dimethoxy-3,3'-bis(2,4,6-tricyclohexylphenyl)-5,5',6,6',7,7',8,8'-octahydro-1,1'-binaphthalene (2.52 g, 2.6 mmol, 1 equiv.) in anhydrous DCM (51.5 mL; 0.5 M) and under N_2 atmosphere, was added BBr_3 (15.6 mL, 1 M solution in DCM, 15.6 mmol, 6 equiv.) at 0 °C. The orange solution was stirred at RT for 3 h after which NMR analysis of an aliquot indicated full conversion. The reaction was quenched by the addition of cold saturated aq. $NaHCO_3$ (20 mL) at 0 °C, and was stirred for 30 min at RT. The layers were separated, and the aqueous phase extracted with DCM (3 x 50 mL). The combined organic phases were dried over Na_2SO_4, filtered and concentrated under reduced pressure. The crude product was purified by flash chromatography (*n*-hexane/EtOAc = 98:2) to afford the title compound as white foam (2.36 mmol, 2.22 g, 93% yield). The spectral data match the ones reported in the literature.

Step 5: Synthesis of (*R*)-4-chloro-2,6-bis(2,4,6-tricyclohexylphenyl)-8,9,10,11,12,13,14,15-octahydrodinaphtho[2,1-*d*:1',2'-*f*][1,3,2]dioxaphosphepine 4-oxide[23]

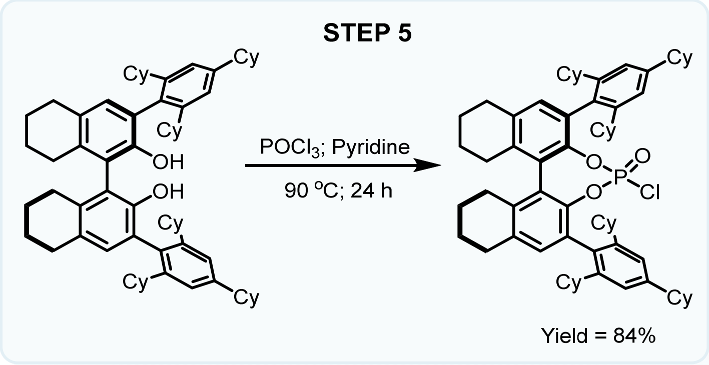

To a stirred solution of (*R*)-3,3'-bis(2,4,6-tricyclohexylphenyl)-5,5',6,6',7,7',8,8'-octahydro-[1,1'-binaphthalene]-2,2'-diol (2.36 g, 2.47 mmol, 1 equiv.) in anhydrous pyridine (12.4 mL, 0.2 M) was added phosphoryl chloride (0.691 mL, 7.41 mmol, 3 equiv.) dropwise over 3 min. The yellow solution was then stirred for 24 h at 90 °C. The reaction was allowed to cool to RT and DCM (50 mL) was added. The organic mixture was washed with water (3 x 50 mL), dried over Na_2SO_4, filtered and concentrated under reduced pressure. The residual pyridine was removed by co-evaporation with *n*-heptane. The crude white solid was purified by flash chromatography (*n*-hexane/EtOAc = 14:1) to afford the title compound as white solid (2.11 g, 2.07 mmol, 84% yield). The spectral data match the ones reported in the literature.

Step 6: Synthesis of (*R*)-4-hydroxy-2,6-bis(2,4,6-tricyclohexylphenyl)-8,9,10,11,12,13,14,15-octahydrodinaphtho[2,1-*d*:1',2'-*f*][1,3,2]dioxaphosphepine 4-oxide (CPA-5)[23]

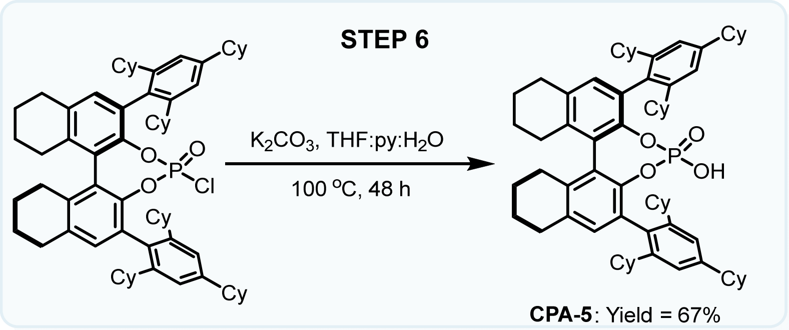

To a stirred solution of (*R*)-4-chloro-2,6-bis(2,4,6-tricyclohexylphenyl)-8,9,10,11,12,13,14,15-octahydrodinaphtho[2,1-*d*:1',2'-*f*][1,3,2]dioxaphosphepine 4-oxide (0.5 g, 0.49 mmol, 1 equiv.) in THF/pyridine/H_2O (2:1:1, 40 mL) was added K_2CO_3 (0.271 g, 1.96 mmol, 4 equiv.). The reaction mixture was stirred for 48 h at 90 °C. Aq. HCl (12 M, 10 mL) was added dropwise, and the reaction was allowed to cool to RT. The layers were separated, and the aqueous phase extracted with DCM (3 x 50 mL). The combined organic phases were washed once with aq. HCl (12 M, 100 mL), dried over Na_2SO_4, filtered and concentrated under reduced pressure. The crude solid was triturated 3 times with MeCN and filtered *in vacuo* to afford **CPA-5** as white solid (0.328 g, 0.33 mmol, 67% yield). The spectral data match the ones reported in the literature.

NB: in an attempt of purification by flash chromatography, **CPA-5** was found to be unstable on silica gel.

Synthesis and characterization of the catalytic products **4**

4a- Methyl (2*R*,3*R*)-2-((4-chlorophenyl)thio)-3-hydroxy-3-phenylpropanoate

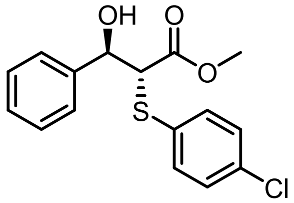

$C_{16}H_{15}ClO_3S$

MW: 322.8 g/mol

Yield: 77%;

Colorless oil

The product (**4a**) was prepared using the general procedure **B** from **1a** (methyl 2-(dimethyl(oxo)-λ^6-sulfaneylidene)acetate), **2a** (benzaldehyde), and **3a** (4-chlorobenzenethiol). The product was obtained as a colorless oil (25 mg; 77% yield). The diastereomeric ratio obtained for the *anti*-couple is greater than 20:1. The enantiomeric excess of the product was determined by CSP HPLC: AD-H column, *n*-hexane/iPrOH 80:20, 0.75 mL/min, t_{maj} = 12.1 min; t_{min} = 17.5 min, 88% ee.

^{1}H NMR (600 MHz, $CDCl_3$) δ 7.36 – 7.34 (m, 5H), 7.21 – 7.14 (m, 4H), 5.00 (d, *J* = 8.2 Hz, 1H), 3.87 (d, *J* = 8.2 Hz, 1H), 3.69 (s, 3H), 3.07 (br s, 1H).

^{13}C NMR (151 MHz, $CDCl_3$) δ 171.8, 139.9, 134.6, 134.6, 131.4, 129.2, 128.5, 128.5, 126.8, 74.5, 57.8, 52.5.

HRMS (MALDI$^+$) m/z calcd for $C_{16}H_{15}ClO_3SNa^+$[M+Na]$^+$: 345.0323, found: 345.0328.

$[\alpha]_D^{RT}$ = +42.5 (c = 1.0 g/100 mL, $CHCl_3$).

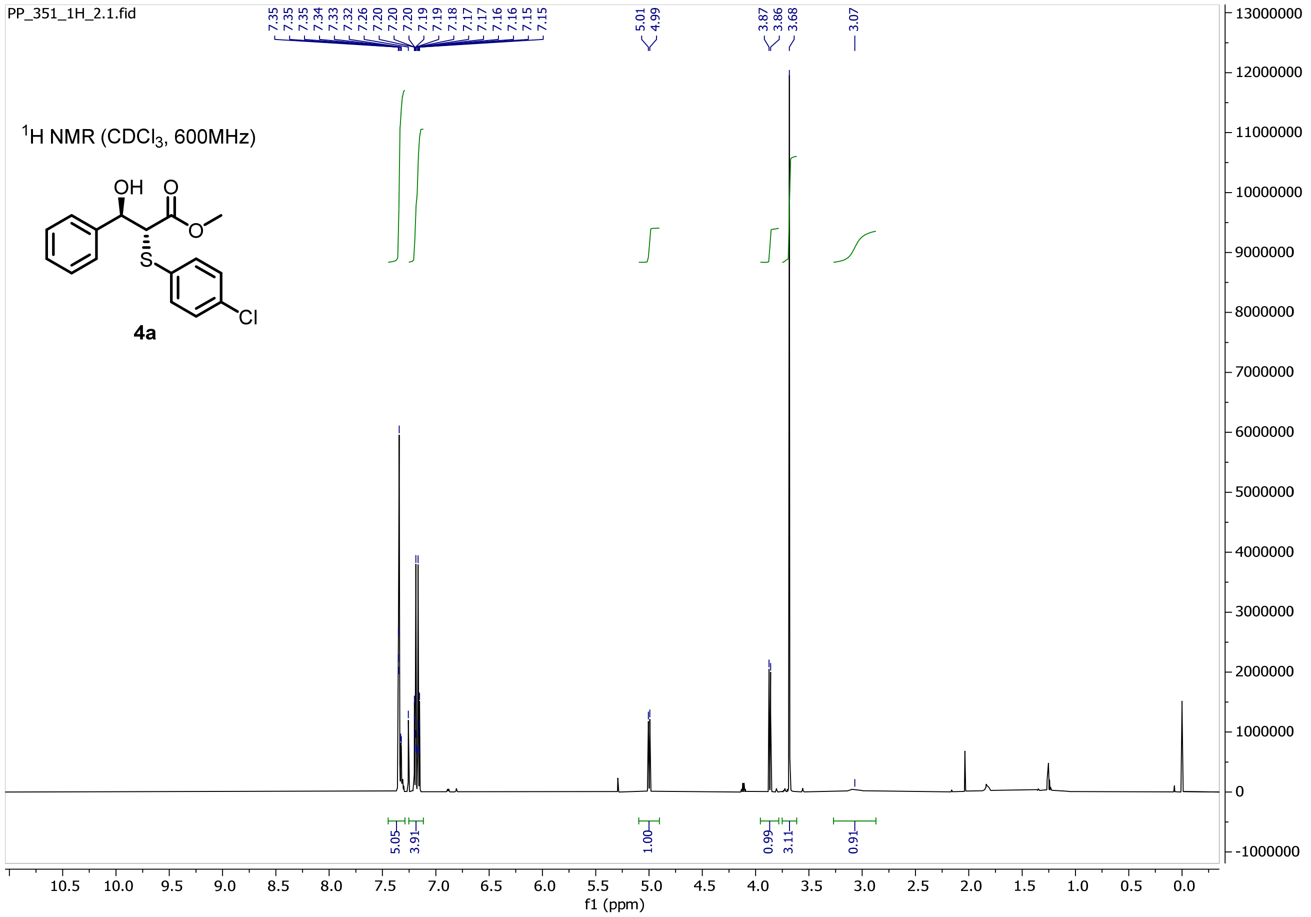
PP_351_1H_2.1.fid
7.35 7.35 7.35 7.34 7.33 7.32 7.26 7.20 7.20 7.20 7.19 7.19 7.19 7.18 7.17 7.17 7.16 7.16 7.15
5.01 4.99
3.87 3.86 3.68
3.07
^{1}H NMR ($CDCl_3$, 600MHz)
OH
O
S
Cl
4a
5.05 3.91 1.00 0.99 3.11 0.91
f1 (ppm)

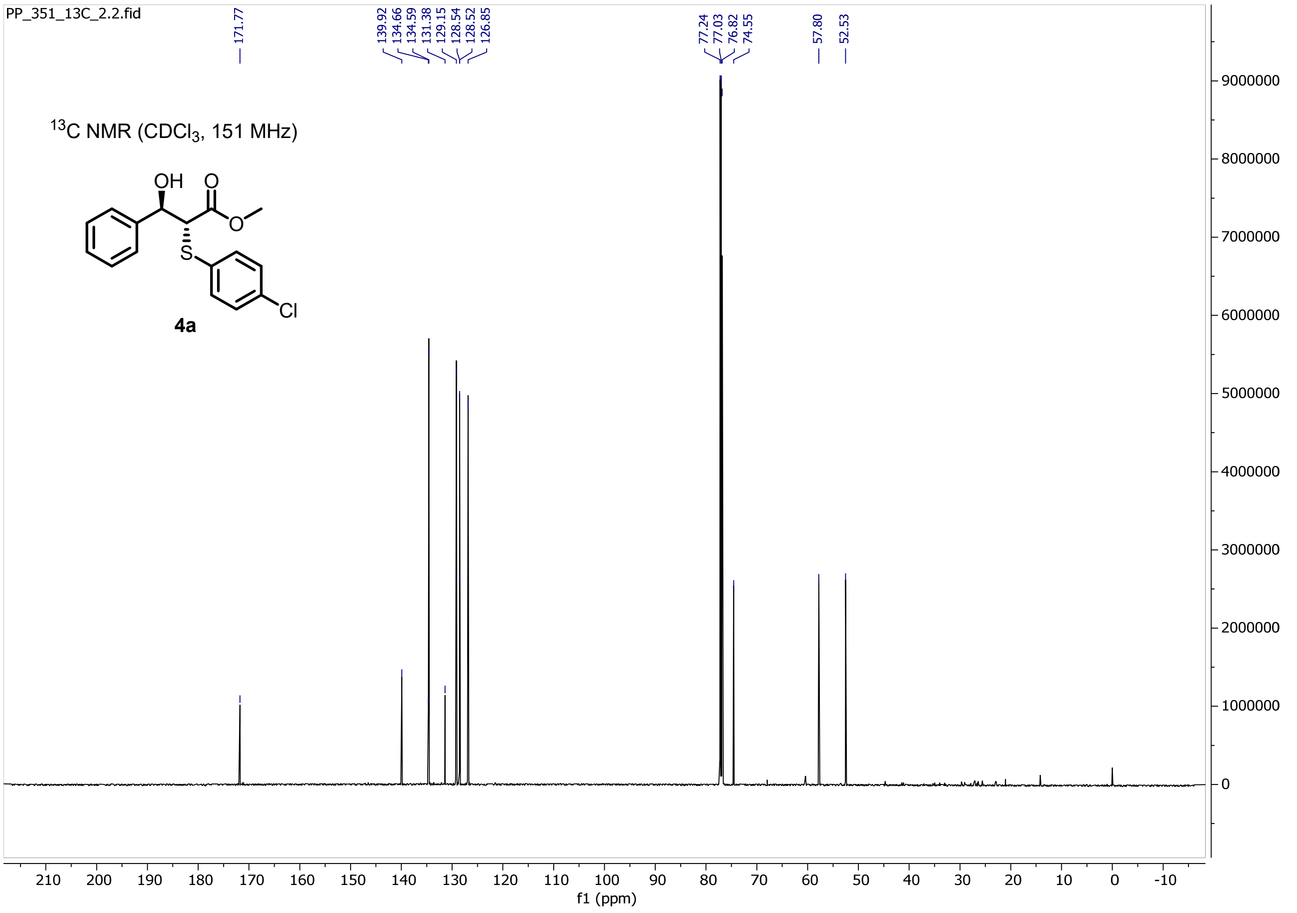
PP_351_13C_2.2.fid
171.77
139.92 134.66 134.59 131.38 129.15 128.54 128.52 126.85
77.24 77.03 76.82 74.55
57.80
52.53
^{13}C NMR ($CDCl_3$, 151 MHz)
OH
O
S
Cl
4a
f1 (ppm)

CSP HPLC traces:

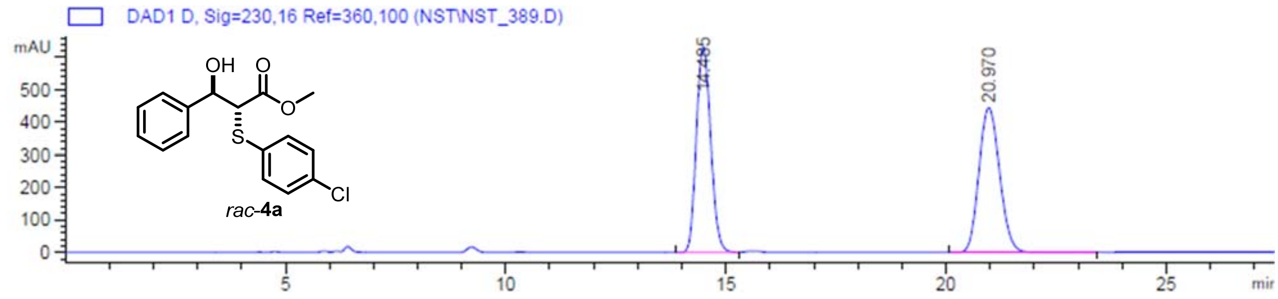

Signal 2: DAD1 D, Sig=230,16 Ref=360,100

Peak #	RetTime [min]	Type	Width [min]	Area [mAU*s]	Height [mAU]	Area %
1	14.485	BB	0.3585	1.43463e4	632.84003	49.6059
2	20.970	BB	0.5184	1.45742e4	444.63690	50.3941
Totals :				2.89206e4	1077.47693	

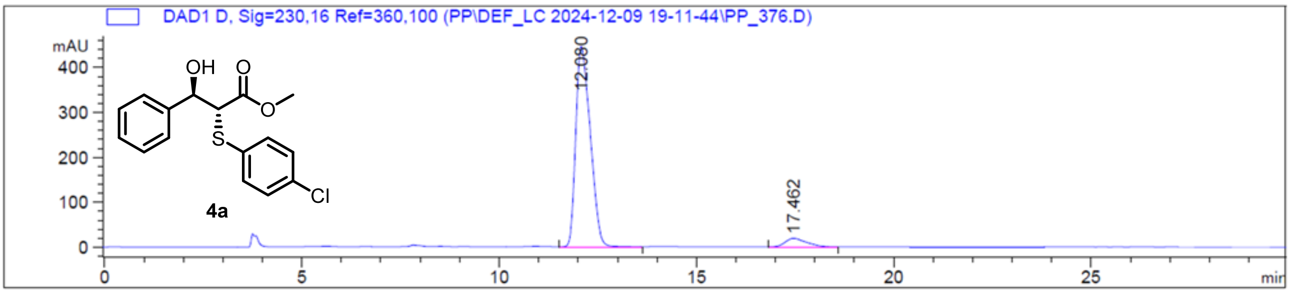

Signal 3: DAD1 D, Sig=230,16 Ref=360,100

Peak #	RetTime [min]	Type	Width [min]	Area [mAU*s]	Height [mAU]	Area %
1	12.080	BB	0.4215	1.13932e4	446.84055	94.1164
2	17.462	BB	0.5532	712.23889	18.91817	5.8836
Totals :				1.21054e4	465.75871	

4b- Methyl (2*R*,3*R*)-2-((4-chlorophenyl)thio)-3-hydroxy-3-(4-methoxyphenyl)propanoate

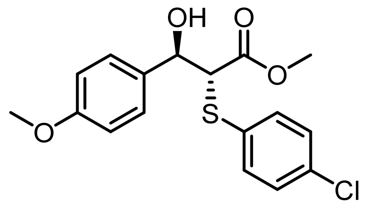

$C_{17}H_{17}ClO_4S$

MW: 352.8 g/mol

Yield: 79%

Colorless oil

The product (**4b**) was prepared using the general procedure **B** from **1a** (methyl 2-(dimethyl(oxo)-λ^6-sulfaneylidene)acetate), **2b** (4-methoxybenzaldehyde), and **3a** (4-chlorobenzenethiol). The product was obtained as a colorless oil (28 mg; 79% yield). The diastereomeric ratio obtained for the *anti*-couple is greater than 20:1. The enantiomeric excess of the product was determined by CSP HPLC: AD-H column, *n*-hexane/iPrOH 80:20, 0.75 mL/min, t_{maj} = 28.5 min; t_{min} = 19.0 min, 95% ee.

^{1}H NMR (600 MHz, $CDCl_3$) δ 7.30 – 7.23 (m, 2H), 7.21 – 7.15 (m, 4H), 6.88 – 6.84 (m, 2H), 4.95 (d, *J* = 8.5 Hz, 1H), 3.86 (d, *J* = 8.5 Hz, 1H), 3.80 (s, 3H), 3.69 (s, 3H), 2.98 (br s, 1H).

^{13}C NMR (151 MHz, $CDCl_3$) δ 171.8, 159.7, 134.6, 134.5, 132.0, 131.4, 129.1, 128.1, 113.9, 74.2, 57.9, 55.3, 52.5.

HRMS (MALDI$^+$) m/z calcd for $C_{17}H_{17}ClO_4SNa^+$[M+Na]$^+$: 375.0428, found: 375.0434.

$[\alpha]_D^{RT}$ = +34.0 (c = 1.0 g/100 mL, $CHCl_3$).

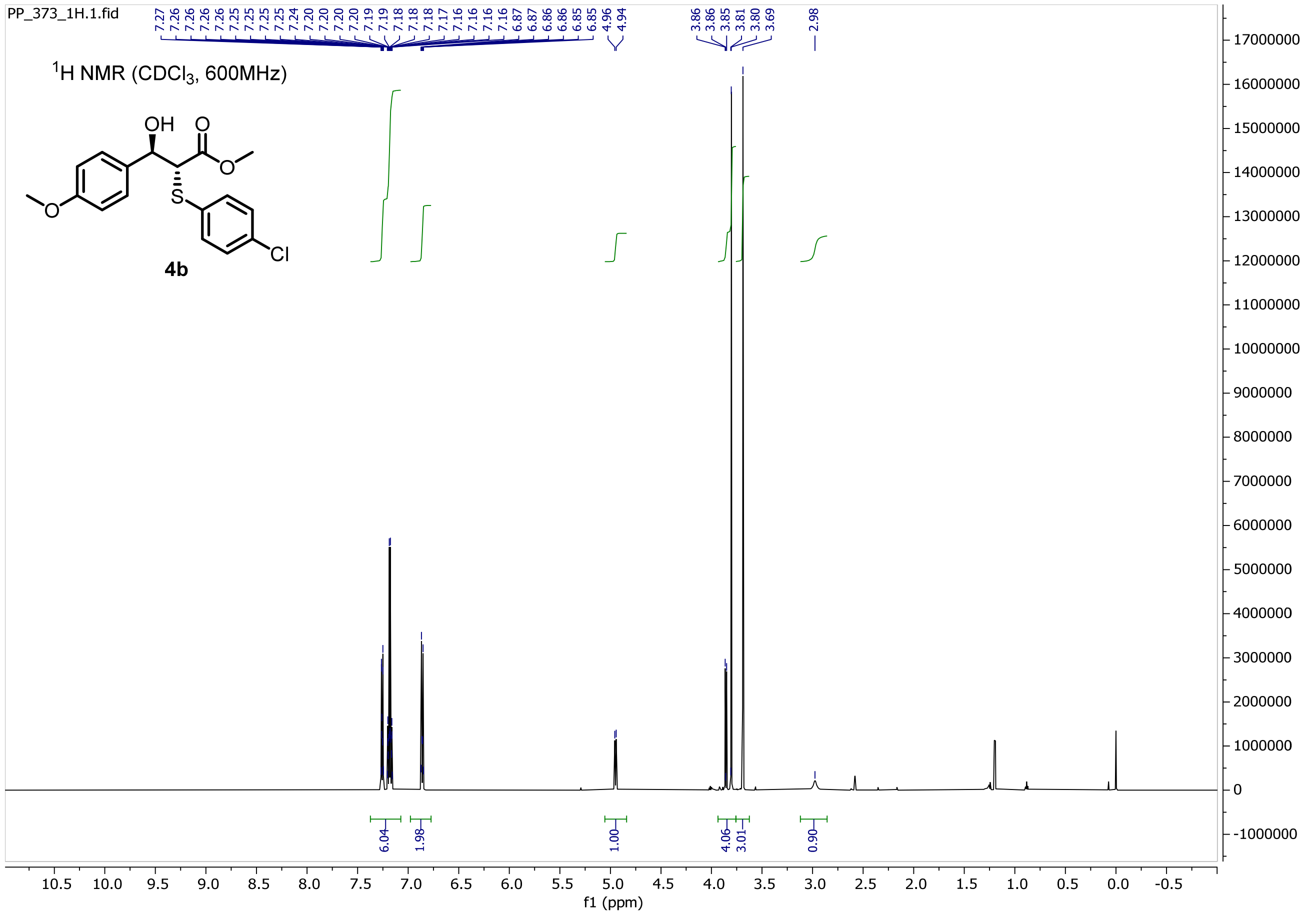
PP_373_1H.1.fid
7.27 7.26 7.26 7.26 7.25 7.25 7.25 7.25 7.24 7.20 7.20 7.20 7.19 7.19 7.18 7.18 7.18 7.17 7.16 7.16 7.16 6.87 6.87 6.86 6.86 6.85 6.85 4.96 4.94
3.86 3.86 3.85 3.81 3.80 3.69
2.98
1H NMR (CDCl3, 600MHz)
OH O
S
Cl
O
O
4b
6.04 1.98 1.00 4.06 3.01 0.90
f1 (ppm)

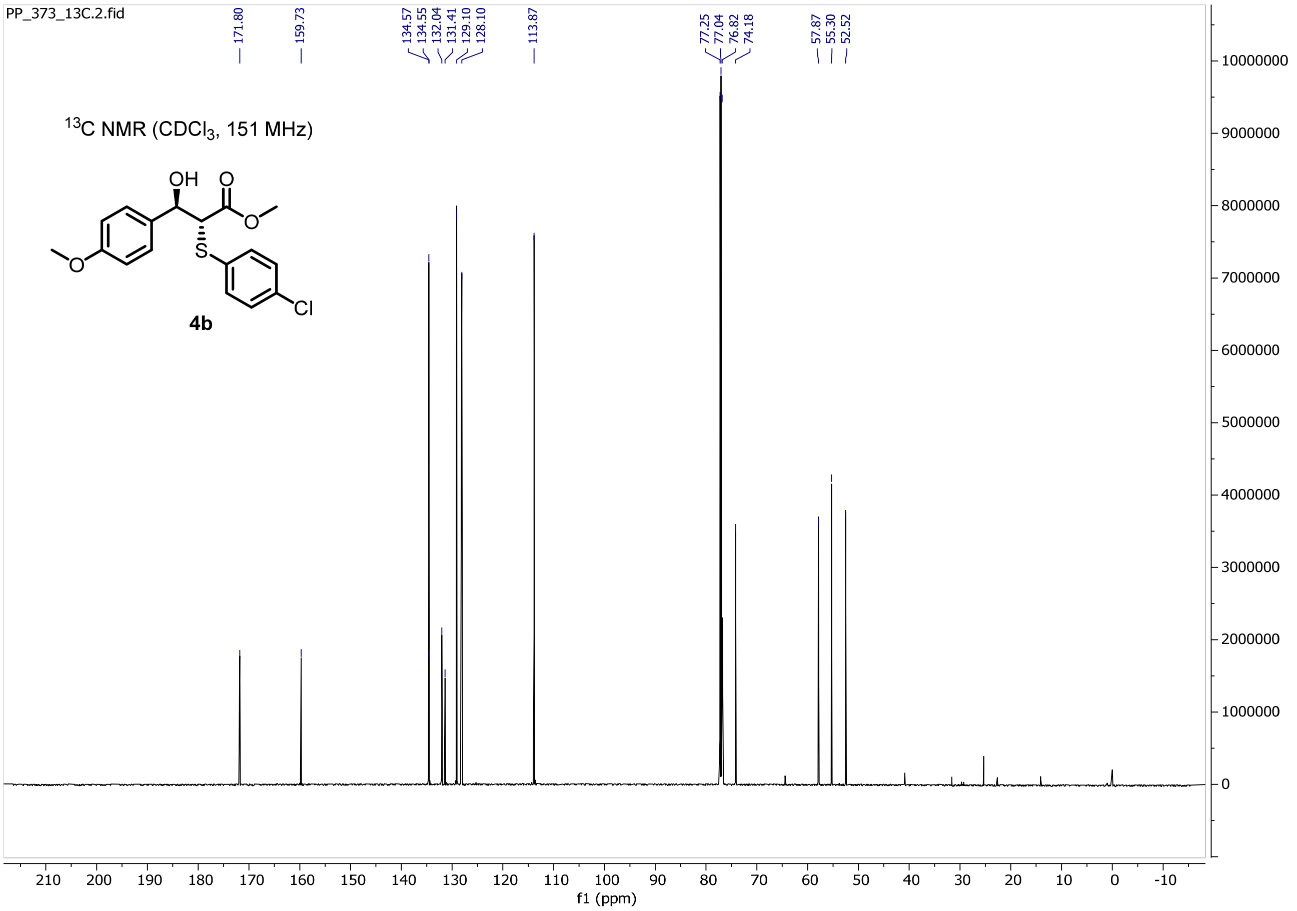
PP_373_13C.2.fid
171.80
159.73
134.57 134.55 132.04 131.41 129.10 128.10
113.87
77.25 77.04 76.82 74.18
57.87 55.30 52.52
13C NMR (CDCl3, 151 MHz)
OH O
S
Cl
O
O
4b
f1 (ppm)

CSP HPLC traces:

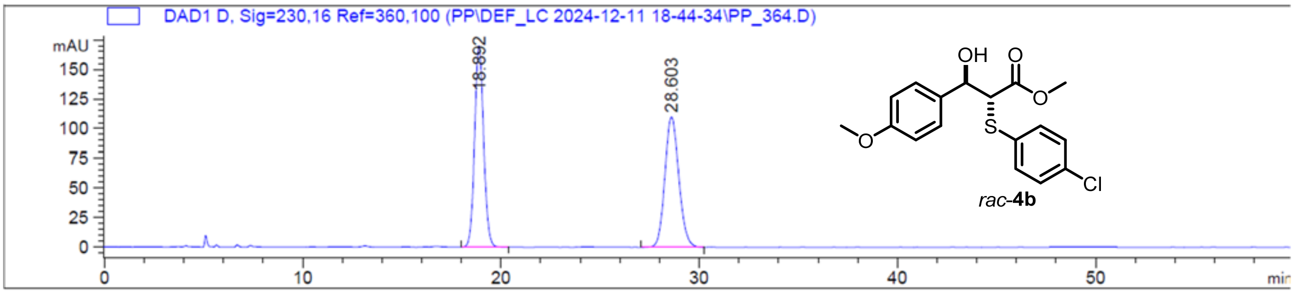

Signal 3: DAD1 D, Sig=230,16 Ref=360,100

Peak #	RetTime [min]	Type	Width [min]	Area [mAU*s]	Height [mAU]	Area %
1	18.892	BB	0.4893	5386.09668	170.17451	49.9563
2	28.603	BB	0.7568	5395.52637	109.90060	50.0437
Totals :				1.07816e4	280.07512	

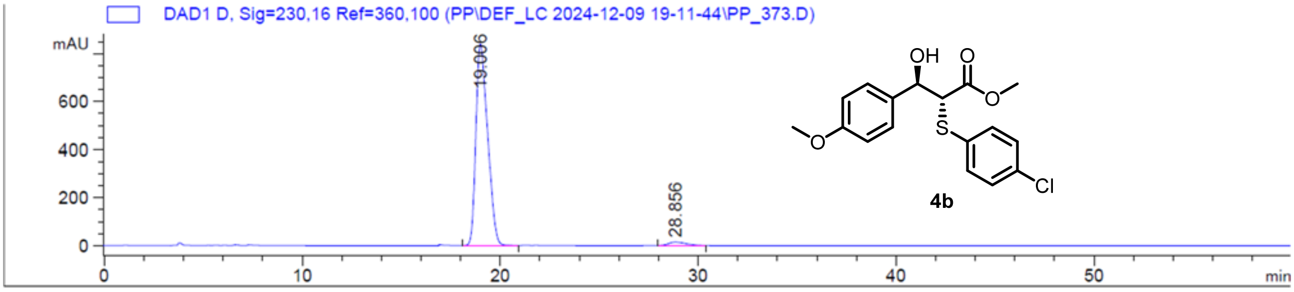

Signal 3: DAD1 D, Sig=230,16 Ref=360,100

Peak #	RetTime [min]	Type	Width [min]	Area [mAU*s]	Height [mAU]	Area %
1	19.006	BB	0.6161	3.46294e4	841.45966	97.5456
2	28.856	BB	0.8773	871.32941	14.33186	2.4544
Totals :				3.55007e4	855.79151	

4c- Methyl (2*R*,3*R*)-3-([1,1'-biphenyl]-4-yl)-2-((4-chlorophenyl)thio)-3-hydroxypropanoate

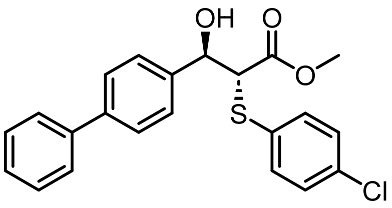

$C_{22}H_{19}ClO_3S$

MW: 398.9 g/mol

Yield: 93%;

White solid

The product (**4c**) was prepared using the general procedure **C** from **1a** (methyl 2-(dimethyl(oxo)-λ^6-sulfaneylidene)acetate), **2c** ([1,1'-biphenyl]-4-carbaldehyde), and **3a** (4-chlorobenzenethiol). The product was obtained as a white solid (35 mg; 93% yield). The diastereomeric ratio obtained for the *anti*-couple is greater than 20:1. The enantiomeric excess of the product was determined by CSP HPLC: AD-H column, *n*-hexane/iPrOH 80:20, 0.75 mL/min, t_{maj} = 15.6 min; t_{min} = 28.5 min, 87% ee.

^{1}H NMR (600 MHz, $CDCl_3$) δ 7.61 – 7.54 (m, 4H), 7.47 – 7.43 (m, 2H), 7.43 – 7.39 (m, 2H), 7.39 – 7.35 (m, 1H), 7.19 (s, 4H), 5.06 (d, *J* = 8.3 Hz, 1H), 3.92 (d, *J* = 8.3 Hz, 1H), 3.72 (s, 3H).

^{13}C NMR (151 MHz, $CDCl_3$) δ 171.8, 141.4, 140.5, 138.8, 134.7, 134.61, 134.59, 131.3, 129.2, 128.8, 127.5, 127.3, 127.2, 127.1, 74.4, 57.7, 52.6.

HRMS (MALDI$^+$) m/z calcd for $C_{18}H_{17}ClO_5SNa^+$ $[M+Na]^+$: 421.0636, found: 421.0641.

$[\alpha]_D^{RT}$ = +4.5 (c = 1.0 g/100 mL, $CHCl_3$).

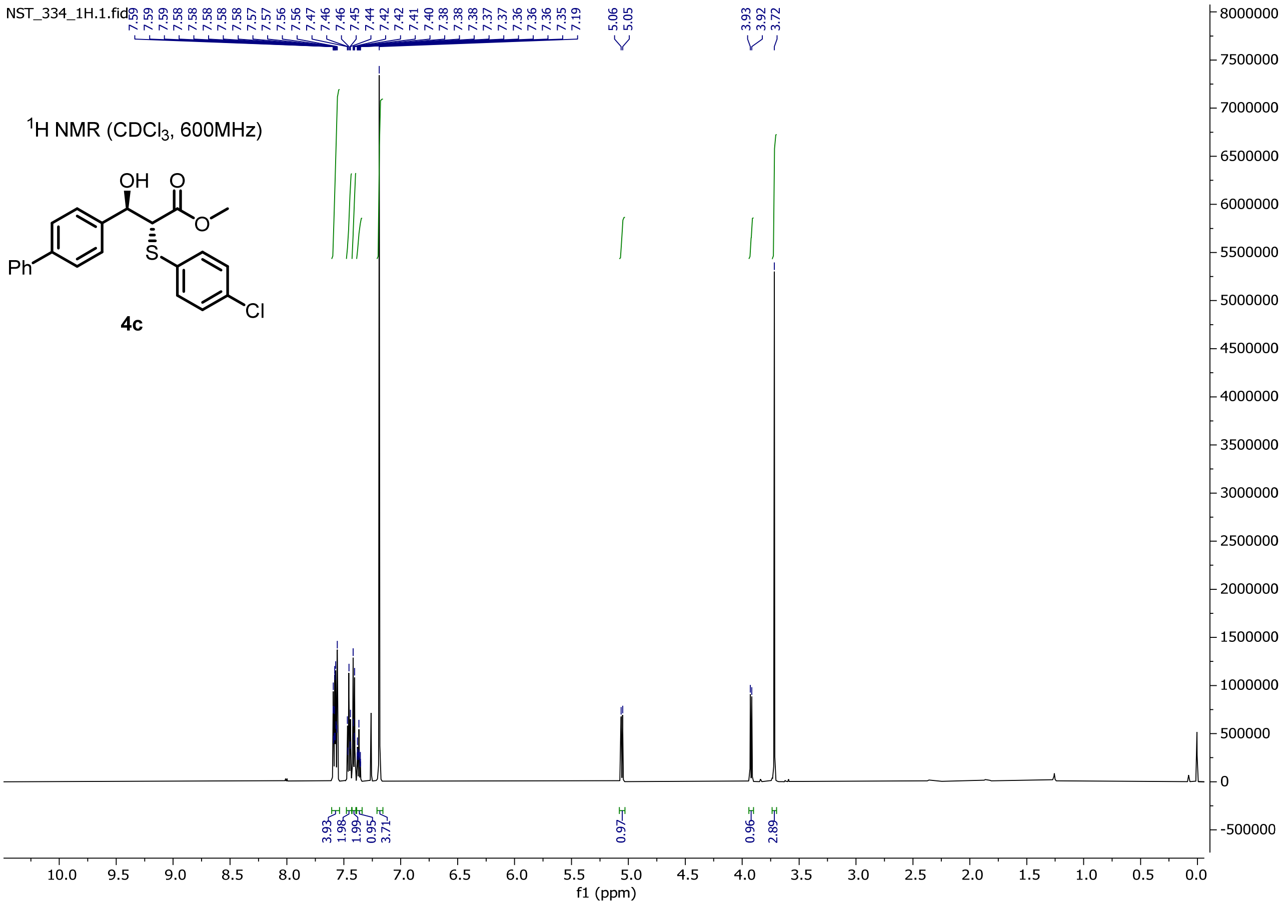
NST_334_1H.1.fid
7.59 7.59 7.58 7.58 7.58 7.58 7.57 7.57 7.56 7.56 7.47 7.46 7.46 7.45 7.44 7.42 7.42 7.41 7.40 7.38 7.38 7.38 7.37 7.37 7.36 7.36 7.36 7.35 7.19
5.06 5.05
3.93 3.92 3.72
1H NMR (CDCl3, 600MHz)
OH
O
Ph
S
Cl
4c
3.93 1.98 1.99 0.95 3.71
0.97
0.96 2.89
f1 (ppm)

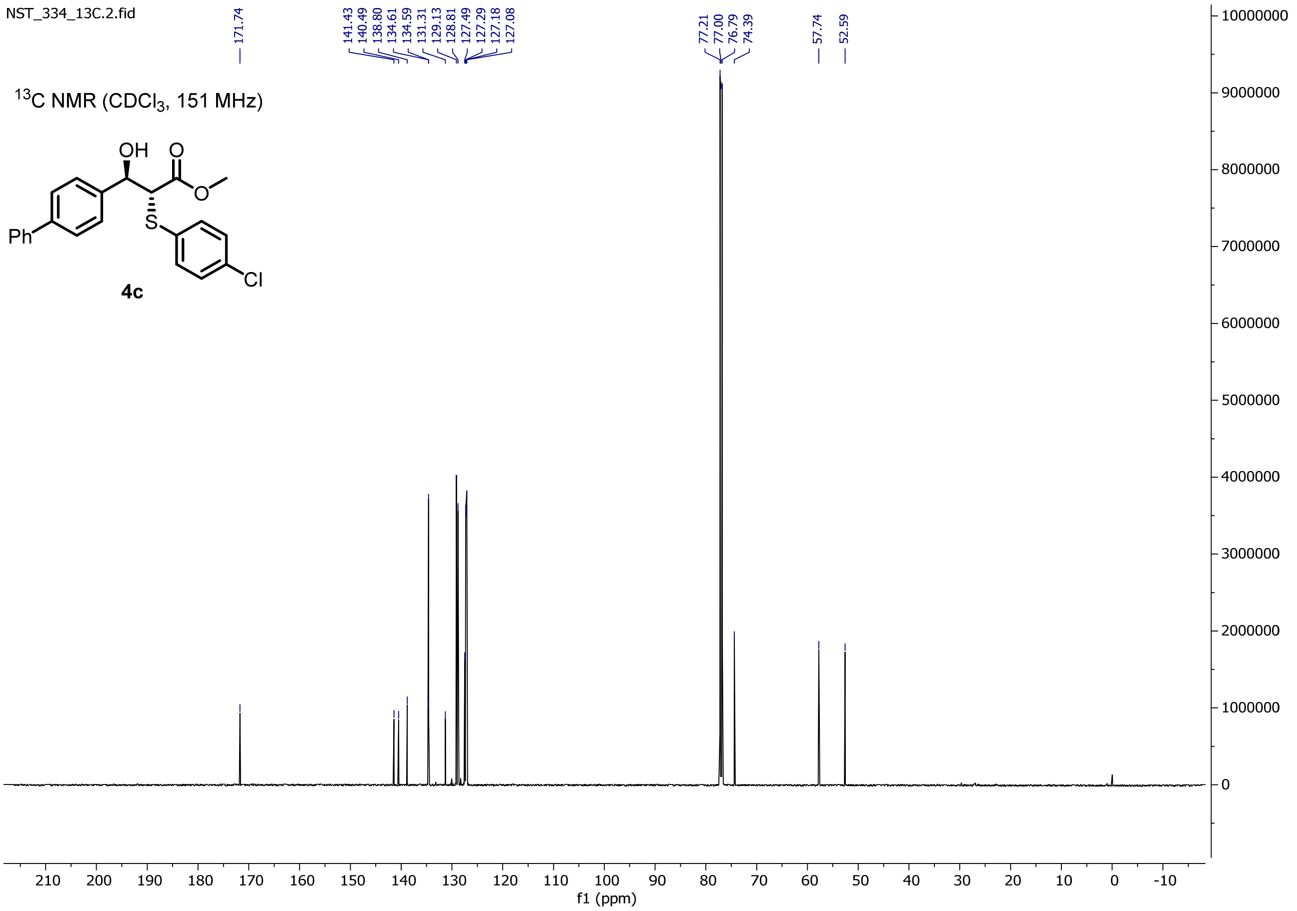
NST_334_13C.2.fid
171.74
141.43 140.49 138.80 134.61 134.59 131.31 129.13 128.81 127.49 127.29 127.18 127.08
77.21 77.00 76.79 74.39
57.74
52.59
13C NMR (CDCl3, 151 MHz)
OH
O
Ph
S
Cl
4c
f1 (ppm)

CSP HPLC traces:

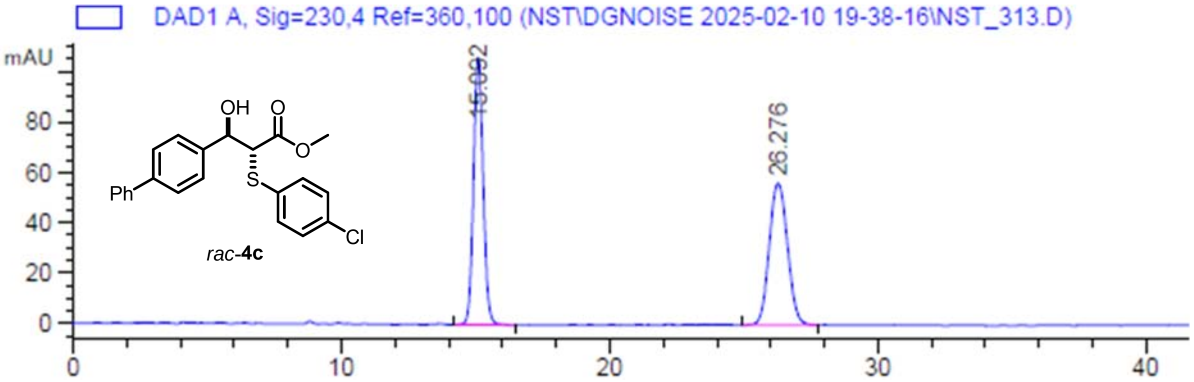

Signal 1: DAD1 A, Sig=230,4 Ref=360,100

Peak #	RetTime [min]	Type	Width [min]	Area [mAU*s]	Height [mAU]	Area %
1	15.092	BB	0.3887	2663.92163	106.17633	49.9980
2	26.276	BB	0.7499	2664.13281	56.52668	50.0020

Totals : 5328.05444 162.70301

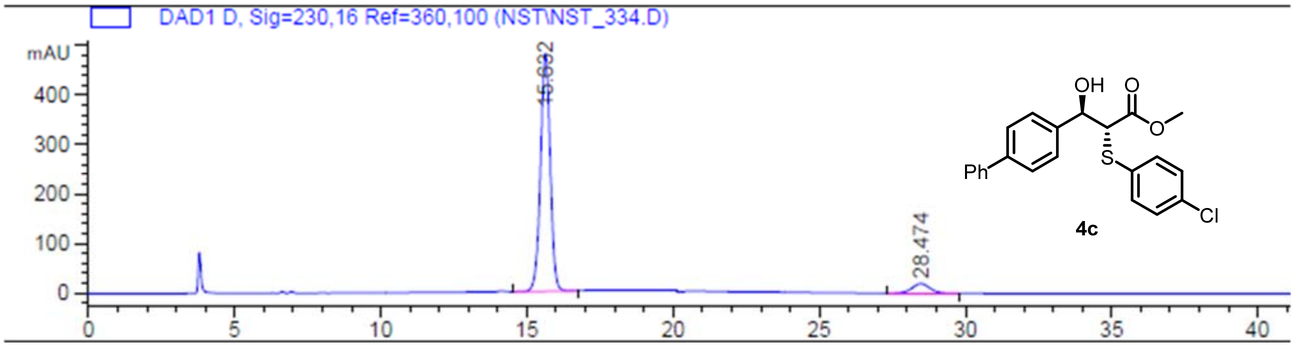

Signal 3: DAD1 D, Sig=230,16 Ref=360,100

Peak #	RetTime [min]	Type	Width [min]	Area [mAU*s]	Height [mAU]	Area %
1	15.632	BB	0.3551	1.10871e4	480.61987	93.4275
2	28.474	BB	0.6379	779.96619	18.80589	6.5725

Totals : 1.18671e4 499.42577

4d- Methyl (2*R*,3*R*)-3-(4-(*tert*-butyl)phenyl)-2-((4-chlorophenyl)thio)-3-hydroxypropanoate

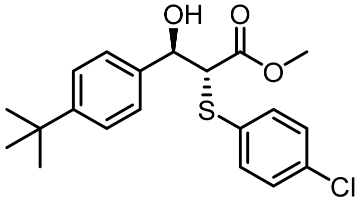

$C_{20}H_{23}ClO_5S$

MW: 378.9 g/mol

Yield: 84%;

Colorless oil

The product (**4d**) was prepared using the general procedure **C** from **1a** (methyl 2-(dimethyl(oxo)-λ^6-sulfaneylidene)acetate), **2d** (4-(*tert*-butyl)benzaldehyde), and **3a** (4-chlorobenzenethiol). The product was obtained as a colorless oil (32 mg; 84% yield). The diastereomeric ratio obtained for the *anti*-couple is greater than 20:1. The enantiomeric excess of the product was determined by CSP HPLC: AD-H column, *n*-hexane/iPrOH 80:20, 0.75 mL/min, t_{maj} = 13.9 min; t_{min} = 11.9 min, 85% ee.

^{1}H NMR (600 MHz, $CDCl_3$) δ 7.36 – 7.32 (m, 2H), 7.28 – 7.23 (m, 2H), 7.17 – 7.13 (m, 2H), 7.12 – 7.08 (m, 2H), 4.98 (d, *J* = 8.6 Hz, 1H), 3.87 (d, *J* = 8.6 Hz, 1H), 3.71 (s, 3H), 1.31 (s, 9H).

^{13}C NMR (151 MHz, $CDCl_3$) δ 171.8, 151.6, 136.8, 134.5, 134.4, 131.5, 129.0, 126.7, 125.4, 74.6, 57.8, 52.6, 34.6, 31.3.

$[\alpha]_D^{RT}$ = +15.2 (c = 1.0 g/100 mL, $CHCl_3$).

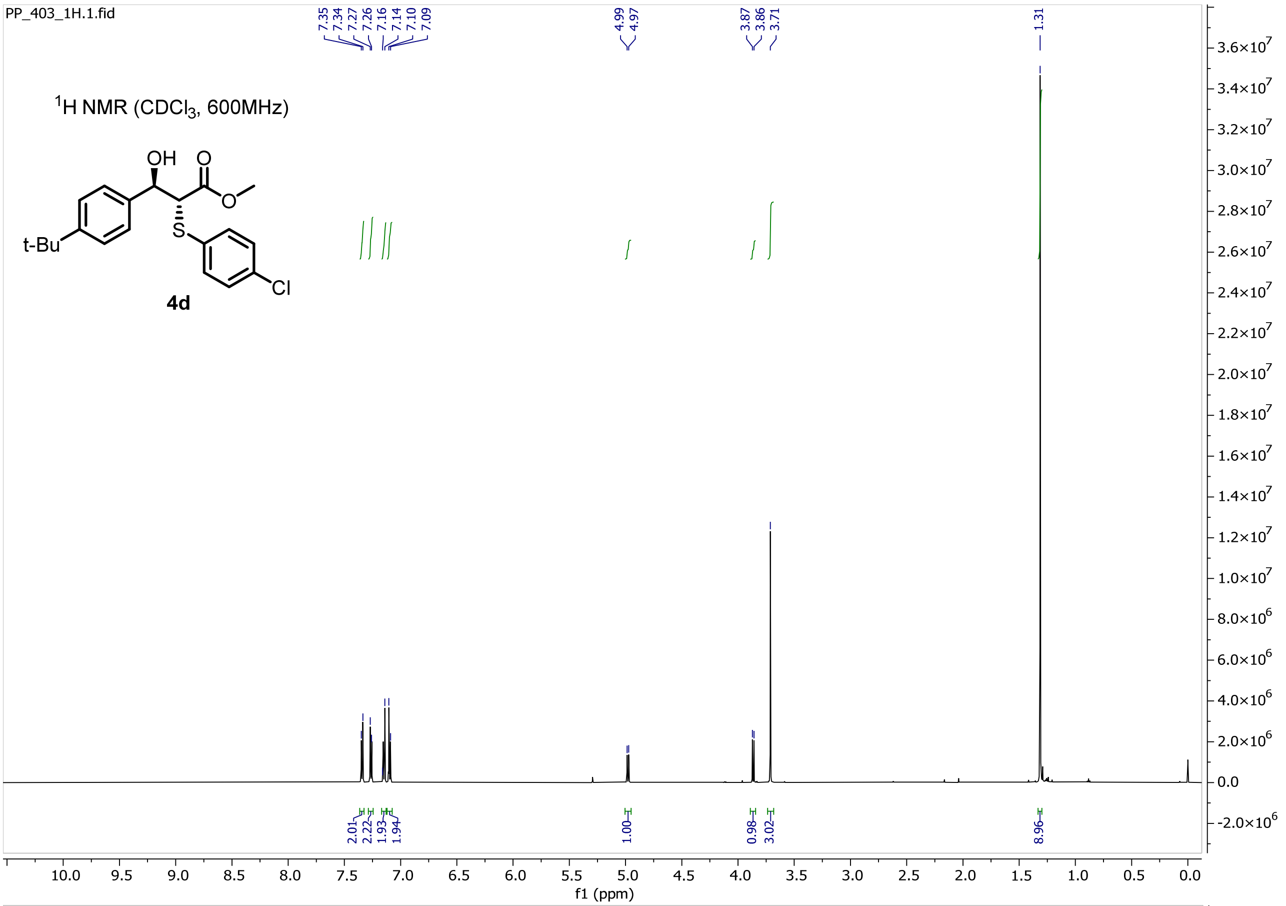
PP_403_1H.1.fid
1H NMR (CDCl3, 600MHz)
OH
O
O
S
t-Bu
Cl
4d
7.35
7.34
7.27
7.26
7.16
7.14
7.10
7.09
4.99
4.97
3.87
3.86
3.71
1.31
2.01
2.22
1.93
1.94
1.00
0.98
3.02
8.96
f1 (ppm)

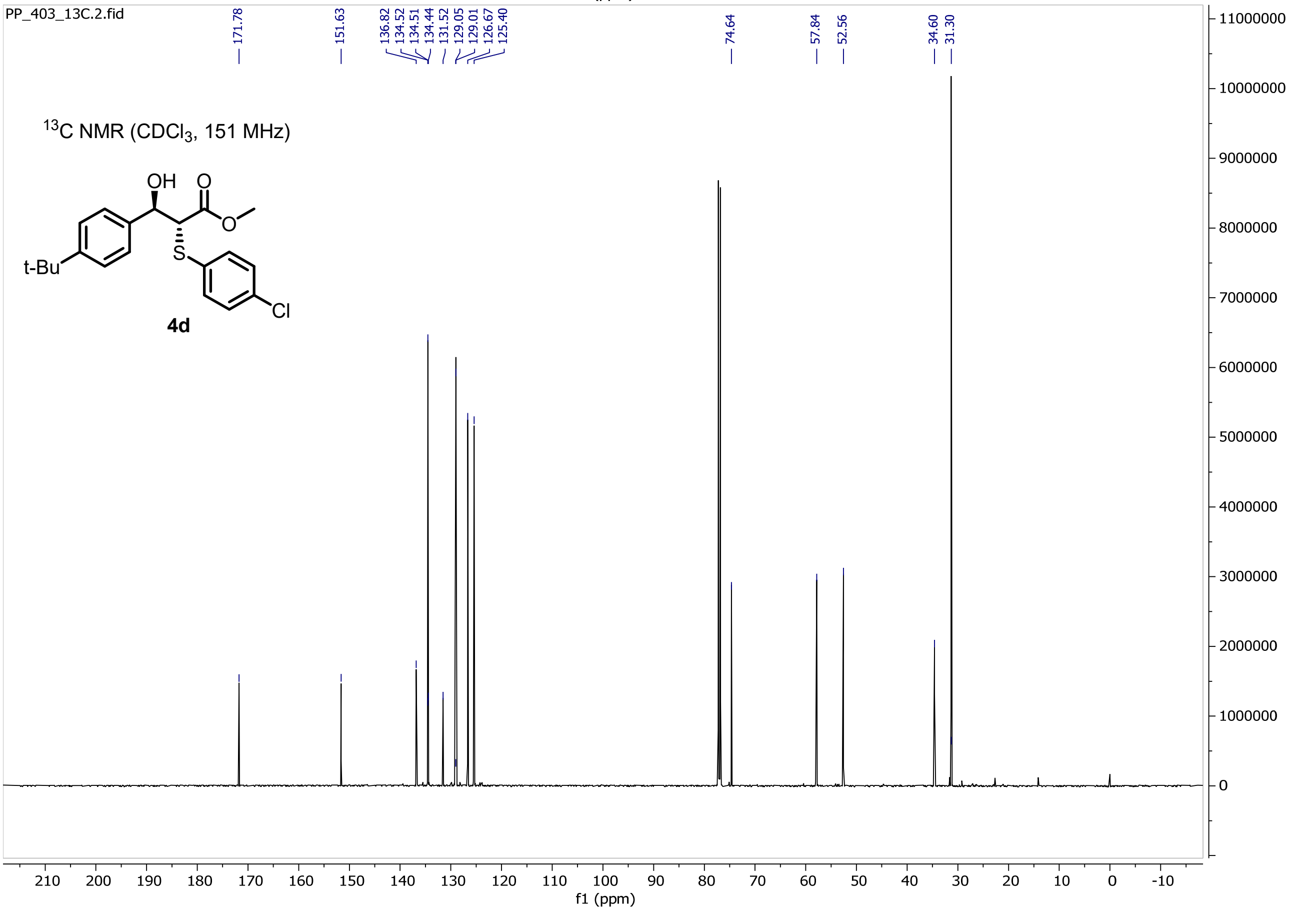
PP_403_13C.2.fid
13C NMR (CDCl3, 151 MHz)
OH
O
O
S
t-Bu
Cl
4d
171.78
151.63
136.82
134.52
134.51
134.44
131.52
129.05
129.01
126.67
125.40
74.64
57.84
52.56
34.60
31.30
f1 (ppm)

CSP HPLC traces:

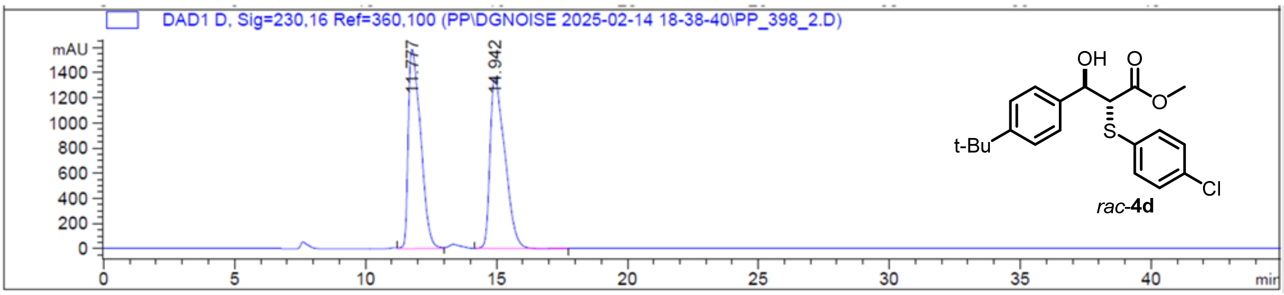

Signal 3: DAD1 D, Sig=230,16 Ref=360,100

Peak #	RetTime [min]	Type	Width [min]	Area [mAU*s]	Height [mAU]	Area %
1	11.777	VV	0.4753	5.20936e4	1585.40662	49.3902
2	14.942	BB	0.5664	5.33800e4	1358.67065	50.6098
Totals :				1.05474e5	2944.07727	

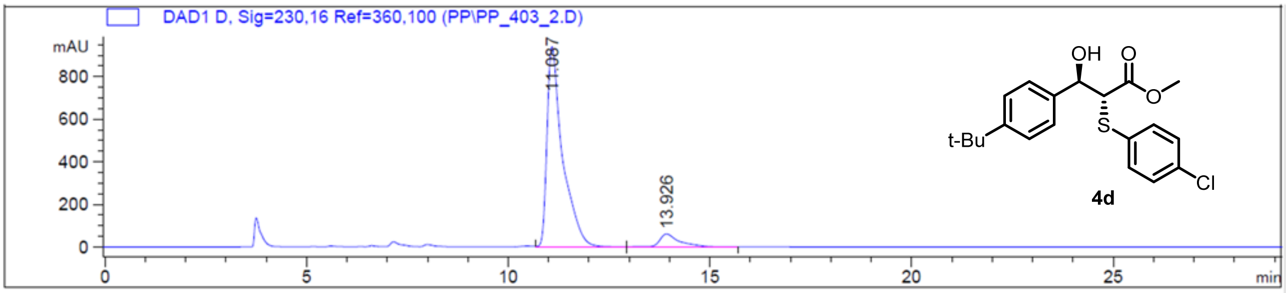

Signal 3: DAD1 D, Sig=230,16 Ref=360,100

Peak #	RetTime [min]	Type	Width [min]	Area [mAU*s]	Height [mAU]	Area %
1	11.087	VB	0.3849	2.52386e4	940.93335	92.6346
2	13.926	BB	0.4800	2006.73389	60.02813	7.3654
Totals :				2.72453e4	1000.96148	

4e- Methyl (2*R*,3*R*)-2-((4-chlorophenyl)thio)-3-hydroxy-3-(*p*-tolyl)propanoate

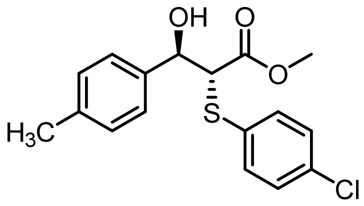

$C_{17}H_{17}ClO_3S$

MW: 336.1 g/mol

Yield: 72%;

Colorless oil

The product (**4e**) was prepared using the general procedure **B** from **1a** (methyl 2-(dimethyl(oxo)-λ^6-sulfaneylidene)acetate), **2e** (4-methylbenzaldehyde), and **3a** (4-chlorobenzenethiol). The product was obtained as a colorless oil (24.3 mg; 72% yield). The diastereomeric ratio obtained for the *anti*-couple is greater than 20:1. The enantiomeric excess of the product was determined by CSP HPLC: AD-H column, *n*-hexane/iPrOH 80:20, 0.75 mL/min, t_{maj} = 13.7 min; t_{min} = 23.0 min, 90% ee.

^{1}H NMR (600 MHz, $CDCl_3$) δ 7.25 – 7.22 (m, 2H), 7.21 – 7.17 (m, 4H), 7.15 (d, *J* = 7.8 Hz, 2H), 4.97 (dd, *J* = 8.3, 5.3 Hz, 1H), 3.88 (d, *J* = 8.2 Hz, 1H), 3.68 (s, 3H), 2.96 (d, *J* = 5.3 Hz, 1H), 2.35 (s, 3H).

^{13}C NMR (151 MHz, $CDCl_3$) δ 171.7, 138.4, 136.9, 134.6, 131.3, 129.2, 129.1, 126.7, 74.4, 57.6, 52.5, 21.2.

HRMS (MALDI$^+$) m/z calcd for $C_{17}H_{17}ClO_3SNa^+$ $[M+Na]^+$: 359.0479, found: 359.0485.

$[\alpha]_D^{RT}$ = +18.8 (c = 1.0 g/100 mL, $CHCl_3$).

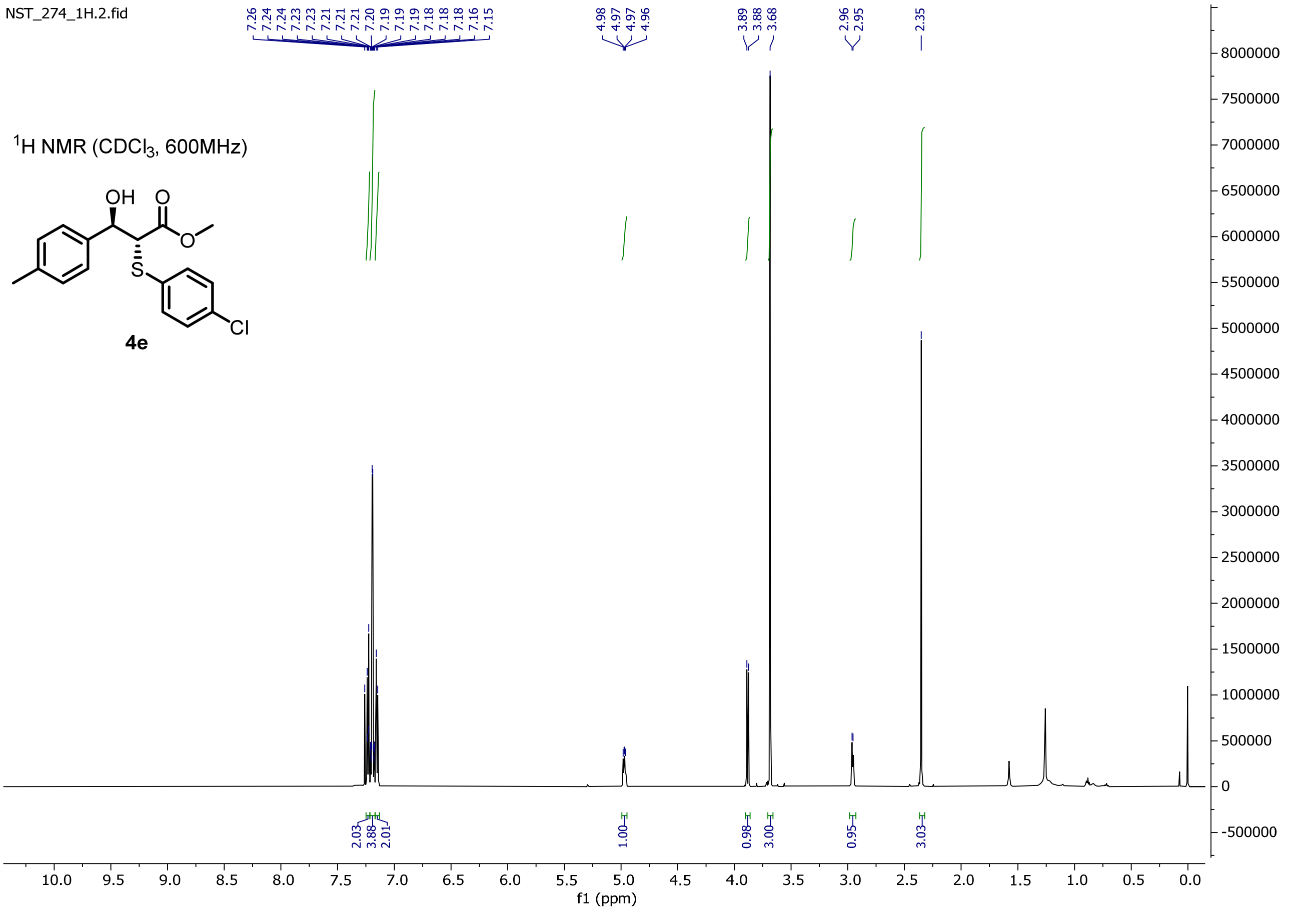
NST_274_1H.2.fid
1H NMR (CDCl3, 600MHz)
4e
f1 (ppm)

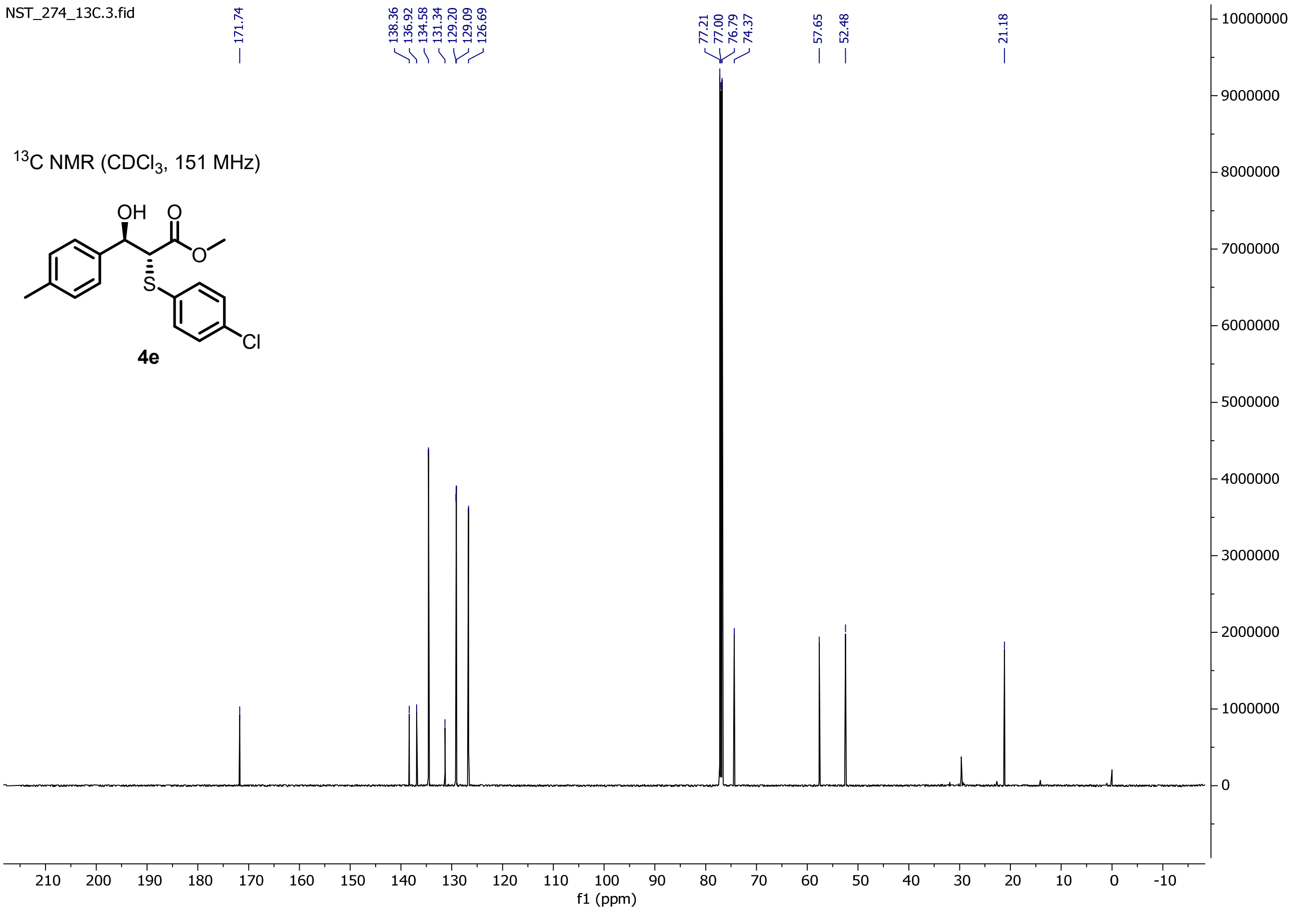
NST_274_13C.3.fid
13C NMR (CDCl3, 151 MHz)
4e
f1 (ppm)

CSP HPLC traces:

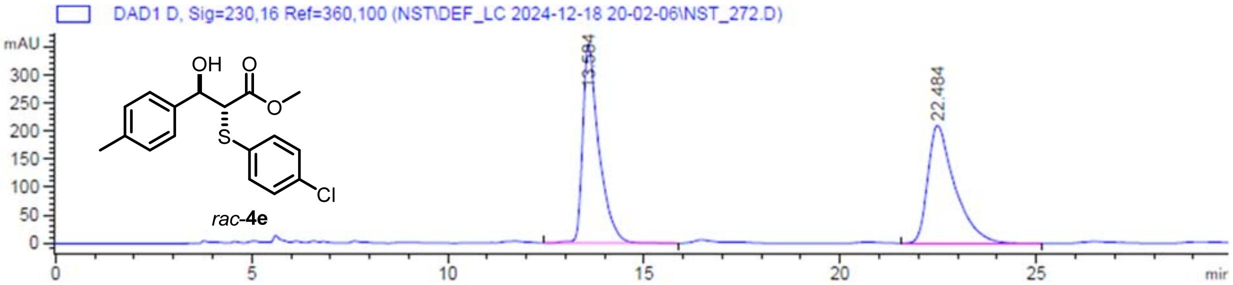

Signal 3: DAD1 D, Sig=230,16 Ref=360,100

Peak #	RetTime [min]	Type	Width [min]	Area [mAU*s]	Height [mAU]	Area %
1	13.584	BB	0.4127	1.01155e4	354.74472	50.2710
2	22.484	BB	0.6958	1.00064e4	209.30698	49.7290
Totals :				2.01219e4	564.05170	

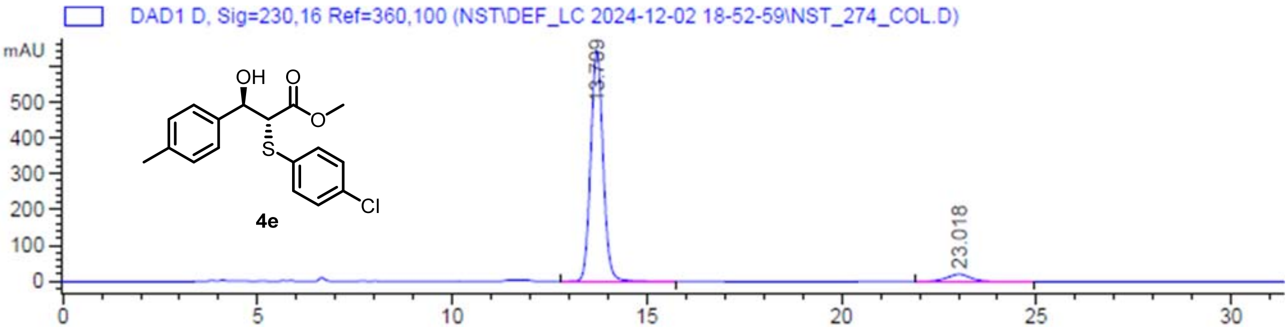

Signal 3: DAD1 D, Sig=230,16 Ref=360,100

Peak #	RetTime [min]	Type	Width [min]	Area [mAU*s]	Height [mAU]	Area %
1	13.709	BB	0.3408	1.41302e4	641.84338	94.8051
2	23.018	BB	0.6129	774.27283	19.18083	5.1949
Totals :				1.49044e4	661.02421	

4f- Methyl (2*R*,3*R*)-2-((4-chlorophenyl)thio)-3-(2,3-dihydrobenzo[*b*][1,4]dioxin-6-yl)-3-hydroxypropanoate

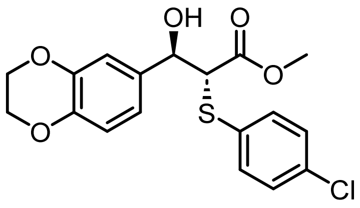

$C_{18}H_{17}ClO_5S$

MW: 380.8 g/mol

Yield: 64%;

Colorless oil

The product (**4f**) was prepared using the general procedure **C** from **1a** (methyl 2-(dimethyl(oxo)-λ^6-sulfaneylidene)acetate), **2f** (2,3-dihydrobenzo[*b*][1,4]dioxine-6-carbaldehyde), and **3a** (4-chlorobenzenethiol). The product was obtained as a colorless oil (24.5 mg; 64% yield). The diastereomeric ratio obtained for the *anti*-couple is greater than 20:1. The enantiomeric excess of the product was determined by CSP HPLC: AD-H column, *n*-hexane/iPrOH 80:20, 0.75 mL/min, t_{maj} = 34.2 min; t_{min} = 25.9 min, 87% ee.

^{1}H NMR (600 MHz, $CDCl_3$) δ 7.20 (s, 4H), 6.84 – 6.80 (m, 3H), 4.89 (d, *J* = 8.4 Hz, 1H), 4.27 – 4.21 (m, 4H), 3.85 (d, *J* = 8.4 Hz, 1H), 3.70 (s, 3H).

^{13}C NMR (151 MHz, $CDCl_3$) δ 171.6, 143.7, 143.5, 134.5, 134.4, 133.2, 131.4, 129.1, 120.0, 117.2, 115.7, 74.2, 64.33, 64.29, 57.6, 52.5.

HRMS (MALDI$^+$) m/z calcd for $C_{18}H_{17}ClO_5SNa^+$[M+Na]$^+$: 403.0377, found: 403.0383.

$[\alpha]_D^{RT}$ = +16.8 (c = 1.0 g/100 mL, $CHCl_3$).

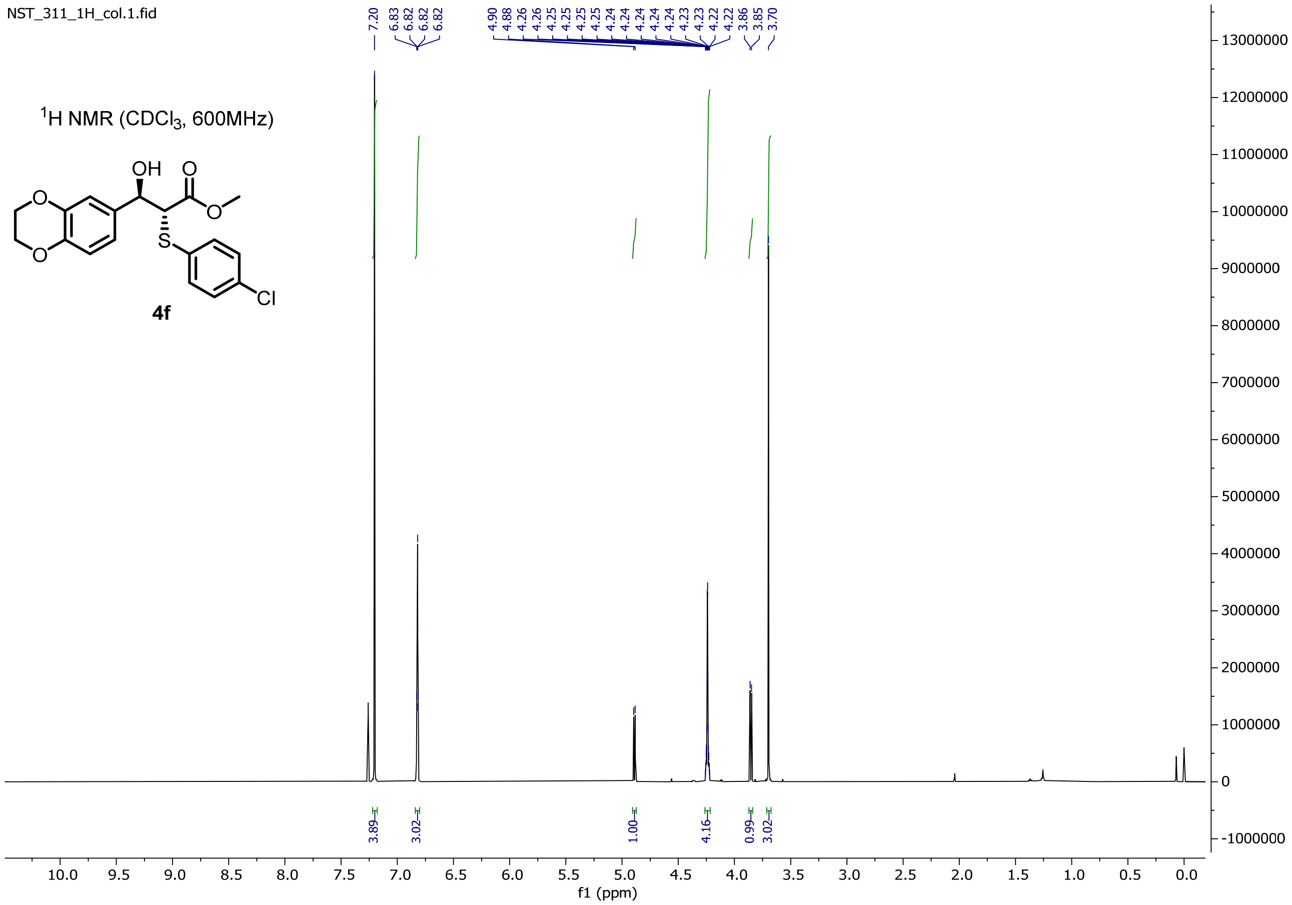
NST_311_1H_col.1.fid
^{1}H NMR (CDCl$_3$, 600MHz)
OH
O
O
O
O
S
Cl
4f
7.20
6.83 6.82 6.82 6.82
4.90 4.88 4.26 4.26 4.25 4.25 4.25 4.25 4.24 4.24 4.24 4.24 4.24 4.23 4.22 4.22 3.86 3.85 3.70
3.89 3.02 1.00 4.16 0.99 3.02
f1 (ppm)

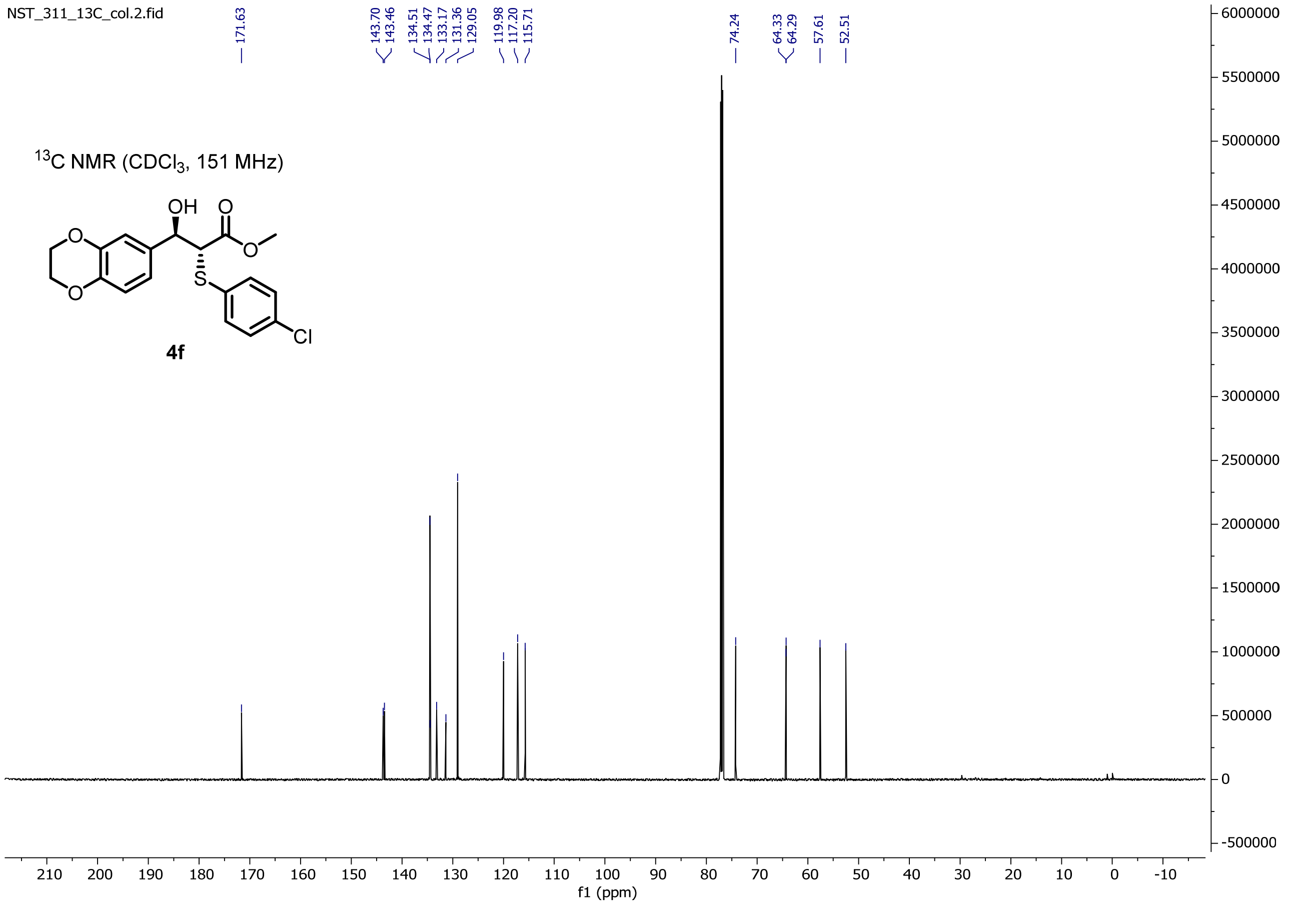
NST_311_13C_col.2.fid
^{13}C NMR (CDCl$_3$, 151 MHz)
OH
O
O
O
O
S
Cl
4f
171.63
143.70 143.46 134.51 134.47 133.17 131.36 129.05 119.98 117.20 115.71
74.24
64.33 64.29 57.61 52.51
f1 (ppm)

CSP HPLC traces:

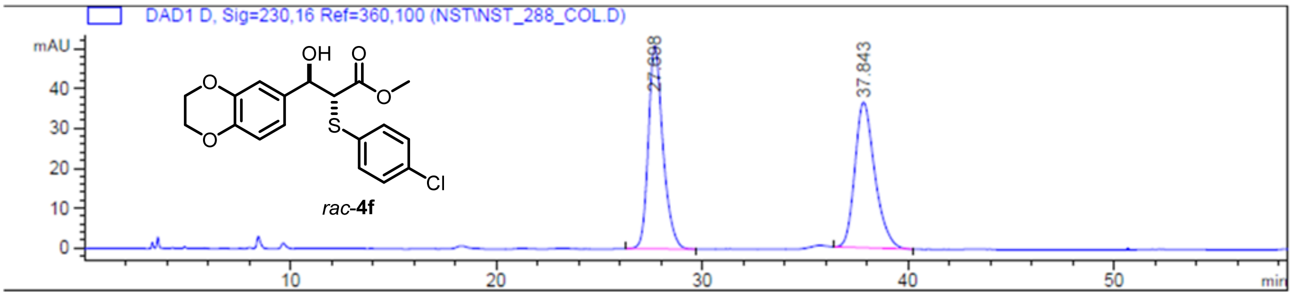

Signal 2: DAD1 B, Sig=254,16 Ref=360,100

Peak #	RetTime [min]	Type	Width [min]	Area [mAU*s]	Height [mAU]	Area %
1	27.697	BB	0.7261	705.92706	14.34627	50.5824
2	37.845	BB	0.9602	689.67230	10.26037	49.4176

Totals : 1395.59937 24.60664

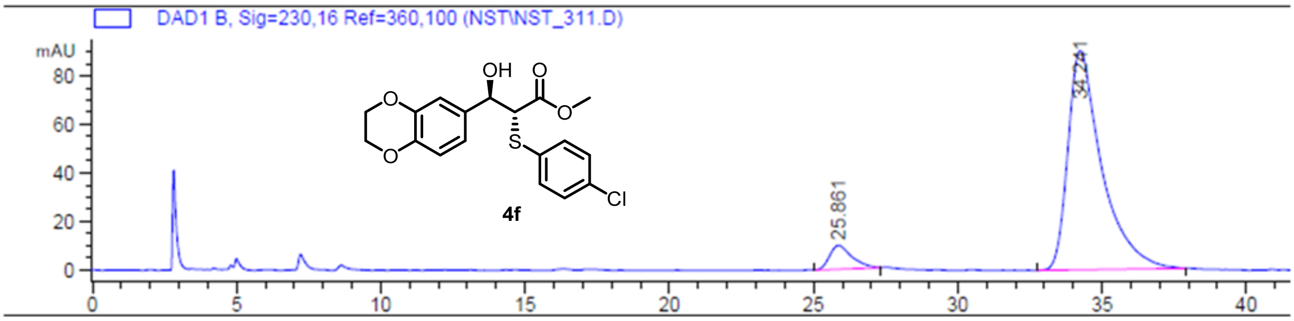

Signal 2: DAD1 B, Sig=230,16 Ref=360,100

Peak #	RetTime [min]	Type	Width [min]	Area [mAU*s]	Height [mAU]	Area %
1	25.861	BB	0.7638	521.83691	9.75963	6.6079
2	34.241	BB	1.1540	7375.38623	90.38462	93.3921

Totals : 7897.22314 100.14425

4g- Methyl (2*R*,3*R*)-2-((4-chlorophenyl)thio)-3-hydroxy-3-(naphthalen-2-yl)propanoate

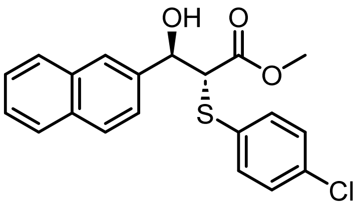

$C_{20}H_{17}ClO_3S$

MW: 372.9 g/mol

Yield: 95%;

White solid

The product (**4g**) was prepared using the general procedure **B** from **1a** (methyl 2-(dimethyl(oxo)-λ^6-sulfaneylidene)acetate), **2g** (2-naphthaldehyde), and **3a** (4-chlorobenzenethiol). The product was obtained as a white solid (35 mg; 95% yield). The diastereomeric ratio obtained for the *anti*-couple is greater than 20:1. The enantiomeric excess of the product was determined by CSP HPLC: AD-H column, *n*-hexane/iPrOH 80:20, 0.75 mL/min, t_{maj} = 19.6 min; t_{min} = 29.6 min, 92% ee.

^{1}H NMR (600 MHz, $CDCl_3$) δ 7.86 – 7.78 (m, 4H), 7.54 – 7.46 (m, 2H), 7.44 (dd, *J* = 8.5, 1.8 Hz, 1H), 7.18 – 7.10 (m, 4H), 5.17 (d, *J* = 8.1 Hz, 1H), 4.01 (d, *J* = 8.1 Hz, 1H), 3.68 (s, 3H).

^{13}C NMR (151 MHz, $CDCl_3$) δ 171.7, 137.1, 134.6, 134.5, 133.3, 133.0, 131.2, 129.1, 128.4, 128.1, 127.7, 126.4, 126.3, 124.0, 74.7, 57.5, 52.6.

HRMS (MALDI$^+$) m/z calcd for $C_{20}H_{18}ClO_3S^+$[M+H]$^+$: 395.0479, found: 395.0485.

$[\alpha]_D^{RT}$ = +5.5 (c = 1.0 g/100 mL, $CHCl_3$).

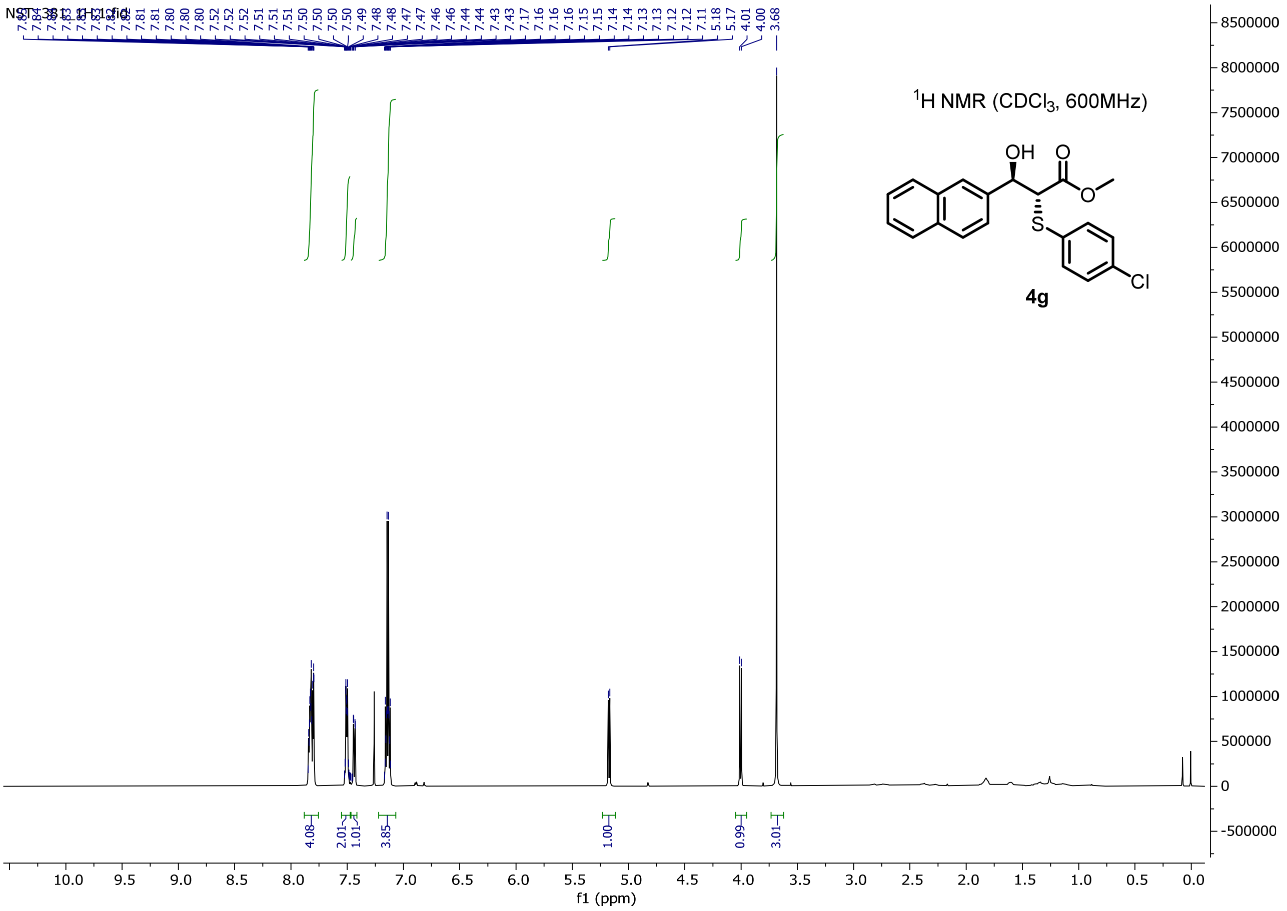
1H NMR (CDCl3, 600MHz)
OH
O
O
S
Cl
4g
4.08
2.01
1.01
3.85
1.00
0.99
3.01
f1 (ppm)

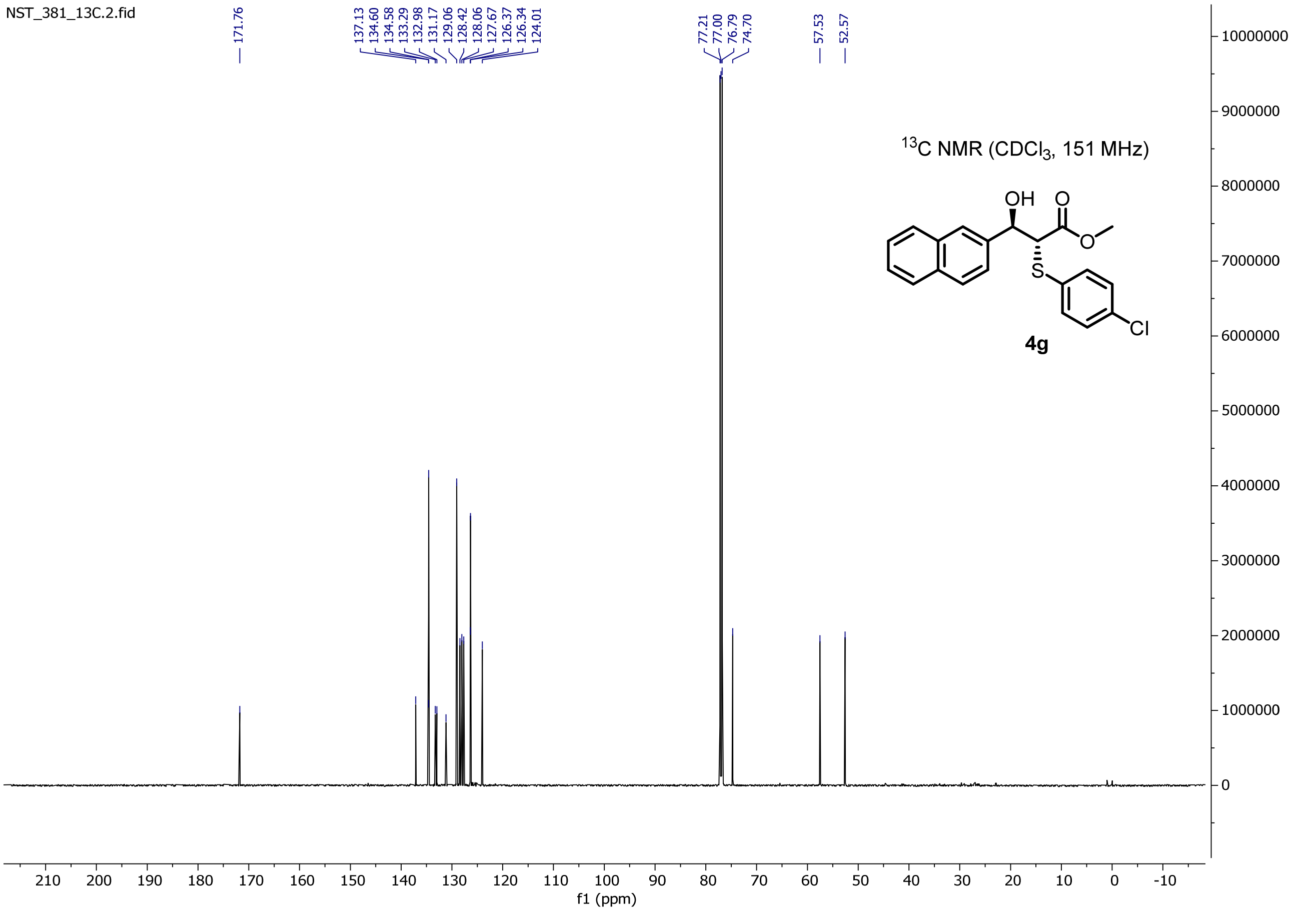
NST_381_13C.2.fid
171.76
137.13
134.60
134.58
133.29
132.98
131.17
129.06
128.42
128.06
127.67
126.37
126.34
124.01
77.21
77.00
76.79
74.70
57.53
52.57
13C NMR (CDCl3, 151 MHz)
OH
O
O
S
Cl
4g
f1 (ppm)

CSP HPLC traces:

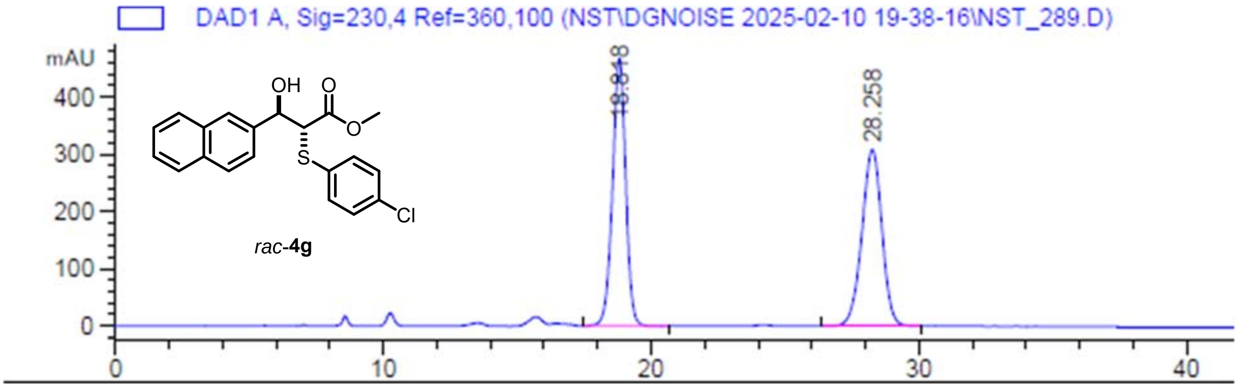

Signal 1: DAD1 A, Sig=230,4 Ref=360,100

Peak #	RetTime [min]	Type	Width [min]	Area [mAU*s]	Height [mAU]	Area %
1	18.818	BB	0.5259	1.60367e4	467.83075	49.9939
2	28.258	BB	0.7942	1.60407e4	308.72638	50.0061
Totals :				3.20774e4	776.55713	

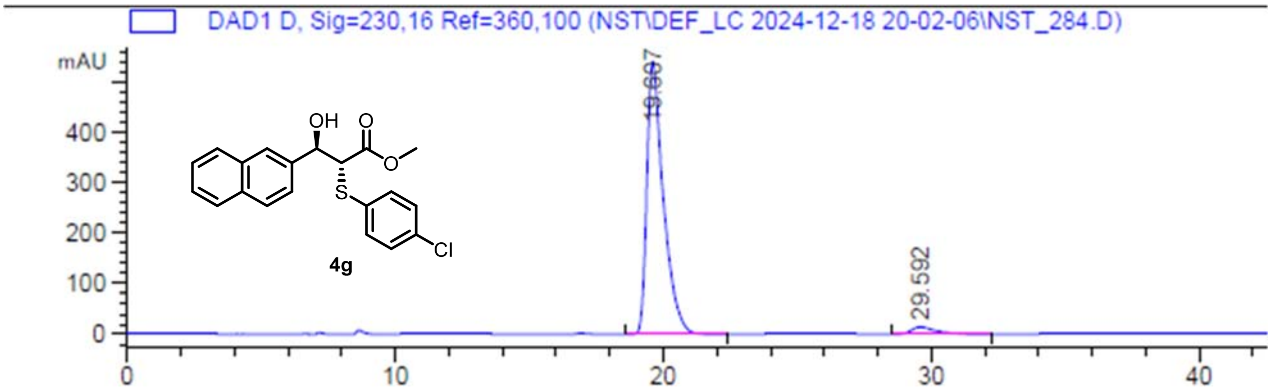

Signal 3: DAD1 D, Sig=230,16 Ref=360,100

Peak #	RetTime [min]	Type	Width [min]	Area [mAU*s]	Height [mAU]	Area %
1	19.607	BB	0.6164	2.29893e4	540.33356	96.1647
2	29.592	BB	0.8984	916.87659	14.40912	3.8353
Totals :				2.39062e4	554.74268	

4h- Methyl (2*R*,3*R*)-2-((4-chlorophenyl)thio)-3-hydroxy-3-(naphthalen-1-yl)propanoate

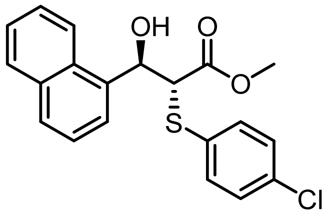

$C_{20}H_{17}ClO_3S$

MW: 372.8 g/mol

Yield: 95%;

Pale yellow solid

The product (**4h**) was prepared using the general procedure **B** from **1a** (methyl 2-(dimethyl(oxo)-λ^6-sulfaneylidene)acetate), **2h** (1-naphthaldehyde), and **3a** (4-chlorobenzenethiol). The product was obtained as a white solid (35 mg; 95% yield). The diastereomeric ratio obtained for the *anti*-couple is greater than 20:1. The enantiomeric excess of the product was determined by CSP HPLC: AD-H column, *n*-hexane/iPrOH 80:20, 0.75 mL/min, t_{maj} = 12.6 min; t_{min} = 20.4 min, 91% ee.

^{1}H NMR (600 MHz, $CDCl_3$) δ 8.02 – 7.97 (m, 1H), 7.89 – 7.83 (m, 1H), 7.80 (br d, *J* = 8.1 Hz, 1H), 7.56 (dt, *J* = 7.0, 1.0 Hz, 1H), 7.51 – 7.47 (m, 2H), 7.43 (dd, *J* = 8.2, 7.1 Hz, 1H), 7.14 – 7.07 (m, 4H), 5.76 (d, *J* = 7.4 Hz, 1H), 4.25 (d, *J* = 7.4 Hz, 1H), 3.66 (s, 3H).

^{13}C NMR (151 MHz, $CDCl_3$) δ 171.7, 135.3, 134.6, 134.5, 133.9, 131.1, 130.7, 129.1, 129.05, 129.03, 126.4, 125.7, 125.1, 124.8, 122.9, 72.1, 56.9, 52.5.

HRMS (MALDI$^+$) m/z calcd for $C_{20}H_{18}ClO_3S^+$[M+H]$^+$: 395.0479, found: 395.0485.

$[\alpha]_D^{RT}$ = +6.9 (c = 1.0 g/100 mL, $CHCl_3$).

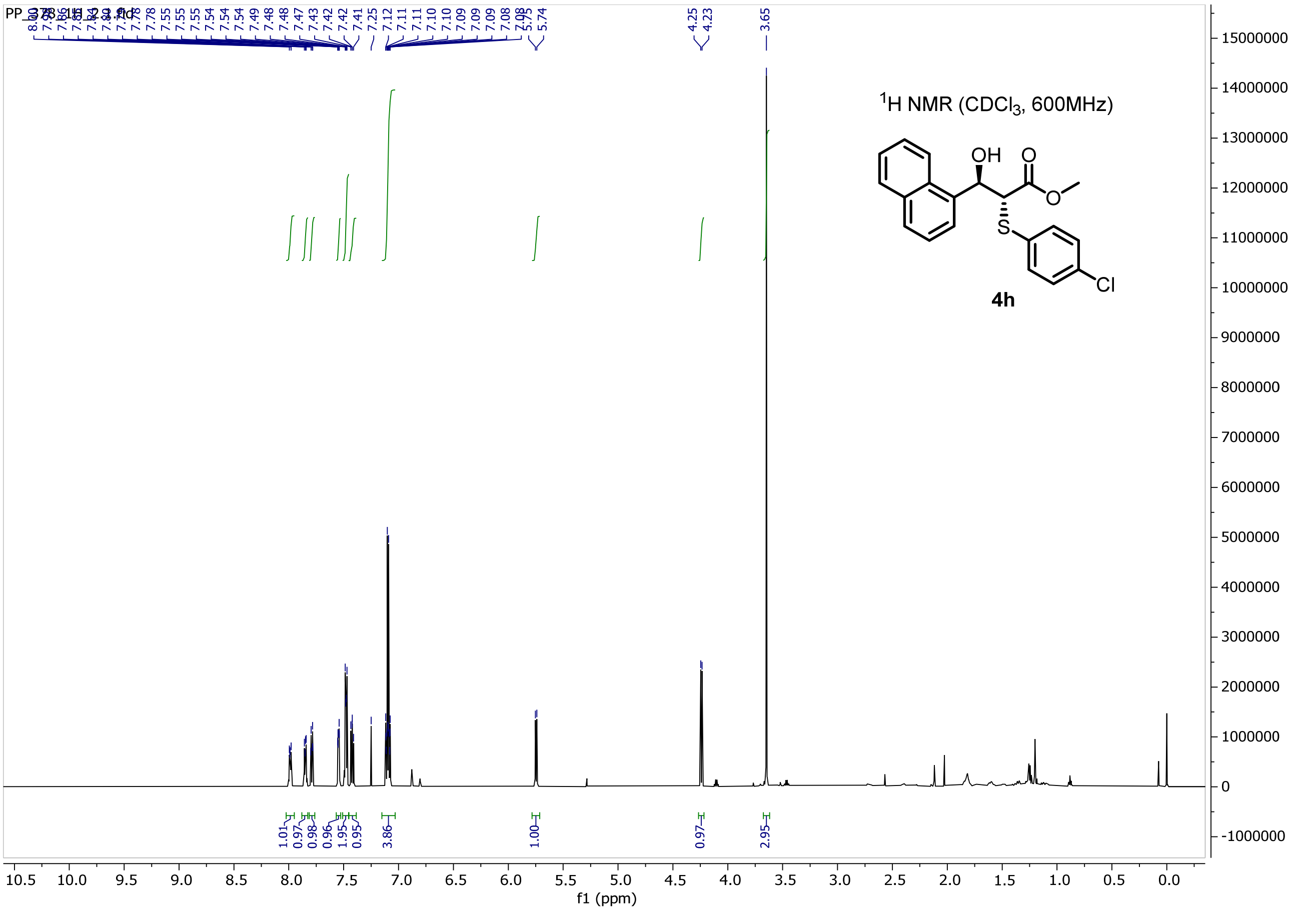
PP_378_1H_2.2.fid
1H NMR (CDCl3, 600MHz)
OH
O
O
S
Cl
4h
f1 (ppm)

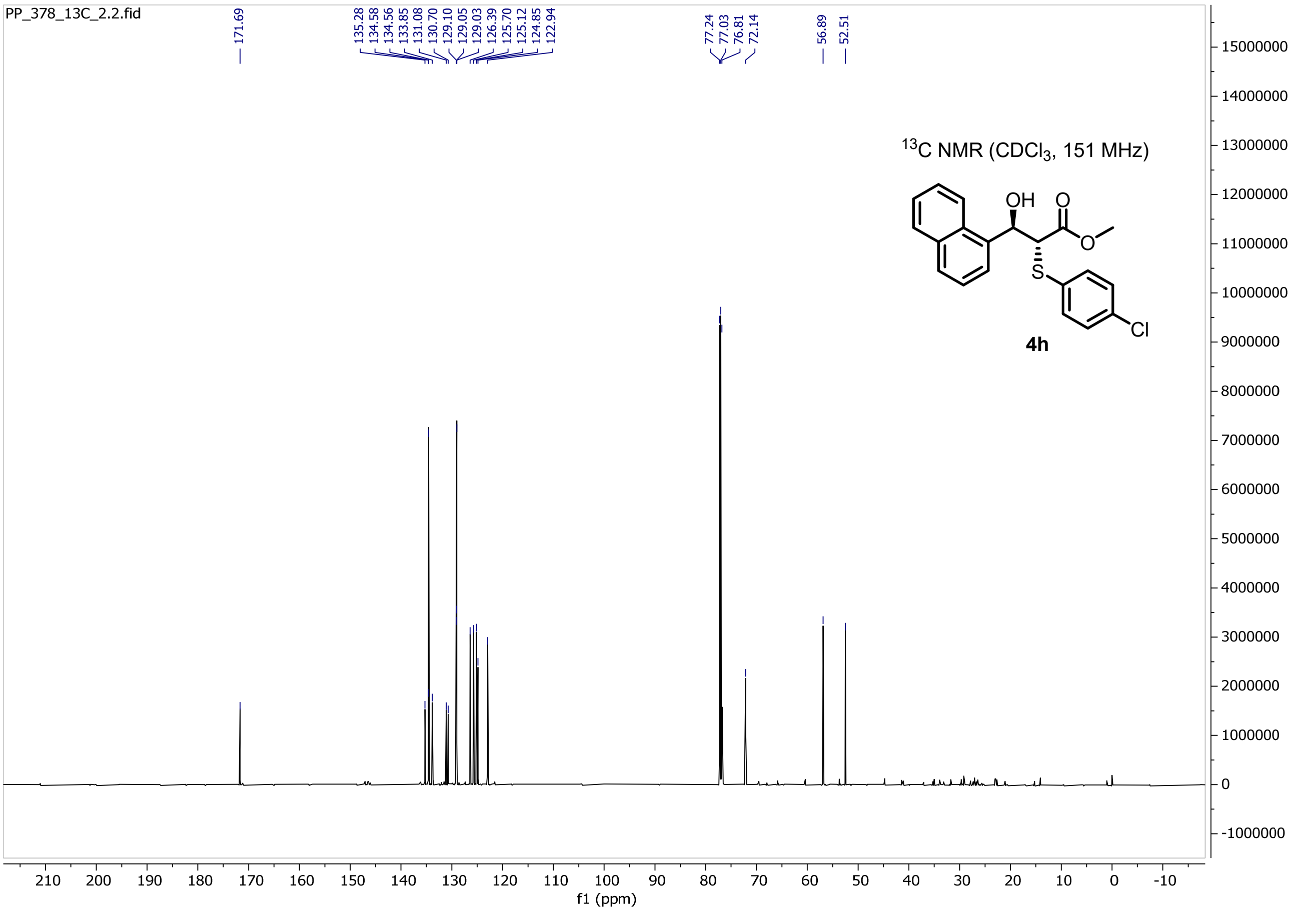
PP_378_13C_2.2.fid
171.69
135.28
134.58
134.56
133.85
131.08
130.70
129.10
129.05
129.03
126.39
125.70
125.12
124.85
122.94
77.24
77.03
76.81
72.14
56.89
52.51
13C NMR (CDCl3, 151 MHz)
OH
O
O
S
Cl
4h
f1 (ppm)

CSP HPLC traces:

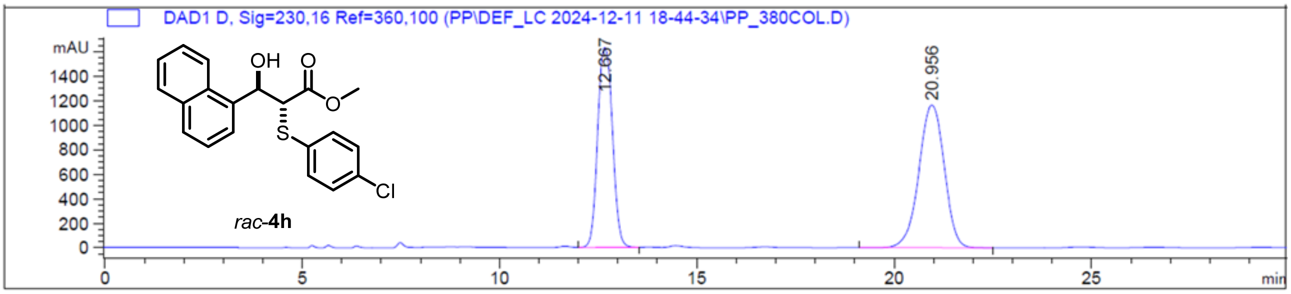

Peak #	RetTime [min]	Type	Width [min]	Area [mAU*s]	Height [mAU]	Area %
1	12.666	BB	0.3281	7472.20947	351.42935	49.8877
2	20.956	BB	0.6395	7505.86035	181.86192	50.1123

Totals : 1.49781e4 533.29128

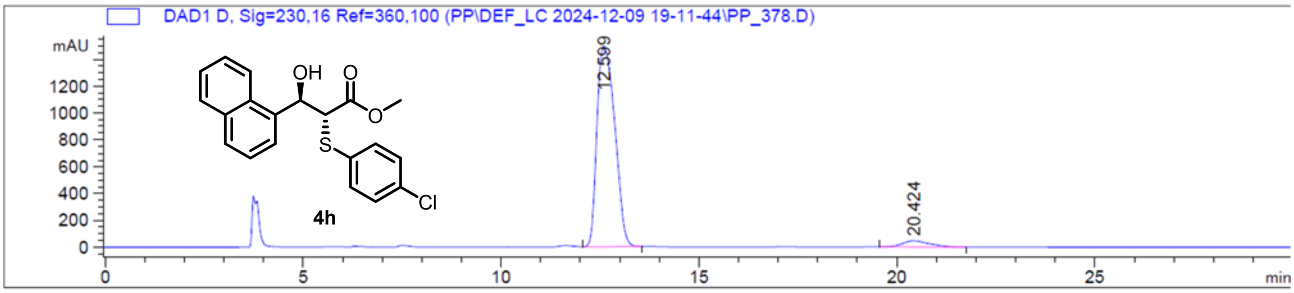

Signal 3: DAD1 D, Sig=230,16 Ref=360,100

Peak #	RetTime [min]	Type	Width [min]	Area [mAU*s]	Height [mAU]	Area %
1	12.599	VB	0.5253	4.68519e4	1496.45154	95.5410
2	20.424	BB	0.6973	2186.63257	46.43828	4.4590

Totals : 4.90385e4 1542.88982

4i- Methyl (2*R*,3*R*)-2-((4-chlorophenyl)thio)-3-(4-fluorophenyl)-3-hydroxypropanoate

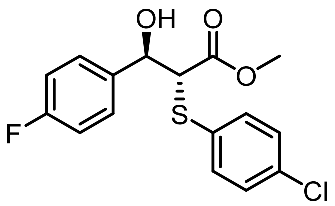

$C_{16}H_{14}ClFO_3S$

MW: 340.8 g/mol

Yield: 82%;

Colorless oil

The product (**4i**) was prepared using the general procedure **B** from **1a** (methyl 2-(dimethyl(oxo)-λ^6-sulfaneylidene)acetate), **2i** (4-fluorobenzaldehyde), and **3a** (4-chlorobenzenethiol). The product was obtained as a colorless oil (28 mg; 82% yield). The diastereomeric ratio obtained for the *anti*-couple is greater than 20:1. The enantiomeric excess of the product was determined by CSP HPLC: AD-H column, *n*-hexane/iPrOH 80:20, 0.75 mL/min, t_{maj} = 17.4 min; t_{min} = 12.2 min, 93% ee.

^{1}H NMR (600 MHz, $CDCl_3$) δ 7.35 – 7.29 (m, 2H), 7.24 – 7.15 (m, 4H), 7.06 – 6.99 (m, 2H), 4.98 (d, *J* = 8.3 Hz, 1H), 3.81 (d, *J* = 8.3 Hz, 1H), 3.69 (s, 3H), 3.14 (br s, 1H).

^{13}C NMR (151 MHz, $CDCl_3$) δ 171.7, 162.7 (d, *J* = 247.3 Hz), 135.8 (d, *J* = 3.2 Hz), 134.8, 134.6, 131.2, 129.2, 128.6 (d, *J* = 8.2 Hz), 115.4 (d, *J* = 21.7 Hz), 73.8, 57.9, 52.6.

^{19}F NMR (376 MHz, $CDCl_3$) δ -113.12 – -113.79 (m, 1F).

HRMS (MALDI$^+$) m/z calcd for $C_{16}H_{14}ClFO_3SNa^+$[M+Na]$^+$: 363.0228, found: 363.0234.

$[\alpha]_D^{RT}$; RT = +50.3 (c = 1.0 g/100 mL, $CHCl_3$).

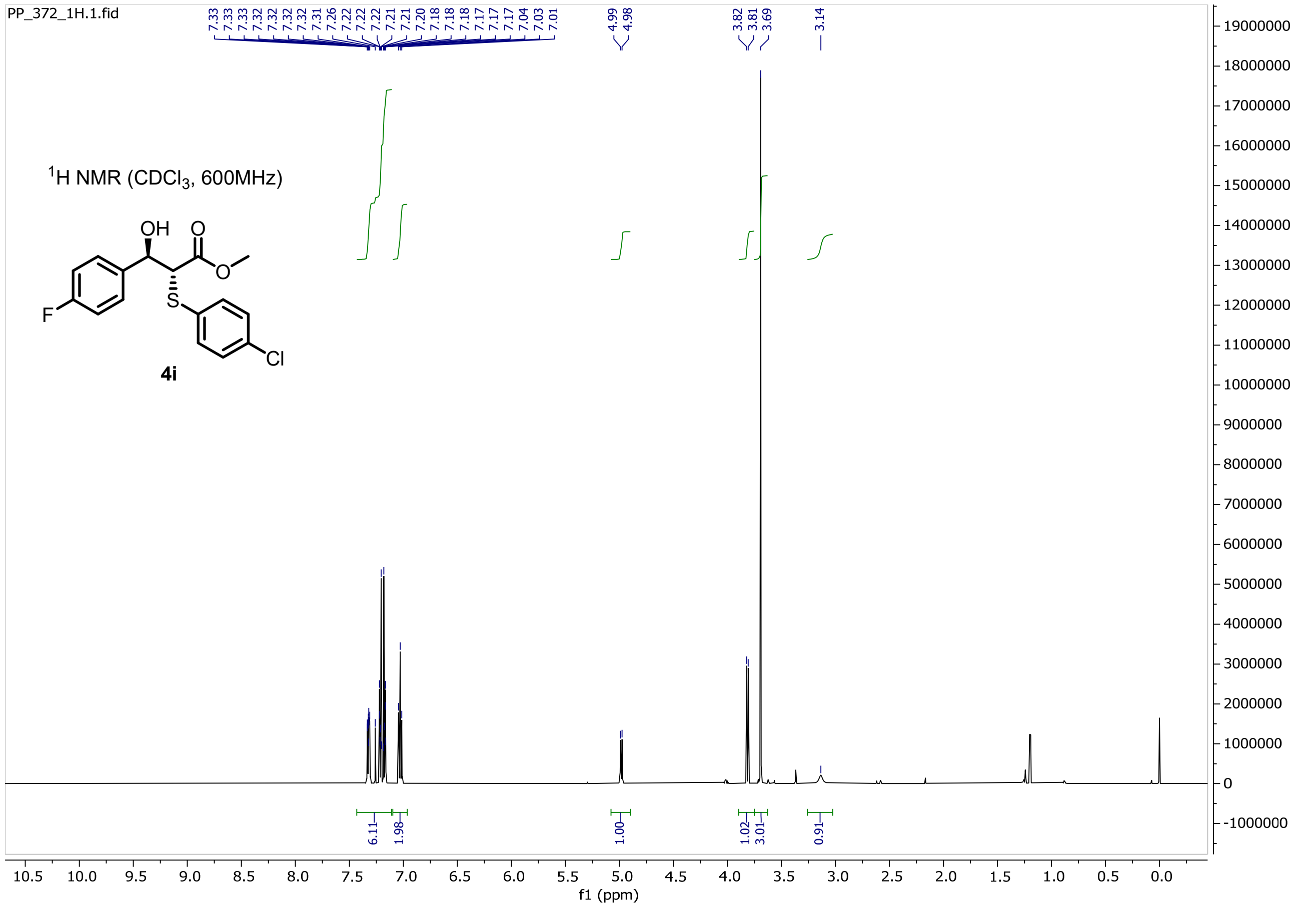
PP_372_1H.1.fid
7.33 7.33 7.33 7.32 7.32 7.32 7.32 7.31 7.26 7.22 7.22 7.22 7.21 7.21 7.20 7.18 7.18 7.18 7.17 7.17 7.17 7.04 7.03 7.01
4.99 4.98
3.82 3.81 3.69
3.14
^{1}H NMR (CDCl$_3$, 600MHz)
OH
O
O
F
S
Cl
4i
6.11
1.98
1.00
1.02
3.01
0.91
f1 (ppm)

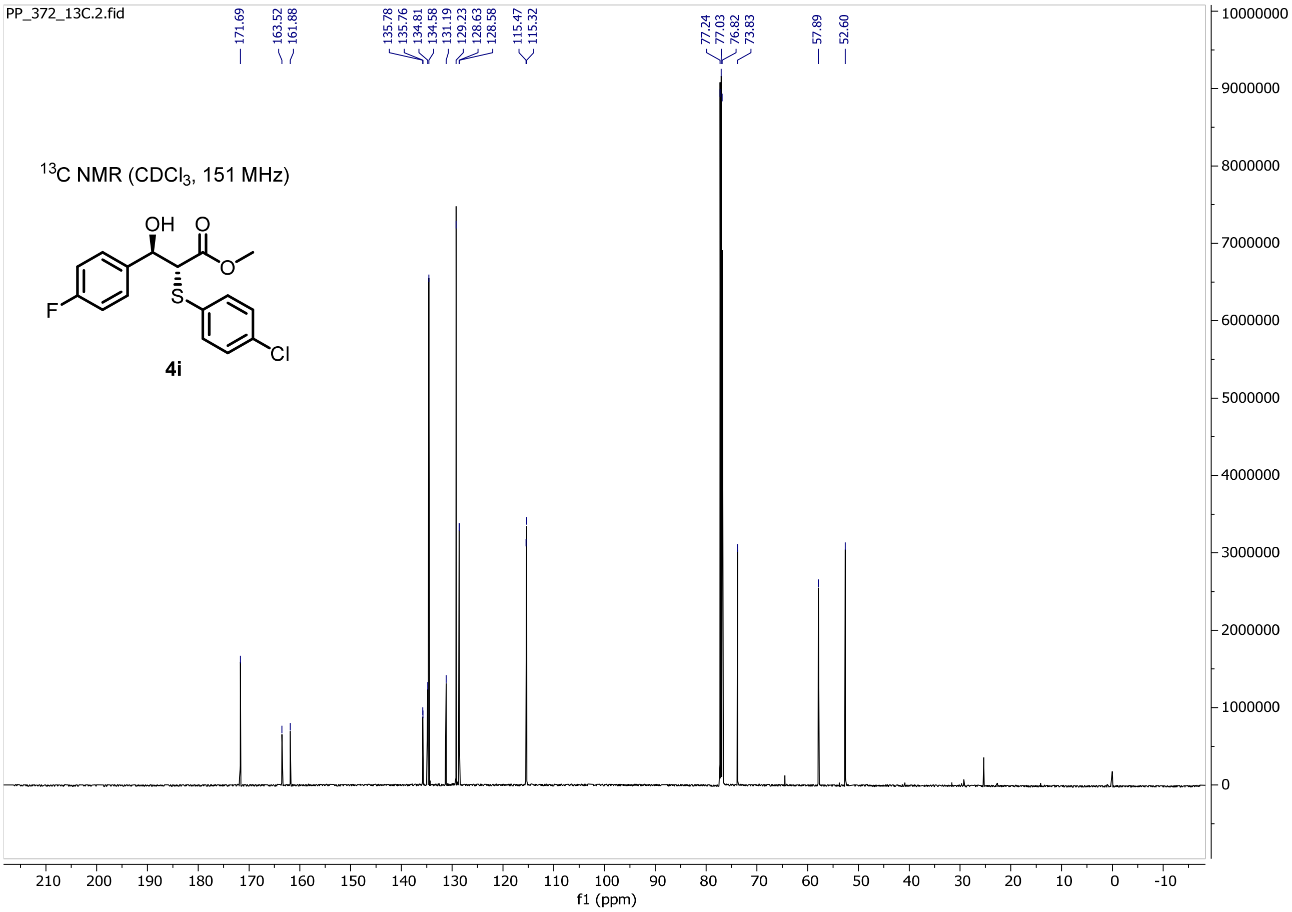
PP_372_13C.2.fid
171.69
163.52 161.88
135.78 135.76 134.81 134.58 131.19 129.23 128.63 128.58
115.47 115.32
77.24 77.03 76.82 73.83
57.89
52.60
^{13}C NMR (CDCl$_3$, 151 MHz)
OH
O
O
F
S
Cl
4i
f1 (ppm)

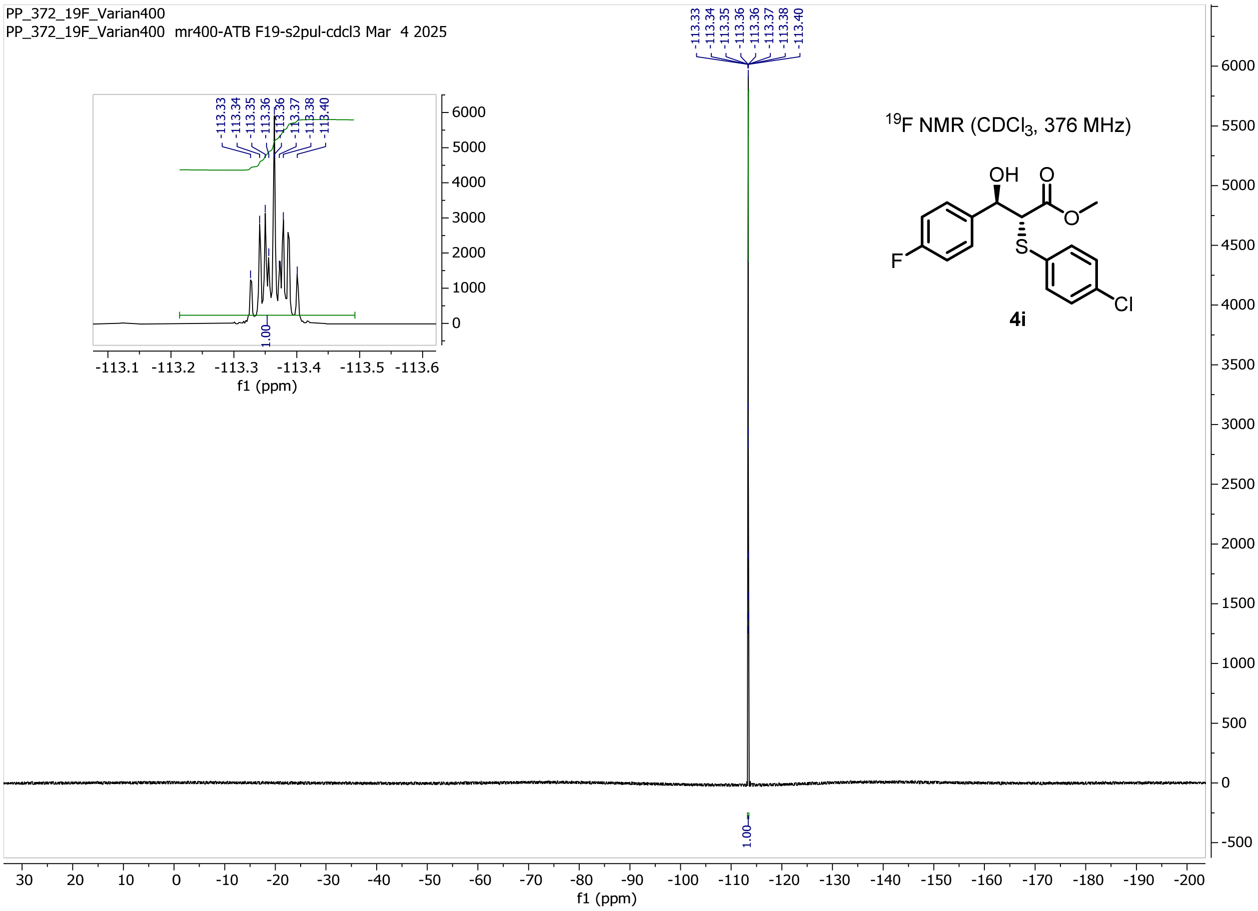
PP_372_19F_Varian400
PP_372_19F_Varian400 mr400-ATB F19-s2pul-cdcl3 Mar 4 2025
-113.33
-113.34
-113.35
-113.36
-113.36
-113.37
-113.38
-113.40
^{19}F NMR (CDCl$_3$, 376 MHz)
OH
O
O
F
S
Cl
4i
1.00
f1 (ppm)

CSP HPLC traces:

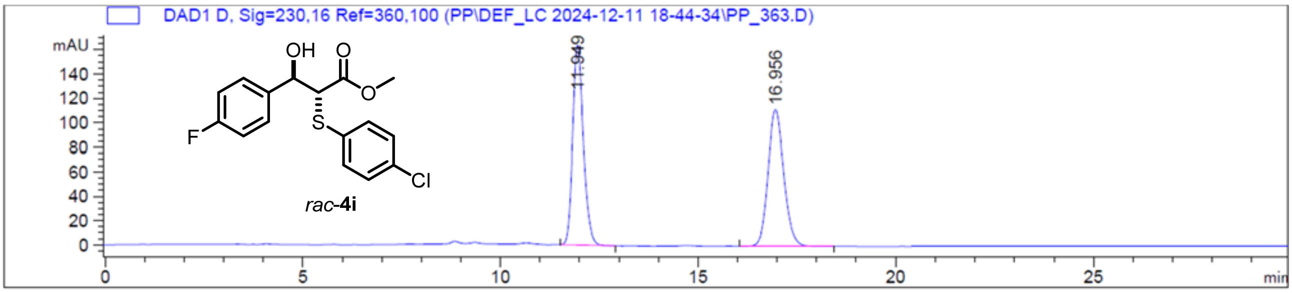

Signal 3: DAD1 D, Sig=230,16 Ref=360,100

Peak #	RetTime [min]	Type	Width [min]	Area [mAU*s]	Height [mAU]	Area %
1	11.949	BB	0.2860	3061.06372	163.83466	49.7767
2	16.956	BB	0.4260	3088.52417	111.89878	50.2233
Totals :				6149.58789	275.73344	

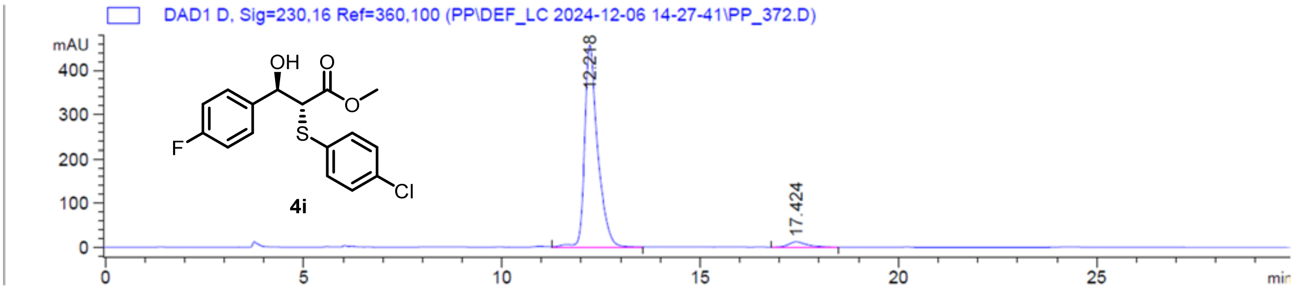

Signal 3: DAD1 D, Sig=230,16 Ref=360,100

Peak #	RetTime [min]	Type	Width [min]	Area [mAU*s]	Height [mAU]	Area %
1	12.218	BB	0.3331	1.03833e4	456.98035	96.4427
2	17.424	BB	0.4714	382.99390	11.96504	3.5573
Totals :				1.07663e4	468.94539	

4j- Methyl (2*R*,3*R*)-3-(4-chlorophenyl)-2-((4-chlorophenyl)thio)-3-hydroxypropanoate

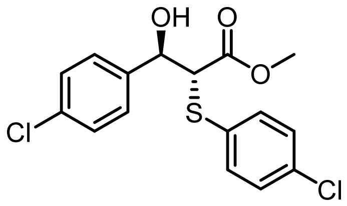

$C_{16}H_{14}Cl_2O_3S$

MW: 357.2 g/mol

Yield: 77%;

Colorless oil

The product (**4j**) was prepared using the general procedure **B** from **1a** (methyl 2-(dimethyl(oxo)-λ^6-sulfaneylidene)acetate), **2j** (4-chlorobenzaldehyde), and **3a** (4-chlorobenzenethiol). The product was obtained as a colorless oil (28 mg; 77% yield). The diastereomeric ratio obtained for the *anti*-couple is greater than 20:1. The enantiomeric excess of the product was determined by CSP HPLC: AD-H column, *n*-hexane/iPrOH 80:20, 0.75 mL/min, t_{maj} = 13.4 min; t_{min} = 21.3 min, 87% ee.

^{1}H NMR (600 MHz, $CDCl_3$) δ 7.33 – 7.27 (m, 4H), 7.24 – 7.17 (m, 4H), 4.98 (d, *J* = 8.0 Hz, 1H), 3.81 (d, *J* = 8.1 Hz, 1H), 3.69 (s, 3H).

^{13}C NMR (151 MHz, $CDCl_3$) δ 171.6, 138.4, 134.8, 134.6, 134.3, 131.0, 129.2, 128.6, 128.2, 73.8, 57.6, 52.6.

HRMS (MALDI$^+$) m/z calcd for $C_{16}H_{14}Cl_2O_3SNa^+$ $[M+Na]^+$: 378.9933, found: 378.9938.

$[\alpha]_D^{RT}$ = +22.1 (c = 1.0 g/100 mL, $CHCl_3$).

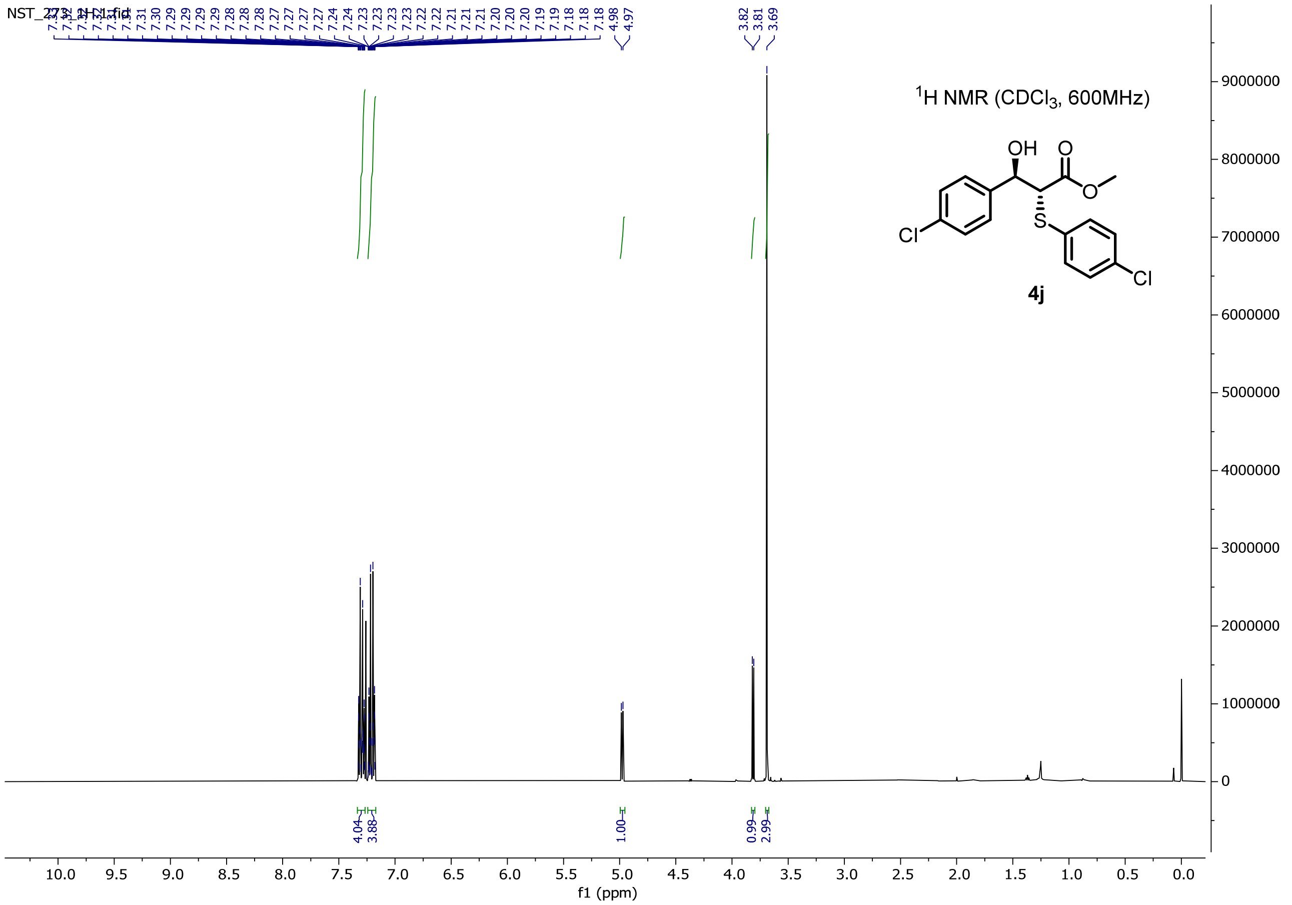

^{1}H NMR (CDCl$_3$, 600MHz)
OH
O
Cl
S
Cl
4j
4.98
4.97
3.82
3.81
3.69
4.04
3.88
1.00
0.99
2.99
f1 (ppm)

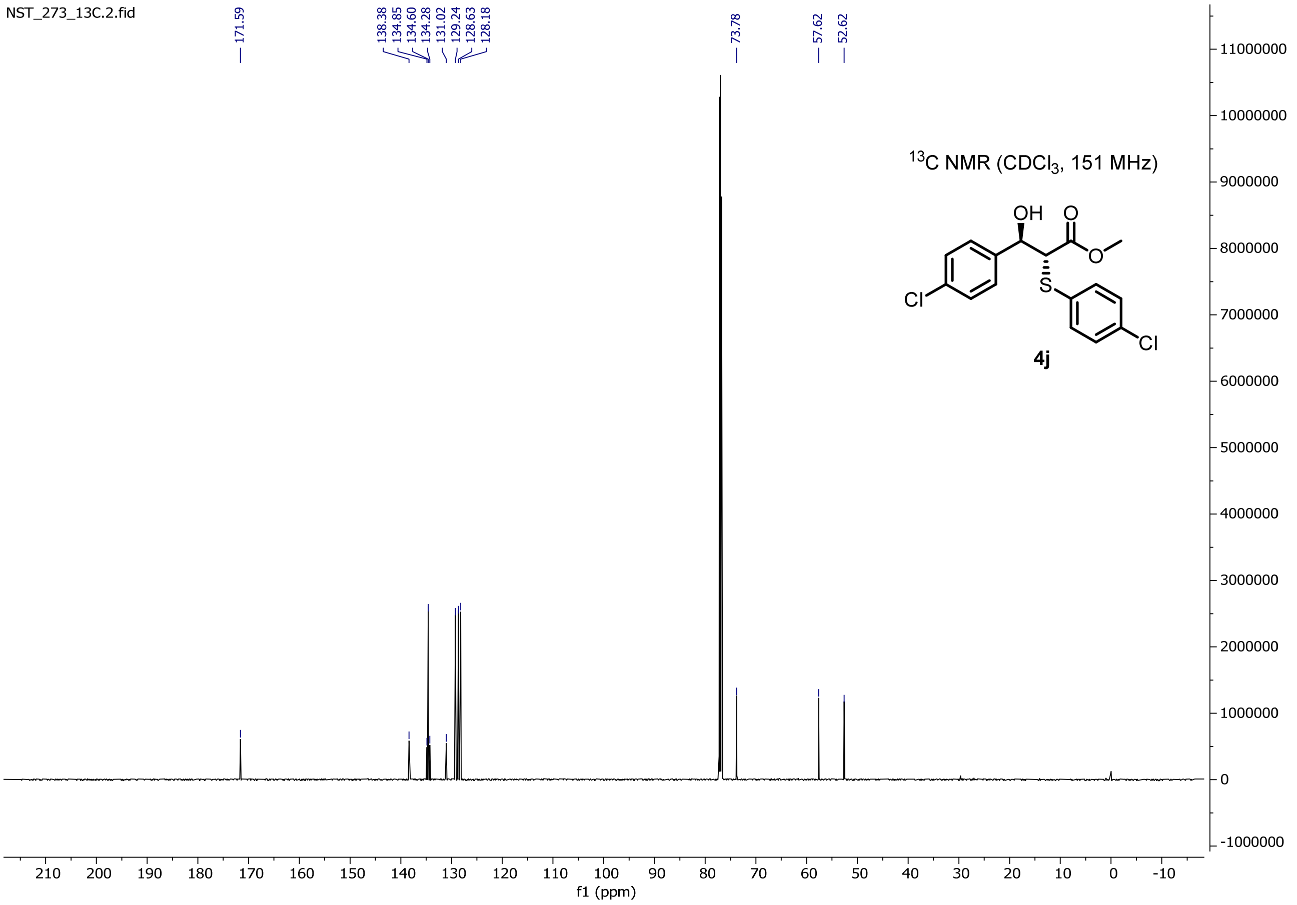

NST_273_13C.2.fid
171.59
138.38
134.85
134.60
134.28
131.02
129.24
128.63
128.18
73.78
57.62
52.62
^{13}C NMR (CDCl$_3$, 151 MHz)
OH
O
Cl
S
Cl
4j
f1 (ppm)

CSP HPLC traces:

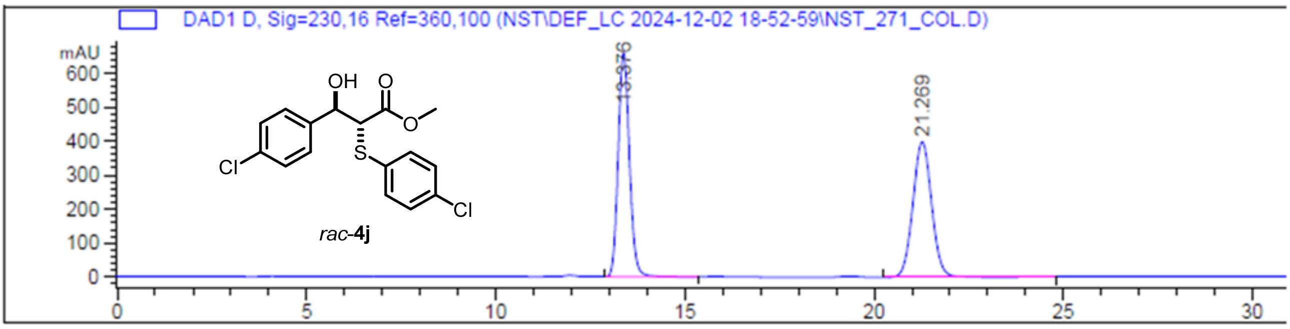

Signal 3: DAD1 D, Sig=230,16 Ref=360,100

Peak #	RetTime [min]	Type	Width [min]	Area [mAU*s]	Height [mAU]	Area %
1	13.376	VB	0.3194	1.36657e4	660.41022	49.8168
2	21.269	BB	0.5370	1.37662e4	398.49814	50.1832

Totals : 2.74320e4 1058.90836

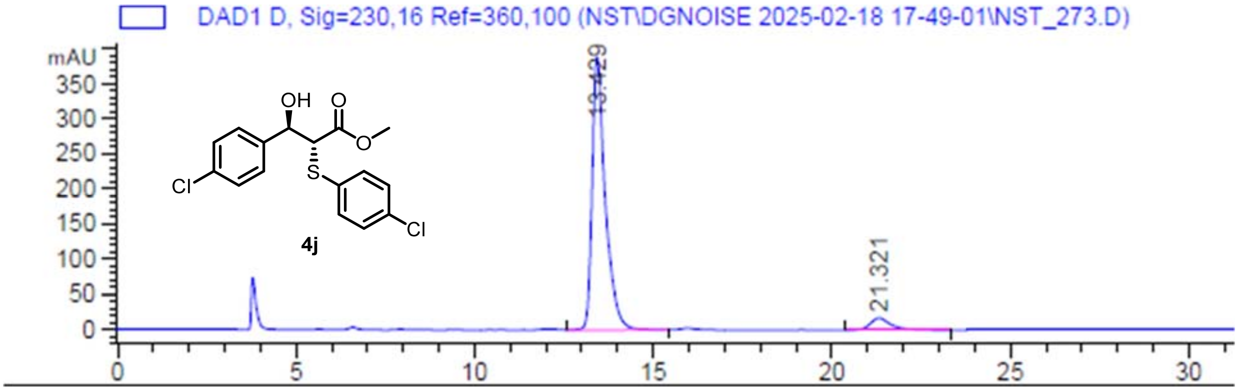

Signal 3: DAD1 D, Sig=230,16 Ref=360,100

Peak #	RetTime [min]	Type	Width [min]	Area [mAU*s]	Height [mAU]	Area %
1	13.429	BB	0.3715	9897.87500	388.15356	93.3636
2	21.321	BB	0.6024	703.55035	17.15710	6.6364

Totals : 1.06014e4 405.31067

4k- Methyl (2*R*,3*R*)-3-(4-bromophenyl)-2-((4-chlorophenyl)thio)-3-hydroxypropanoate

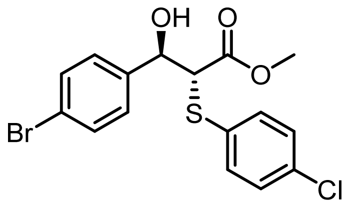

$C_{16}H_{14}ClBrO_3S$

MW: 401.7 g/mol

Yield: 72%;

White solid

The product (**4k**) was prepared using the general procedure **B** from **1a** (methyl 2-(dimethyl(oxo)-λ^6-sulfaneylidene)acetate), **2k** (4-bromobenzaldehyde), and **3a** (4-chlorobenzenethiol). The product was obtained as a white solid (29 mg; 72% yield). The diastereomeric ratio obtained for the *anti*-couple is greater than 20:1. The enantiomeric excess of the product was determined by CSP HPLC: AD-H column, *n*-hexane/iPrOH 80:20, 0.75 mL/min, t_{maj} = 14.3 min; t_{min} = 24.3 min, 88% ee.

^{1}H NMR (600 MHz, $CDCl_3$) δ 7.46 (d, *J* = 8.5 Hz, 2H), 7.23 – 7.17 (m, 6H), 4.95 (d, *J* = 8.1 Hz, 1H), 3.81 (d, *J* = 8.0 Hz, 1H), 3.68 (s, 3H).

^{13}C NMR (151 MHz, $CDCl_3$) δ 171.6, 138.9, 134.8, 134.6, 131.6, 131.1, 129.2, 128.5, 122.5, 73.9, 57.6, 52.6.

HRMS (MALDI$^+$) m/z calcd for $C_{16}H_{14}ClBrO_3SNa^+$ $[M+Na]^+$: 422.9428, found: 422.9431.

$[\alpha]_D^{RT}$ = +21.2 (c = 1.0 g/100 mL, $CHCl_3$).

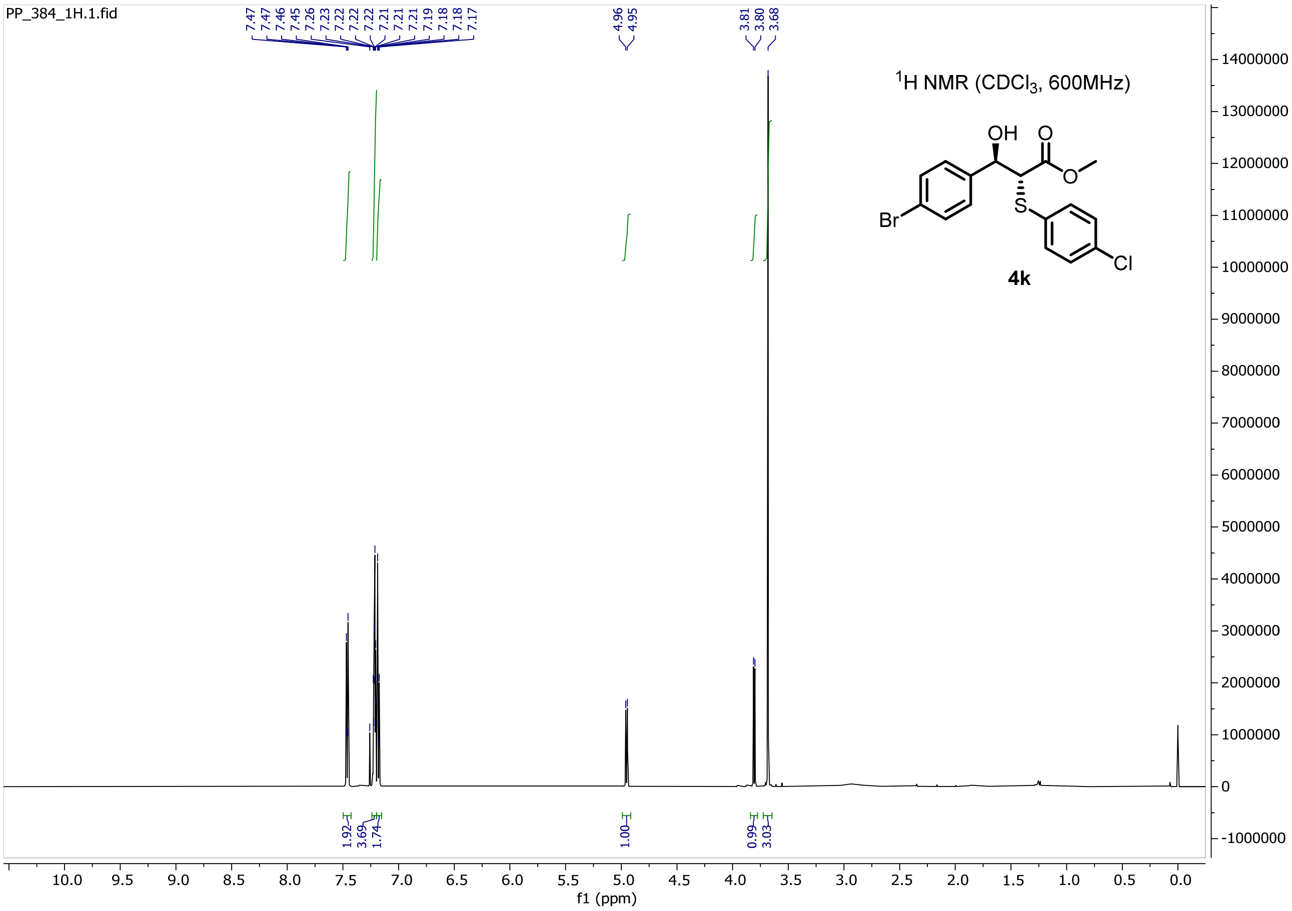
PP_384_1H.1.fid
7.47
7.47
7.46
7.45
7.26
7.23
7.22
7.22
7.21
7.21
7.21
7.19
7.18
7.18
7.17
4.96
4.95
3.81
3.80
3.68
^{1}H NMR (CDCl$_3$, 600MHz)
OH
O
O
S
Br
Cl
4k
1.92
3.69
1.74
1.00
0.99
3.03
f1 (ppm)

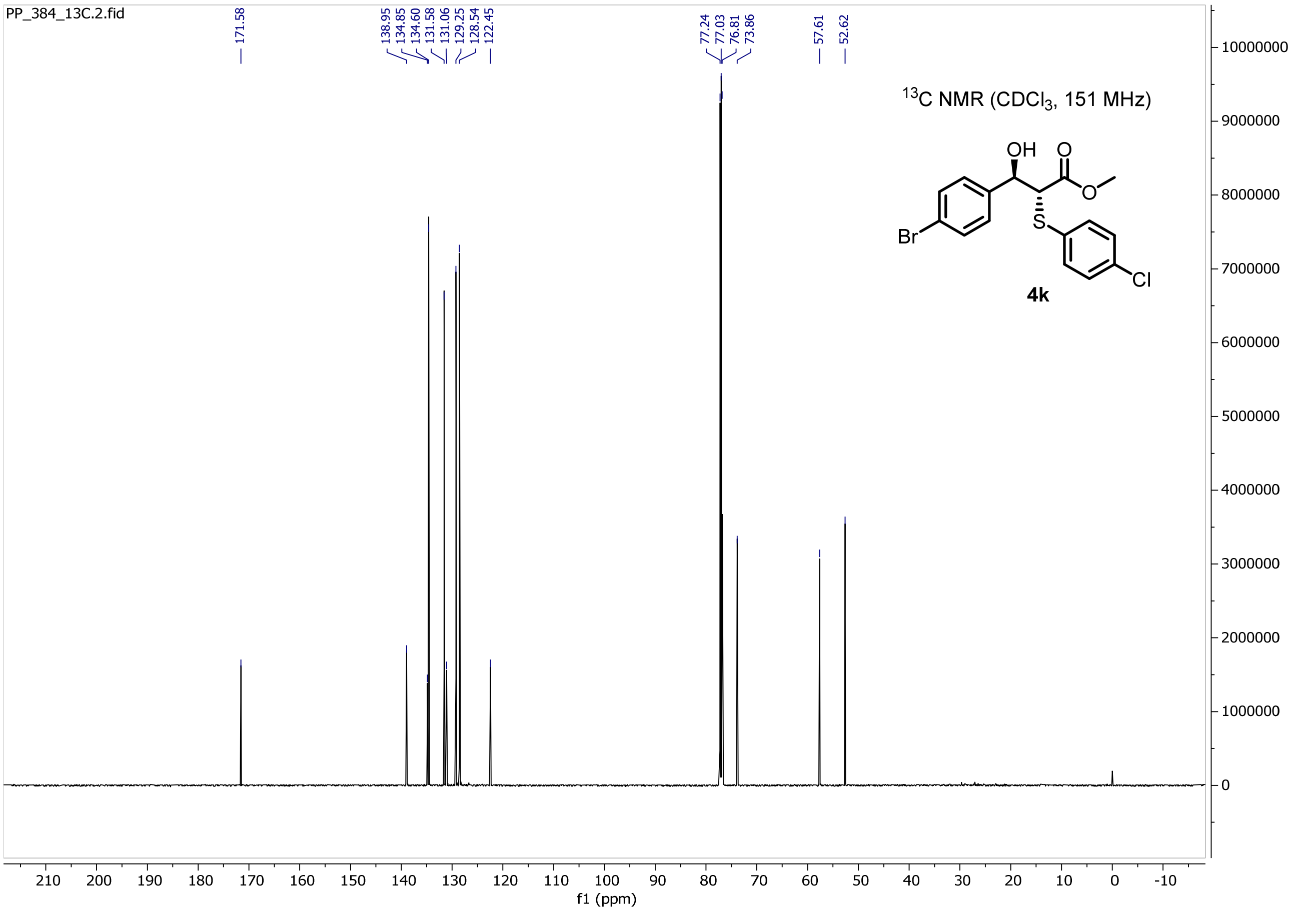
PP_384_13C.2.fid
171.58
138.95
134.85
134.60
131.58
131.06
129.25
128.54
122.45
77.24
77.03
76.81
73.86
57.61
52.62
^{13}C NMR (CDCl$_3$, 151 MHz)
OH
O
O
S
Br
Cl
4k
f1 (ppm)

CSP HPLC traces:

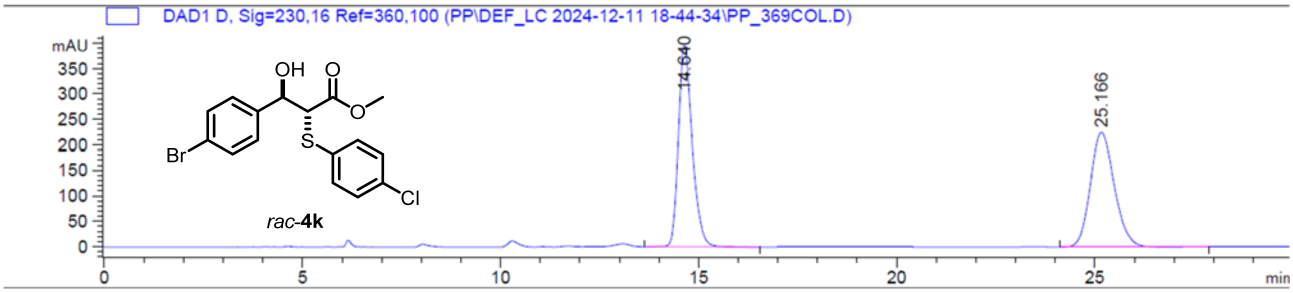

Signal 3: DAD1 D, Sig=230,16 Ref=360,100

Peak #	RetTime [min]	Type	Width [min]	Area [mAU*s]	Height [mAU]	Area %
1	14.640	BB	0.3701	9626.18945	395.32007	50.3698
2	25.166	BB	0.6472	9484.84277	225.27524	49.6302

Totals : 1.91110e4 620.59531

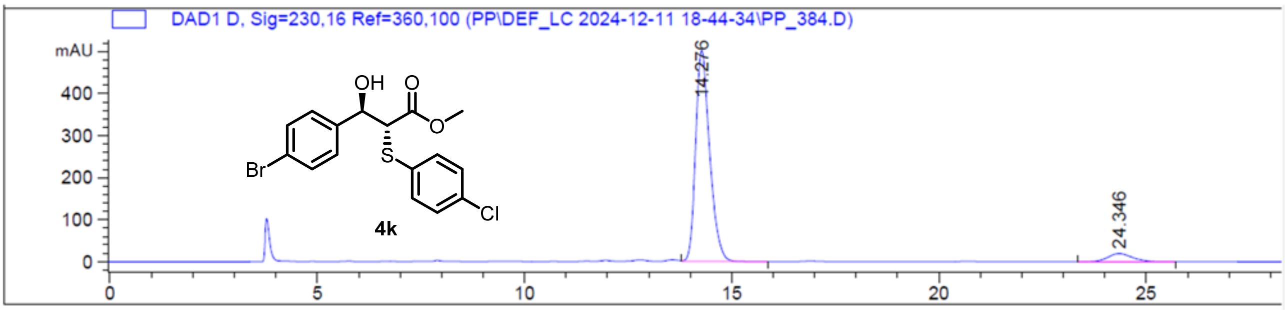

Signal 3: DAD1 D, Sig=230,16 Ref=360,100

Peak #	RetTime [min]	Type	Width [min]	Area [mAU*s]	Height [mAU]	Area %
1	14.276	VB	0.3580	1.17973e4	502.23886	93.7989
2	24.346	BB	0.6222	779.93500	19.18830	6.2011

Totals : 1.25773e4 521.42716

4l- Methyl (2*R*,3*R*)-2-((4-chlorophenyl)thio)-3-hydroxy-3-(4-iodophenyl)propanoate

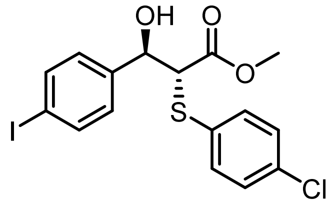

$C_{16}H_{14}ClO_3SI$

MW: 448.7 g/mol

Yield: 53%;

White solid

The product (**4l**) was prepared using the general procedure **B** from **1a** (methyl 2-(dimethyl(oxo)-λ^6-sulfaneylidene)acetate), **2l** (4-iodobenzaldehyde), and **3a** (4-chlorobenzenethiol). The product was obtained as a white solid (24 mg; 53% yield). The diastereomeric ratio obtained for the *anti*-couple is greater than 20:1. The enantiomeric excess of the product was determined by CSP HPLC: AD-H column, *n*-hexane/iPrOH 80:20, 0.75 mL/min, t_{maj} = 16.5 min; t_{min} = 32.9 min, 87% ee.

^{1}H NMR (600 MHz, $CDCl_3$) δ 7.66 (d, *J* = 8.4 Hz, 2H), 7.24 – 7.16 (m, 4H), 7.10 – 7.06 (m, 2H), 4.94 (d, *J* = 8.1 Hz, 1H), 3.80 (d, *J* = 8.1 Hz, 1H), 3.68 (s, 3H), 3.15 (br s, 1H).

^{13}C NMR (151 MHz, $CDCl_3$) δ 171.5, 139.6, 137.5, 134.8, 134.6, 131.1, 129.3, 128.7, 94.2, 74.0, 57.6, 52.6.

HRMS (MALDI$^+$) m/z calcd for $C_{16}H_{14}ClIO_3SNa^+$[M+Na]$^+$: 470.9289, found 470.9294.

$[\alpha]_D^{RT}$ = +13.5 (c = 1.0 g/100 mL, $CHCl_3$).

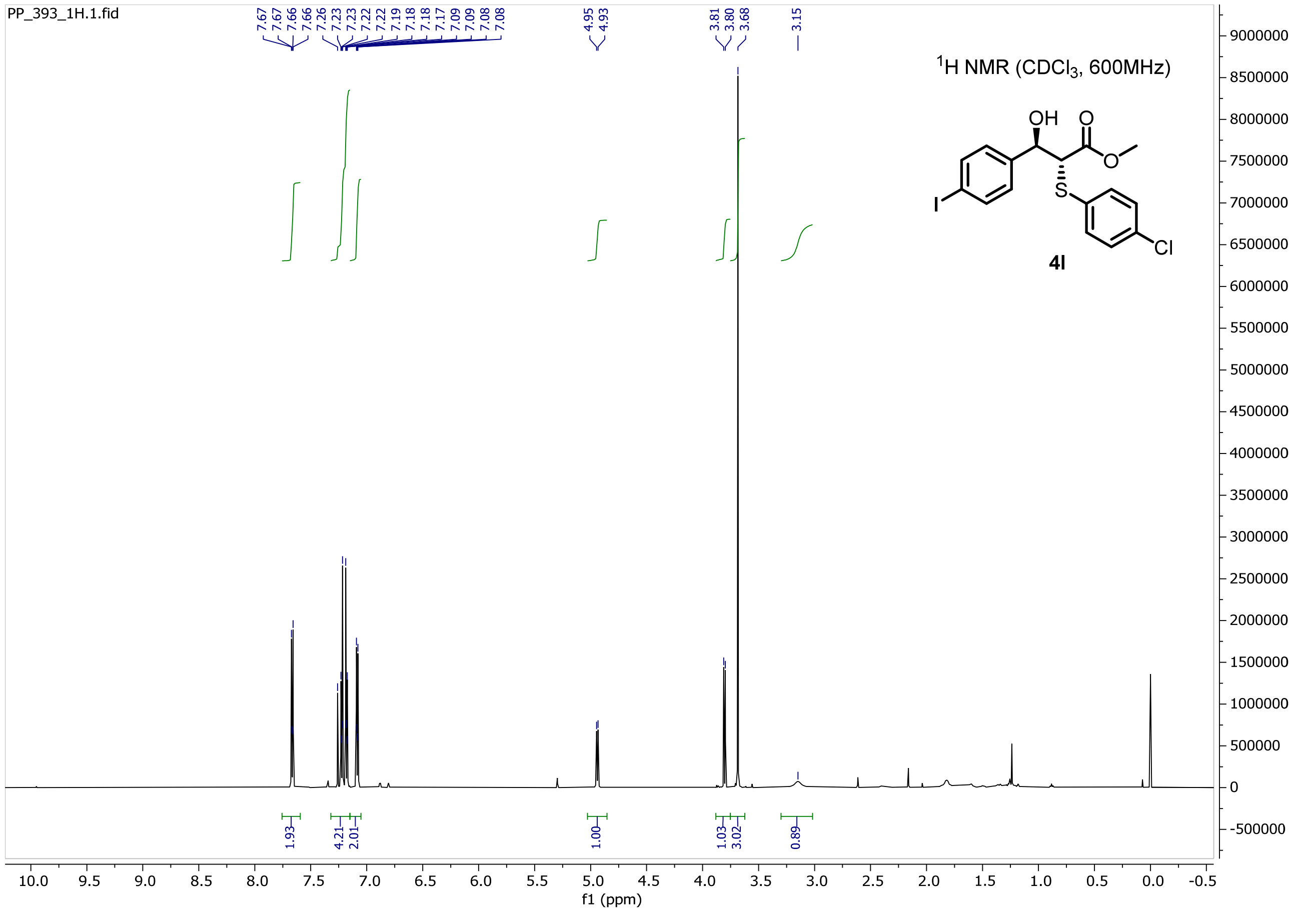
PP_393_1H.1.fid
7.67
7.67
7.66
7.66
7.26
7.23
7.23
7.22
7.22
7.19
7.18
7.18
7.17
7.09
7.09
7.08
7.08
4.95
4.93
3.81
3.80
3.68
3.15
1H NMR (CDCl3, 600MHz)
OH
O
O
I
S
Cl
4l
1.93
4.21
2.01
1.00
1.03
3.02
0.89
f1 (ppm)

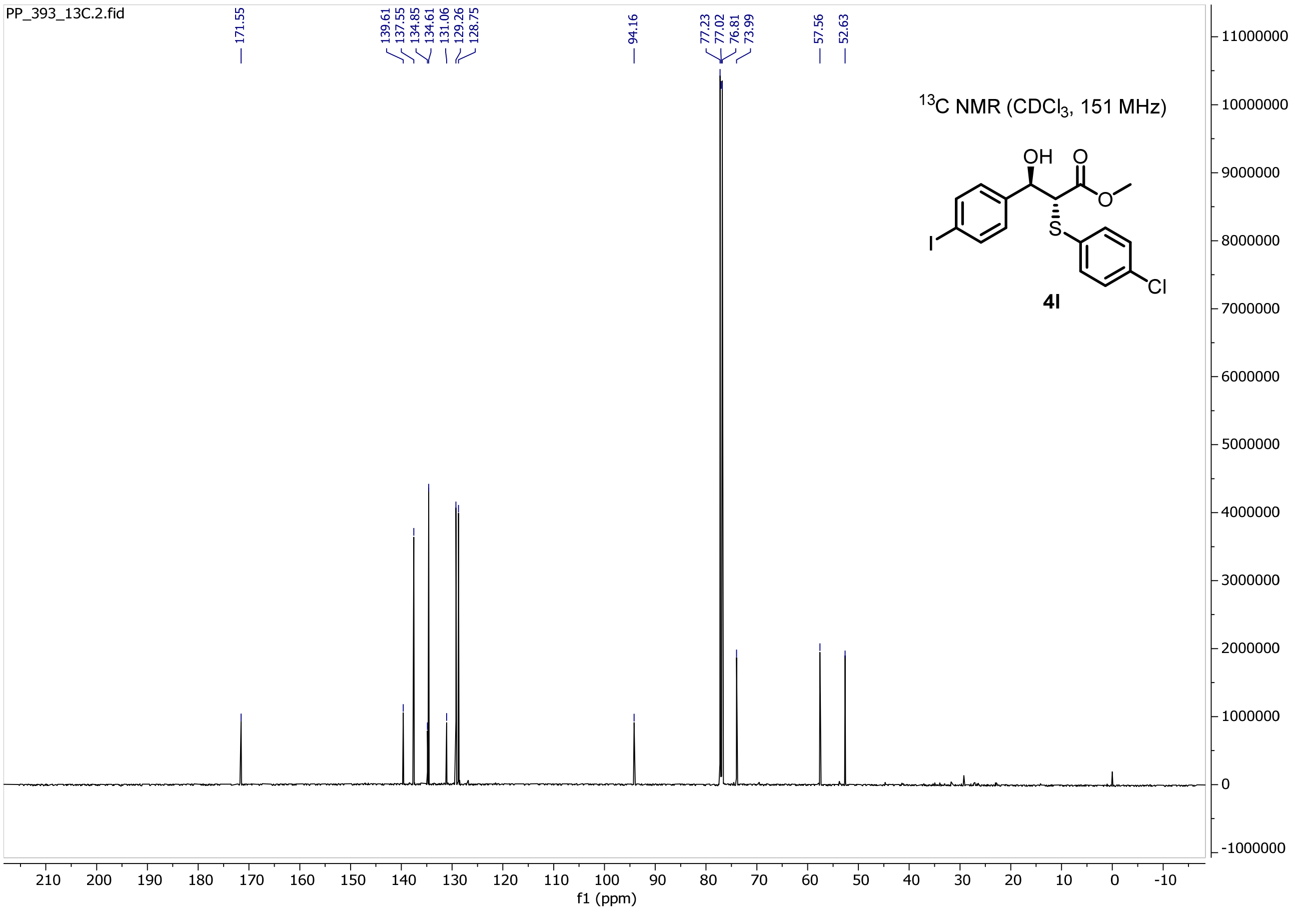
PP_393_13C.2.fid
171.55
139.61
137.55
134.85
134.61
131.06
129.26
128.75
94.16
77.23
77.02
76.81
73.99
57.56
52.63
13C NMR (CDCl3, 151 MHz)
OH
O
O
I
S
Cl
4l
f1 (ppm)

CSP HPLC traces:

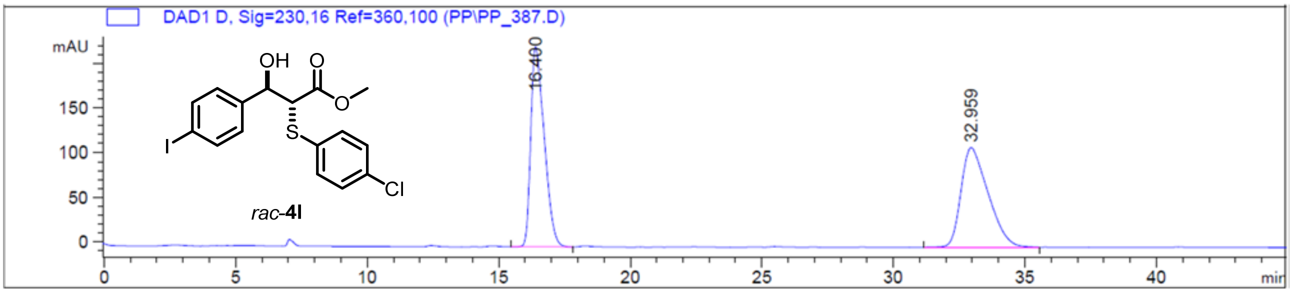

Signal 3: DAD1 D, Sig=230,16 Ref=360,100

Peak #	RetTime [min]	Type	Width [min]	Area [mAU*s]	Height [mAU]	Area %
1	16.400	BB	0.5198	7952.78906	224.19821	49.9217
2	32.959	BB	1.0621	7977.73047	111.05025	50.0783

Totals : 1.59305e4 335.24847

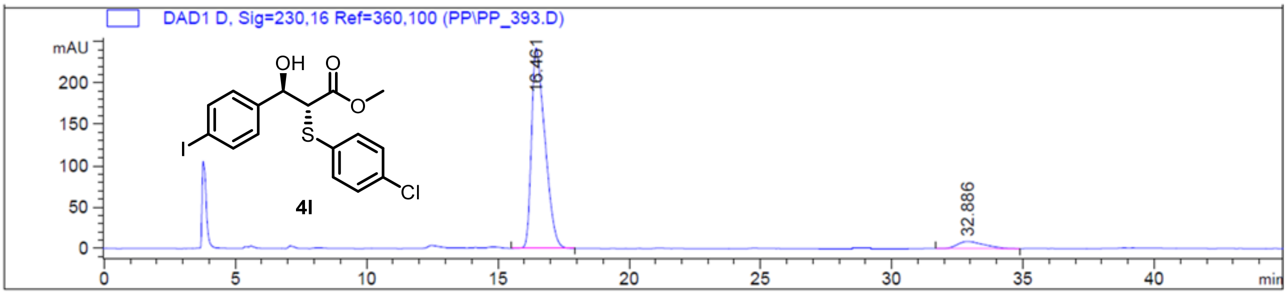

Signal 3: DAD1 D, Sig=230,16 Ref=360,100

Peak #	RetTime [min]	Type	Width [min]	Area [mAU*s]	Height [mAU]	Area %
1	16.461	BB	0.5095	8577.51953	242.01067	93.4479
2	32.886	BB	0.9849	601.41351	8.40361	6.5521

Totals : 9178.93304 250.41427

4m- Methyl (2*R*,3*R*)-2-((4-chlorophenyl)thio)-3-hydroxy-3-(4-(4,4,5,5-tetramethyl-1,3,2-dioxaborolan-2-yl) phenyl)propanoate

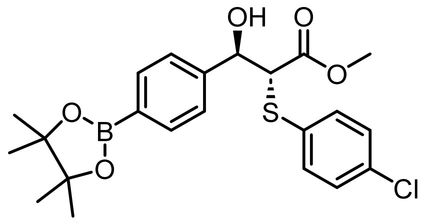

$C_{22}H_{26}BClO_5S$

MW: 448.8 g/mol

Yield 79%;

Colorless oil

The product (**4m**) was prepared using the general procedure **C** from **1a** (methyl 2-(dimethyl(oxo)-λ^6-sulfaneylidene)acetate), **2m** (4-(4,4,5,5-tetramethyl-1,3,2-dioxaborolan-2-yl)benzaldehyde), and **3a** (4-chlorobenzenethiol). The product was obtained as a colorless oil (71 mg; 79% yield). The diastereomeric ratio obtained for the *anti*-couple is greater than 20:1. The enantiomeric excess of the product was determined by CSP HPLC: AD-H column, *n*-hexane/iPrOH 80:20, 0.75 mL/min, t_{maj} = 26.6 min; t_{min} = 29.2 min, 86% ee.

^{1}H NMR (600 MHz, $CDCl_3$) δ 7.78 (d, *J* = 8.1 Hz, 2H), 7.34 (d, *J* = 8.0 Hz, 2H), 7.19 (s, 4H), 5.00 (d, *J* = 7.9 Hz, 1H), 3.89 (d, *J* = 7.9 Hz, 1H), 3.66 (s, 3H), 1.35 (s, 12H).

^{13}C NMR (151 MHz, $CDCl_3$) δ 171.7, 142.9, 134.9, 134.62, 134.58, 131.2, 129.1, 126.0, 83.9, 74.4, 57.4, 52.5, 24.8.

HRMS (MALDI$^+$) m/z calcd for $C_{22}H_{26}BClO_5SNa^+$[M+Na]$^+$: 471.1175, found: 471.1165.

$[\alpha]_D^{RT}$ = +17.2 (c = 1.0 g/100 mL, $CHCl_3$).

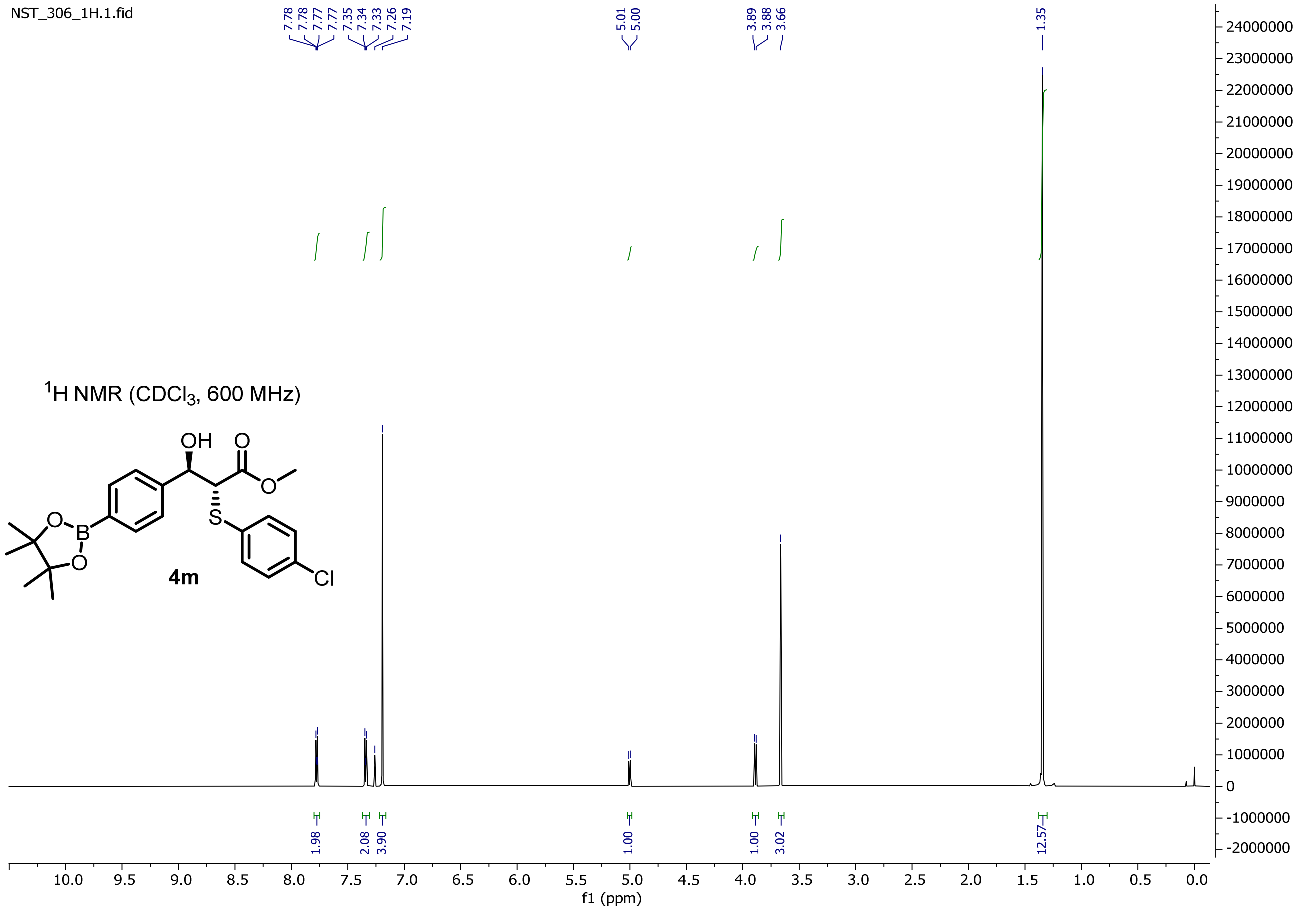
NST_306_1H.1.fid
7.78
7.78
7.77
7.77
7.35
7.34
7.33
7.26
7.19
5.01
5.00
3.89
3.88
3.66
1.35
1H NMR (CDCl3, 600 MHz)
OH
O
O
B
O
O
S
Cl
4m
1.98
2.08
3.90
1.00
1.00
3.02
12.57
f1 (ppm)

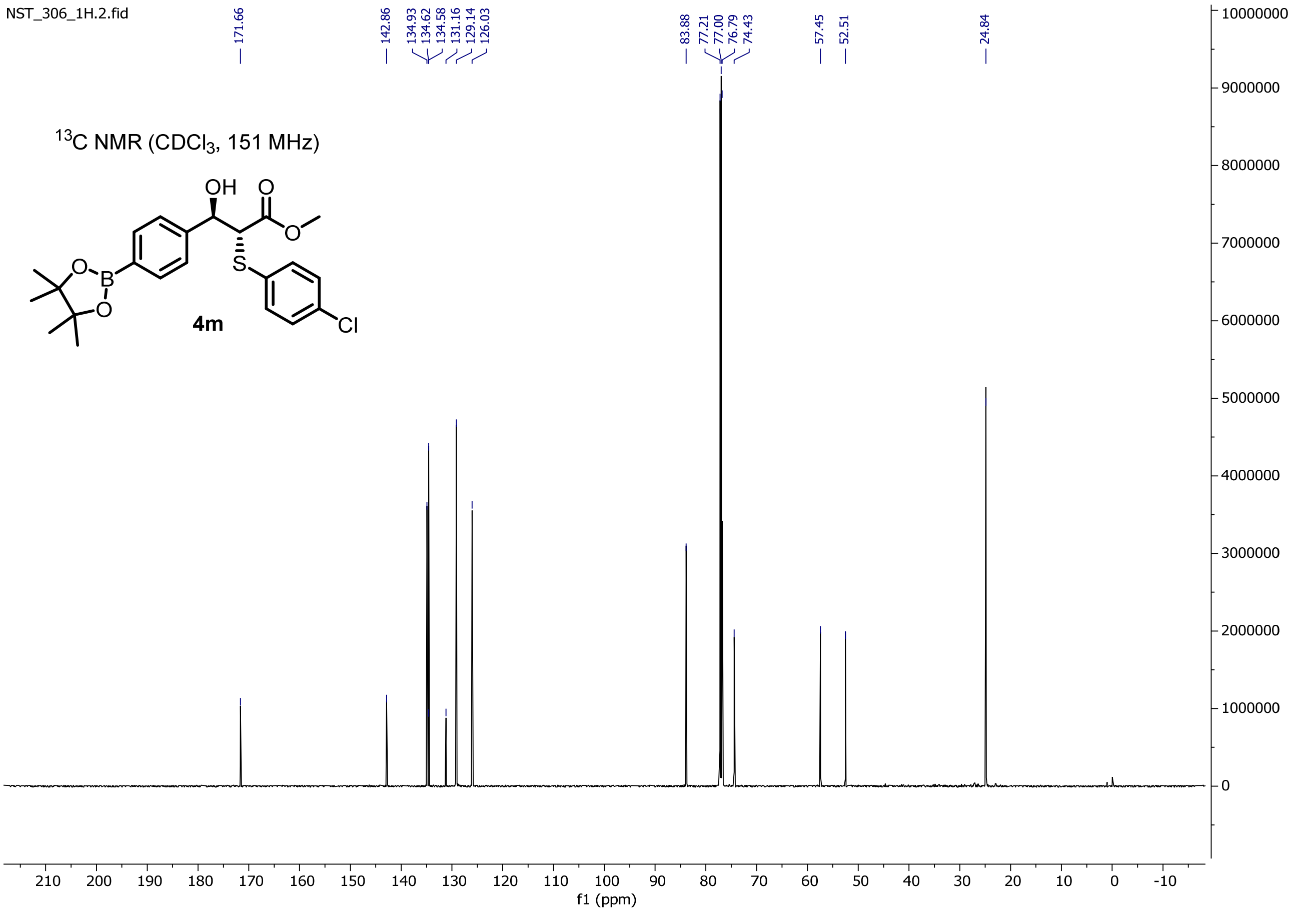
NST_306_1H.2.fid
171.66
142.86
134.93
134.62
134.58
131.16
129.14
126.03
83.88
77.21
77.00
76.79
74.43
57.45
52.51
24.84
13C NMR (CDCl3, 151 MHz)
OH
O
O
B
O
O
S
Cl
4m
f1 (ppm)

CSP HPLC traces:

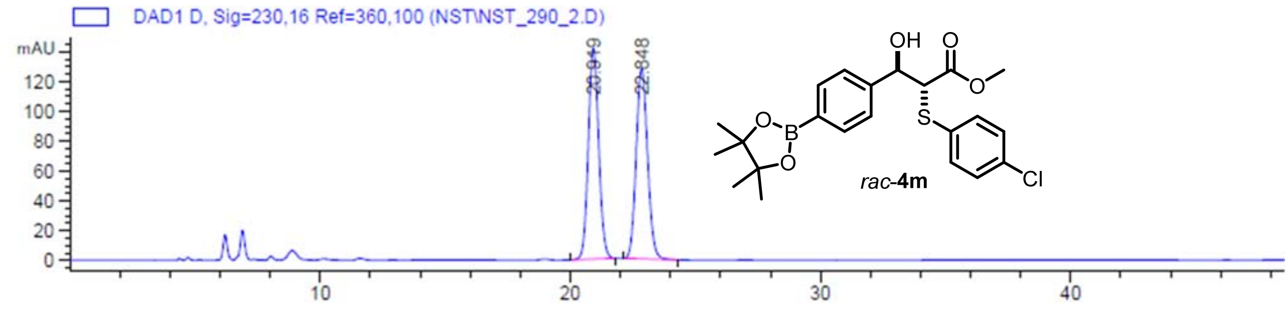

Signal 2: DAD1 D, Sig=230,16 Ref=360,100

Peak #	RetTime [min]	Type	Width [min]	Area [mAU*s]	Height [mAU]	Area %
1	20.919	BB	0.4484	4088.13550	141.79567	50.2943
2	22.848	BB	0.4934	4040.29932	127.58797	49.7057

Totals : 8128.43481 269.38364

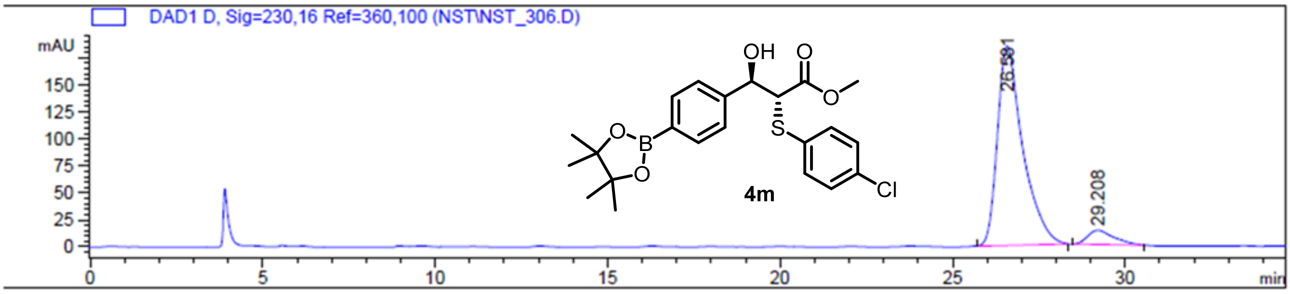

Signal 4: DAD1 D, Sig=230,16 Ref=360,100

Peak #	RetTime [min]	Type	Width [min]	Area [mAU*s]	Height [mAU]	Area %
1	26.581	BB	0.7493	9446.55273	184.51065	93.0649
2	29.208	BB	0.7731	703.94708	13.18089	6.9351

Totals : 1.01505e4 197.69154

4n- Methyl 4-((1*R*,2*R*)-2-((4-chlorophenyl)thio)-1-hydroxy-3-methoxy-3-oxopropyl)benzoate

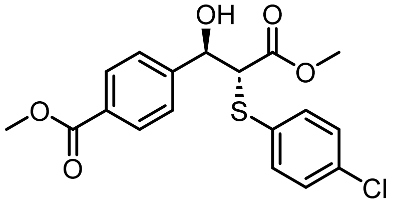

$C_{18}H_{17}ClO_5S$

MW: 380.8 g/mol

Yield: 92%;

Pale yellow solid

The product (**4n**) was prepared using the general procedure **C** from **1a** (methyl 2-(dimethyl(oxo)-λ^6-sulfaneylidene)acetate), **2n** (methyl 4-formylbenzoate), and **3a** (4-chlorobenzenethiol). The product was obtained as a pale yellow solid (35 mg; 92% yield). The diastereomeric ratio obtained for the *anti*-couple is greater than 20:1. The enantiomeric excess of the product was determined by CSP HPLC: AD-H column, *n*-hexane/iPrOH 80:20, 0.75 mL/min, t_{maj} = 19.5 min; t_{min} = 27.5 min, 85% ee.

^{1}H NMR (600 MHz, $CDCl_3$) δ 8.03 – 7.97 (m, 2H), 7.44 – 7.40 (m, 2H), 7.24 – 7.15 (m, 4H), 5.05 (d, *J* = 7.8 Hz, 1H), 3.92 (s, 3H), 3.85 (d, *J* = 7.8 Hz, 1H), 3.67 (s, 3H).

^{13}C NMR (151 MHz, $CDCl_3$) δ 171.5, 166.7, 144.8, 134.8, 134.6, 130.9, 130.1, 129.7, 129.3, 126.8, 73.9, 57.5, 52.6, 52.2.

HRMS (MALDI$^+$) m/z calcd for $C_{20}H_{17}ClO_3SNa^+$ [M+Na]$^+$: 403.0377, found 403.0383.

$[\alpha]_D^{RT}$ = +21.9 (c = 1.0 g/100 mL, $CHCl_3$).

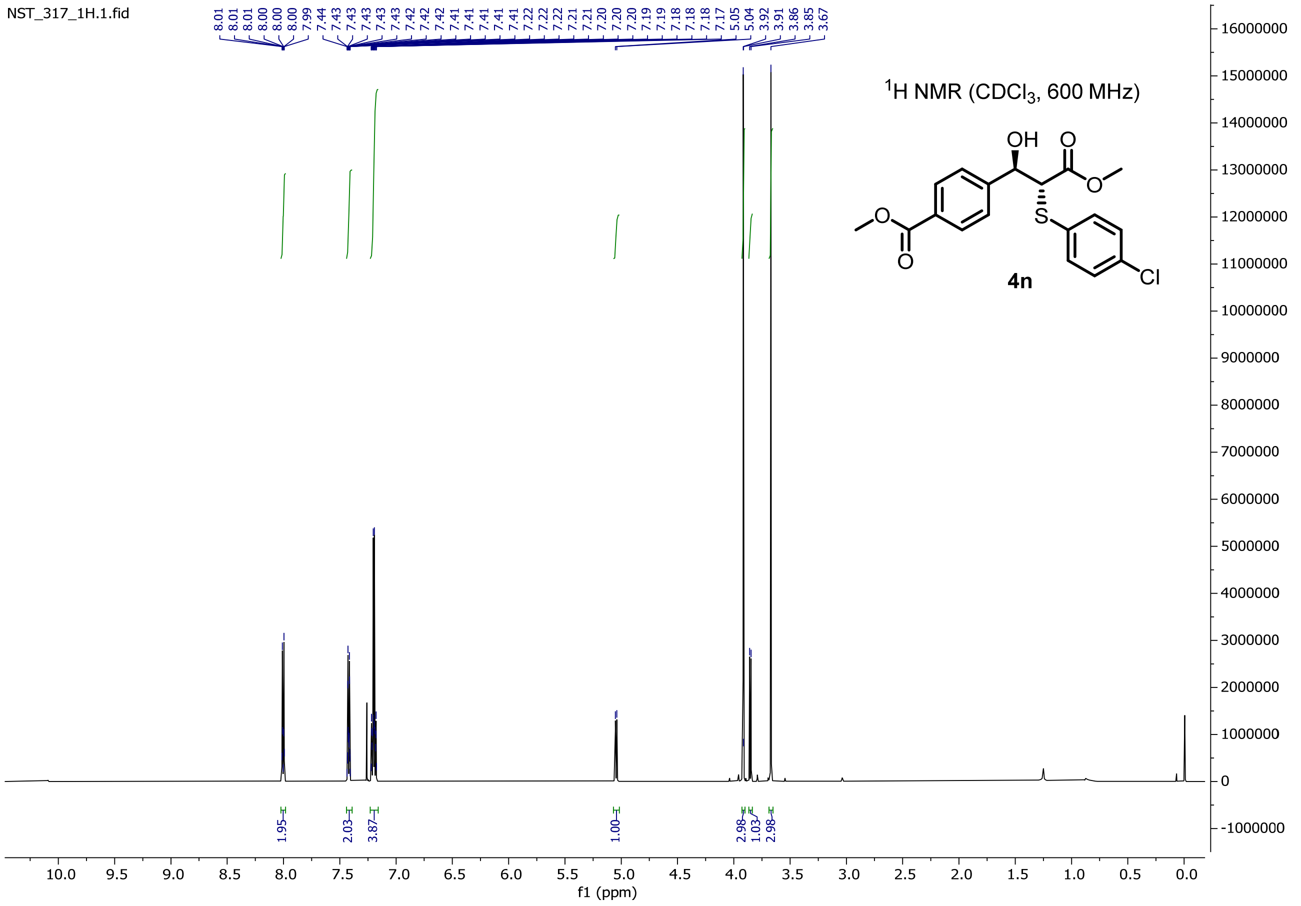
NST_317_1H.1.fid
8.01
8.01
8.01
8.00
8.00
8.00
7.99
7.44
7.43
7.43
7.43
7.43
7.42
7.42
7.42
7.41
7.41
7.41
7.41
7.41
7.22
7.22
7.22
7.21
7.21
7.20
7.20
7.20
7.19
7.19
7.18
7.18
7.18
7.17
5.05
5.04
3.92
3.91
3.86
3.85
3.67
¹H NMR (CDCl3, 600 MHz)
OH
O
O
O
S
O
Cl
4n
1.95
2.03
3.87
1.00
2.98
1.03
2.98
f1 (ppm)

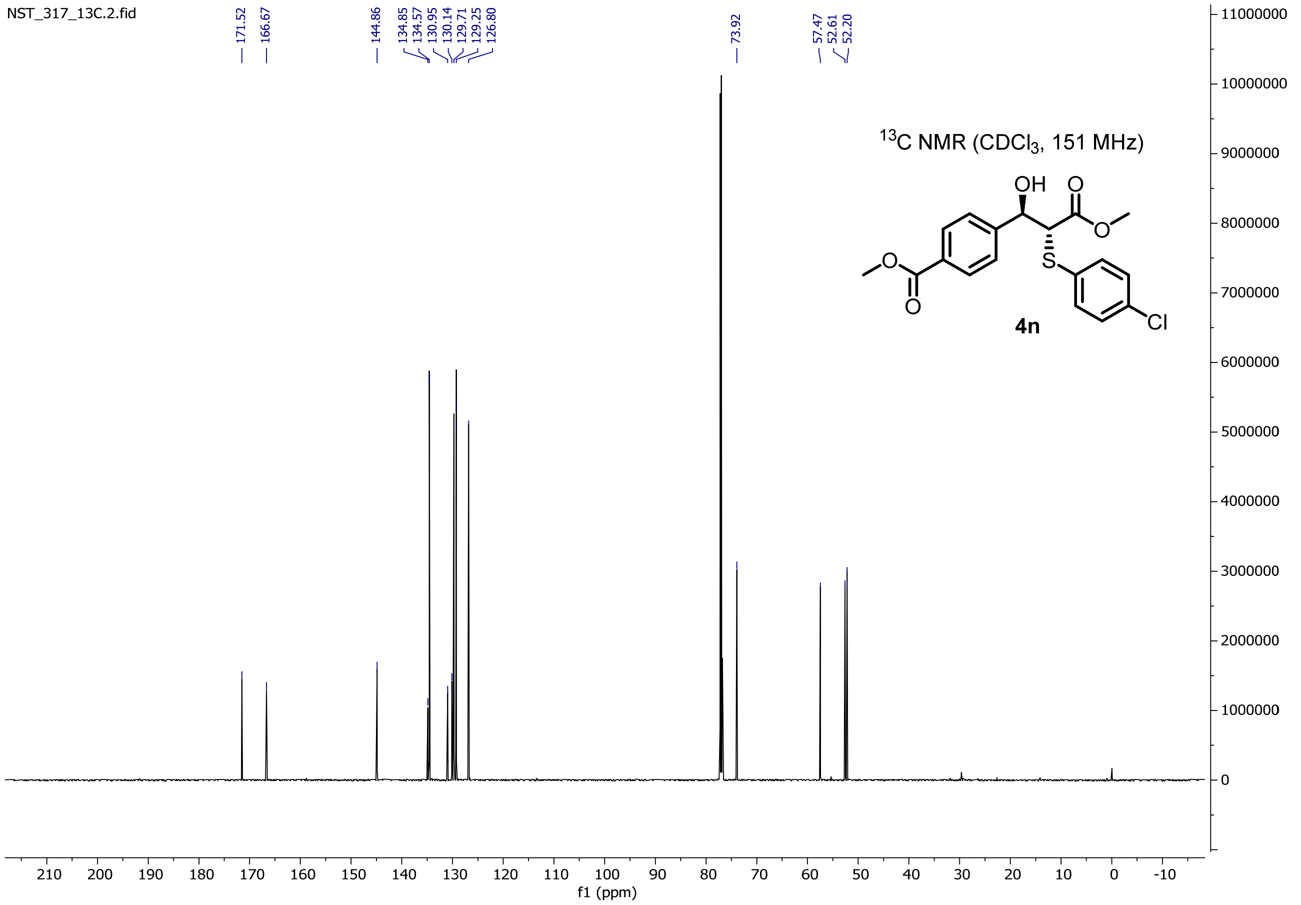
NST_317_13C.2.fid
171.52
166.67
144.86
134.85
134.57
130.95
130.14
129.71
129.25
126.80
73.92
57.47
52.61
52.20
¹³C NMR (CDCl3, 151 MHz)
OH
O
O
O
S
O
Cl
4n
f1 (ppm)

CSP HPLC traces:

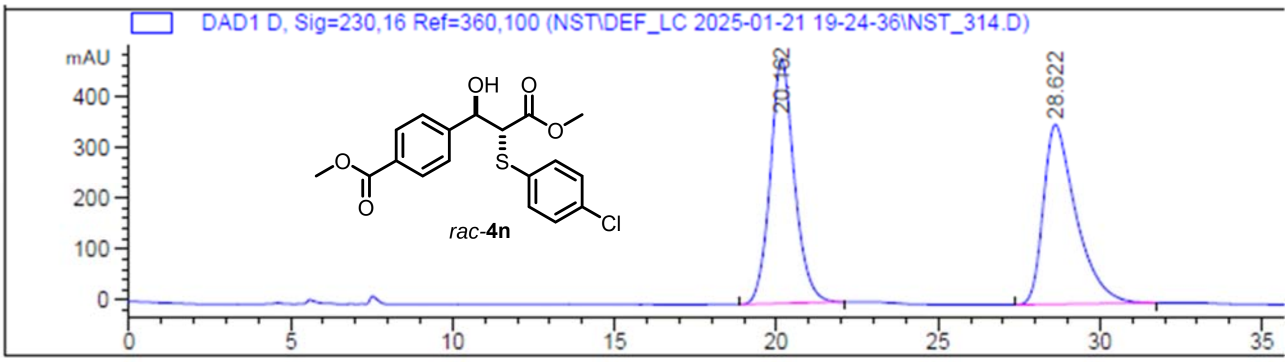

Signal 3: DAD1 D, Sig=230,16 Ref=360,100

Peak #	RetTime [min]	Type	Width [min]	Area [mAU*s]	Height [mAU]	Area %
1	20.162	BB	0.7753	2.48492e4	480.54150	50.0745
2	28.622	BB	1.0428	2.47753e4	353.02954	49.9255
Totals :				4.96246e4	833.57104	

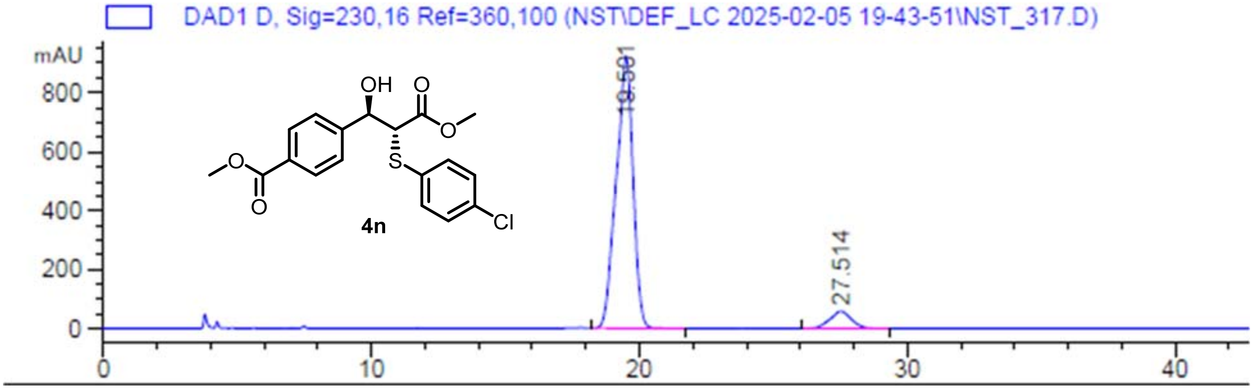

Signal 3: DAD1 D, Sig=230,16 Ref=360,100

Peak #	RetTime [min]	Type	Width [min]	Area [mAU*s]	Height [mAU]	Area %
1	19.501	BB	0.6239	4.03078e4	922.20831	92.3987
2	27.514	BB	0.8066	3315.95313	59.28054	7.6013
Totals :				4.36237e4	981.48885	

4o- Methyl (2*R*,3*R*)-2-((4-chlorophenyl)thio)-3-hydroxy-3-(4-nitrophenyl)propanoate

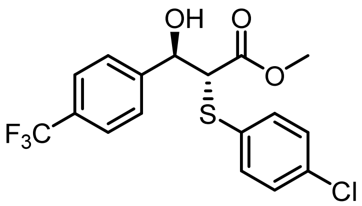

$C_{17}H_{14}ClF_3O_3S$

MW: 390.8 g/mol

Yield: 87%;

Colorless oil

The product (**4o**) was prepared using the general procedure **D** but running the reaction at 40 °C from **1a** (methyl 2-(dimethyl(oxo)-λ^6-sulfaneylidene)acetate), **2o** (4-trifluoromethylbenzaldehyde), and **3a** (4-chlorobenzenethiol). The product was obtained as a colorless oil (34 mg; 87% yield). The diastereomeric ratio obtained for the *anti*-couple is greater than 20:1. The enantiomeric excess of the product was determined by CSP HPLC: AD-H column, *n*-hexane/iPrOH 80:20, 0.75 mL/min, t_{maj} = 11.9 min; t_{min} = 22.0 min, 90% ee.

^{1}H NMR (600 MHz, $CDCl_3$) δ 7.59 (d, *J* = 7.1 Hz, 2H), 7.47 (d, *J* = 8.9 Hz, 2H), 7.24 – 7.13 (m, 4H), 5.06 (d, *J* = 8.0 Hz, 1H), 3.84 (d, *J* = 8.0 Hz, 1H), 3.70 (s, 3H), 3.32 (br s, 1H).

^{13}C NMR (151 MHz, $CDCl_3$) δ 171.5, 143.8, 134.9, 134.6, 130.9, 130.6 (q, *J* = 32.6 Hz), 129.3, 127.3, 125.4 (q, *J* = 3.8 Hz), 123.9 (q, *J* = 272.3 Hz), 73.9, 57.6, 52.7.

^{19}F NMR (376 MHz, $CDCl_3$) δ -62.6 (s, 3F).

HRMS (MALDI$^+$) m/z calcd for $C_{17}H_{14}ClF_3O_3SNa^+$ $[M+Na]^+$: 413.0202, found 413.0206.

$[\alpha]_D^{RT}$ = +42.4 (c = 1.0 g/100 mL, $CHCl_3$).

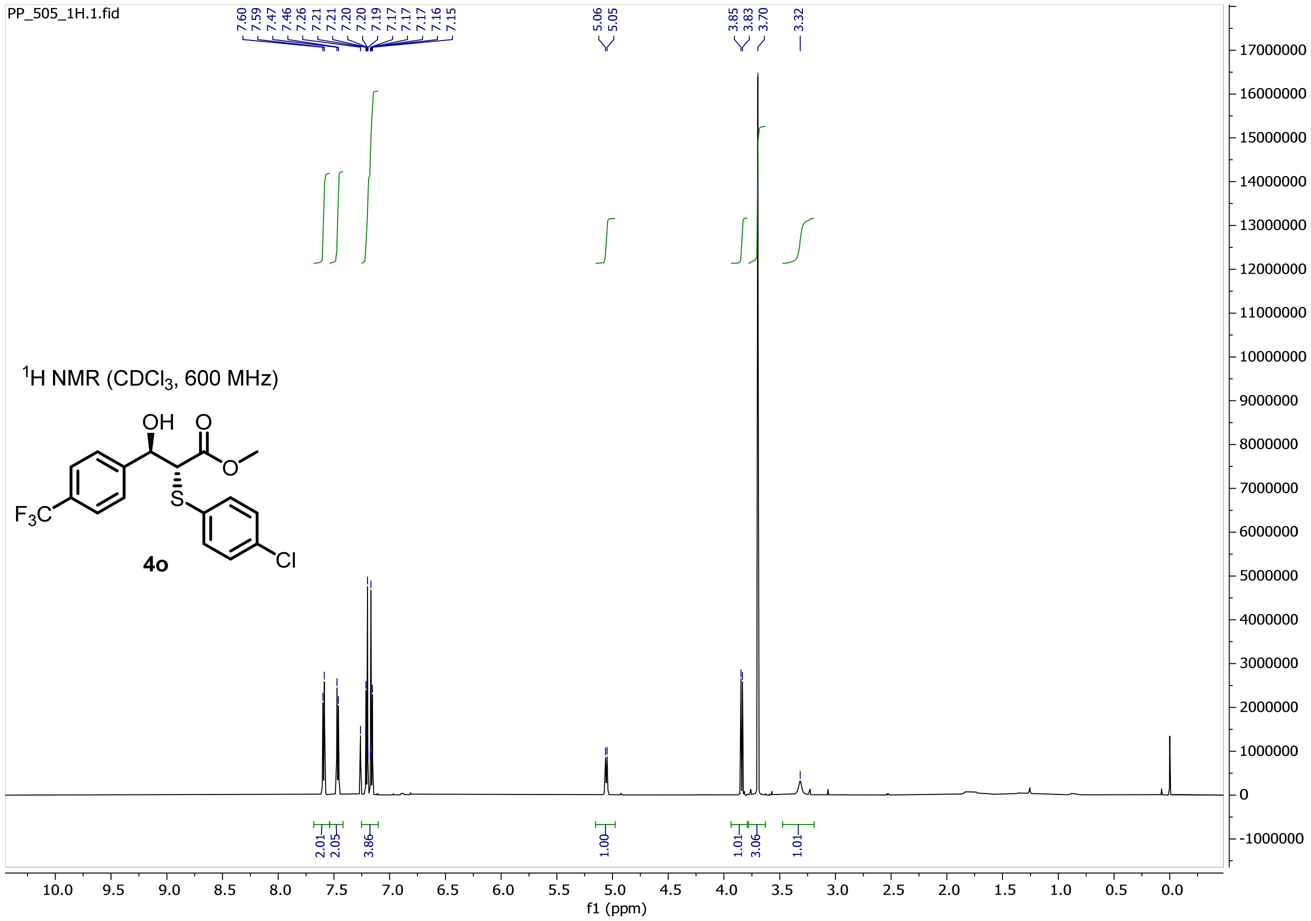
PP_505_1H.1.fid
7.60 7.59 7.47 7.46 7.26 7.21 7.21 7.20 7.20 7.19 7.17 7.17 7.17 7.16 7.15
5.06 5.05
3.85 3.83 3.70
3.32
^1H NMR ($CDCl_3$, 600 MHz)
OH
O
O
S
F_3C
Cl
4o
2.01
2.05
3.86
1.00
1.01
3.06
1.01
f1 (ppm)

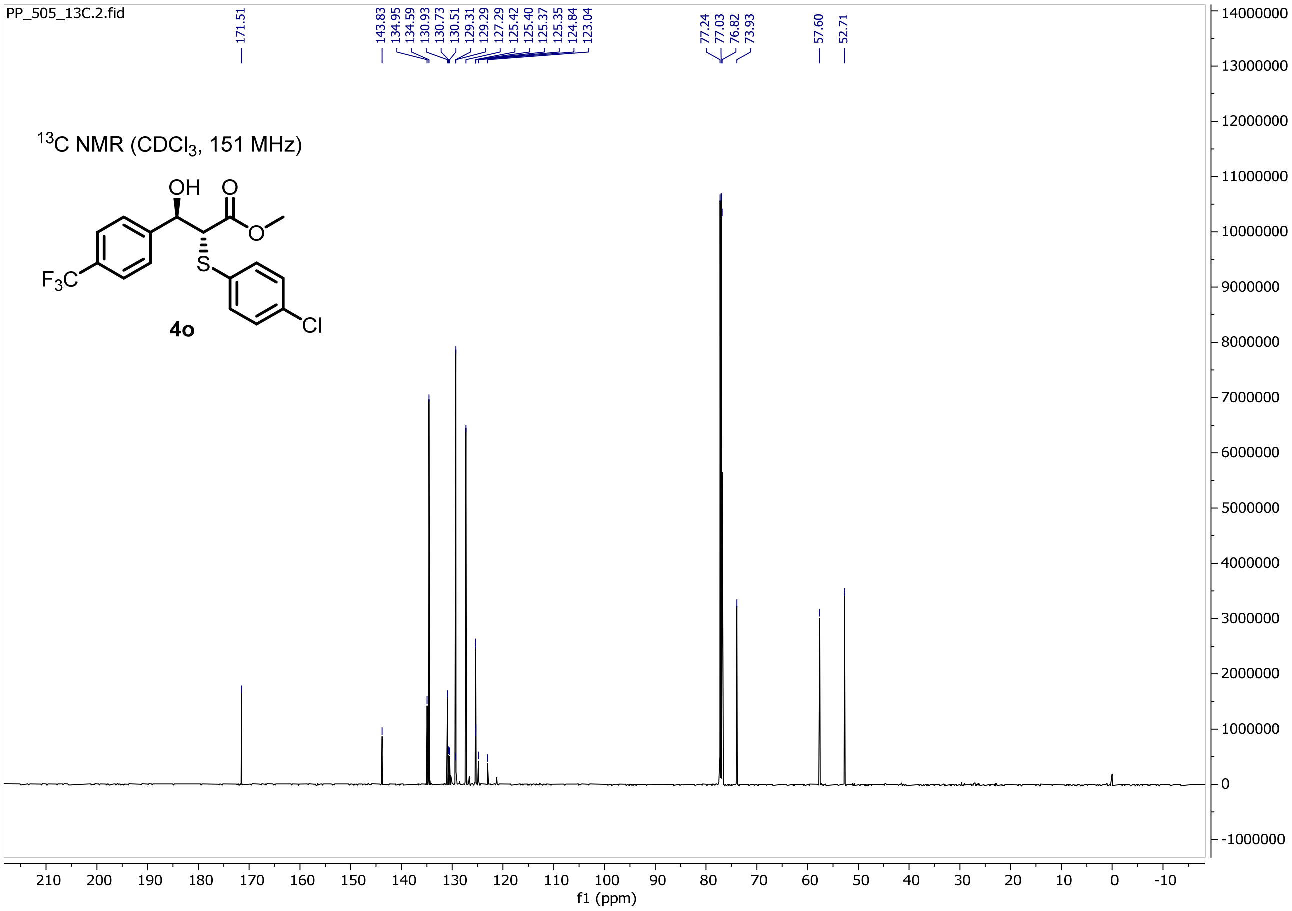
PP_505_13C.2.fid
171.51
143.83 134.95 134.59 130.93 130.73 130.51 129.31 129.29 127.29 125.42 125.40 125.37 125.35 124.84 123.04
77.24 77.03 76.82 73.93
57.60
52.71
^{13}C NMR ($CDCl_3$, 151 MHz)
OH
O
O
S
F_3C
Cl
4o
f1 (ppm)

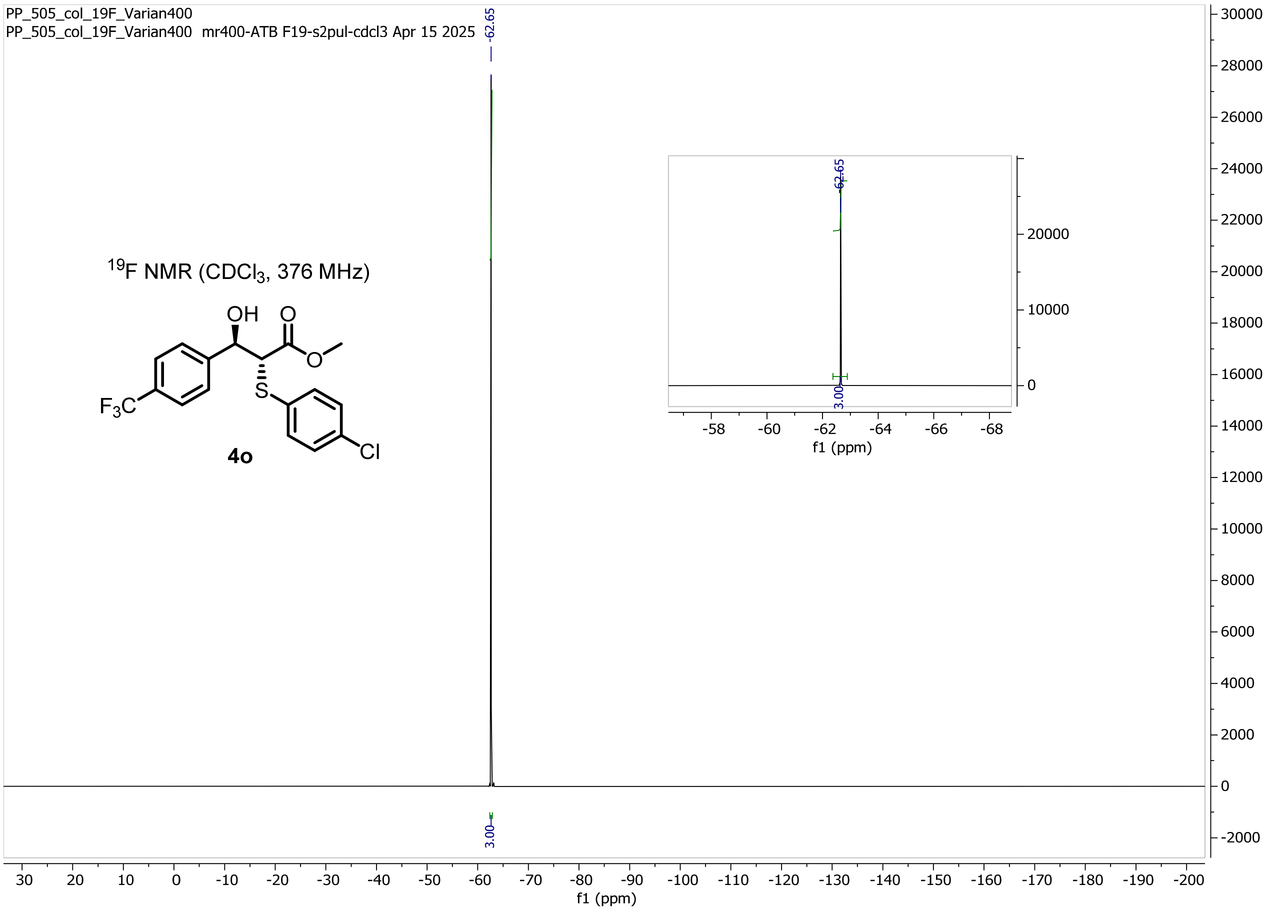

PP_505_col_19F_Varian400
PP_505_col_19F_Varian400 mr400-ATB F19-s2pul-cdcl3 Apr 15 2025
-62.65
^{19}F NMR (CDCl$_3$, 376 MHz)
OH
O
O
S
F$_3$C
Cl
4o
3.00
f1 (ppm)

CSP HPLC traces:

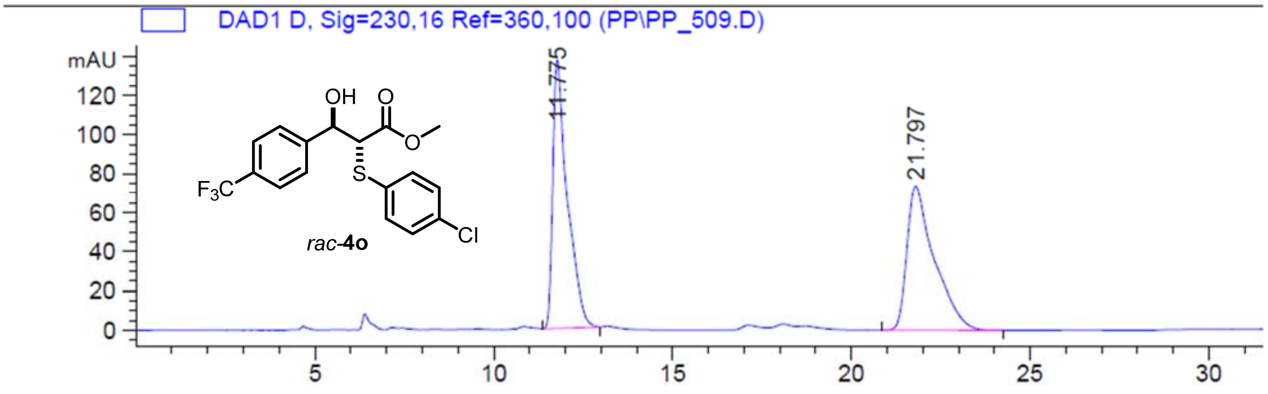

Signal 2: DAD1 D, Sig=230,16 Ref=360,100

Peak #	RetTime [min]	Type	Width [min]	Area [mAU*s]	Height [mAU]	Area %
1	11.775	BB	0.3954	3844.54126	136.98073	49.7110
2	21.797	BB	0.7481	3889.23779	73.41003	50.2890
Totals :				7733.77905	210.39076	

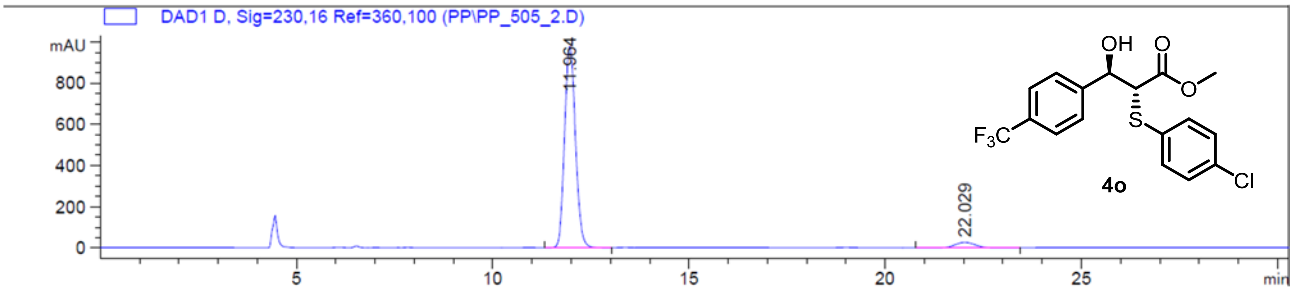

Signal 2: DAD1 D, Sig=230,16 Ref=360,100

Peak #	RetTime [min]	Type	Width [min]	Area [mAU*s]	Height [mAU]	Area %
1	11.964	BB	0.3061	1.89611e4	978.58557	95.1643
2	22.029	BB	0.5621	963.50153	26.75558	4.8357
Totals :				1.99246e4	1005.34115	

4p- Methyl (2*R*,3*R*)-2-((4-chlorophenyl)thio)-3-hydroxy-3-(4-nitrophenyl)propanoate

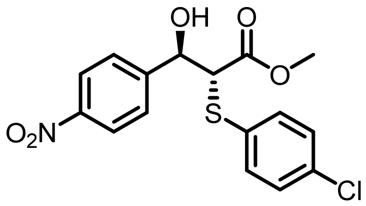

$C_{16}H_{14}ClNO_5S$

MW: 367.0ii g/mol

Yield: 63%;

Colorless oil

The product (**4p**) was prepared using the general procedure **D** from **1a** (methyl 2-(dimethyl(oxo)-λ^6-sulfaneylidene)acetate), **2p** (4-nitrobenzaldehyde), and **3a** (4-chlorobenzenethiol). The product was obtained as a colorless oil (23 mg; 63% yield). The diastereomeric ratio obtained for the *anti*-couple is greater than 20:1. The enantiomeric excess of the product was determined by CSP HPLC: AD-H column, *n*-hexane/iPrOH 80:20, 0.75 mL/min, t_{maj} = 17.7 min; t_{min} = 34.7 min, 88% ee.

^{1}H NMR (600 MHz, $CDCl_3$) δ 8.25 – 8.18 (m, 2H), 7.58 – 7.51 (m, 2H), 7.26 – 7.19 (m, 4H), 5.10 (d, *J* = 7.7 Hz, 1H), 3.85 (d, *J* = 7.7 Hz, 1H), 3.69 (s, 3H).

^{13}C NMR (151 MHz, $CDCl_3$) δ 171.3, 147.8, 147.1, 135.2, 134.6, 130.6, 129.4, 127.8, 123.6, 73.4, 57.4, 52.8.

HRMS (MALDI$^-$) m/z calcd for $C_{16}H_{13}ClNO_5S$ [M-H]$^-$: 366.0208, found 366.0203.

$[\alpha]_D^{RT}$ = +30.7 (c = 1.0 g/100 mL, $CHCl_3$).

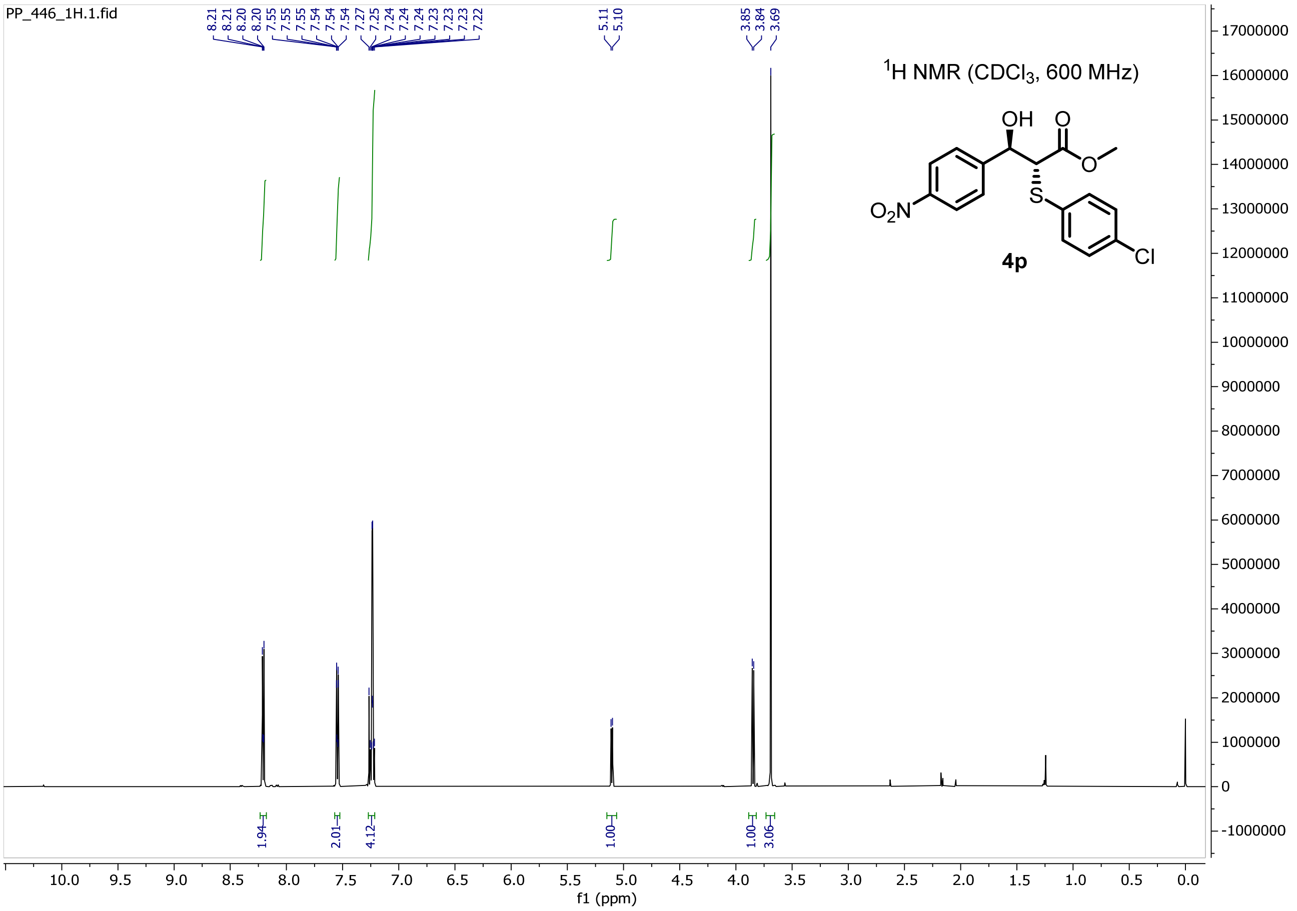
PP_446_1H.1.fid
8.21
8.21
8.20
8.20
7.55
7.55
7.54
7.54
7.54
7.27
7.25
7.24
7.24
7.24
7.23
7.23
7.23
7.22
5.11
5.10
3.85
3.84
3.69
¹H NMR (CDCl3, 600 MHz)
OH
O
O
S
O2N
Cl
4p
1.94
2.01
4.12
1.00
1.00
3.06
f1 (ppm)

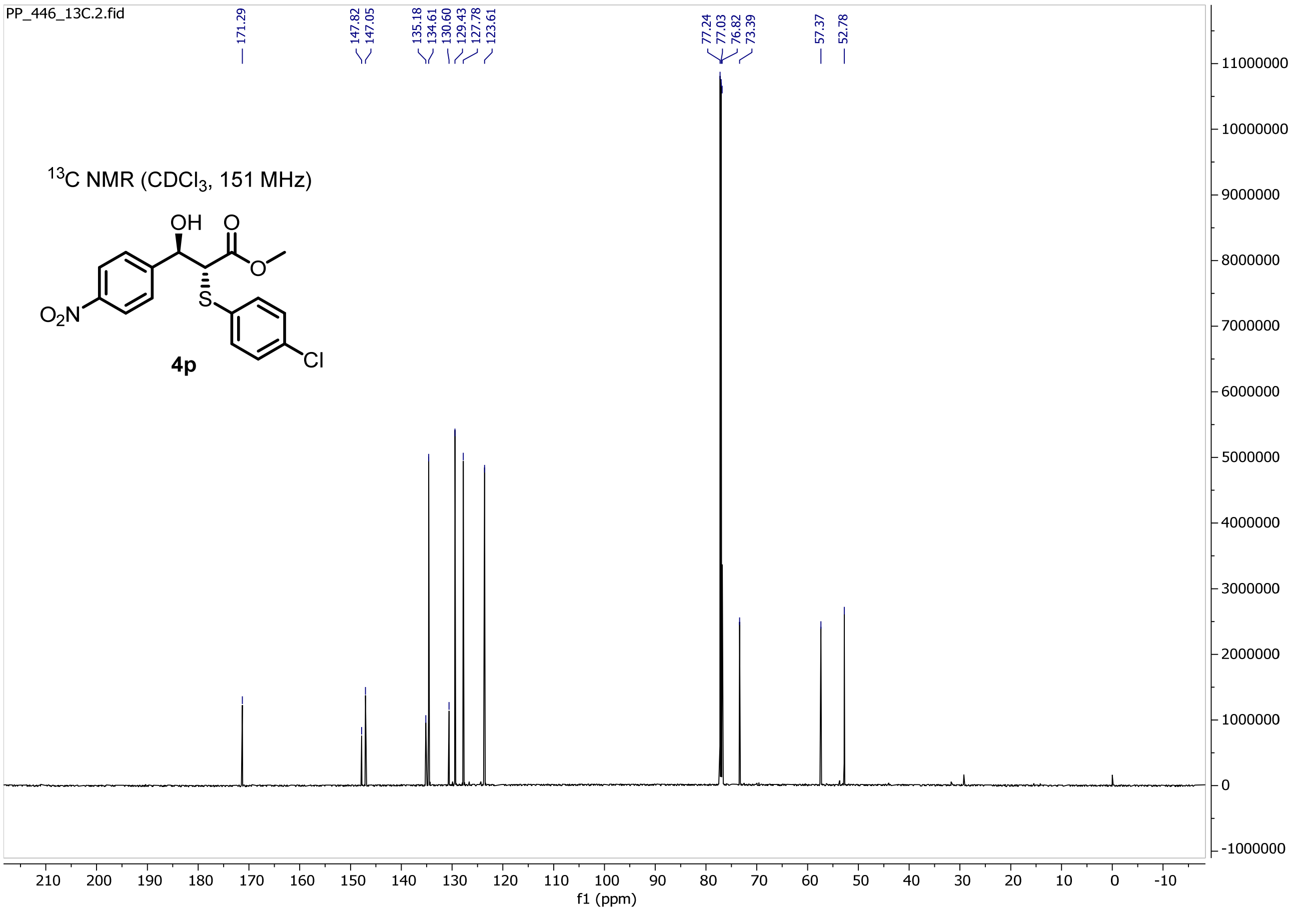
PP_446_13C.2.fid
171.29
147.82
147.05
135.18
134.61
130.60
129.43
127.78
123.61
77.24
77.03
76.82
73.39
57.37
52.78
¹³C NMR (CDCl3, 151 MHz)
OH
O
O
S
O2N
Cl
4p
f1 (ppm)

CSP HPLC traces:

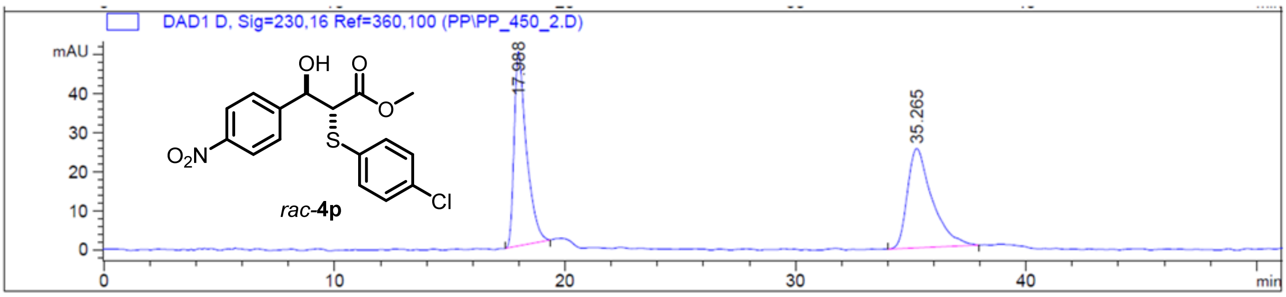

Signal 3: DAD1 D, Sig=230,16 Ref=360,100

Peak #	RetTime [min]	Type	Width [min]	Area [mAU*s]	Height [mAU]	Area %
1	17.988	BB	0.5413	1850.48157	49.82500	49.4052
2	35.265	BB	1.0688	1895.04150	25.44859	50.5948
Totals :				3745.52307	75.27359	

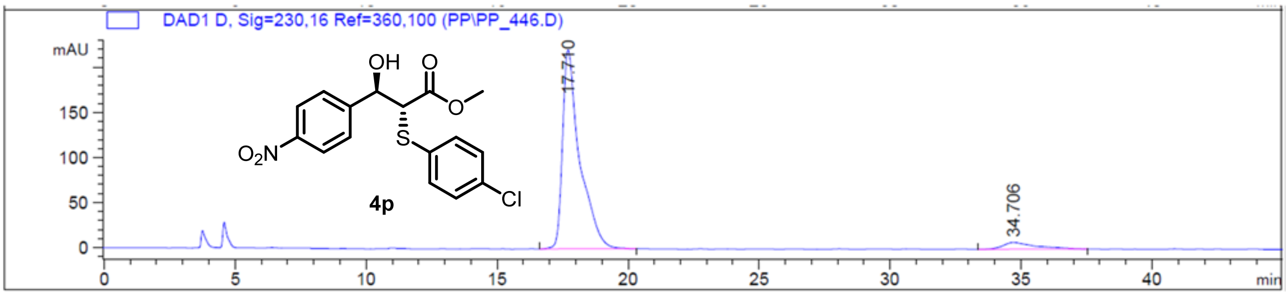

Signal 3: DAD1 D, Sig=230,16 Ref=360,100

Peak #	RetTime [min]	Type	Width [min]	Area [mAU*s]	Height [mAU]	Area %
1	17.710	BB	0.6492	1.00962e4	220.90950	94.0702
2	34.706	BB	1.1220	636.42731	7.35156	5.9298
Totals :				1.07327e4	228.26106	

4q-Methyl (2*R*,3*R*)-2-((4-chlorophenyl)thio)-3-hydroxy-3-(pyridin-3-yl)propanoate

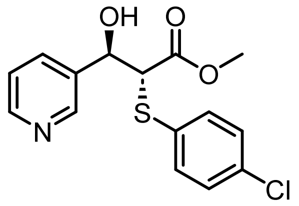

$C_{15}H_{14}ClNO_3S$

MW: 323.8 g/mol

Yield: 40%;

Pale yellow oil

The product (**4q**) was prepared using the general procedure **D** from **1a** (methyl 2-(dimethyl(oxo)-λ^6-sulfaneylidene)acetate), **2q** (nicotinaldehyde), and **3a** (4-chlorobenzenethiol). The product was obtained as a pale yellow oil (25 mg; 40% yield). The diastereomeric ratio obtained for the *anti*-couple is greater than 20:1. The enantiomeric excess of the product was determined by CSP HPLC: AD-H column, *n*-hexane/iPrOH 80:20, 0.75 mL/min, t_{maj} = 15.3 min; t_{min} = 24.1 min, 88% ee.

^{1}H NMR (600 MHz, $CDCl_3$) δ 8.53 (d, *J* = 2.2 Hz, 1H), 8.46 (dd, *J* = 4.9, 1.6 Hz, 1H), 7.68 (dt, *J* = 7.9, 2.2 Hz, 1H), 7.24 (dd, *J* = 7.9, 4.9 Hz, 1H), 7.21 – 7.17 (m, 2H), 7.16 – 7.11 (m, 2H), 5.02 (d, *J* = 8.7 Hz, 1H), 3.82 (d, *J* = 8.7 Hz, 1H), 3.72 (s, 3H).

^{13}C NMR (151 MHz, $CDCl_3$) δ 171.4, 149.3, 148.5, 135.9, 134.8, 134.6, 134.5, 130.8, 129.3, 123.5, 72.2, 57.6, 52.7.

HRMS (MALDI$^+$) m/z calcd for $C_{15}H_{15}ClNO_3S^+$ $[M+H]^+$: 324.0456, found: 324.0461.

$[\alpha]_D^{RT}$ = +36.7 (c = 1.0 g/100 mL, $CHCl_3$).

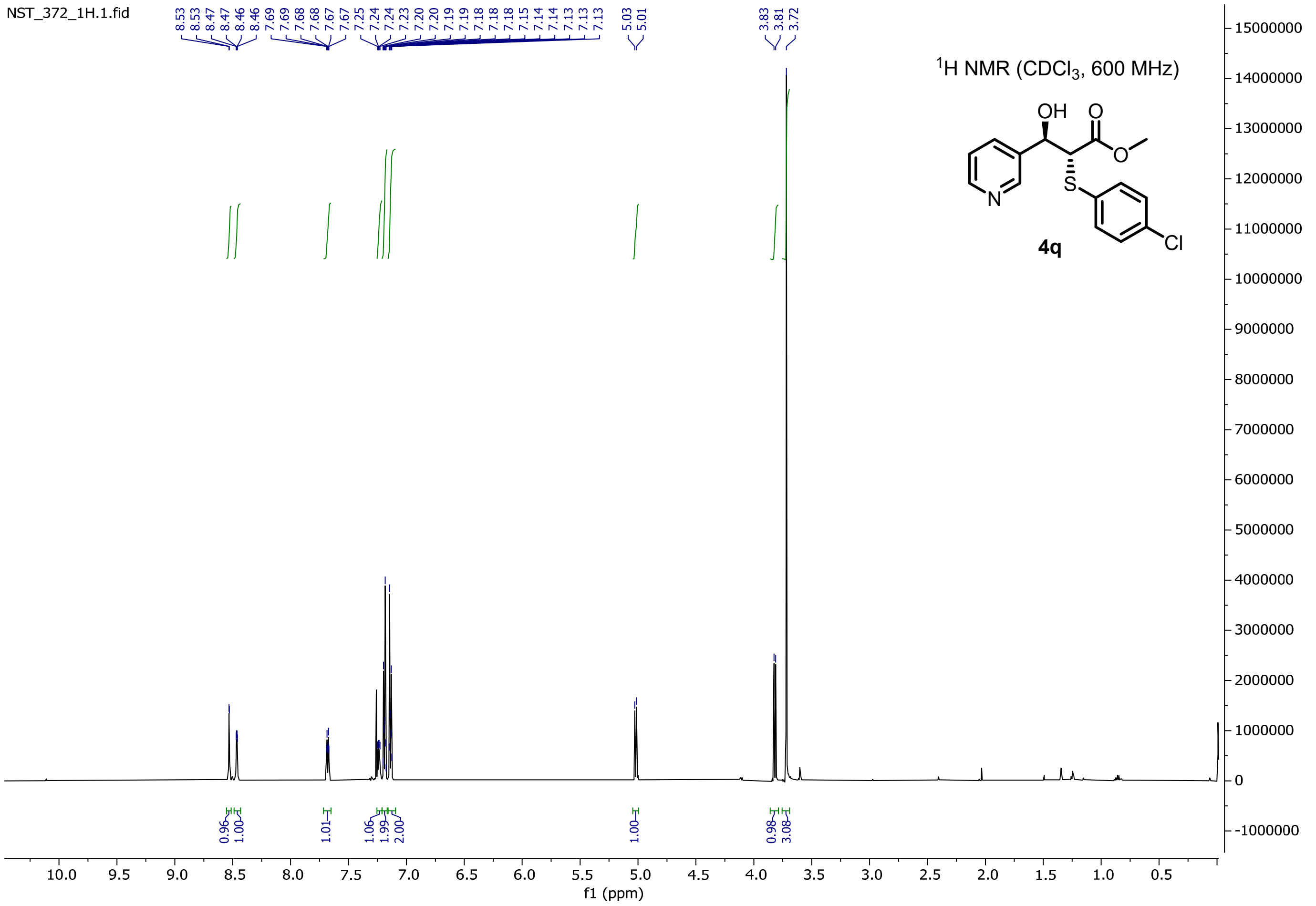
NST_372_1H.1.fid
8.53 8.53 8.47 8.46 8.46 7.69 7.69 7.68 7.68 7.67 7.67 7.25 7.24 7.24 7.23 7.20 7.20 7.19 7.19 7.18 7.18 7.18 7.15 7.14 7.14 7.13 7.13 7.13 7.13
5.03 5.01
3.83 3.81 3.72
1H NMR (CDCl3, 600 MHz)
OH
O
O
S
N
Cl
4q
0.96 1.00 1.01 1.06 1.99 2.00 1.00 0.98 3.08
f1 (ppm)

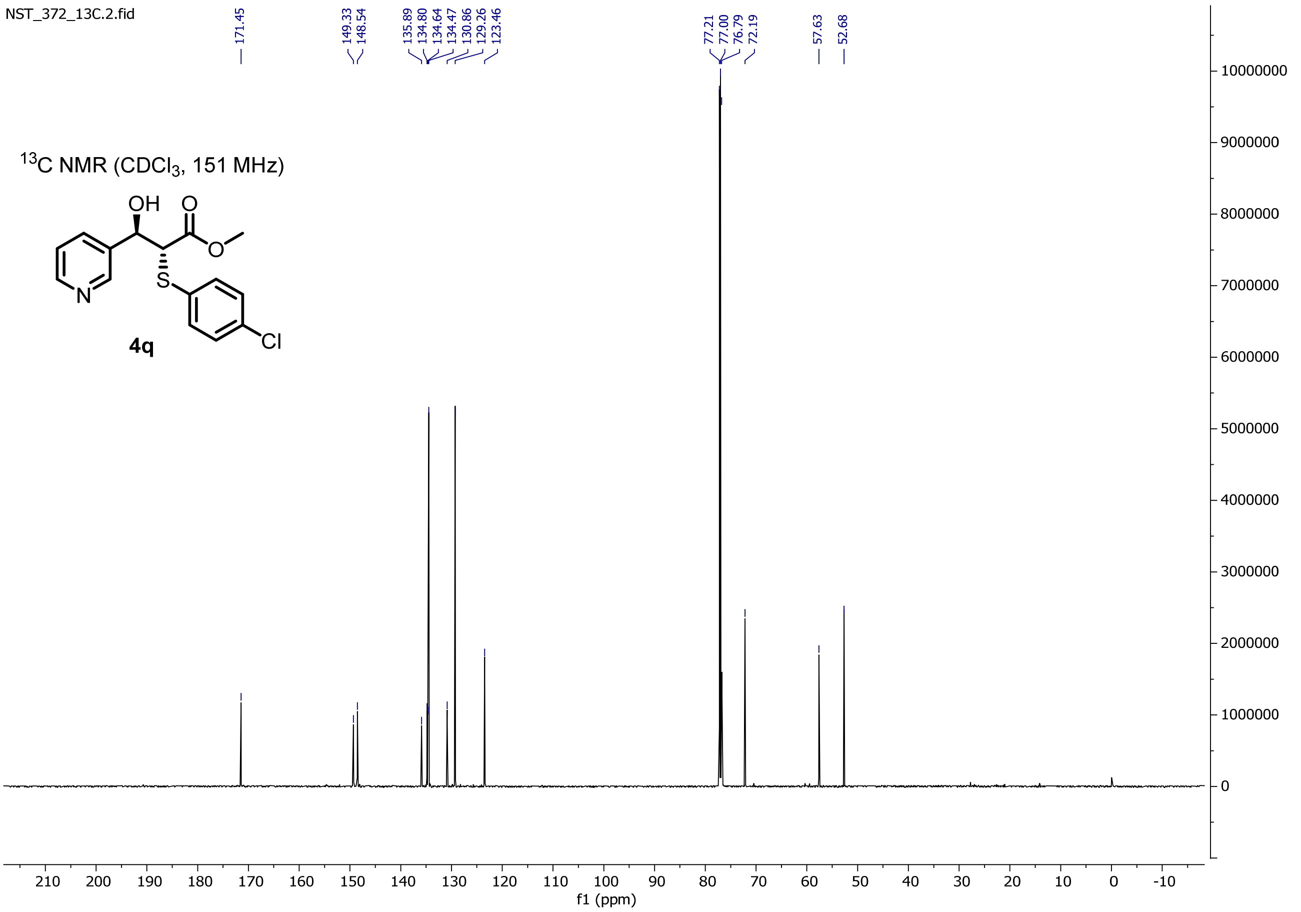
NST_372_13C.2.fid
171.45
149.33 148.54
135.89 134.80 134.64 134.47 130.86 129.26 123.46
77.21 77.00 76.79 72.19
57.63
52.68
13C NMR (CDCl3, 151 MHz)
OH
O
O
S
N
Cl
4q
f1 (ppm)

CSP HPLC traces:

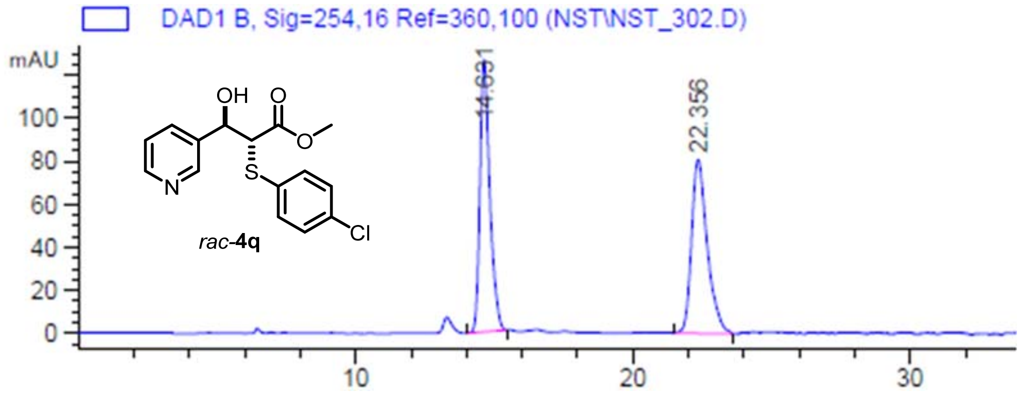

Signal 2: DAD1 B, Sig=254,16 Ref=360,100

Peak #	RetTime [min]	Type	Width [min]	Area [mAU*s]	Height [mAU]	Area %
1	14.631	BB	0.3664	3108.84180	126.63940	49.4375
2	22.356	BB	0.5619	3179.59204	80.66530	50.5625
Totals :				6288.43384	207.30470	

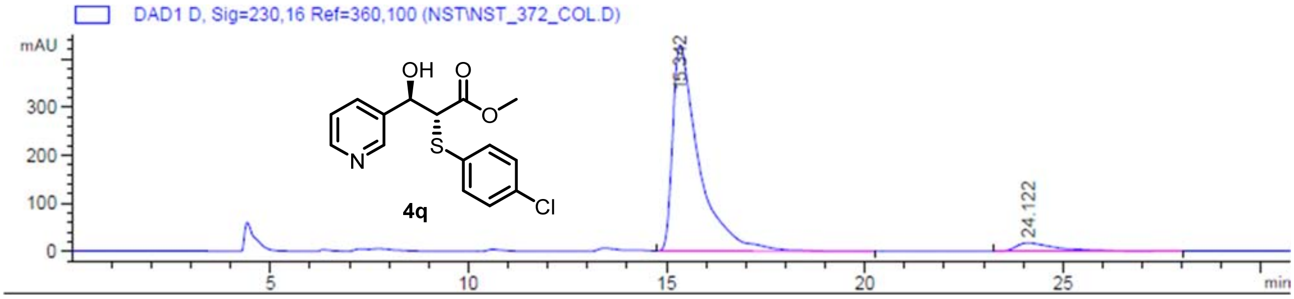

Signal 2: DAD1 D, Sig=230,16 Ref=360,100

Peak #	RetTime [min]	Type	Width [min]	Area [mAU*s]	Height [mAU]	Area %
1	15.342	BB	0.6603	1.97417e4	427.83264	93.8156
2	24.122	BB	1.0147	1301.38550	17.84694	6.1844
Totals :				2.10431e4	445.67958	

4r- Methyl (2*R*,3*R*)-2-((4-chlorophenyl)thio)-3-hydroxy-3-(*o*-tolyl)propanoate

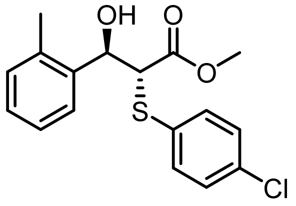

$C_{17}H_{17}ClO_3S$

MW: 336.8 g/mol

Yield: 86%;

Colorless oil

The product (**4r**) was prepared using the general procedure **B** from **1a** (methyl 2-(dimethyl(oxo)-λ^6-sulfaneylidene)acetate), **2r** (2-methylbenzaldehyde), and **3a** (4-chlorobenzenethiol). The product was obtained as a colorless oil (29 mg; 86% yield). The diastereomeric ratio obtained for the *anti*-couple is greater than 20:1. The enantiomeric excess of the product was determined by CSP HPLC: AD-H column, *n*-hexane/iPrOH 80:20, 0.75 mL/min, t_{maj} = 11.9 min; t_{min} = 13.5 min, 89% ee.

^{1}H NMR (600 MHz, $CDCl_3$) δ 7.32 (dd, *J* = 7.5, 1.6 Hz, 1H), 7.22 – 7.11 (m, 7H), 5.28 (dd, *J* = 8.4, 4.3 Hz, 1H), 3.90 (d, *J* = 8.4 Hz, 1H), 3.70 (s, 3H), 2.97 (d, *J* = 5.2 Hz, 1H), 2.37 (s, 3H).

^{13}C NMR (151 MHz, $CDCl_3$) δ 171.8, 138.0, 135.9, 134.73, 134.71, 131.4, 130.6, 129.1, 128.3, 126.3, 126.1, 70.7, 57.5, 52.5, 19.6.

HRMS (MALDI$^+$) m/z calcd for $C_{17}H_{17}ClO_3SNa^+$ $[M+Na]^+$: 359.0479, found: 359.0485.

$[\alpha]_D^{RT}$ = +5.0 (c = 1.0 g/100 mL, $CHCl_3$).

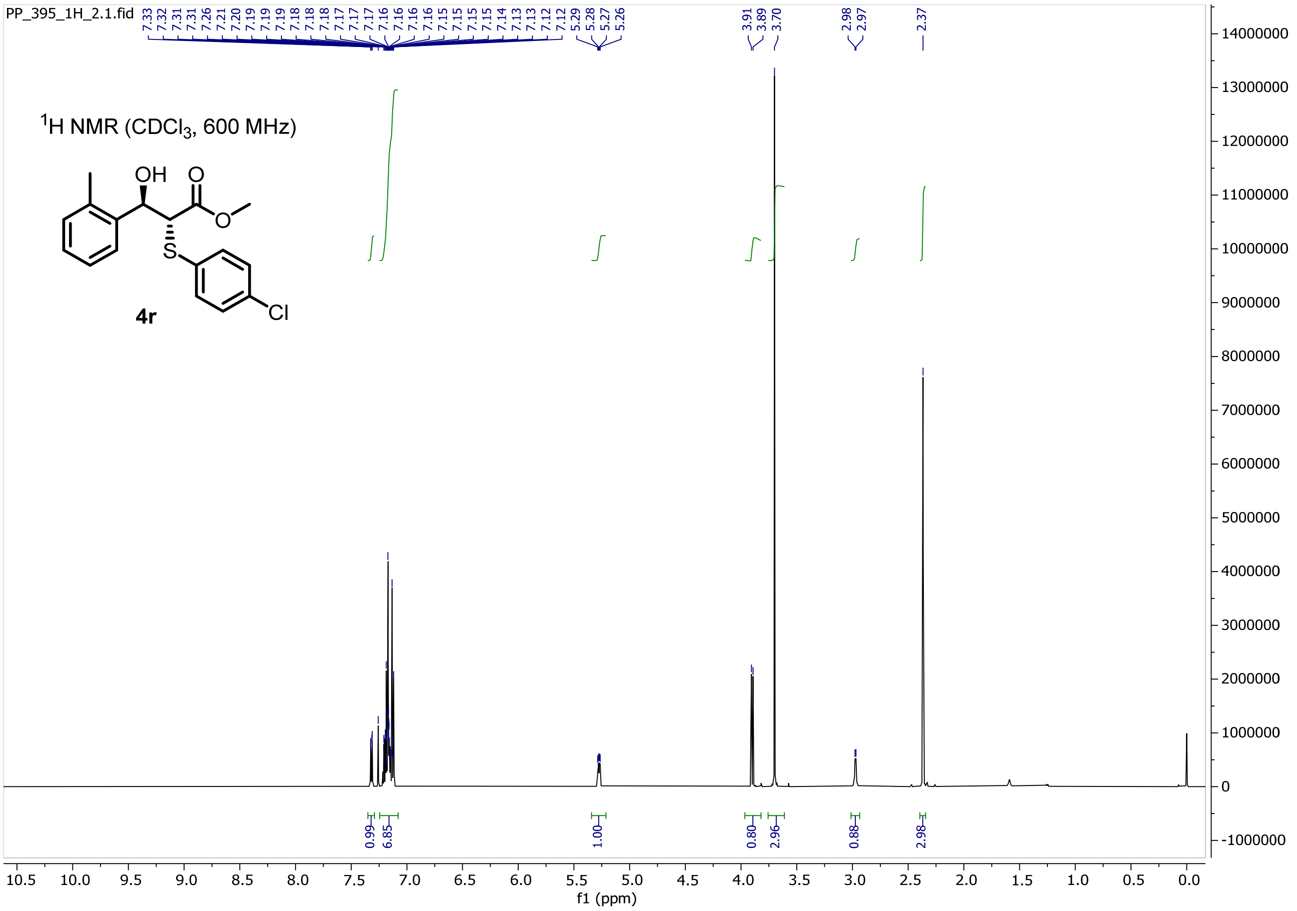
PP_395_1H_2.1.fid
7.33 7.32 7.31 7.26 7.21 7.20 7.19 7.19 7.19 7.18 7.18 7.18 7.17 7.17 7.16 7.16 7.16 7.16 7.15 7.15 7.15 7.15 7.14 7.13 7.13 7.12 7.12 5.29 5.28 5.27 5.26 3.91 3.89 3.70 2.98 2.97 2.37
1H NMR (CDCl3, 600 MHz)
OH
O
S
Cl
4r
0.99 6.85 1.00 0.80 2.96 0.88 2.98
f1 (ppm)

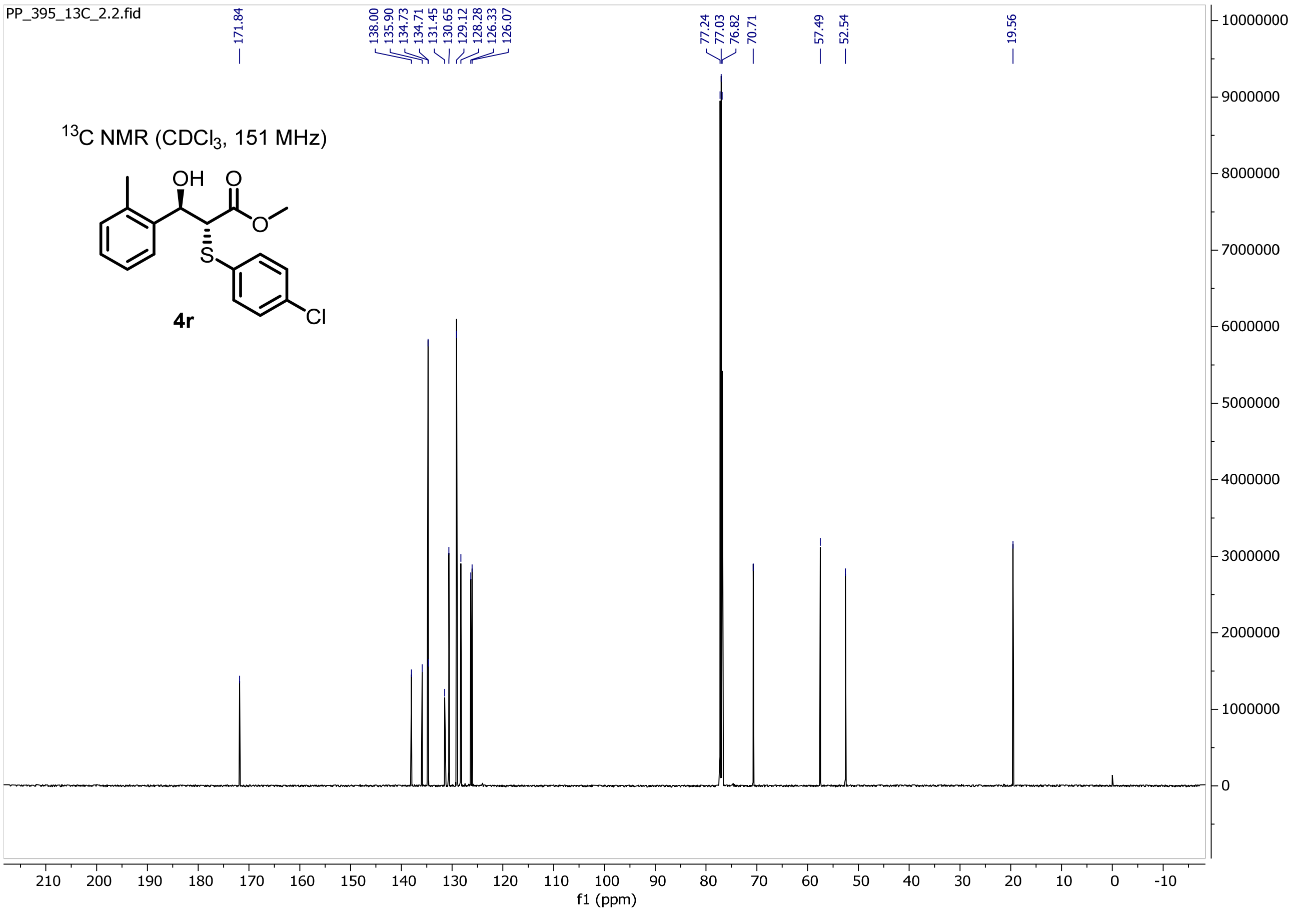
PP_395_13C_2.2.fid
171.84 138.00 135.90 134.73 134.71 131.45 130.65 129.12 128.28 126.33 126.07 77.24 77.03 76.82 70.71 57.49 52.54 19.56
13C NMR (CDCl3, 151 MHz)
OH
O
S
Cl
4r
f1 (ppm)

CSP HPLC traces:

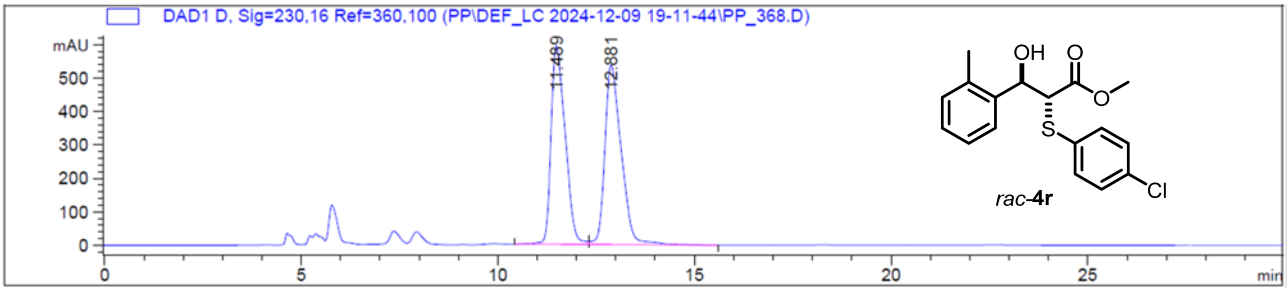

Signal 3: DAD1 D, Sig=230,16 Ref=360,100

Peak #	RetTime [min]	Type	Width [min]	Area [mAU*s]	Height [mAU]	Area %
1	11.489	BV	0.4112	1.48272e4	594.03088	48.8761
2	12.881	VB	0.4228	1.55092e4	537.17975	51.1239
Totals :				3.03364e4	1131.21063	

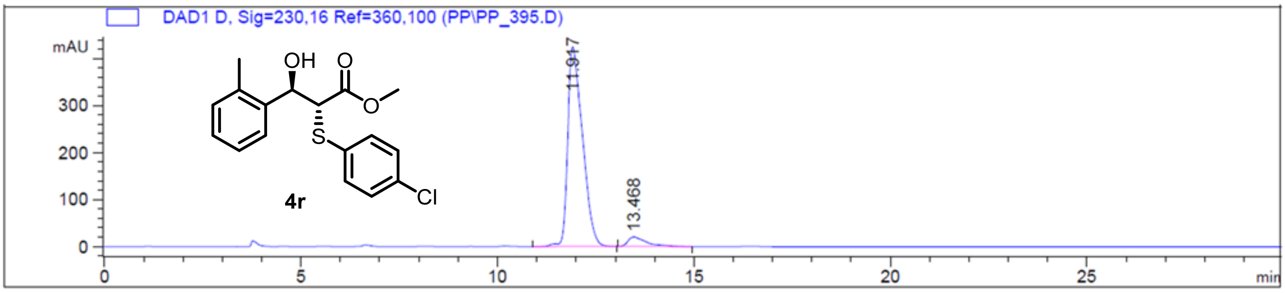

Signal 3: DAD1 D, Sig=230,16 Ref=360,100

Peak #	RetTime [min]	Type	Width [min]	Area [mAU*s]	Height [mAU]	Area %
1	11.917	BB	0.3699	1.10636e4	424.68369	94.3971
2	13.468	BB	0.4568	656.67670	20.01576	5.6029
Totals :				1.17203e4	444.69945	

4s- Methyl (2*R*,3*R*)-2-((4-chlorophenyl)thio)-3-hydroxy-3-(m-tolyl)propanoate

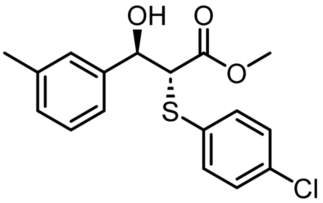

$C_{17}H_{17}ClO_3S$

MW: 336.8 g/mol

Yield: 72%;

Colorless oil

The product (**4s**) was prepared using the general procedure **B** from **1a** (methyl 2-(dimethyl(oxo)-λ^6-sulfaneylidene)acetate), **2s** (3-methylbenzaldehyde), and **3a** (4-chlorobenzenethiol). The product was obtained as a colorless oil (24.3 mg; 72% yield). The diastereomeric ratio obtained for the *anti*-couple is greater than 20:1. The enantiomeric excess of the product was determined by CSP HPLC: AD-H column, *n*-hexane/iPrOH 80:20, 0.75 mL/min, t_{maj} = 11.0 min; t_{min} = 13.6 min, 85% ee.

^{1}H NMR (600 MHz, $CDCl_3$) δ 7.25 – 7.21 (m, 1H), 7.21 – 7.15 (m, 4H), 7.15 – 7.11 (m, 3H), 4.96 (d, *J* = 8.3 Hz, 1H), 3.88 (d, *J* = 8.3 Hz, 1H), 3.69 (s, 3H), 2.33 (s, 3H).

^{13}C NMR (151 MHz, $CDCl_3$) δ 171.7, 139.7, 138.2, 134.59, 134.56, 131.4, 129.3, 129.1, 128.4, 127.4, 124.0, 74.6, 57.6, 52.5, 21.4.

HRMS (MALDI$^+$) m/z calcd for $C_{17}H_{17}ClO_3SNa^+$ $[M+Na]^+$: 359.0479, found: 359.0485.

$[\alpha]_D^{RT}$ = +70.9 (c = 1.0 g/100 mL, $CHCl_3$).

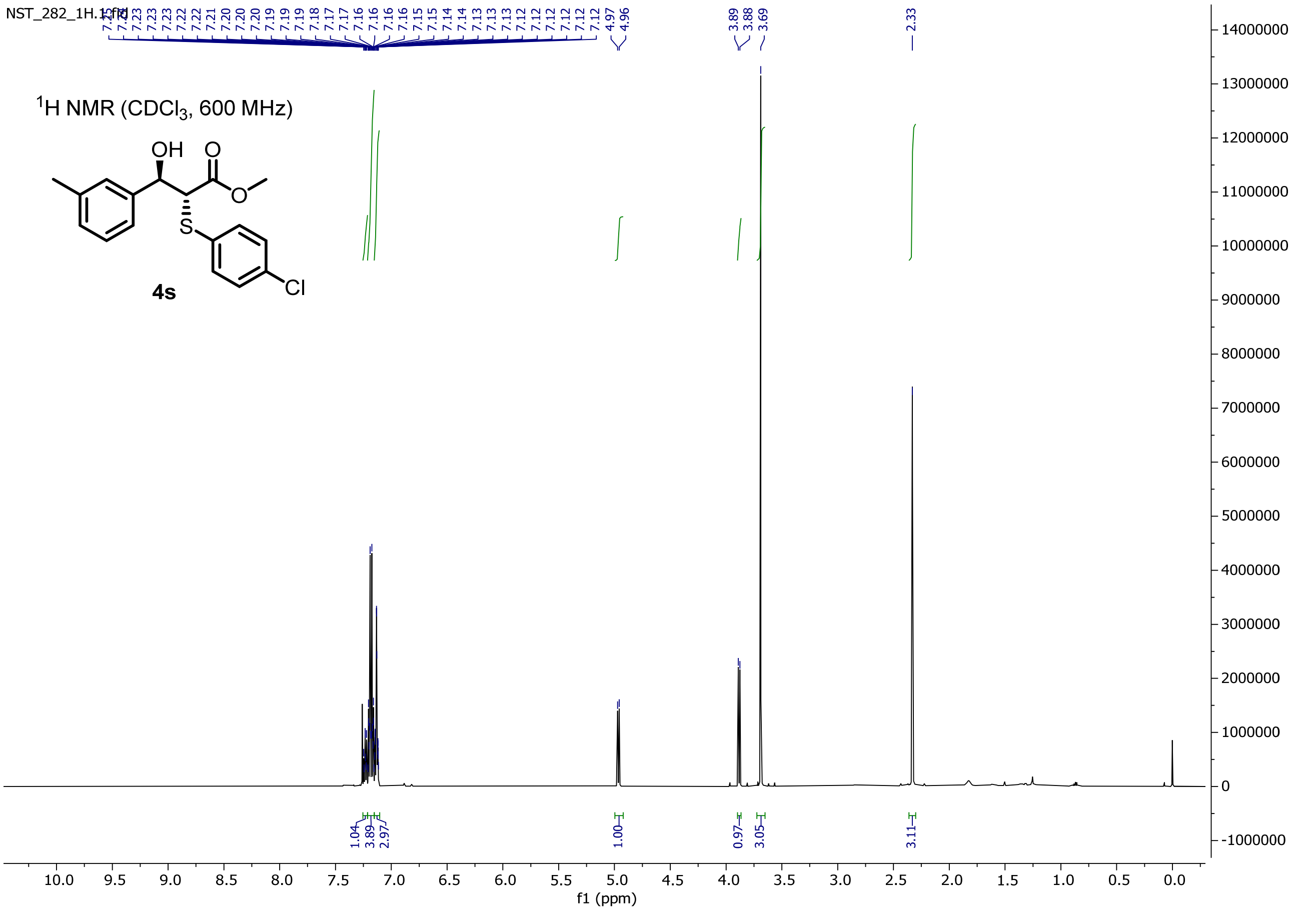
NST_282_1H.1.fid
1H NMR (CDCl3, 600 MHz)
OH
O
S
Cl
4s
4.97
4.96
3.89
3.88
3.69
2.33
1.04
3.89
2.97
1.00
0.97
3.05
3.11
f1 (ppm)

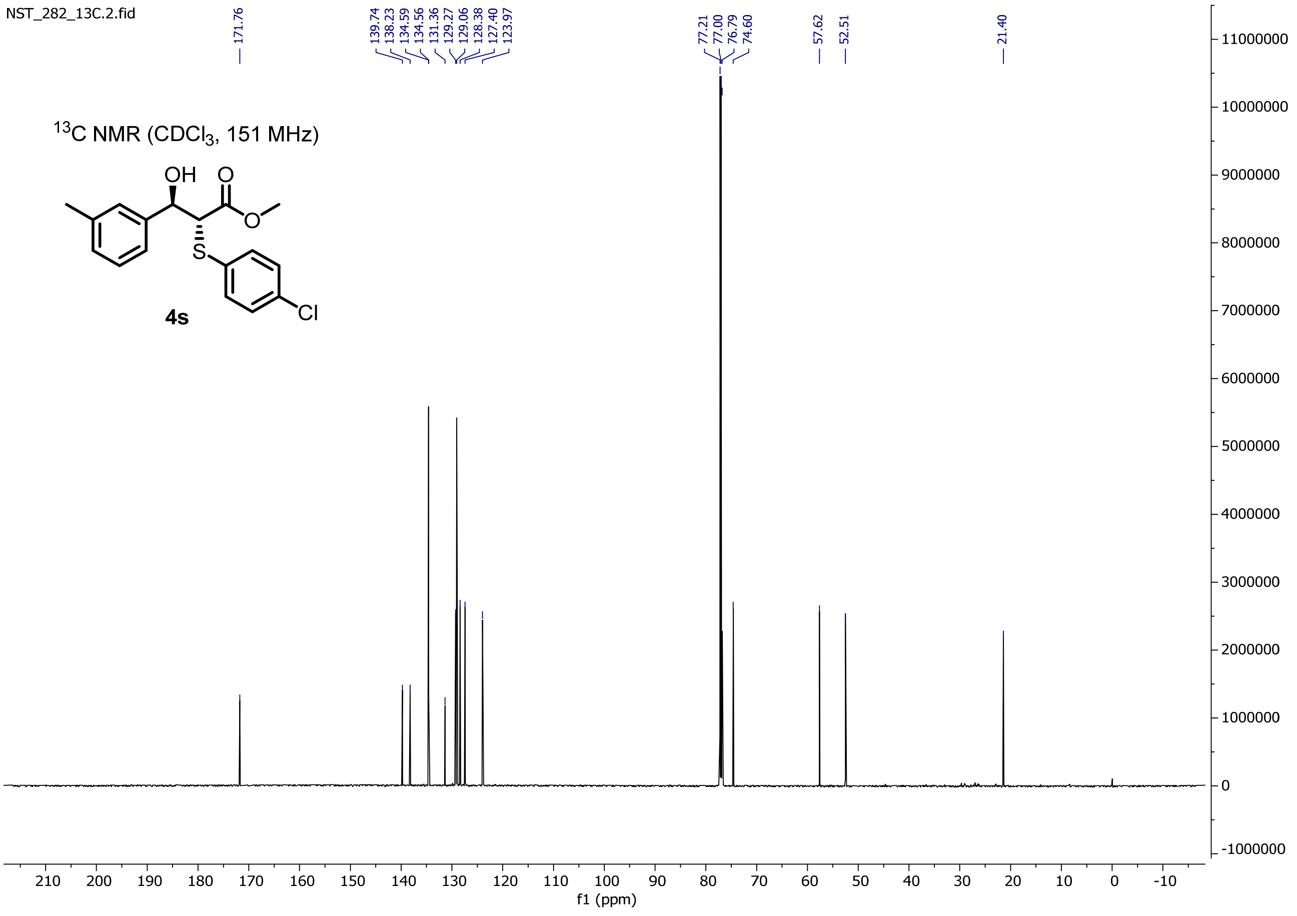
NST_282_13C.2.fid
171.76
139.74
138.23
134.59
134.56
131.36
129.27
129.06
128.38
127.40
123.97
77.21
77.00
76.79
74.60
57.62
52.51
21.40
13C NMR (CDCl3, 151 MHz)
OH
O
S
Cl
4s
f1 (ppm)

CSP HPLC traces:

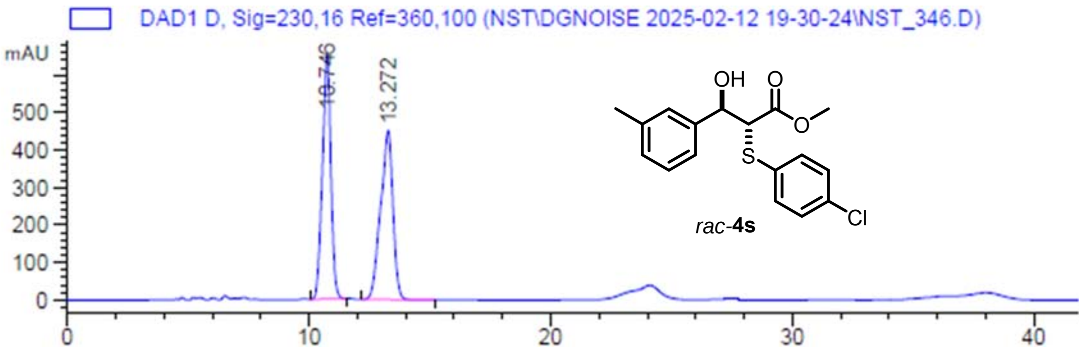

Signal 3: DAD1 D, Sig=230,16 Ref=360,100

Peak #	RetTime [min]	Type	Width [min]	Area [mAU*s]	Height [mAU]	Area %
1	10.746	BB	0.3517	1.59138e4	654.36469	49.8789
2	13.272	BB	0.4983	1.59910e4	452.44550	50.1211

Totals : 3.19048e4 1106.81018

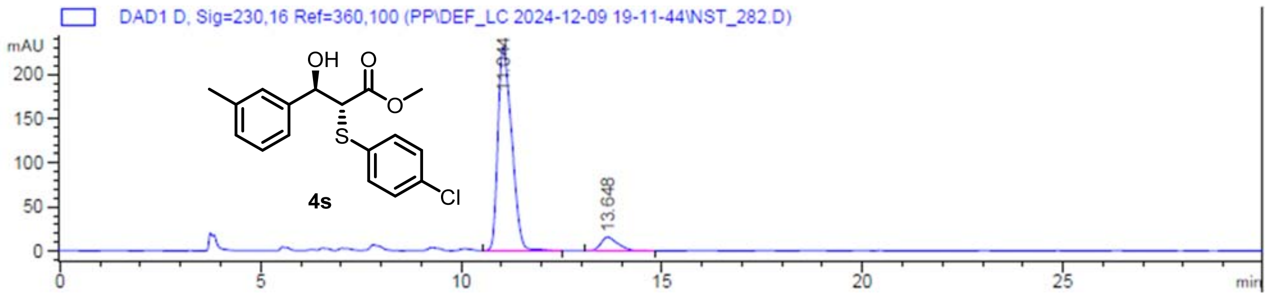

Signal 3: DAD1 D, Sig=230,16 Ref=360,100

Peak #	RetTime [min]	Type	Width [min]	Area [mAU*s]	Height [mAU]	Area %
1	11.044	BB	0.3902	5523.08887	231.62326	92.3414
2	13.648	BB	0.4294	458.07074	15.56057	7.6586

Totals : 5981.15961 247.18383

4t- Methyl (2*R*,3*R*)-2-((4-chlorophenyl)thio)-3-hydroxy-3-(4-methoxyphenyl)propanoate

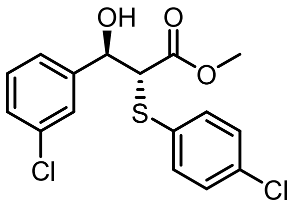

$C_{16}H_{14}Cl_2O_3S$

MW: 357.2 g/mol

Yield: 70%;

Colorless oil

The product (**4t**) was prepared using the general procedure **B** from **1a** (methyl 2-(dimethyl(oxo)-λ^6-sulfaneylidene)acetate), **2t** (3-chlorobenzaldehyde), and **3a** (4-chlorobenzenethiol).The product was obtained as a colorless oil (25 mg; 70% yield). The diastereomeric ratio obtained for the *anti*-couple is greater than 20:1. The enantiomeric excess of the product was determined by CSP HPLC: AD-H column, *n*-hexane/iPrOH 80:20, 0.75 mL/min, t_{maj} = 12.1 min; t_{min} = 14.0 min, 82% ee.

^{1}H NMR (600 MHz, $CDCl_3$) δ 7.36 – 7.32 (m, 1H), 7.30 – 7.24 (m, 2H), 7.24 – 7.17 (m, 5H), 4.97 (d, *J* = 8.0 Hz, 1H), 3.83 (d, *J* = 8.0 Hz, 1H), 3.69 (s, 3H).

^{13}C NMR (151 MHz, $CDCl_3$) δ 171.5, 142.0, 134.9, 134.6, 134.4, 131.1, 129.7, 129.3, 128.6, 127.1, 125.0, 73.9, 57.6, 52.6.

HRMS (MALDI$^+$) m/z calcd for $C_{16}H_{14}Cl_2O_3SNa^+$ $[M+Na]^+$: 378.9933, found: 378.9938.

$[\alpha]_D^{RT}$ = +41.2 (c = 1.0 g/100 mL, $CHCl_3$).

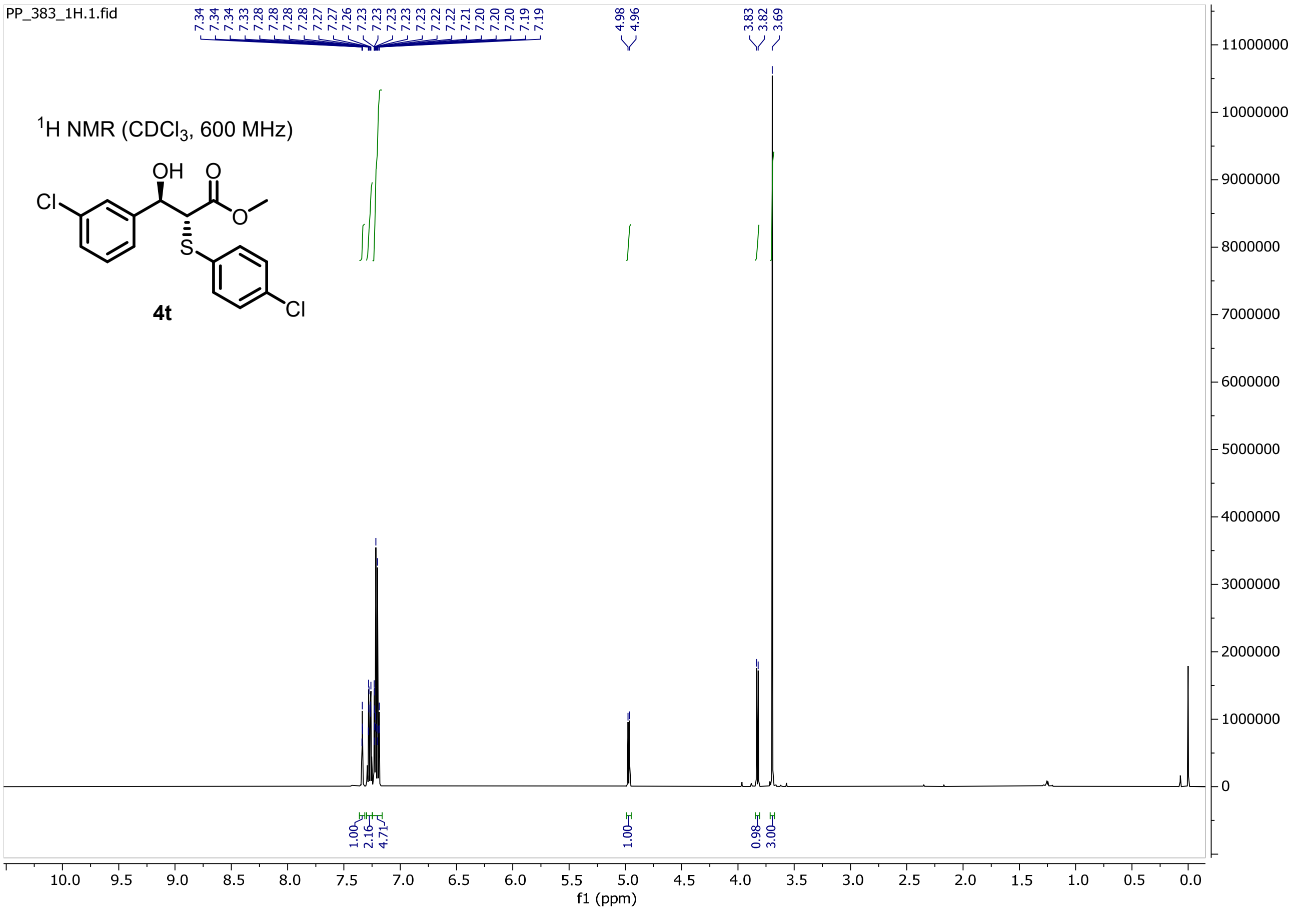
PP_383_1H.1.fid
7.34
7.34
7.34
7.33
7.28
7.28
7.28
7.28
7.27
7.27
7.26
7.23
7.23
7.23
7.23
7.23
7.22
7.22
7.21
7.20
7.20
7.20
7.19
7.19
4.98
4.96
3.83
3.82
3.69
^{1}H NMR (CDCl$_3$, 600 MHz)
OH
O
Cl
O
S
Cl
4t
1.00
2.16
4.71
1.00
0.98
3.00
f1 (ppm)

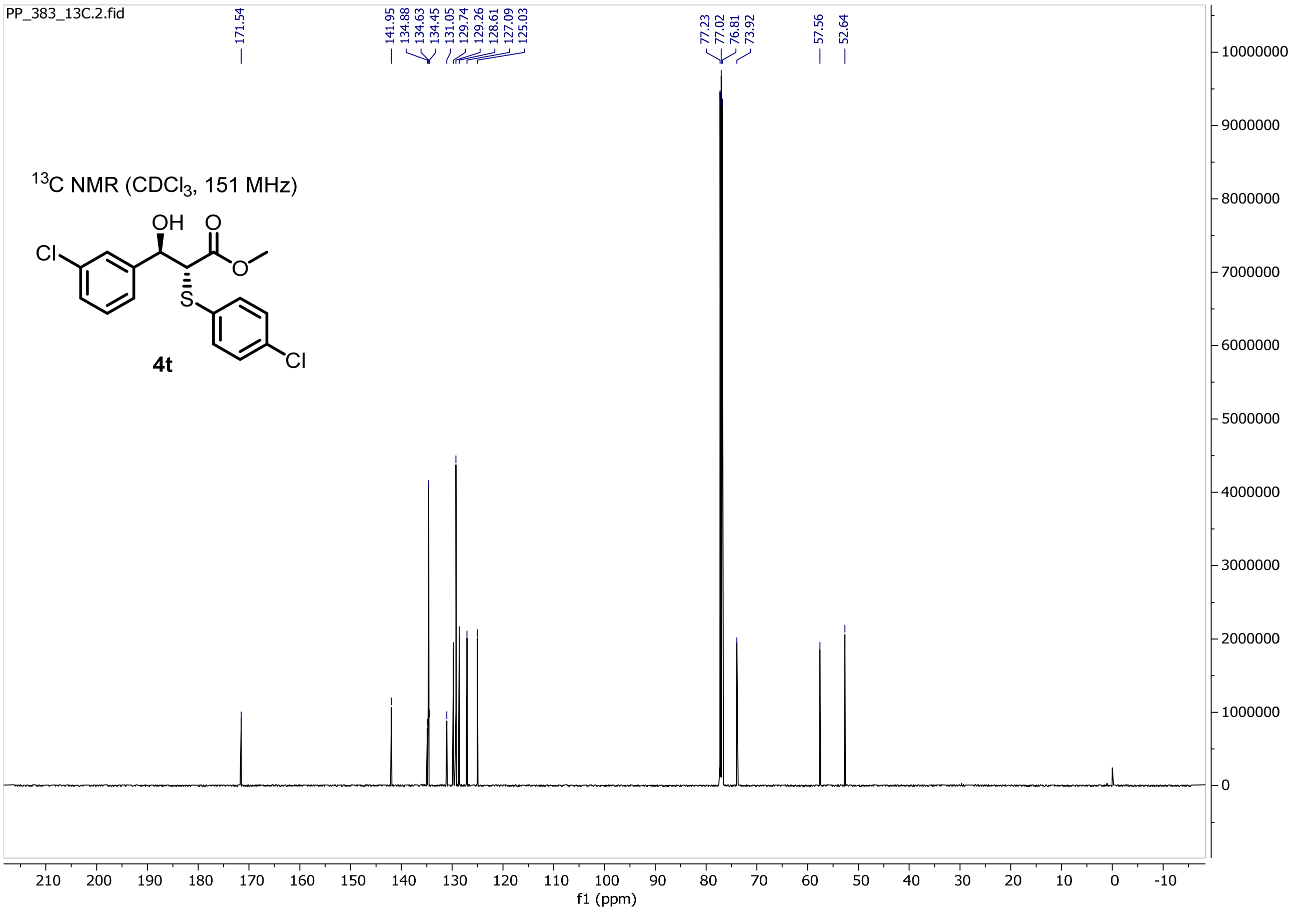
PP_383_13C.2.fid
171.54
141.95
134.88
134.63
134.45
131.05
129.74
129.26
128.61
127.09
125.03
77.23
77.02
76.81
73.92
57.56
52.64
^{13}C NMR (CDCl$_3$, 151 MHz)
OH
O
Cl
O
S
Cl
4t
f1 (ppm)

CSP HPLC traces:

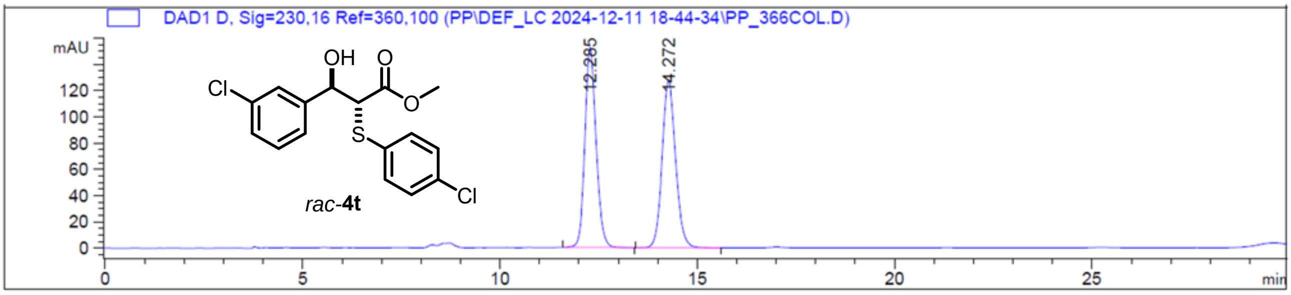

Signal 3: DAD1 D, Sig=230,16 Ref=360,100

Peak #	RetTime [min]	Type	Width [min]	Area [mAU*s]	Height [mAU]	Area %
1	12.285	BB	0.3024	3008.00610	152.28351	49.9482
2	14.272	BB	0.3626	3014.23975	127.13368	50.0518

Totals : 6022.24585 279.41719

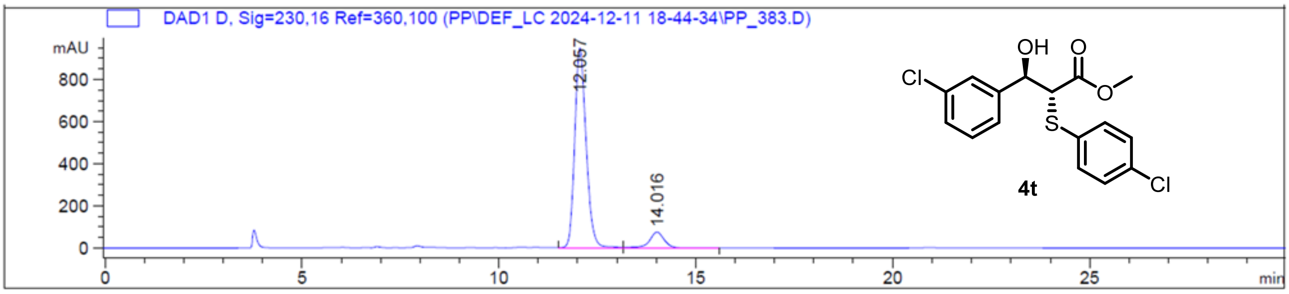

Signal 3: DAD1 D, Sig=230,16 Ref=360,100

Peak #	RetTime [min]	Type	Width [min]	Area [mAU*s]	Height [mAU]	Area %
1	12.057	VV	0.3086	1.90822e4	949.12561	90.8963
2	14.016	VB	0.3828	1911.16602	74.61333	9.1037

4u- Ethyl (2*R*,3*R*)-2-((4-chlorophenyl)thio)-3-hydroxy-3-phenylpropanoate

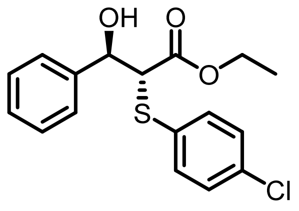

$C_{17}H_{17}ClO_3S$

MW: 380.8 g/mol

Yield: 74%;

Colorless oil

The product (**4u**) was prepared using the general procedure **C** from **1b** (ethyl 2-(dimethyl(oxo)-λ^6-sulfaneylidene)acetate), **2a** (benzaldehyde), and **3a** (4-chlorobenzenethiol). The product was obtained as a colorless oil (25 mg; 74% yield). The diastereomeric ratio obtained for the *anti*-couple is greater than 20:1. The enantiomeric excess of the product was determined by CSP HPLC: AD-H column, *n*-hexane/iPrOH 80:20, 0.75 mL/min, t_{maj} = 10.7 min; t_{min} = 20.4 min, 78% ee.

^{1}H NMR (600 MHz, $CDCl_3$) δ 7.37 – 7.30 (m, 5H), 7.20 (s, 4H), 5.01 (d, *J* = 7.9 Hz, 1H), 4.13 (q, *J* = 7.2, 2H), 3.87 (d, *J* = 7.9 Hz, 1H), 1.15 (t, *J* = 7.1 Hz, 3H).

^{13}C NMR (151 MHz, $CDCl_3$) δ 171.4, 140.0, 134.58, 134.56, 131.4, 129.1, 128.5, 128.4, 126.8, 74.4, 61.6, 57.7, 14.0.

HRMS (MALDI$^+$) m/z calcd for $C_{17}H_{17}ClO_3SNa^+$[M+Na]$^+$: 359.0479, found 359.0483.

$[\alpha]_D^{RT}$ = +35.1 (c = 1.0 g/100 mL, $CHCl_3$).

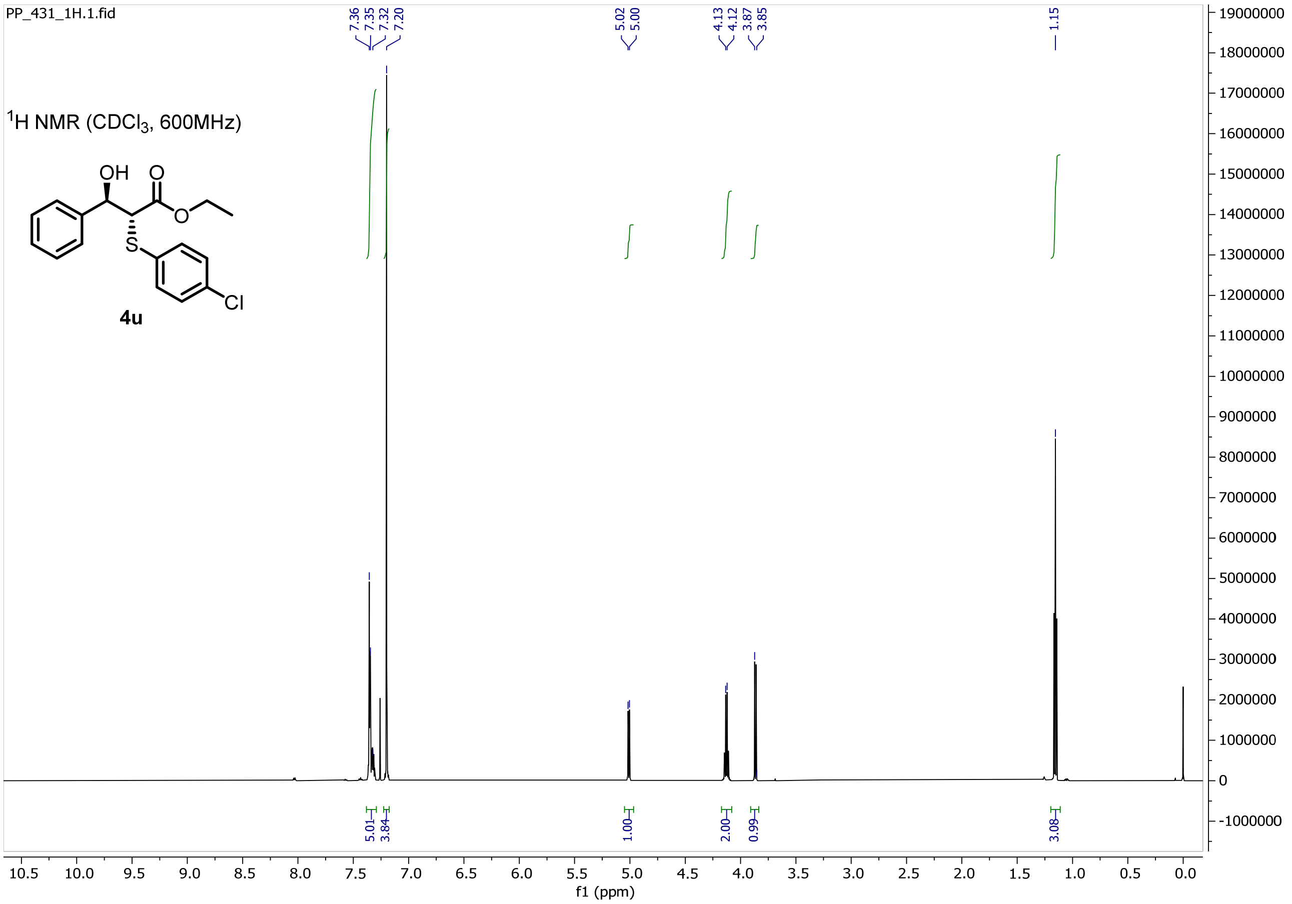
PP_431_1H.fid
1H NMR (CDCl3, 600MHz)
OH
O
O
S
Cl
4u
7.36
7.35
7.32
7.20
5.02
5.00
4.13
4.12
3.87
3.85
1.15
5.01
3.84
1.00
2.00
0.99
3.08
f1 (ppm)

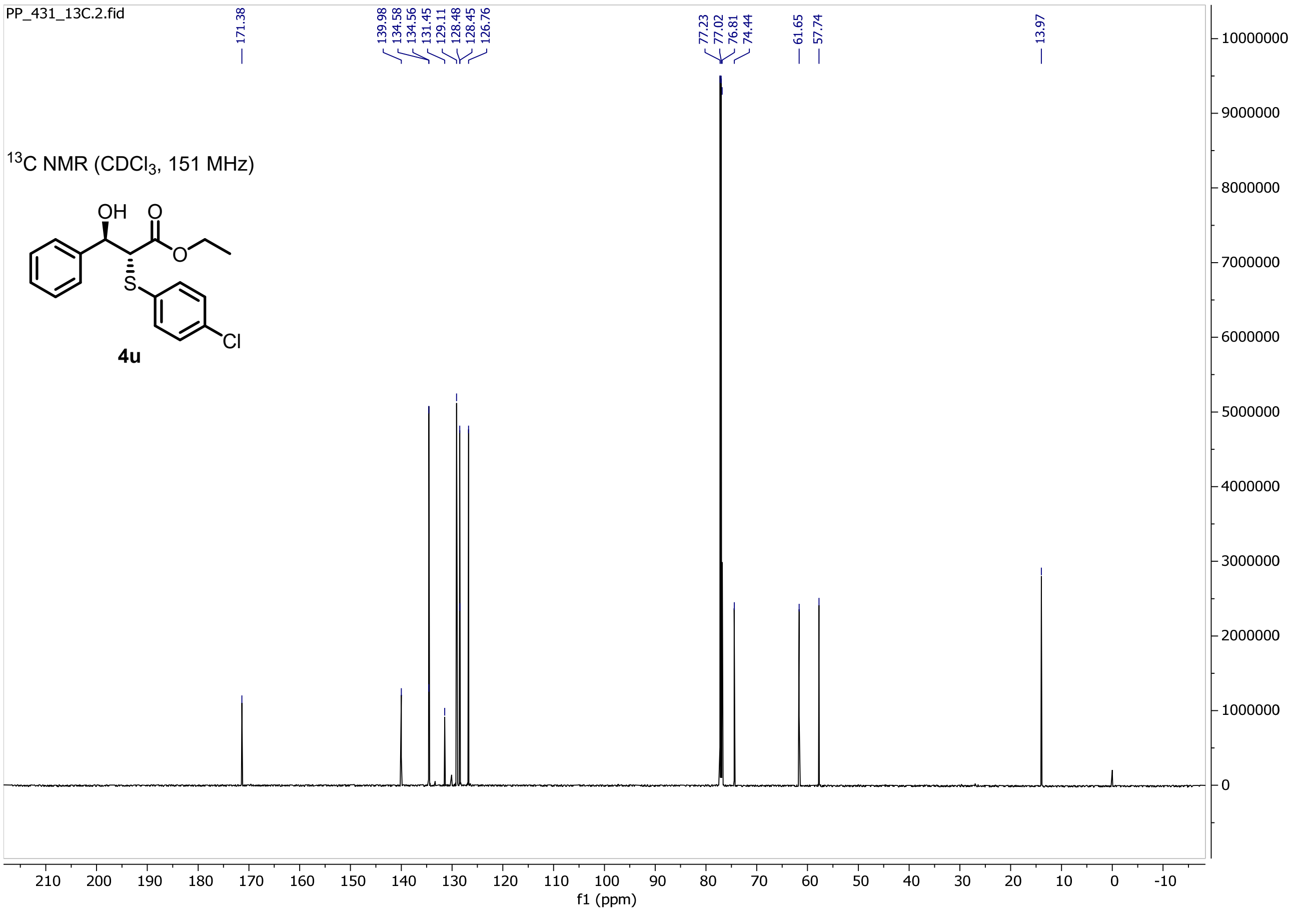
PP_431_13C.2.fid
13C NMR (CDCl3, 151 MHz)
OH
O
O
S
Cl
4u
171.38
139.98
134.58
134.56
131.45
129.11
128.48
128.45
126.76
77.23
77.02
76.81
74.44
61.65
57.74
13.97
f1 (ppm)

CSP HPLC traces:

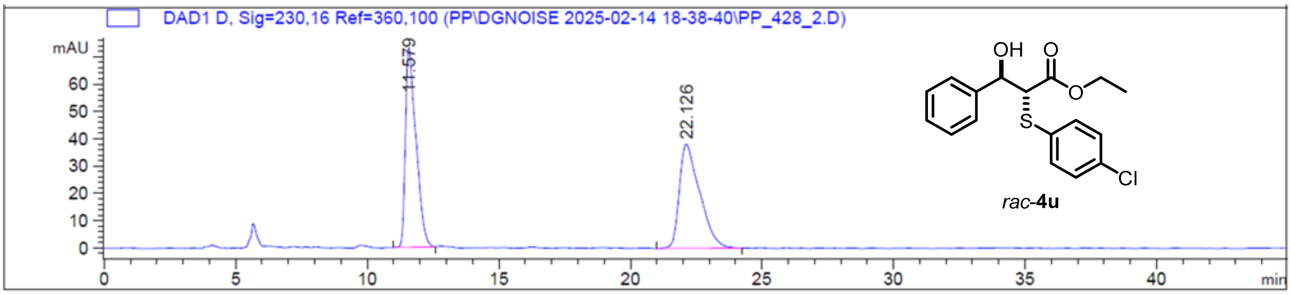

Signal 3: DAD1 D, Sig=230,16 Ref=360,100

Peak #	RetTime [min]	Type	Width [min]	Area [mAU*s]	Height [mAU]	Area %
1	11.579	BB	0.3981	2028.49451	73.01926	49.5926
2	22.126	BB	0.7682	2061.82593	38.17236	50.4074

Totals : 4090.32043 111.19161

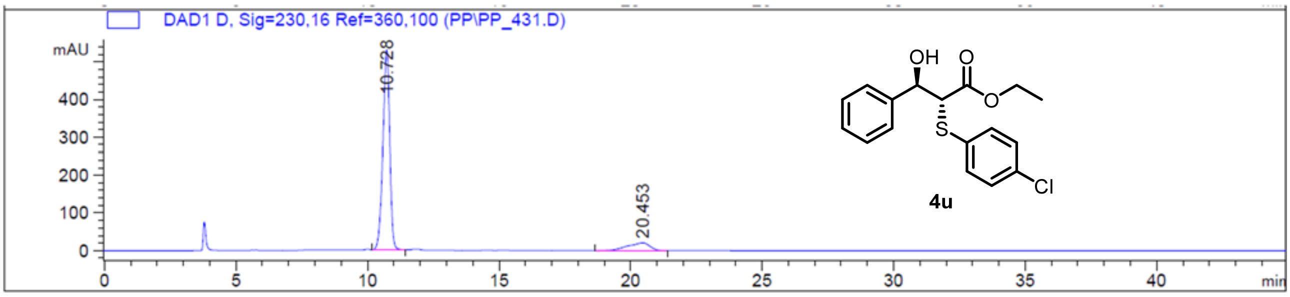

Signal 3: DAD1 D, Sig=230,16 Ref=360,100

Peak #	RetTime [min]	Type	Width [min]	Area [mAU*s]	Height [mAU]	Area %
1	10.728	VB	0.2765	9577.75781	530.76599	89.1955
2	20.453	BB	0.8017	1160.18176	20.27227	10.8045

Totals : 1.07379e4 551.03826

4v- Isobutyl (2*R*,3*R*)-2-((4-chlorophenyl)thio)-3-hydroxy-3-phenylpropanoate

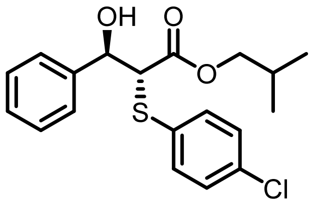

$C_{19}H_{21}ClO_3S$

MW: 364.1 g/mol

Yield: 69%;

Colorless oil

The product (**4v**) was prepared using the general procedure **C** from **1c** (isobutyl 2-(dimethyl(oxo)-λ^6-sulfaneylidene)acetate), **2a** (benzaldehyde), and **3a** (4-chlorobenzenethiol). The product was obtained as a colorless oil (25 mg; 69% yield). The diastereomeric ratio obtained for the *anti*-couple is greater than 20:1. The enantiomeric excess of the product was determined by CSP HPLC: AD-H column, *n*-hexane/iPrOH 80:20, 0.75 mL/min, t_{maj} = 9.9 min; t_{min} = 22.1 min, 79% ee.

^{1}H NMR (600 MHz, $CDCl_3$) δ 7.38 – 7.29 (m, 5H), 7.21 – 7.17 (m, 4H), 5.01 (d, *J* = 7.8 Hz, 1H), 3.89 (d, *J* = 7.8 Hz, 1H), 3.86 (dd, *J* = 10.6, 6.6 Hz, 1H), 3.83 (dd, *J* = 10.5, 6.7 Hz, 1H), 3.20 (br s, 1H), 1.86 – 1.78 (m, 1H), 0.83 (d, *J* = 6.7 Hz, 3H), 0.82 (d, *J* = 6.7 Hz, 3H).

^{13}C NMR (151 MHz, $CDCl_3$) δ 171.5, 140.0, 134.5, 134.4, 131.6, 129.1, 128.5, 128.4, 126.7, 74.4, 71.7, 57.8, 27.6, 18.9.

HRMS (MALDI$^+$) m/z calcd for $C_{17}H_{15}NO_3SNa^+[M+Na]^+$: 387.0792, found 387.0798.

$[\alpha]_D^{RT}$ = +29.7 (c = 1.0 g/100 mL, $CHCl_3$).

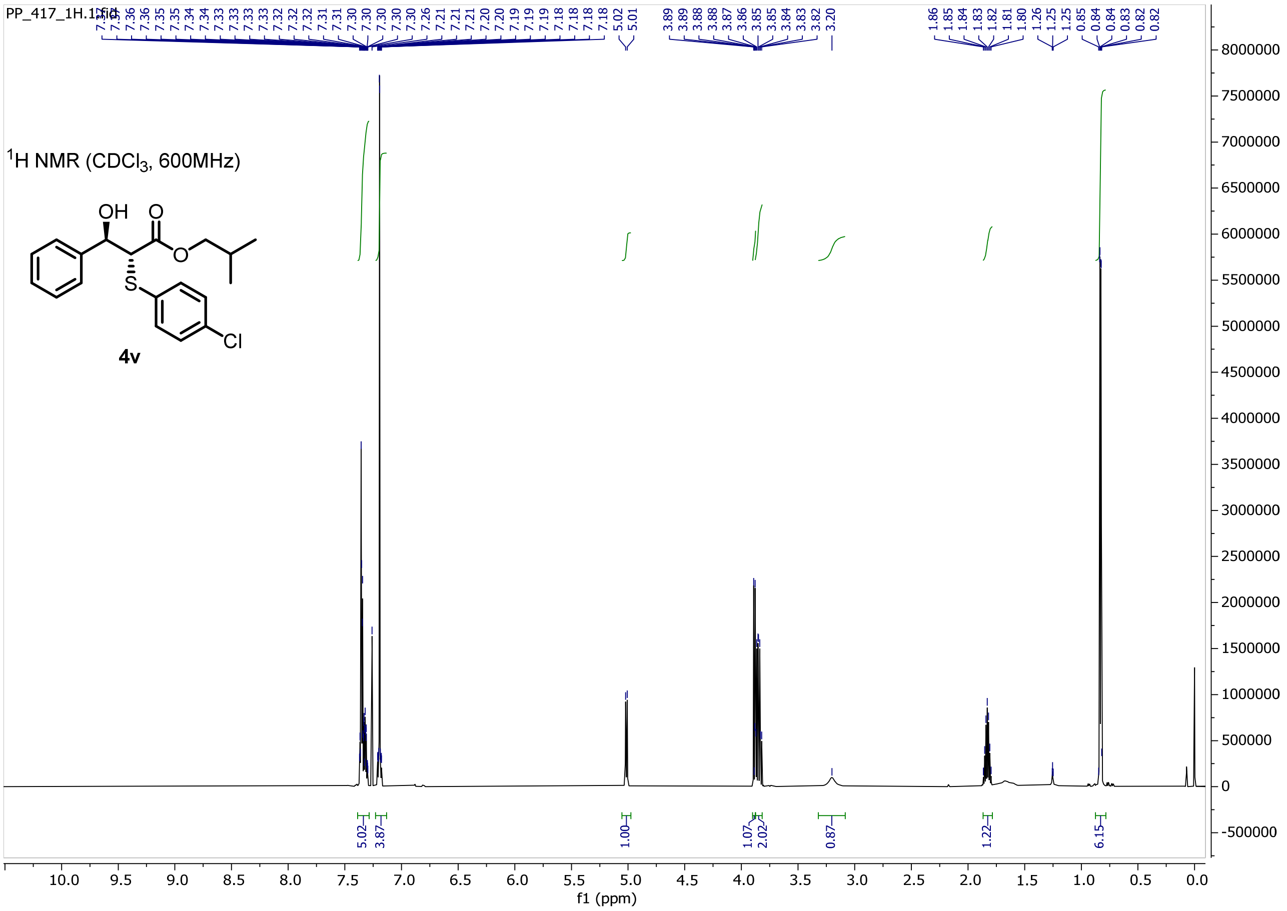
PP_417_1H.1.fid
^{1}H NMR (CDCl$_3$, 600MHz)
OH
O
O
S
Cl
4v
5.02
3.87
1.00
1.07
2.02
0.87
1.22
6.15
f1 (ppm)

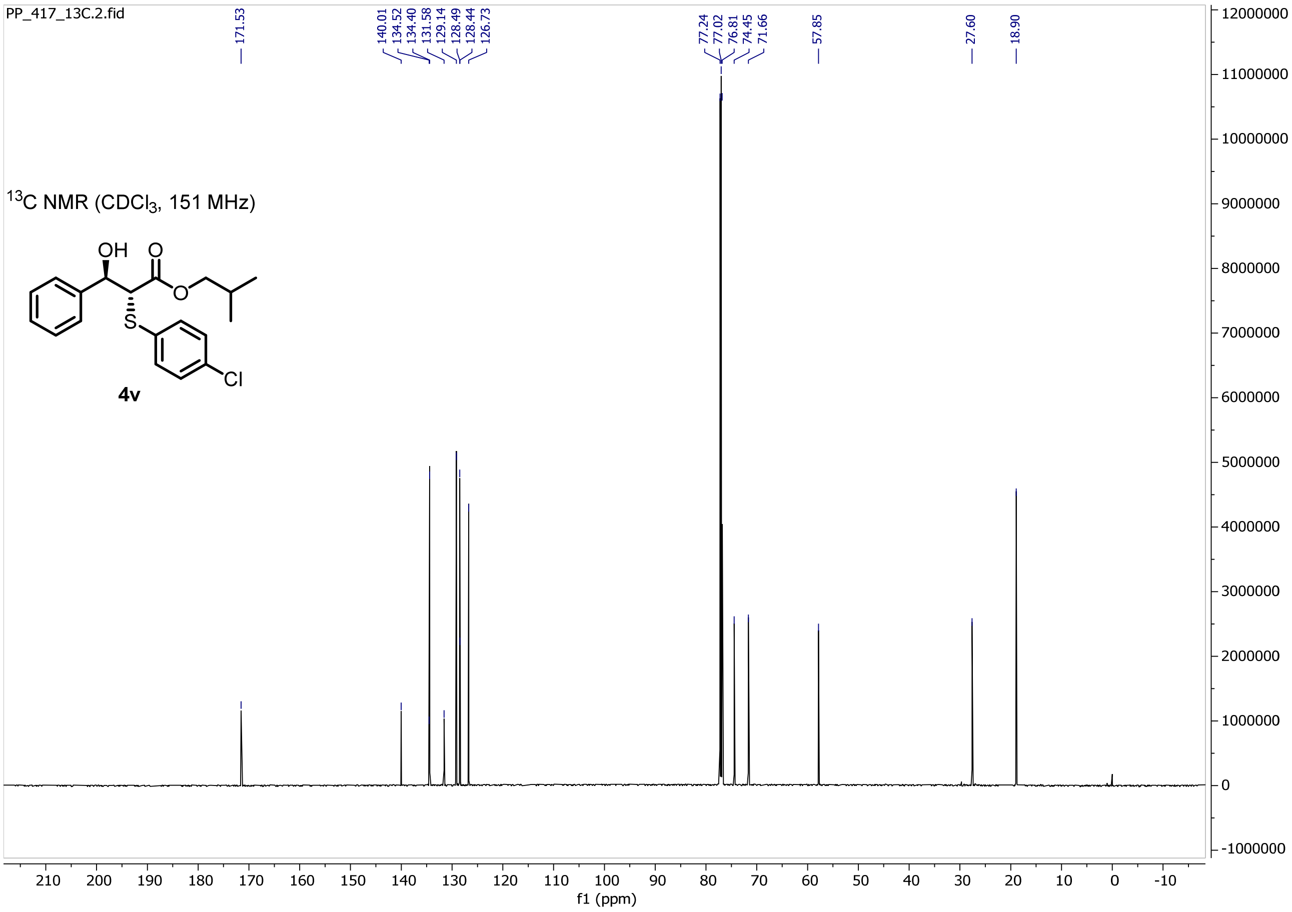
PP_417_13C.2.fid
171.53
140.01
134.52
134.40
131.58
129.14
128.49
128.44
126.73
77.24
77.02
76.81
74.45
71.66
57.85
27.60
18.90
^{13}C NMR (CDCl$_3$, 151 MHz)
OH
O
O
S
Cl
4v
f1 (ppm)

CSP HPLC traces:

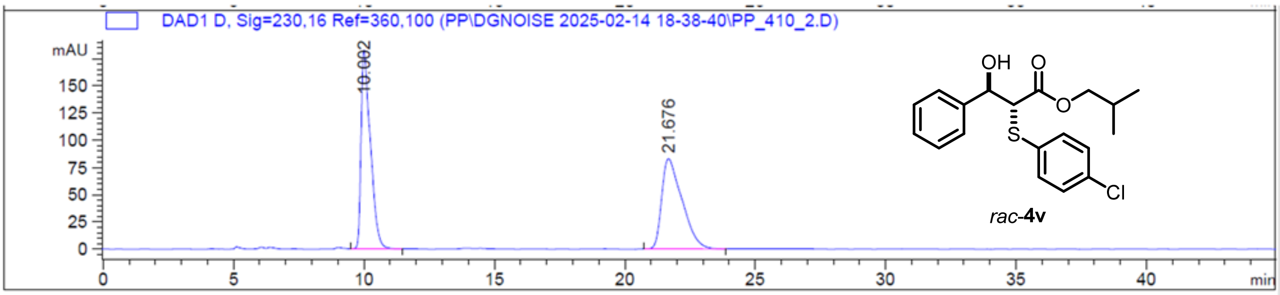

Signal 3: DAD1 D, Sig=230,16 Ref=360,100

Peak #	RetTime [min]	Type	Width [min]	Area [mAU*s]	Height [mAU]	Area %
1	10.002	BB	0.3547	4520.29395	182.68576	50.1226
2	21.676	BB	0.7771	4498.17725	83.15192	49.8774
Totals :				9018.47119	265.83768	

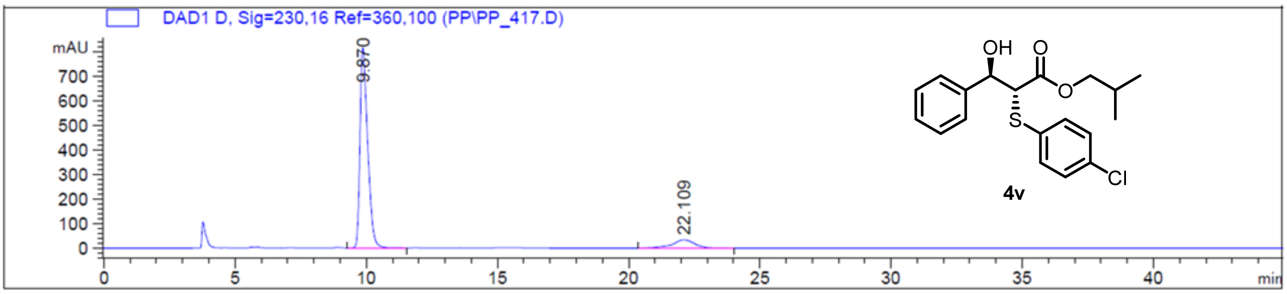

Signal 3: DAD1 D, Sig=230,16 Ref=360,100

Peak #	RetTime [min]	Type	Width [min]	Area [mAU*s]	Height [mAU]	Area %
1	9.870	BB	0.2975	1.65040e4	817.77826	89.6371
2	22.109	BB	0.8645	1908.01208	33.00026	10.3629
Totals :				1.84120e4	850.77851	

4w- Allyl (2*R*,3*R*)-2-((4-chlorophenyl)thio)-3-hydroxy-3-phenylpropanoate

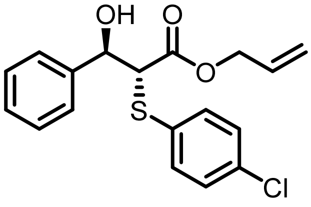

$C_{18}H_{17}ClO_3S$

MW: 348.1 g/mol

Yield: 86%;

Colorless oil

The product (**4w**) was prepared using the general procedure **C** from **1d** (allyl 2-(dimethyl(oxo)-λ^6-sulfaneylidene)acetate), **2a** (benzaldehyde), and **3a** (4-chlorobenzenethiol). The product was obtained as a colorless oil (30 mg; 86% yield). The diastereomeric ratio obtained for the *anti*-couple is greater than 20:1. The enantiomeric excess of the product was determined by CSP HPLC: AD-H column, *n*-hexane/iPrOH 80:20, 0.75 mL/min, t_{maj} = 10.9 min; t_{min} = 22.1 min, 80% ee.

^{1}H NMR (600 MHz, $CDCl_3$) δ 7.38 – 7.30 (m, 5H), 7.22 – 7.15 (m, 4H), 5.78 (ddt, *J* = 17.2, 10.4, 5.8 Hz, 1H), 5.28 – 5.18 (m, 2H), 5.02 (d, *J* = 8.1 Hz, 1H), 4.60 – 4.54 (m, 2H), 3.89 (d, *J* = 8.1 Hz, 1H), 3.10 (br s, 1H).

^{13}C NMR (151 MHz, $CDCl_3$) δ 171.0, 139.9, 134.68, 134.66, 131.32, 131.28, 129.1, 128.5, 126.8, 118.9, 74.5, 66.1, 57.8.

HRMS (MALDI$^+$) m/z calcd for $C_{17}H_{15}ClO_3SNa^+[M+Na]^+$: 371.0479, found: 371.0485.

$[\alpha]_D^{RT}$ = +35.4 (c = 1.0 g/100 mL, $CHCl_3$).

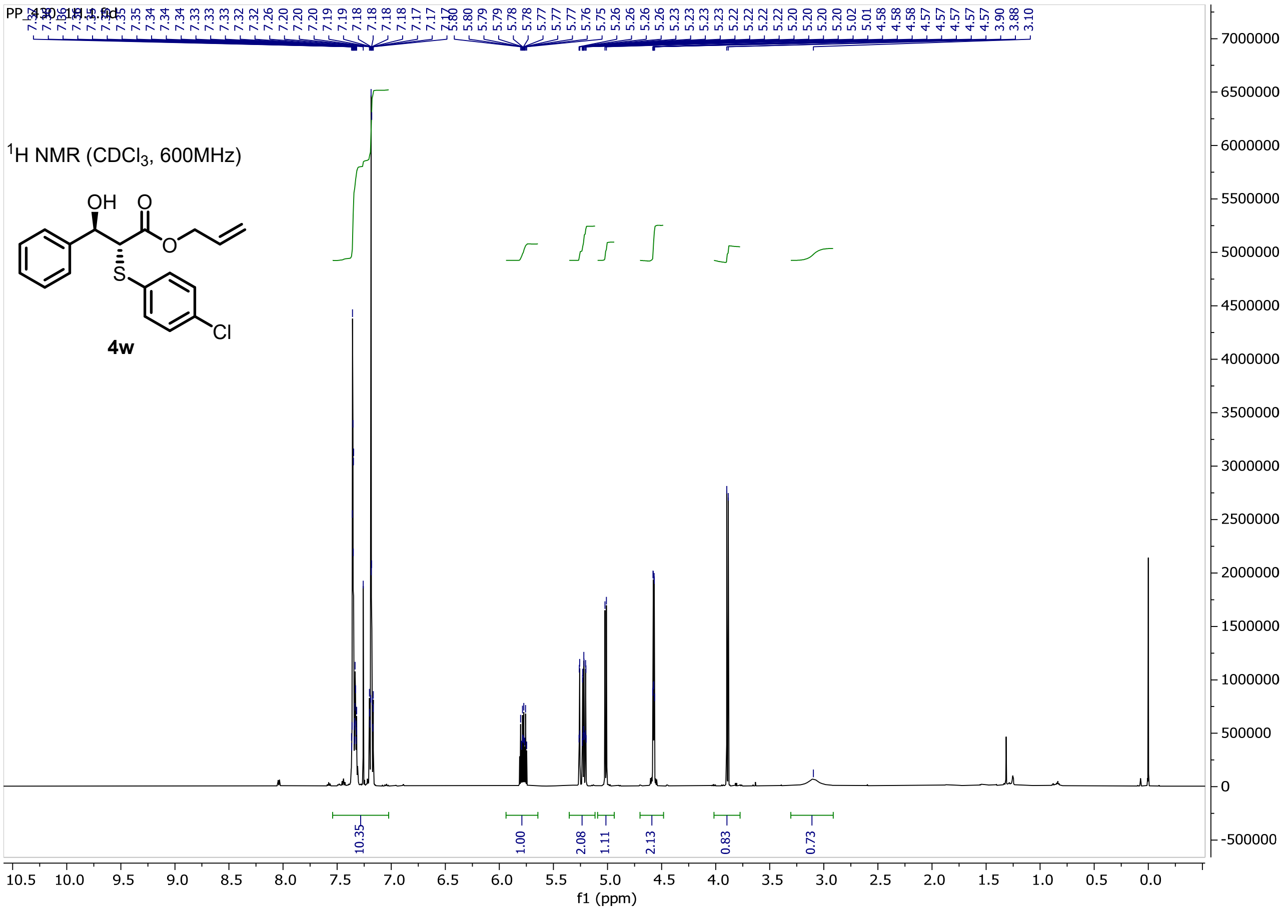

¹H NMR (CDCl₃, 600MHz)
4w
f1 (ppm)

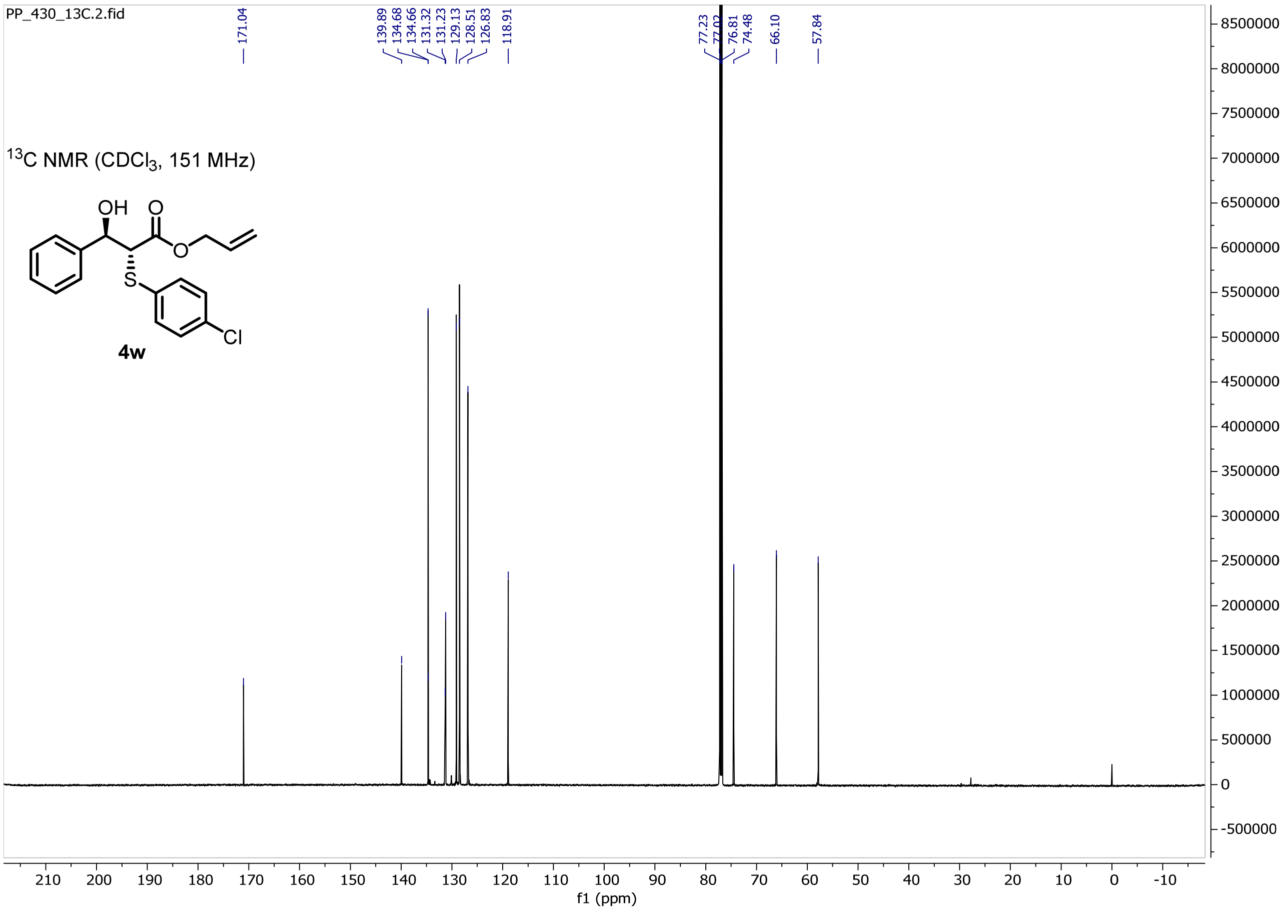

PP_430_13C.2.fid
171.04
139.89
134.68
134.66
131.32
131.23
129.13
128.51
126.83
118.91
77.23
77.02
76.81
74.48
66.10
57.84
¹³C NMR (CDCl₃, 151 MHz)
4w
f1 (ppm)

CSP HPLC traces:

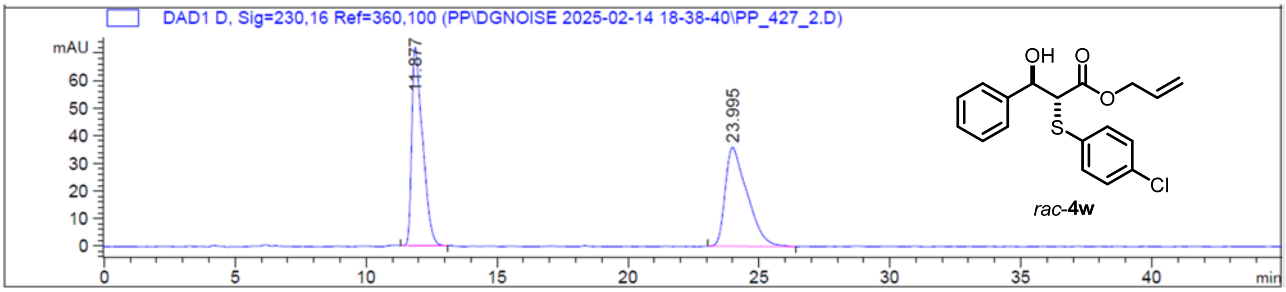

Signal 3: DAD1 D, Sig=230,16 Ref=360,100

Peak #	RetTime [min]	Type	Width [min]	Area [mAU*s]	Height [mAU]	Area %
1	11.877	BB	0.4105	2064.31738	72.00672	49.6091
2	23.995	BB	0.8435	2096.84985	35.78910	50.3909
Totals :				4161.16724	107.79582	

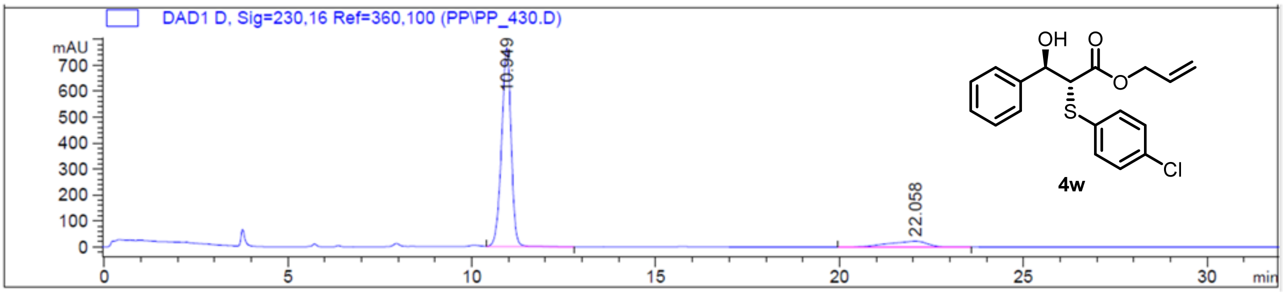

Signal 3: DAD1 D, Sig=230,16 Ref=360,100

Peak #	RetTime [min]	Type	Width [min]	Area [mAU*s]	Height [mAU]	Area %
1	10.949	VB	0.2751	1.38901e4	767.48932	90.1710
2	22.058	BB	0.9593	1514.08276	21.82715	9.8290
Totals :				1.54042e4	789.31647	

4x- Benzyl (2*R*,3*R*)-2-((4-chlorophenyl)thio)-3-hydroxy-3-phenylpropanoate

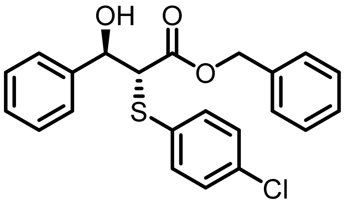

$C_{22}H_{19}ClO_3S$

MW: 398.1 g/mol

Yield: 88%;

Colorless oil

The product (**4x**) was prepared using the general procedure **C** from **1e** (benzyl 2-(dimethyl(oxo)-λ^6-sulfaneylidene)acetate), **2a** (benzaldehyde), and **3a** (4-chlorobenzenethiol). The product was obtained as a colorless oil (35 mg; 88% yield). The diastereomeric ratio obtained for the *anti*-couple is greater than 20:1. The enantiomeric excess of the product was determined by CSP HPLC: AD-H column, *n*-hexane/iPrOH 80:20, 0.75 mL/min, t_{maj} = 15.0 min; t_{min} = 33.1 min, 80% ee.

^{1}H NMR (600 MHz, $CDCl_3$) δ 7.37 – 7.30 (m, 8H), 7.21 – 7.16 (m, 2H), 7.15 – 7.09 (m, 4H), 5.14 (d, *J* = 12.2 Hz, 1H), 5.05 (d, *J* = 12.2 Hz, 1H), 5.02 (d, *J* = 7.9 Hz, 1H), 3.92 (d, *J* = 7.9 Hz, 1H), 3.13 (br s, 1H).

^{13}C NMR (151 MHz, $CDCl_3$) δ 171.2, 139.9, 135.0, 134.7, 134.6, 131.1, 129.1, 128.56, 128.53, 128.49, 128.45, 128.3, 74.4, 67.2, 57.7.

HRMS (MALDI$^+$) m/z calcd for $C_{22}H_{19}ClO_3SNa^+[M+Na]^+$: 421.0636, found: 421.0643.

$[\alpha]_D^{RT}$ = +21.8 (c = 1.0 g/100 mL, $CHCl_3$).

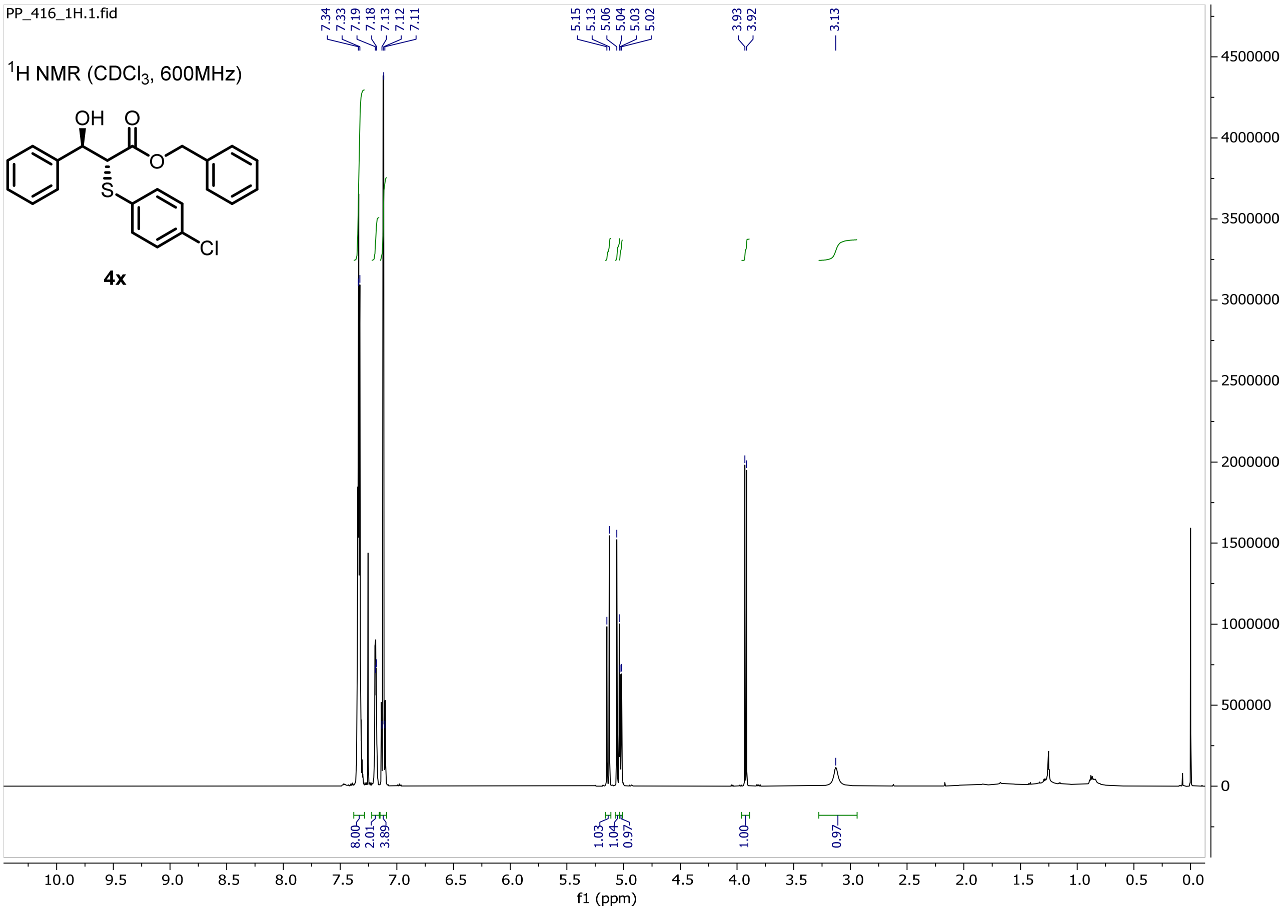
PP_416_1H.1.fid
1H NMR (CDCl3, 600MHz)
4x
f1 (ppm)

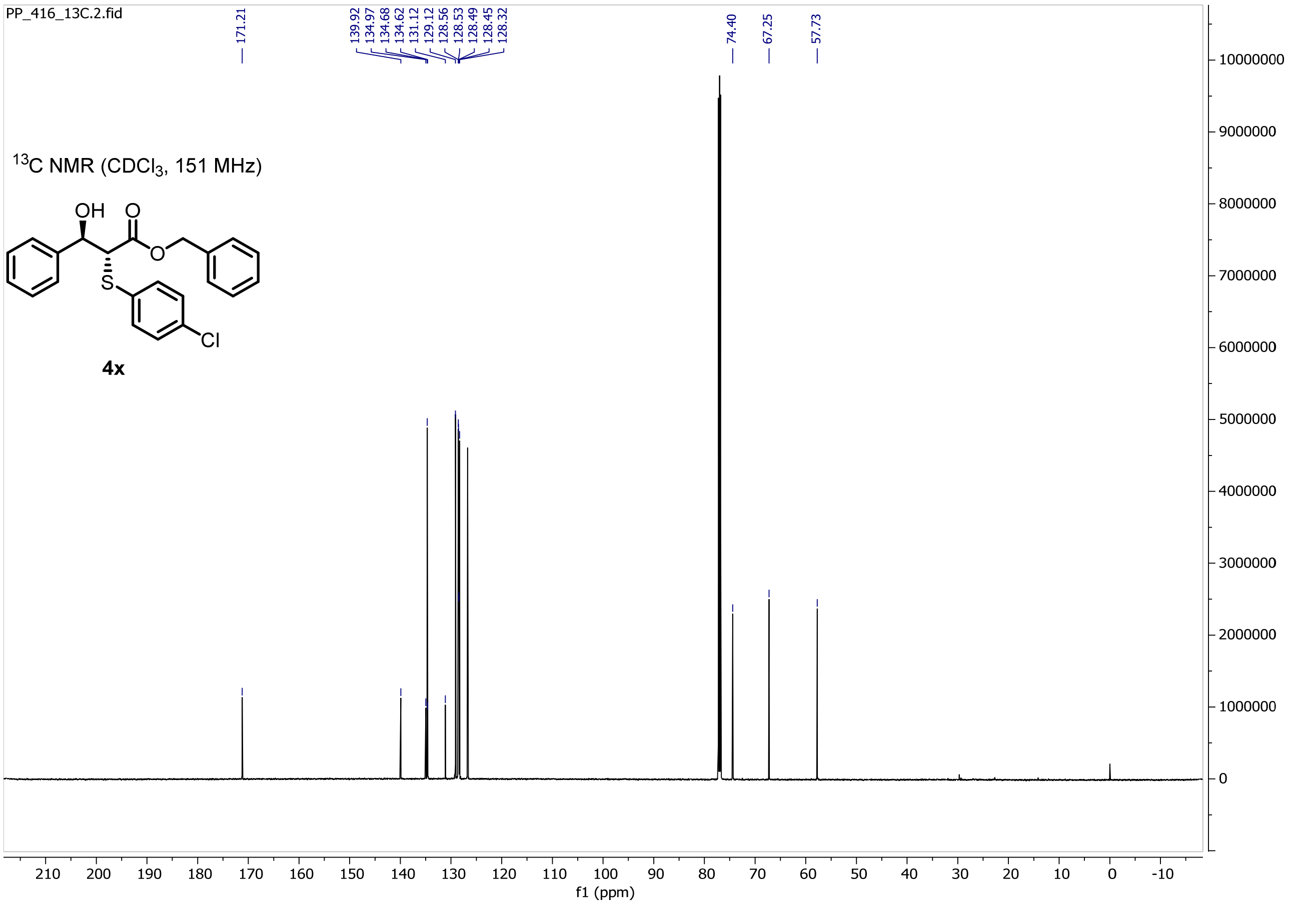
PP_416_13C.2.fid
13C NMR (CDCl3, 151 MHz)
4x
f1 (ppm)

CSP HPLC traces:

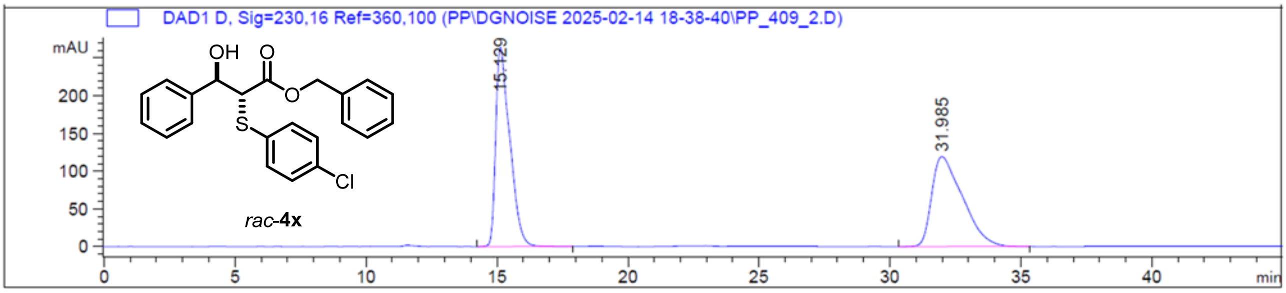

Signal 3: DAD1 D, Sig=230,16 Ref=360,100

Peak #	RetTime [min]	Type	Width [min]	Area [mAU*s]	Height [mAU]	Area %
1	15.129	BB	0.5335	9845.02246	263.74899	50.1487
2	31.985	BB	1.1742	9786.63477	119.15659	49.8513

Totals : 1.96317e4 382.90558

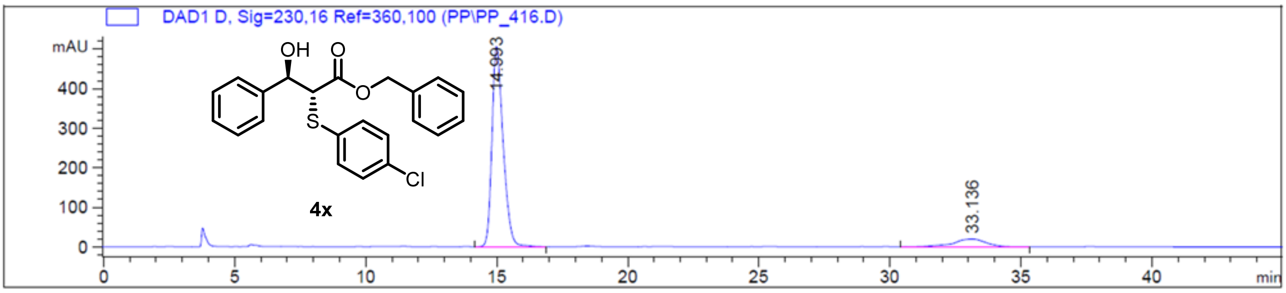

Signal 3: DAD1 D, Sig=230,16 Ref=360,100

Peak #	RetTime [min]	Type	Width [min]	Area [mAU*s]	Height [mAU]	Area %
1	14.993	BB	0.4439	1.50032e4	505.97562	89.7840
2	33.136	BB	1.1588	1707.13501	19.66477	10.2160

Totals : 1.67103e4 525.64039

4y- Methyl (2*R*,3*R*)-2-((4-cyanophenyl)thio)-3-hydroxy-3-phenylpropanoate

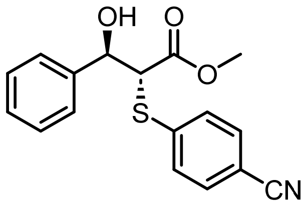

$C_{17}H_{15}NO_3S$

MW: 313.4 g/mol

Yield: 50%;

Colorless oil

The product (**4y**) was prepared using the general procedure **B** from **1a** (methyl 2-(dimethyl(oxo)-λ^6-sulfaneylidene)acetate), **2a** (benzaldehyde), and **3b** (4-mercaptobenzonitrile). The product was obtained as a colorless oil (16 mg; 50% yield). The diastereomeric ratio obtained for the *anti*-couple is greater than 20:1. The enantiomeric excess of the product was determined by CSP HPLC: AD-H column, *n*-hexane/iPrOH 80:20, 0.75 mL/min, t_{maj} = 25.0 min; t_{min} = 44.0 min, 97% ee.

^{1}H NMR (600 MHz, CDCl3) δ 7.49 – 7.44 (m, 2H), 7.38 – 7.30 (m, 5H), 7.30 – 7.24 (m, 2H), 5.07 (d, *J* = 8.2 Hz, 1H), 4.04 (d, *J* = 8.3 Hz, 1H), 3.73 (s, 3H), 3.05 (br s, 1H).

^{13}C NMR (151 MHz, $CDCl_3$) δ 171.4, 140.8, 139.5, 132.3, 130.6, 128.7, 128.6, 126.7, 118.4, 110.6, 74.7, 56.3, 52.8.

HRMS (MALDI$^+$) m/z calcd for $C_{17}H_{15}NO_3SNa^+[M+Na]^+$:336.0665, found: 336.0670.

$[\alpha]_D^{RT}$ = +31.0 (c = 1.0 g/100 mL, $CHCl_3$).

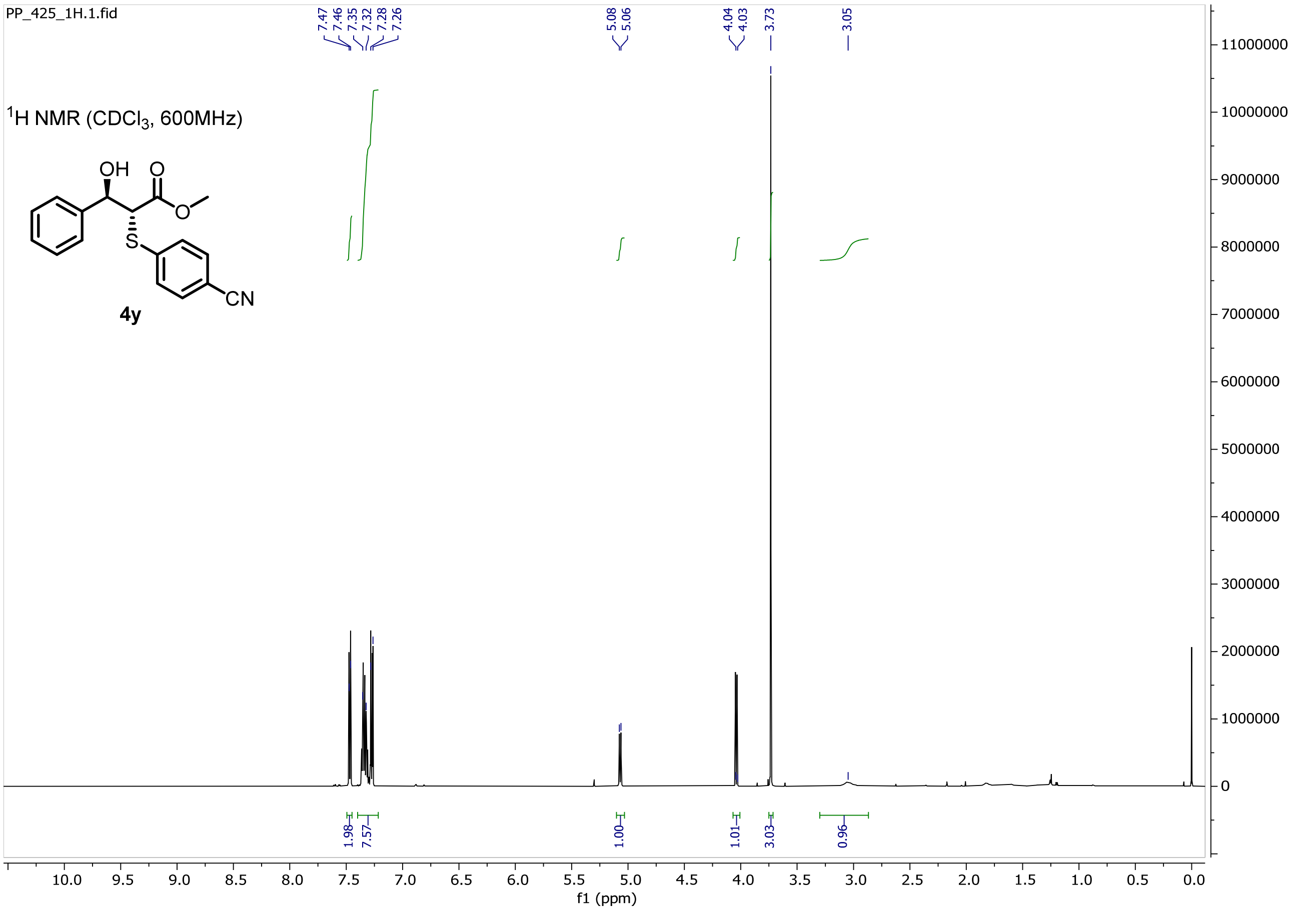
PP_425_1H.1.fid
7.47
7.46
7.35
7.32
7.28
7.26
5.08
5.06
4.04
4.03
3.73
3.05
^1H NMR (CDCl$_3$, 600MHz)
OH
O
O
S
CN
4y
1.98
7.57
1.00
1.01
3.03
0.96
f1 (ppm)

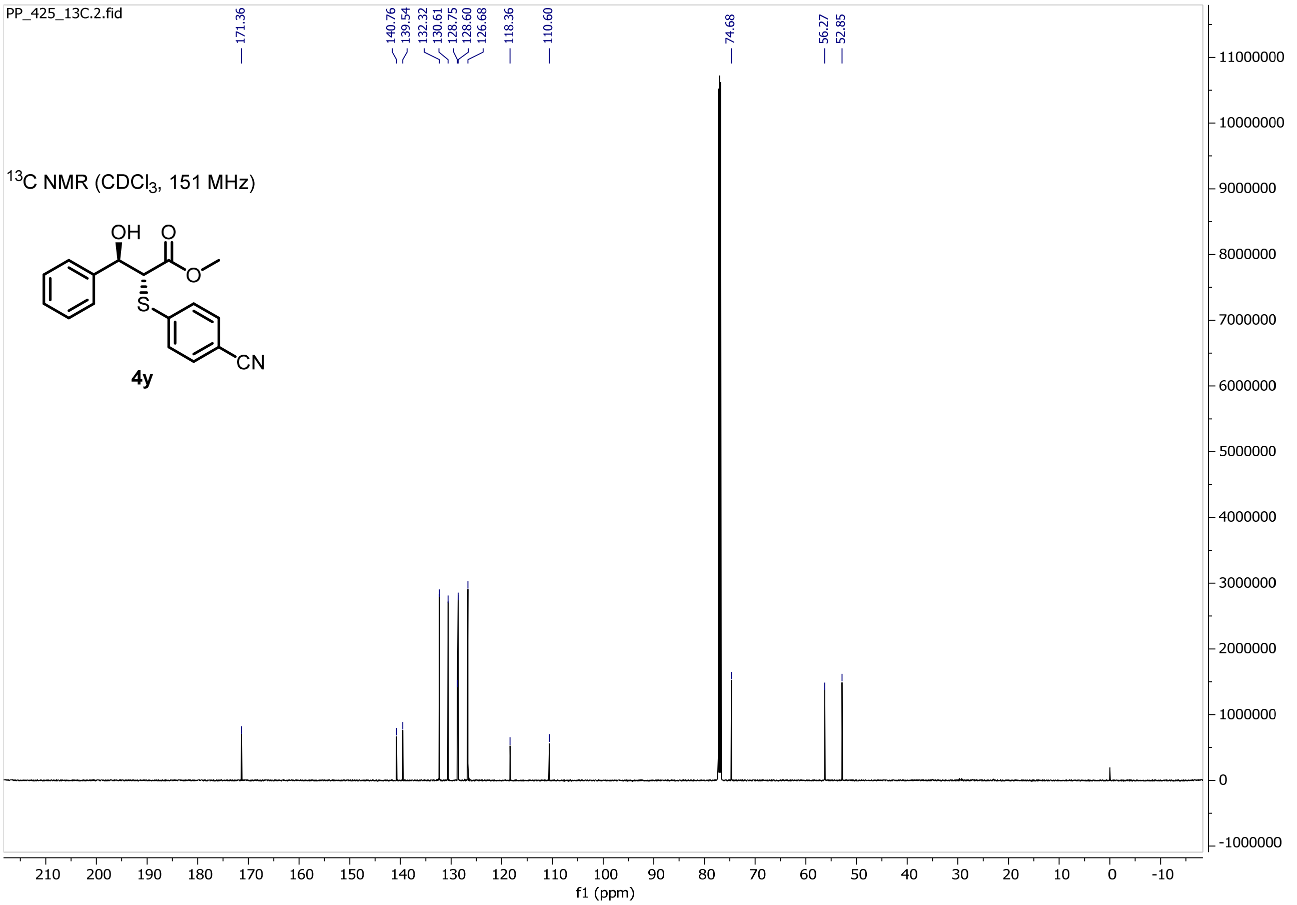
PP_425_13C.2.fid
171.36
140.76
139.54
132.32
130.61
128.75
128.60
126.68
118.36
110.60
74.68
56.27
52.85
^{13}C NMR (CDCl$_3$, 151 MHz)
OH
O
O
S
CN
4y
f1 (ppm)

CSP HPLC traces:

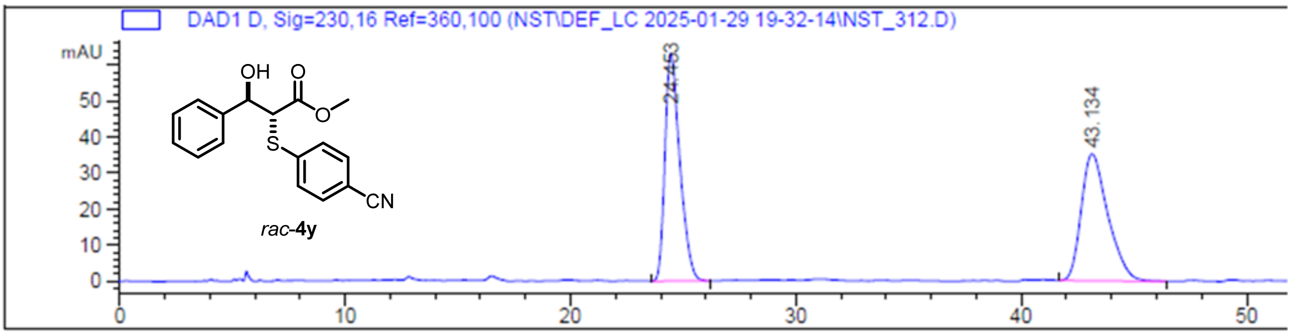

Signal 3: DAD1 D, Sig=230,16 Ref=360,100

Peak #	RetTime [min]	Type	Width [min]	Area [mAU*s]	Height [mAU]	Area %
1	24.453	BB	0.6837	2899.81299	63.38597	49.9110
2	43.134	BB	1.1942	2910.15503	35.28648	50.0890

Totals : 5809.96802 98.67245

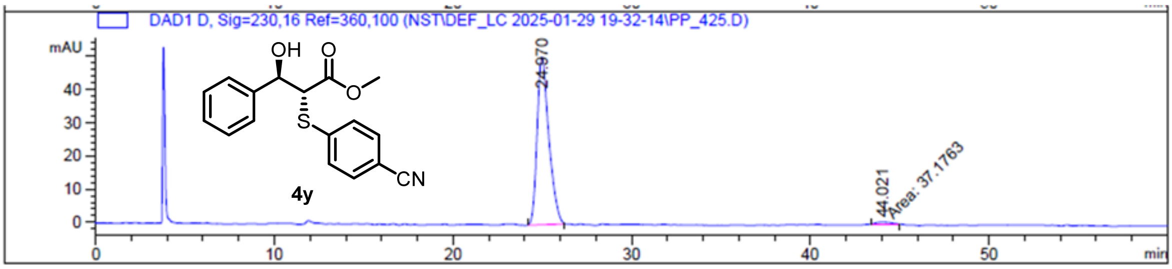

Signal 3: DAD1 D, Sig=230,16 Ref=360,100

Peak #	RetTime [min]	Type	Width [min]	Area [mAU*s]	Height [mAU]	Area %
1	24.970	BB	0.6807	2301.30273	50.39983	98.4102
2	44.021	MM T	0.9446	37.17625	6.55923e-1	1.5898

Totals : 2338.47898 51.05575

4z- Methyl (2*R*,3*R*)-3-hydroxy-3-phenyl-2-((4-(trifluoromethyl)phenyl)thio)propanoate

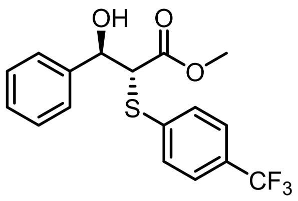

$C_{17}H_{15}F_3O_3S$

MW: 356.3 g/mol

Yield: 49%;

White solid

The product (**4z**) was prepared using the general procedure **C** from **1a** (methyl 2-(dimethyl(oxo)-λ^6-sulfaneylidene)acetate), **2a** (benzaldehyde), and **3c** (4-(trifluoromethyl)benzenethiol). The product was obtained as a white solid (17.5 mg; 49% yield). The diastereomeric ratio obtained for the *anti*-couple is greater than 20:1. The enantiomeric excess of the product was determined by CSP HPLC: AD-H column, *n*-hexane/iPrOH 80:20, 0.75 mL/min, t_{maj} = 11.5 min; t_{min} = 16.1 min, 77% ee.

^{1}H NMR (600 MHz, $CDCl_3$) δ 7.45 (d, *J* = 8.1 Hz, 2H), 7.38 – 7.28 (m, 7H), 5.05 (d, *J* = 8.2 Hz, 1H), 4.00 (d, *J* = 8.2 Hz, 1H), 3.72 (s, 3H).

^{13}C NMR (151 MHz, $CDCl_3$) δ 171.6, 139.7, 138.4, 131.6, 129.6 (q, *J* = 32.7 Hz), 128.7, 128.6, 126.7, 125.7 (q, *J* = 3.8 Hz), 123.8 (q, *J* = 272.3 Hz), 74.7, 56.8, 52.7.

^{19}F NMR (376 MHz, $CDCl_3$) δ -62.79 (s, 3F).

HRMS (MALDI$^+$) m/z calcd for $C_{17}H_{15}F_3O_3SNa^+$[M+Na]$^+$:379.0586, found 379.0592.

$[\alpha]_D^{RT}$ = +23.4 (c = 1.0 g/100 mL, $CHCl_3$).

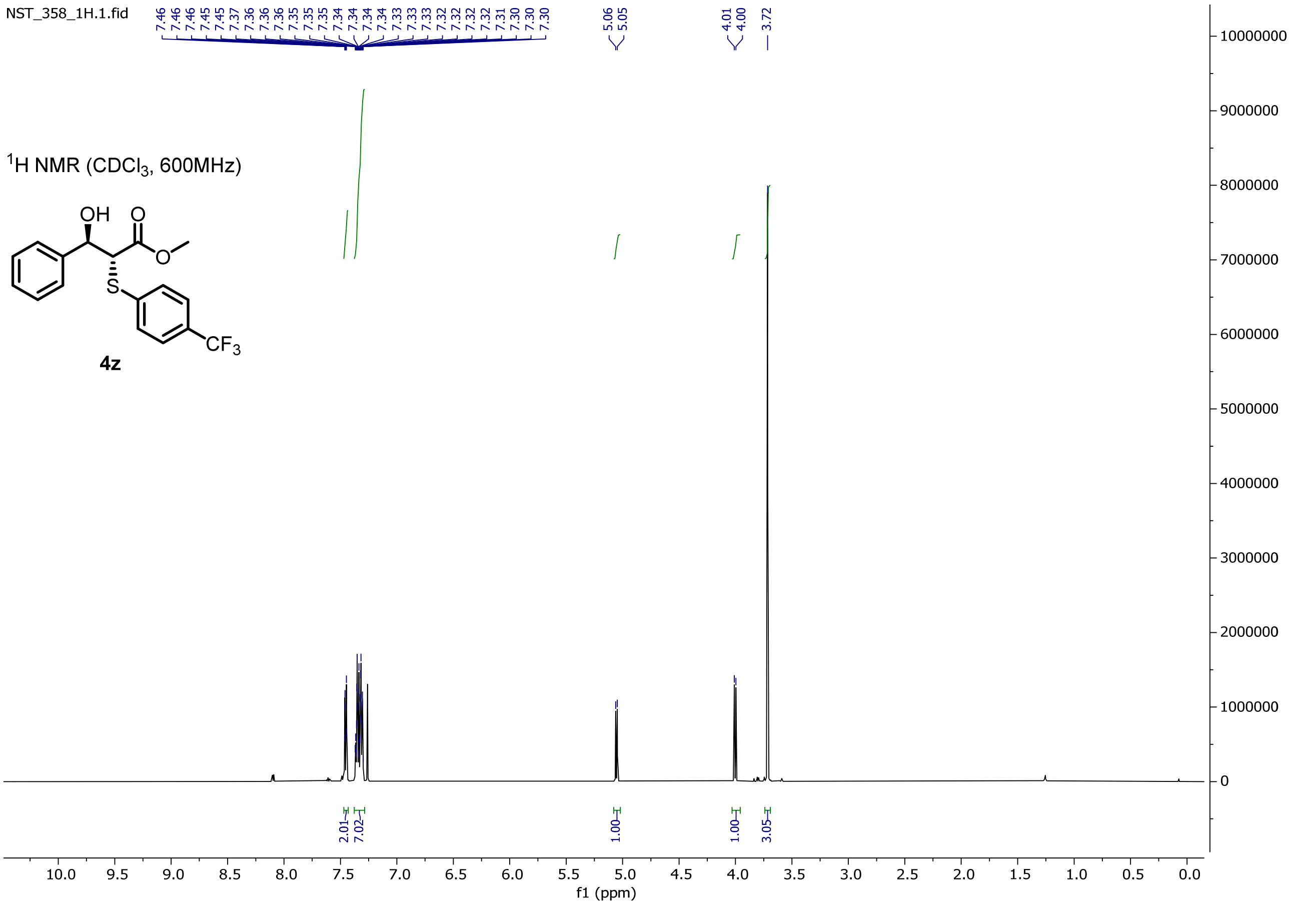
NST_358_1H.1.fid
7.46 7.46 7.46 7.45 7.45 7.37 7.36 7.36 7.36 7.35 7.35 7.35 7.34 7.34 7.34 7.34 7.33 7.33 7.33 7.32 7.32 7.32 7.32 7.31 7.30 7.30 7.30
5.06 5.05
4.01 4.00
3.72
^{1}H NMR (CDCl$_3$, 600MHz)
OH
O
O
S
CF$_3$
4z
2.01 7.02 1.00 1.00 3.05
f1 (ppm)

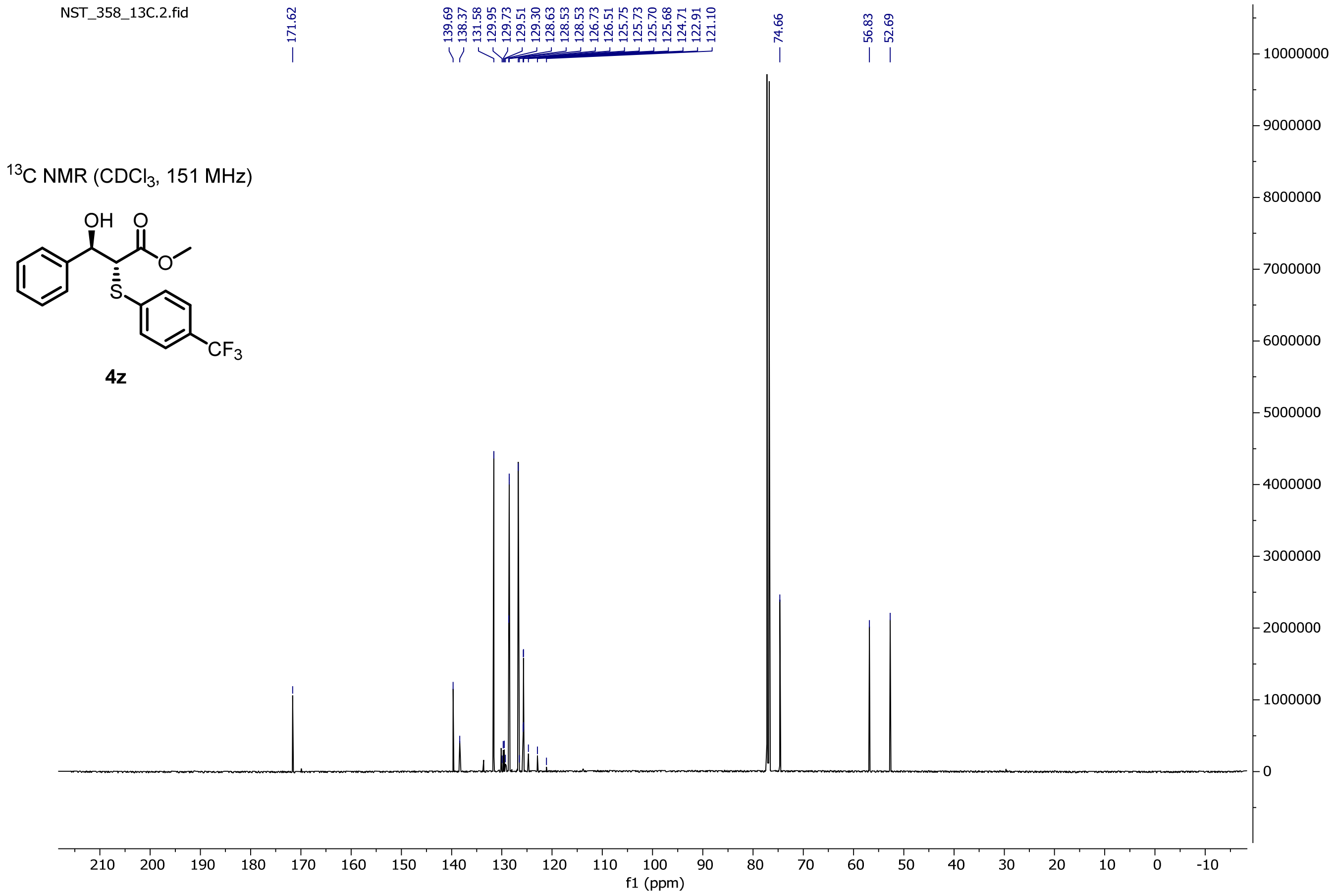
NST_358_13C.2.fid
171.62
139.69 138.37 131.58 129.95 129.73 129.51 129.30 128.63 128.53 128.53 126.73 126.51 125.75 125.73 125.70 125.68 124.71 122.91 121.10
74.66
56.83 52.69
^{13}C NMR (CDCl$_3$, 151 MHz)
OH
O
O
S
CF$_3$
4z
f1 (ppm)

NST_358_19F_Varian400
NST_358_19F_Varian400 mr400-ATB F19-s2pul-cdcl3 May 15 2025

^{19}F NMR ($CDCl_3$, 376 MHz)

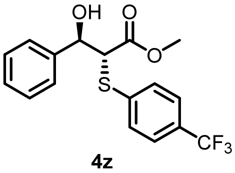

4z

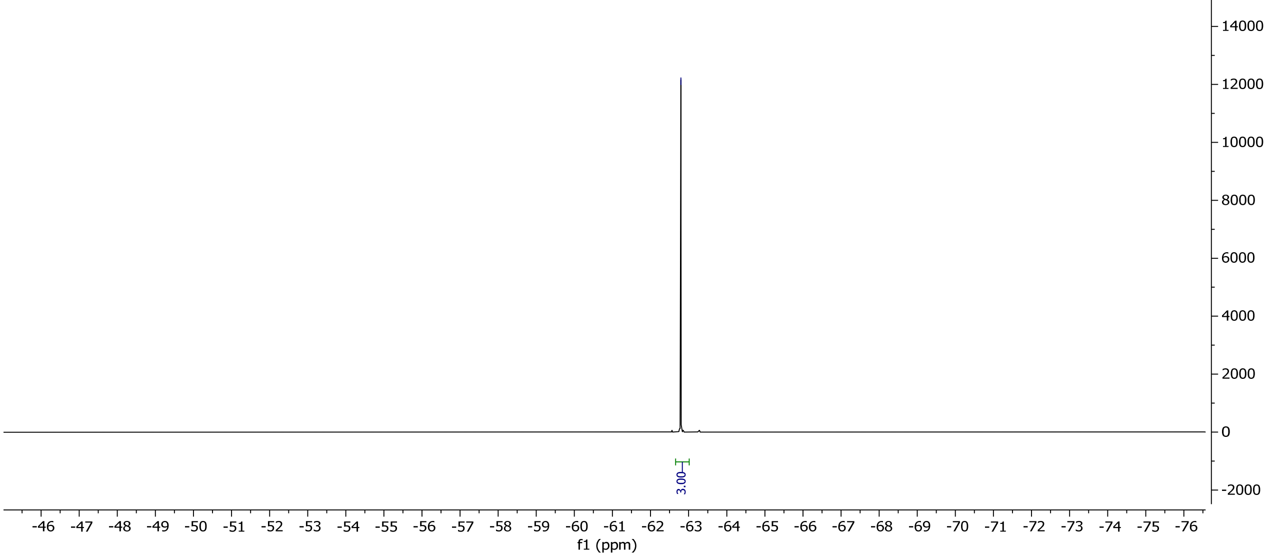

CSP HPLC traces:

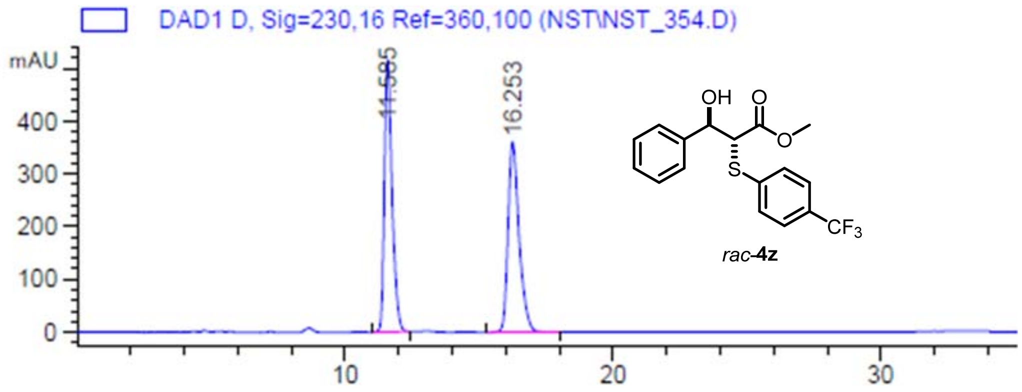

Signal 2: DAD1 D, Sig=230,16 Ref=360,100

Peak #	RetTime [min]	Type	Width [min]	Area [mAU*s]	Height [mAU]	Area %
1	11.585	BB	0.2974	1.02247e4	515.45801	49.7506
2	16.253	BB	0.4304	1.03272e4	360.26288	50.2494
Totals :				2.05519e4	875.72089	

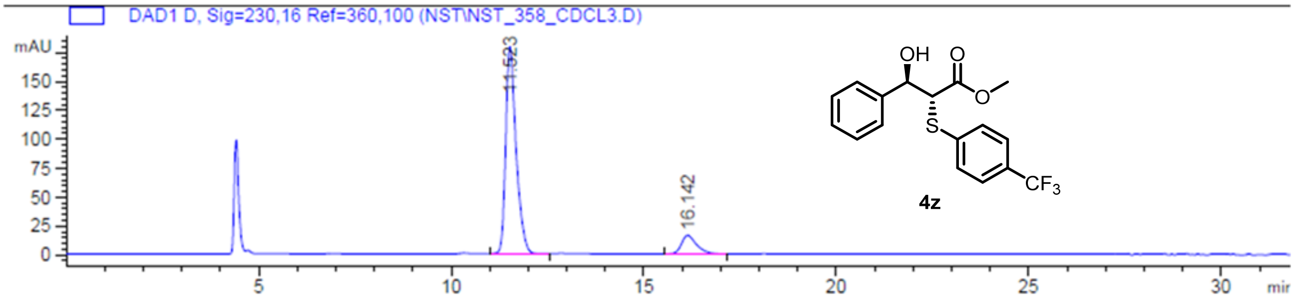

Signal 3: DAD1 D, Sig=230,16 Ref=360,100

Peak #	RetTime [min]	Type	Width [min]	Area [mAU*s]	Height [mAU]	Area %
1	11.523	BB	0.2784	3356.82104	179.30179	88.6534
2	16.142	BB	0.3987	429.63513	16.03016	11.3466
Totals :				3786.45618	195.33195	

4aa- Methyl (2*R*,3*R*)-2-((4-fluorophenyl)thio)-3-hydroxy-3-phenylpropanoate

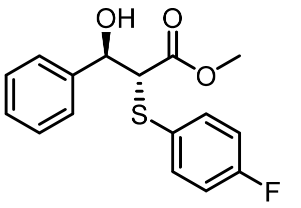

$C_{16}H_{15}FO_3S$

MW: 306.3 g/mol

Yield: 72%;

Colorless oil

The product (**4aa**) was prepared using the general procedure **B** from **1a** (methyl 2-(dimethyl(oxo)-λ^6-sulfaneylidene)acetate), **2a** (benzaldehyde), and **3d** (4-fluorobenzenethiol). The product was obtained as a colorless oil (22 mg; 72% yield). The diastereomeric ratio obtained for the *anti*-couple is greater than 20:1. The enantiomeric excess of the product was determined by CSP HPLC: AD-H column, *n*-hexane/iPrOH 80:20, 0.75 mL/min, t_{maj} = 11.4 min; t_{min} = 15.6 min, 91% ee.

^{1}H NMR (600 MHz, $CDCl_3$) δ 7.39 – 7.30 (m, 5H), 7.24 – 7.19 (m, 2H), 6.96 – 6.89 (m, 2H), 4.99 (d, *J* = 8.2 Hz, 1H), 3.83 (d, *J* = 8.2 Hz, 1H), 3.67 (s, 3H), 3.06 (br s, 1H).

^{13}C NMR (151 MHz, $CDCl_3$) δ 171.8, 163.0 (d, *J* = 249.0 Hz), 140.0, 136.1 (d, *J* = 8.3 Hz), 128.5, 127.8 (d, *J* = 3.3 Hz), 126.9, 116.1 (d, *J* = 21.9 Hz), 74.5, 58.2, 52.4.

^{19}F NMR (376 MHz, $CDCl_3$) δ -112.16 – -112.24 (m, 1F).

HRMS (MALDI$^+$) m/z calcd for $C_{16}H_{15}FO_3SNa^+$[M+Na]$^+$: 329.0618, found: 329.0623.

$[\alpha]_D^{RT}$ = +49.9 (c = 1.0 g/100 mL, $CHCl_3$).

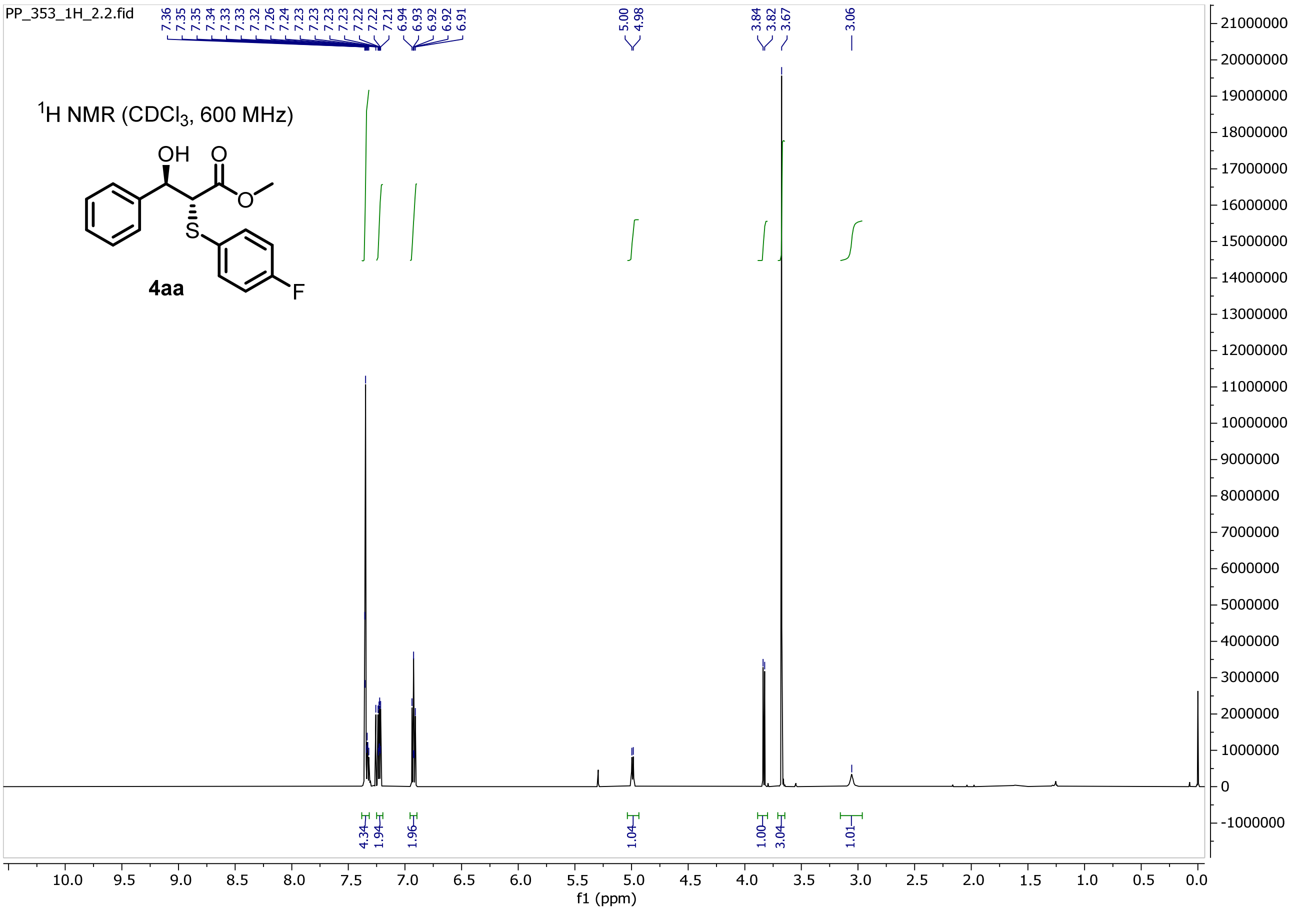
PP_353_1H_2.2.fid
1H NMR (CDCl3, 600 MHz)
OH
O
S
F
4aa
7.36 7.35 7.35 7.34 7.33 7.33 7.32 7.26 7.24 7.23 7.23 7.23 7.23 7.22 7.22 7.21 6.94 6.93 6.92 6.92 6.91
5.00 4.98
3.84 3.82 3.67
3.06
4.34 1.94 1.96 1.04 1.00 3.04 1.01
f1 (ppm)

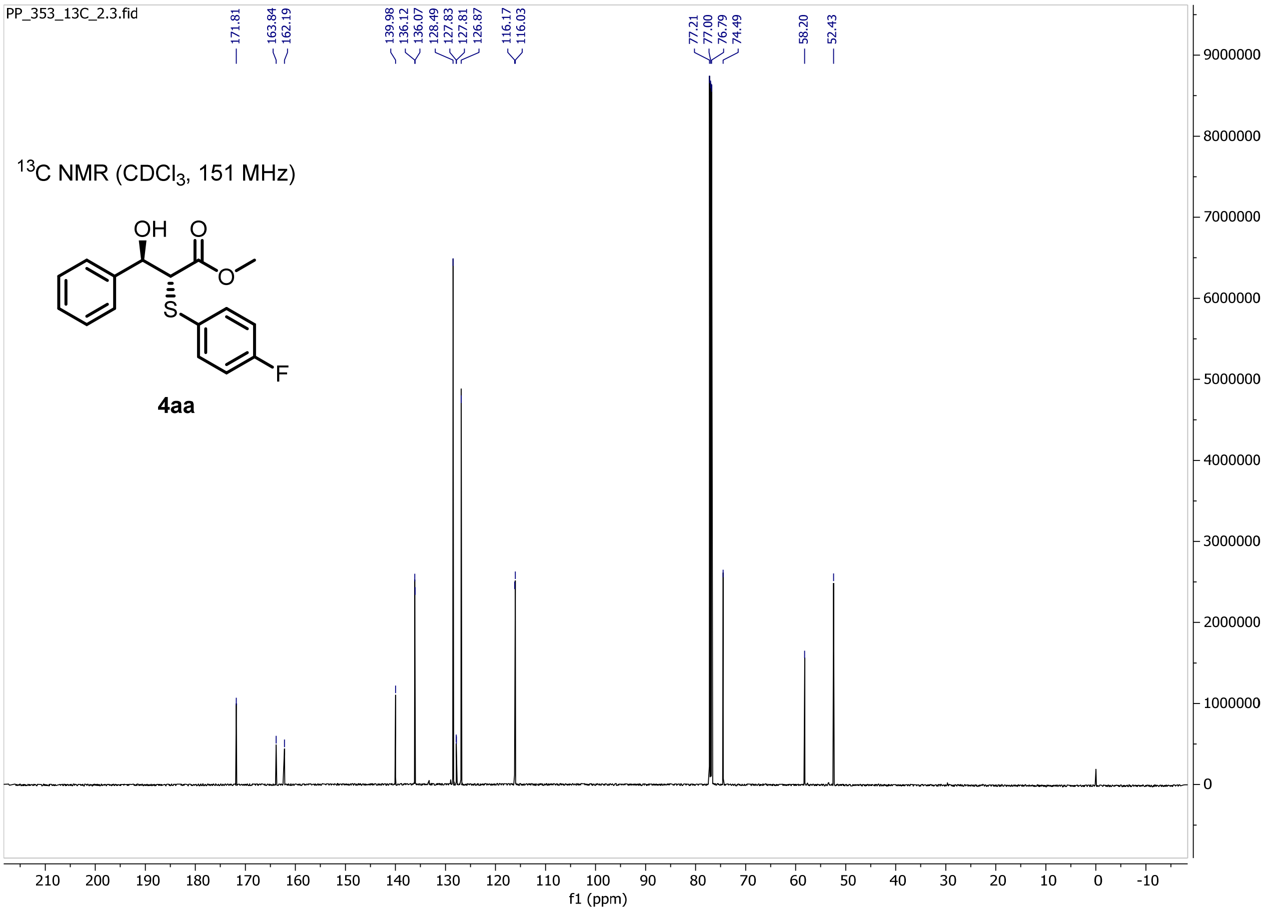
PP_353_13C_2.3.fid
13C NMR (CDCl3, 151 MHz)
OH
O
S
F
4aa
171.81
163.84 162.19
139.98 136.12 136.07 128.49 127.83 127.81 126.87 116.17 116.03
77.21 77.00 76.79 74.49
58.20
52.43
f1 (ppm)

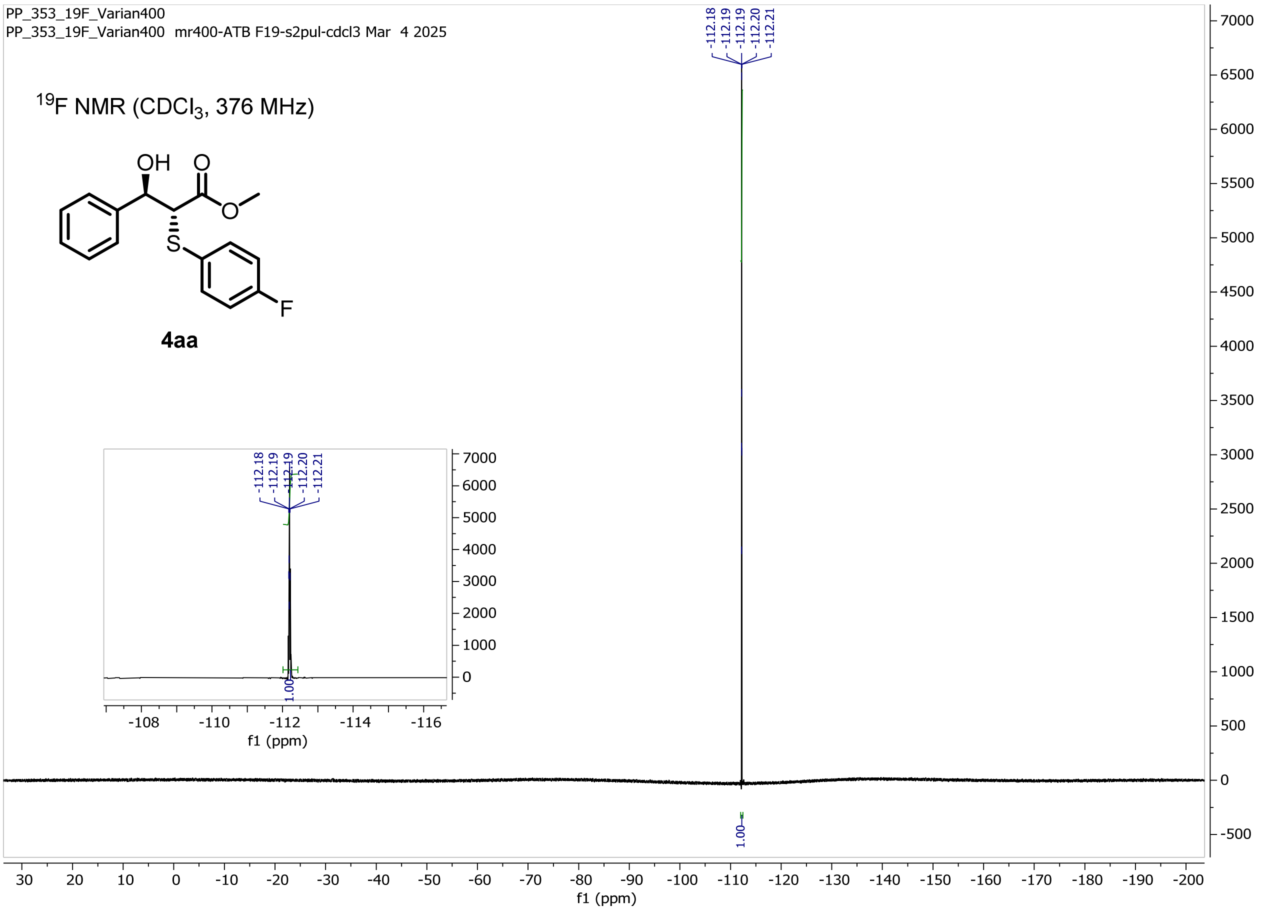

PP_353_19F_Varian400
PP_353_19F_Varian400 mr400-ATB F19-s2pul-cdcl3 Mar 4 2025
19F NMR (CDCl3, 376 MHz)
OH
O
O
S
F
4aa
-112.18
-112.19
-112.19
-112.20
-112.21
1.00
f1 (ppm)

CSP HPLC traces:

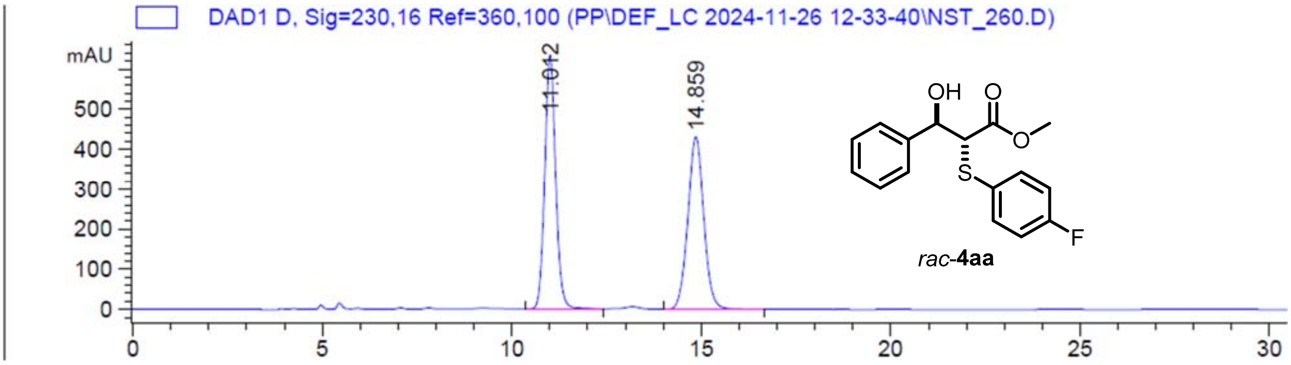

Signal 3: DAD1 D, Sig=230,16 Ref=360,100

Peak #	RetTime [min]	Type	Width [min]	Area [mAU*s]	Height [mAU]	Area %
1	11.012	BB	0.3052	1.26733e4	633.90033	50.2353
2	14.859	BB	0.4440	1.25546e4	430.84000	49.7647
Totals :				2.52279e4	1064.74033	

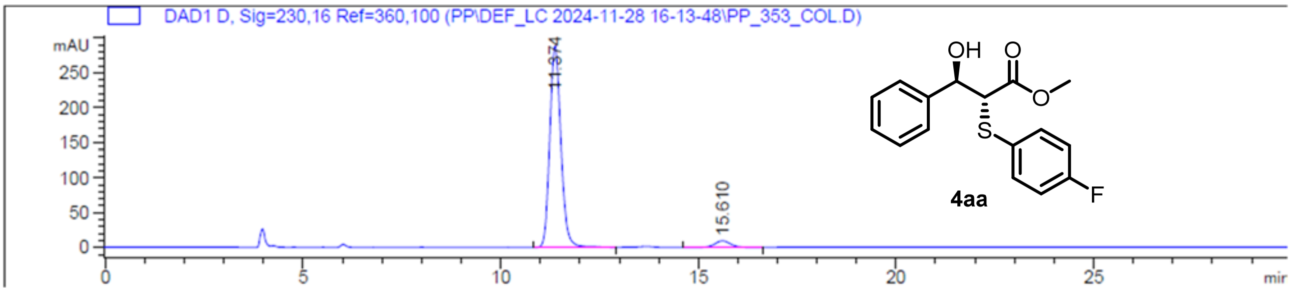

Signal 3: DAD1 D, Sig=230,16 Ref=360,100

Peak #	RetTime [min]	Type	Width [min]	Area [mAU*s]	Height [mAU]	Area %
1	11.374	BB	0.3016	5634.46973	288.81094	95.6118
2	15.610	BB	0.4236	258.60156	9.32378	4.3882
Totals :				5893.07129	298.13473	

4ab- Methyl (2*R*,3*R*)-2-((4-bromophenyl)thio)-3-hydroxy-3-phenylpropanoate

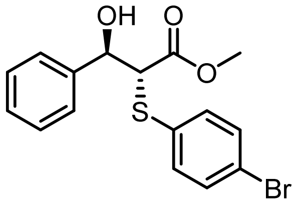

$C_{16}H_{15}BrO_3S$

MW: 367.2 g/mol

Yield: 66%;

White solid

The product (**4ab**) was prepared using the general procedure **C** from **1a** (methyl 2-(dimethyl(oxo)-λ^6-sulfaneylidene)acetate), **2a** (benzaldehyde), and **3e** (4-bromobenzenethiol). The diastereomeric ratio obtained for the *anti*-couple is greater than 20:1. The product was obtained as a colorless oil (23.9 mg; 66% yield). The enantiomeric excess of the product was determined by CSP HPLC: AD-H column, *n*-hexane/iPrOH 80:20, 0.75 mL/min, t_{maj} = 13.6 min; t_{min} = 20.2 min, 86% ee.

^{1}H NMR (600 MHz, $CDCl_3$) δ 7.38 – 7.30 (m, 7H), 7.12 – 7.06 (m, 2H), 5.01 (dd, *J* = 8.2, 5.3 Hz, 1H), 3.88 (d, *J* = 8.2 Hz, 1H), 3.69 (s, 3H), 3.04 (d, *J* = 5.4 Hz, 1H).

^{13}C NMR (151 MHz, $CDCl_3$) δ 171.7, 139.8, 134.7, 132.1, 132.0, 128.6, 128.5, 126.8, 122.7, 74.5, 57.6, 52.6.

HRMS (MALDI$^+$) m/z calcd for $C_{16}H_{15}BrO_3SNa^+[M+Na]^+$: 388.9817, found: 388.9820.

$[\alpha]_D^{RT}$ = +33.9 (c = 1.0 g/100 mL, $CHCl_3$).

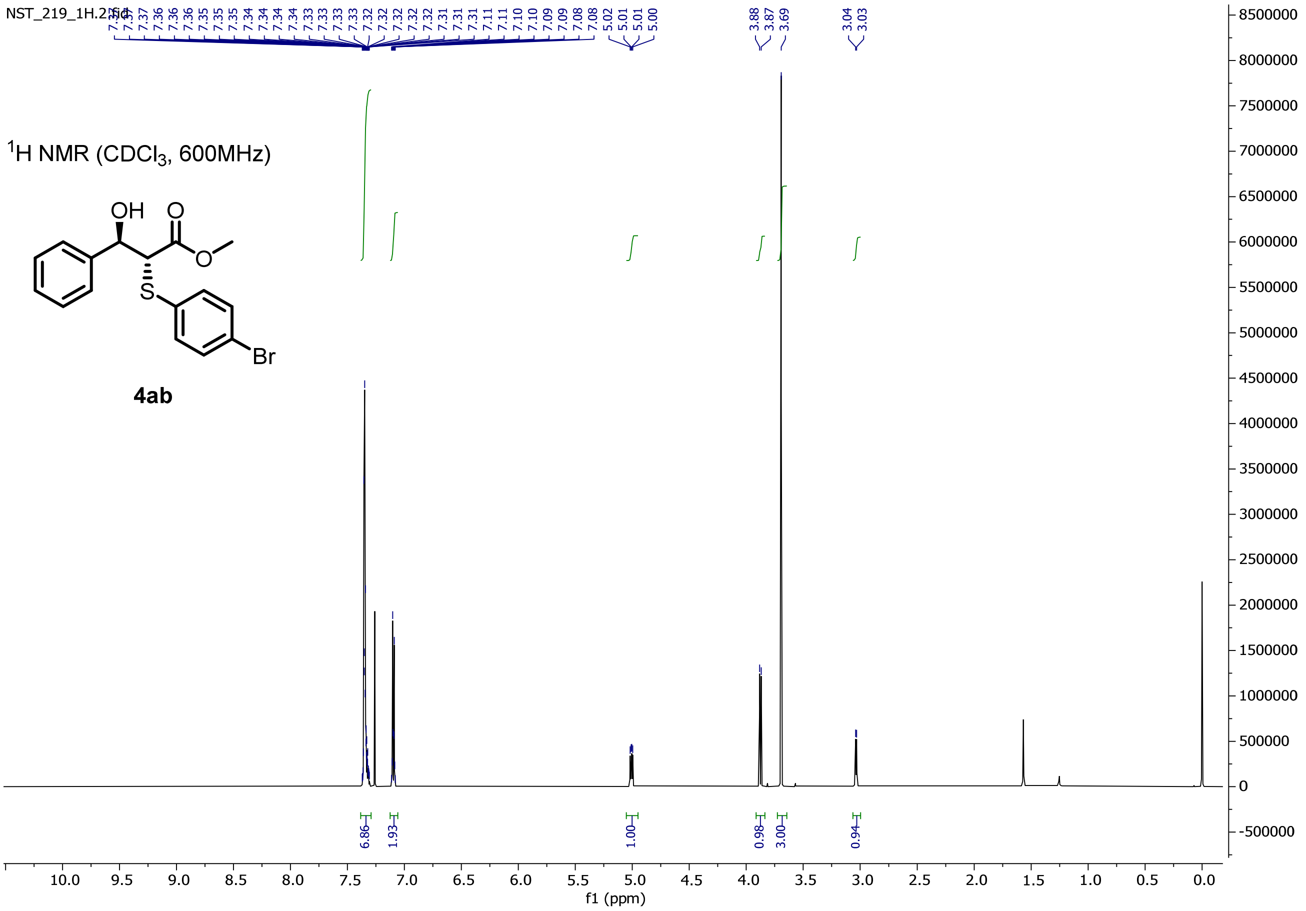
NST_219_1H.2.fid
7.37
7.36
7.36
7.35
7.35
7.34
7.34
7.34
7.34
7.33
7.33
7.33
7.32
7.32
7.32
7.32
7.31
7.31
7.31
7.11
7.10
7.09
7.09
7.08
7.08
5.02
5.01
5.01
5.00
3.88
3.87
3.69
3.04
3.03
^{1}H NMR ($CDCl_3$, 600MHz)
OH
O
O
S
Br
4ab
6.86
1.93
1.00
0.98
3.00
0.94
f1 (ppm)

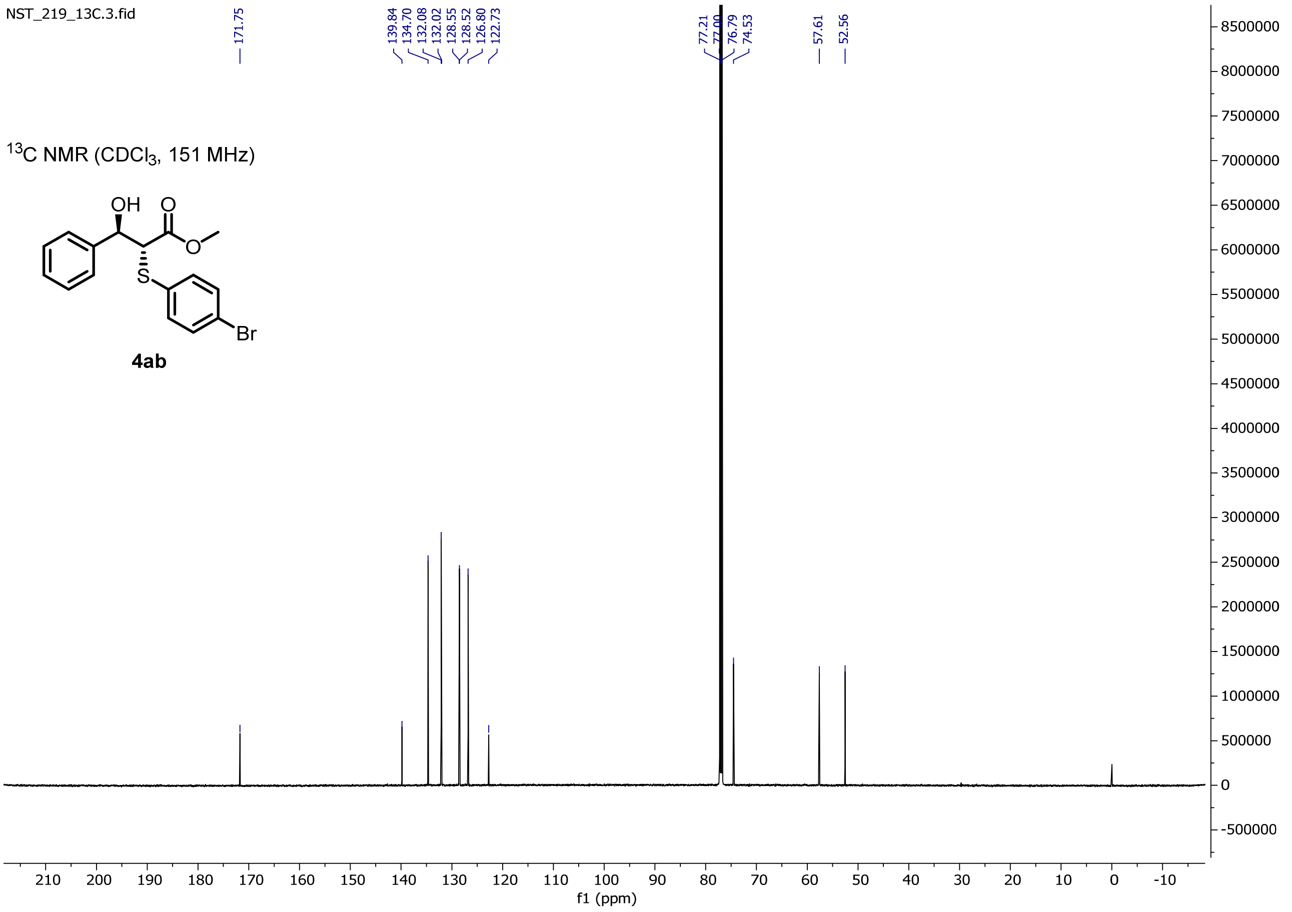
NST_219_13C.3.fid
171.75
139.84
134.70
132.08
132.02
128.55
128.52
126.80
122.73
77.21
77.00
76.79
74.53
57.61
52.56
^{13}C NMR ($CDCl_3$, 151 MHz)
OH
O
O
S
Br
4ab
f1 (ppm)

CSP HPLC traces:

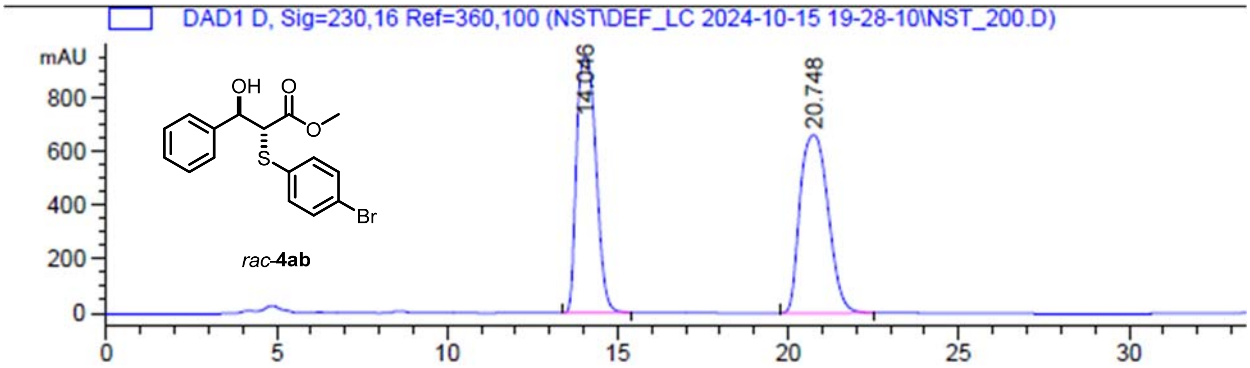

Signal 3: DAD1 D, Sig=230,16 Ref=360,100

Peak #	RetTime [min]	Type	Width [min]	Area [mAU*s]	Height [mAU]	Area %
1	14.046	BB	0.6456	3.75925e4	950.11163	49.8915
2	20.748	BB	0.9350	3.77560e4	660.38678	50.1085

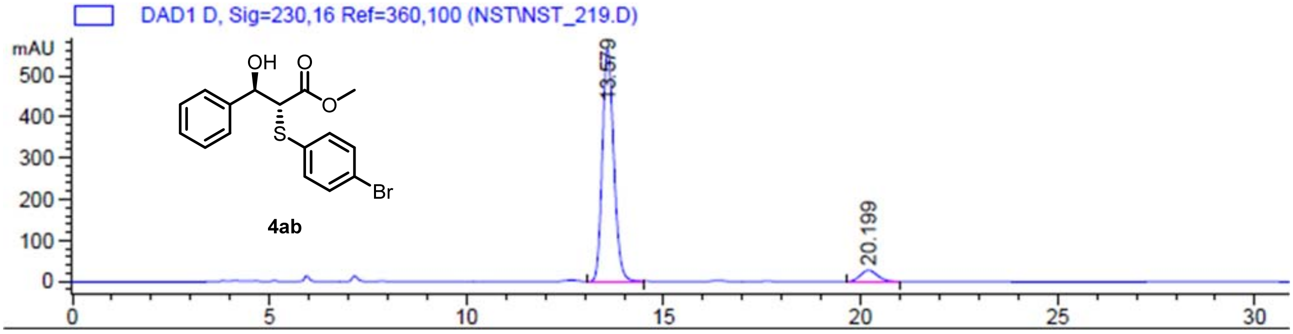

Signal 3: DAD1 D, Sig=230,16 Ref=360,100

Peak #	RetTime [min]	Type	Width [min]	Area [mAU*s]	Height [mAU]	Area %
1	13.579	VB	0.3115	1.13800e4	563.82849	93.1646
2	20.199	BB	0.4677	834.94885	27.54262	6.8354
Totals :				1.22150e4	591.37111	

4ac- Methyl (2*R*,3*R*)-3-hydroxy-3-phenyl-2-(phenylthio)propanoate

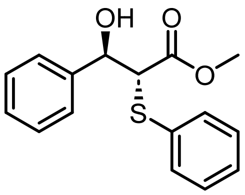

$C_{16}H_{16}O_3S$

MW: 288.4 g/mol

Yield: 82%;

Colorless oil

The product (**4ac**) was prepared using the general procedure **B** from **1a** (methyl 2-(dimethyl(oxo)-λ^6-sulfaneylidene)acetate), **2a** (benzaldehyde), and **3f** (benzenethiol). The product was obtained as a colorless oil (20 mg; 82% yield). The diastereomeric ratio obtained for the *anti*-couple is greater than 20:1. The enantiomeric excess of the product was determined by CSP HPLC: AD-H column, n-hexane/iPrOH 80:20, 0.75 mL/min, t_{maj} = 11.4 min; t_{min} = 13.8 min, 85% ee.

^{1}H NMR (600 MHz, $CDCl_3$) δ 7.41 – 7.19 (m, 10H), 5.02 (dd, *J* = 8.0, 3.7 Hz, 1H), 3.93 (d, *J* = 7.8 Hz, 1H), 3.66 (s, 3H), 3.13 (d, *J* = 4.8 Hz, 1H).

^{13}C NMR (151 MHz, $CDCl_3$) δ 171.9, 140.1, 133.3, 132.8, 129.0, 128.5, 128.4, 128.3, 126.8, 74.5, 57.7, 52.4.

HRMS (MALDI$^-$) m/z calcd for $C_{16}H_{15}O_3S^-$[M-H$^+$]: 287.0747, found: 287.0744.

$[\alpha]_D^{RT}$ = +64.5 (c = 1.0 g/100 mL, $CHCl_3$).

The relative configuration of product **4ac** was assigned as *anti* by comparing its NMR data with literature values[25].

The absolute configuration of the major enantiomer of product **4ac** was assigned as 2R,3R by comparing its specific optical rotation with the reported value for the 2R,3R enantiomer: $[\alpha]_D^{25^\circ C}$ = +68.8 (c = 0.85 g/100 mL, $CHCl_3$)[26].

[25] S.-K. Kang, D.-C. Park, H.-S. Rho, S.-H. Yoon, J.-S. Shin, *J. Chem. Soc., Perkin Trans. 1* **1994**, 3513-3514.
[26] E. J. Corey, S. Choi, *Tetrahedron Lett.* **2000**, *41*, 2769–2772.

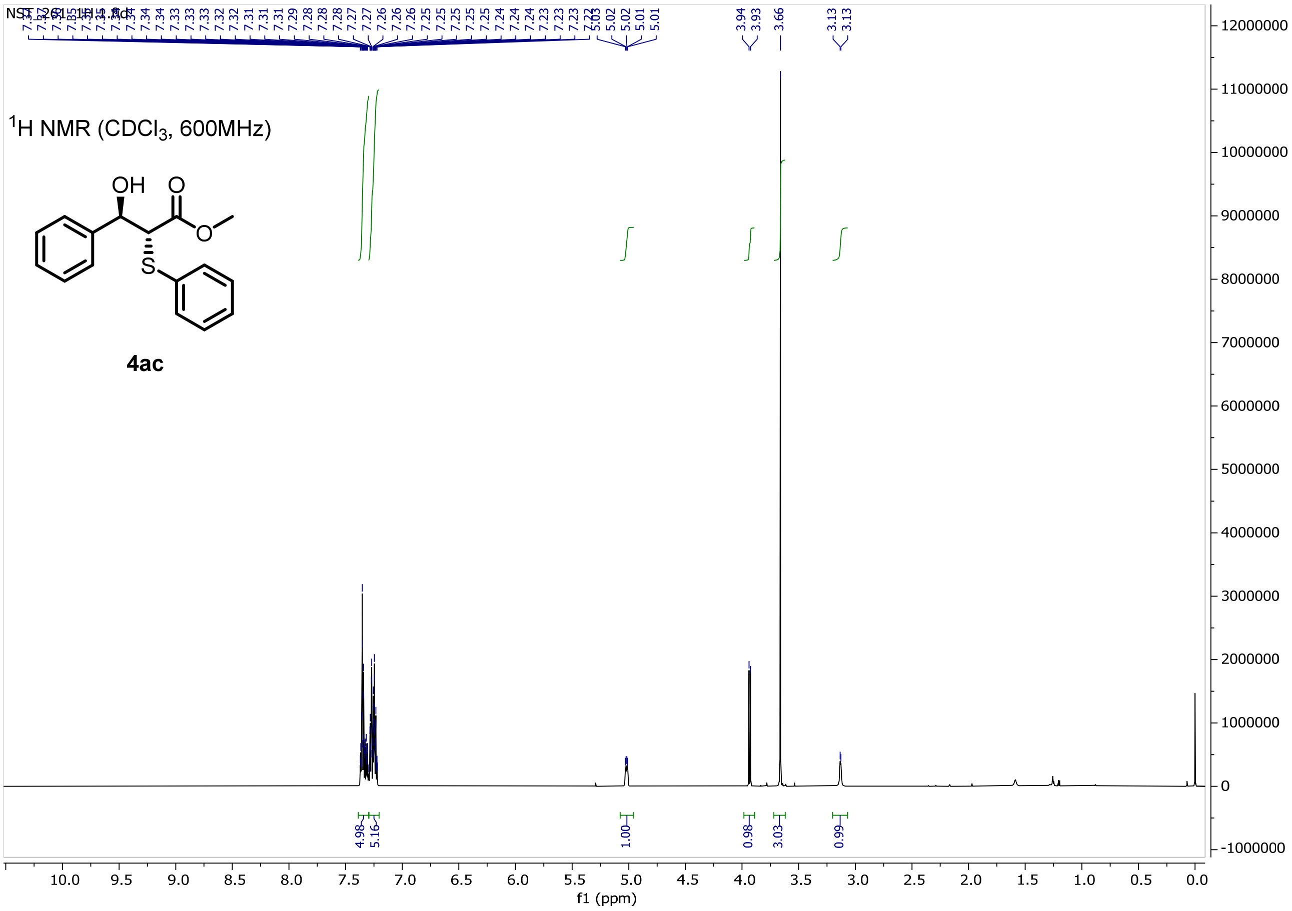

NST_261_1H.1.fid
1H NMR (CDCl3, 600MHz)
OH
O
O
S
4ac
3.94
3.93
3.66
3.13
3.13
5.03
5.02
5.01
5.01
4.98
5.16
1.00
0.98
3.03
0.99
f1 (ppm)

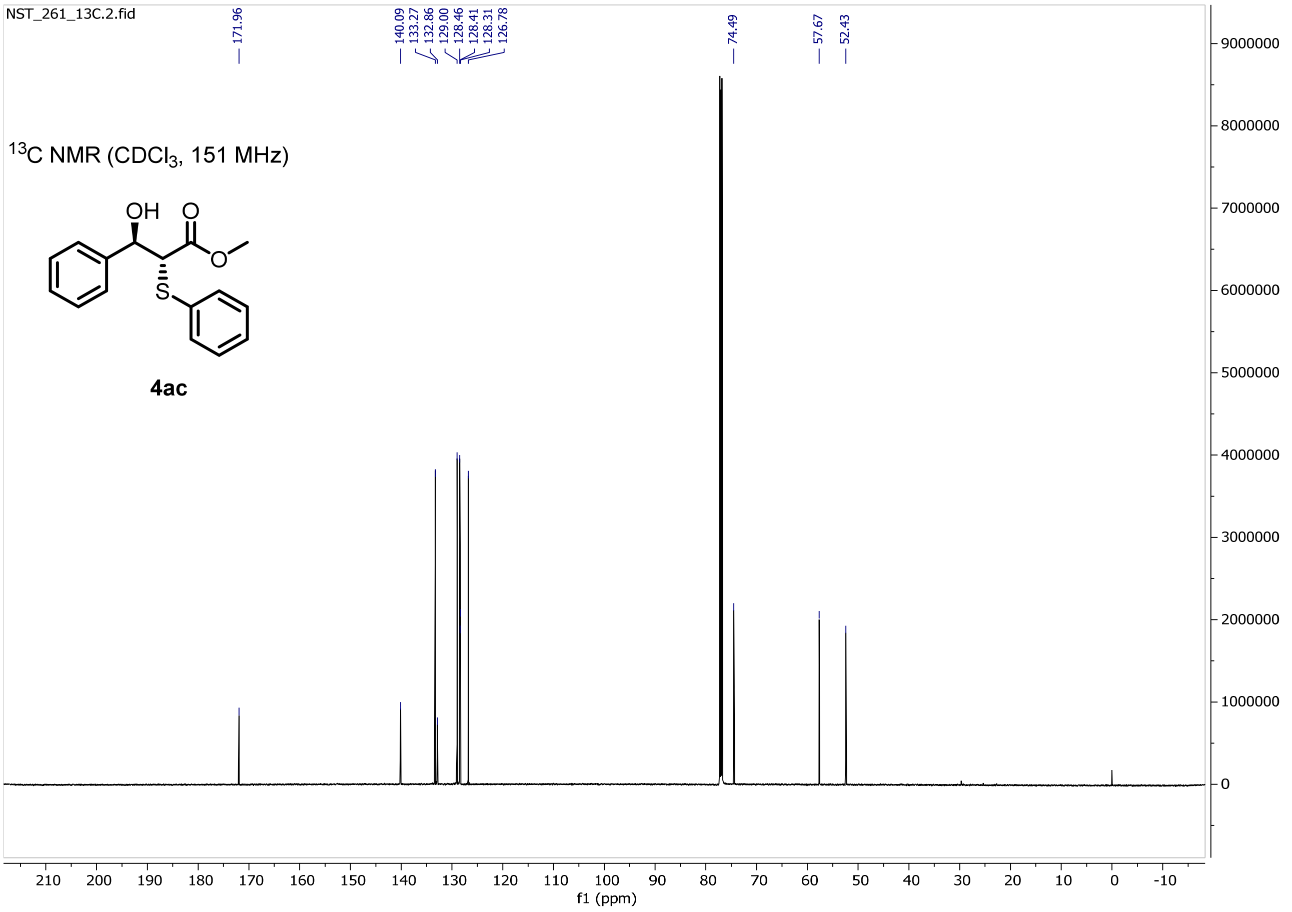

NST_261_13C.2.fid
171.96
140.09
133.27
132.86
129.00
128.46
128.41
128.31
126.78
74.49
57.67
52.43
13C NMR (CDCl3, 151 MHz)
OH
O
O
S
4ac
f1 (ppm)

CSP HPLC traces:

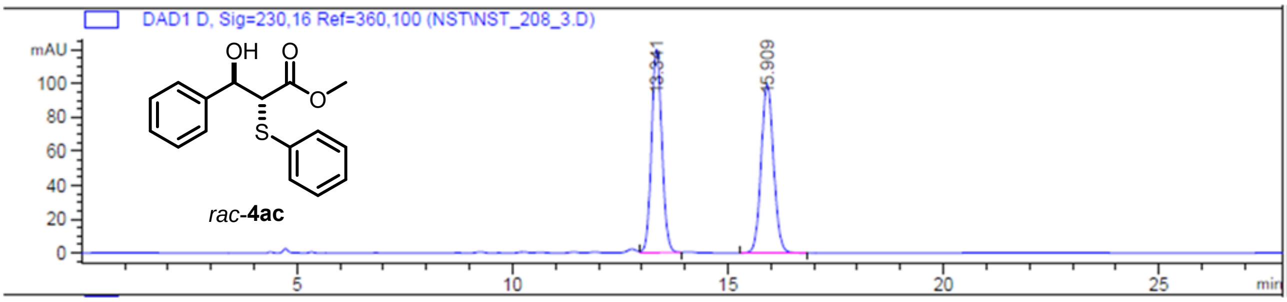

Signal 2: DAD1 D, Sig=230,16 Ref=360,100

Peak #	RetTime [min]	Type	Width [min]	Area [mAU*s]	Height [mAU]	Area %
1	13.341	VB	0.2591	2006.85449	119.98997	50.0931
2	15.909	BB	0.3108	1999.39832	99.33409	49.9069
Totals :				4006.25281	219.32407	

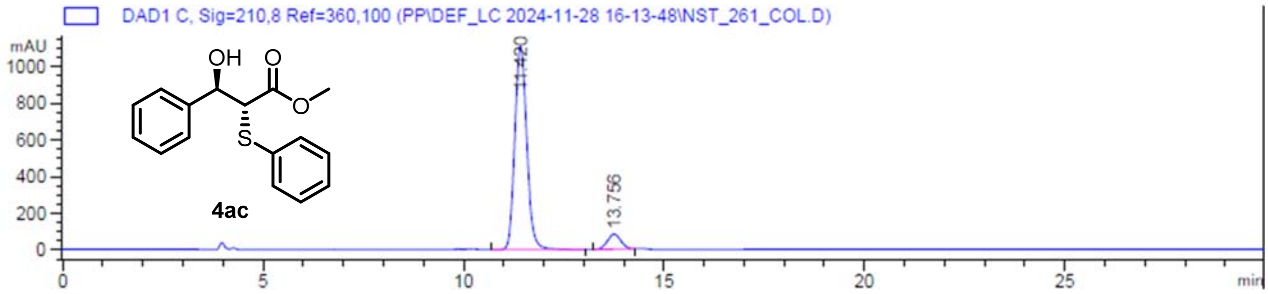

Signal 2: DAD1 C, Sig=210,8 Ref=360,100

Peak #	RetTime [min]	Type	Width [min]	Area [mAU*s]	Height [mAU]	Area %
1	11.420	BB	0.3269	2.31661e4	1112.50793	92.4833
2	13.756	BB	0.3618	1882.84131	81.40509	7.5167
Totals :				2.50489e4	1193.91302	

4ad- Methyl (2*R*,3*R*)-3-hydroxy-3-phenyl-2-(p-tolylthio)propanoate

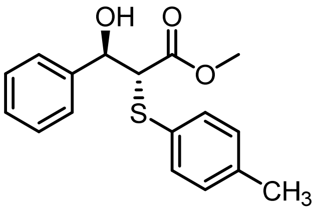

$C_{17}H_{18}O_3S$

MW: 302.4 g/mol

Yield: 73%;

Colorless oil

The product (**4ad**) was prepared using the general procedure **B** from **1a** (methyl 2-(dimethyl(oxo)-λ^6-sulfaneylidene)acetate), **2a** (benzaldehyde), and **3g** (4-methylbenzenethiol). The product was obtained as a colorless oil (22 mg; 73% yield). The diastereomeric ratio obtained for the *anti*-couple is greater than 20:1. The enantiomeric excess of the product was determined by CSP HPLC: AD-H column, *n*-hexane/iPrOH 80:20, 0.75 mL/min, t_{maj} = 11.0 min; t_{min} = 13.9 min, 73% ee.

^{1}H NMR (600 MHz, $CDCl_3$) δ 7.38 – 7.30 (m, 5H), 7.19 – 7.16 (m, 2H), 7.08 – 7.04 (m, 2H), 5.00 (dd, *J* = 8.1, 5.4 Hz, 1H), 3.87 (d, *J* = 8.1 Hz, 1H), 3.66 (s, 3H), 3.14 (d, *J* = 5.4 Hz, 1H), 2.32 (s, 3H).

^{13}C NMR (151 MHz, $CDCl_3$) δ 172.0, 140.1, 138.7, 133.8, 129.8, 129.0, 128.4, 128.3, 126.8, 74.3, 57.9, 52.4, 21.1.

HRMS (MALDI$^+$) m/z calcd for $C_{17}H_{18}O_3SNa^+$ $[M+Na]^+$: 325.0869, found: 325.0874.

$[\alpha]_D^{RT}$ = +45.2 (c = 1.0 g/100 mL, $CHCl_3$).

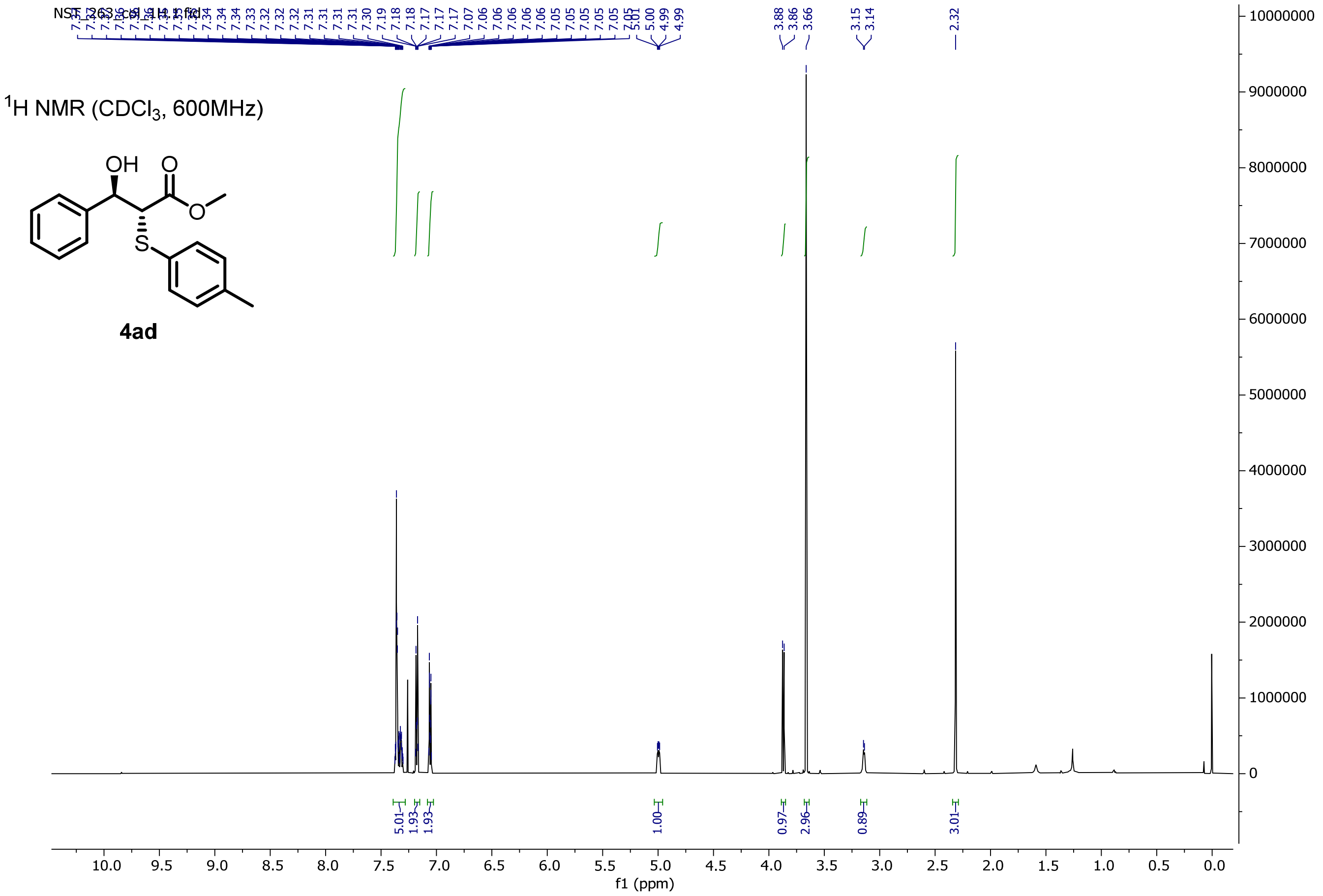
NST_263_col_1H.1.fid
7.34 7.34 7.34 7.33 7.32 7.32 7.32 7.31 7.31 7.31 7.31 7.30 7.19 7.18 7.18 7.17 7.17 7.17 7.07 7.06 7.06 7.06 7.06 7.06 7.05 7.05 7.05 7.05 7.05 7.05 5.01 5.00 4.99 4.99
3.88 3.86 3.66
3.15 3.14
2.32
1H NMR (CDCl3, 600MHz)
OH
O
O
S
4ad
5.01 1.93 1.93 1.00 0.97 2.96 0.89 3.01
10.0 9.5 9.0 8.5 8.0 7.5 7.0 6.5 6.0 5.5 5.0 4.5 4.0 3.5 3.0 2.5 2.0 1.5 1.0 0.5 0.0
f1 (ppm)
10000000 9000000 8000000 7000000 6000000 5000000 4000000 3000000 2000000 1000000 0

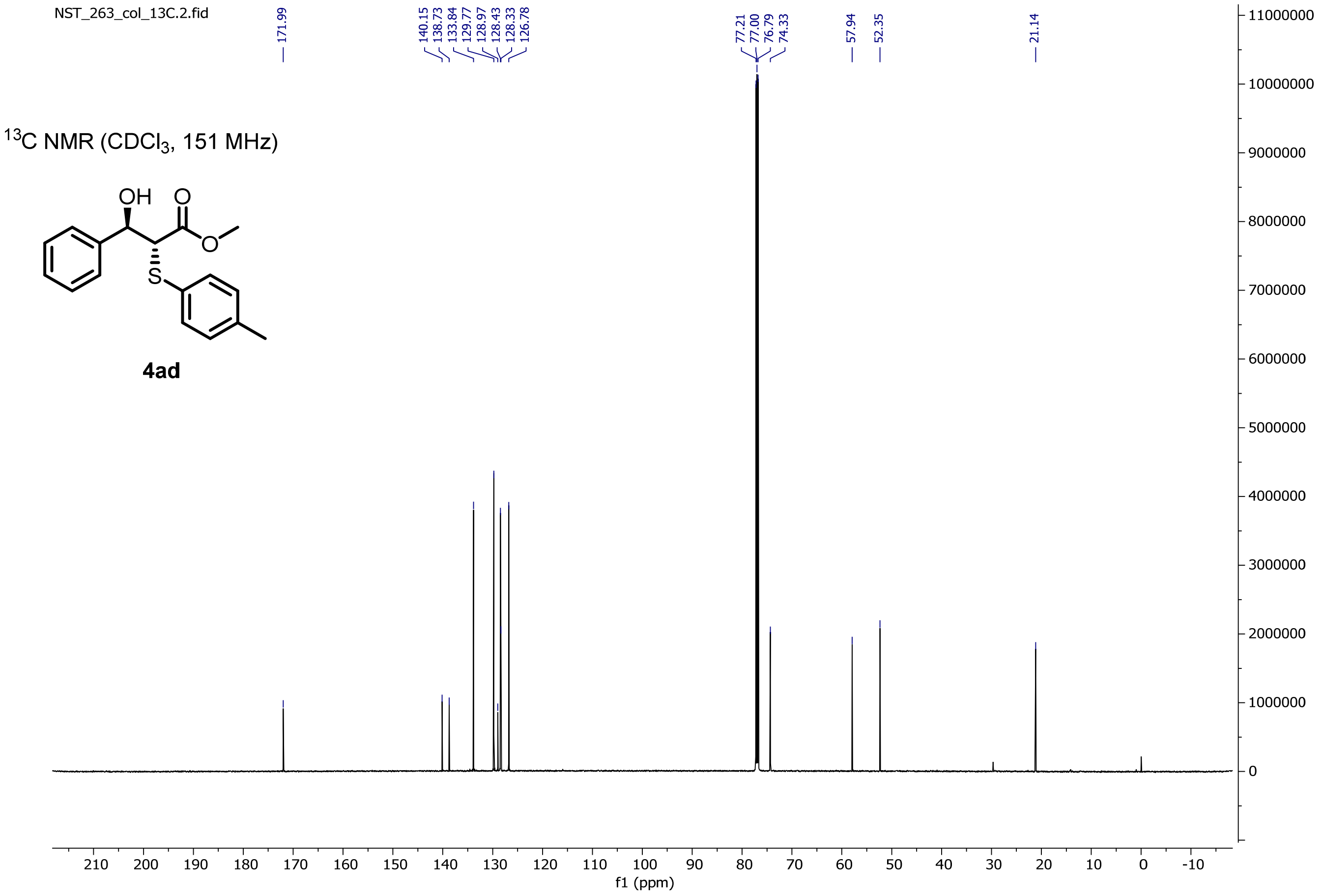
NST_263_col_13C.2.fid
171.99
140.15 138.73 133.84 129.77 128.97 128.43 128.33 126.78
77.21 77.00 76.79 74.33
57.94 52.35
21.14
13C NMR (CDCl3, 151 MHz)
OH
O
O
S
4ad
210 200 190 180 170 160 150 140 130 120 110 100 90 80 70 60 50 40 30 20 10 0 -10
f1 (ppm)
11000000 10000000 9000000 8000000 7000000 6000000 5000000 4000000 3000000 2000000 1000000 0

CSP HPLC traces:

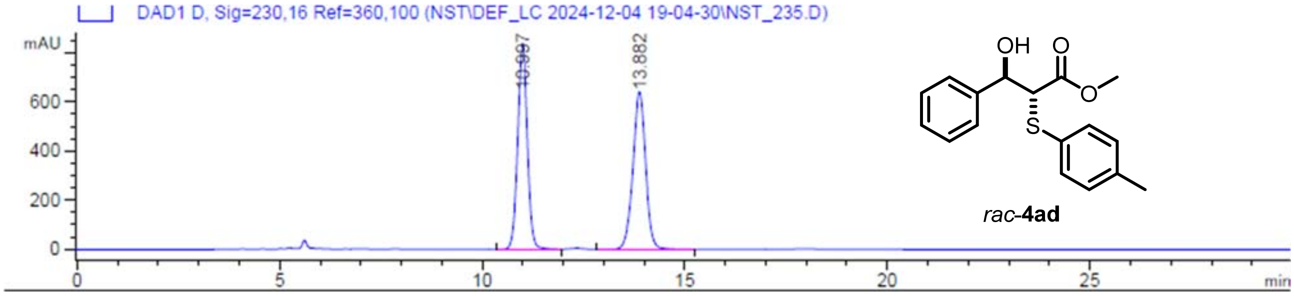

Signal 3: DAD1 D, Sig=230,16 Ref=360,100

Peak #	RetTime [min]	Type	Width [min]	Area [mAU*s]	Height [mAU]	Area %
1	10.997	BB	0.2537	1.37545e4	837.26263	49.7317
2	13.882	BB	0.3311	1.39029e4	640.83020	50.2683

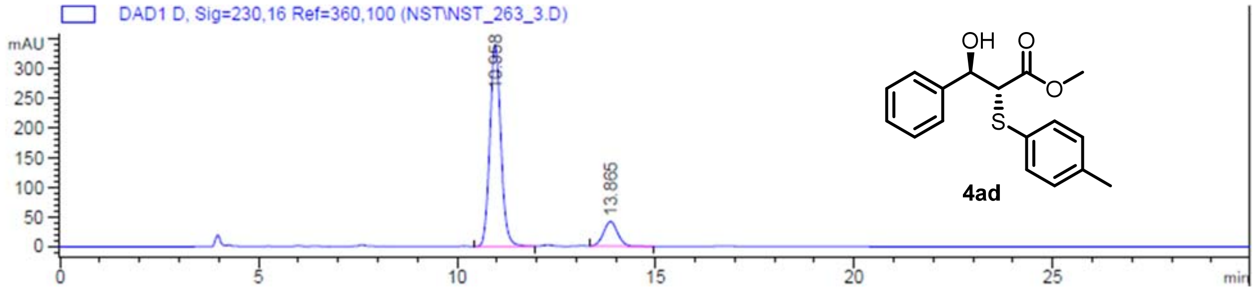

Signal 3: DAD1 D, Sig=230,16 Ref=360,100

Peak #	RetTime [min]	Type	Width [min]	Area [mAU*s]	Height [mAU]	Area %
1	10.958	BB	0.2960	6539.27490	340.62939	86.3695
2	13.865	BB	0.3832	1032.00061	41.63879	13.6305
Totals :				7571.27551	382.26818	

4ae- Methyl (2*R*,3*R*)-3-hydroxy-2-((4-methoxyphenyl)thio)-3-phenylpropanoate

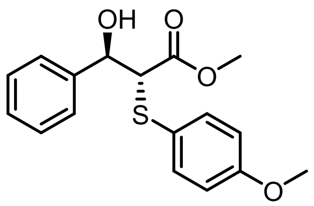

$C_{17}H_{18}O_4S$

MW: 318.4 g/mol

Yield: 90%

Colorless oil

The product (**4ae**) was prepared using the general procedure **C** from **1a** (methyl 2-(dimethyl(oxo)-λ^6-sulfaneylidene)acetate), **2a** (benzaldehyde), and **3h** (4-methoxybenzenethiol). The product was obtained as a colorless oil (28.7 mg; 90% yield). The diastereomeric ratio obtained for the *anti*-couple is greater than 20:1. The enantiomeric excess of the product was determined by CSP HPLC: AD-H column, *n*-hexane/iPrOH 80:20, 0.75 mL/min, t_{maj} = 18.2 min; t_{min} = 21.2 min, 52% ee.

^{1}H NMR (600 MHz, CDCl3) δ 7.39 – 7.34 (m, 4H), 7.34 – 7.30 (m, 1H), 7.24 – 7.19 (m, 2H), 6.80 – 6.75 (m, 2H), 4.97 (d, *J* = 8.0 Hz, 1H), 3.80 (d, *J* = 8.0 Hz, 1H), 3.78 (s, 3H), 3.66 (s, 3H).

^{13}C NMR (151 MHz, $CDCl_3$) δ 172.0, 160.2, 140.2, 136.3, 128.4, 128.3, 126.8, 122.8, 114.5, 74.2, 58.3, 55.3, 52.3.

HRMS (MALDI$^+$) m/z calcd for $C_{17}H_{18}O_4SNa^+$ [M+Na]$^+$: 341.0818, found 341.0821.

$[\alpha]_D^{RT}$ = +30.6 (c = 1.0 g/100 mL, $CHCl_3$).

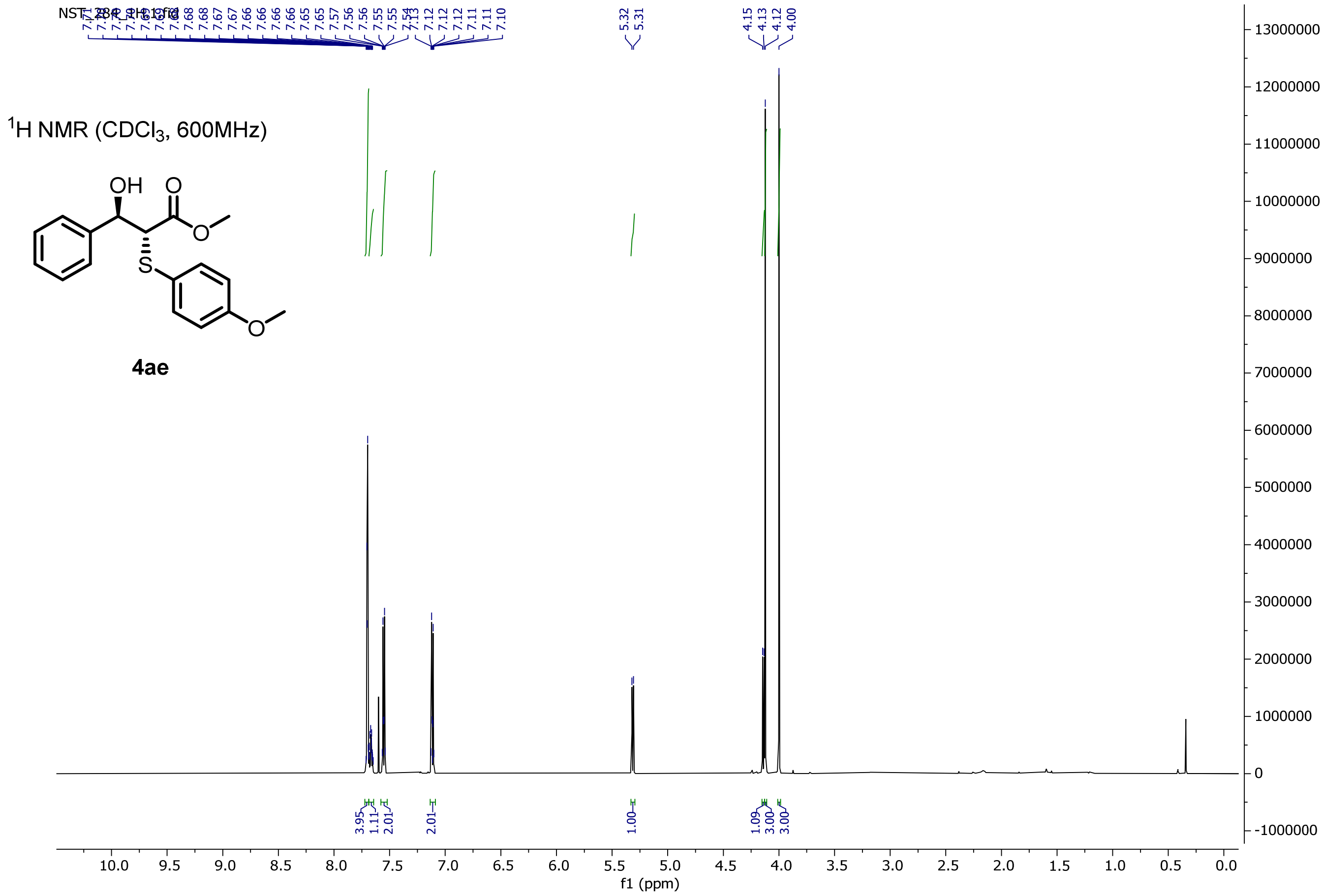
NST_284_1H.1.fid
1H NMR (CDCl3, 600MHz)
OH
O
O
S
O
4ae
7.68
7.68
7.67
7.67
7.66
7.66
7.66
7.66
7.65
7.65
7.57
7.56
7.55
7.55
7.54
7.13
7.12
7.12
7.12
7.11
7.11
7.10
5.32
5.31
4.15
4.13
4.12
4.00
3.95
1.11
2.01
2.01
1.00
1.09
3.00
3.00
f1 (ppm)

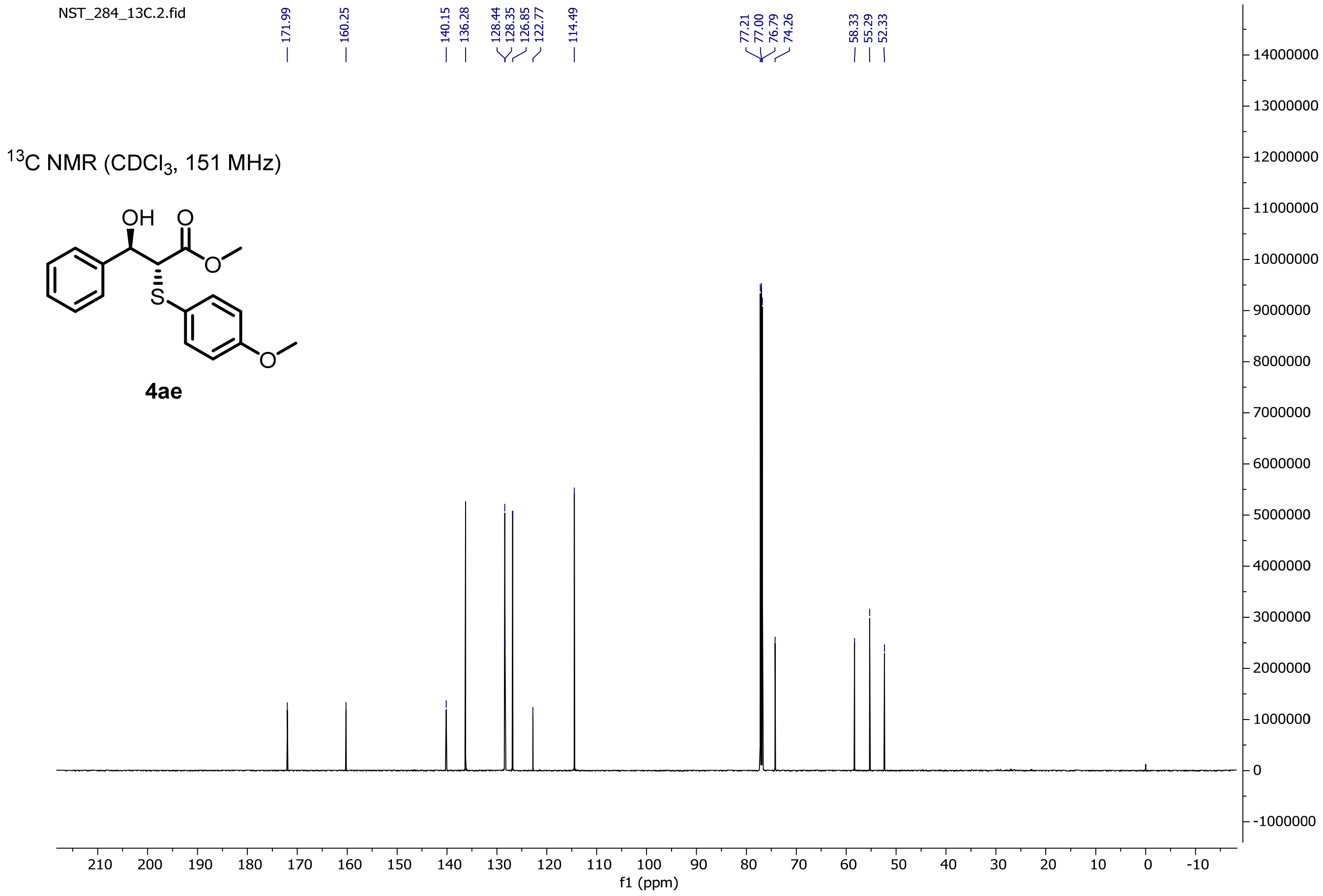
NST_284_13C.2.fid
13C NMR (CDCl3, 151 MHz)
OH
O
O
S
O
4ae
171.99
160.25
140.15
136.28
128.44
128.35
126.85
122.77
114.49
77.21
77.00
76.79
74.26
58.33
55.29
52.33
f1 (ppm)

CSP HPLC traces:

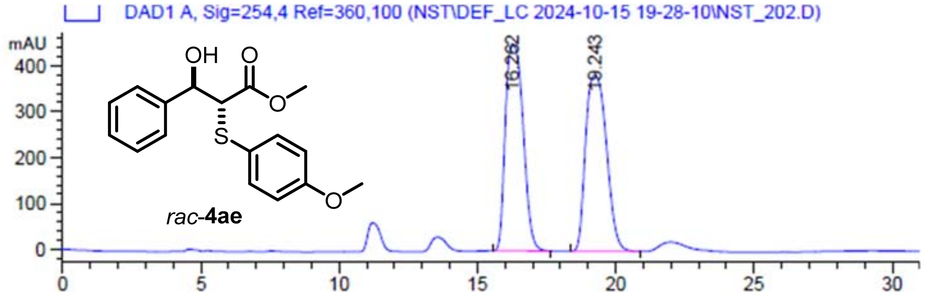

Signal 1: DAD1 A, Sig=254,4 Ref=360,100

Peak #	RetTime [min]	Type	Width [min]	Area [mAU*s]	Height [mAU]	Area %
1	16.262	BB	0.7310	2.01325e4	450.86514	49.9210
2	19.243	BB	0.8626	2.01962e4	383.35471	50.0790

Totals : 4.03287e4 834.21985

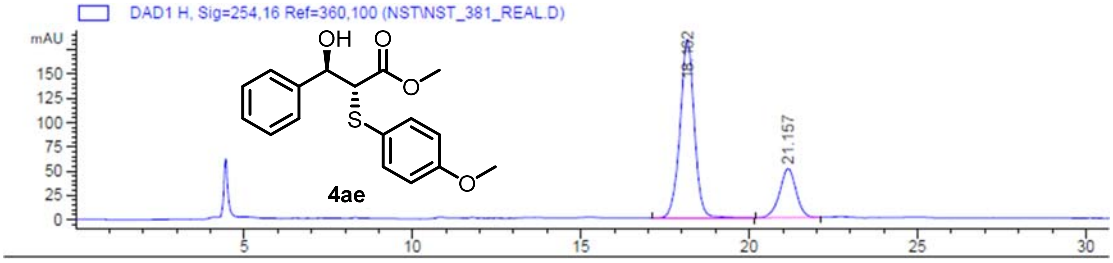

Signal 3: DAD1 H, Sig=254,16 Ref=360,100

Peak #	RetTime [min]	Type	Width [min]	Area [mAU*s]	Height [mAU]	Area %
1	18.162	BB	0.4446	5399.92822	183.87741	75.9671
2	21.157	BB	0.5146	1708.31995	50.50333	24.0329

Totals : 7108.24817 234.38074

4af- Methyl (2*R*,3*R*)-3-hydroxy-2-(naphthalen-2-ylthio)-3-phenylpropanoate

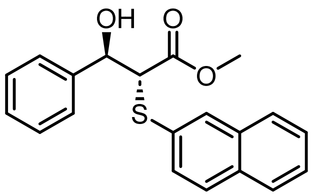

$C_{20}H_{18}O_3S$

MW: 338.4 g/mol

Yield: 78%;

White solid

The product (**4af**) was prepared using the general procedure **B** from **1a** (methyl 2-(dimethyl(oxo)-λ^6-sulfaneylidene)acetate), **2a** (benzaldehyde), and **3i** (naphthalene-2-thiol). The product was obtained as a white solid (26.4 mg; 78% yield). The diastereomeric ratio obtained for the *anti*-couple is greater than 20:1. The enantiomeric excess of the product was determined by CSP HPLC: AD-H column, *n*-hexane/iPrOH 80:20, 0.75 mL/min, t_{maj} = 16.7 min; t_{min} = 21.0 min, 60% ee.

^{1}H NMR (600 MHz, $CDCl_3$) δ 7.81 – 7.76 (m, 1H), 7.74 – 7.67 (m, 3H), 7.51 – 7.45 (m, 2H), 7.41 – 7.36 (m, 2H), 7.36 – 7.29 (m, 4H), 5.08 (d, *J* = 7.9 Hz, 1H), 4.02 (d, *J* = 7.9 Hz, 1H), 3.67 (s, 3H), 2.62 (br s, 1H).

^{13}C NMR (151 MHz, $CDCl_3$) δ 172.0, 140.1, 133.4, 132.7, 132.5, 130.1, 129.9, 128.6, 128.5, 128.4, 127.64, 127.61, 126.8, 126.7, 126.6, 74.6, 57.7, 52.5.

HRMS (MALDI$^+$) m/z calcd for $C_{20}H_{18}O_3SNa^+$ $[M+Na]^+$: 361.0869, found: 361.0872.

$[\alpha]_D^{RT}$ = +46.8 (c = 1.0 g/100 mL, $CHCl_3$).

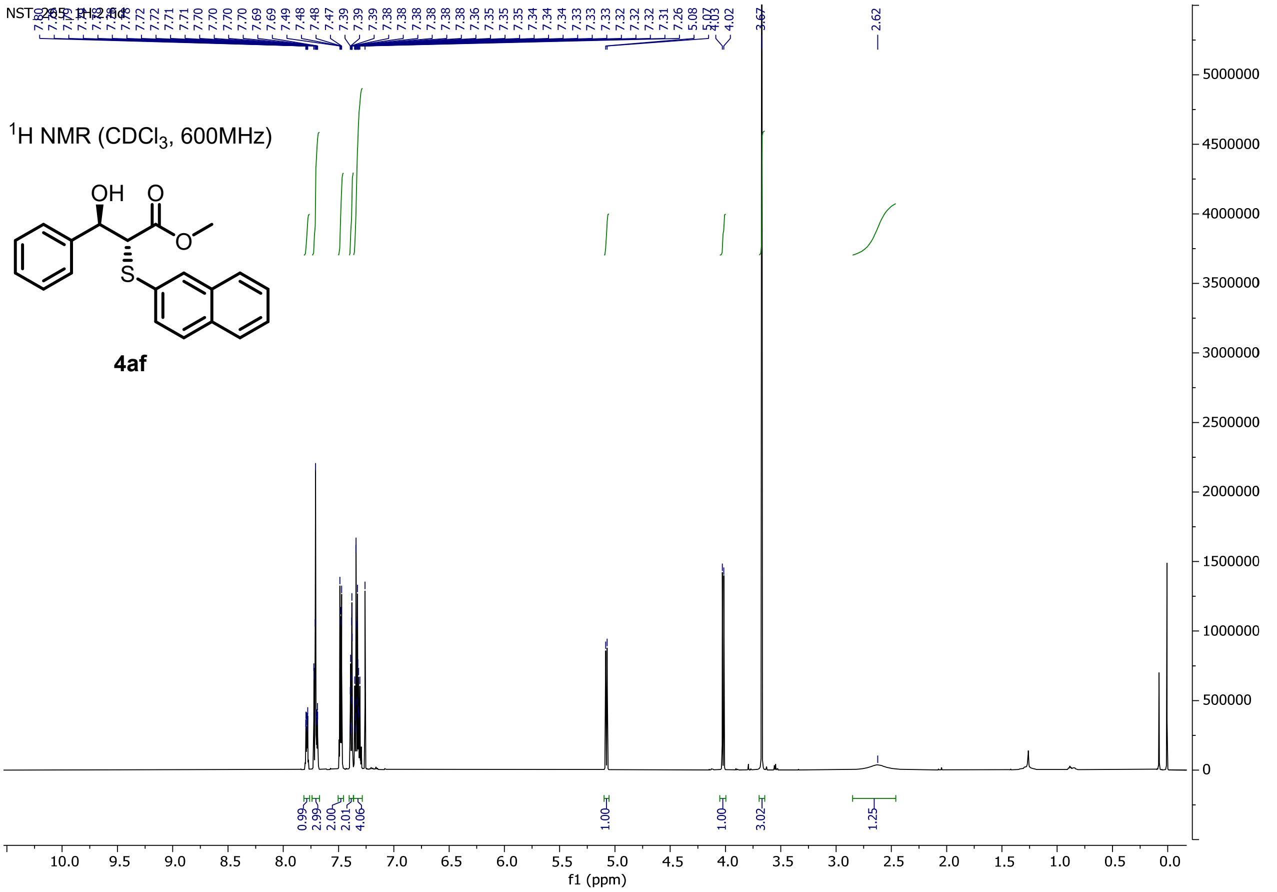
NST_265_1H.2.fid
1H NMR (CDCl3, 600MHz)
OH
O
O
S
4af
f1 (ppm)

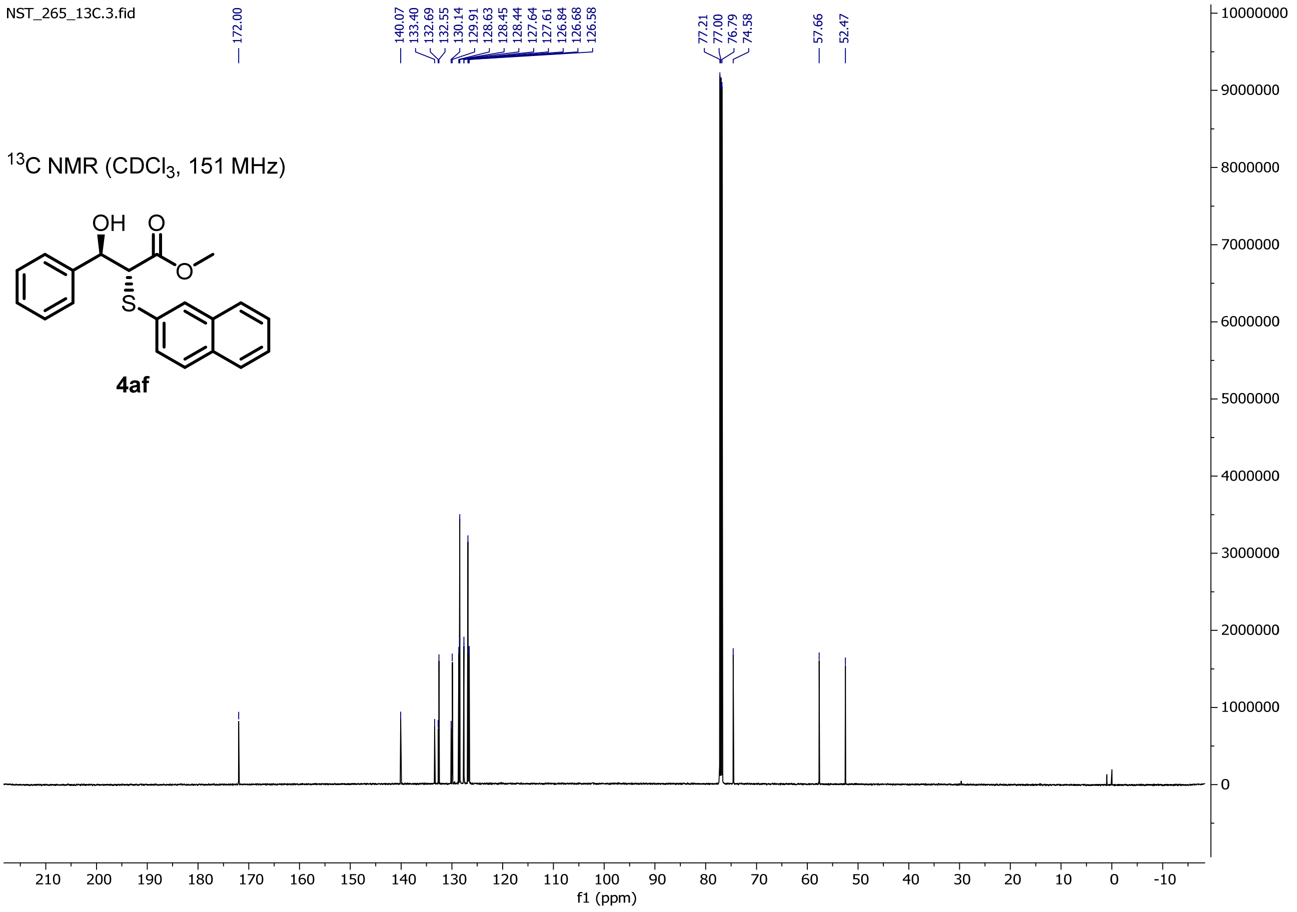
NST_265_13C.3.fid
13C NMR (CDCl3, 151 MHz)
OH
O
O
S
4af
f1 (ppm)

CSP HPLC traces:

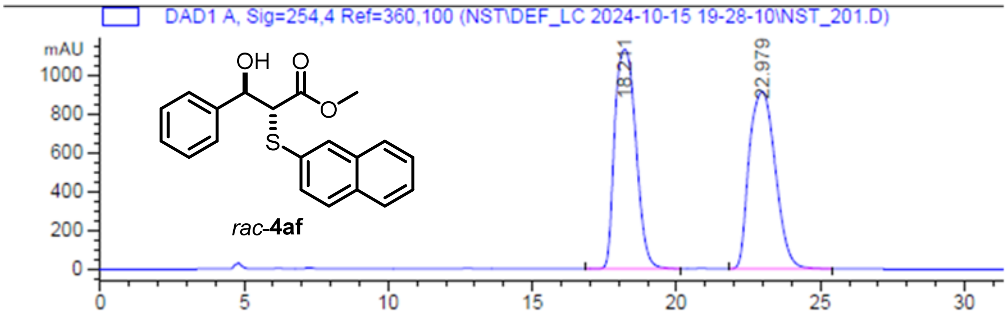

Signal 1: DAD1 A, Sig=254,4 Ref=360,100

Peak #	RetTime [min]	Type	Width [min]	Area [mAU*s]	Height [mAU]	Area %
1	18.211	BB	0.8365	5.76787e4	1131.89893	50.0190
2	22.979	BB	1.0301	5.76348e4	913.51489	49.9810

Totals : 1.15314e5 2045.41382

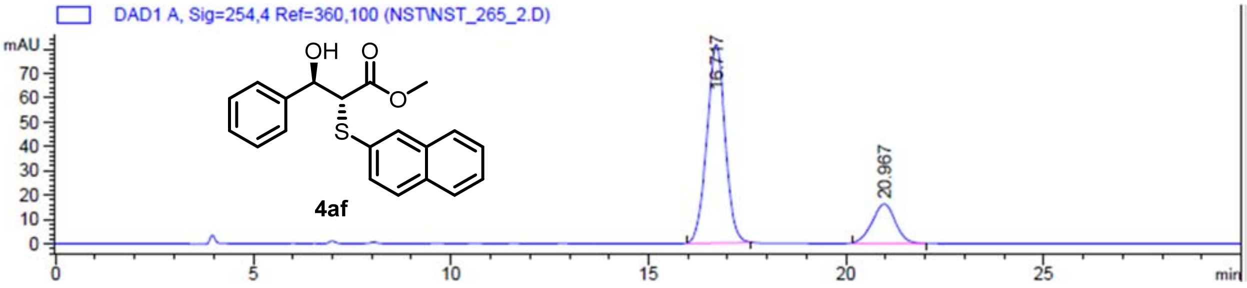

Signal 1: DAD1 A, Sig=254,4 Ref=360,100

Peak #	RetTime [min]	Type	Width [min]	Area [mAU*s]	Height [mAU]	Area %
1	16.717	BB	0.4912	2623.29761	81.56301	79.8311
2	20.967	BB	0.6139	662.76172	16.18090	20.1689

Totals : 3286.05933 97.74391

4ag- Methyl (2*R*,3*R*)-2-((4-cyanophenyl)thio)-3-hydroxy-3-phenylpropanoate

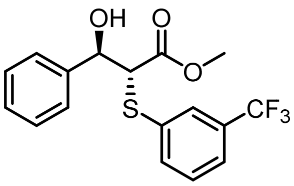

$C_{17}H_{15}F_3O_3S$

MW: 356.3 g/mol

Yield: 55%;

Colorless oil

The product (**4ag**) was prepared using the general procedure **C** from **1a** (methyl 2-(dimethyl(oxo)-λ^6-sulfaneylidene)acetate), **2a** (benzaldehyde), and **3j** (3-(trifluoromethyl)benzenethiol). The product was obtained as a colorless oil (19.5 mg; 55% yield). The diastereomeric ratio obtained for the *anti*-couple is greater than 20:1. The enantiomeric excess of the product was determined by CSP HPLC: AD-H column, *n*-hexane/iPrOH 80:20, 0.75 mL/min, t_{maj} = 10.2 min; t_{min} = 11.9 min, 88% ee.

^1H NMR (600 MHz, $CDCl_3$) δ 7.48 (br d, *J* = 7.7 Hz, 1H), 7.43 (br d, *J* = 7.7 Hz, 1H), 7.40 (br s, 1H), 7.38 – 7.28 (m, 6H), 5.03 (d, *J* = 8.4 Hz, 1H), 3.93 (d, *J* = 8.4 Hz, 1H), 3.71 (s, 3H).

^{13}C NMR (151 MHz, $CDCl_3$) δ 171.7, 139.7, 136.0, 134.5, 131.3 (q, *J* = 32.6 Hz), 129.41 (q, *J* = 7.3 Hz), 129.37, 128.7, 128.5, 126.8, 124.8 (q, *J* = 3.7 Hz), 124.4 (q, *J* = 272.7 Hz), 74.8, 57.5, 52.6.

^{19}F NMR (376 MHz, $CDCl_3$) δ -62.86 (s, 3F).

HRMS (MALDI$^+$) m/z calcd for $C_{17}H_{15}F_3O_3SNa^+$[M+Na]$^+$: 379.0586, found 379.0592.

$[\alpha]_D^{RT}$ = +18.6 (c = 1.0 g/100 mL, $CHCl_3$).

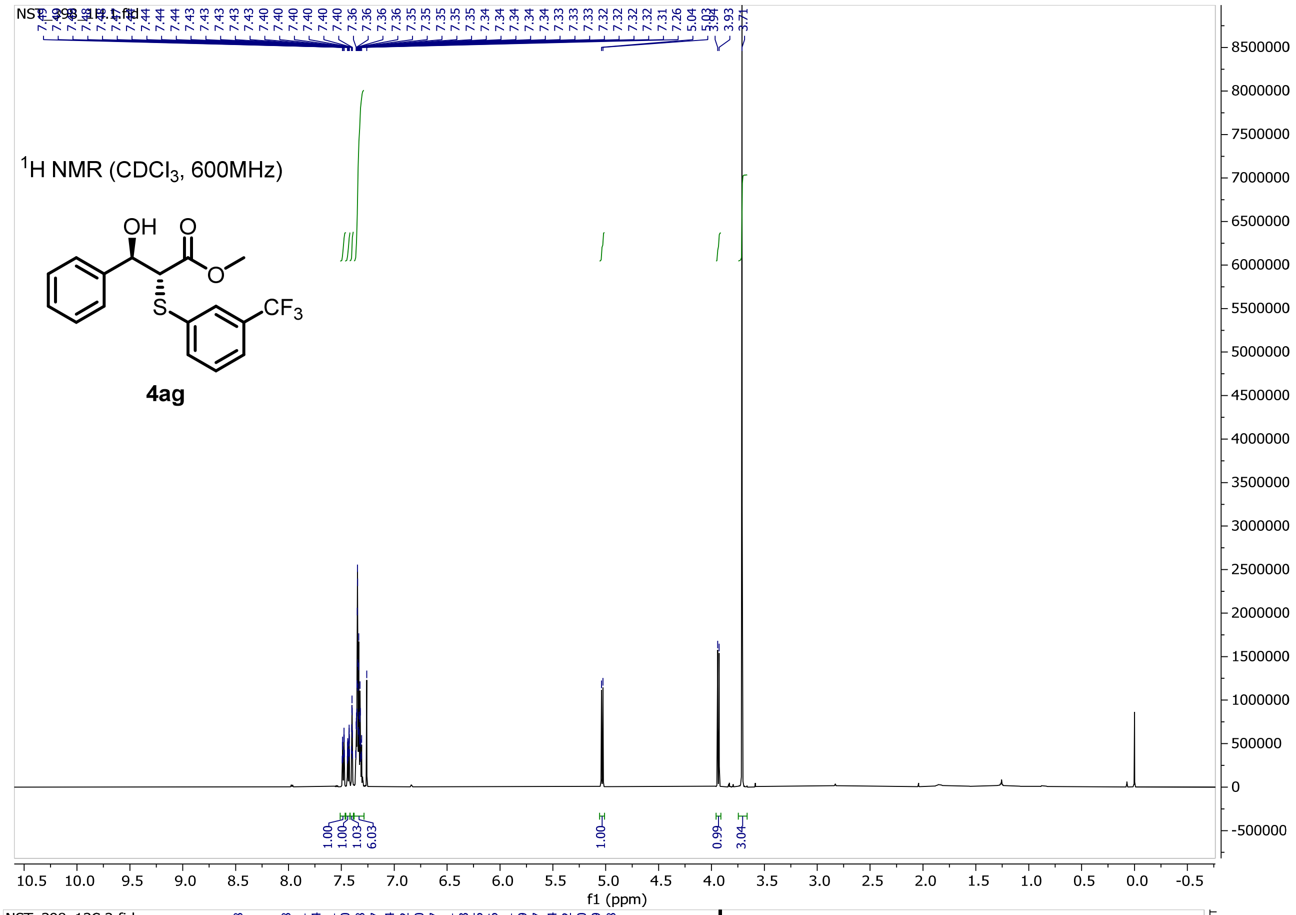

1H NMR (CDCl3, 600MHz)
OH
O
S
CF3
4ag
5.04
5.03
3.94
3.93
3.71
1.00
1.00
1.03
6.03
1.00
0.99
3.04
f1 (ppm)

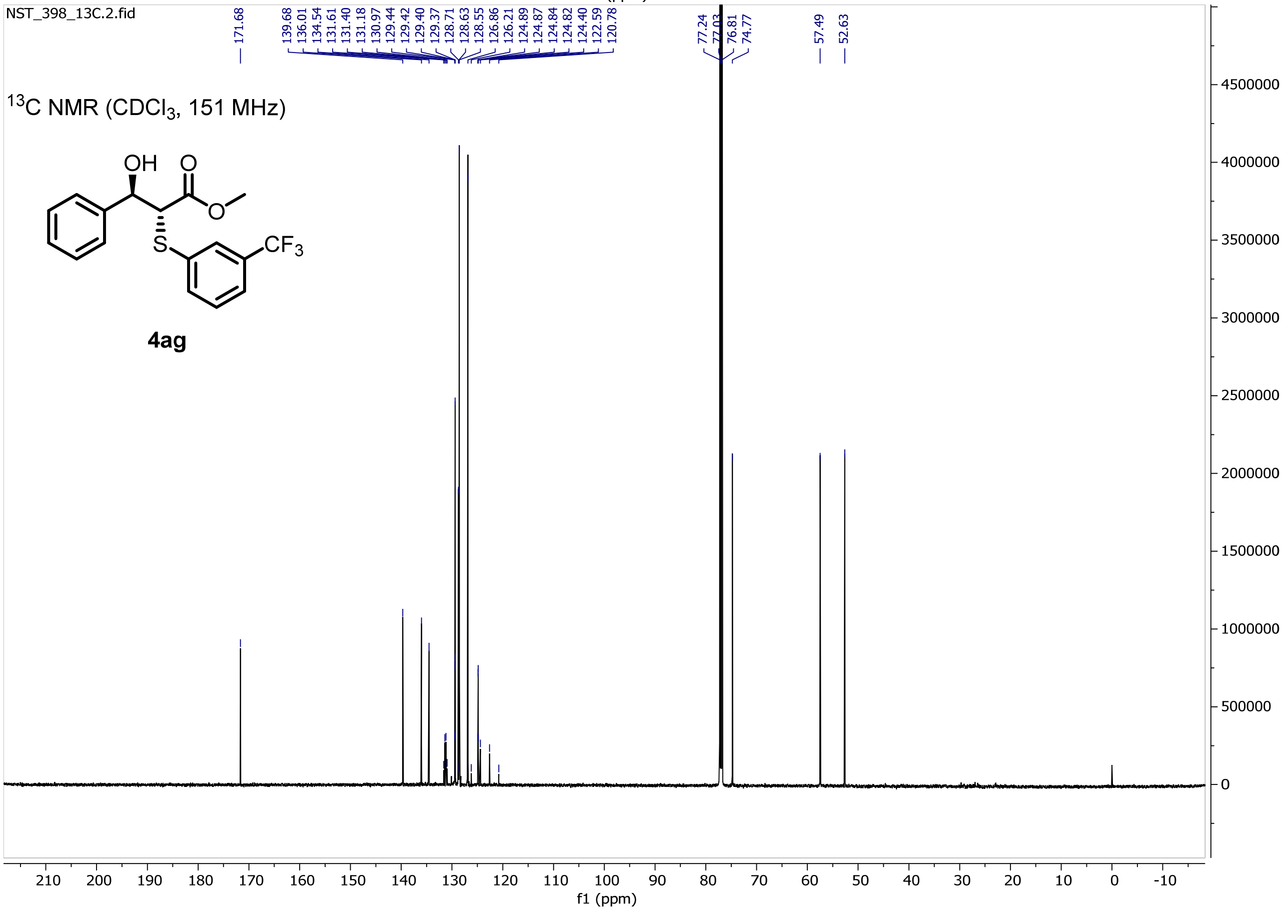

NST_398_13C.2.fid
171.68
139.68
136.01
134.54
131.61
131.40
131.18
130.97
129.44
129.42
129.40
129.37
128.71
128.63
128.55
126.86
126.21
124.89
124.87
124.84
124.82
124.40
122.59
120.78
77.24
77.03
76.81
74.77
57.49
52.63
13C NMR (CDCl3, 151 MHz)
OH
O
S
CF3
4ag
f1 (ppm)

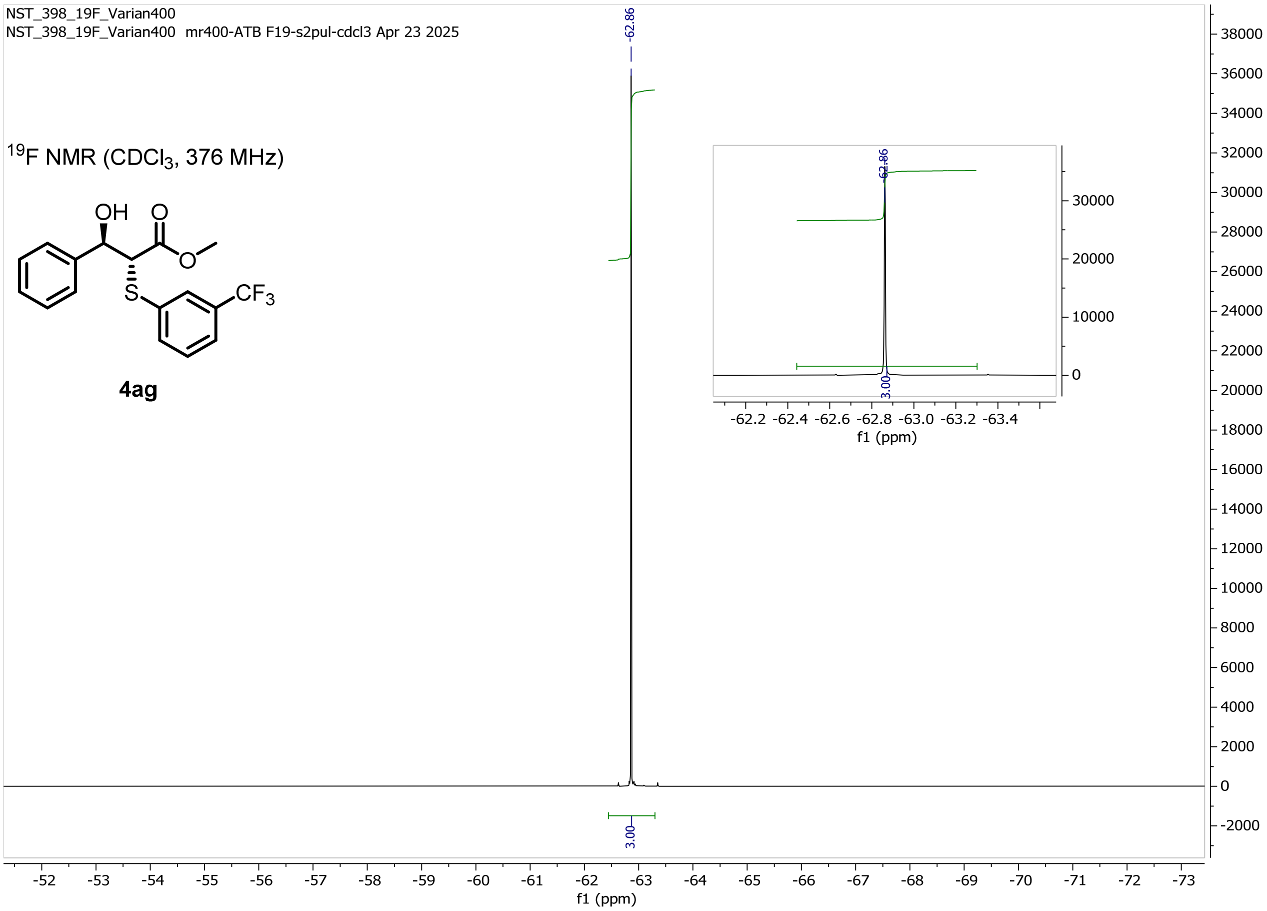
NST_398_19F_Varian400
NST_398_19F_Varian400 mr400-ATB F19-s2pul-cdcl3 Apr 23 2025
-62.86
19F NMR (CDCl3, 376 MHz)
OH
O
S
CF3
4ag
3.00
f1 (ppm)

CSP HPLC traces:

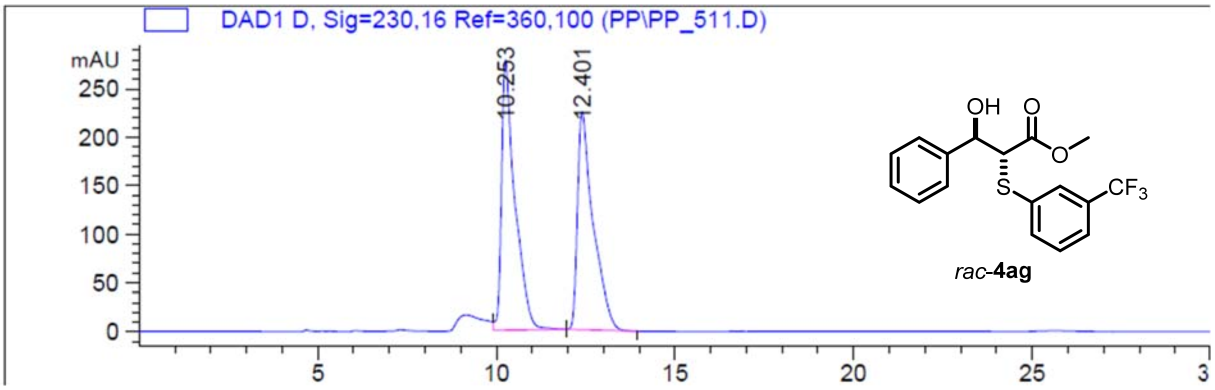

Signal 2: DAD1 D, Sig=230,16 Ref=360,100

Peak #	RetTime [min]	Type	Width [min]	Area [mAU*s]	Height [mAU]	Area %
1	10.253	VB	0.3622	7217.52002	278.66309	51.4273
2	12.401	BB	0.4290	6816.88672	224.04103	48.5727
Totals :				1.40344e4	502.70412	

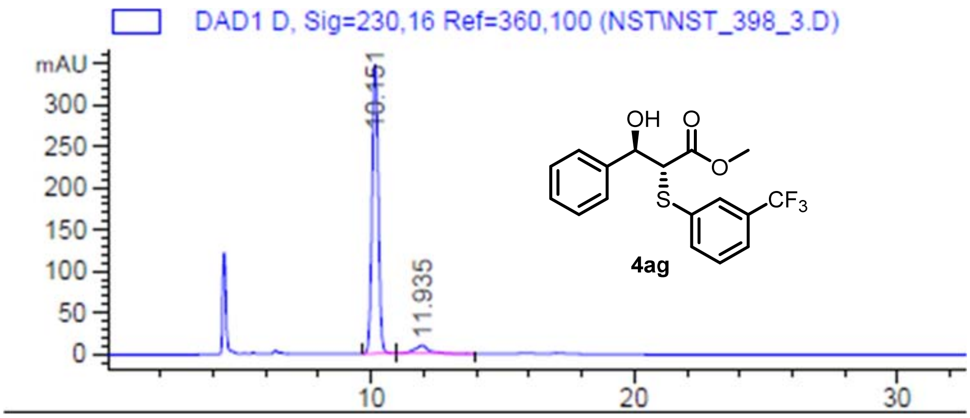

Signal 3: DAD1 D, Sig=230,16 Ref=360,100

Peak #	RetTime [min]	Type	Width [min]	Area [mAU*s]	Height [mAU]	Area %
1	10.151	BB	0.2511	5638.66162	347.80576	94.0336
2	11.935	BB	0.5101	357.77301	9.70545	5.9664
Totals :				5996.43463	357.51121	

4ah- Methyl (2*R*,3*R*)-2-((3-chlorophenyl)thio)-3-hydroxy-3-phenylpropanoate

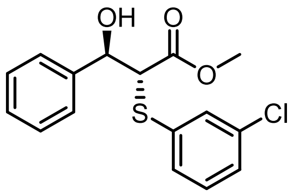

$C_{16}H_{15}ClO_3S$

MW: 322.8 g/mol

Yield: 80%

Colorless oil

The product (**4ah**) was prepared using the general procedure **C** from **1a** (methyl 2-(dimethyl(oxo)-λ^6-sulfaneylidene)acetate), **2a** (benzaldehyde), and **3k** (3-chlorobenzenethiol). The product was obtained as a colorless oil (27.2 mg; 80% yield). The diastereomeric ratio obtained for the *anti*-couple is greater than 20:1. The enantiomeric excess of the product was determined by CSP HPLC: AD-H column, *n*-hexane/iPrOH 80:20, 0.75 mL/min, t_{maj} = 11.3 min; t_{min} = 14.6 min, 79% ee.

^{1}H NMR (600 MHz, $CDCl_3$) δ 7.36 – 7.30 (m, 5H), 7.21 (dt, *J* = 7.3, 2.0 Hz, 1H), 7.17 – 7.12 (m, 3H), 5.02 (d, *J* = 8.2 Hz, 1H), 3.91 (d, *J* = 8.2 Hz, 1H), 3.70 (s, 3H), 3.05 (br s, 1H).

^{13}C NMR (151 MHz, $CDCl_3$) δ 171.7, 139.8, 135.0, 134.5, 132.5, 130.8, 129.9, 128.6, 128.5, 128.3, 126.8, 74.7, 57.6, 52.6.

HRMS (MALDI$^+$) m/z calcd for $C_{16}H_{15}ClO_3SNa^+$[M+Na]$^+$: 345.0323, found: 345.0328.

$[\alpha]_D^{RT}$ = +49.4 (c = 1.0 g/100 mL, $CHCl_3$).

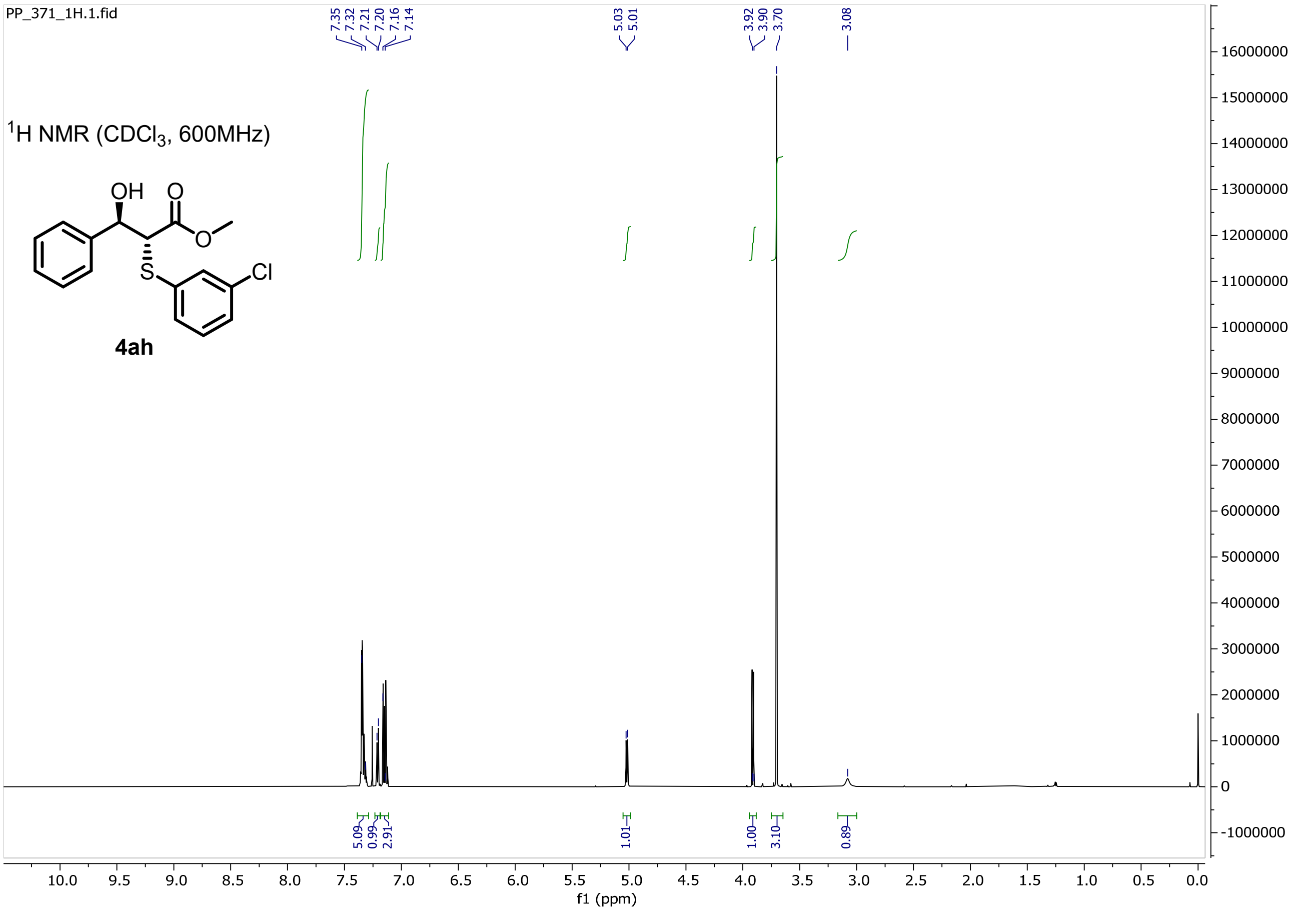
PP_371_1H.1.fid
^1H NMR ($CDCl_3$, 600MHz)
OH
O
O
S
Cl
4ah
7.35
7.32
7.21
7.20
7.16
7.14
5.03
5.01
3.92
3.90
3.70
3.08
5.09
0.99
2.91
1.01
1.00
3.10
0.89
f1 (ppm)

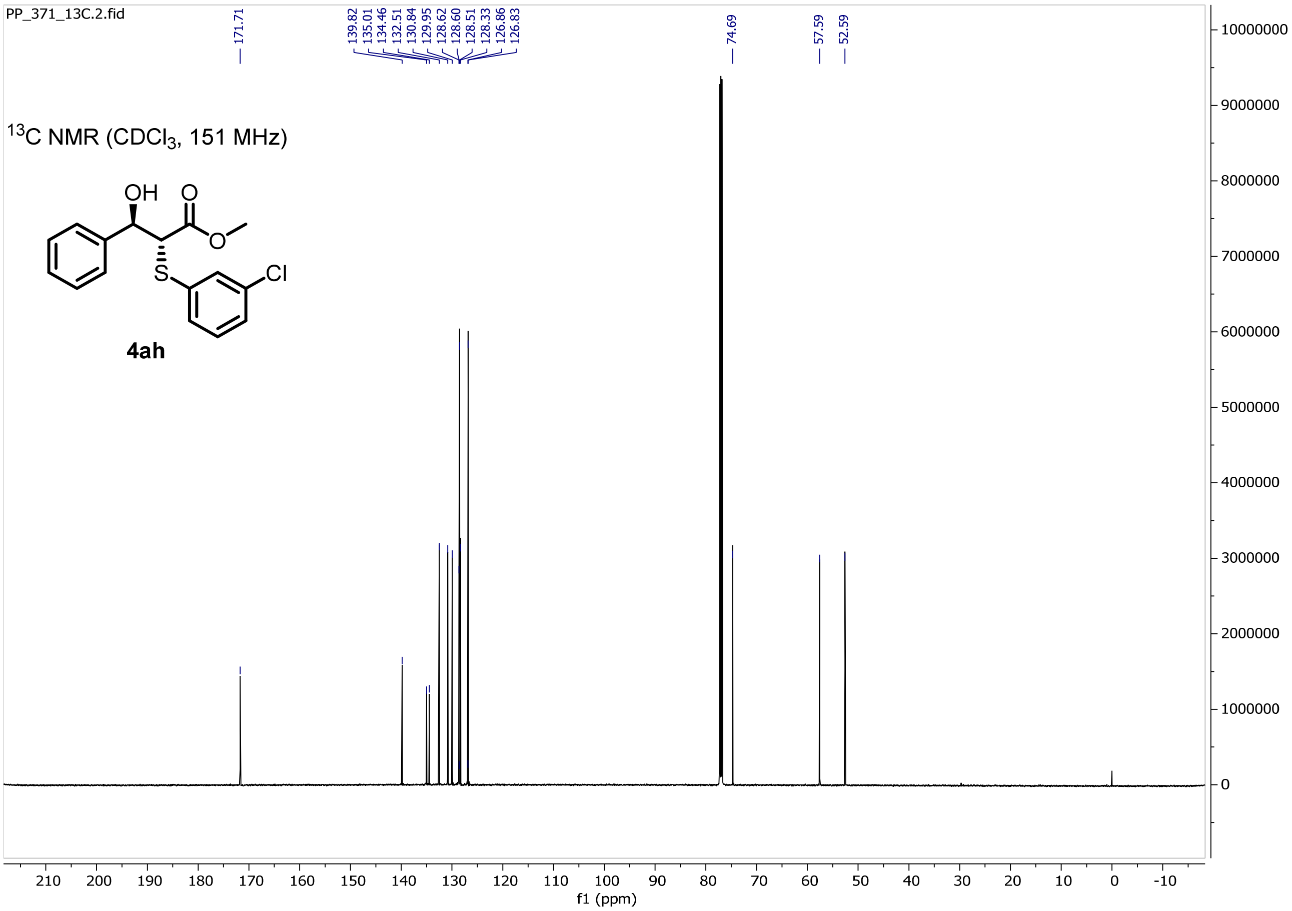
PP_371_13C.2.fid
^{13}C NMR ($CDCl_3$, 151 MHz)
OH
O
O
S
Cl
4ah
171.71
139.82
135.01
134.46
132.51
130.84
129.95
128.62
128.60
128.51
128.33
126.86
126.83
74.69
57.59
52.59
f1 (ppm)

CSP HPLC traces:

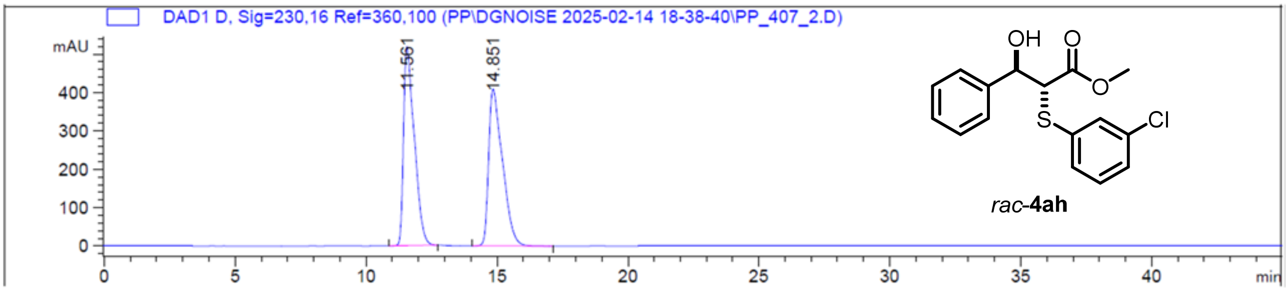

Signal 3: DAD1 D, Sig=230,16 Ref=360,100

Peak #	RetTime [min]	Type	Width [min]	Area [mAU*s]	Height [mAU]	Area %
1	11.561	BB	0.4057	1.47627e4	519.20398	49.6337
2	14.851	BB	0.5257	1.49807e4	408.68027	50.3663

Totals : 2.97434e4 927.88425

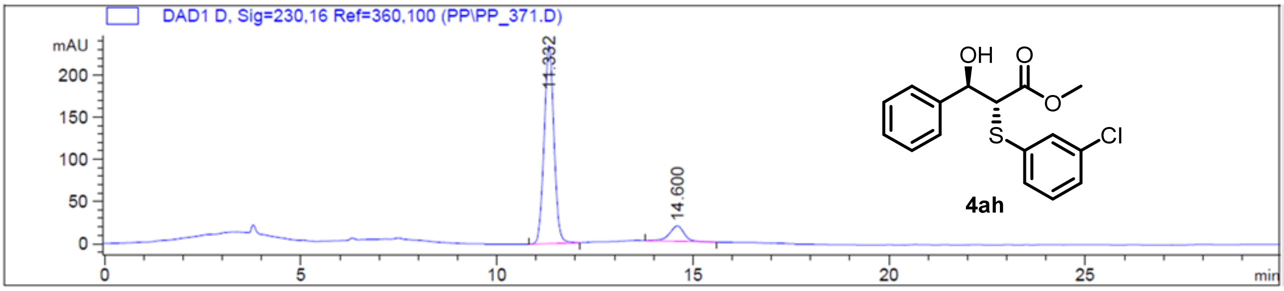

Signal 3: DAD1 D, Sig=230,16 Ref=360,100

Peak #	RetTime [min]	Type	Width [min]	Area [mAU*s]	Height [mAU]	Area %
1	11.332	BB	0.2682	4064.90405	234.53128	89.5529
2	14.600	BB	0.3911	474.20560	18.24978	10.4471

Totals : 4539.10965 252.78106

4ai- Methyl (2*R*,3*R*)-3-hydroxy-2-((4-methoxyphenyl)thio)-3-phenylpropanoate

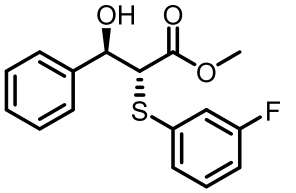

$C_{16}H_{15}FO_3S$

MW: 306.3 g/mol

Yield: 75%;

Colorless oil

The product (**4ai**) was prepared using the general procedure **C** from **1a** (methyl 2-(dimethyl(oxo)-λ^6-sulfaneylidene)acetate), **2a** (benzaldehyde), and **3l** (3-fluorobenzenethiol). The product was obtained as a colorless oil (23 mg; 75% yield). The diastereomeric ratio obtained for the *anti*-couple is greater than 20:1. The enantiomeric excess of the product was determined by CSP HPLC: AD-H column, *n*-hexane/iPrOH 80:20, 0.75 mL/min, t_{maj} = 12.9 min; t_{min} = 16.6 min, 72% ee.

^{1}H NMR (600 MHz, $CDCl_3$) δ 7.39 – 7.29 (m, 5H), 7.19 (m, 1H), 7.05 – 7.01 (m, 1H), 6.97 – 6.91 (m, 2H), 5.03 (d, *J* = 8.1 Hz, 1H), 3.94 (d, *J* = 8.1 Hz, 1H), 3.70 (s, 3H).

^{13}C NMR (151 MHz, $CDCl_3$) δ 171.8, 162.4 (d, *J* = 250.7 Hz), 139.8, 135.2 (d, *J* = 7.7 Hz), 130.2 (d, *J* = 8.5 Hz), 128.61, 128.57, 128.2 (d, *J* = 3.0 Hz), 126.8, 119.4 (d, *J* = 22.4 Hz), 115.2 (d, *J* = 21.2 Hz), 74.6, 57.5, 52.6.

^{19}F NMR (376 MHz, $CDCl_3$) δ -111.7 (m, 1F).

HRMS (MALDI$^+$) m/z calcd for $C_{16}H_{15}FO_3SNa^+[M+Na]^+$: 329.0618, found: 329.0623.

$[\alpha]_D^{RT}$ = +33.7 (c = 1.0 g/100 mL, $CHCl_3$).

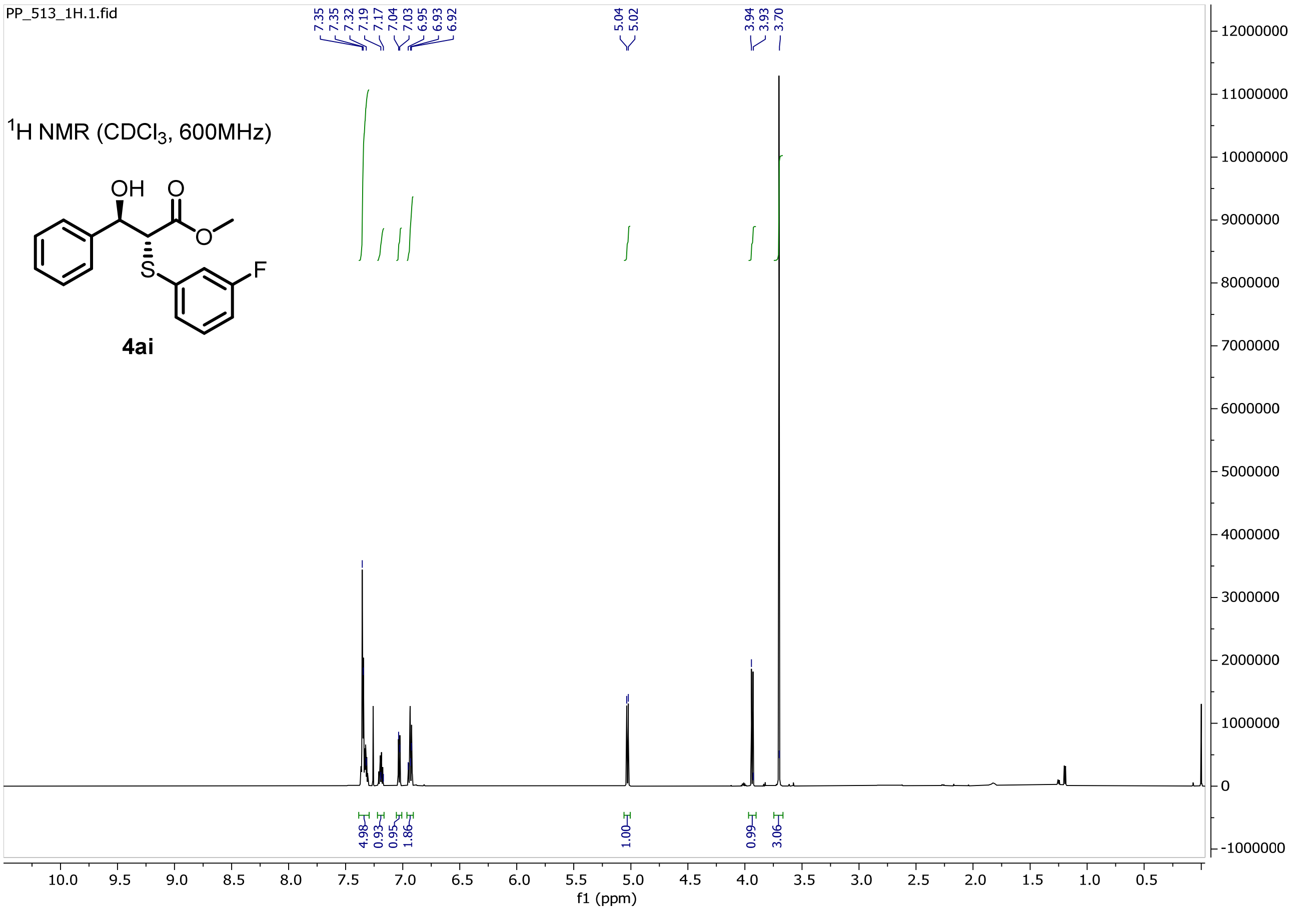
PP_513_1H.1.fid
1H NMR (CDCl3, 600MHz)
OH
O
O
S
F
4ai
7.35
7.35
7.32
7.19
7.17
7.04
7.03
6.95
6.93
6.92
5.04
5.02
3.94
3.93
3.70
4.98
0.93
0.95
1.86
1.00
0.99
3.06
f1 (ppm)

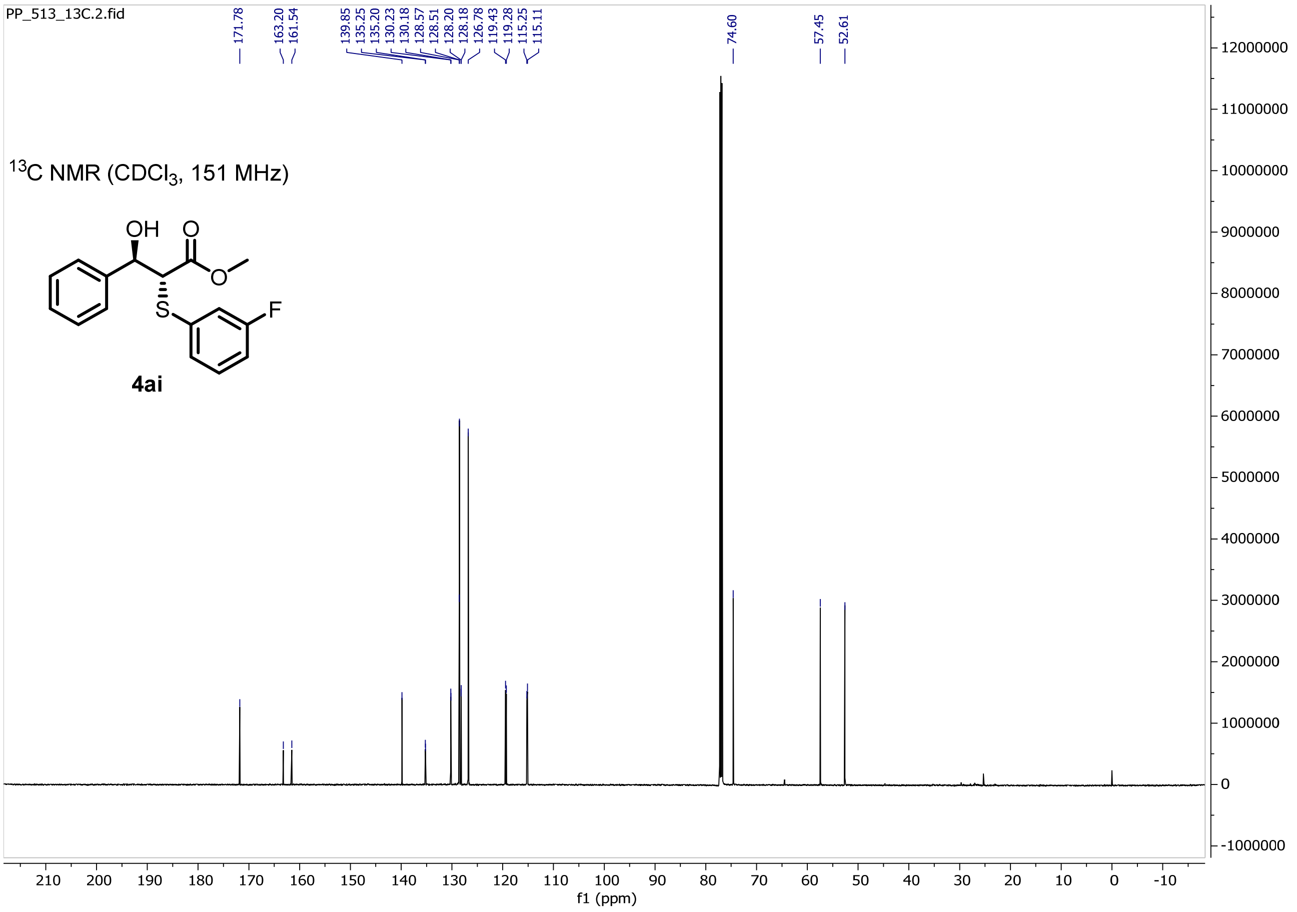
PP_513_13C.2.fid
13C NMR (CDCl3, 151 MHz)
OH
O
O
S
F
4ai
171.78
163.20
161.54
139.85
135.25
135.20
130.23
130.18
128.57
128.51
128.20
128.18
126.78
119.43
119.28
115.25
115.11
74.60
57.45
52.61
f1 (ppm)

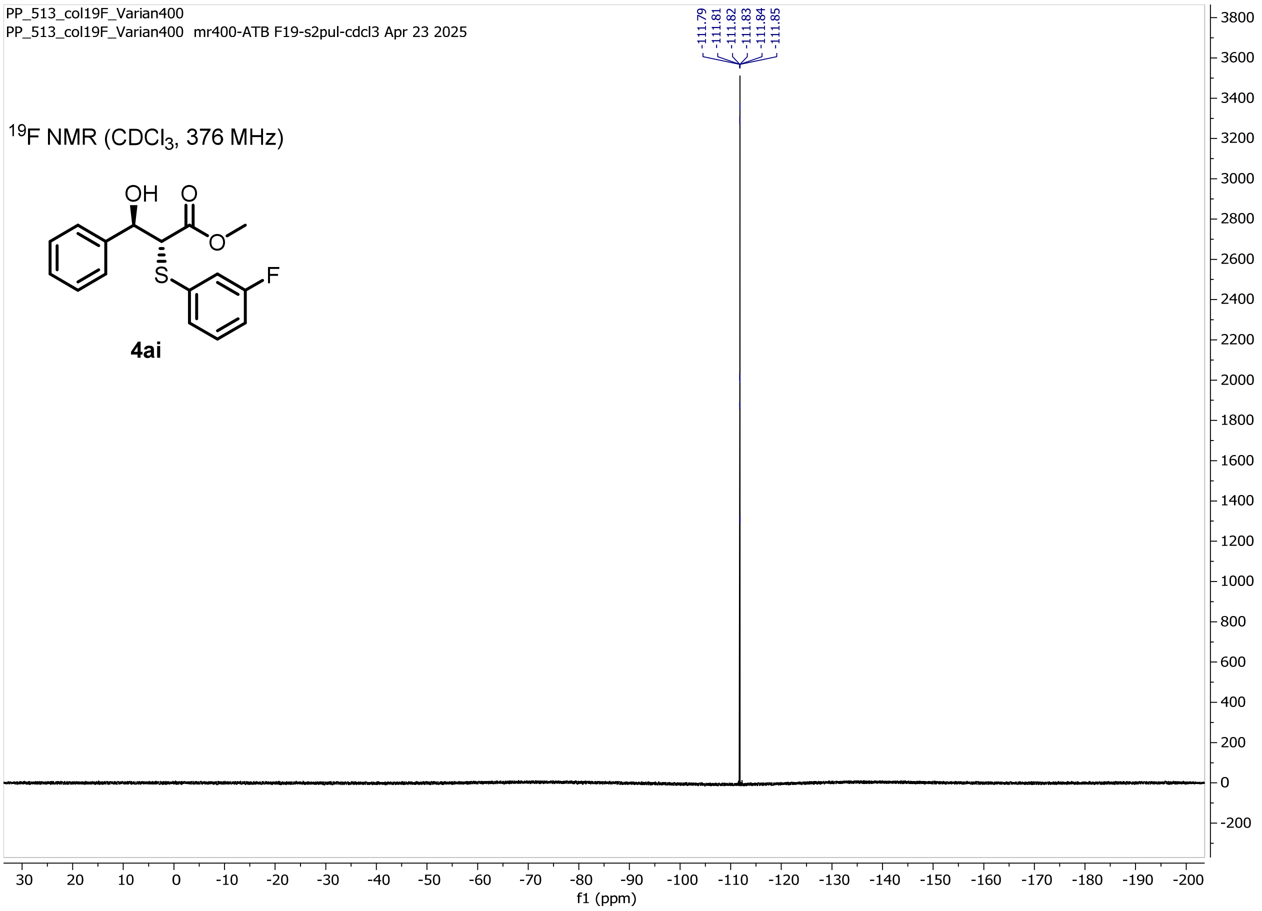
PP_513_col19F_Varian400
PP_513_col19F_Varian400 mr400-ATB F19-s2pul-cdcl3 Apr 23 2025
-111.79
-111.81
-111.82
-111.83
-111.84
-111.85
19F NMR (CDCl3, 376 MHz)
OH
O
O
S
F
4ai
f1 (ppm)

CSP HPLC traces:

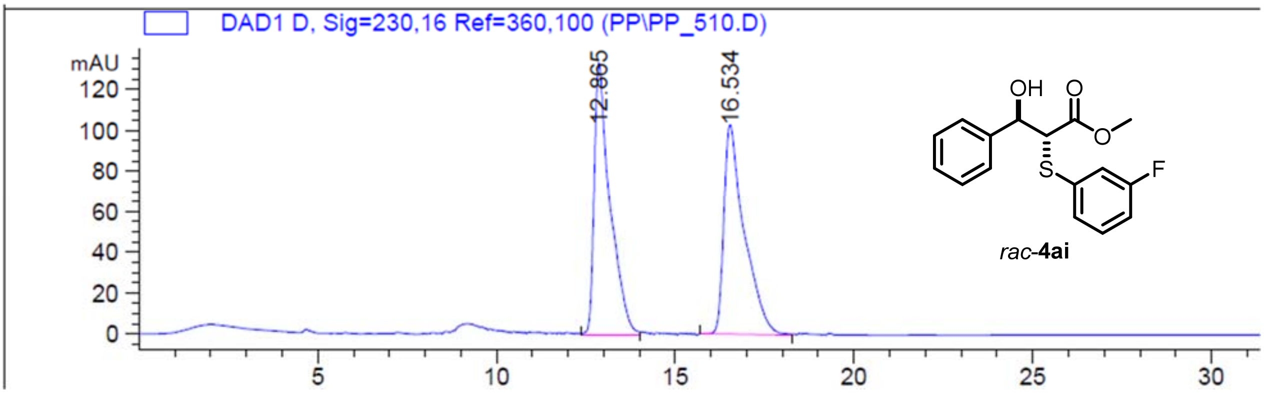

Signal 2: DAD1 D, Sig=230,16 Ref=360,100

Peak #	RetTime [min]	Type	Width [min]	Area [mAU*s]	Height [mAU]	Area %
1	12.865	BV	0.4343	4091.53418	133.23271	49.9829
2	16.534	BV	0.5602	4094.33423	102.89901	50.0171

Totals : 8185.86841 236.13172

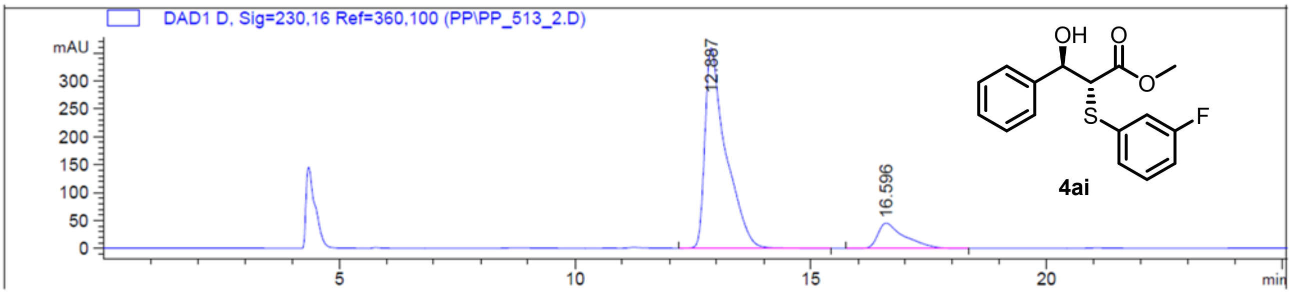

Signal 2: DAD1 D, Sig=230,16 Ref=360,100

Peak #	RetTime [min]	Type	Width [min]	Area [mAU*s]	Height [mAU]	Area %
1	12.887	BB	0.4330	1.10520e4	359.14740	86.2055
2	16.596	BB	0.5497	1768.53613	45.27699	13.7945

Totals : 1.28206e4 404.42439

4aj- Methyl (2*R*,3*R*)-2-((2-chlorophenyl)thio)-3-hydroxy-3-phenylpropanoate

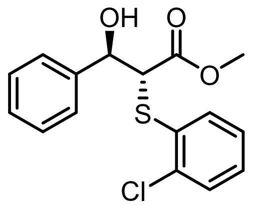

$C_{16}H_{15}ClO_3S$

MW: 322.8 g/mol

Yield: 90%

Colorless oil

The product (**4aj**) was prepared using the general procedure **C** from **1a** (methyl 2-(dimethyl(oxo)-λ^6-sulfaneylidene)acetate), **2a** (benzaldehyde), and **3m** (2-chlorobenzenethiol). The product was obtained as a colorless oil (29 mg; 90% yield). The diastereomeric ratio obtained for the *anti*-couple is greater than 20:1. The enantiomeric excess of the product was determined by CSP HPLC: AD-H column, *n*-hexane/iPrOH 80:20, 0.75 mL/min, t_{maj} = 12.6 min; t_{min} = 14.1 min, 83% ee.

^{1}H NMR (600 MHz, $CDCl_3$) δ 7.40 – 7.36 (m, 4H), 7.35 – 7.27 (m, 3H), 7.21 (td, *J* = 7.9, 1.7 Hz, 1H), 7.14 (td, *J* = 7.6, 1.4 Hz, 1H), 5.10 (d, *J* = 7.1 Hz, 1H), 4.10 (d, *J* = 7.1 Hz, 1H), 3.61 (s, 3H), 3.28 (br s, 1H).

^{13}C NMR (151 MHz, $CDCl_3$) δ 171.6, 140.1, 137.5, 134.8, 131.9, 130.1, 129.6, 128.5, 128.4, 127.2, 126.5, 74.5, 56.0, 52.4.

HRMS (MALDI$^+$) m/z calcd for $C_{16}H_{15}ClO_3SNa^+$[M+Na]$^+$:345.0323, found: 345.0328.

$[\alpha]_D^{RT}$ = +59.1 (c = 1.0 g/100 mL, $CHCl_3$).

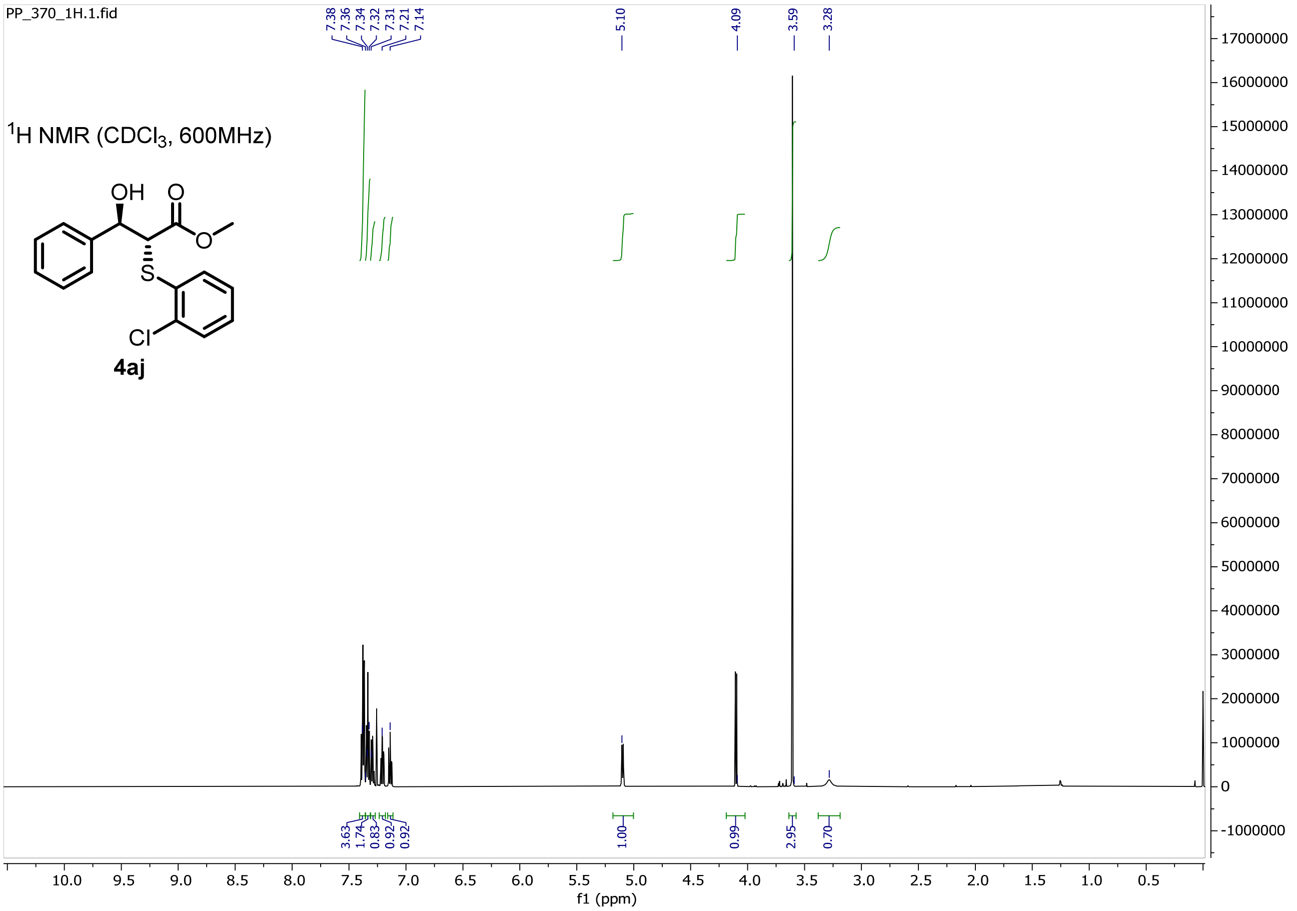
PP_370_1H.1.fid
7.38 7.36 7.34 7.32 7.31 7.21 7.14
5.10
4.09
3.59
3.28
^{1}H NMR (CDCl$_3$, 600MHz)
OH O
O
S
Cl
4aj
3.63 1.74 0.83 0.92 0.92
1.00
0.99
2.95
0.70
f1 (ppm)

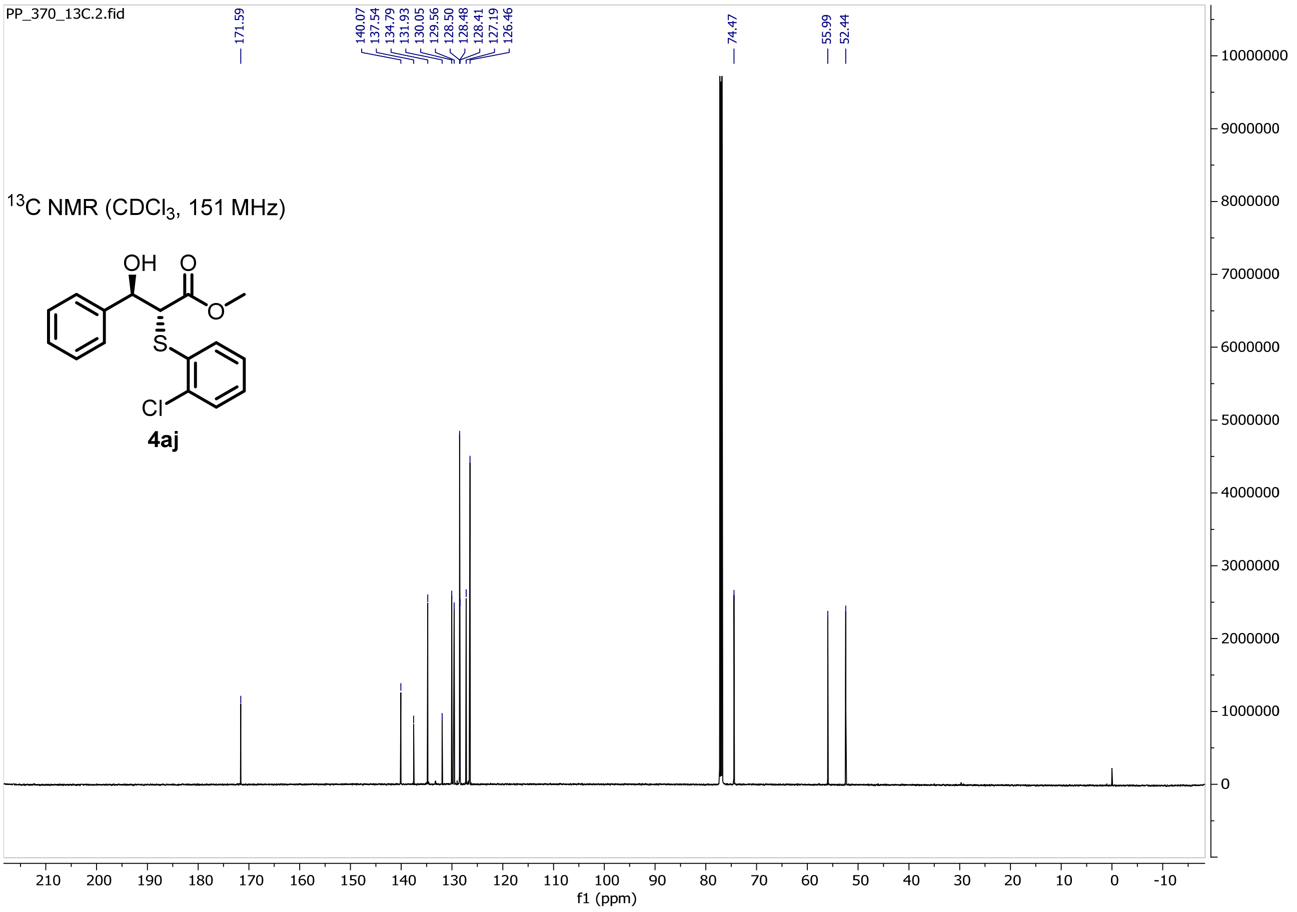
PP_370_13C.2.fid
171.59
140.07 137.54 134.79 131.93 130.05 129.56 128.50 128.48 128.41 127.19 126.46
74.47
55.99
52.44
^{13}C NMR (CDCl$_3$, 151 MHz)
OH O
O
S
Cl
4aj
f1 (ppm)

CSP HPLC traces:

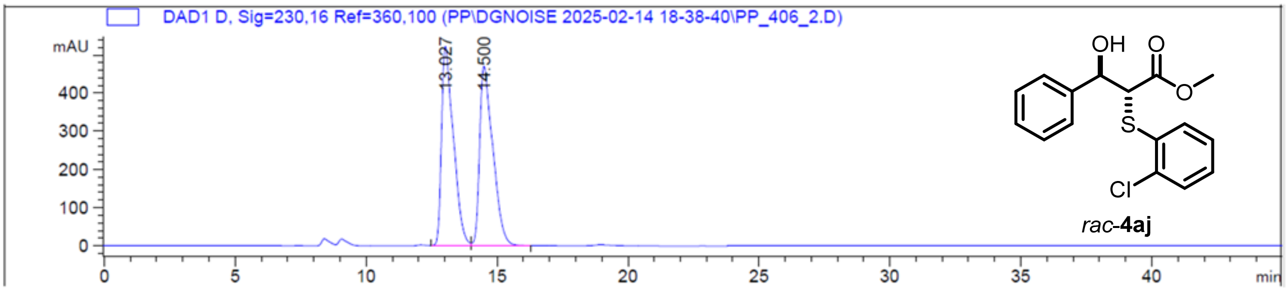

Signal 3: DAD1 D, Sig=230,16 Ref=360,100

Peak #	RetTime [min]	Type	Width [min]	Area [mAU*s]	Height [mAU]	Area %
1	13.027	BV	0.4475	1.63483e4	521.85071	49.8622
2	14.500	VB	0.5006	1.64386e4	469.41165	50.1378
Totals :				3.27869e4	991.26236	

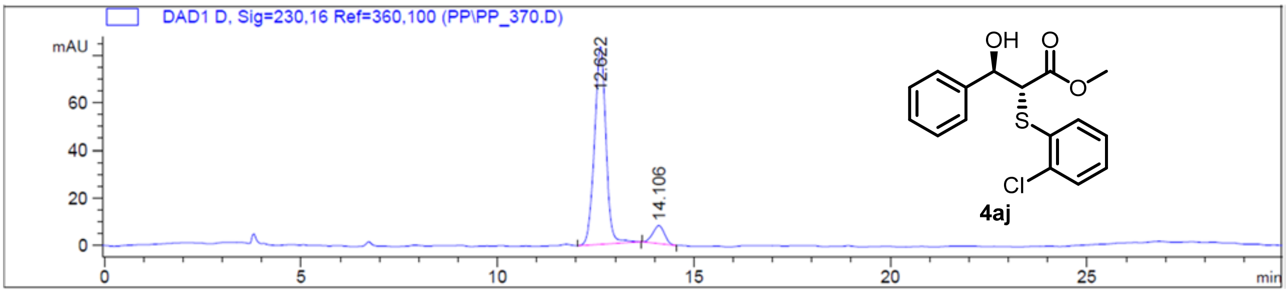

Signal 3: DAD1 D, Sig=230,16 Ref=360,100

Peak #	RetTime [min]	Type	Width [min]	Area [mAU*s]	Height [mAU]	Area %
1	12.622	BB	0.3104	1685.99451	83.20709	91.4738
2	14.106	BB	0.3180	157.15027	7.63946	8.5262
Totals :				1843.14478	90.84656	

Synthetic elaborations

Reduction of the ester moiety[27]

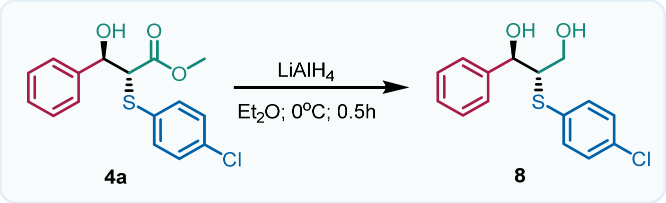

8 - (1*R*,2*S*)-2-((4-Chlorophenyl)thio)-1-phenylpropane-1,3-diol

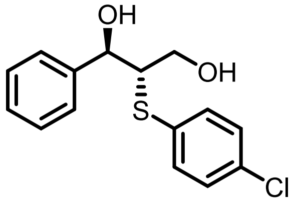

$C_{15}H_{15}ClO_2S$

MW: 294.8 g/mol

Yield: 64%

Colorless oil

In a 5mL two neck round bottom flask equipped with a magnetic stirring bar, $LiAlH_4$ (0.12 mmol, 1.2 equiv., 62.5 µL of a 2.4 M solution in THF) was added to a solution of **4a** (0.1 mmol, 1 equiv., 32.3 mg), in Et_2O (0.2 mL; 0.5 M), , cooled to 0 °C with an ice bath. The resulting solution was stirred at 0 °C for 30 minutes. The mixture was quenched with a few drops of water, then extracted with EtOAc (3x 10 mL). The organic phases were combined and washed with a saturated solution of sodium chloride, then dried with Na_2SO_4 and filtered. Next, the solvent was evaporated under vacuum and the residue analyzed by ^{1}H NMR to determine the diastereomeric ratio (> 20:1). Flash chromatography (*n*-hexane/EtOAc mixtures) afforded compound **8** in 64% yield (19.0 mg). The enantiomeric excess of the product was determined by CSP HPLC: AS-H column, *n*-hexane/iPrOH 80:20, 0.75 mL/min, t_{maj} = 12.3 min; t_{min} = 10.7 min, 87% ee, and was found to be consistent with the ee value of starting **4a**.

^{1}H NMR (400 MHz, $CDCl_3$) δ 7.38 – 7.20 (m, 9H), 4.93 (d, *J* = 5.6 Hz, 1H), 3.90 – 3.75 (m, 2H), 3.41 (dt, *J* = 5.9, 4.9 Hz, 1H), 3.01 (br s, 1H), 2.60 (br s, 1H).

^{13}C NMR (151 MHz, $CDCl_3$) δ 141.1, 133.8, 133.7, 132.3, 129.2, 128.5, 128.1, 126.3, 75.8, 62.5, 58.4.

HRMS (MALDI$^+$) m/z calcd for $C_{25}H_{25}ClO_2SNa^+$ [M+Na]$^+$: 447.1156, found 447.1161.

$[\alpha]_D^{RT}$ = +12.4 (c = 1.0 g/100 mL, $CHCl_3$).

---

[27] Procedure adapted from: E. J. Corey, S. Choi, *Tetrahedron Lett.* **2000**, *41*, 2769–2772.

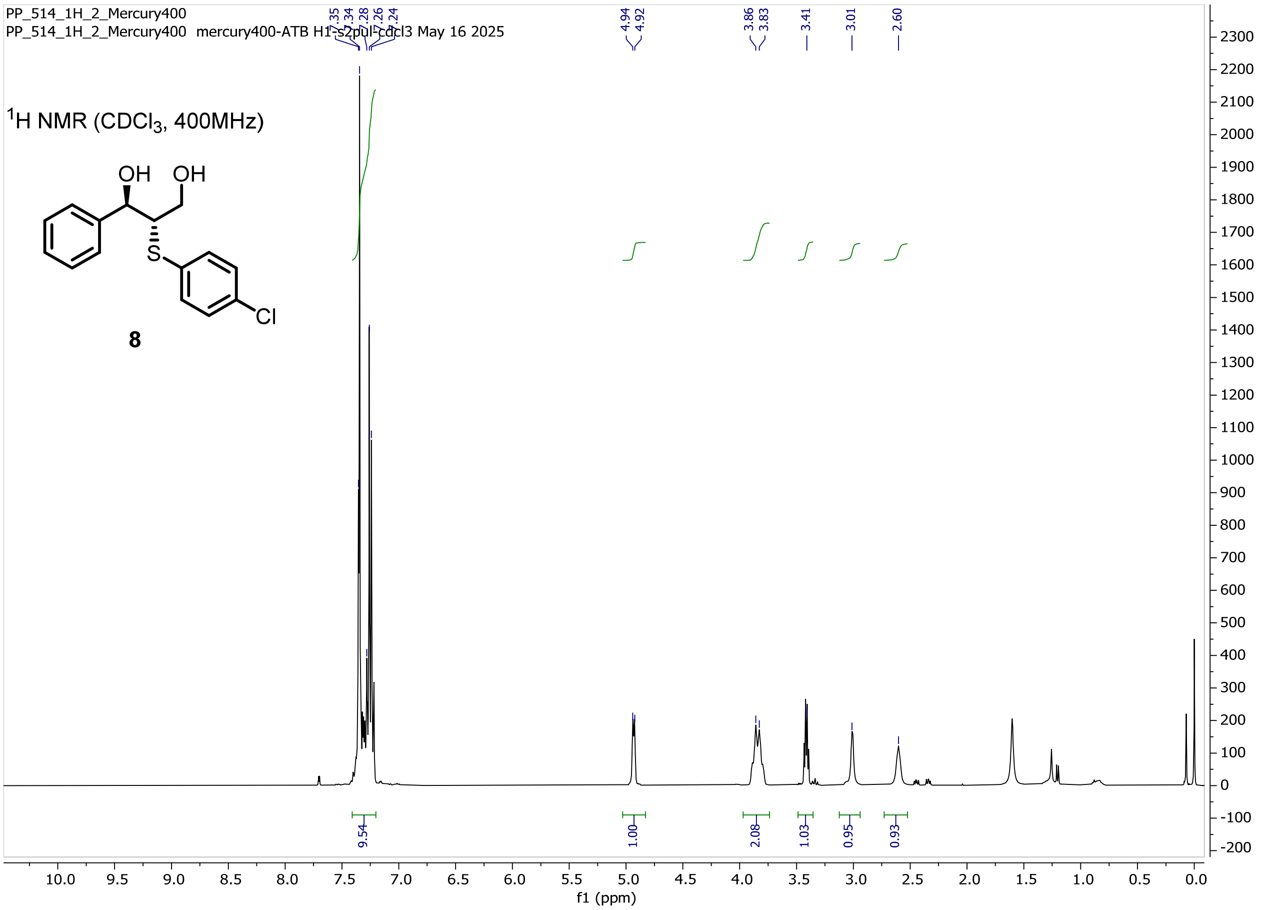
PP_514_1H_2_Mercury400
PP_514_1H_2_Mercury400 mercury400-ATB H1-s2pul-cdcl3 May 16 2025
1H NMR (CDCl3, 400MHz)
OH
OH
S
Cl
8
7.35
7.34
7.28
7.26
7.24
4.94
4.92
3.86
3.83
3.41
3.01
2.60
9.54
1.00
2.08
1.03
0.95
0.93
f1 (ppm)

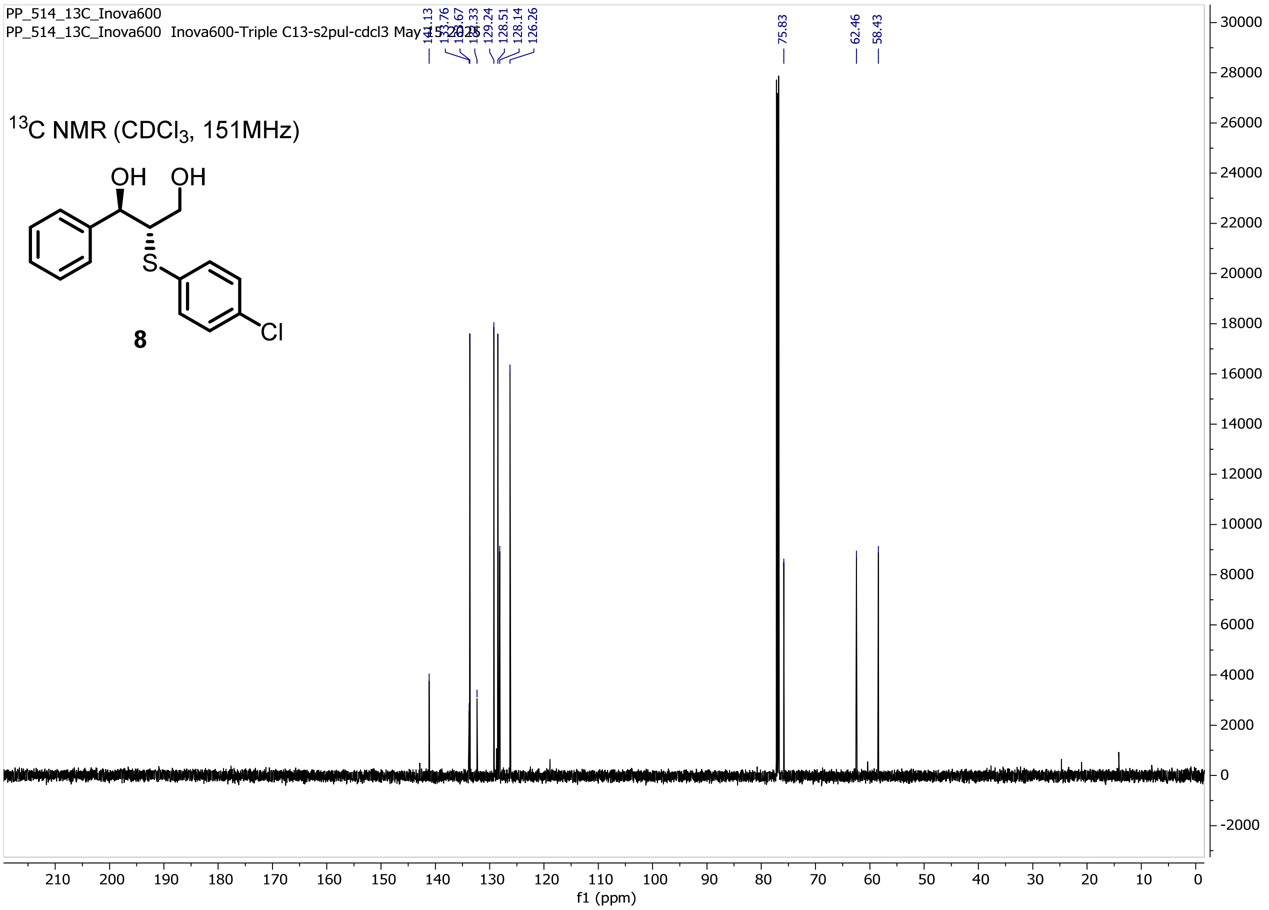
PP_514_13C_Inova600
PP_514_13C_Inova600 Inova600-Triple C13-s2pul-cdcl3 May 15 2025
13C NMR (CDCl3, 151MHz)
OH
OH
S
Cl
8
141.13
133.76
133.67
132.33
129.24
128.51
128.14
126.26
75.83
62.46
58.43
f1 (ppm)

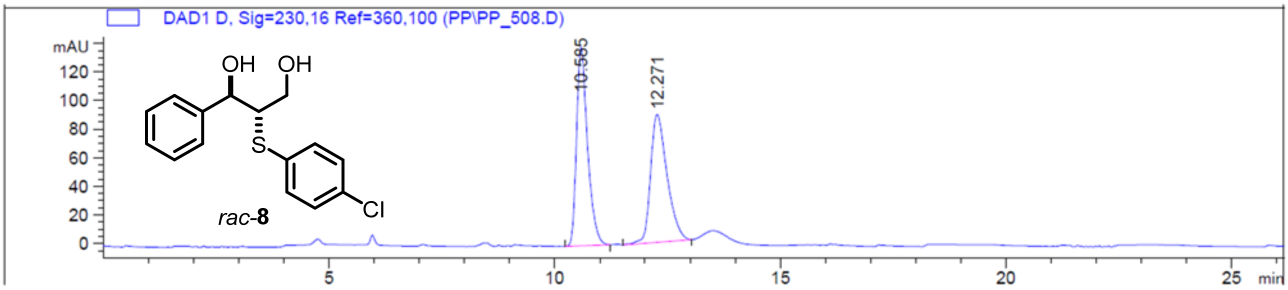

Signal 2: DAD1 D, Sig=230,16 Ref=360,100

Peak #	RetTime [min]	Type	Width [min]	Area [mAU*s]	Height [mAU]	Area %
1	10.585	BB	0.2518	2354.09790	138.83966	50.8368
2	12.271	BB	0.3829	2276.60083	89.46475	49.1632
Totals :				4630.69873	228.30441	

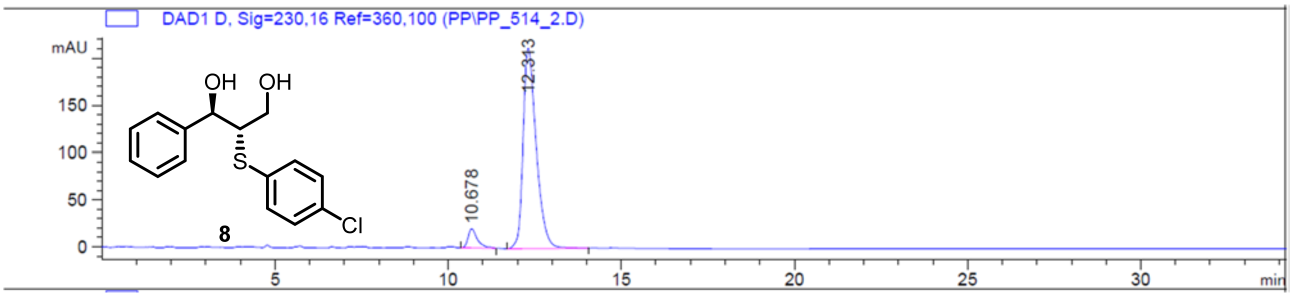

Signal 2: DAD1 D, Sig=230,16 Ref=360,100

Peak #	RetTime [min]	Type	Width [min]	Area [mAU*s]	Height [mAU]	Area %
1	10.678	BV	0.2777	386.14340	20.32258	6.4604
2	12.313	BB	0.3990	5590.95020	212.44096	93.5396
Totals :				5977.09360	232.76355	

Ritter reaction via episulfonium ion[28]

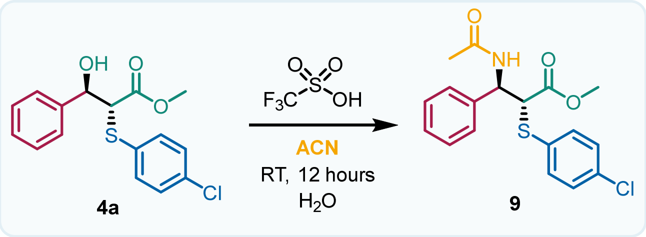

9 - Methyl (2*R*,3*R*)-3-acetamido-2-((4-chlorophenyl)thio)-3-phenylpropanoate

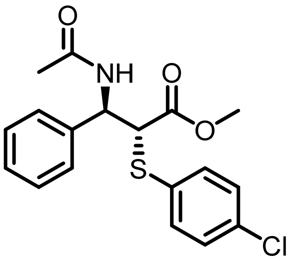

$C_{18}H_{18}ClNO_3S$

MW: 363.8 g/mol

Yield: 95%

White solid

To a solution of **4a** (0.1 mmol, 32.3 mg) in acetonitrile (500 µL) was added an aqueous solution of trifluoromethanesulfonic acid (150.0 mg, 1 mmol, 1 M). The mixture was stirred at room temperature overnight. The reaction was quenched with a saturated solution of $NaHCO_3$ and extracted three times with CH_2Cl_2. The combined organic layers were dried over anhydrous Na_2SO_4, filtered, and concentrated under vacuum. The residue was analyzed by ^{1}H NMR to determine the diastereomeric ratio (19:1). Flash chromatography (*n*-hexane/EtOAc mixtures) afforded product **9** in 95% yield (36.4 mg). The enantiomeric excess of the major diastereoisomer of the product was determined by CSP HPLC: AD-H column, *n*-hexane/iPrOH 98:2, 0.50 mL/min, t_{maj} = 17.1 min; t_{min} = 12.7 min, 85% ee, and was found to be consistent with the ee value of starting **4a**.

^{1}H NMR (600 MHz, $CDCl_3$) [signals of the major diastereoisomer] δ 7.46 – 7.41 (m, 2H), 7.33 – 7.28 (m, 4H), 7.25 – 7.21 (m, 2H), 7.20 – 7.18 (m, 2H), 5.54 (dd, *J* = 9.1, 4.5 Hz, 1H), 3.99 (d, *J* = 4.5 Hz, 1H), 3.54 (s, 3H), 2.10 (s, 3H).

^{13}C NMR (151 MHz, $CDCl_3$) [signals of the major diastereoisomer] δ 171.8, 169.8, 138.7, 135.1, 134.9, 131.2, 129.4, 128.71, 127.96, 126.2, 55.7, 54.1, 52.5, 23.4.

HRMS (MALDI$^+$) m/z calcd for $C_{25}H_{25}ClO_2SNa^+$ [M+Na]$^+$: 447.1156, found 447.1161

The relative configuration of the major diastereoisomer of compound **9** was assigned as *anti* on the basis of the stereospecificity of the mechanism of the reaction, which proceeds via an episulfonium ion intermediate[28].

[28] Procedure adapted from: A. Toshimitsu, C. Hirosawa, K. Nakano, T. Mukai, K. Tamao, *Phosphorous, Sulfur, Silicon Relat. Elem.* **1997**, *120-121*, 355–356.

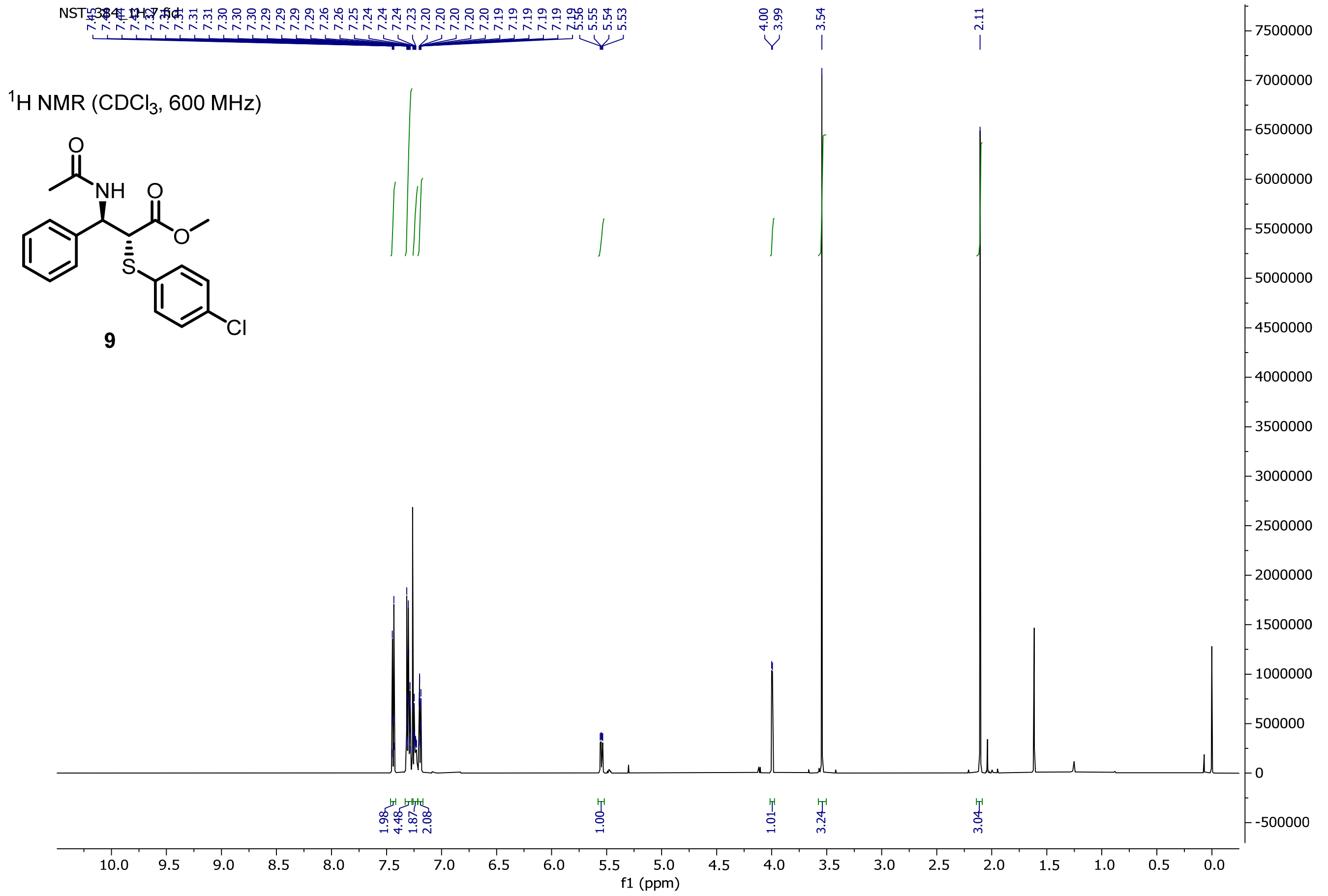
NST_384_1H.7.fid
^{1}H NMR (CDCl$_3$, 600 MHz)
9
5.56 5.55 5.54 5.53
4.00 3.99
3.54
2.11
1.98 4.48 1.87 2.08
1.00
1.01
3.24
3.04
f1 (ppm)

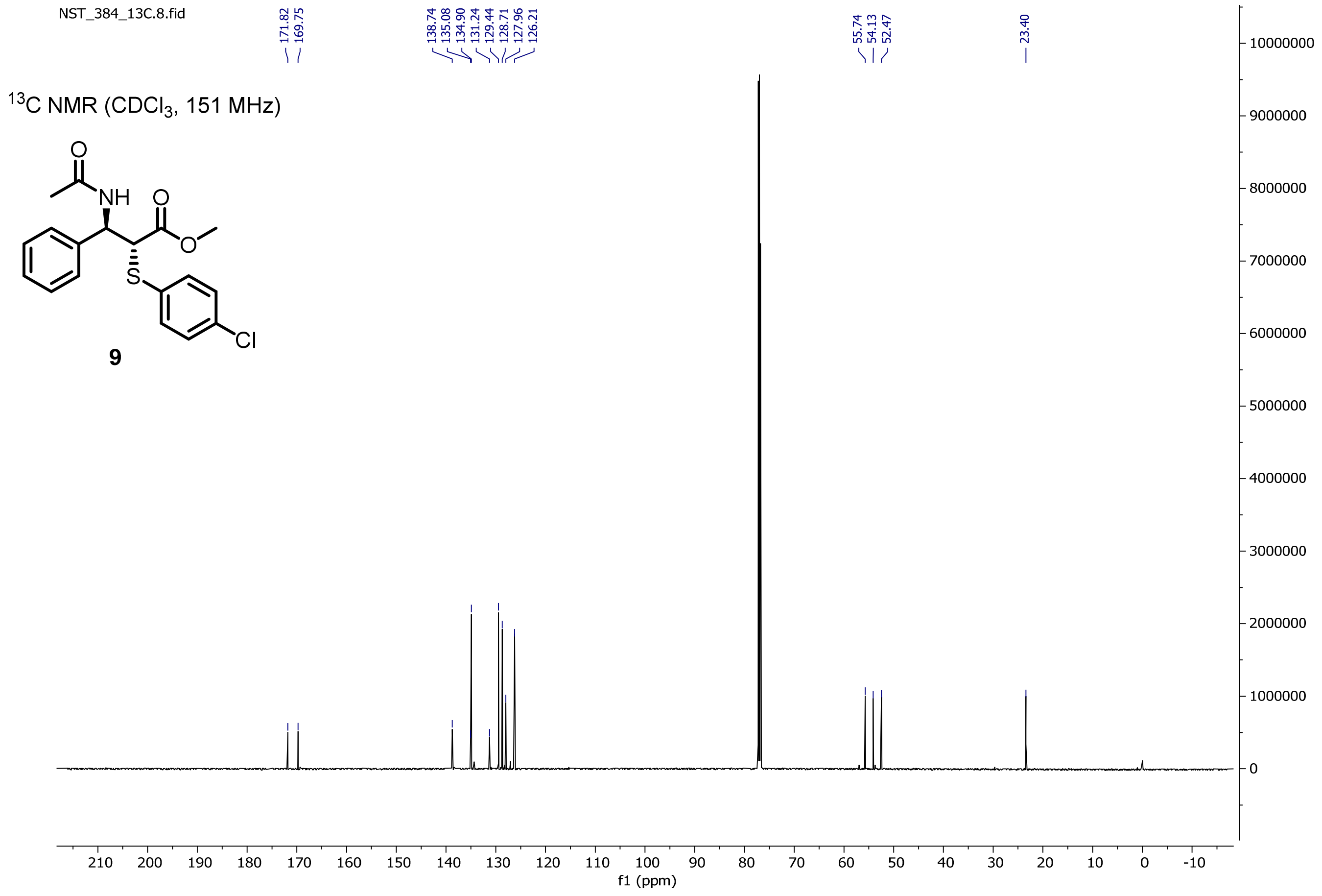
NST_384_13C.8.fid
^{13}C NMR (CDCl$_3$, 151 MHz)
9
171.82 169.75
138.74 135.08 134.90 131.24 129.44 128.71 127.96 126.21
55.74 54.13 52.47
23.40
f1 (ppm)

CSP HPLC traces:

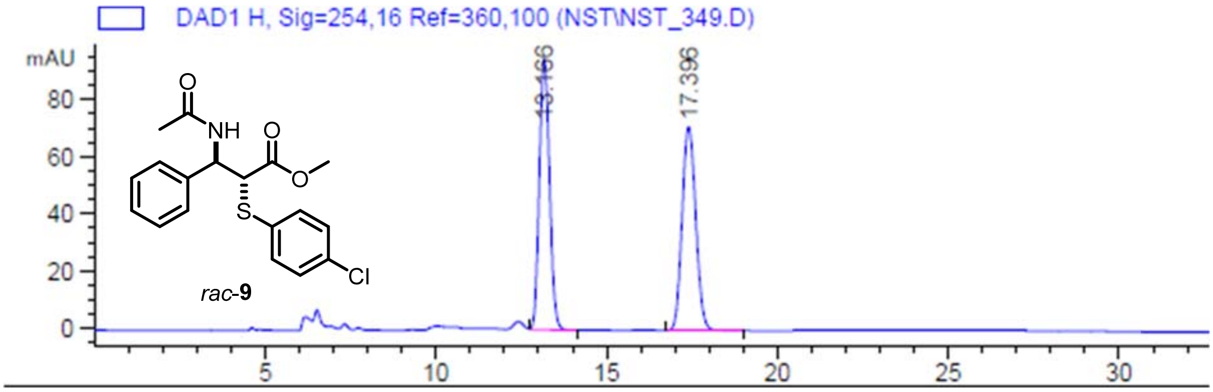

Signal 3: DAD1 H, Sig=254,16 Ref=360,100

Peak #	RetTime [min]	Type	Width [min]	Area [mAU*s]	Height [mAU]	Area %
1	13.166	VB	0.3265	1970.55737	94.80427	49.9419
2	17.396	BB	0.4377	1975.14417	71.17683	50.0581

Totals : 3945.70154 165.98110

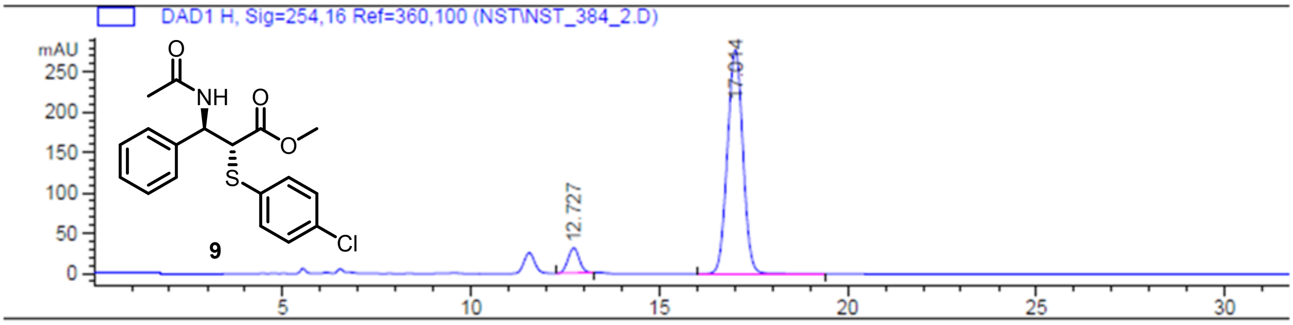

Signal 3: DAD1 H, Sig=254,16 Ref=360,100

Peak #	RetTime [min]	Type	Width [min]	Area [mAU*s]	Height [mAU]	Area %
1	12.727	BB	0.3260	644.06415	31.04861	7.4999
2	17.014	BB	0.4474	7943.62158	277.97388	92.5001

Totals : 8587.68573 309.02249

Friedel-Crafts reaction with mesitylene[29]

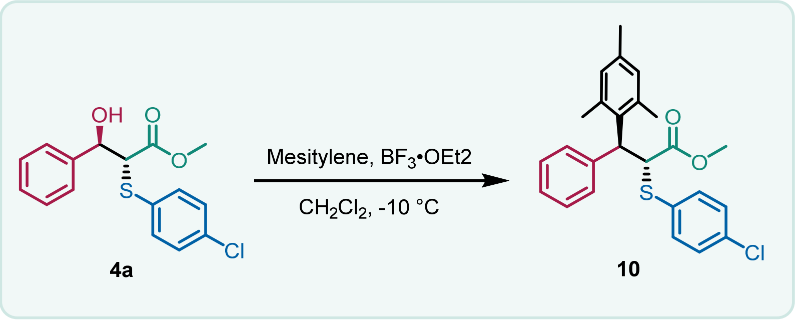

10 - Methyl (2*R*,3*S*)-2-((4-chlorophenyl)thio)-3-mesityl-3-phenylpropanoate

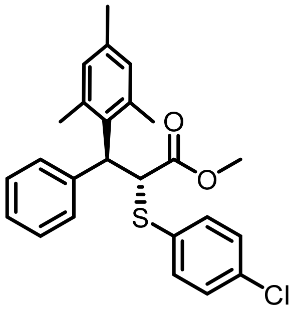

$C_{25}H_{25}ClO_2S$

MW: 425.0 g/mol

Yield: 84%

White solid

To a solution of **4a** (0.1 mmol, 1 equiv., 32.3 mg) in DCM (1 mL) at -10 °C, 1,3,5-trimethylbenzene (0.11 mmol, 1.1 equiv., 15.3 µL) and $BF_3 \cdot OEt_2$ (0.2 mmol, 2 equiv., 200 µL) were added. After 2 h, the reaction was quenched with water and extracted with CH_2Cl_2 (3 x 5 mL). The organic phases were collected, dried over Na_2SO_4, filtered and evaporated under reduced pressure. The residue was analyzed by ^{1}H NMR to determine the diastereomeric ratio (> 20:1). Flash chromatography (*n*-hexane/EtOAc mixtures) afforded product **10** as a white solid in 84% yield (35.8 mg). The enantiomeric excess of the product was determined by CSP HPLC: AD-H column, *n*-hexane/iPrOH 98:2, 0.50 mL/min, t_{maj} = 17.1 min; t_{min} = 15.1 min, 90% ee, and was found to be consistent with the ee value of starting **4a**.

^{1}H NMR (600 MHz, $CDCl_3$) δ 7.41 – 7.36 (m, 2H), 7.32 – 7.29 (m, 2H), 7.28 – 7.25 (m, 4H), 7.23 – 7.15 (m, 1H), 6.83 (br s, 1H), 6.64 (br s, 1H), 4.79 (d, *J* = 11.5 Hz, 1H), 4.73 (d, *J* = 11.5 Hz, 1H), 3.31 (s, 3H), 2.42 (s, 3H), 2.19 (s, 3H), 2.01 (s, 3H) [the spectrum shows some broad signals due to the presence of slowly interconverting rotamers].

^{13}C NMR (151 MHz, $CDCl_3$) δ 171.3, 139.4, 137.3 (br), 137.0 (br), 136.6, 135.6, 134.5, 131.4, 131.0 (br), 129.2, 128.2, 128.0, 126.1, 51.9, 51.8, 44.7, 21.5 (br), 20.7, 20.6 (br) [the spectrum shows some broad signals due to the presence of slowly interconverting rotamers].

HRMS (MALDI$^+$) m/z calcd for $C_{25}H_{25}ClO_2SNa^+$ $[M+Na]^+$: 447.1156, found 447.1161

$[\alpha]_D^{RT}$ = -81.6 (c = 1.0 g/100 mL, $CHCl_3$).

The relative configuration of compound **10** was assigned as *anti* on the basis of the stereospecificity of the mechanism of the reaction, which proceeds via an episulfonium ion intermediate[29].

[29] Procedure adapted from: A. Toshimitsu, C. Hirosawa, K. Nakano, T. Mukai, K. Tamao, *Phosphorous, Sulfur, Silicon Relat. Elem.* **1997**, *120-121*, 355–356.

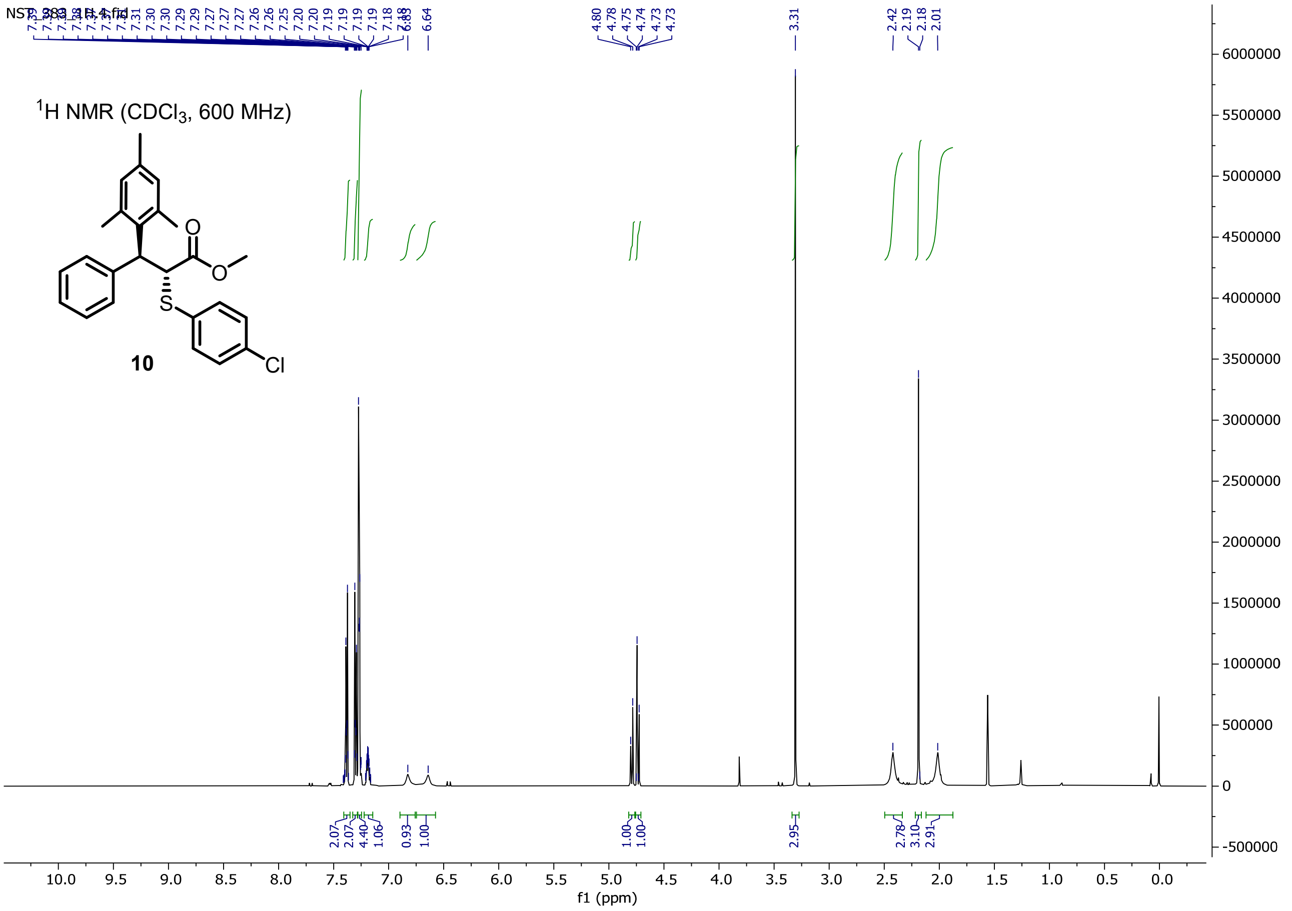

1H NMR (CDCl3, 600 MHz)
10
Cl
4.80
4.78
4.75
4.74
4.73
4.73
3.31
2.42
2.19
2.18
2.01
6.64
2.07
2.07
4.40
1.06
0.93
1.00
1.00
1.00
2.95
2.78
3.10
2.91
f1 (ppm)

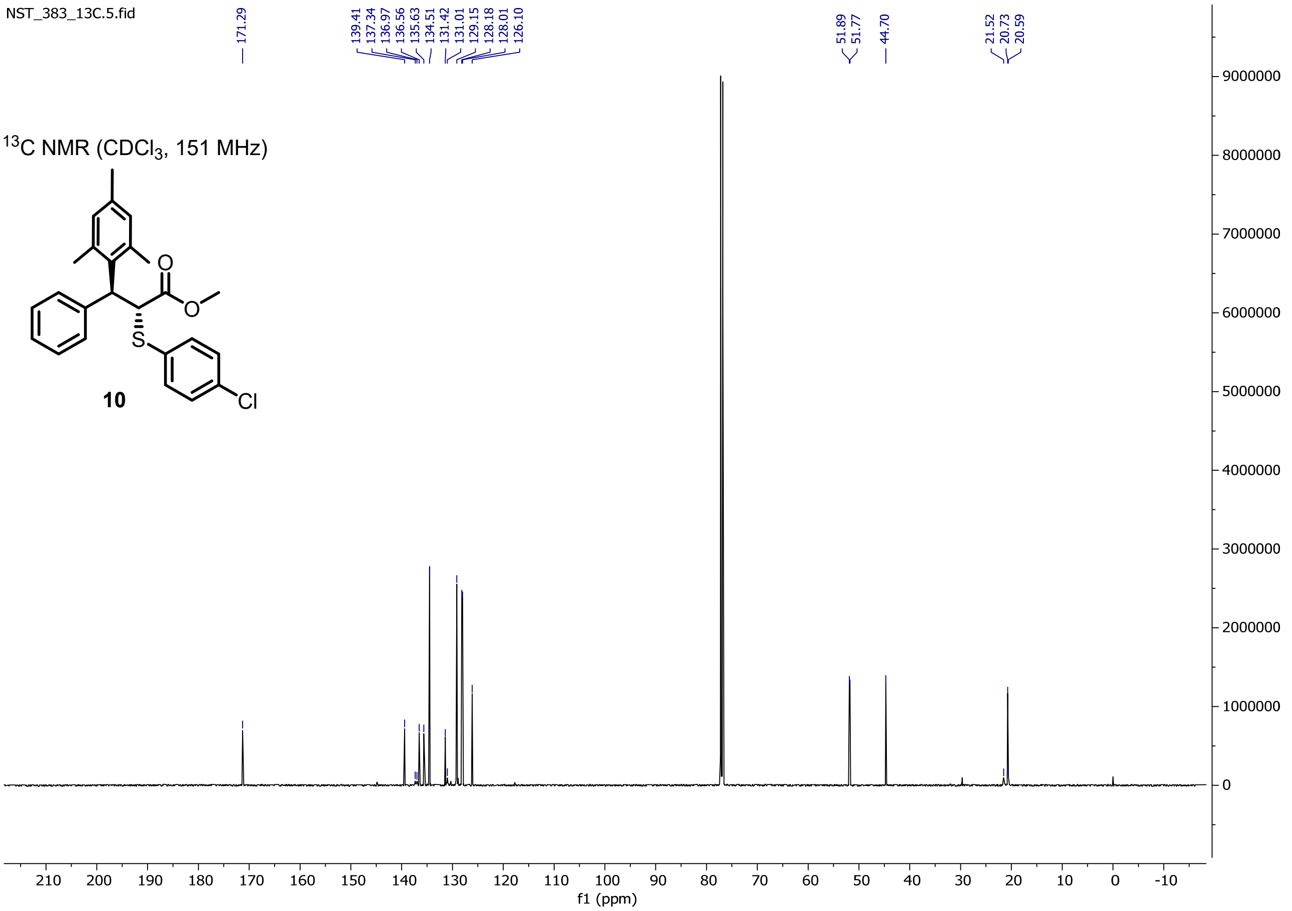

NST_383_13C.5.fid
13C NMR (CDCl3, 151 MHz)
10
Cl
171.29
139.41
137.34
136.97
136.56
135.63
134.51
131.42
131.01
129.15
128.18
128.01
126.10
51.89
51.77
44.70
21.52
20.73
20.59
f1 (ppm)

CSP HPLC traces:

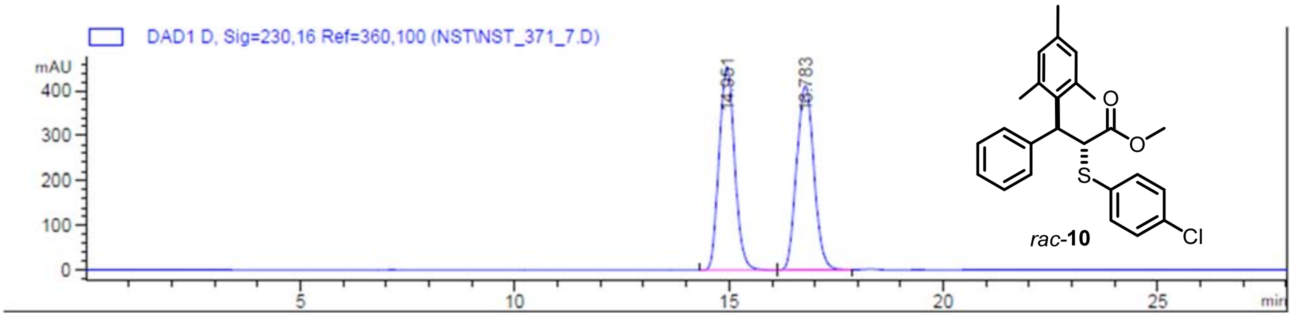

Signal 2: DAD1 D, Sig=230,16 Ref=360,100

Peak #	RetTime [min]	Type	Width [min]	Area [mAU*s]	Height [mAU]	Area %
1	14.951	BB	0.4032	1.14784e4	453.61710	49.8725
2	16.783	BB	0.4497	1.15371e4	410.72720	50.1275
Totals :				2.30156e4	864.34430	

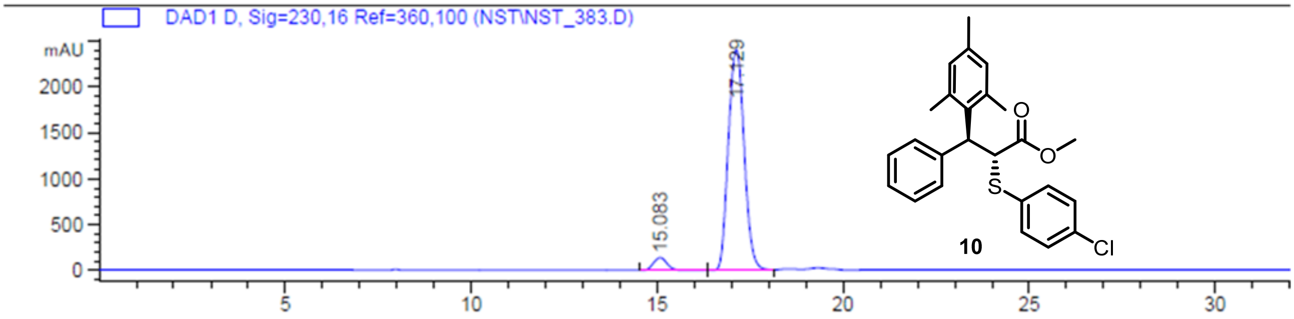

Signal 2: DAD1 D, Sig=230,16 Ref=360,100

Peak #	RetTime [min]	Type	Width [min]	Area [mAU*s]	Height [mAU]	Area %
1	15.083	BB	0.4055	3582.97559	140.52238	4.7845
2	17.129	BV	0.4781	7.13042e4	2403.87891	95.2155
Totals :				7.48872e4	2544.40129	

X-ray data for compound 4ab

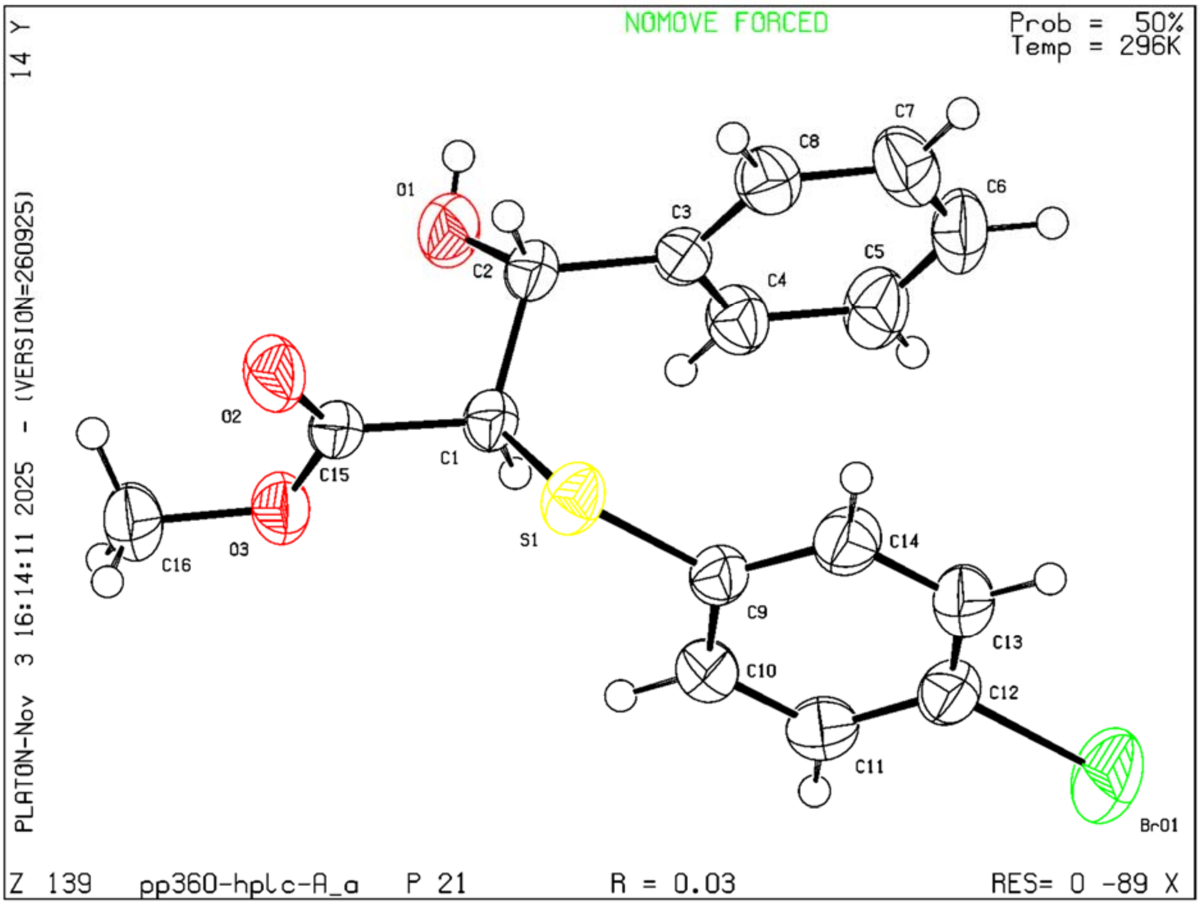

A specimen of $C_{16}H_{15}BrO_3S$, approximate dimensions 0.400 mm x 0.400 mm x 0.450 mm, was used for the X-ray crystallographic analysis. The crystals were obtained from a CH_2Cl_2/*n*-hexane mixture as solvent. The X-ray intensity data were measured (λ = 0.71073 Å). The integration of the data using a monoclinic unit cell yielded a total of 10523 reflections to a maximum θ angle of 25.99° (0.81 Å resolution), of which 3061 were independent (average redundancy 3.438, completeness = 98.7%, R_{int}= 4.17%, R_{sig} = 5.01%) and 2917 (95.30%) were greater than 2σ(F^2). The final cell constants of a = 8.844(2) Å, b = 5.5769(12) Å, c = 16.324(3) Å, β = 97.296(6)°, volume = 798.6(3) Å^3, are based upon the refinement of the XYZ-centroids of reflections above 20 σ(I). The calculated minimum and maximum transmission coefficients (based on crystal size) are 0.3750 and 0.4100. The structure was solved and refined using the Bruker SHELXTL Software Package, using the space group $P2_1$, with Z = 2 for the formula unit, $C_{16}H_{15}BrO_3S$. The final anisotropic full-matrix least-squares refinement on F^2 with196 variables converged at R1 = 3.23%, for the observed data and wR2 = 8.58% for all data. The goodness-of-fit was 1.051. The largest peak in the final difference electron density synthesis was 0.340 e$^-$/Å^3 and the largest hole was -0.444 e$^-$/Å^3 with an RMS deviation of 0.046 e$^-$/Å^3. On the basis of the final model, the calculated density was 1.527 g/cm^3 and F(000), 372 e$^-$. Flack parameter for (1*R*,2*R*) configuration was 0.05(5).

The structure was deposited in the CCDC/FIZ Karlsruhe deposition service, with deposition number CCDC 2500315.

*Table S8. Sample and crystal data for **4ab**.*

**Identification code**	**4ab**	
**Chemical formula**	$C_{16}H_{15}BrO_3S$	
**Formula weight**	367.25 g/mol	
**Temperature**	296(2) K	
**Wavelength**	0.71073 Å	
**Crystal size**	0.400 x 0.400 x 0.450 mm	
**Crystal system**	monoclinic	
**Space group**	P 1 21 1	
**Unit cell dimensions**	a = 8.844(2) Å	α = 90°
	b = 5.5769(12) Å	β = 97.296(6)°
	c = 16.324(3) Å	γ = 90°
**Volume**	798.6(3) $Å^3$	
**Z**	2	
**Density (calculated)**	1.527 g/cm^3	
**Absorption coefficient**	2.710 mm^{-1}	
**F(000)**	372	

*Table S9. Data collection and structure refinement for **4ab**.*

**Theta range for data collection**	2.32 to 25.99°	
**Index ranges**	-10<=h<=10, -6<=k<=6, -20<=l<=20	
**Reflections collected**	10523	
**Independent reflections**	3061 [R(int) = 0.0417]	
**Max. and min. transmission**	0.4100 and 0.3750	
**Structure solution technique**	direct methods	
**Structure solution program**	SHELXT 2014/5 (Sheldrick, 2014)	
**Refinement method**	Full-matrix least-squares on F^2	
**Refinement program**	SHELXL-2017/1 (Sheldrick, 2017)	
**Function minimized**	$\Sigma w(F_o^2 - F_c^2)^2$	
**Data / restraints / parameters**	3061 / 1 / 196	
**Goodness-of-fit on F^2**	1.051	
**Final R indices**	2917 data; I>2σ(I)	R1 = 0.0323, wR2 = 0.0852
	all data	R1 = 0.0336, wR2 = 0.0858
**Weighting scheme**	$w=1/[\sigma^2(F_o^2)+(0.0350P)^2+0.1662P]$<br>where $P=(F_o^2+2F_c^2)/3$	
**Absolute structure parameter**	0.050(5)	
**Extinction coefficient**	0.0710(110)	
**Largest diff. peak and hole**	0.340 and -0.444 $eÅ^{-3}$	
**R.M.S. deviation from mean**	0.046 $eÅ^{-3}$	

Table S10. *Atomic coordinates and equivalent isotropic atomic displacement parameters (Å^2) for **4ab**.*

U(eq) is defined as one third of the trace of the orthogonalized U_{ij} tensor.

	**x/a**	**y/b**	**z/c**	**U(eq)**
Br01	0.18350(6)	0.39687(15)	0.42792(3)	0.0890(3)
S1	0.56888(10)	0.74182(15)	0.76866(5)	0.0449(3)
O3	0.7750(3)	0.2603(5)	0.87856(16)	0.0471(6)
O2	0.7506(3)	0.6074(6)	0.94388(18)	0.0551(7)
O1	0.4395(4)	0.2882(6)	0.9371(2)	0.0629(9)
C1	0.5547(4)	0.4823(6)	0.83541(19)	0.0364(7)
C9	0.4630(4)	0.6394(6)	0.6751(2)	0.0384(7)
C15	0.7043(4)	0.4618(6)	0.89293(19)	0.0378(7)
C3	0.2682(4)	0.5011(6)	0.83306(19)	0.0364(7)
C10	0.5024(4)	0.4324(7)	0.6354(2)	0.0459(8)
C4	0.2261(4)	0.3079(6)	0.7814(2)	0.0444(8)
C2	0.4211(4)	0.4979(7)	0.8871(2)	0.0409(7)
C11	0.4181(4)	0.3588(7)	0.5627(2)	0.0479(8)
C8	0.1692(4)	0.6918(7)	0.8345(2)	0.0453(8)
C12	0.2966(4)	0.4973(8)	0.5291(2)	0.0466(8)
C14	0.3408(5)	0.7748(7)	0.6400(2)	0.0476(8)
C5	0.0870(5)	0.3101(8)	0.7313(3)	0.0551(10)
C13	0.2571(5)	0.7026(8)	0.5659(2)	0.0538(10)
C16	0.9157(5)	0.2109(9)	0.9310(3)	0.0574(10)
C6	0.9886(5)	0.4996(9)	0.7338(3)	0.0570(10)
C7	0.0283(5)	0.6896(8)	0.7855(3)	0.0569(10)

Table S11. *Bond lengths (Å) for **4ab**.*

Br01-C12	1.904(4)	S1-C9	1.781(4)
S1-C1	1.826(3)	O3-C15	1.321(4)
O3-C16	1.444(5)	O2-C15	1.197(4)
O1-C2	1.423(5)	O1-H1O	0.73(7)
C1-C15	1.527(4)	C1-C2	1.539(4)
C1-H1A	0.98	C9-C14	1.382(5)
C9-C10	1.390(5)	C3-C8	1.380(5)
C3-C4	1.390(5)	C3-C2	1.518(4)
C10-C11	1.383(5)	C10-H10	0.93
C4-C5	1.388(6)	C4-H4	0.93
C2-H2	0.98	C11-C12	1.379(6)
C11-H11	0.93	C8-C7	1.393(6)
C8-H8	0.93	C12-C13	1.358(6)

C14-C13	1.395(6)	C14-H14	0.93
C5-C6	1.373(6)	C5-H5	0.93
C13-H13	0.93	C16-H16A	0.96
C16-H16B	0.96	C16-H16C	0.96
C6-C7	1.372(7)	C6-H6	0.93
C7-H7	0.93		

***Table S12**. Bond angles (°) for **4ab**.*

C9-S1-C1	100.89(15)	C15-O3-C16	116.6(3)
C2-O1-H1O	104.(5)	C15-C1-C2	109.4(3)
C15-C1-S1	107.8(2)	C2-C1-S1	113.5(2)
C15-C1-H1A	108.7	C2-C1-H1A	108.7
S1-C1-H1A	108.7	C14-C9-C10	119.3(3)
C14-C9-S1	118.7(3)	C10-C9-S1	122.0(3)
O2-C15-O3	124.9(3)	O2-C15-C1	124.7(3)
O3-C15-C1	110.3(3)	C8-C3-C4	119.1(3)
C8-C3-C2	121.3(3)	C4-C3-C2	119.6(3)
C11-C10-C9	120.5(3)	C11-C10-H10	119.8
C9-C10-H10	119.8	C5-C4-C3	120.0(3)
C5-C4-H4	120.0	C3-C4-H4	120.0
O1-C2-C3	111.8(3)	O1-C2-C1	103.2(3)
C3-C2-C1	111.7(3)	O1-C2-H2	110.0
C3-C2-H2	110.0	C1-C2-H2	110.0
C12-C11-C10	118.9(4)	C12-C11-H11	120.6
C10-C11-H11	120.6	C3-C8-C7	120.5(4)
C3-C8-H8	119.8	C7-C8-H8	119.8
C13-C12-C11	121.8(4)	C13-C12-Br01	119.7(3)
C11-C12-Br01	118.5(3)	C9-C14-C13	120.2(3)
C9-C14-H14	119.9	C13-C14-H14	119.9
C6-C5-C4	120.5(4)	C6-C5-H5	119.8
C4-C5-H5	119.8	C12-C13-C14	119.2(4)
C12-C13-H13	120.4	C14-C13-H13	120.4
O3-C16-H16A	109.5	O3-C16-H16B	109.5
H16A-C16-H16B	109.5	O3-C16-H16C	109.5
H16A-C16-H16C	109.5	H16B-C16-H16C	109.5
C7-C6-C5	119.9(3)	C7-C6-H6	120.0
C5-C6-H6	120.0	C6-C7-C8	120.0(4)
C6-C7-H7	120.0	C8-C7-H7	120.0

***Table S13**. Torsion angles (°) for **4ab**.*

C9-S1-C1-C15	145.0(2)	C9-S1-C1-C2	-93.6(2)
C1-S1-C9-C14	124.0(3)	C1-S1-C9-C10	-58.1(3)
C16-O3-C15-O2	2.5(5)	C16-O3-C15-C1	-177.4(3)
C2-C1-C15-O2	-59.7(4)	S1-C1-C15-O2	64.2(4)
C2-C1-C15-O3	120.1(3)	S1-C1-C15-O3	-116.0(3)
C14-C9-C10-C11	-1.7(5)	S1-C9-C10-C11	-179.7(3)
C8-C3-C4-C5	0.9(5)	C2-C3-C4-C5	-179.6(3)
C8-C3-C2-O1	125.2(3)	C4-C3-C2-O1	-54.3(4)
C8-C3-C2-C1	-119.7(3)	C4-C3-C2-C1	60.8(4)
C15-C1-C2-O1	-55.8(3)	S1-C1-C2-O1	-176.3(2)
C15-C1-C2-C3	-176.0(3)	S1-C1-C2-C3	63.5(3)
C9-C10-C11-C12	1.6(6)	C4-C3-C8-C7	0.7(5)
C2-C3-C8-C7	-178.8(3)	C10-C11-C12-C13	-0.5(6)
C10-C11-C12-Br01	178.9(3)	C10-C9-C14-C13	0.7(5)
S1-C9-C14-C13	178.7(3)	C3-C4-C5-C6	-1.6(6)
C11-C12-C13-C14	-0.5(6)	Br01-C12-C13-C14	-179.9(3)
C9-C14-C13-C12	0.4(6)	C4-C5-C6-C7	0.6(6)
C5-C6-C7-C8	1.0(6)	C3-C8-C7-C6	-1.6(6)

***Table S14**. Anisotropic atomic displacement parameters ($Å^2$) for **4ab**.*

The anisotropic atomic displacement factor exponent takes the form: $-2\pi^2[h^2a^{*2}U_{11}+ ... + 2 h k a^*b^*U_{12}]$

	**U_{11}**	**U_{22}**	**U_{33}**	**U_{23}**	**U_{13}**	**U_{12}**
Br01	0.0788(4)	0.1234(5)	0.0571(3)	-0.0284(3)	-0.0215(2)	0.0065(3)
S1	0.0457(5)	0.0466(5)	0.0403(5)	-0.0001(4)	-0.0022(3)	-0.0118(4)
O3	0.0398(13)	0.0535(14)	0.0453(13)	-0.0063(11)	-0.0054(10)	0.0050(11)
O2	0.0401(14)	0.0672(17)	0.0542(16)	-0.0206(14)	-0.0087(11)	0.0028(12)
O1	0.0451(15)	0.098(3)	0.0455(16)	0.0298(16)	0.0038(13)	0.0051(15)
C1	0.0327(15)	0.0426(17)	0.0325(15)	-0.0020(13)	-0.0010(12)	-0.0031(13)
C9	0.0349(16)	0.0466(17)	0.0336(15)	0.0044(14)	0.0041(12)	-0.0032(14)
C15	0.0302(15)	0.052(2)	0.0308(14)	-0.0037(14)	0.0012(11)	-0.0018(13)
C3	0.0308(15)	0.0470(17)	0.0316(15)	0.0020(13)	0.0039(11)	-0.0047(13)
C10	0.0415(18)	0.054(2)	0.0420(17)	-0.0020(16)	0.0046(14)	0.0099(16)
C4	0.0384(17)	0.0451(19)	0.048(2)	-0.0046(14)	0.0002(14)	-0.0018(13)
C2	0.0329(16)	0.059(2)	0.0298(15)	-0.0010(14)	-0.0013(12)	-0.0021(15)
C11	0.052(2)	0.049(2)	0.0434(18)	-0.0075(17)	0.0091(15)	0.0030(17)
C8	0.0421(18)	0.0457(19)	0.0479(19)	0.0005(15)	0.0048(15)	-0.0014(14)
C12	0.0431(18)	0.061(2)	0.0355(17)	-0.0015(16)	0.0018(14)	-0.0051(16)
C14	0.056(2)	0.0446(19)	0.0418(19)	-0.0037(15)	0.0035(15)	0.0097(16)

	U_{11}	U_{22}	U_{33}	U_{23}	U_{13}	U_{12}
C5	0.048(2)	0.062(2)	0.051(2)	-0.0023(17)	-0.0082(17)	-0.0151(17)
C13	0.048(2)	0.065(3)	0.046(2)	-0.0003(19)	-0.0026(16)	0.0133(18)
C16	0.046(2)	0.070(3)	0.053(2)	0.000(2)	-0.0065(16)	0.0157(19)
C6	0.0385(19)	0.074(3)	0.054(2)	0.018(2)	-0.0104(16)	-0.0087(19)
C7	0.0410(19)	0.058(2)	0.071(3)	0.015(2)	0.0046(18)	0.0099(16)

Table S15. *Hydrogen atomic coordinates and isotropic atomic displacement parameters (Å^2) for **4ab**.*

	x/a	y/b	z/c	U(eq)
H1A	0.5418	0.3380	0.8010	0.044
H10	0.5862	0.3428	0.6580	0.055
H4	0.2911	0.1772	0.7804	0.053
H2	0.4318	0.6413	0.9221	0.049
H11	0.4428	0.2182	0.5368	0.058
H8	0.1968	0.8227	0.8684	0.054
H14	0.3144	0.9146	0.6658	0.057
H5	0.0602	0.1823	0.6959	0.066
H13	0.1752	0.7940	0.5419	0.065
H16A	0.9953	0.3106	0.9148	0.086
H16B	0.9425	0.0454	0.9256	0.086
H16C	0.9028	0.2440	0.9874	0.086
H6	-0.1049	0.4992	0.7005	0.068
H7	-0.0389	0.8169	0.7879	0.068
H1O	0.385(8)	0.304(11)	0.967(4)	0.09(2)

Table S16. *Hydrogen bond distances (Å) and angles (°) for **4ab**.*

	Donor-H	Acceptor-H	Donor-Acceptor	Angle
O1-H1O···O2	0.73(7)	2.28(7)	2.907(4)	144.(6)
C2-H2···O1	0.98	2.57	3.388(5)	141.4
O1-H1O···O2	0.73(7)	2.28(7)	2.907(4)	144.(6)
C2-H2···O1	0.98	2.57	3.388(5)	141.4
O1-H1O···O2	0.73(7)	2.28(7)	2.907(4)	144.(6)
C2-H2···O1	0.98	2.57	3.388(5)	141.4
C2-H2···O1	0.98	2.57	3.388(5)	141.4
O1-H1O···O2	0.73(7)	2.28(7)	2.907(4)	144.(6)
C2-H2···O1	0.98	2.57	3.388(5)	141.4
O1-H1O···O2	0.73(7)	2.28(7)	2.907(4)	144.(6)
C2-H2···O1	0.98	2.57	3.388(5)	141.4
O1-H1O···O2	0.73(7)	2.28(7)	2.907(4)	144.(6)

	Donor-H	Acceptor-H	Donor-Acceptor	Angle
C2-H2···O1	0.98	2.57	3.388(5)	141.4
O1-H1O···O2	0.73(7)	2.28(7)	2.907(4)	144.(6)
